# GLOBAL, REGIONAL, AND COUNTRY-SPECIFIC LIFETIME RISK OF STROKE, 1990–2016

**DOI:** 10.1056/NEJMoa1804492

**Published:** 2018-12-20

**Authors:** Valery L. Feigin, Grant Nguyen, Kelly Cercy, Catherine O. Johnson, Tahiya Alam, Priyakumari Ganesh Parmar, Amanuel Alemu Abajobir, Kalkidan Hassen Abate, Foad Abd-Allah, Ayenew Negesse Abejie, Gebre Yitayih Abyu, Zanfina Ademi, Gina Agarwal, Muktar Beshir Ahmed, Rufus Olusola Akinyemi, Rajaa Al-Raddadi, Leopold N. Aminde, Catherine Amlie-Lefond, Hossein Ansari, Hamid Asayesh, Solomon Weldegebreal Asgedom, Tesfay Mehari Atey, Henok Tadesse Ayele, Maciej Banach, Amitava Banerjee, Aleksandra Barac, Suzanne L. Barker-Collo, Till Bärnighausen, Lars Barregard, Sanjay Basu, Neeraj Bedi, Masoud Behzadifar, Yannick Béjot, Derrick A. Bennett, Isabela M. Bensenor, Derbew Fikadu Berhe, Dube Jara Boneya, Michael Brainin, Ismael Ricardo Campos-Nonato, Valeria Caso, Carlos A. Castañeda-Orjuela, Jacquelin Castillo Rivas, Ferrán Catalá-López, Hanne Christensen, Michael H. Criqui, Albertino Damasceno, Lalit Dandona, Rakhi Dandona, Kairat Davletov, Barbora de Courten, Gabrielle deVeber, Klara Dokova, Dumessa Edessa, Matthias Endres, Emerito Jose Aquino Faraon, Maryam S. Farvid, Florian Fischer, Kyle Foreman, Mohammad H. Forouzanfar, Seana L. Gall, Tsegaye Tewelde Gebrehiwot, Richard F. Gillum, Maurice Giroud, Alessandra C. Goulart, Rahul Gupta, Rajeev Gupta, Vladimir Hachinski, Randah Ribhi Hamadeh, Graeme J Hankey, Habtamu Abera Hareri, Rasmus Havmoeller, Simon I Hay, Mohamed I Hegazy, Desalegn Tsegaw Hibstu, Spencer Lewis James, Panniyammakal Jeemon, Denny John, Jost B. Jonas, Jacek Jóźwiak, Rizwan Kalani, Amit Kandel, Amir Kasaeian, Andre P. Kengne, Yousef Saleh Khader, Abdur Rahman Khan, Young-Ho Khang, Jagdish Khubchandani, Daniel Kim, Yun Jin Kim, Mika Kivimaki, Yoshihiro Kokubo, Dhaval Kolte, Jacek A. Kopec, Soewarta Kosen, Rita Krishnamurthi, G Anil Kumar, Alessandra Lafranconi, Pablo M. Lavados, Yirga Legesse, Yongmei Li, Xiaofeng Liang, Warren D. Lo, Stefan Lorkowski, Paulo A. Lotufo, Clement T. Loy, Mark T. Mackay, Mahdi Mahdavi, Azeem Majeed, Reza Malekzadeh, Deborah Carvalho Malta, Abdullah A. Mamun, Lorenzo G. Mantovani, Sheila Cristina Ouriques Martins, Kedar K. Mate, Mohsen Mazidi, Suresh Mehata, Toni Meier, Yohannes Adama Melaku, Walter Mendoza, George A. Mensah, Atte Meretoja, Haftay Berhane Mezgebe, Tomasz Miazgowski, Ted R. Miller, Norlinah Mohamed Ibrahim, Shafiu Mohammed, Ali H. Mokdad, Mahmood Moosazadeh, Andrew E. Moran, Kamarul Imran Musa, Ruxandra Irina Negoi, Minh Nguyen, Quyen Le Nguyen, Trang Huyen Nguyen, Tung Thanh Nguyen, Thanh Trung Nguyen, Dina Nur Anggraini Ningrum, Bo Norrving, Jean Jacques N. Noubiap, Martin J. O’Donnell, Andrew Toyin Olagunju, Oyere K. Onuma, Mayowa O. Owolabi, Mahboubeh Parasaeian, George C. Patton, Michael Piradov, Martin A. Pletcher, Farshad Pourmalek, V Prakash, Mostafa Qorbani, Mahfuzar Rahman, Muhammad Aziz Rahman, Rajesh Kumar Rai, Annemarei Ranta, David Rawaf, Salman Rawaf, Andre M. N. Renzaho, Stephen R. Robinson, Ramesh Sahathevan, Amirhossein Sahebkar, Joshua A. Salomon, Paola Santalucia, Itamar S. Santos, Benn Sartorius, Aletta E. Schutte, Sadaf G. Sepanlou, Azadeh Shafieesabet, Masood Ali Shaikh, Morteza Shamsizadeh, Kevin N. Sheth, Mekonnen Sisay Shiferaw, Min-Jeong Shin, Ivy Shiue, Diego Augusto Santos Silva, Eugene Sobngwi, Michael Soljak, Reed J. D. Sorensen, Luciano A. Sposato, Saverio Stranges, Rizwan Abdulkader Suliankatchi, Rafael Tabarés-Seisdedos, David Tanne, Cuong Tat Nguyen, JS Thakur, Amanda G. Thrift, David L. Tirschwell, Roman Topor-Madry, Bach Xuan Tran, Luong Thanh Tran, Thomas Truelsen, Nikolaos Tsilimparis, Stefanos Tyrovolas, Kingsley N. Ukwaja, Olalekan A. Uthman, Tommi Vasankari, Narayanaswamy Venketasubramanian, Vasiliy Victorovich Vlassov, Wenzhi Wang, Andrea Werdecker, Charles D.A. Wolfe, Gelin Xu, Yuichiro Yano, Naohiro Yonemoto, Chuanhua Yu, Zoubida Zaidi, Maysaa El Sayed Zaki, Maigeng Zhou, Boback Ziaeian, Ben Zipkin, Theo Vos, Michael Kravchenko, Mohsen Naghavi, Yuri Varakin, Chirstopher J. L. Murray, Gregory A. Roth

**Affiliations:** Institute for Health Metrics and Evaluation, University of Washington, Seattle, WA; Institute for Health Metrics and Evaluation, University of Washington, Seattle, WA; Institute for Health Metrics and Evaluation, University of Washington, Seattle, WA; Institute for Health Metrics and Evaluation, University of Washington, Seattle, WA; Institute for Health Metrics and Evaluation, University of Washington, Seattle, WA; Auckland University of Technology, Auckland, New Zealand; School of Public Health, University of Queensland, Brisbane, QLD, Australia; Jimma University, Jimma, Ethiopia; Department of Neurology, Cairo University, Cairo, Egypt; Debre Markos University, Debre Markos, Ethiopia; Mekelle University, Mekelle, Ethiopia; University of Basel, Basel, Switzerland; McMaster University, Hamilton, ONT, Canada; Department of Epidemiology, College of Health Sciences, Jimma University, Jimma, Ethiopia; University of Ibadan, Ibadan, Nigeria (R.O.A.); Newcastle University, Newcastle upon Tyne, UK; Newcastle University, Newcastle upon Tyne, UK; Joint Program of Family and Community Medicine, Jeddah, Saudi Arabia; Faculty of Medicine, the University of Queensland, Brisbane, QLD, Australia; Seattle Children’s Hospital, Seattle, WA; Health Promotion Research Center, Department of Epidemiology and Biostatistics, Zahedan University of Medical Sciences, Zahedan, Iran; Department of Medical Emergency, School of Paramedic, Qom University of Medical Sciences, Qom, Iran; Mekelle University, Mekelle, Ethiopia; Mekelle University, Mekelle, Ethiopia; Department of Epidemiology, Biostatiststics, and Occupational Health, McGill University, Montreal, QC, Canada; Department of Hypertension, Medical University of Lodz, Poland, and Polish Mother’s Memorial Hospital Research Institute, Lodz, Poland; Farr Institute of Health Informatics Research, University College London, London, UK; Faculty of Medicine, University of Belgrade, Belgrade, Serbia; School of Psychology, University of Auckland, Auckland, New Zealand; Africa Health Research Institute, Mtubatuba, South Africa; Department of Occupational and Environmental Medicine, Sahlgrenska Academy, University of Gothenburg, Gothenburg, Sweden; Stanford University, Stanford, CA; College of Public Health and Tropical Medicine, Jazan University, Jazan, Saudi Arabia; Social Determinants of Health Research Center, Lorestan University of Medical Sciences, Khorramabad, Iran, and Health Management and Economics Research Center, Iran University of Medical Sciences, Tehran, Iran; University Hospital and Medical School of Dijon, University of Burgundy, Dijon, France; Nuffield Department of Population Health, University of Oxford, Oxford, UK; University of São Paulo, São Paulo, Brazil; School of Pharmacy, Mekelle University, Mekelle, Ethiopia; Department of Public Heatlh, Debre Markos University, Debre Markos, Ethiopia; Danube-University Krems, Krems, Austriaa; Harvard T H Chan School of Public Health, Boston, MA; University of Perugia, Perugia, Italy; Colombian National Health Observatory, Instituto Nacional de Salud, Bogota, Colombia, and Epidemiology and Public Health Evaluation Group, Public Health Department, Universidad Nacional de Colombia, Bogota, Colombia; Caja Costarricense de Seguro Social, San Jose, Costa Rica, and Universidad de Costa Rica, San Pedro, Montes de Oca, Costa Rica; Department of Medicine, University of Valencia, INCLIVA Health Research Institute and CIBERSAM, Valencia, Spain; Clinical Epidemiology Program, Ottawa Hospital Research Institute, Ottawa, ON, Canada; Bispebjerg University Hospital, Copenhagen, Denmark; University of California, San Diego, La Jolla, CA; Faculty of Medicine, Eduardo Mondlane University, Maputo, Mozambique; Public Health Foundation of India, Gurugram, India; Public Health Foundation of India, Gurugram, India; School of Public Health, Kazakh National Medical University, Almaty Kazakhstan; Monash University, Melbourne, VIC, Australia, and Monash Medical Center, Clayton, VIC, Australia; The Hospital for Sick Children, University of Toronto, Toronto, ON, Canada; Department of Social Medicine, Faculty of Public Health, Medical University – Varna, Varna, Bulgaria; Haramaya University, Harrar, Ethiopia; Charité University Medicine Berlin, Berlin, Germany; College of Public Health, University of the Philippines Manila, Manila, Philippines, and Department of Health, Manila, Philippines; Department of Nutrition, Harvard T.H. Chan School of Public Health, Boston, MA, and Harvard/MGH Center on Genomics, Vulnerable Populations, and Health Disparities, Mongan Institute for Health Policy, Massachusetts General Hospital, Boston, MA; School of Public Health, Bielefeld University, Bielefeld, Germany; Imperial College London, London, UK; Seattle Genetics, Seattle, WA; University of Tasmania, Hobart, TAS, Australia; Jimma University, Jimma, Ethiopia; Howard University, Washington, DC; University Hospital of Dijon, Dijon, France; Center for Clinical and Epidemiological Research Center, Hospital Universitario, University of São Paulo, São Paulo, Brazil; West Virginia Bureau for Public Health, Charleston, WV; Eternal Heart Care Centre and Research Institute, Jaipur, India; Western University, London, ON, Canada; Arabian Gulf University, Manama, Bahrain; School of Medicine and Pharmacology, University of Western Australia, Perth, WA, Australia; Harry Perkins Institute of Medical Research, Nedlands, WA, Australia; and Western Australian Neuroscience Research Institute, Nedlands, WA, Australia; Addis Ababa University, Addis Ababa, Ethiopia; Karolinska Institutet, Stockholm, Sweden; Oxford Big Data Institute, Li Ka Shing Centre for Health Information and Discovery, University of Oxford, Oxford, UK; Faculty of Medicine, Cairo University, Cairo, Egypt; College of Medicine and Health Sciences, Hawassa University, Hawassa, Ethiopia; Institute for Health Metrics and Evaluation, University of Washington, Seattle, WA; Centre for Chronic Disease Control, New Delhi, India, and Centre for Control of Chronic Conditions, Public Health Foundation of India, Gurugram, India; Campbell Collaboration, New Delhi, India; Department of Ophthalmology, Medical Faculty Mannheim, Ruprecht-Karls-University Heidelberg, Mannheim, Germany; Institute of Health and Nutrition Sciences, Czestochowa University of Technology, Czestochowa, Poland; University of Washington, Seattle, WA; University at Buffalo, Buffalo, NY; Hematology-Oncology and Stem Cell Transplantation Research Center, Tehran University of Medical Sciences, Tehran, Iran, and Hematologic Malignancies Research Center, Tehran University of Medical Sciences, Tehran, Iran; South African Medical Research Council, Cape Town, South Africa; Department of Community Medicine, Public Health and Family Medicine, Jordan University of Science and Technology, Irbid, Jordan; University of Cape Town, Cape Town, South Africa; Department of Health Policy and Management, Seoul National University College of Medicine, Seoul, South Korea, and Institute of Health Policy and Management, Seoul National University Medical Center, Seoul, South Korea; Department of Nutrition and Health Science, Ball State University, Muncie, IN; Department of Health Sciences, Northeastern University, Boston, MA; School of Medicine, Xiamen University Malaysia Campus, Sepang, Malaysia; Department of Epidemiology and Public Health, University College London, London, UK; Clinicum, Faculty of Medicine, University of Helsinki, Helsinki, Finland; Department of Preventive Cardiology, National Cerebral and Cardiovascular Center, Suita, Japan; Division of Cardiology, Brown University, Providence, RI; University of British Columbia, Vancouver, BC, Canada; Center for Community Empowerment, Health Policy and Humanities, National Institute of Health Research & Development, Jakarta, Indonesia; National Institute for Stroke and Applied Neurosciences (V.L.F.); Auckland University of Technology, Auckland, New Zealand; Public Health Foundation of India, Gurugram, India; University of Milano Bicocca, Monza, Italy; Servicio de Neurologia, Clinica Alemana, Universidad del Desarrollo, Santiago, Chile; Debre Markos University, Debre Markos, Ethiopia (A.N.A.); Mekelle University, Mekelle, Ethiopia; San Francisco VA Medical Center, San Francisco, CA; Chinese Center for Disease Control and Prevention, Beijing, China; Departments of Pediatrics and Neurology, Ohio State University, Columbus, OH, and Nationwide Children’s Hospital, Columbus, OH; Institute of Nutrition, Friedrich Schiller University Jena, Jena, Germany, and Competence Cluster for Nutrition and Cardiovascular Health (nutriCARD) Halle-Jena-Leipzig, Jena, Germany; University of São Paulo, São Paulo, Brazil; The University of Sydney, Sydney, NSW, Australia; Royal Children's Hospital Melbourne, Melbourne, VIC, Australia; National Institute of Health Research, Tehran University of Medical Sciences, Tehran, Iran; Erasmus University Rotterdam, Rotterdam, Netherlands; Department of Primary Care & Public Health, Imperial College London, London, UK; Digestive Diseases Research Institute, Tehran University of Medical Sciences, Tehran, Iran; Universidade Federal de Minas Gerais, Minas Gerais, Brazil; The University of Queensland, Brisbane, QLD, Australia; University of Milano Bicocca, Monza, Italy; Hospital de Clinicas de Porto Alegre, Porto Alegre, Brazil, and Hospital Moinhos de Vento, Porto Alegre, Brazil; McGill University, Montreal, QC, Canada; Department of Biology and Biological Engineering, Food and Nutrition Science, Chalmers University of Technology, Gothenburg, Sweden; Ipas Nepal, Kathmandu, Nepal; Competence Cluster for Nutrition and Cardiovascular Health (nutriCARD), Martin Luther University Halle-Wittenberg, Halle (Saale), Germany; School of Public Health, Mekelle University, Mekelle, Ethiopia; United Nations Population Fund, Lima, Peru; Center for Translation Research and Implementation Science, National Heart, Lung, and Blood Institute, National Institutes of Health, Bethesda, MD; Department of Medicine, University of Melbourne, Melbourne, VIC, Australia; Department of Neurology, Helsinki University Hospital, Helsinki, Finland; Mekelle University, Mekelle, Ethiopia; Pomeranian Medical University, Szczecin, Poland; Pacific Institute for Research & Evaluation, Calverton, MD; School of Public Health, Curtin University, Perth, WA, Australia; Department of Medicine, Universiti Kebangsaan Malaysia Medical Center, Bandar Tun Razak, Malaysia; Health Systems and Policy Research Unit, Ahmadu Bello University, Zaria, Nigeria; Institute for Health Metrics and Evaluation, University of Washington, Seattle, WA; Health Science Research Center, Addiction Institute, Mazandaran University of Medical Sciences, Sari, Iran; Columbia University, New York, NY; School of Medical Sciences, University of Science Malaysia, Kubang Kerian, Malaysia; Carol Davila University of Medicine and Pharmacy, Bucharest, Romania; Institute for Health Metrics and Evaluation, University of Washington, Seattle, WA; Institute for Global Health Innovations, Duy Tan University, Da Nang, Vietnam; Institute for Global Health Innovations, Duy Tan University, Da Nang, Vietnam; Institute for Global Health Innovations, Duy Tan University, Da Nang, Vietnam; Institute for Global Health Innovations, Duy Tan University, Da Nang, Vietnam; Department of Public Health, Semarang State University, Semarang City, Indonesia; Skane University Hospital, Department of Clinical Sciences Lund, Neurology, Lund, Sweden; Medical Diagnostic Centre, Yaoundé, Cameroon; National University of Ireland Galway, Galway, Ireland; Discipline of Psychiatry, School of Medicine, University of Adelaide, Adelaide, SA, Australia; Department of Psychiatry, College of Medicine, University of Lagos, Lagos, Nigeria; and Department of Psychiatry, Lagos University Teaching Hospital, Lagos, Nigeria; World Health Organization, Geneva, Switzerland; Department of Medicine, University of Ibadan, Ibadan, Nigeria, and Blossom Specialist Medical Center, Ibadan, Nigeria; Non-Communicable Diseases Research Center, Tehran University of Medical Sciences, Tehran, Iran; Department of Epidemiology and Biostatistics, School of Public Health, Tehran University of Medical Sciences, Tehran, Iran; Murdoch Childrens Research Institute, Department of Paediatrics, University of Melbourne, Melbourne, VIC, Australia; Research Center of Neurology, Moscow, Russia; Institute for Health Metrics and Evaluation, University of Washington, Seattle, WA; University of British Columbia, Vancouver, BC, Canada; Charotar University of Science and Technology, Anand, India; Non-Communicable Diseases Research Center, Alborz University of Medical Sciences, Karaj, Iran; BRAC, Dhaka, Bangladesh; Austin Clinical School of Nursing, La Trobe University, Melbourne, VIC, Australia; Society for Health and Demographic Surveillance, Suri, India; University of Otago, Wellington, New Zealand; WHO Collaborating Centre, Imperial College London, London, UK; North Hampshire Hospitals, Basingstroke, UK; University College London Hospitals, London, UK; Imperial College London, London, UK; Western Sydney University, Penrith, NSW, Australia; RMIT University, Bundoora, VIC, Australia; Ballarat Health Services, Ballarat, VIC, Australia; Florey Institute of Neuroscience and Mental Health, Parkville, VIC, Australia; Mashhad University of Medical Sciences, Mashhad, Iran; University of Western Australia, Perth, WA, Australia; Department of Global Health and Population, Harvard T H Chan School of Public Health, Boston, MA; Department of Global Health and Population, Harvard T H Chan School of Public Health, Boston, MA ; Foundation IRCCS Maggiore Hospital Policlinico, Milan, Italy; Istituto di Ricerche Farmacologiche Mario Negri, Milan, Italy; Internal Medicine Department, University of São Paulo, São Paulo, Brazil; Public Health Medicine, School of Nursing and Public Health, University of KwaZulu- Natal, Durban, South Africa; Hypertension in Africa Research Team (HART), North-West University, Potchefstroom, South Africa; South African Medical Research Council, Potchefstroom, South Africa; Digestive Diseases Research Institute, Tehran University of Medical Sciences, Tehran, Iran; Department of Rehabilitation Medicine, New York University Langone Medical Center, New York, NY; Independent Consultant, Karachi, Pakistan; Department of Medical Surgical Nursing, School of Nursing and Midwifery, Hamadan University of Medical Sciences, Hamadan, Iran; School of Medicine, Yale University, New Haven, CT; Haramaya University, Harar, Ethiopia; Department of Public Health Sciences, Korea University, Seoul, South Korea; Institut für Medizinische Epidemiologie, Biometrie und Informatik, Martin-Luther-Universität Halle-Wittenberg, Bonn, Germany; Federal University of Santa Catarina, Florianopolis, Brazil; University of Yaoundé, Yaoundé, Cameroon; Department of Primary Care & Public Health, Imperial College London, London, UK; Institute for Health Metrics and Evaluation, University of Washington, Seattle, WA; Department of Clinical Neurological Sciences, Western University, London, ON, Canada; Department of Epidemiology & Biostatistics, Schulich School of Medicine & Dentistry, Western University, London, ON, Canada; Ministry of Health, Kingdom of Saudi Arabia, Riyadh, Saudi Arabia; Department of Medicine, University of Valencia, INCLIVA Health Research Institute and CIBERSAM, Valencia, Spain; Chaim Sheba Medical Center, Tel Hashomer, Israel; Institute for Global Health Innovations, Duy Tan University, Da Nang, Vietnam; School of Public Health, Post Graduate Institute of Medical Education and Research, Chandigarh, India; Department of Medicine, School of Clinical Sciences at Monash Health, Monash University, Melbourne, VIC, Australia; Institute for Health Metrics and Evaluation, University of Washington, Seattle, WA; Institute of Public Health, Faculty of Health Sciences, Jagiellonian University Medical College, Kraków, Poland; Faculty of Health Sciences, Wroclaw Medical University, Wroclaw, Poland; Hanoi Medical University, Hanoi, Vietnam; Institute for Global Health Innovations, Duy Tan University, Da Nang, Vietnam; Department of Neurology, Rigshospitalet, University of Copenhagen, Copenhagen, Denmark; University Heart Center of Hamburg, Hamburg, Germany; Parc Sanitari Sant Joan de Déu, Fundació Sant Joan de Déu, Universitat de Barcelona, CIBERSAM, Barcelona, Spain; Department of Internal Medicine, Federal Teaching Hospital, Abakaliki, Nigeria; Warwick Medical School, University of Warwick, Coventry, UK; UKK Institute for Health Promotion Research, Tampere, Finland; Raffles Neuroscience Center, Raffles Hospital, Singapored; National Research University Higher School of Economics, Moscow, Russia; Beijing Neurosurgical Institute, Beijing, China; Competence Center Mortality-Follow-Up of the German National Cohort, Federal Institute for Population Research, Wiesbaden, Germany; Division of Health and Social Care Research, King’s College London, London, UK; Department of Neurology, Jinling Hospital, Nanjing University School of Medicine, Nanjing, China; Department of Preventive Medicine, Northwestern University, Chicago, IL; Department of Biostatistics, School of Public Health, Kyoto University, Kyoto, Japan; Department of Epidemiology and Biostatistics, School of Public Health, Wuhan University, Wuhan, China; Global Health Institute, Wuhan University, Wuhan, China; University Hospital of Setif, Setif, Algeria (; Faculty of Medicine, Mansoura University, Mansoura, Egypt; National Center for Chronic and Noncommunicable Disease Control and Prevention, Chinese Center for Disease Control and Prevention, Beijing, China; University of California Los Angeles, Los Angeles, CA; Institute for Health Metrics and Evaluation, University of Washington, Seattle, WA; Institute for Health Metrics and Evaluation, University of Washington, Seattle, WA; Research Center of Neurology, Moscow, Russia; Institute for Health Metrics and Evaluation, University of Washington, Seattle, WA; Research Center of Neurology, Moscow, Russia; Institute for Health Metrics and Evaluation, University of Washington, Seattle, WA; Institute for Health Metrics and Evaluation, University of Washington, Seattle, WA; Department of Medicine, University of Washington, Seattle, WA

**Keywords:** stroke, lifetime risk, prevention

## Abstract

**Background:**

Lifetime stroke risk has been calculated in a limited number of selected populations. We determined lifetime risk of stroke globally and at the regional and country level.

**Methods:**

Using Global Burden of Disease Study estimates of stroke incidence and the competing risks of non-stroke mortality, we estimated the cumulative lifetime risk of ischemic stroke, hemorrhagic stroke, and total stroke (with 95% uncertainty intervals [UI]) for 195 countries among adults over 25 years) for the years 1990 and 2016 and according to the GBD Study Socio-Demographic Index (SDI).

**Results:**

The global estimated lifetime risk of stroke from age 25 onward was 24.9% (95% UI: 23.5–26.2): 24.7% (23.3–26.0) in men and 25.1% (23.7–26.5) in women. The lifetime risk of ischemic stroke was 18.3% and of hemorrhagic stroke was 8.2%. The risk of stroke was 23.5% in high SDI countries, 31.1% in high-middle SDI countries, and 13.2% in low SDI countries with UIs not overlapping for these categories. The greatest estimated risk of stroke was in East Asia (38.8%) and Central and Eastern Europe (31.7 and 31.6 %%), and lowest in Eastern Sub-Saharan Africa (11.8%). From 1990 to 2016, there was a relative increase of 8.9% in global lifetime risk.

**Conclusions:**

The global lifetime risk of stroke is approximately 25% starting at age 25 in both men and women. There is geographical variation in the lifetime risk of stroke, with particularly high risk in East Asia, Central and Eastern Europe.

## Introduction

Stroke accounts for almost 5% of all disability-adjusted life years (DALYs)^1^ and 10% of all deaths worldwide,^2^ with the bulk of this burden (over 75% of deaths from stroke and 81% of DALYs) falling on low- and middle-income countries.^3^ The total global burden of stroke is increasing^1-3^ and prevention of stroke may require an improved understanding of risk among younger individuals. Stroke prevention strategies in low and middle income countries may differ from those adopted for high-income countries due to differences in access to health care, health technologies and relative rates of stroke risk factors.^4^

Estimates of lifetime risk, the cumulative probability of someone of a given age and sex developing a disease during their remaining lifespan after accounting for competing mortality, provide a measure of disease risk.^5^ Lifetime stroke risk estimates may be useful for long-term health system planning.^6^ In addition, estimates of the lifetime risk of stroke across the age spectrum on a national level may serve as a useful summary metric for gauging the impact of stroke prevention strategies.

There are limited data on trends in the lifetime risk of stroke. Prior estimates of lifetime stroke risk have been reported in a limited number of selected populations^6-12^. Diverging trends in stroke incidence and mortality rates have been observed between developed (decreasing) and developing countries (increasing),^13^ against a background of increasing life expectancy for almost all countries.^14^

We used Global Burden of Disease (GBD) 2016 study estimates to provide global, regional, and country-specific lifetime risk of stroke in 1990 and 2016 by pathological subtype, age, sex and Socio-Demographic Index (SDI), accounting for competing risk of mortality due to all other non-stroke causes of death. The GBD is an ongoing global collaboration that uses all available epidemiological data to provide a comparative assessment of health loss across 328 causes for 195 countries and territories.

## Methods

We used estimates from the GBD 2016 study^1, 2^, of first-ever-in-a-lifetime stroke, cause- specific mortality, and all-cause mortality at the global, regional (21 GBD regions nested within 7 GBD super-regions), and national (195 countries) levels by age and sex (see Supplement Table 1 and Supplement Table 2). Analysis was performed separately for ischemic stroke and hemorrhagic stroke (intracerebral hemorrhage and non-traumatic subarachnoid hemorrhage). The GBD 2016 study used all available representative population-based data on incidence, prevalence, case fatality and mortality to produce comparable estimates of disease burden for 195 countries, by sex and 5-year age categories. Mortality was estimated using the Cause of Death Ensemble Model, which produces cause- specific smoothed mortality cause fractions over time using vital registration and verbal autopsy data as well as country-specific covariates. Incidence was estimated using DisMod- MR, a Bayesian meta-regression disease modelling tool. Details of the methods used to estimate stroke incidence and mortality have been previously published and are summarized in the Supplement.

Countries were categorized by quintiles of the GBD SDI for the year 2016.^15^ SDI is a composite indicator of development similar to the Human Development Index.^16^ SDI uses as input country-level income per capita, average educational attainment among individuals over age 15, and total fertility rate.

We estimated lifetime risk at a given age as the cumulative risk of stroke occurrence during the remaining lifetime, assuming the rates of stroke incidence, prevalence, and stroke mortality in each following 5 year age category. In this way, risk at each age represents the risk of stroke from that age onwards, conditional on survival to that age without having died or having had a nonfatal stroke. Further details of this method are provided in the Supplement. To account for the competing risks of stroke and mortality within a specific age group, we calculated the probability of stroke-deleted mortality and experiencing a stroke, then scaled the separate event probabilities to match the combined probability of having either a stroke or dying in an age group.

We calculated the lifetime risk only for people aged 25 years and older because stroke incidence rates in younger people are low and are less dependent on the modifiable risks and health systems that determine stroke burden in older populations.

Uncertainty intervals were the 2.5^th^ and 97.5^th^ percentile of the distribution for each estimate. Significance was reported when uncertainty intervals did not overlap.

## Results

### Global, regional, and national lifetime risk of stroke in 2016

In 2016, the lifetime risk of stroke globally was 24.9% (95% UI: 23.5–26.2), with large regional and between-country differences (Table 1, Supplement Table S3). The highest risk was estimated in China (39.3% [37.5–41.1]) with similarly high levels in Latvia, Bosnia and Herzegovina, Romania, Montenegro, Russia, Macedonia, and Bulgaria. Among the 21 GBD regions, East Asia (38.8% [37.0–40.6]), Central and Eastern Europe (31.7% [95% UI: 30.0– 33.3] and 31.6% [95% UI: 27.6–35.6], respectively) had the highest risk, and Eastern Sub- Saharan Africa (11.8% [95% UI: 10.9–12.8]) had the lowest risk. The risk was greatest in high-middle (31.1% [29.0–33.0]) and middle SDI countries (29.3% [27.8-30.8]), and lowest in low SDI countries (13.2% [12.3–14.2]). 

**Table 1:** Lifetime risk of stroke (LTR in %) (with 95%UI) globally and regionally (21GBDregions and 7 super regions) in 2016 and its percentage change (with 95% UI) from 1990 to 2016 by pathological type of stroke and sex

GBD super regions	GBD regions	Men		Women		Both sexes	
		LTR (95% UI)	Percentage change(95%CI) 1990-2015	LTR (95% UI)	Percentage change(95%CI) 1990-2015	LTR (95% UI)	Percentage change(95%CI) 1990-2015
Global		24.7 (23.3,26.0)	15.4 (12.5, 18.2)	25.1 (23.7, 26.5)	3.2 (0.2, 6.1)	24.9 (23.5, 26.2)	8.9 (6.2, 11.5)
High-income	Southern Latin America	17.8 (16.3, 19.3)	-14.2 (-20.4, -7.6)	20.6 (18.9, 22.3)	-14.5 (-20.7, -8.4)	19.2 (17.8, 20.5)	-14.1 (-19.0, -8.7)
	Western Europe	22.2 (20.9, 23.4)	4.2 (0.3, 8.2)	23.3 (21.9, 24.6)	-4.3 (-7.9, -0.4)	22.7 (21.4, 23.9)	-0.4 (-3.6, 3.1)
	High-income North America	22.4 (21.1, 23.7)	4.9 (1.7, 8.7)	25.1 (23.6, 26.4)	0.5 (-2.8, 3.8)	23.8 (22.4, 25.0)	2.7 (-0.3, 5.9)
	Australasia	20.9 (19.4, 22.4)	8.1 (1.1, 14.8)	23.0 (21.5, 24.7)	1.4 (-4.6, 7.9)	21.9 (20.6, 23.4)	4.7 (-0.5, 10.1)
	High-income Asia Pacific	22.2 (20.6, 23.8)	-11.4 (-16.3, -6.6)	23.5 (21.8, 25.2)	-15.1 (-19.7, -10.6)	22.8 (21.2, 24.3)	-13.5 (-17.4, -9.4)
Latin America and Caribbean	Caribbean	18.0 (16.6,19.3)	1.3 (-4.5, 6.8)	20.8 (19.3, 22.3)	-0.3 (-6.1, 5.9)	19.4 (18.0,20.7)	0.5 (-4.1, 5.4)
	Central Latin America	14.1 (13.1, 15.1)	0.0 (-4.3, 4.3)	16.4 (15.2, 17.6)	-2.4 (-6.4, 1.7)	15.2 (14.2, 16.4)	-1.3 (-4.8, 2.6)
	Tropical Latin America	18.9 (17.6, 20.2)	-10.4 (-13.9, -6.6)	19.5 (18.1, 20.9)	-15.1 (-18.8, -11.0)	19.1 (17.9,20.5)	-12.8 (-16.0, -9.2)
	Andean Latin America	15.5 (14.0, 17.0)	-0.9 (-9.0, 8.1)	17.9 (16.2, 19.6)	-0.1 (-7.8, 8.1)	16.7 (15.2,18.2)	-0.3 (-6.7, 6.5)
Sub-Saharan Africa	Central Sub-Saharan Africa	11.6 (10.6, 12.7)	12.4 (3.8, 20.8)	13.8 (12.6, 15.1)	1.4 (-6.9, 9.4)	12.8 (11.7, 13.8)	6.1 (-0.9, 12.9)
	Eastern Sub-Saharan Africa	11.2 (10.3, 12.3)	13.8 (5.4, 22.5)	12.5 (11.4, 13.6)	6.7 (-0.1, 13.9)	11.8 (10.9, 12.8)	9.8 (3.8, 16.1)
	Southern Sub-Saharan Africa	10.0 (9.2, 10.9)	-18.1 (-23.8, -12.3)	14.9 (13.7, 16.1)	-14.0 (-18.9, -9.0)	12.5 (11.6, 13.5)	-15.4 (-19.9, -11.1)
	Western Sub-Saharan Africa	13.0 (11.9, 14.2)	10.5 (2.3, 19.1)	15.8 (14.5, 17.3)	7.0 (-0.6, 15.8)	14.4 (13.3, 15.7)	7.9 (2.0, 14.4)
North Africa andMiddle East	North Africa and Middle East	19.4 (17.8, 20.9)	10.2 (4.7, 15.7)	23.1 (21.4, 24.8)	3.7 (-0.8, 8.0)	21.2 (19.6, 22.8)	6.4 (2.5, 10.5)
South Asia	South Asia	13.5 (12.5, 14.5)	15.6 (11.0, 20.0)	15.9 (14.7, 17.1)	19.6 (14.5, 24.6)	14.6 (13.6, 15.7)	17.6 (13.6, 21.3)
Southeast Asia, East Asia, and Oceania	East Asia	40.6 (38.7, 42.3)	35.9 (31.9, 39.8)	36.3 (34.5, 38.1)	20.7 (16.6, 24.4)	38.8 (37.0, 40.6)	29.7 (26.1, 33.0)
	SoutheastAsia	19.6 (18.3, 20.9)	6.9 (2.7, 11.5)	20.0 (18.8, 21.4)	14.2 (9.7, 18.9)	19.8 (18.6, 21.1)	10.4 (6.7, 14.2)
	Oceania	15.5 (13.8,17.2)	1.7 (-9.0, 13.0)	16.5 (14.6, 18.3)	1.6 (-9.2, 12.8)	16.0 (14.2, 17.6)	1.8 (-8.8, 12.7)
Central Europe, Eastern Europe, and Central Asia	Central Asia	22.7 (21.1, 24.4)	-2.4 (-8.1, 3.9)	26.1 (24.4, 27.9)	-10.8 (-15.2, -6.2)	24.4 (22.8, 25.9)	-7.7 (-11.7, -3.6)
	Eastern Europe	26.8 (22.0, 31.6)	-6.9 (-22.9, 11.0)	36.5 (31.2, 41.9)	-8.7 (-21.5, 3.7)	31.6 (27.6, 35.6)	-8.8 (-19.7, 2.7)
	Central Europe	29.8 (28.0, 31.5)	13.9 (9.2, 18.9)	33.7 (31.8, 35.5)	4.2 (-0.2, 8.7)	31.7 (30.0, 33.3)	8.7 (4.8, 12.8)

### Contribution of Non-Stroke Mortality to Lifetime Risk of Stroke

Supplement Figure S1A-C and Supplement Table S5 show the hypothetical national lifetime stroke risk if all countries experienced the average non-stroke mortality rate of high SDI countries. In such a counterfactual scenario, the lifetime risk of stroke is no longer lowest in sub-Saharan Africa. The largest increases in lifetime risk of stroke due to decreased non- stroke mortality in this hypothetical scenario were in Oceania (from 16% to 30%), sub- Saharan Africa (from 12 to 22%), and South Asia (from 15 to 21%). Smaller increases were seen for other low and middle-income countries, reflecting geographic variation in competing non-stroke mortality as a major determinant of lifetime stroke risk (Supplement Figures S9A- R).

### Lifetime risk by sex, age, and stroke type

**Figure 1:**
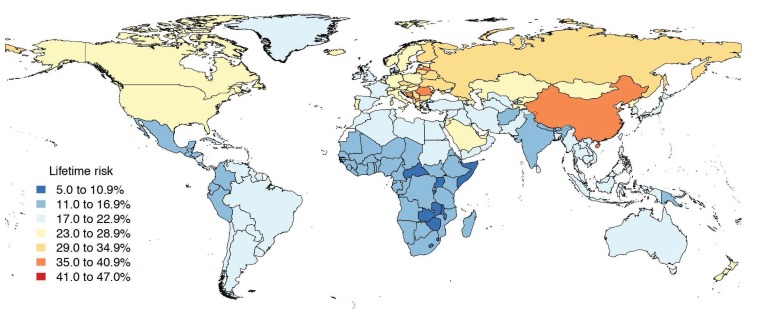
Global map showing lifetime risk of stroke occurrence (in %), both sexes combined, 2016.

In 2016, the lifetime risk of stroke in men (24.7% [95% UI 23.3–26.0]) globally was not significantly different than in women (25.1% [23.7–26.5]) (Table 1), but there were regional (Table 1; Figure 1; Supplement Figures S10A and S10B) and between-country differences in sex-specific risk. The greatest risk in men was in China (41.1% [39.2–42.9]) where there was also the largest difference between men (41.1% [39.2-42.9]) and women (36.7% [35.0-38.6]). Latvia had the greatest risk in women (41.7% [37.7–45.4]) with similar levels in Russia, Montenegro, Romania, Bosnia and Herzegovina, Lithuania, Macedonia, Bulgaria, Ukraine, Slovakia, Albania, Serbia and Belarus. Among 21 GBD regions, the highest lifetime risk in men (Table 1; Supplement Figure S2B) was in East Asia (40.6% [38.7-42.3]), while in women (Supplement Figure S2C) the highest risk was in both Eastern Europe (36.5% [31.2-41.9]) and East Asia (36.3% [34.5-38.1]).

The risk was significantly higher in women than men in Central Latin America, Southern and Western Sub-Saharan Africa, North Africa and Middle East, South Asia, and Central Europe. The lifetime risk of hemorrhagic stroke showed less variation by sex than ischemic stroke. The lifetime risk of ischemic stroke was about two times higher than the risk of hemorrhagic stroke in both men and women across different regions (Table 1) and SDI level quintiles (Supplement Table S6).

**Figure 2:**
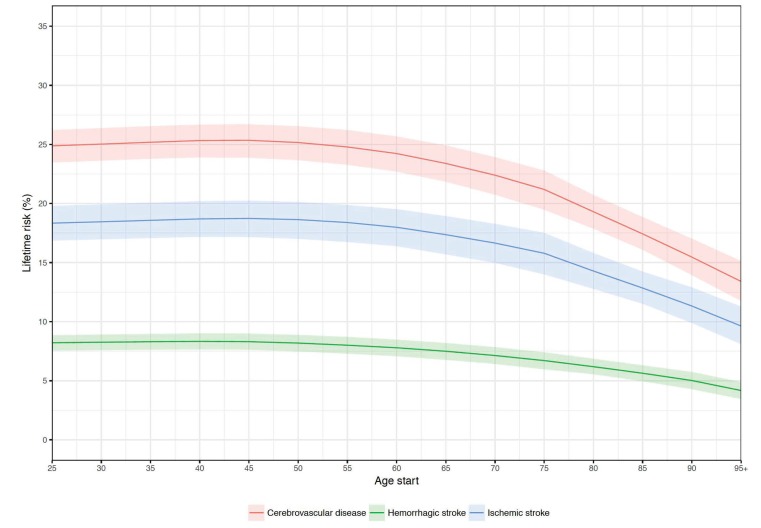
Global remaining lifetime risk of stroke occurrence (in% with 95% UI) by pathological types, age, and sex, 2016. Relationship between lifetime risk of stroke and age. Each colored line represents a trend of the relationship for the specified pathological type. The 95% confidence interval is within the shaded region surrounding each line. Modeled age starts at 25.

In 2016, the lifetime risk of total stroke was not significantly different between age 25 (24.7% [23.3-26.0]) and 70 years (22.6% [21.0-24.1]) in men, and women (25.1% [23.7-26.5] and 22.3% [20.6-23.9], respectively) (Supplement Figures S11A and S11B; Supplement Table S7). After age 70, the remaining lifetime risk decreased, reaching 13.4% (11.8–15.1) for adults aged 95 years (Figure 2).

Similar age patterns in lifetime risk were apparent for both ischemic and hemorrhagic strokes across all SDI geographies, with less decline with ageing for hemorrhagic stroke in low-middle and low SDI countries. (Supplement Figures S4-S8). The lifetime risks for ischemic and hemorrhagic separately add up to more than the total risk for all stroke because total risk is inclusive of both subtypes and represents the risk of getting either an ischemic or hemorrhagic stroke.

### Differences for lifetime risk in 1990 and 2016

Globally from 1990 to 2016, there was a significant increase in the average lifetime risk of stroke from 22% to 24%, a relative increase of 9% (Table 1; Supplement Table S4). The relative increase in the risk was greater for men (15.4% [12.5-18.2]) than women (3.2% [0.2- 6.1]), and for ischemic stroke (12.7% [8.9, 16.3]) than hemorrhagic stroke (4.0% [0.2, 7.6]). There was a significant increase in risk in Western and Eastern Sub-Saharan Africa, North Africa and Middle East, Central Europe, East Asia, South Asia and Southeast Asia. There was a significant reduction in risk in Central Asia, Southern and Tropical Latin America, high- income Asia Pacific, and Southern Sub-Saharan Africa. There were no significant changes estimated in the remaining GBD regions.

## Discussion

The global lifetime risk of stroke from age 25 onward is estimated to have increased from 22% to 24% over the past three decades, with the risk of ischemic stroke exceeding the risk of hemorrhagic stroke (18% vs 8%, respectively). This increase in risk is the result of flat or increasing stroke incidence in many middle-SDI regions with simultaneous declines in the competing risks of non-stroke mortality.

The estimated global lifetime risk of stroke declined with age, due to age-related competing risks from other diseases. In low SDI countries with the youngest populations, such as Sub- Saharan Africa, estimated lower lifetime stroke risk is the result of high competing risk of mortality at both young and old ages and does not represent substantially lower stroke incidence or more effective prevention and treatment strategies.^17, 2^, In contrast, we estimated the highest estimated lifetime stroke risks are found in East Asia, Central and Eastern Europe.

Many of our national estimated lifetime stroke risks are similar or higher compared to what was observed for specific populations in the same country, including the Framingham Heart cohort (21.1% for women and 16.9% for men ),^18^ in a Japanese cohort (18.9% for men and 20.2% for women),^8^ and in a Chinese cohort (18.0% for men and 14.7% for women).^7^ Our estimates are lower than that for women in the Netherlands (29.8%) but similar to estimates there among men (22.8%).^9^ We estimated ischemic stroke to be more frequent than hemorrhagic stroke which is comparable to the findings of other population-based studies.6, 8, 12, 13. 19

Regional variation in lifetime cardiovascular risk across subpopulations has been shown previously by the Cardiovascular Lifetime Risk Pooling Project, and support our finding of large geographic variation in total stroke risk.^10^ The greater increase in the lifetime risk of ischemic stroke compared to hemorrhagic stroke from 1990 to 2016 may be related to reduction in the incidence of hemorrhagic stroke as opposed to minor increases in the incidence of ischemic stroke over the last two decades.^3^ Although our findings of similar lifetime risk of stroke in men and women are in concordance with some other observations, there have been studies 8-10, 19 in which the risk was greater in women compared with that in men, and the reasons for these differences between studies is unclear. The Global Burden of Disease Study Comparative Risk Assessment 4, 20 estimated that elevated blood pressure was the leading attributable risk for stroke across all levels of the SDI, with greater attribution to air pollution and low fruit intake in low-SDI countries and high body-mass index and high fasting plasma glucose in high-SDI countries.

Estimates of lifetime risk of a disease is new for the GBD study, which has previously published several other summary measures of health including years of life lost prematurely, years lives with disability,^3, 21^ and stroke burden associated with various risk factors.^4^ Lifetime risk may be useful for stroke prevention and public education. High estimates of lifetime risk of stroke suggest the possible value of intensive primary stroke prevention measures throughout the lifespan and suggest that strategies to reduce cardiovascular risk remain relevant for both younger and older adults.

The main strength of our study was that we systematically evaluated the lifetime risk of using data and methods that allow for comparable estimates between location and over time. We provided estimates of the lifetime risk of stroke for people aged 25 years and over (up to age 95) as opposed to stroke lifetime risk estimates from other studies, where the risk of stroke was estimated for people aged 45 or over.^6,8-10^ Furthermore, our lifetime stroke risk estimates account for competing risk of mortality from other causes of death and represent whole populations, adding to the generalizability of these results. 

Our approach has limitations. The accuracy of lifetime stroke risk estimates was limited by the accuracy and availability of epidemiological data from the countries studied. There was still lack of sufficient epidemiological data on stroke incidence and case fatality for most countries of the world. In countries without data on stroke incidence, estimates were dependent on geospatial statistical models incorporating data from neighboring countries and country-level risk exposure data, which is widely available. The ability to differentiate stroke from other acute neurological events and to differentiate ischemic from hemorrhagic strokes was impeded by the nature of health system in each country, by the technology available to diagnose strokes, and the customary manner of coding disease entities. We did not differentiate risk due to subarachnoid hemorrhage and intracerebral hemorrhage, which were combined as an estimate of total hemorrhagic stroke. There is significant subnational variation in stroke burden within large countries and our results represent only average national risk. Standard error was increased using a standard algorithm when data from subnational regions were used to represent an entire country. Finally, we analyzed only the lifetime risk of first-ever stroke and not recurrent stroke.

In conclusion, our study provides comprehensive global, regional, and country-specific estimates of the lifetime risk of stroke by sex, age, with imprecision introduced by limited data in many countries. The global lifetime risk of stroke is approximately 25% starting at age 25 in both men and women and there is large geographical variation, with particularly high lifetime risk in East Asia, Central and Eastern Europe.

## Supplementary Materials

Supplementary Materials
