# Supplementary material for: GLOBAL, REGIONAL, AND COUNTRY-SPECIFIC LIFETIME RISK OF STROKE, 1990–2016
Source: N Engl J Med. Author manuscript; Available in PMC 2018 Dec 20. (PMC6247346; doi:10.1056/NEJMoa1804492)
Supplement: Supplementary Materials [file NEJMoa1804492_Feigin_Supplement.pdf]

Supplements: Tables and Figures

GBD 2016 Stroke Modelling Methods Summary 3

Lifetime Risk of Stroke Methods 4

Figures 5- 50

Figure S1A. Lifetime risk of stroke for males, high-SDI counterfactual, 2016 5

Figure S1B. Lifetime risk of stroke for females, high-SDI counterfactual, 2016 6

Figure S1C. Lifetime risk of stroke for both sexes, high-SDI counterfactual, 2016........ 7

Figure S2A. Lifetime risk of stroke for both sexes combined, by GBD region, 2016 8

Figure S2B. Lifetime risk of stroke for males, by GBD region, 2016 9

Figure S2C. Lifetime risk of stroke for females, by GBD region, 2016 10

Figure S3A. Global lifetime risk of stroke occurrence by cause and age for both sexes combined, 2016 11

Figure S3B. Global lifetime risk of stroke occurrence by cause and age for males, 2016 12

Figure S3C. Global lifetime risk of stroke occurrence by cause and age for females, 2016 13

Figure S4A. High SDI lifetime risk of stroke occurrence by cause and age for both sexes combined, 2016 14

Figure S4B. High SDI lifetime risk of stroke occurrence by cause and age for males, 2016 15

Figure S4C. High SDI lifetime risk of stroke occurrence by cause and age for females, 2016 16

Figure S5A. High-middle SDI lifetime risk of stroke occurrence by cause and age for both sexes combined, 2016 17

Figure S5B. High-middle SDI lifetime risk of stroke occurrence by cause and age for males, 2016........18

Figure S5C. High-middle SDI lifetime risk of stroke occurrence by cause and age for females, 2016......19

Figure S6A. Middle SDI lifetime risk of stroke occurrence by cause and age for both sexes combined, 2016 20

Figure S6B. Middle SDI lifetime risk of stroke occurrence by cause and age for males, 2016 21

Figure S6C. Middle SDI lifetime risk of stroke occurrence by cause and age for females, 2016 22

Figure S7A. Low-middle SDI lifetime risk of stroke occurrence by cause and age for both sexes combined, 2016 23

Figure S7B. Low-middle SDI lifetime risk of stroke occurrence by cause and age for males, 2016 24

Figure S7C. Low-middle SDI lifetime risk of stroke occurrence by cause and age for females, 2016 25

Figure S8A. Low SDI lifetime risk of stroke occurrence by cause and age for both sexes combined, 2016 26

Figure S8B. Low SDI lifetime risk of stroke occurrence by cause and age for males, 2016 27

Figure S8C. Low SDI lifetime risk of stroke occurrence by cause and age for females, 2016........28

Figure S9A. Incident stroke vs non-stroke mortality by age, global, both sexes, 2016 29
Figure S9B. Incident stroke vs non-stroke mortality by age, global, male, 2016....................................30
Figure S9C. Incident stroke vs non-stroke mortality by age, global, female, 2016 31
Figure S9D. Incident stroke vs non-stroke mortality by age, high SDI, both sexes, 2016....................... 32
Figure S9E. Incident stroke vs non-stroke mortality by age, high SDI, males, 2016 33
Figure S9F. Incident stroke vs non-stroke mortality by age, high SDI, females, 2016 34
Figure S9G. Incident stroke vs non-stroke mortality by age, high-middle SDI, both sexes, 2016 35
Figure S9H. Incident stroke vs non-stroke mortality by age, high-middle SDI, males, 2016 36
Figure S9I. Incident stroke vs non-stroke mortality by age, high-middle SDI, females, 2016 37
Figure S9J. Incident stroke vs non-stroke mortality by age, middle SDI, both sexes, 2016.....................38
Figure S9K. Incident stroke vs non-stroke mortality by age, middle SDI, males, 2016............................39
Figure S9L. Incident stroke vs non-stroke mortality by age, middle SDI, females, 2016.........................40
Figure S9M. Incident stroke vs non-stroke mortality by age, low-middle SDI, both sexes, 2016 41
Figure S9N. Incident stroke vs non-stroke mortality by age, low-middle SDI, males, 2016 42
Figure S9O. Incident stroke vs non-stroke mortality by age, low-middle SDI, females, 2016.................43
Figure S9P. Incident stroke vs non-stroke mortality by age, low SDI, both sexes, 2016 44
Figure S9Q. Incident stroke vs non-stroke mortality by age, low SDI, both sexes, 2016 45
Figure S9R. Incident stroke vs non-stroke mortality by age, low SDI, both sexes, 2016 46
Figure S10A. Lifetime risk of cerebrovascular disease in females, 2016 47
Figure S10B. Lifetime risk of cerebrovascular disease for males, 2016..48
Figure S11A. Global lifetime risk of stroke occurrence by cause and age in men 49
Figure S11B. Global lifetime risk of stroke occurrence by cause and age in women 50

Tables 51
Table S1. GBD 2016 location hierarchy with levels..51
Table S2. Proportion of incident stroke subtypes by location, both sexes, in 2016................................68
Table S3. Lifetime risk of stroke (LTR in %) (with 95% UI) globally and regionally (21 GBD regions and 7 super regions) in 2016 and its percentage change (with 95% UI) from 1990 to 2016 by pathological type of stroke and sex...72
Table S4. Lifetime risk of stroke in 2016, by country, region, and sex 74
Table S5. Lifetime risk of stroke counterfactual, 2016 100
Table S6. Lifetime risk of stroke in 2016, by sociodemographic index quintiles 103
Table S7. Lifetime risk of stroke in 2016, by age 104
Table S8. Citations by cause 105

GBD 2016 Stroke Modelling Methods Summary

Methods to estimate all-cause mortality and cause-specific mortality been previously described.[1] Stroke modeling and methodology used to estimate GBD 2016 stroke burden (including an updated list of publications included in this analysis) has also been described in detail.[2] In brief, we used all available estimates of stroke incidence, prevalence and case fatality from systematic reviews of the scientific literature, population surveys, and stroke registries. Stroke was defined based on the WHO clinical criteria.[3] All available national mortality data were compiled. Non-specific cause codes were redistributed based on expert opinion and statistical methods.[2] The total for all cause-specific deaths was fitted within an envelope for all-cause mortality. Deaths were compiled into 240 causes, including IS and HS. For stroke death estimates, GBD defined stroke ICD-10 codes as IS, HS, or nonspecific as to type. The parent category of cerebrovascular disease was based on the mapping of the detailed causes. Deaths coded as due to G45 (transient ischemic attack) were coded as IS and deaths coded as due to nonruptured aneurysms (ICD code I67.0) were coded as HS. Nonspecific codes, including I64, I67.9, I68.8, I69.4-I69.9, were redistributed to IS or HS using a regression model. An ensemble model was used to estimate a continuous time-series for mortality by age, sex, country (developed or developing), and year. Country-level covariates associated with stroke were used in the model and out-of-sample validity testing was used to assess model performance. 95% uncertainty intervals (UI) were estimated using 1000 draws from the posterior distribution for each age-sex-country group. Disease prevalence was estimated using DisMod state-transition disease modeling software[9] and Bayesian staistical models. IS and HS were modelled separately and combined.

1. Abajobir, A. A., et al. (2017). "Global, regional, and national age-sex specific mortality for 264 causes of death, 1980-2016: a systematic analysis for the Global Burden of Disease Study 2016." The Lancet 390(10100): 1151-1210

2. Roth, G. A., et al. (2015). "Methods for Estimating the Global Burden of Cerebrovascular Diseases." Neuroepidemiology 45(3): 146-151.

3. Aho K, Harmsen P, Hatano S, Marquardsen J, Smirnov VE, Strasser T: Cerebrovascular disease in the community: results of a WHO collaborative study. Bulletin of the World Health Organization 1980;58:113-130.

Lifetime Risk of Stroke Methods

Using all-cause mortality and stroke incidence, prevalence, and mortality, we calculated the following metrics for five-year age groups between age *i* and age *i + 4*:

Stroke incidence hazard: $hazard_i = \frac{incidence_i}{1 - prevalence_i}$

Stroke-deleted mortality rate: $mort_i = overall\ mortality_i - stroke\ mortality_i$

Using stroke incidence hazard and the stroke-deleted mortality rate, we can estimate the probability of surviving from age 25 to age a without experiencing a stroke:

$$survival_a = 1 - e^{-5 * \sum_{i=25}^{a} (mort_i + hazard_i)}$$

The probability of experiencing a stroke for five-year age groups between age *a* and age *a + 4* (for example 25-29) can be calculated from the stroke incidence hazard:

$$risk_a = 1 - e^{-5 * hazard_a}$$

Using the probability of survival from age 25 to age *a* and the probability of experiencing a stroke in each age group, we can estimate the lifetime risk of experiencing a stroke, beginning at age *i*:

$$\frac{1}{survival_i} * \sum_{a=i}^{95+} survival_a * risk_a$$

We subtracted stroke mortality from overall mortality because the eligible population for stroke deaths is limited to those who have already had a stroke, all of whom have already been excluded from the eligible population to avoid double-counting of events. To account for the competing risks of stroke and mortality within a specific age group, we calculated the probability of stroke-deleted mortality and stroke incidence hazard separately, and then scaled the separate event probabilities to match the combined probability of both events.

Figure 1A. Lifetime risk of Stroke for males, High–SDI counterfactual, 2016

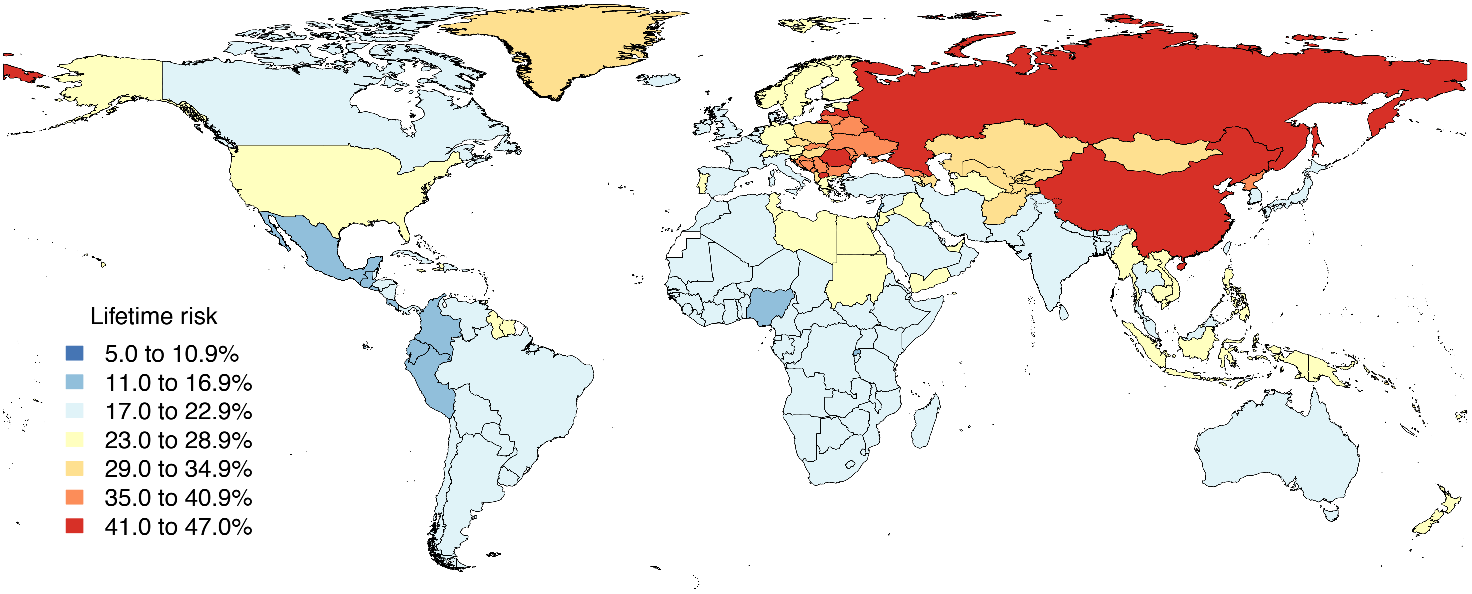

Figure 1B. Lifetime risk of Stroke for females, High–SDI counterfactual, 2016

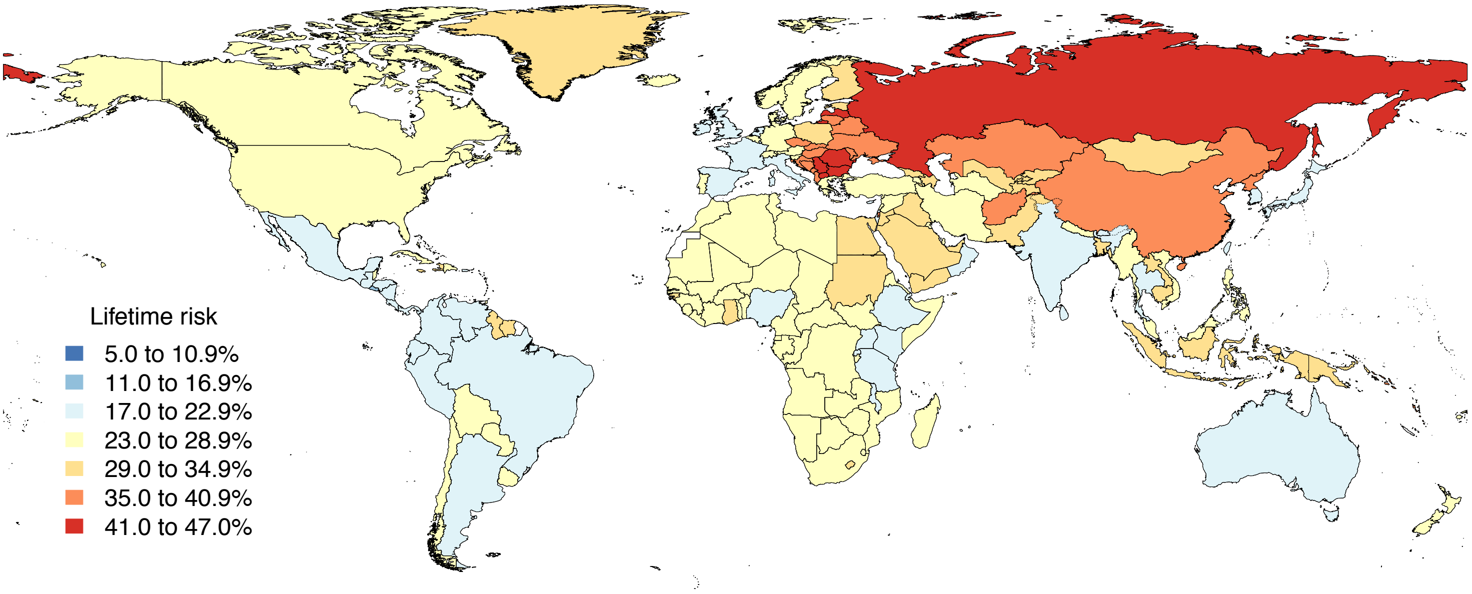

Figure 1C. Lifetime risk of Stroke for both sexes combined, High–SDI counterfactual, 2016

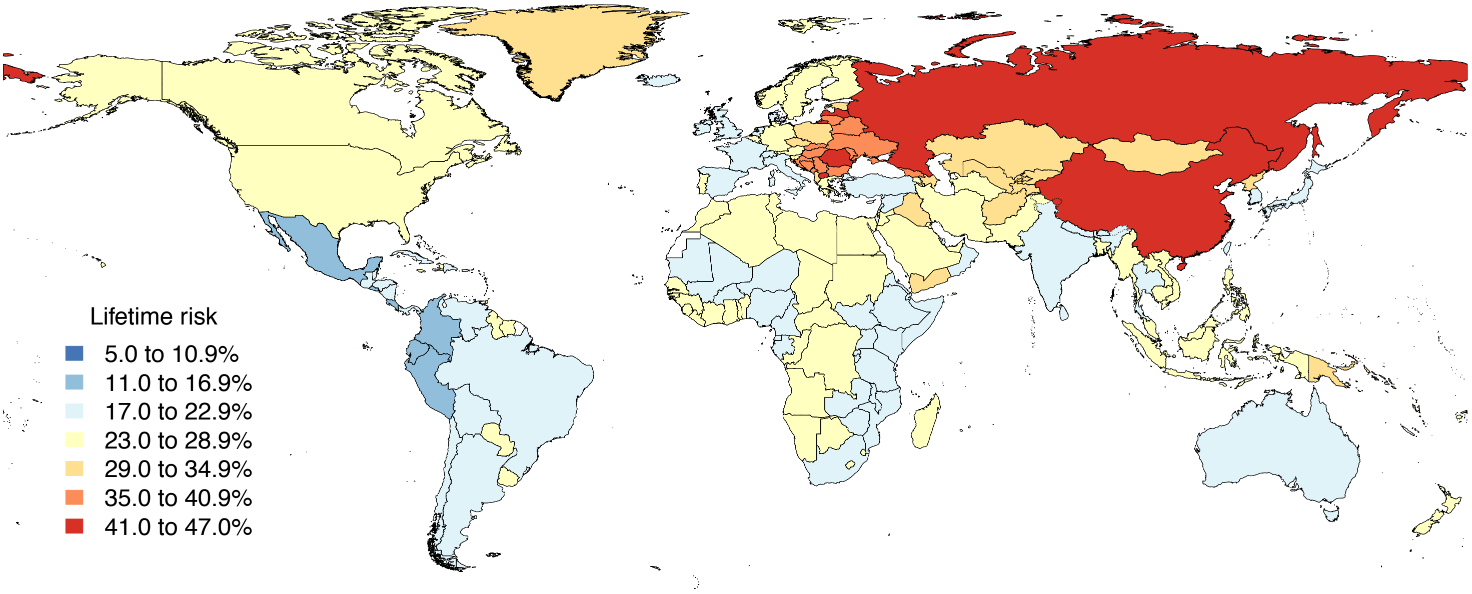

Figure S2A. Lifetime risk of Stroke for both sexes combined by GBD region, 2016.

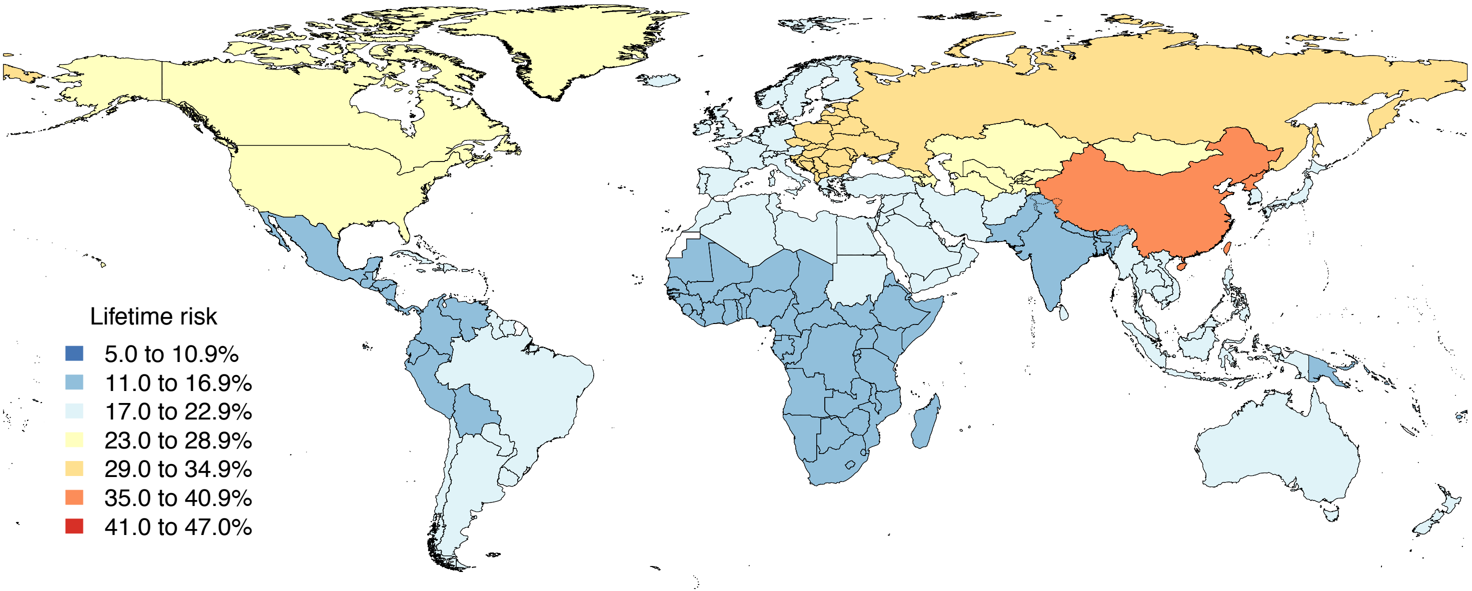

Figure S2B. Lifetime risk of Stroke for males by GBD region, 2016.

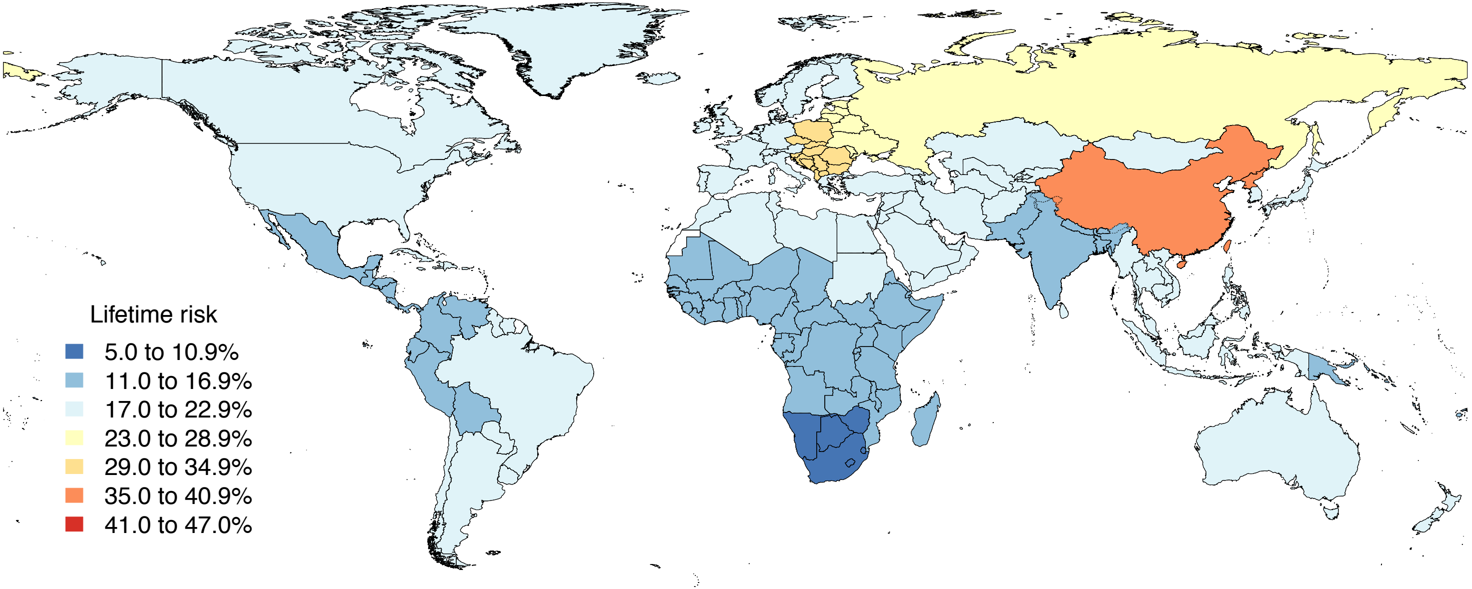

Figure S2C. Lifetime risk of Stroke for females by GBD region, 2016.

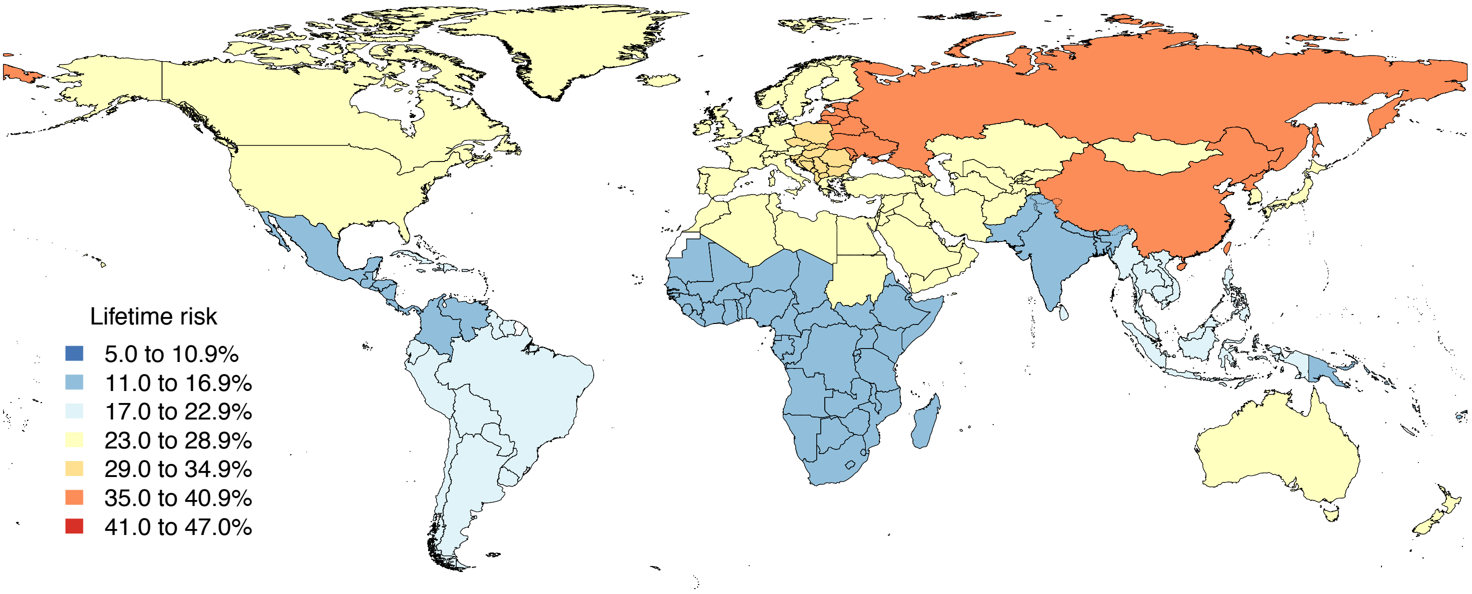

Figure 3A. Global lifetime risk of stroke occurrence by cause and age for both sexes combined, 2016.

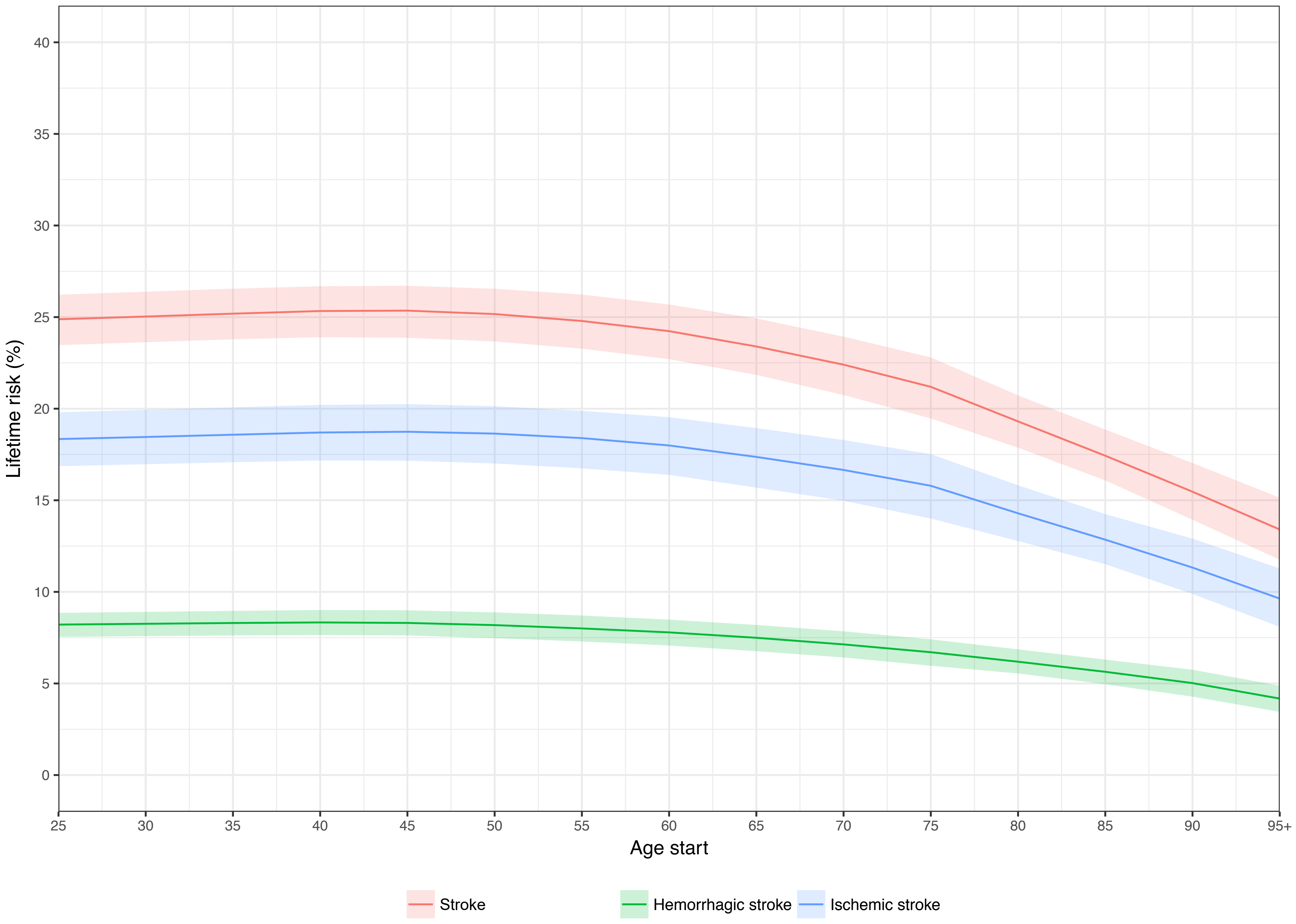

Figure 3B. Global lifetime risk of stroke occurrence by cause and age for males, 2016.

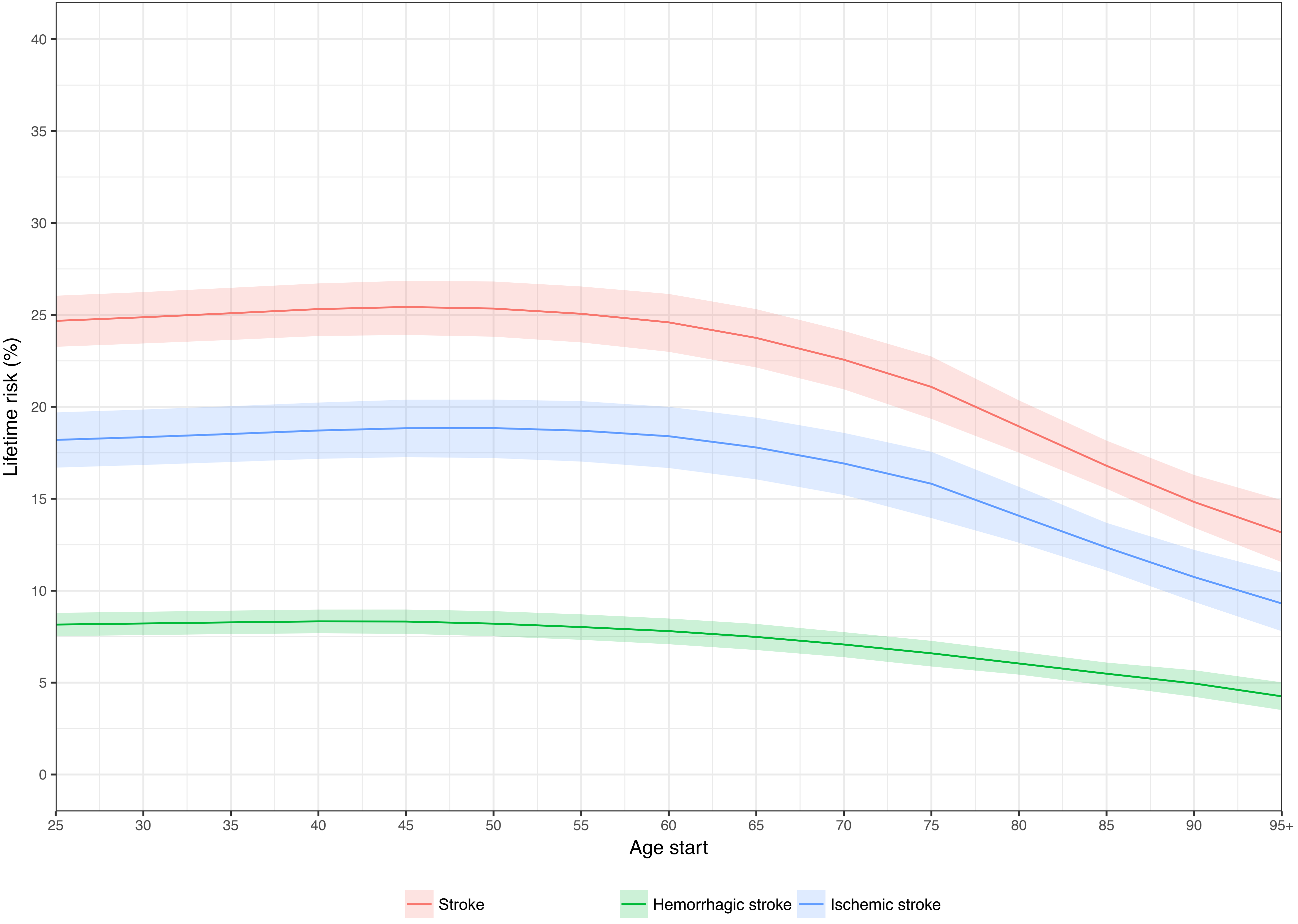

Figure 3C. Global lifetime risk of stroke occurrence by cause and age for females, 2016.

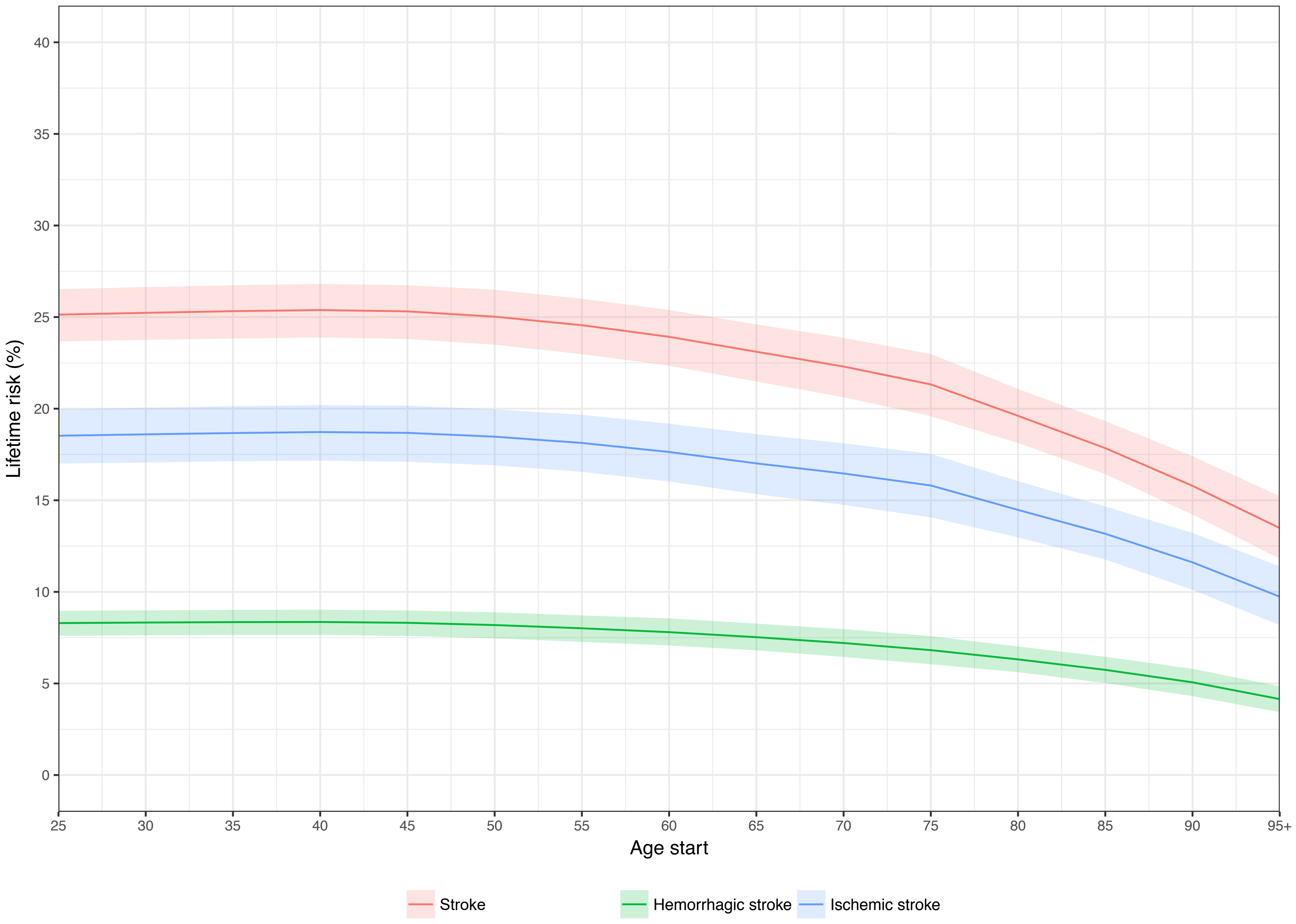

Figure 4A. High SDI lifetime risk of stroke occurrence by cause and age for both sexes combined, 2016.

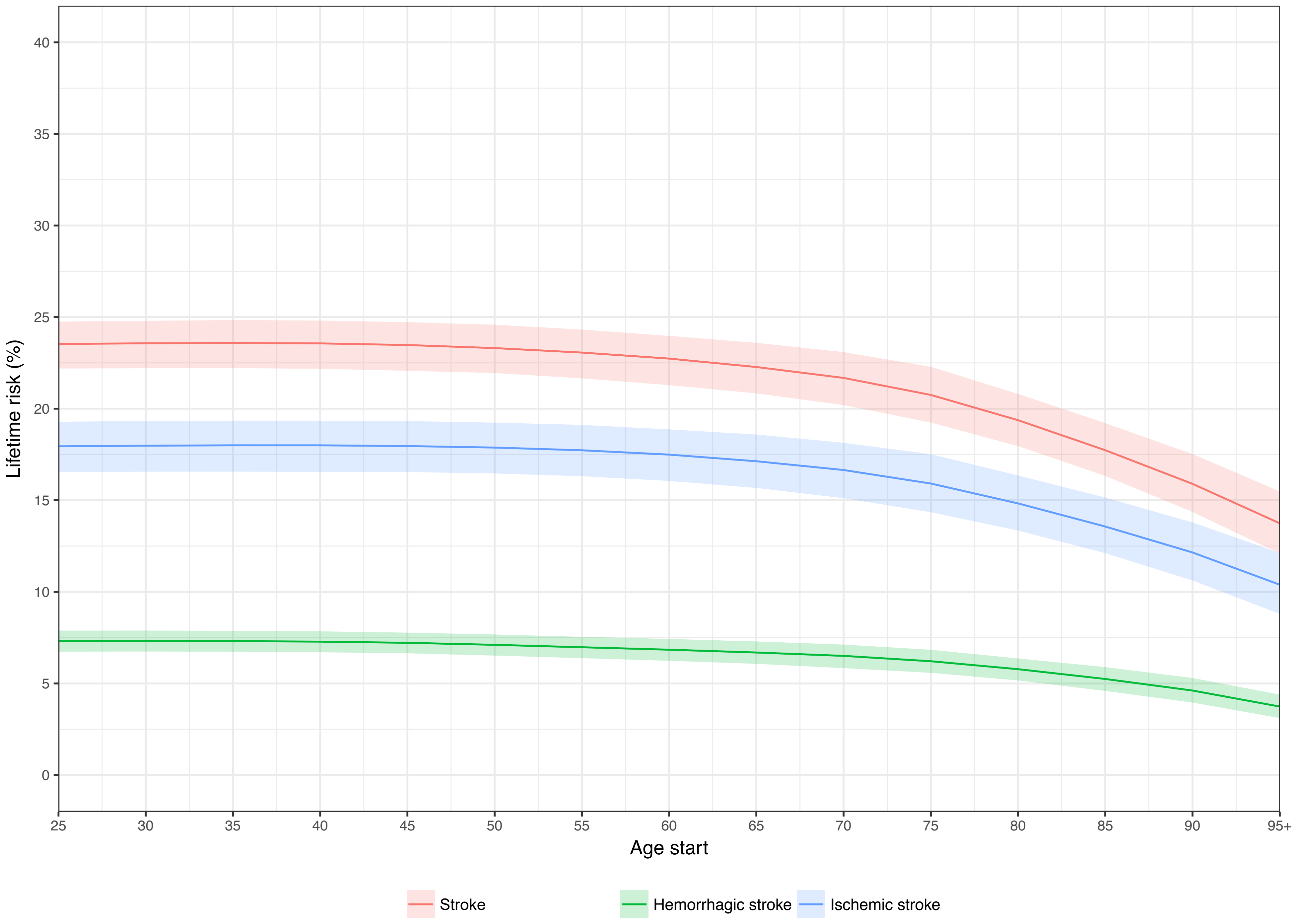

Figure 4B. High SDI lifetime risk of stroke occurrence by cause and age for males, 2016.

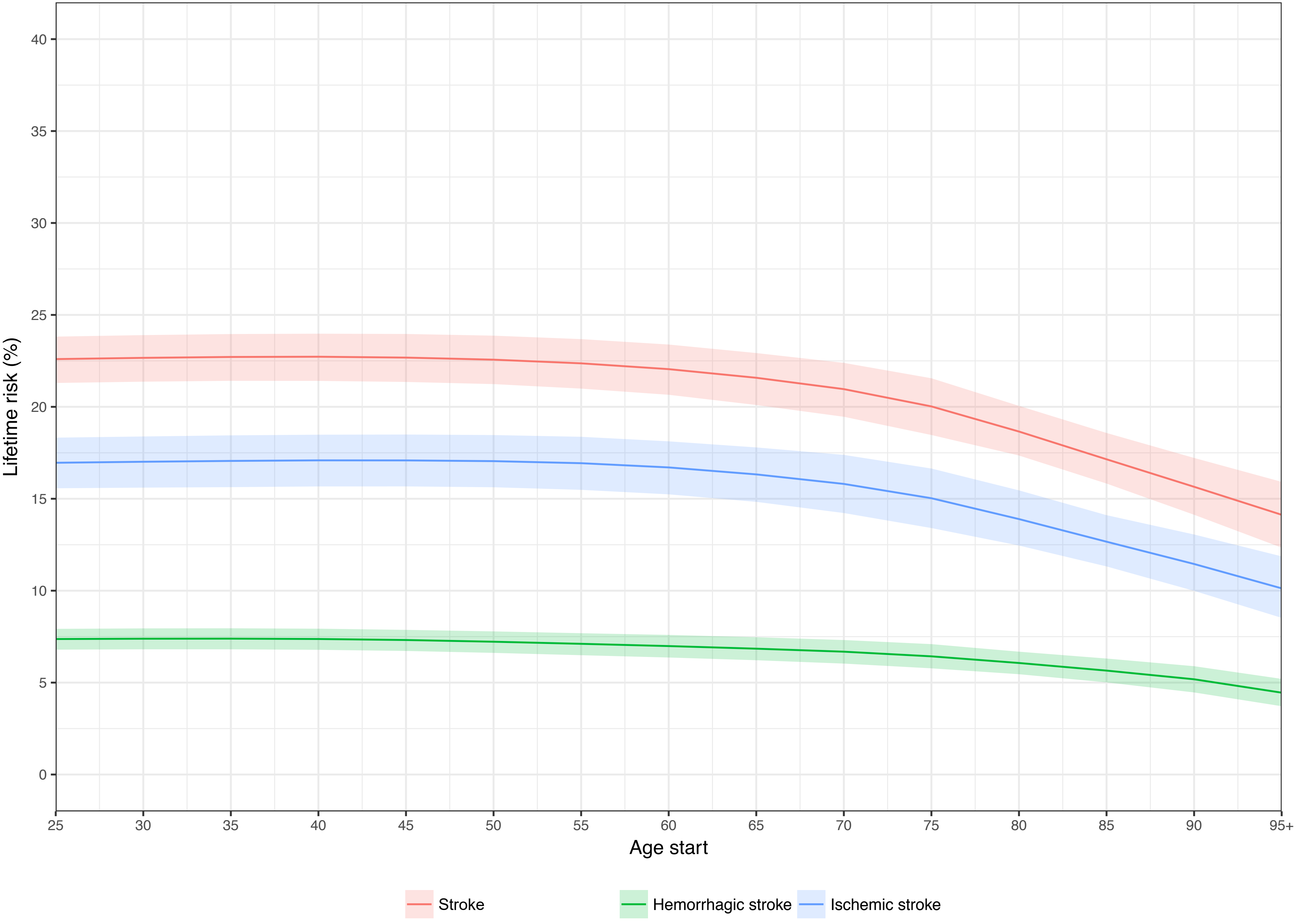

Figure 4C. High SDI lifetime risk of stroke occurrence by cause and age for females, 2016.

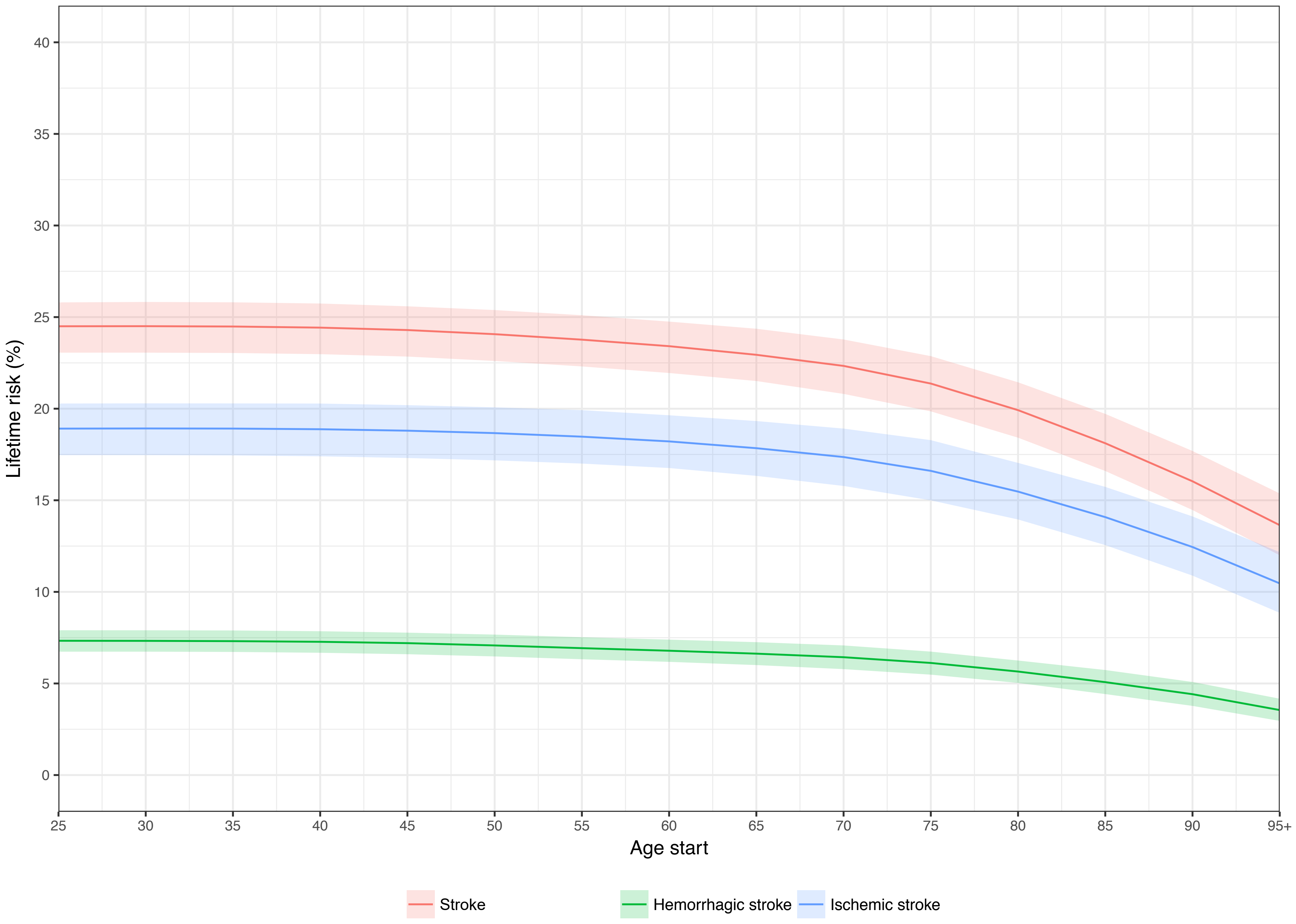

Figure 5A. High−middle SDI lifetime risk of stroke occurrence by cause and age for both sexes combined, 2016.

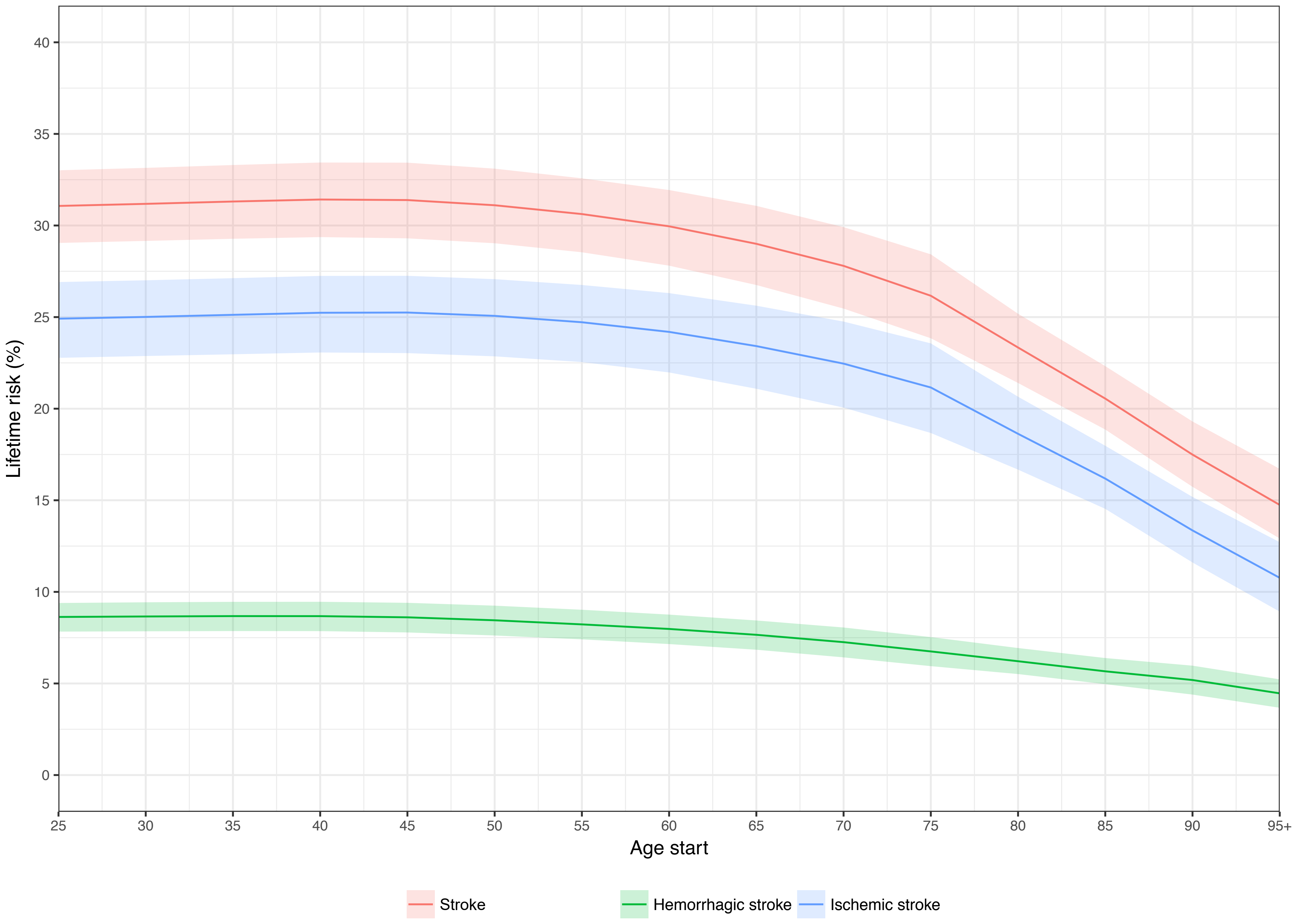

Figure 5B. High−middle SDI lifetime risk of stroke occurrence by cause and age for males, 2016.

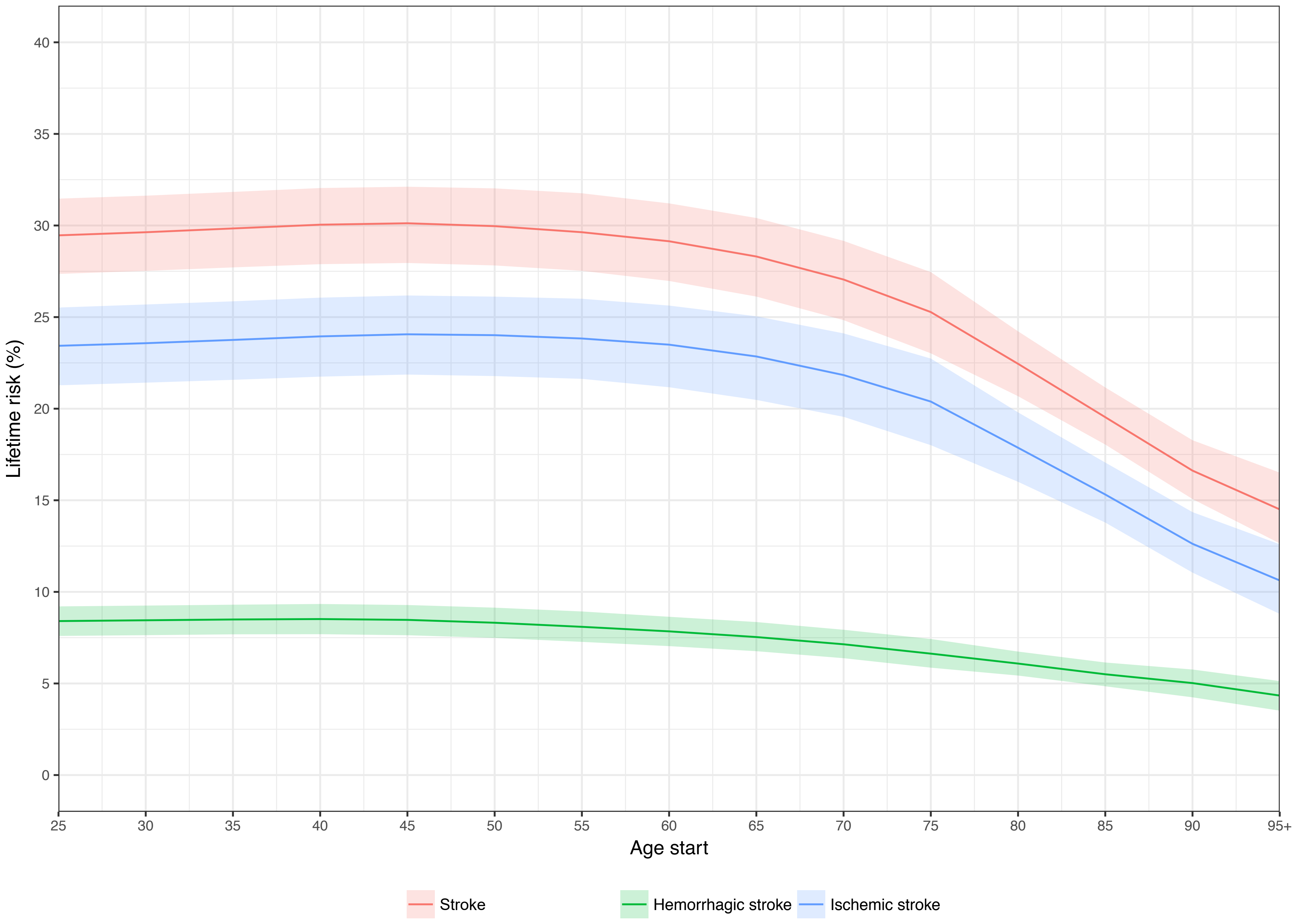

Figure 5C. High−middle SDI lifetime risk of stroke occurrence by cause and age for females, 2016.

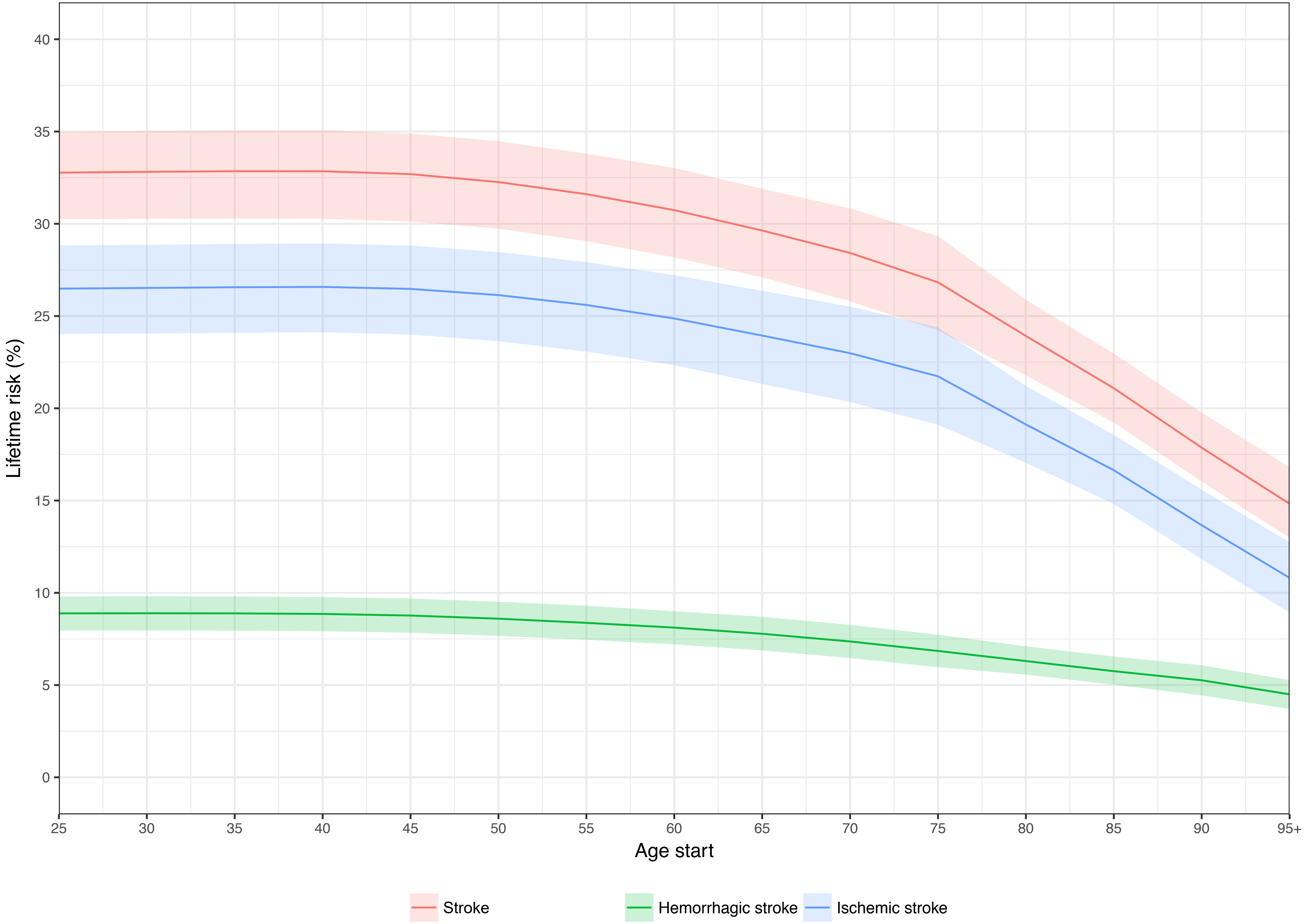

Figure 6A. Middle SDI lifetime risk of stroke occurrence by cause and age for both sexes combined, 2016.

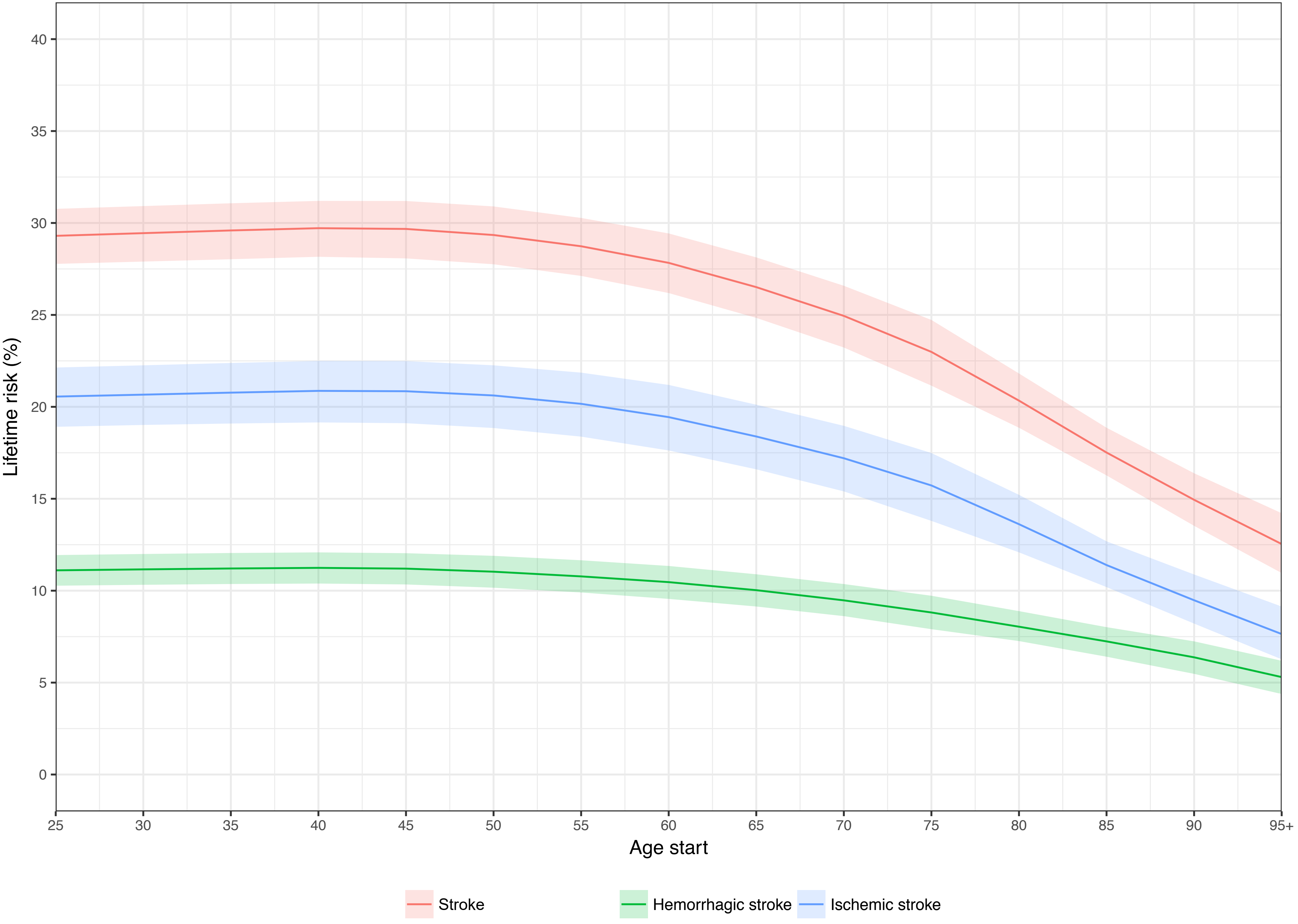

Figure 6B. Middle SDI lifetime risk of stroke occurrence by cause and age for males, 2016.

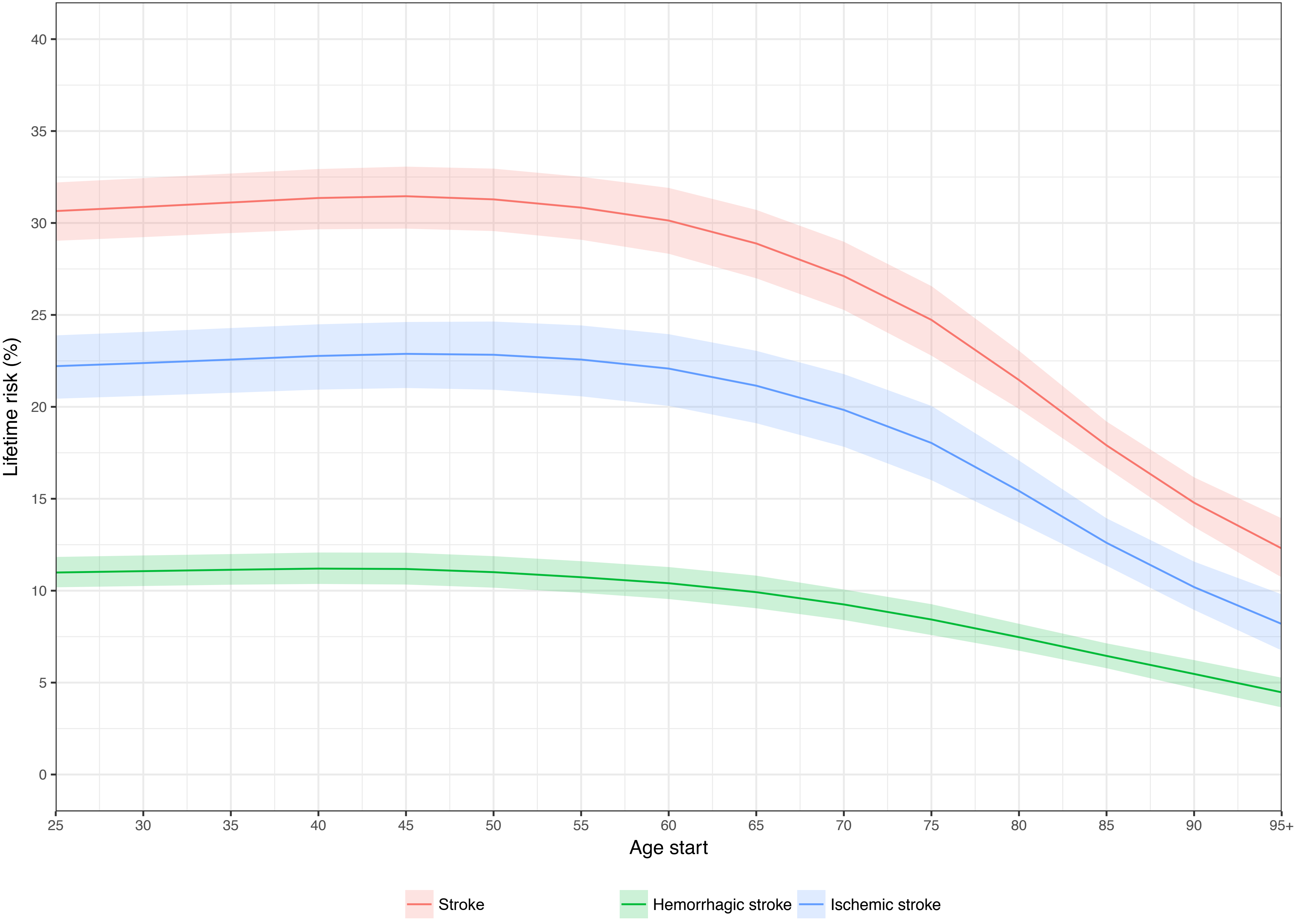

Figure 6C. Middle SDI lifetime risk of stroke occurrence by cause and age for females, 2016.

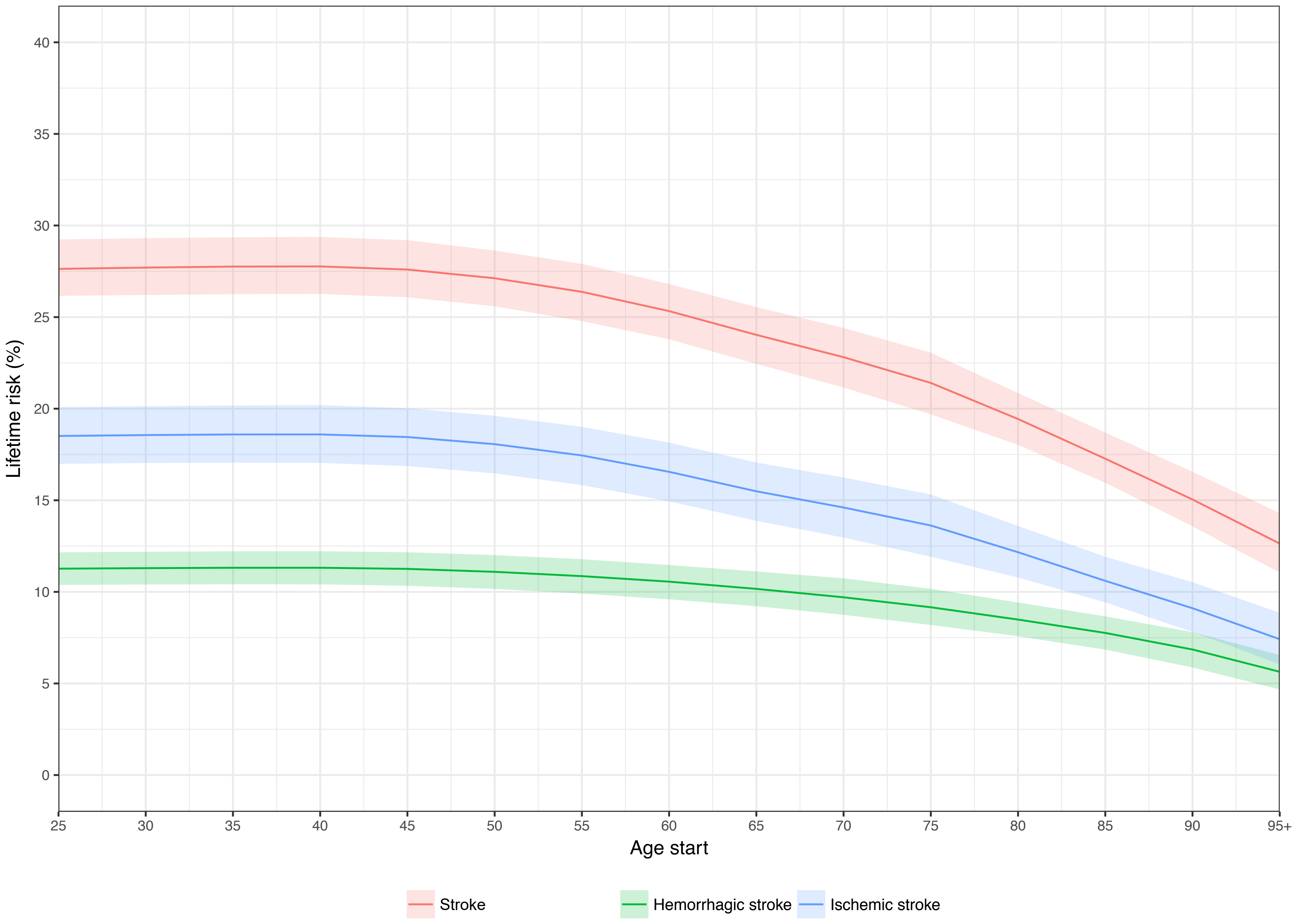

Figure 7A. Low–middle SDI lifetime risk of stroke occurrence by cause and age for both sexes combined, 2016.

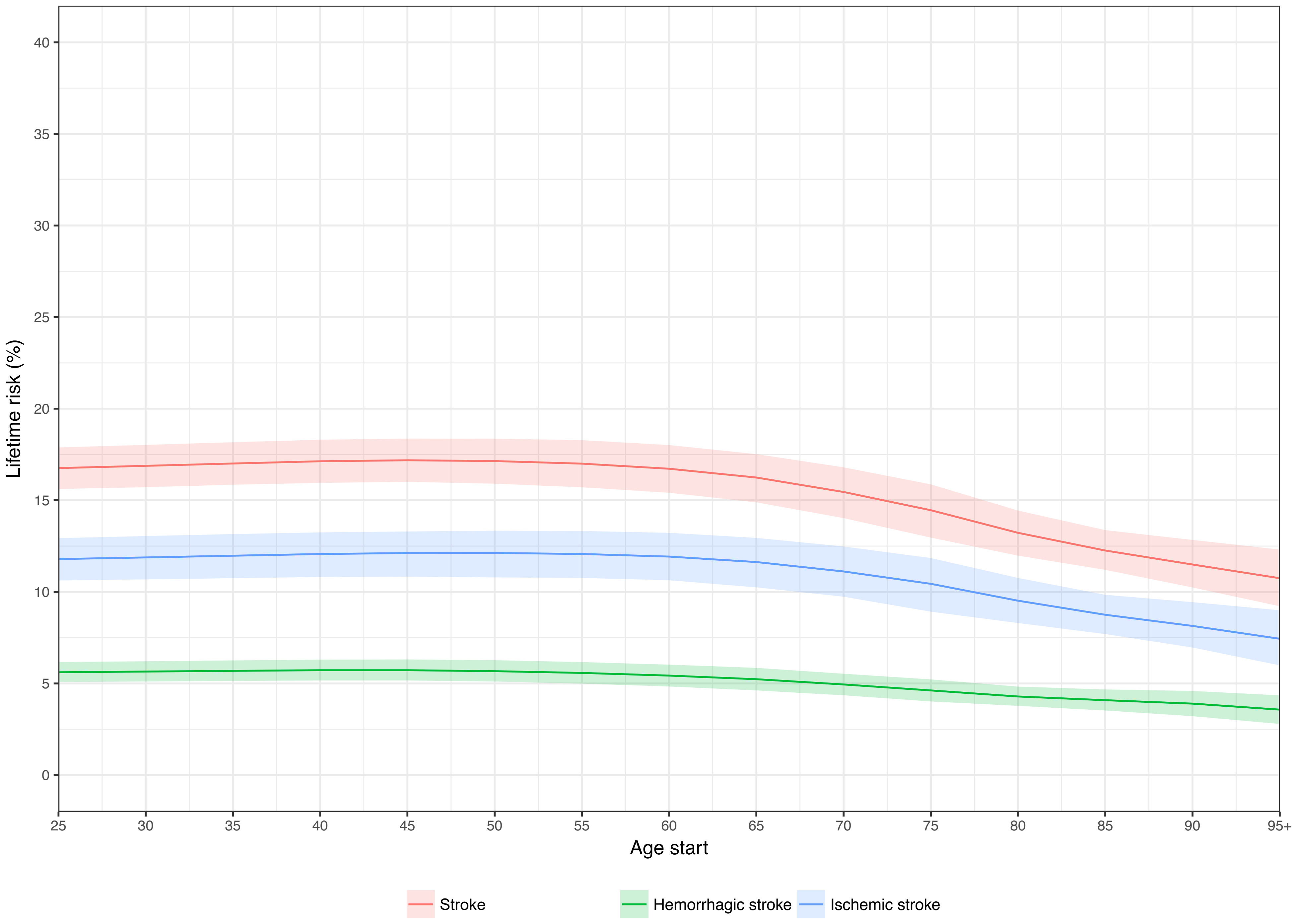

Figure 7B. Low–middle SDI lifetime risk of stroke occurrence by cause and age for males, 2016.

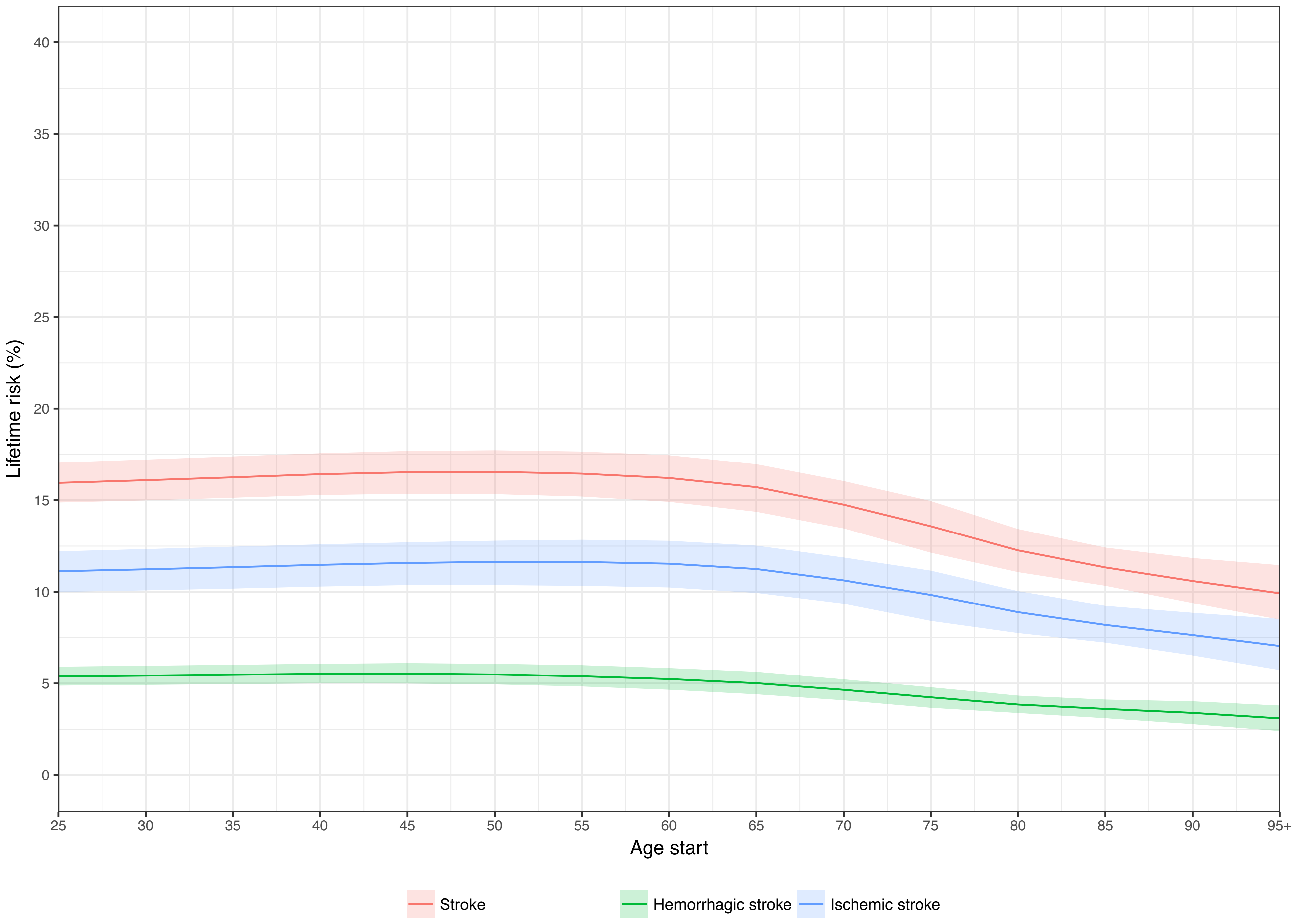

Figure 7C. Low–middle SDI lifetime risk of stroke occurrence by cause and age for females, 2016.

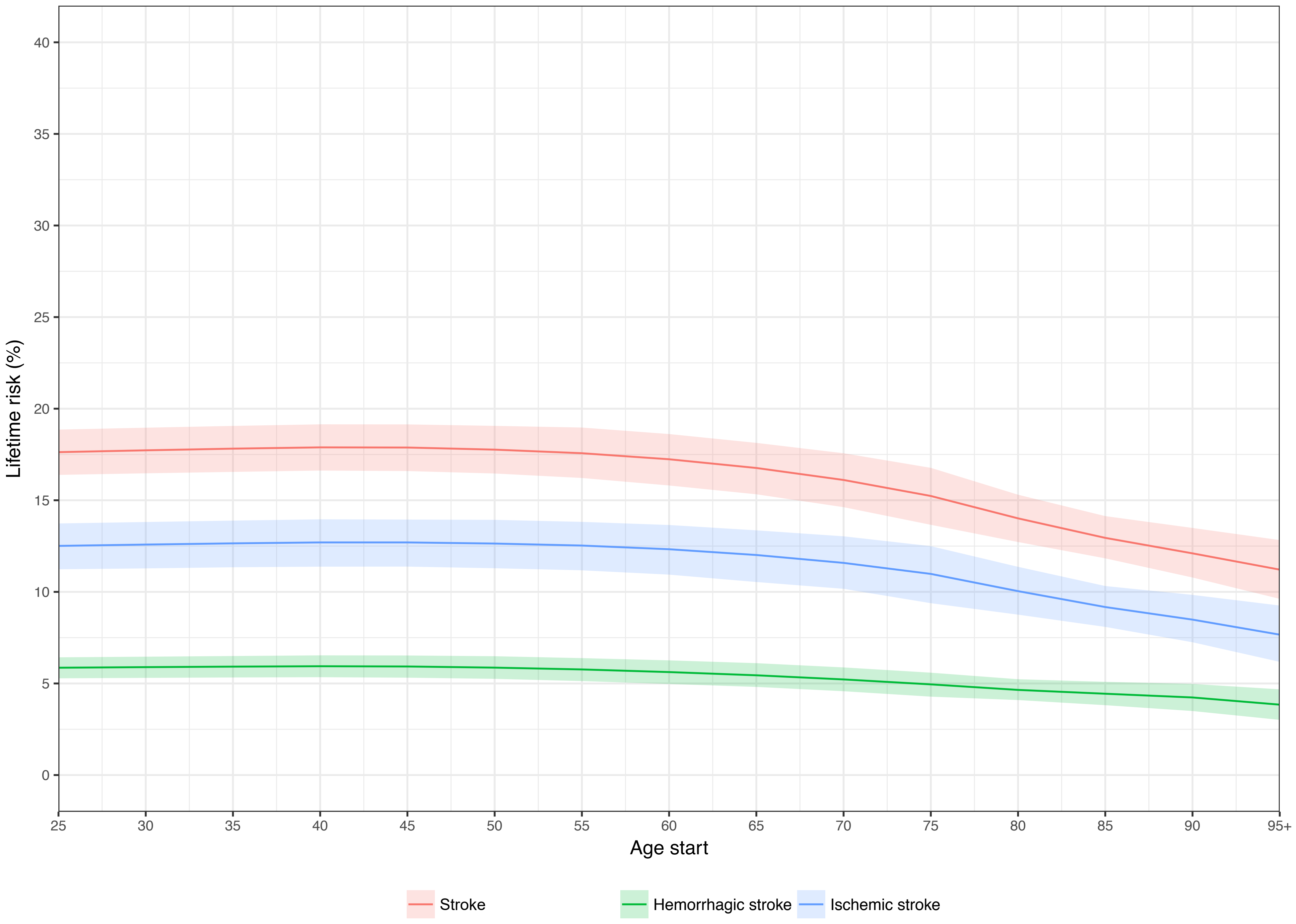

Figure 8A. Low SDI lifetime risk of stroke occurrence by cause and age for both sexes combined, 2016.

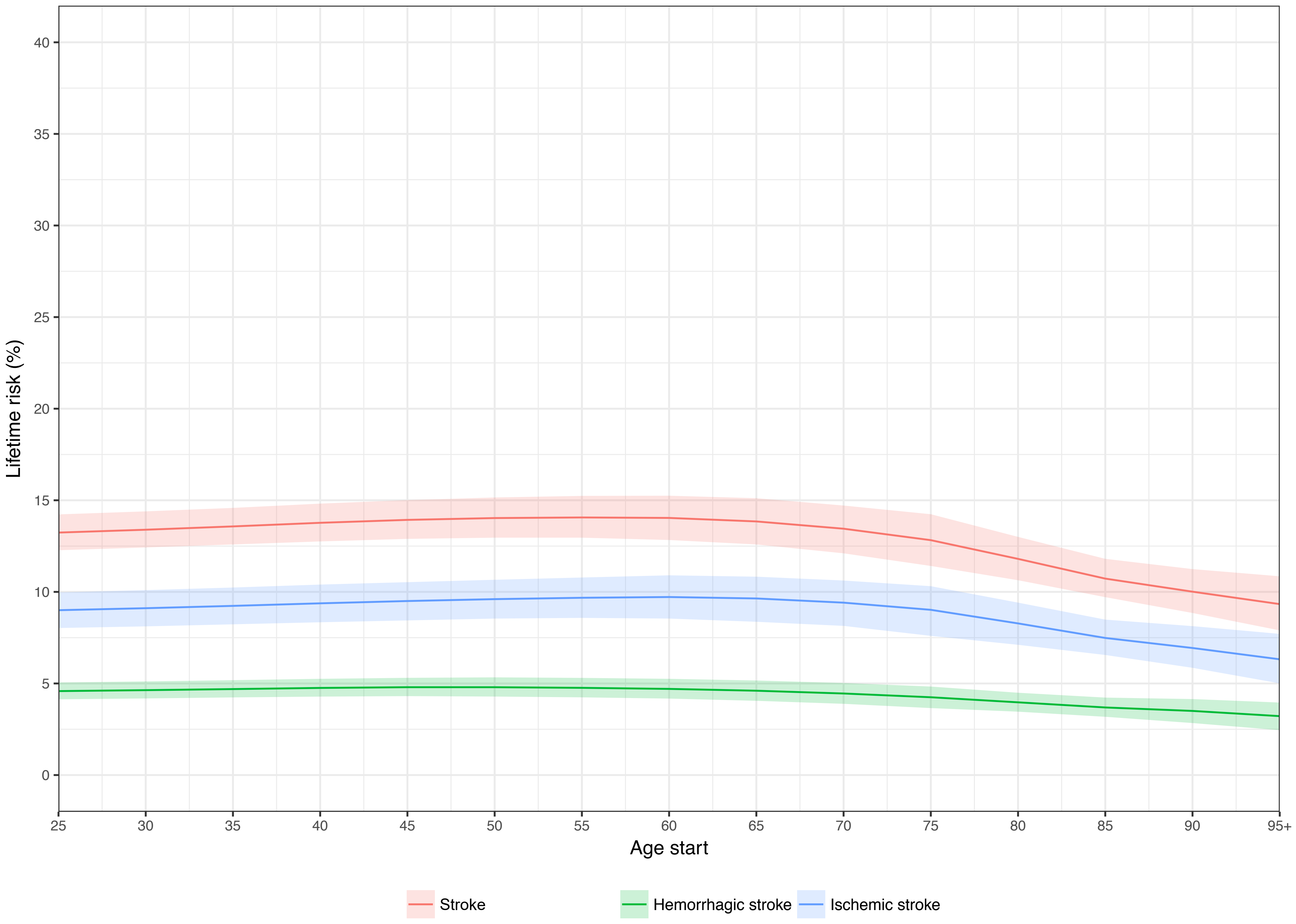

Figure 8B. Low SDI lifetime risk of stroke occurrence by cause and age for males, 2016.

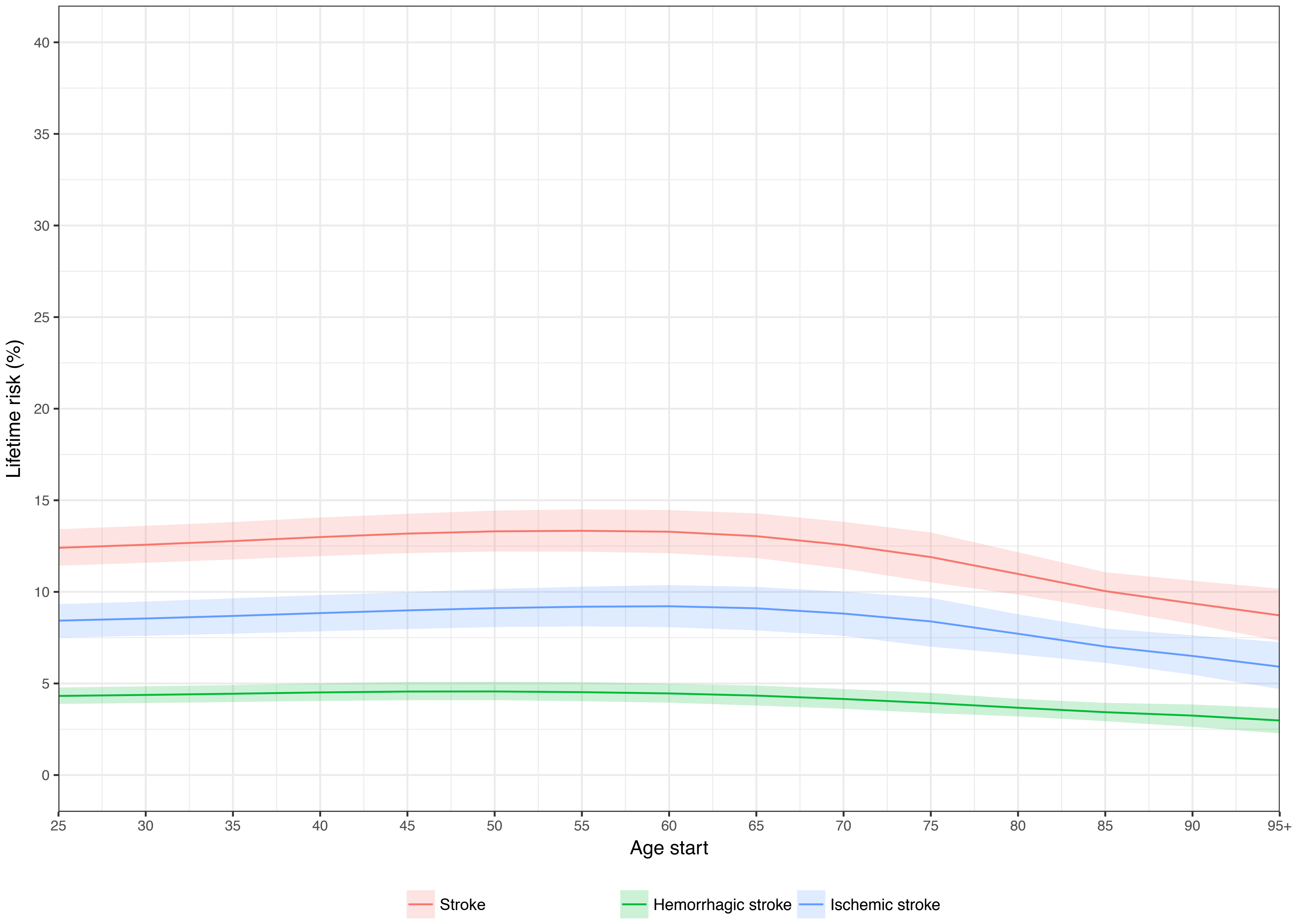

Figure 8C. Low SDI lifetime risk of stroke occurrence by cause and age for females, 2016.

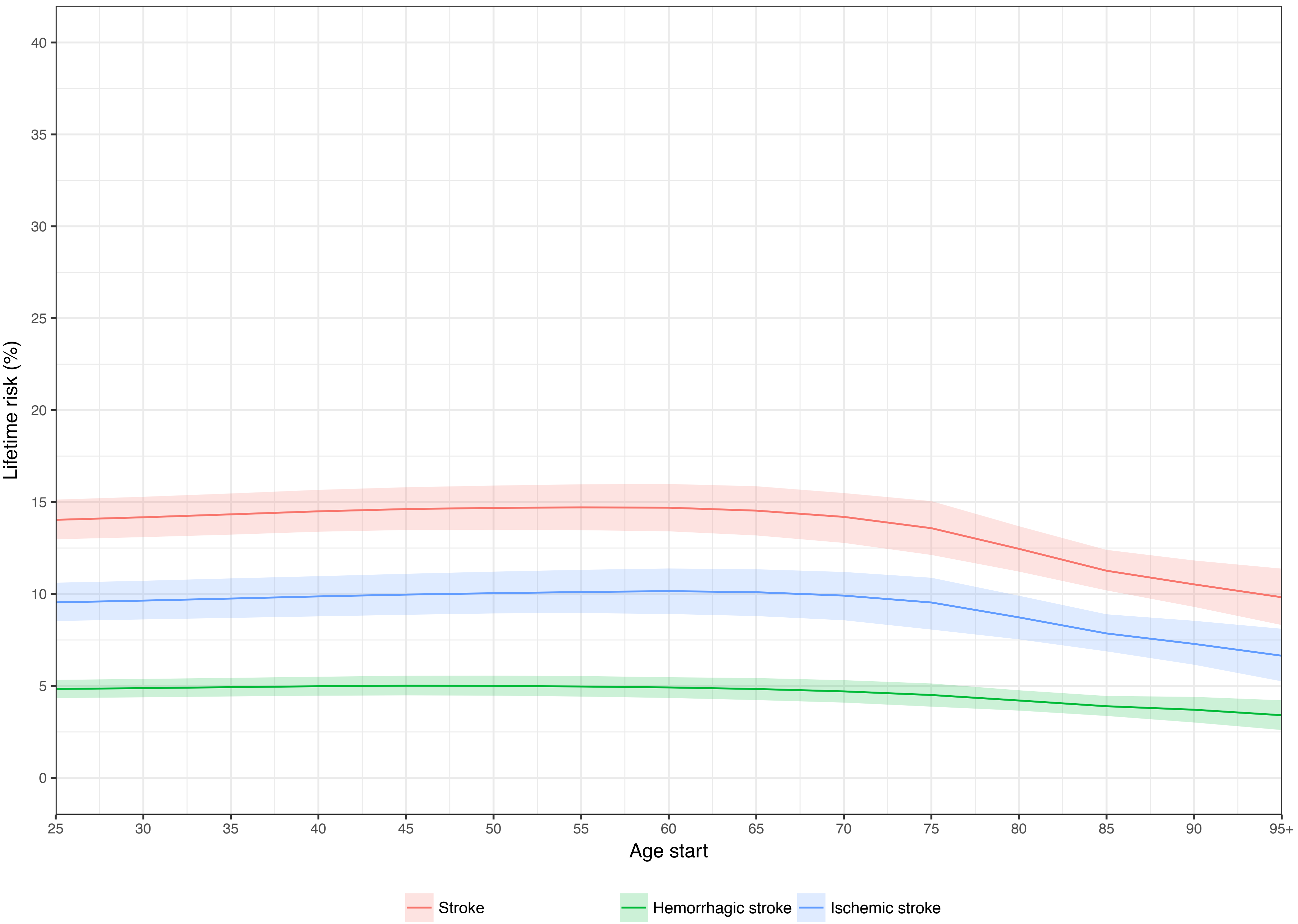

Figure S9A. Incident stroke vs non-stroke mortality by age, global, both sexes, 2016

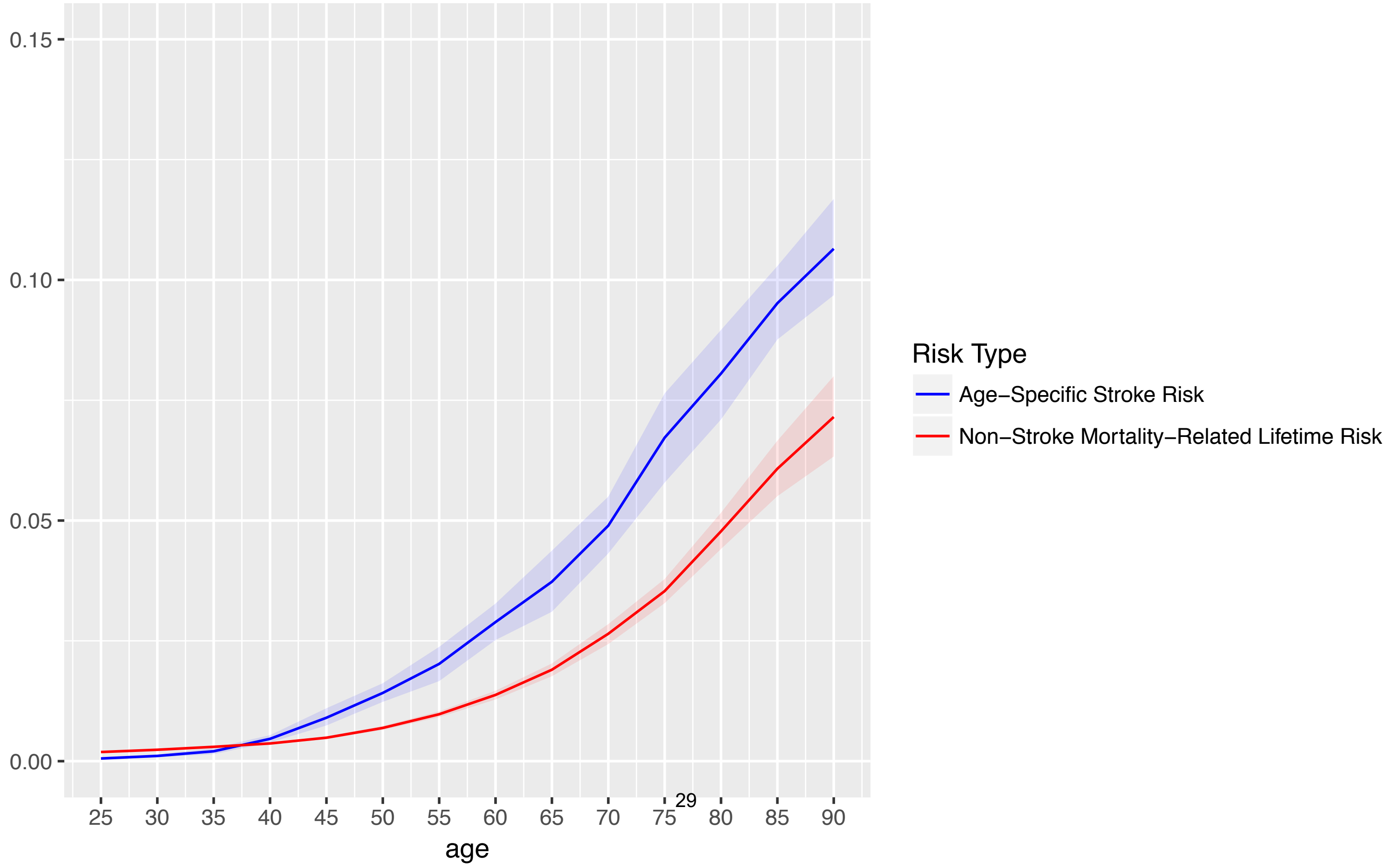

Figure S9B. Incident stroke vs non-stroke mortality by age, global, male, 2016

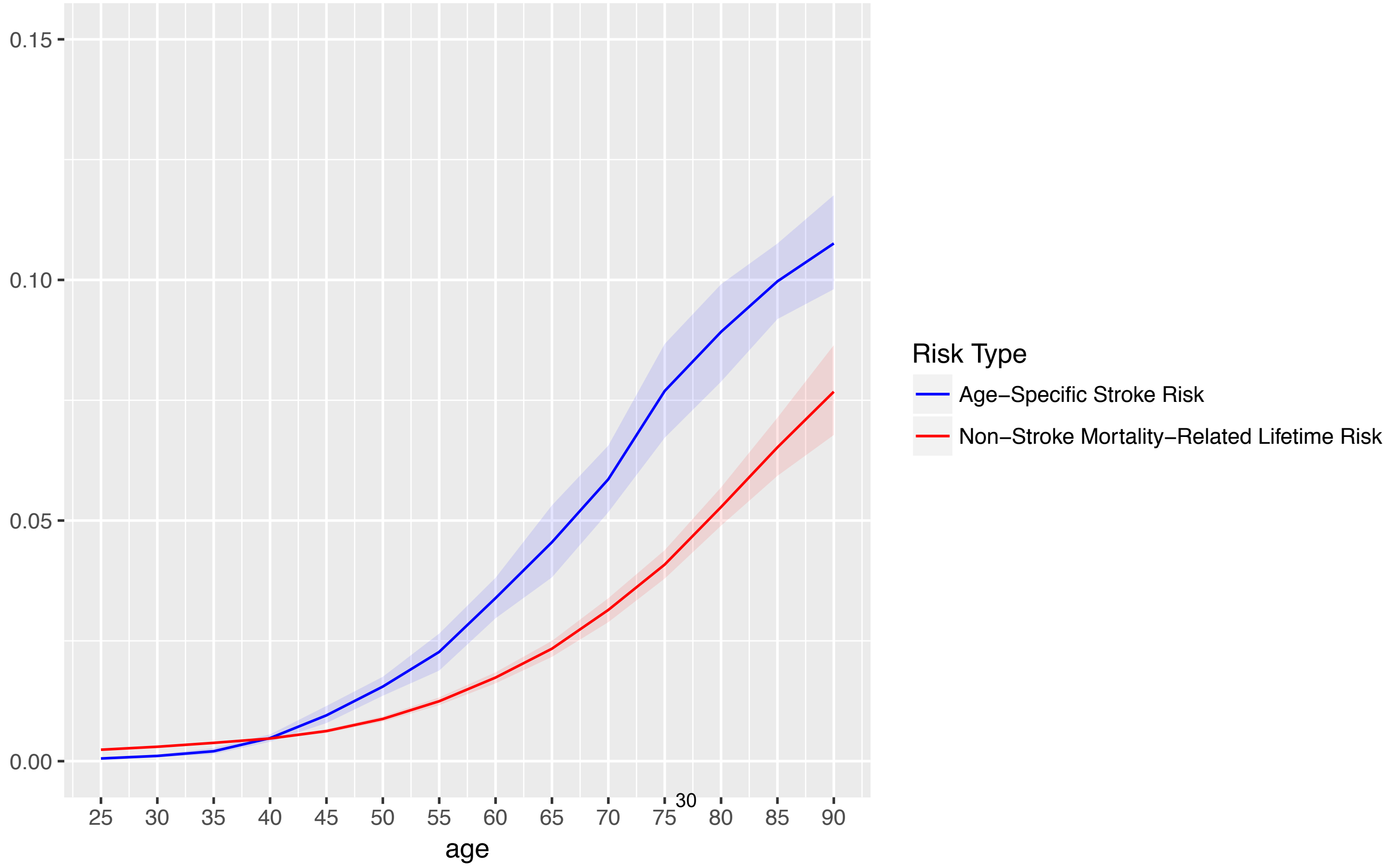

Figure S9C. Incident stroke vs non-stroke mortality by age, global, female, 2016

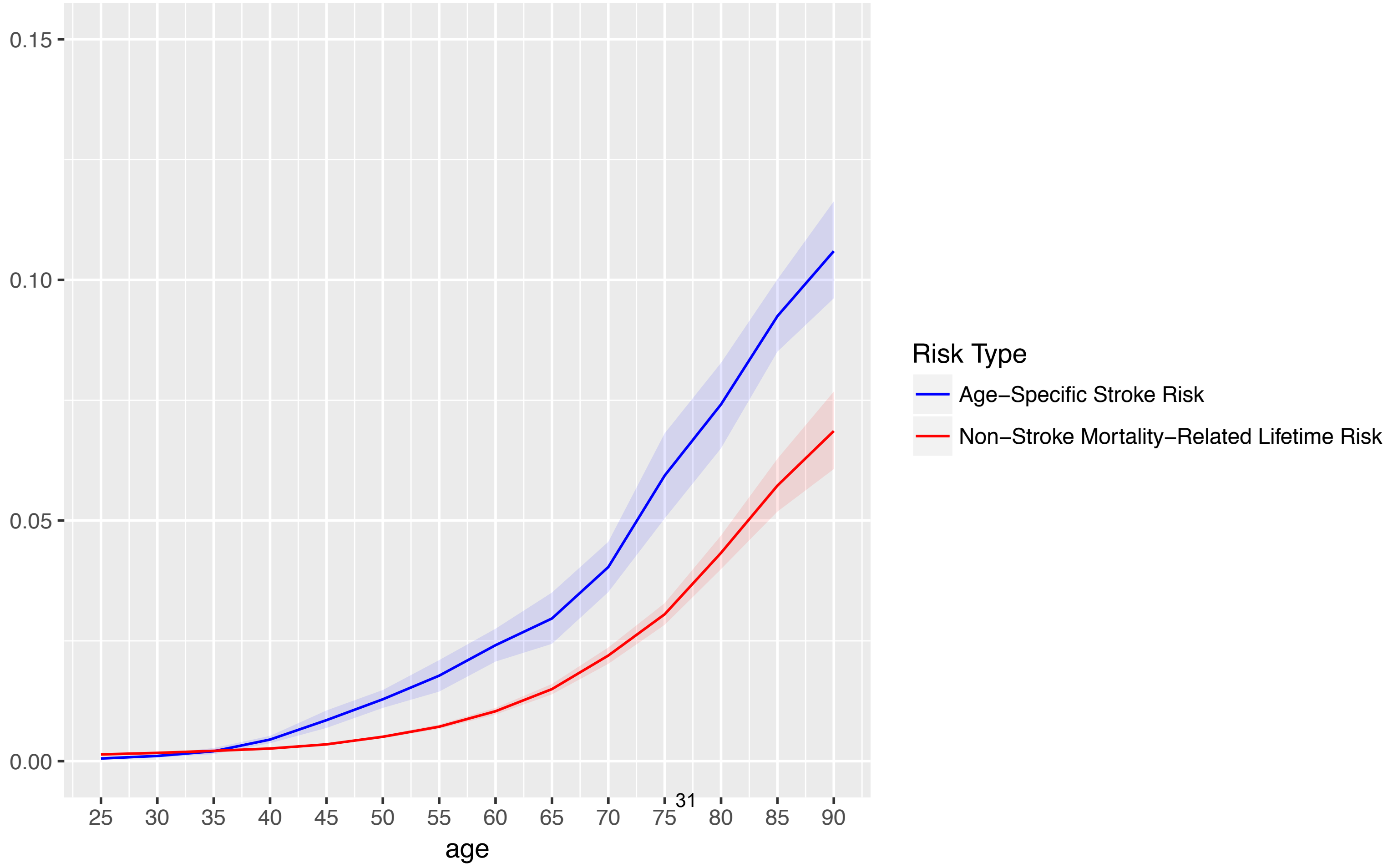

Figure S9D. Incident stroke vs non-stroke mortality by age, high SDI, both sexes, 2016

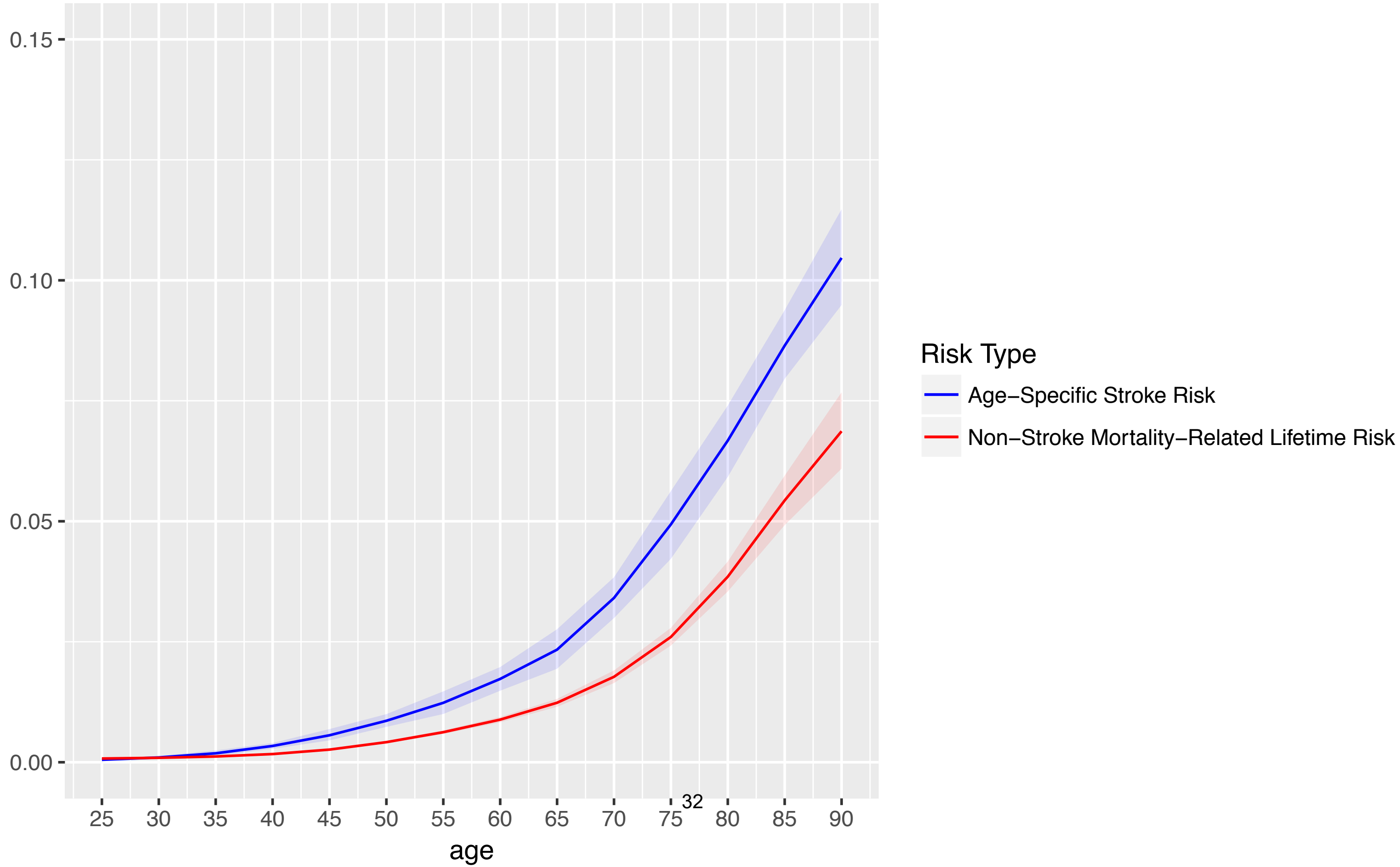

Figure S9E. Incident stroke vs non-stroke mortality by age, high SDI, males, 2016

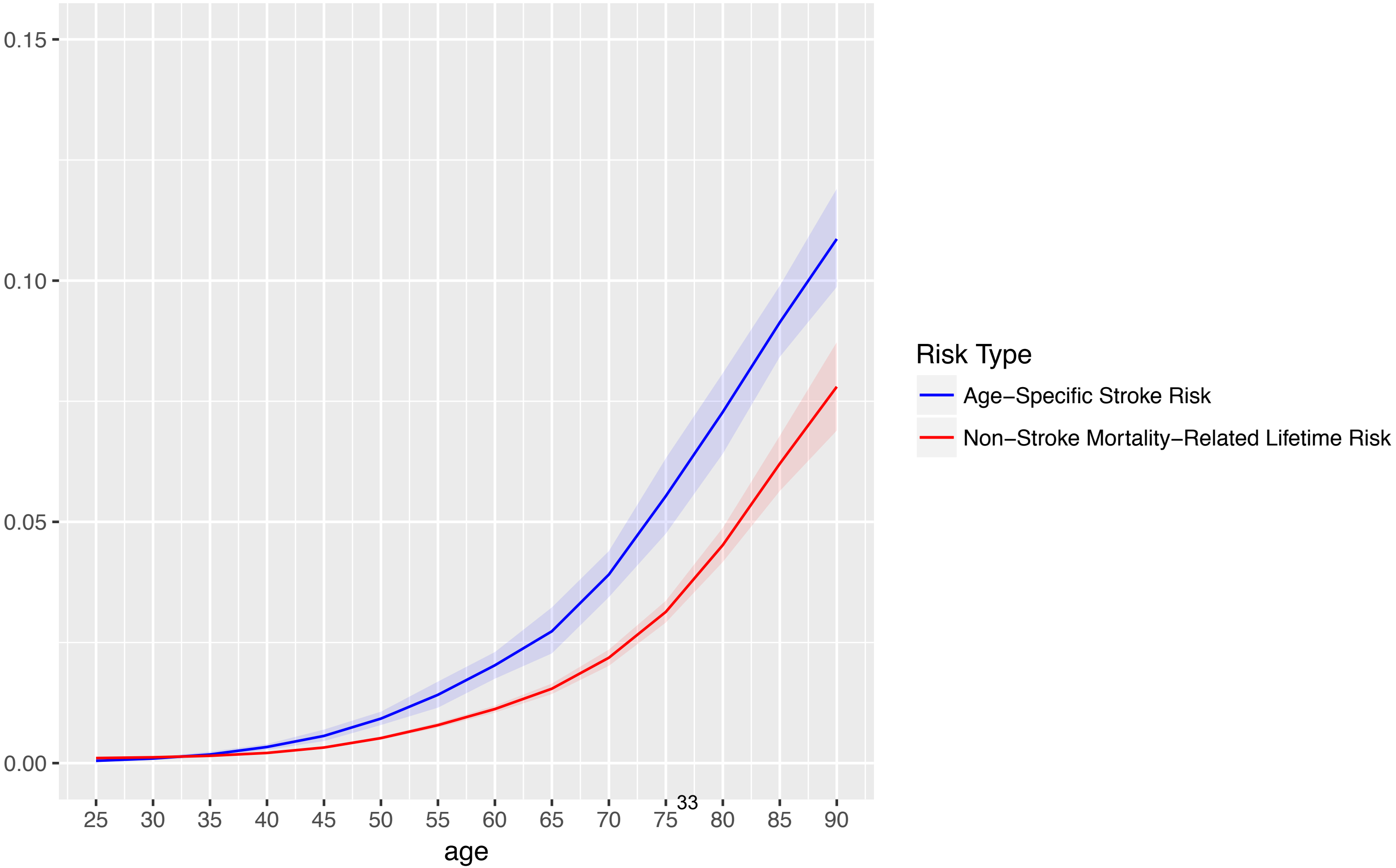

Figure S9F. Incident stroke vs non-stroke mortality by age, high SDI, females, 2016

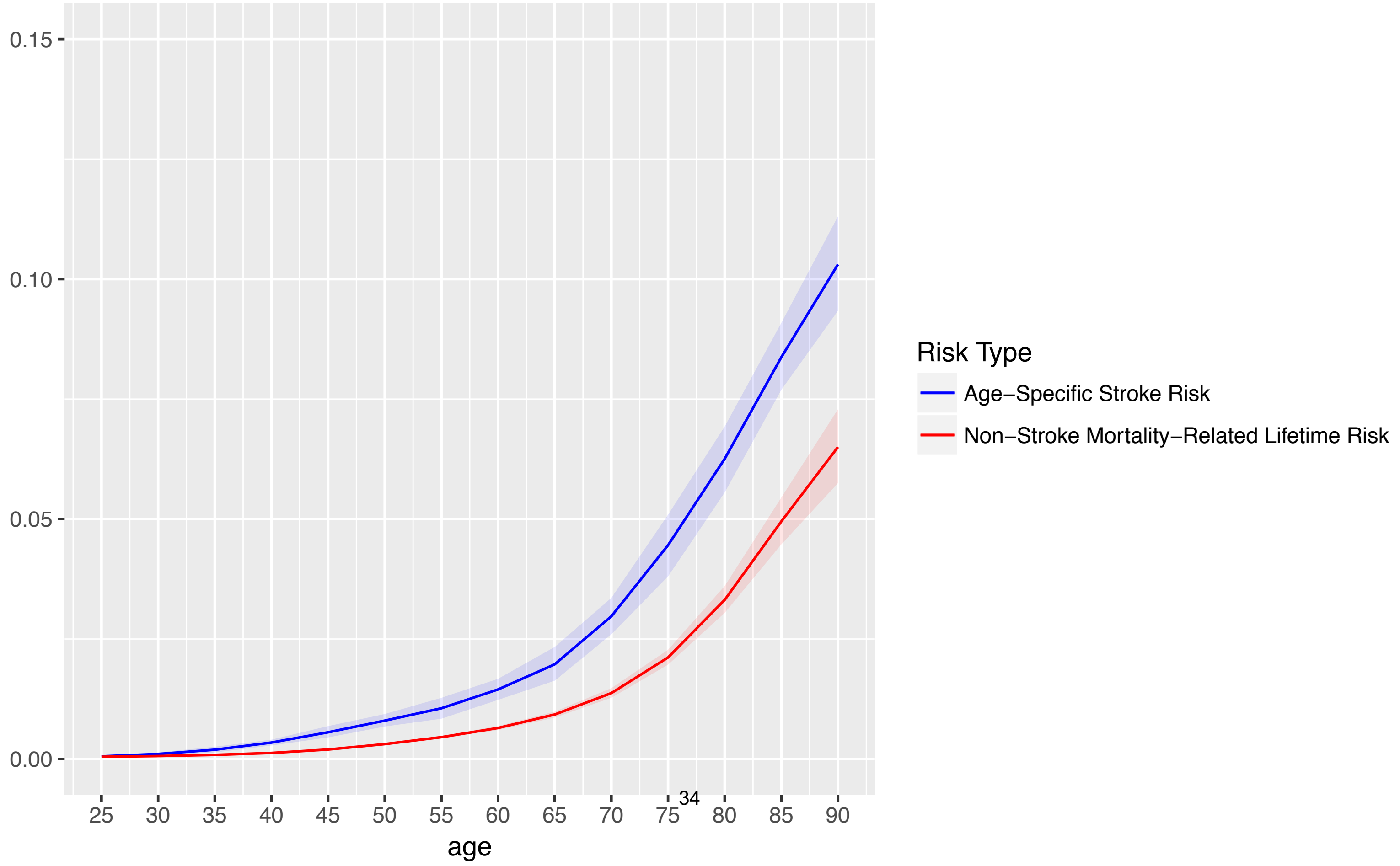

Figure S9G. Incident stroke vs non-stroke mortality by age, high-middle SDI, both sexes, 2016

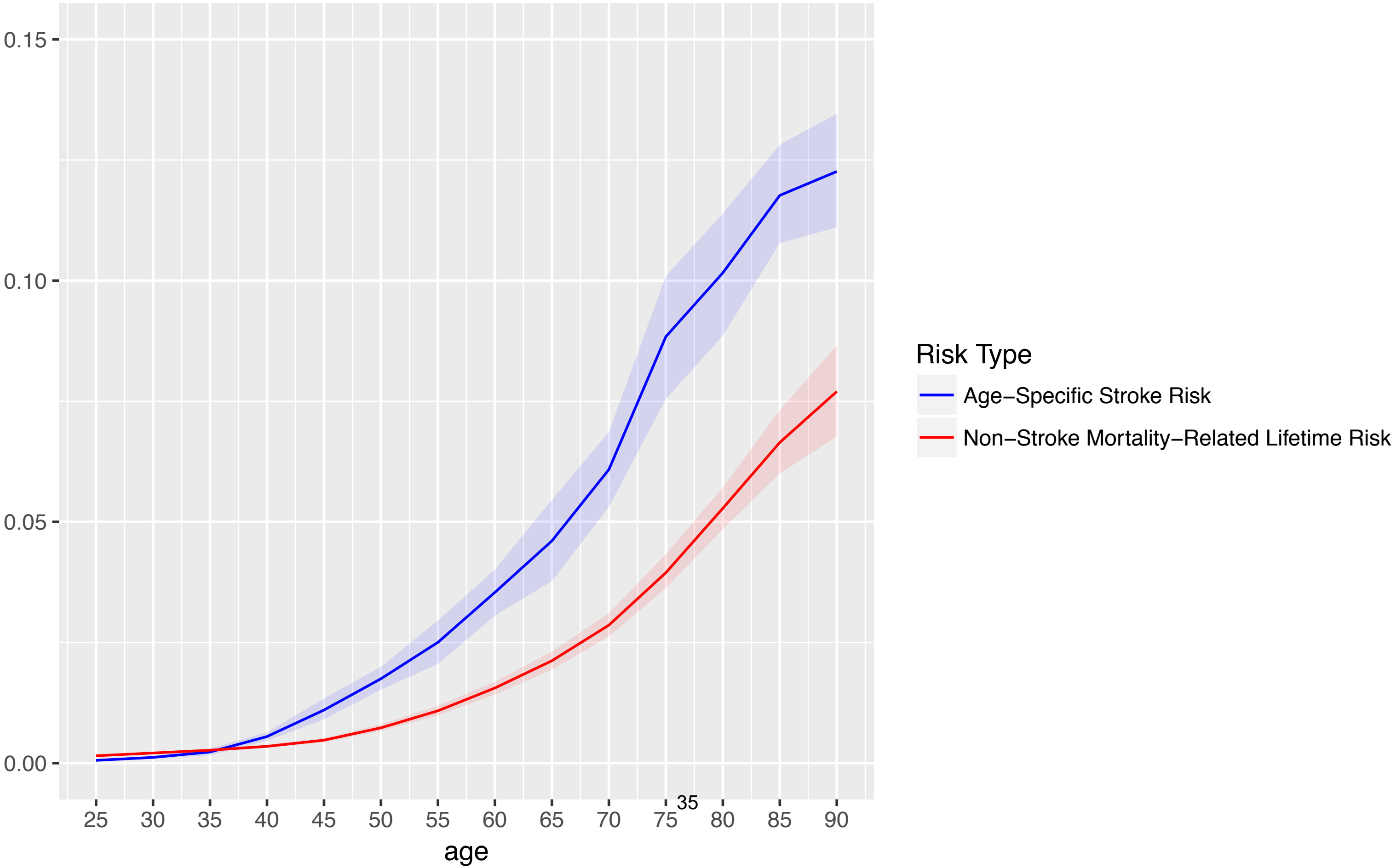

Figure S9H. Incident stroke vs non-stroke mortality by age, high-middle SDI, males, 2016

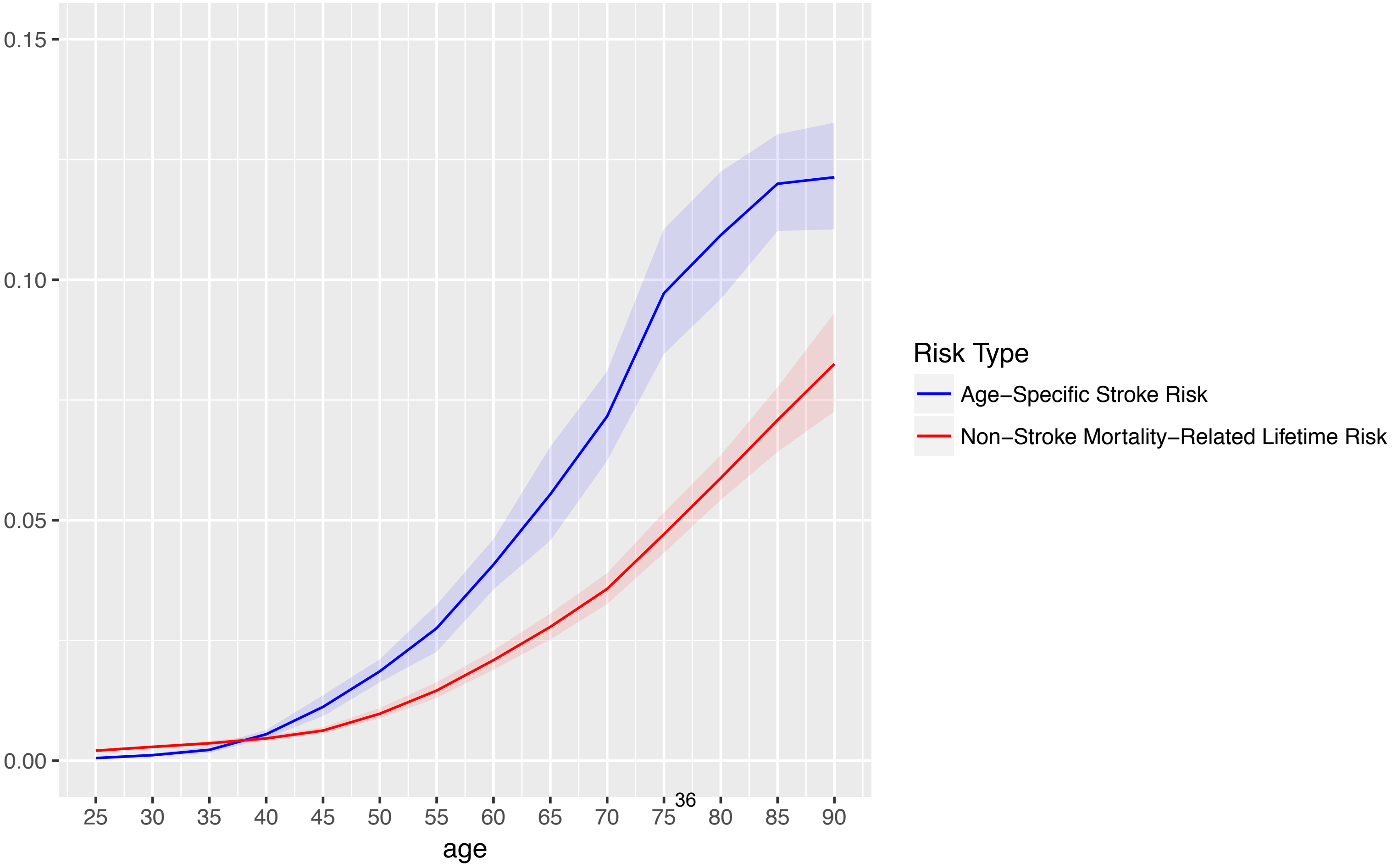

Figure S9I. Incident stroke vs non-stroke mortality by age, high-middle SDI, females, 2016

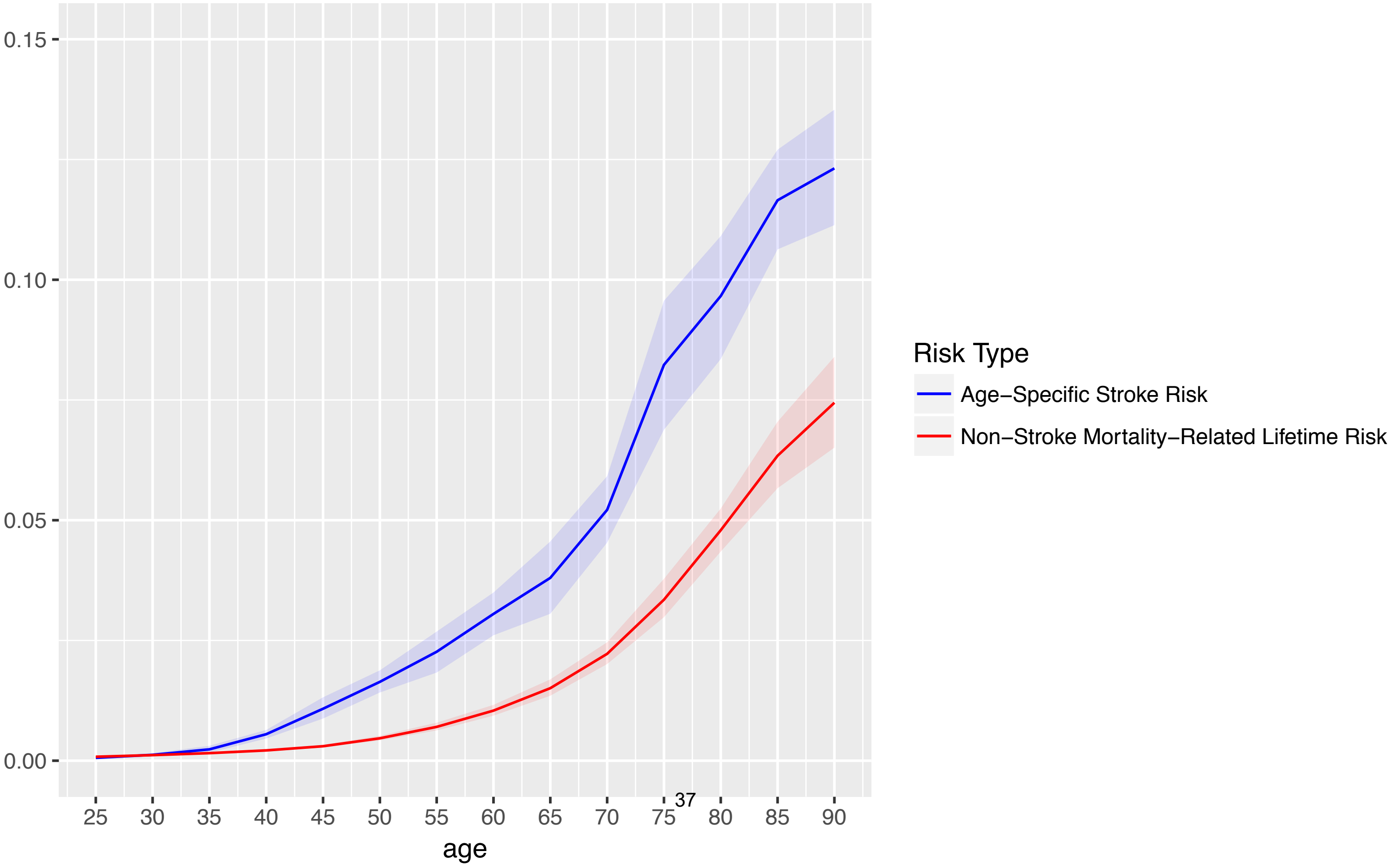

Figure S9J. Incident stroke vs non-stroke mortality by age, middle SDI, both sexes, 2016

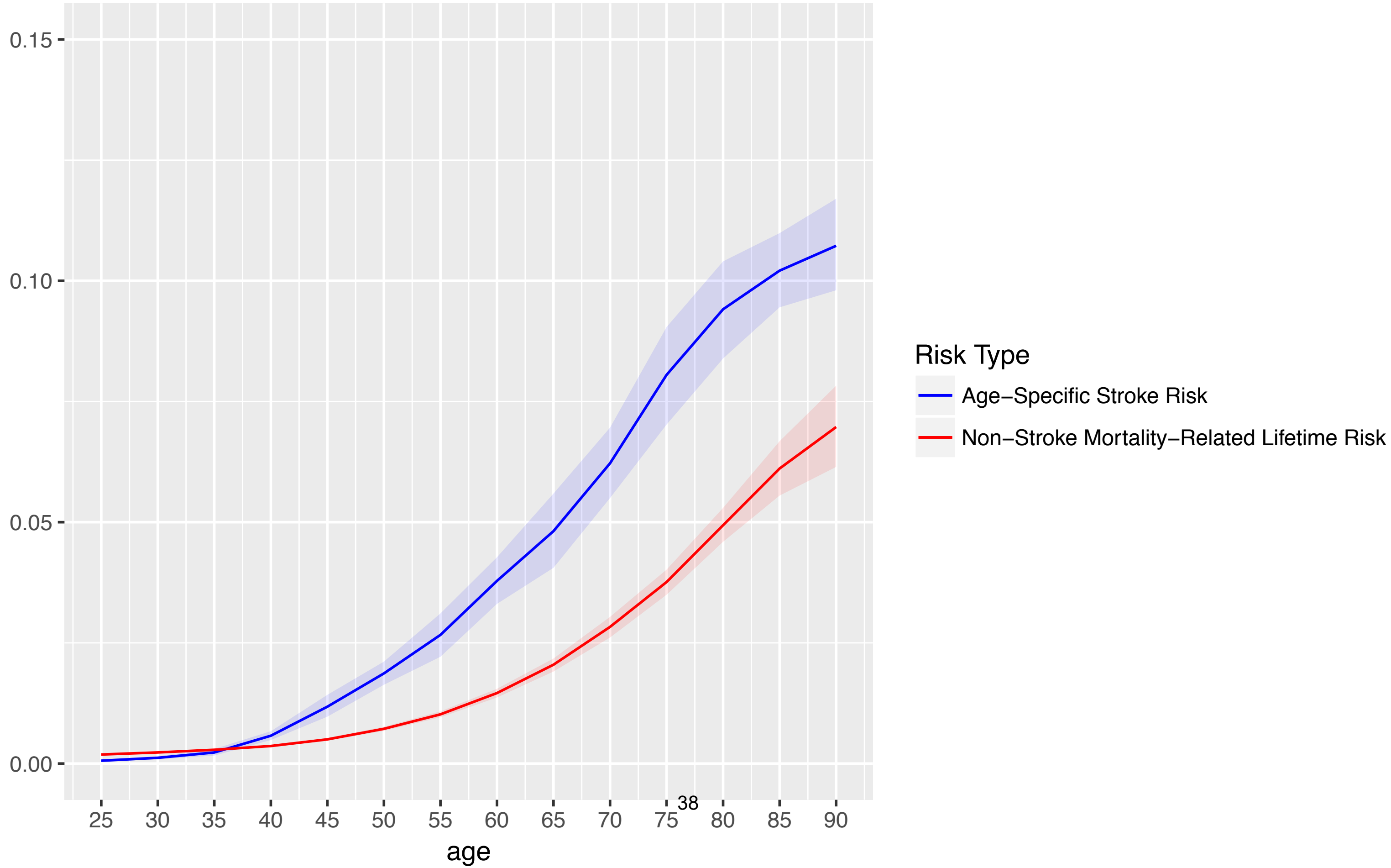

Figure S9K. Incident stroke vs non-stroke mortality by age, middle SDI, males, 2016

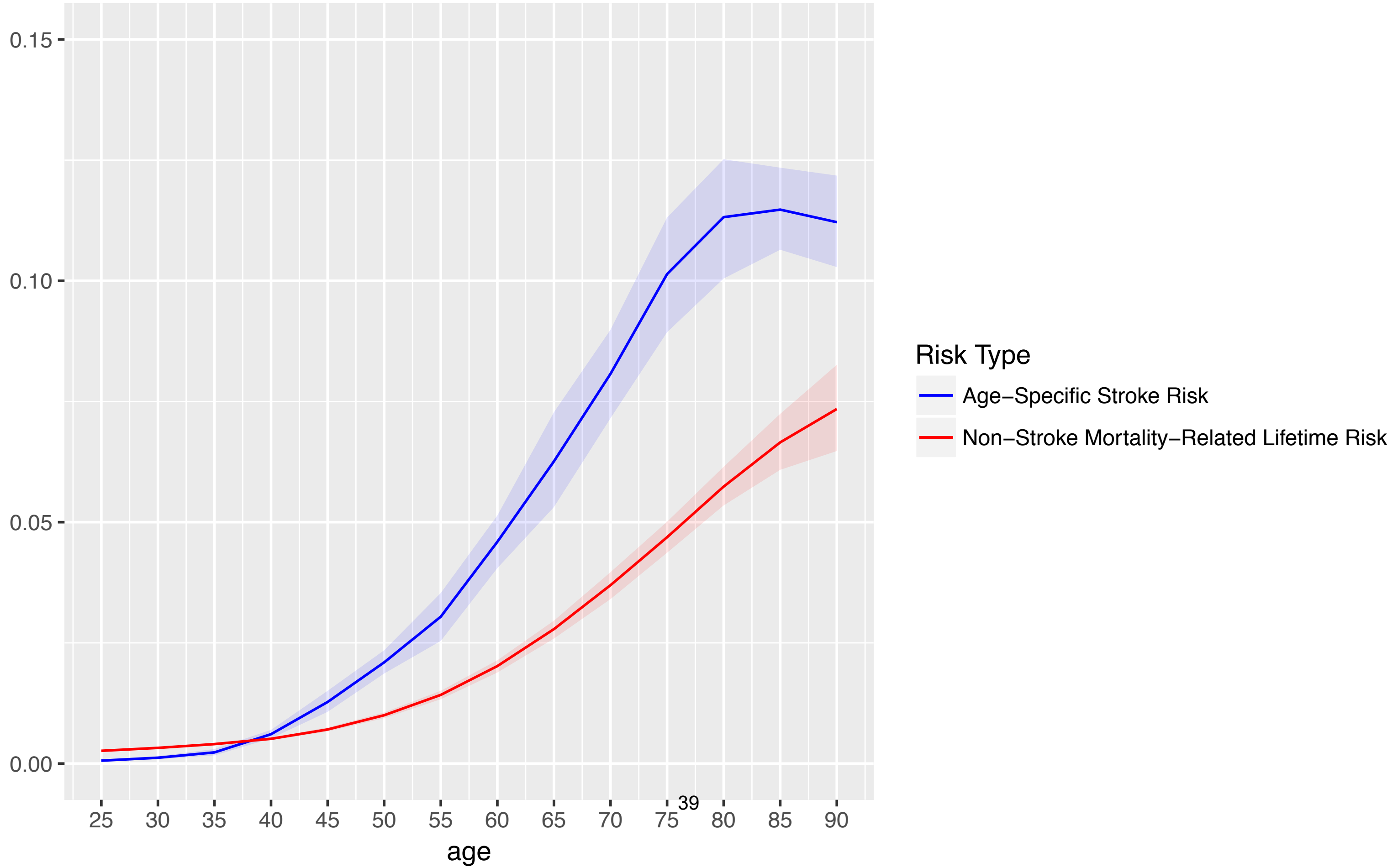

Figure S9L. Incident stroke vs non-stroke mortality by age, middle SDI, females, 2016

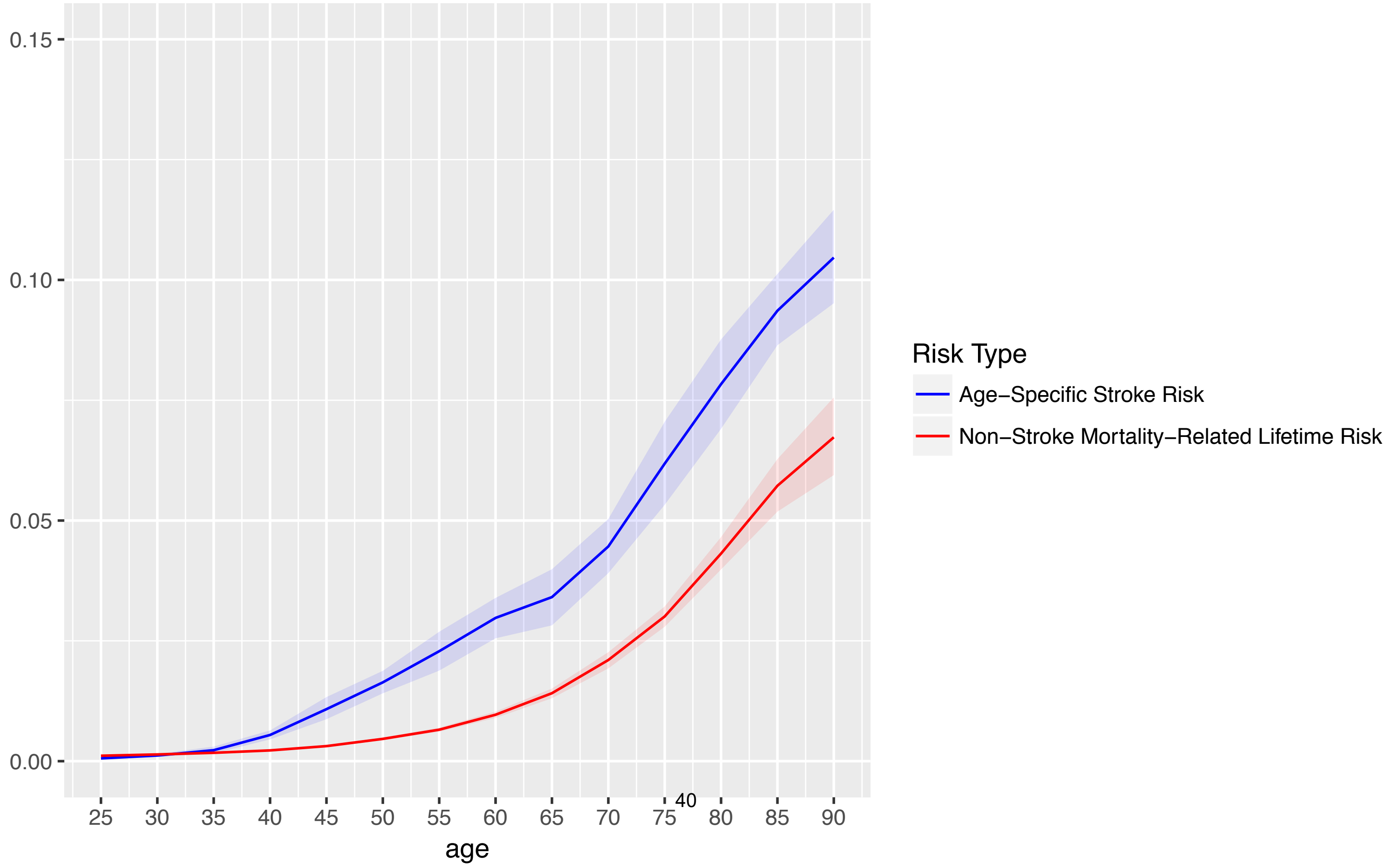

Figure S9M. Incident stroke vs non-stroke mortality by age, low-middle SDI, both sexes, 2016

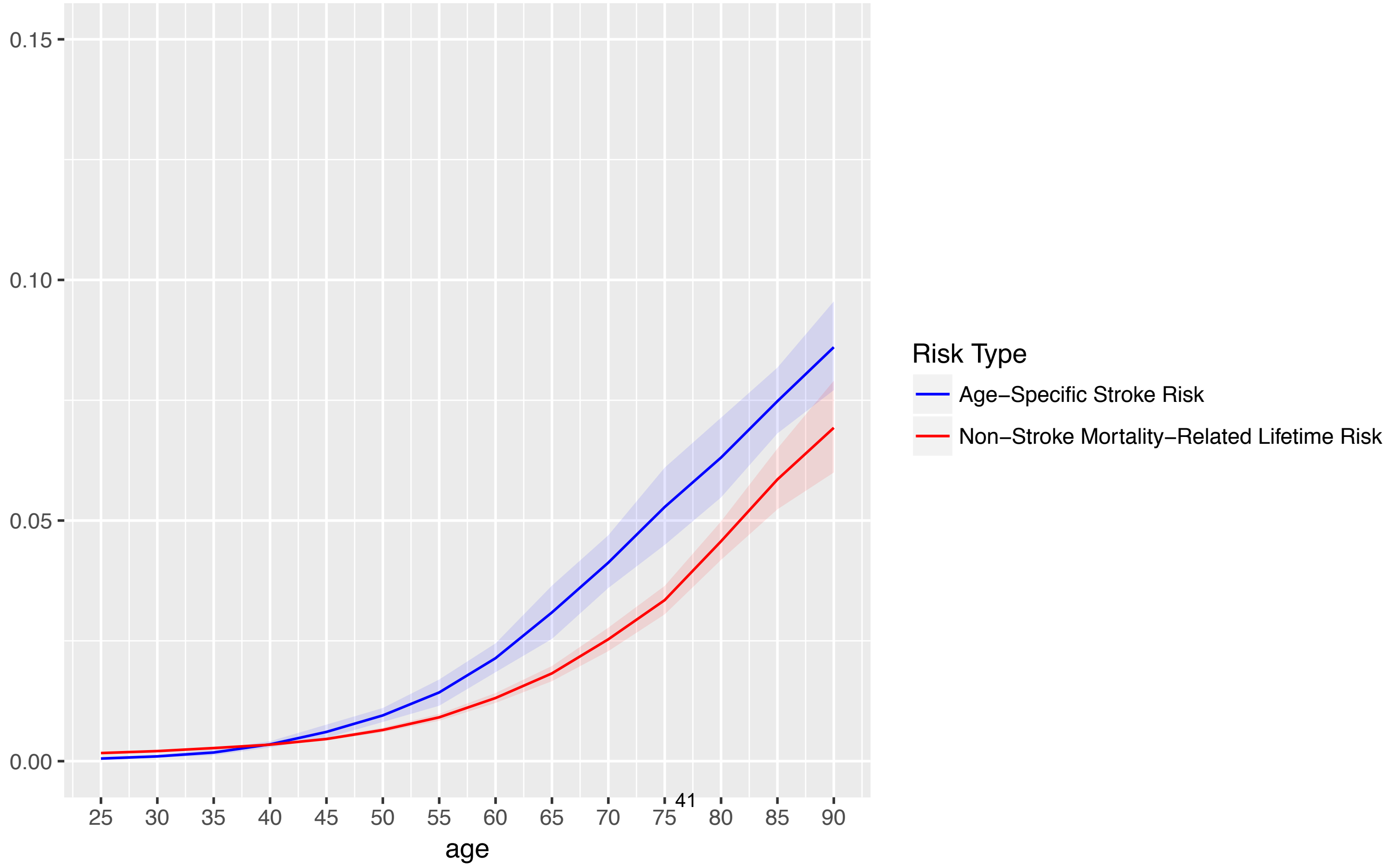

Figure S9N. Incident stroke vs non-stroke mortality by age, low-middle SDI, males, 2016

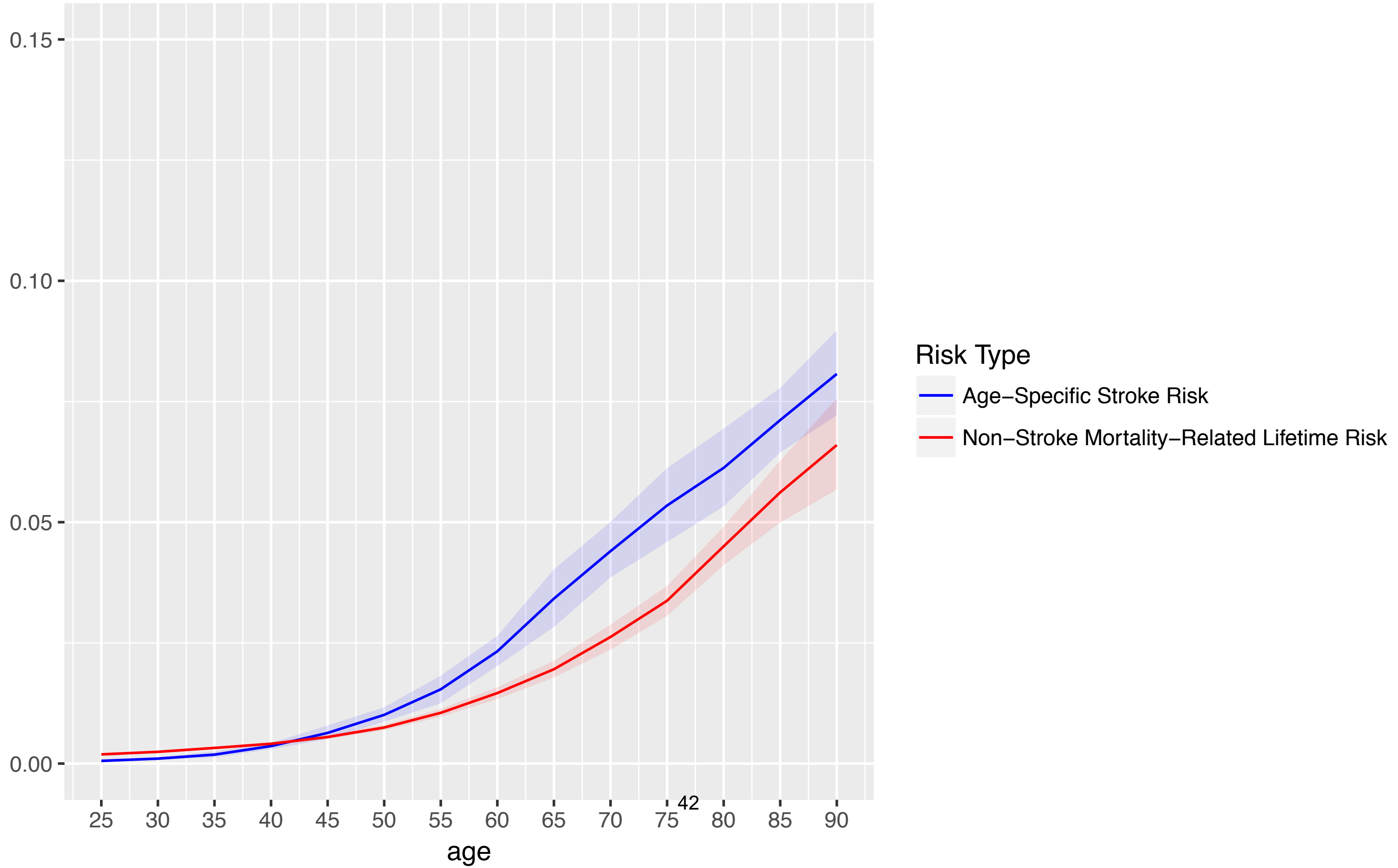

Figure S9O. Incident stroke vs non-stroke mortality by age, low-middle SDI, females, 2016

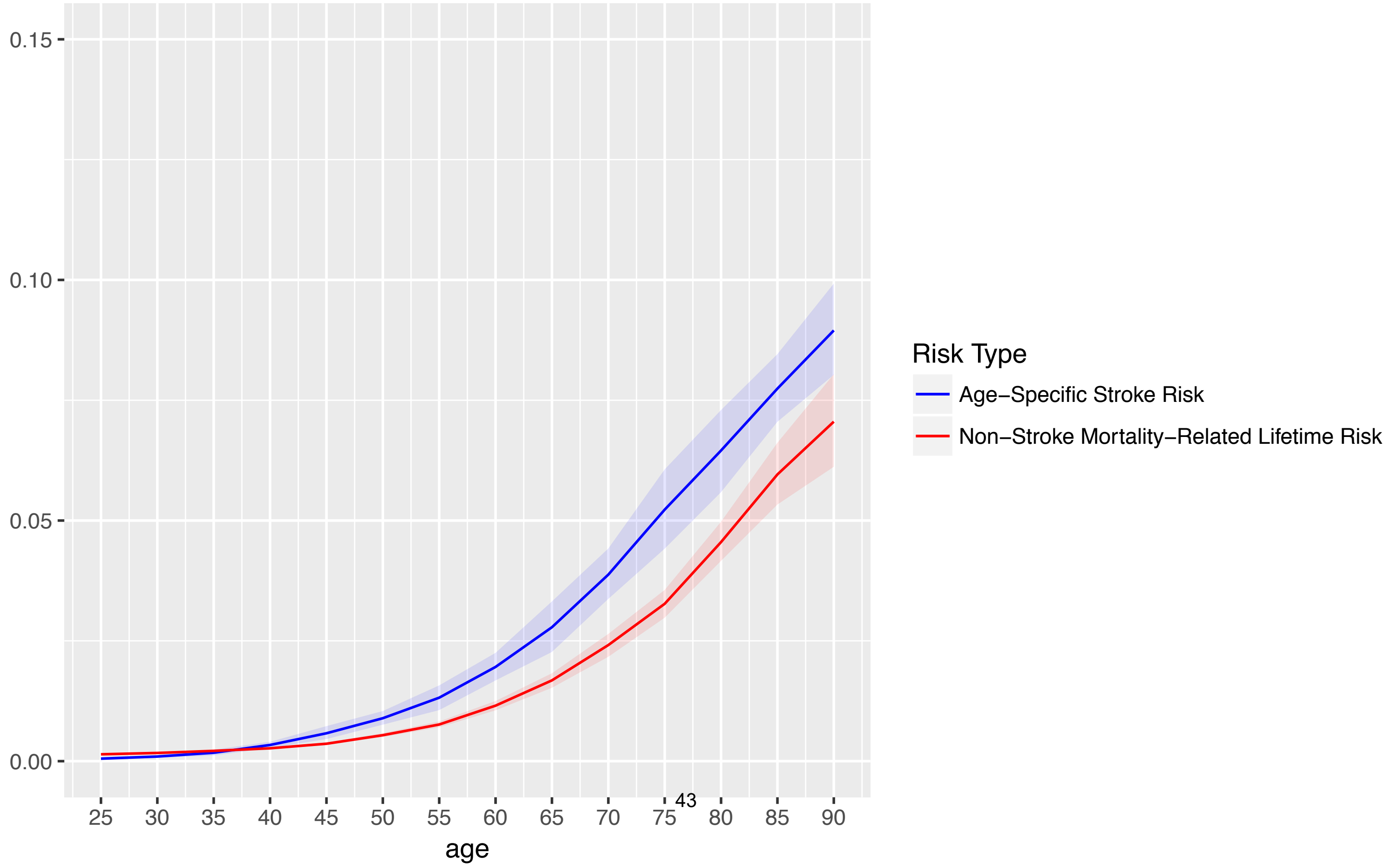

Figure S9P. Incident stroke vs non-stroke mortality by age, low SDI, both sexes, 2016

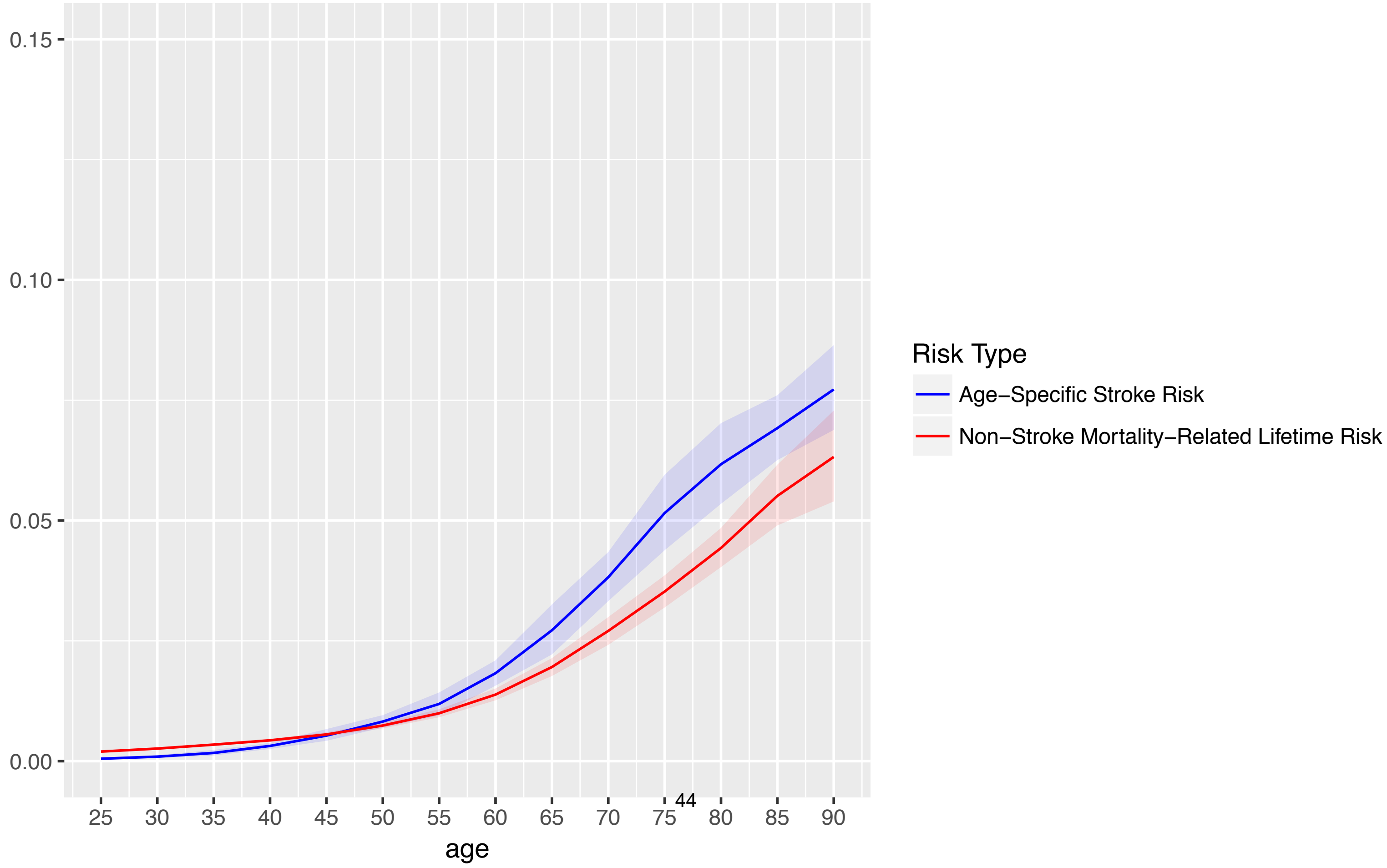

Figure S9Q. Incident stroke vs non-stroke mortality by age, low SDI, both sexes, 2016

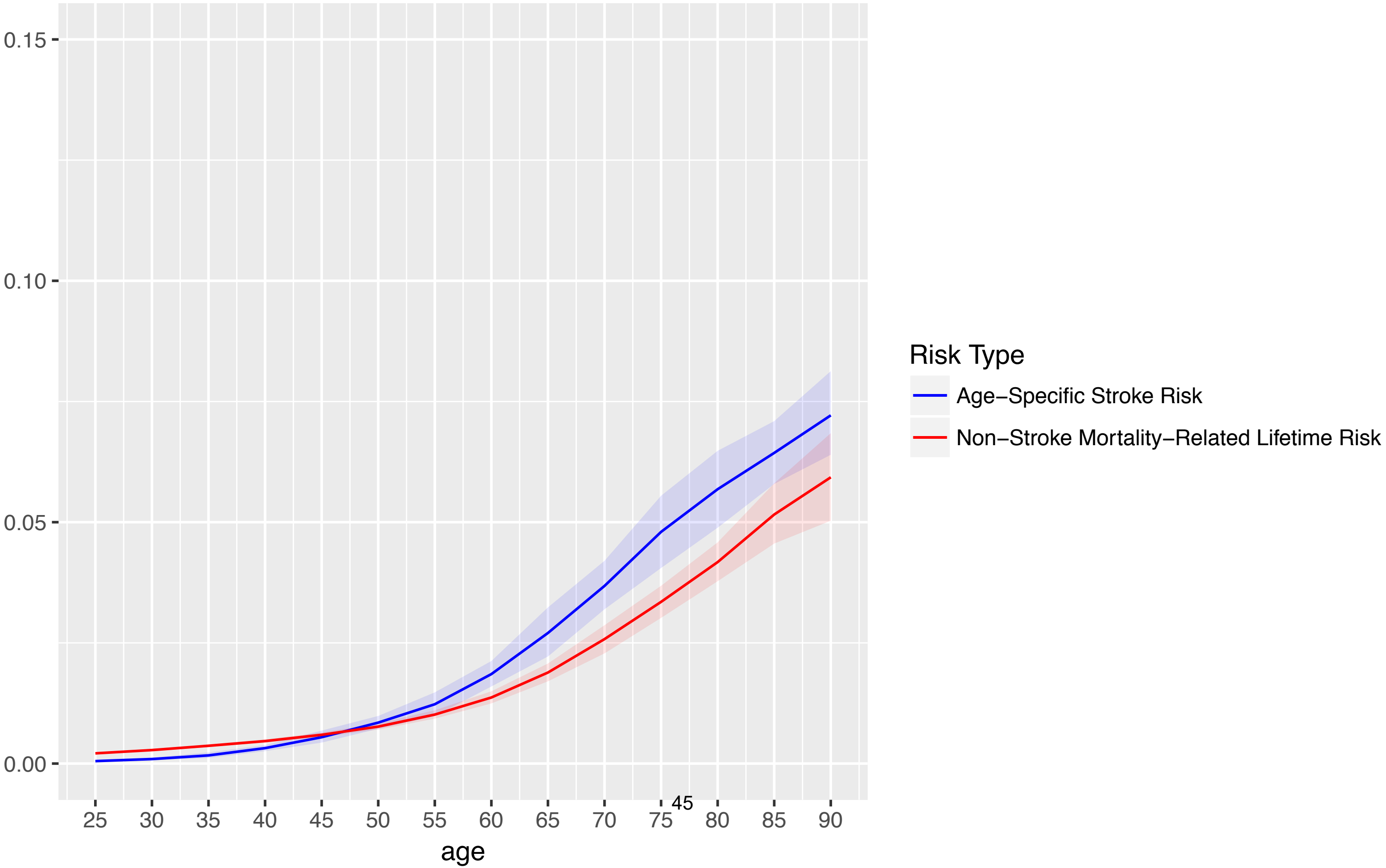

Figure S9R. Incident stroke vs non-stroke mortality by age, low SDI, both sexes, 2016

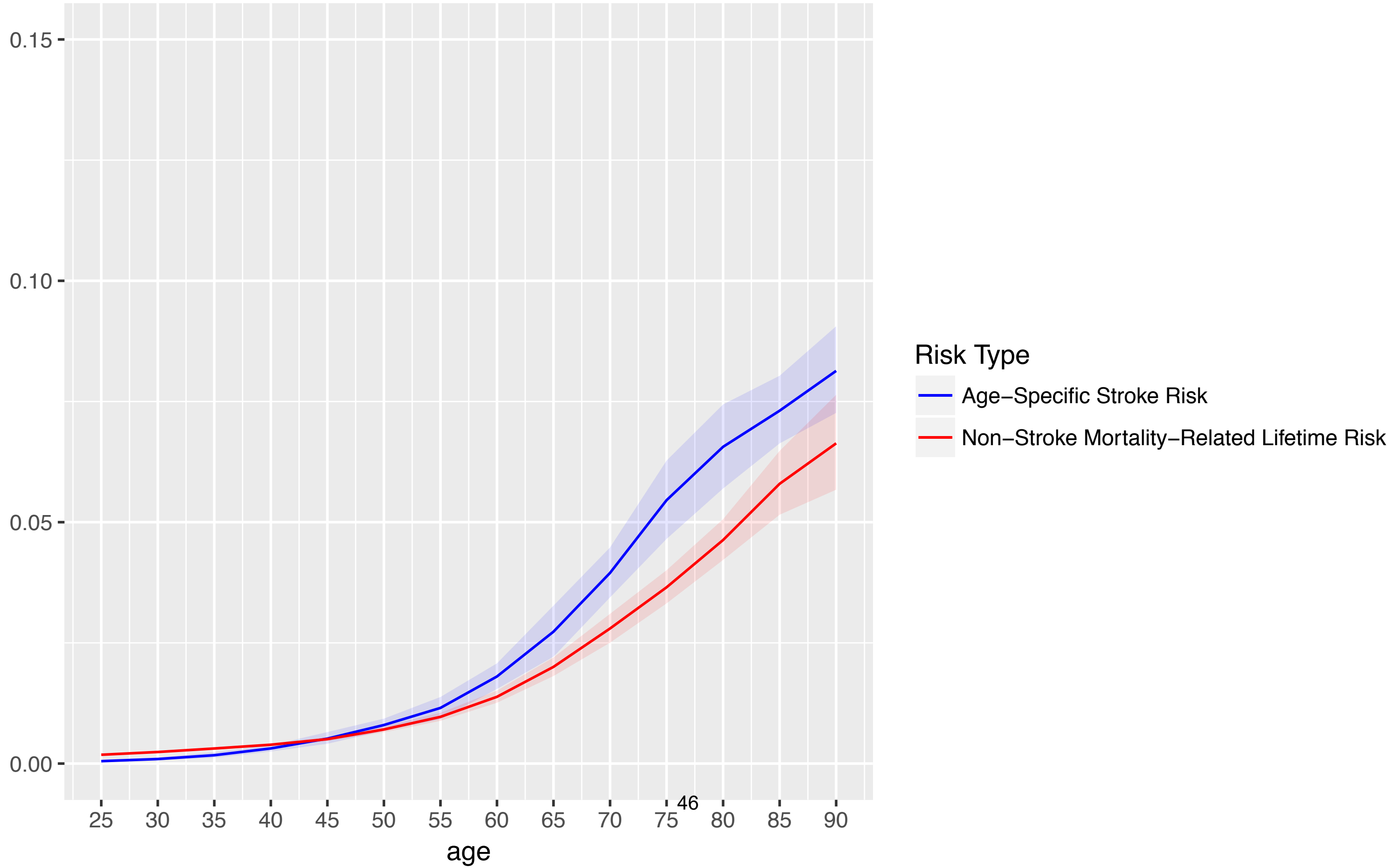

Figure S10A. Lifetime risk of Cerebrovascular disease for females, 2016.

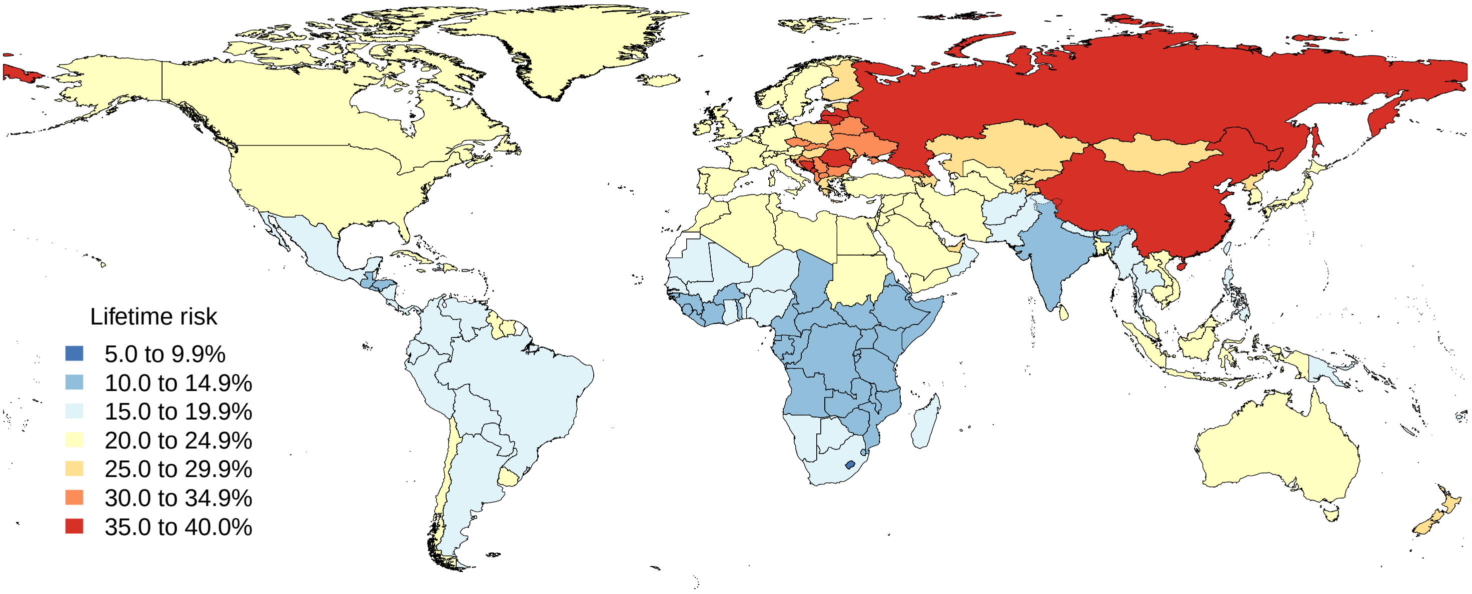

Figure S10B. Lifetime risk of Cerebrovascular disease for males, 2016.

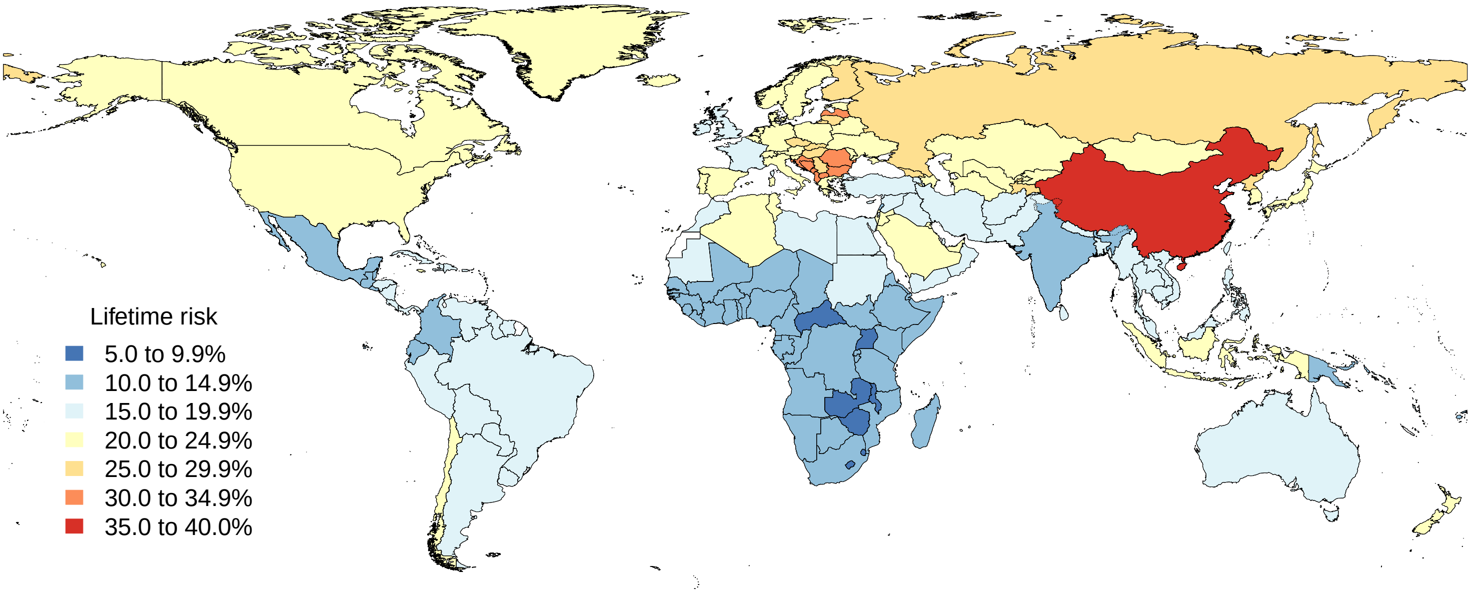

Figure S11A. Global lifetime risk of stroke occurrence by cause and age in men

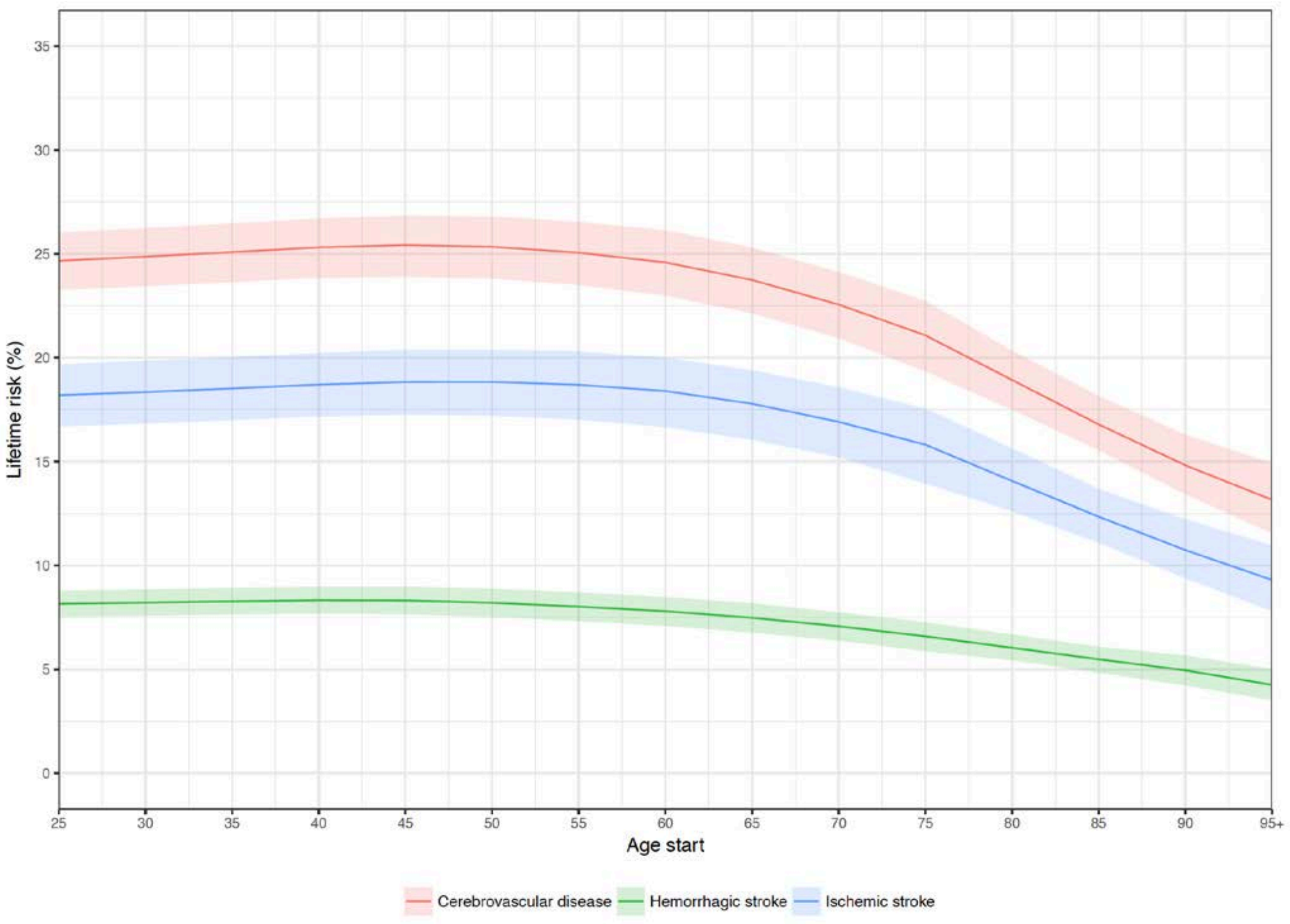

Figure S11B. Global lifetime risk of stroke occurrence by cause and age in women

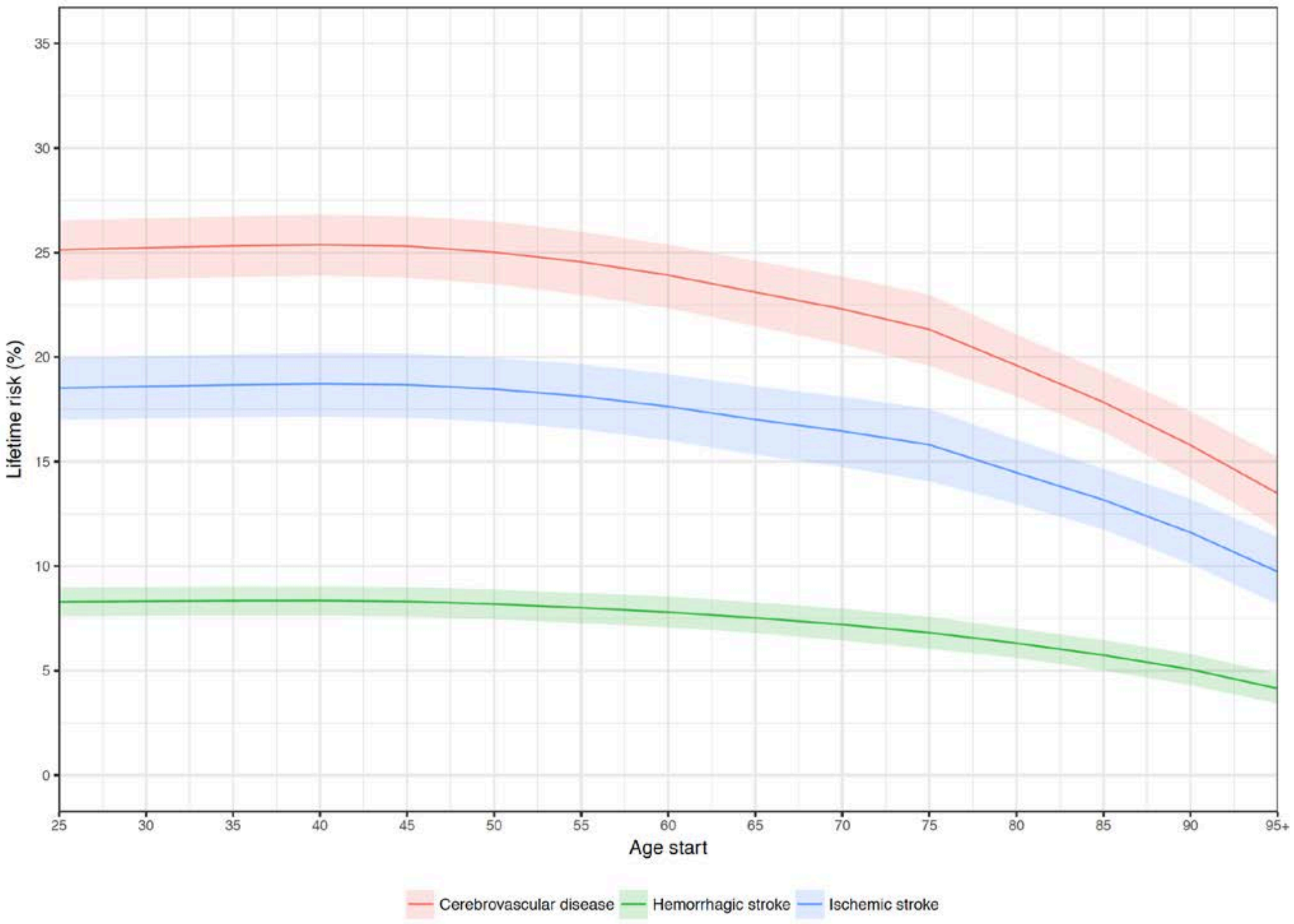

Table S1: GBD 2016 location hierarchy with levels	
**Location**	**Level**
Global	0
Southeast Asia, East Asia, and Oceania	1
East Asia	2
China	3
Anhui	4
Beijing	4
Chongqing	4
Fujian	4
Gansu	4
Guangdong	4
Guangxi	4
Guizhou	4
Hainan	4
Hebei	4
Heilongjiang	4
Henan	4
Hong Kong Special Administrative Region of China	4
Hubei	4
Hunan	4
Inner Mongolia	4
Jiangsu	4
Jiangxi	4
Jilin	4
Liaoning	4
Macao Special Administrative Region of China	4
Ningxia	4
Qinghai	4
Shaanxi	4
Shandong	4
Shanghai	4
Shanxi	4
Sichuan	4
Tianjin	4
Tibet	4
Xinjiang	4
Yunnan	4
Zhejiang	4
North Korea	3
Taiwan	3
Southeast Asia	2
Cambodia	3
Indonesia	3
Aceh	4
Bali	4
Bangka Belitung	4
Banten	4

Appendix Table 2: GBD 2016 location hierarchy with levels

Location	Level
Bengkulu	4
Gorontalo	4
Jakarta	4
Jambi	4
Jawa Barat	4
Jawa Tengah	4
Jawa Timur	4
Kalimantan Barat	4
Kalimantan Selatan	4
Kalimantan Tengah	4
Kalimantan Timur	4
Kalimantan Utara	4
Kepulauan Riau	4
Lampung	4
Maluku	4
Maluku Utara	4
Nusa Tenggara Barat	4
Nusa Tenggara Timur	4
Papua	4
Papua Barat	4
Riau	4
Sulawesi Barat	4
Sulawesi Selatan	4
Sulawesi Tengah	4
Sulawesi Tenggara	4
Sulawesi Utara	4
Sumatera Barat	4
Sumatera Selatan	4
Sumatera Utara	4
Yogyakarta	4
Laos	3
Malaysia	3
Maldives	3
Mauritius	3
Myanmar	3
Philippines	3
Sri Lanka	3
Seychelles	3
Thailand	3
Timor-Leste	3
Vietnam	3
Oceania	2
American Samoa	3
Federated States of Micronesia	3
Fiji	3
Guam	3

Appendix Table 2: GBD 2016 location hierarchy with levels	
**Location**	**Level**
Kiribati	3
Marshall Islands	3
Northern Mariana Islands	3
Papua New Guinea	3
Samoa	3
Solomon Islands	3
Tonga	3
Vanuatu	3
Central Europe, Eastern Europe, and Central Asia	1
Central Asia	2
Armenia	3
Azerbaijan	3
Georgia	4
Georgia	3
Kazakhstan	3
Kyrgyzstan	3
Mongolia	3
Tajikistan	3
Turkmenistan	3
Uzbekistan	3
Central Europe	2
Albania	3
Bosnia and Herzegovina	3
Bulgaria	3
Croatia	3
Czech Republic	3
Hungary	3
Macedonia	3
Montenegro	3
Poland	3
Romania	3
Serbia	3
Slovakia	3
Slovenia	3
Eastern Europe	2
Belarus	3
Estonia	3
Latvia	3
Lithuania	3
Moldova	3
Russia	3
Ukraine	3
High-income	1
High-income Asia Pacific	2
Brunei	3
Japan	3

Appendix Table 2: GBD 2016 location hierarchy with levels

Location	Level
Aichi	4
Akita	4
Aomori	4
Chiba	4
Ehime	4
Fukui	4
Fukuoka	4
Fukushima	4
Gifu	4
Gunma	4
Hiroshima	4
Hokkaidō	4
Hyōgo	4
Ibaraki	4
Ishikawa	4
Iwate	4
Kagawa	4
Kagoshima	4
Kanagawa	4
Kōchi	4
Kumamoto	4
Kyōto	4
Mie	4
Miyagi	4
Miyazaki	4
Nagano	4
Nagasaki	4
Nara	4
Niigata	4
Ôita	4
Okayama	4
Okinawa	4
Ōsaka	4
Saga	4
Saitama	4
Shiga	4
Shimane	4
Shizuoka	4
Tochigi	4
Tokushima	4
Tōkyō	4
Tottori	4
Toyama	4
Wakayama	4
Yamagata	4
Yamaguchi	4

Appendix Table 2: GBD 2016 location hierarchy with levels

Location	Level
Yamanashi	4
South Korea	3
Singapore	3
Australasia	2
Australia	3
New Zealand	3
Western Europe	2
Andorra	3
Austria	3
Belgium	3
Cyprus	3
Denmark	3
Finland	3
France	3
Germany	3
Greece	3
Iceland	3
Ireland	3
Israel	3
Italy	3
Luxembourg	3
Malta	3
Netherlands	3
Norway	3
Portugal	3
Spain	3
Sweden	3
Stockholm	4
Sweden except Stockholm	4
Switzerland	3
United Kingdom	3
England	4
East Midlands	5
Derby	6
Derbyshire	6
Leicester	6
Leicestershire	6
Lincolnshire	6
Northamptonshire	6
Nottingham	6
Nottinghamshire	6
Rutland	6
East of England	5
Bedford	6
Cambridgeshire	6
Central Bedfordshire	6

Appendix Table 2: GBD 2016 location hierarchy with levels

Location	Level
Essex	6
Hertfordshire	6
Luton	6
Norfolk	6
Peterborough	6
Southend-on-Sea	6
Suffolk	6
Thurrock	6
Greater London	5
Barking and Dagenham	6
Barnet	6
Bexley	6
Brent	6
Bromley	6
Camden	6
Croydon	6
Ealing	6
Enfield	6
Greenwich	6
Hackney	6
Hammersmith and Fulham	6
Haringey	6
Harrow	6
Havering	6
Hillingdon	6
Hounslow	6
Islington	6
Kensington and Chelsea	6
Kingston upon Thames	6
Lambeth	6
Lewisham	6
Merton	6
Newham	6
Redbridge	6
Richmond upon Thames	6
Southwark	6
Sutton	6
Tower Hamlets	6
Waltham Forest	6
Wandsworth	6
Westminster	6
North East England	5
County Durham	6
Darlington	6
Gateshead	6
Hartlepool	6

Appendix Table 2: GBD 2016 location hierarchy with levels

Location	Level
Middlesbrough	6
Newcastle upon Tyne	6
North Tyneside	6
Northumberland	6
Redcar and Cleveland	6
South Tyneside	6
Stockton-on-Tees	6
Sunderland	6
North West England	5
Blackburn with Darwen	6
Blackpool	6
Bolton	6
Bury	6
Cheshire East	6
Cheshire West and Chester	6
Cumbria	6
Halton	6
Knowsley	6
Lancashire	6
Liverpool	6
Manchester	6
Oldham	6
Rochdale	6
Salford	6
Sefton	6
St Helens	6
Stockport	6
Tameside	6
Trafford	6
Warrington	6
Wigan	6
Wirral	6
South East England	5
Bracknell Forest	6
Brighton and Hove	6
Buckinghamshire	6
East Sussex	6
Hampshire	6
Isle of Wight	6
Kent	6
Medway	6
Milton Keynes	6
Oxfordshire	6
Portsmouth	6
Reading	6
Slough	6

Appendix Table 2: GBD 2016 location hierarchy with levels	
**Location**	**Level**
Southampton	6
Surrey	6
West Berkshire	6
West Sussex	6
Windsor and Maidenhead	6
Wokingham	6
South West England	5
Bath and North East Somerset	6
Bournemouth	6
Bristol, City of	6
Cornwall	6
Devon	6
Dorset	6
Gloucestershire	6
North Somerset	6
Plymouth	6
Poole	6
Somerset	6
South Gloucestershire	6
Swindon	6
Torbay	6
Wiltshire	6
West Midlands	5
Birmingham	6
Coventry	6
Dudley	6
Herefordshire, County of	6
Sandwell	6
Shropshire	6
Solihull	6
Staffordshire	6
Stoke-on-Trent	6
Telford and Wrekin	6
Walsall	6
Warwickshire	6
Wolverhampton	6
Worcestershire	6
Yorkshire and the Humber	5
Barnsley	6
Bradford	6
Calderdale	6
Doncaster	6
East Riding of Yorkshire	6
Kingston upon Hull, City of	6
Kirklees	6
Leeds	6

Appendix Table 2: GBD 2016 location hierarchy with levels	
**Location**	**Level**
North East Lincolnshire	6
North Lincolnshire	6
North Yorkshire	6
Rotherham	6
Sheffield	6
Wakefield	6
York	6
Northern Ireland	4
Scotland	4
Wales	4
Southern Latin America	2
Argentina	3
Chile	3
Uruguay	3
High-income North America	2
Canada	3
Greenland	3
United States	3
Alabama	4
Alaska	4
Arizona	4
Arkansas	4
California	4
Colorado	4
Connecticut	4
Delaware	4
District of Columbia	4
Florida	4
Georgia	3
Georgia	4
Hawaii	4
Idaho	4
Illinois	4
Indiana	4
Iowa	4
Kansas	4
Kentucky	4
Louisiana	4
Maine	4
Maryland	4
Massachusetts	4
Michigan	4
Minnesota	4
Mississippi	4
Missouri	4
Montana	4

Appendix Table 2: GBD 2016 location hierarchy with levels	
**Location**	**Level**
Nebraska	4
Nevada	4
New Hampshire	4
New Jersey	4
New Mexico	4
New York	4
North Carolina	4
North Dakota	4
Ohio	4
Oklahoma	4
Oregon	4
Pennsylvania	4
Rhode Island	4
South Carolina	4
South Dakota	4
Tennessee	4
Texas	4
Utah	4
Vermont	4
Virginia	4
Washington	4
West Virginia	4
Wisconsin	4
Wyoming	4
Latin America and Caribbean	1
Caribbean	2
Antigua and Barbuda	3
The Bahamas	3
Barbados	3
Belize	3
Bermuda	3
Cuba	3
Dominica	3
Dominican Republic	3
Grenada	3
Guyana	3
Haiti	3
Jamaica	3
Puerto Rico	3
Saint Lucia	3
Saint Vincent and the Grenadines	3
Suriname	3
Trinidad and Tobago	3
Virgin Islands, U.S.	3
Andean Latin America	2
Bolivia	3

Appendix Table 2: GBD 2016 location hierarchy with levels	
**Location**	**Level**
Ecuador	3
Peru	3
Central Latin America	2
Colombia	3
Costa Rica	3
El Salvador	3
Guatemala	3
Honduras	3
Mexico	3
Aguascalientes	4
Baja California	4
Baja California Sur	4
Campeche	4
Chiapas	4
Chihuahua	4
Coahuila	4
Colima	4
Distrito Federal	4
Distrito Federal	4
Durango	4
Guanajuato	4
Guerrero	4
Hidalgo	4
Jalisco	4
México	4
Michoacán de Ocampo	4
Morelos	4
Nayarit	4
Nuevo León	4
Oaxaca	4
Puebla	4
Querétaro	4
Quintana Roo	4
San Luis Potosí	4
Sinaloa	4
Sonora	4
Tabasco	4
Tamaulipas	4
Tlaxcala	4
Veracruz de Ignacio de la Llave	4
Yucatán	4
Zacatecas	4
Nicaragua	3
Panama	3
Venezuela	3
Tropical Latin America	2

Appendix Table 2: GBD 2016 location hierarchy with levels	
**Location**	**Level**
Brazil	3
Acre	4
Alagoas	4
Amapá	4
Amazonas	4
Bahia	4
Ceará	4
Distrito Federal	4
Distrito Federal	4
Espírito Santo	4
Goiás	4
Maranhão	4
Mato Grosso	4
Mato Grosso do Sul	4
Minas Gerais	4
Pará	4
Paraíba	4
Paraná	4
Pernambuco	4
Piaui	4
Rio de Janeiro	4
Rio Grande do Norte	4
Rio Grande do Sul	4
Rondônia	4
Roraima	4
Santa Catarina	4
São Paulo	4
Sergipe	4
Tocantins	4
Paraguay	3
North Africa and Middle East	1
North Africa and Middle East	2
Afghanistan	3
Algeria	3
Bahrain	3
Egypt	3
Iran	3
Iraq	3
Jordan	3
Kuwait	3
Lebanon	3
Libya	3
Morocco	3
Palestine	3
Oman	3
Qatar	3

Appendix Table 2: GBD 2016 location hierarchy with levels	
**Location**	**Level**
Saudi Arabia	3
'Asir	4
Bahah	4
Eastern Province	4
Ha'il	4
Jawf	4
Jizan	4
Madinah	4
Makkah	4
Najran	4
Northern Borders	4
Qassim	4
Riyadh	4
Tabuk	4
Sudan	3
Syria	3
Tunisia	3
Turkey	3
United Arab Emirates	3
Yemen	3
South Asia	1
South Asia	2
Bangladesh	3
Bhutan	3
India	3
Andhra Pradesh	4
Andhra Pradesh, Rural	5
Andhra Pradesh, Urban	5
Arunāchal Pradesh	4
Arunāchal Pradesh, Rural	5
Arunāchal Pradesh, Urban	5
Assam	4
Assam, Rural	5
Assam, Urban	5
Bihār	4
Bihār, Rural	5
Bihār, Urban	5
Chhattīsgarh	4
Chhattīsgarh, Rural	5
Chhattīsgarh, Urban	5
Delhi	4
Delhi, Rural	5
Delhi, Urban	5
Goa	4
Goa, Rural	5
Goa, Urban	5

Appendix Table 2: GBD 2016 location hierarchy with levels

Location	Level
Gujarāt	4
Gujarāt, Rural	5
Gujarāt, Urban	5
Haryāna	4
Haryāna, Rural	5
Haryāna, Urban	5
Himachal Pradesh	4
Himachal Pradesh, Rural	5
Himachal Pradesh, Urban	5
Jammu and Kashmīr	4
Jammu and Kashmīr, Rural	5
Jammu and Kashmīr, Urban	5
Jharkhand	4
Jharkhand, Rural	5
Jharkhand, Urban	5
Karnātaka	4
Karnātaka, Rural	5
Karnātaka, Urban	5
Kerala	4
Kerala, Rural	5
Kerala, Urban	5
Madhya Pradesh	4
Madhya Pradesh, Rural	5
Madhya Pradesh, Urban	5
Mahārāshtra	4
Mahārāshtra, Rural	5
Mahārāshtra, Urban	5
Manipur	4
Manipur, Rural	5
Manipur, Urban	5
Meghālaya	4
Meghālaya, Rural	5
Meghālaya, Urban	5
Mizoram	4
Mizoram, Rural	5
Mizoram, Urban	5
Nāgāland	4
Nāgāland, Rural	5
Nāgāland, Urban	5
Orissa	4
Orissa, Rural	5
Orissa, Urban	5
Punjab	4
Punjab, Rural	5
Punjab, Urban	5
Rājasthān	4

Appendix Table 2: GBD 2016 location hierarchy with levels

Location	Level
Rājasthān, Rural	5
Rājasthān, Urban	5
Sikkim	4
Sikkim, Rural	5
Sikkim, Urban	5
Tamil Nādu	4
Tamil Nādu, Rural	5
Tamil Nādu, Urban	5
Telangana	4
Telangana, Rural	5
Telangana, Urban	5
Tripura	4
Tripura, Rural	5
Tripura, Urban	5
Uttar Pradesh	4
Uttar Pradesh, Rural	5
Uttar Pradesh, Urban	5
Uttarakhand	4
Uttarakhand, Rural	5
Uttarakhand, Urban	5
West Bengal	4
West Bengal, Rural	5
West Bengal, Urban	5
The Six Minor Territories	4
The Six Minor Territories, Rural	5
The Six Minor Territories, Urban	5
Nepal	3
Pakistan	3
Sub-Saharan Africa	1
Central Sub-Saharan Africa	2
Angola	3
Central African Republic	3
Congo	3
Democratic Republic of the Congo	3
Equatorial Guinea	3
Gabon	3
Eastern Sub-Saharan Africa	2
Burundi	3
Comoros	3
Djibouti	3
Eritrea	3
Ethiopia	3
Kenya	3
Baringo	4
Bomet	4
Bungoma	4

Appendix Table 2: GBD 2016 location hierarchy with levels	
**Location**	**Level**
Busia	4
Elgeyo-Marakwet	4
Embu	4
Garissa	4
HomaBay	4
Isiolo	4
Kajiado	4
Kakamega	4
Kericho	4
Kiambu	4
Kilifi	4
Kirinyaga	4
Kisii	4
Kisumu	4
Kitui	4
Kwale	4
Laikipia	4
Lamu	4
Machakos	4
Makueni	4
Mandera	4
Marsabit	4
Meru	4
Migori	4
Mombasa	4
Murang’a	4
Nairobi	4
Nakuru	4
Nandi	4
Narok	4
Nyamira	4
Nyandarua	4
Nyeri	4
Samburu	4
Siaya	4
TaitaTaveta	4
TanaRiver	4
TharakaNithi	4
TransNzoia	4
Turkana	4
UasinGishu	4
Vihiga	4
Wajir	4
WestPokot	4
Madagascar	3
Malawi	3

Appendix Table 2: GBD 2016 location hierarchy with levels	
**Location**	**Level**
Mozambique	3
Rwanda	3
Somalia	3
South Sudan	3
Tanzania	3
Uganda	3
Zambia	3
Southern Sub-Saharan Africa	2
Botswana	3
Lesotho	3
Namibia	3
South Africa	3
Eastern Cape	4
Free State	4
Gauteng	4
KwaZulu-Natal	4
Limpopo	4
Mpumalanga	4
North-West	4
Northern Cape	4
Western Cape	4
Swaziland	3
Zimbabwe	3
Western Sub-Saharan Africa	2
Benin	3
Burkina Faso	3
Cameroon	3
Cape Verde	3
Chad	3
Cote d'Ivoire	3
The Gambia	3
Ghana	3
Guinea	3
Guinea-Bissau	3
Liberia	3
Mali	3
Mauritania	3
Niger	3
Nigeria	3
Sao Tome and Principe	3
Senegal	3
Sierra Leone	3
Togo	3

Table S2. Proportion of indcident stroke subtypes by location, both sexes, in 2016				
Location	Female		Male	
	Ischemic stroke	Hemorrhagic stroke	Ischemic stroke	Hemorrhagic stroke
**Global**	70.05%	29.95%	70.01%	29.99%
**Southeast Asia, East Asia, and Oceania**	64.71%	35.29%	68.93%	31.07%
**East Asia**	65.20%	34.80%	69.86%	30.14%
China	65.30%	34.70%	69.95%	30.05%
North Korea	55.52%	44.48%	60.61%	39.39%
Taiwan	68.64%	31.36%	68.67%	31.33%
**Southeast Asia**	62.58%	37.42%	61.40%	38.60%
Cambodia	56.57%	43.43%	60.82%	39.18%
Indonesia	63.02%	36.98%	62.00%	38.00%
Laos	60.07%	39.93%	61.22%	38.78%
Malaysia	62.47%	37.53%	62.83%	37.17%
Maldives	67.13%	32.87%	66.10%	33.90%
Myanmar	63.99%	36.01%	61.50%	38.50%
Philippines	58.19%	41.81%	57.11%	42.89%
Sri Lanka	69.74%	30.26%	70.04%	29.96%
Thailand	65.31%	34.69%	62.85%	37.15%
Timor-Leste	60.07%	39.93%	61.73%	38.27%
Vietnam	60.97%	39.03%	60.00%	40.00%
Oceania	57.92%	42.08%	64.11%	35.89%
Fiji	61.12%	38.88%	67.71%	32.29%
Kiribati	62.52%	37.48%	64.52%	35.48%
Marshall Islands	60.43%	39.57%	64.36%	35.64%
Federated States of Micronesia	58.80%	41.20%	63.33%	36.67%
Papua New Guinea	56.91%	43.09%	63.50%	36.50%
Samoa	59.44%	40.56%	63.65%	36.35%
Solomon Islands	56.69%	43.31%	62.93%	37.07%
Tonga	61.38%	38.62%	66.94%	33.06%
Vanuatu	58.42%	41.58%	63.39%	36.61%
**Central Europe, Eastern Europe, and Central Asia**	79.27%	20.73%	76.54%	23.46%
**Central Asia**	65.53%	34.47%	62.80%	37.20%
Armenia	72.63%	27.37%	68.58%	31.42%
Azerbaijan	61.72%	38.28%	59.19%	40.81%
Georgia	64.85%	35.15%	62.44%	37.56%
Kazakhstan	71.80%	28.20%	69.55%	30.45%
Kyrgyzstan	67.40%	32.60%	66.69%	33.31%
Mongolia	48.19%	51.81%	43.29%	56.71%
Tajikistan	65.07%	34.93%	64.33%	35.67%
Turkmenistan	47.03%	52.97%	40.41%	59.59%
Uzbekistan	63.97%	36.03%	62.54%	37.46%
**Central Europe**	79.36%	20.64%	77.40%	22.60%
Albania	60.22%	39.78%	57.84%	42.16%
Bosnia and Herzegovina	82.90%	17.10%	81.45%	18.55%
Bulgaria	75.76%	24.24%	73.53%	26.47%
Croatia	79.80%	20.20%	79.64%	20.36%
Czech Republic	79.56%	20.44%	77.99%	22.01%
Hungary	81.38%	18.62%	79.65%	20.35%
Macedonia	74.64%	25.36%	73.32%	26.68%
Montenegro	57.43%	42.57%	59.13%	40.87%
Poland	82.00%	18.00%	79.95%	20.05%
Romania	77.38%	22.62%	75.70%	24.30%
Serbia	80.57%	19.43%	78.97%	21.03%
Slovakia	81.42%	18.58%	78.00%	22.00%
Slovenia	75.49%	24.51%	73.07%	26.93%
**Eastern Europe**	81.02%	18.98%	78.46%	21.54%
Belarus	80.28%	19.72%	77.23%	22.77%

Estonia	80.63%	19.37%	78.96%	21.04%
Latvia	82.32%	17.68%	80.41%	19.59%
Lithuania	83.12%	16.88%	81.00%	19.00%
Moldova	77.16%	22.84%	74.02%	25.98%
Russian Federation	81.00%	19.00%	78.56%	21.44%
Ukraine	81.29%	18.71%	78.40%	21.60%
**High-income**	70.57%	29.43%	69.00%	31.00%
**High-income Asia Pacific**	68.29%	31.71%	68.03%	31.97%
Brunei	65.77%	34.23%	67.74%	32.26%
Japan	68.16%	31.84%	67.40%	32.60%
South Korea	68.56%	31.44%	69.93%	30.07%
Singapore	69.93%	30.07%	70.98%	29.02%
Australasia	66.34%	33.66%	68.52%	31.48%
Australia	65.41%	34.59%	67.99%	32.01%
New Zealand	70.01%	29.99%	70.51%	29.49%
**Western Europe**	68.11%	31.89%	67.08%	32.92%
Andorra	66.22%	33.78%	63.67%	36.33%
Austria	68.98%	31.02%	69.16%	30.84%
Belgium	70.18%	29.82%	68.14%	31.86%
Cyprus	60.03%	39.97%	58.19%	41.81%
Denmark	66.76%	33.24%	65.88%	34.12%
Finland	71.17%	28.83%	71.34%	28.66%
France	67.99%	32.01%	66.21%	33.79%
Germany	71.67%	28.33%	71.27%	28.73%
Greece	65.16%	34.84%	63.18%	36.82%
Iceland	71.55%	28.45%	70.79%	29.21%
Ireland	65.83%	34.17%	65.56%	34.44%
Israel	67.96%	32.04%	66.09%	33.91%
Italy	66.52%	33.48%	64.72%	35.28%
Luxembourg	65.98%	34.02%	67.96%	32.04%
Malta	62.25%	37.75%	59.24%	40.76%
Netherlands	67.04%	32.96%	66.79%	33.21%
Norway	68.04%	31.96%	67.40%	32.60%
Portugal	66.80%	33.20%	63.38%	36.62%
Spain	66.86%	33.14%	65.24%	34.76%
Sweden	69.28%	30.72%	69.12%	30.88%
Switzerland	65.29%	34.71%	66.88%	33.12%
United Kingdom	65.21%	34.79%	65.05%	34.95%
**Southern Latin America**	70.51%	29.49%	67.25%	32.75%
Argentina	70.15%	29.85%	66.95%	33.05%
Chile	70.76%	29.24%	67.31%	32.69%
Uruguay	72.32%	27.68%	69.66%	30.34%
**High-income North America**	74.67%	25.33%	72.16%	27.84%
Canada	74.92%	25.08%	73.12%	26.88%
United States	74.64%	25.36%	72.06%	27.94%
**Latin America and Caribbean**	67.95%	32.05%	71.40%	28.60%
Caribbean	67.10%	32.90%	67.81%	32.19%
Antigua and Barbuda	66.08%	33.92%	64.23%	35.77%
The Bahamas	66.28%	33.72%	65.42%	34.58%
Barbados	67.05%	32.95%	66.17%	33.83%
Belize	67.08%	32.92%	66.12%	33.88%
Cuba	70.34%	29.66%	70.43%	29.57%
Dominica	66.51%	33.49%	65.25%	34.75%
Dominican Republic	67.31%	32.69%	66.47%	33.53%
Grenada	65.23%	34.77%	65.81%	34.19%
Guyana	62.30%	37.70%	62.97%	37.03%
Haiti	63.47%	36.53%	67.57%	32.43%
Jamaica	64.79%	35.21%	65.47%	34.53%

Saint Lucia	64.82%	35.18%	64.58%	35.42%
Saint Vincent and the Grenadines	64.96%	35.04%	64.02%	35.98%
Suriname	62.62%	37.38%	62.44%	37.56%
Trinidad and Tobago	66.16%	33.84%	65.83%	34.17%
**Andean Latin America**	69.04%	30.96%	70.92%	29.08%
Bolivia	67.72%	32.28%	70.11%	29.89%
Ecuador	68.48%	31.52%	70.10%	29.90%
Peru	69.76%	30.24%	71.65%	28.35%
**Central Latin America**	66.67%	33.33%	67.95%	32.05%
Colombia	65.35%	34.65%	67.12%	32.88%
Costa Rica	69.34%	30.66%	69.60%	30.40%
El Salvador	68.00%	32.00%	70.16%	29.84%
Guatemala	67.29%	32.71%	68.93%	31.07%
Honduras	71.18%	28.82%	70.43%	29.57%
Mexico	67.35%	32.65%	68.85%	31.15%
Nicaragua	66.88%	33.12%	67.85%	32.15%
Panama	66.52%	33.48%	66.14%	33.86%
Venezuela	64.13%	35.87%	64.78%	35.22%
**Tropical Latin America**	68.97%	31.03%	74.92%	25.08%
Brazil	69.29%	30.71%	75.31%	24.69%
Paraguay	56.35%	43.65%	60.54%	39.46%
**North Africa and Middle East**	76.55%	23.45%	74.82%	25.18%
Algeria	76.20%	23.80%	73.81%	26.19%
Bahrain	77.71%	22.29%	76.44%	23.56%
Egypt	76.37%	23.63%	74.19%	25.81%
Iran	82.58%	17.42%	81.94%	18.06%
Iraq	74.56%	25.44%	73.56%	26.44%
Jordan	80.43%	19.57%	79.02%	20.98%
Kuwait	81.13%	18.87%	78.64%	21.36%
Lebanon	77.09%	22.91%	78.94%	21.06%
Libya	76.93%	23.07%	74.27%	25.73%
Morocco	76.29%	23.71%	73.53%	26.47%
Palestine	76.83%	23.17%	74.44%	25.56%
Oman	68.23%	31.77%	62.08%	37.92%
Qatar	77.09%	22.91%	76.09%	23.91%
Saudi Arabia	77.44%	22.56%	76.50%	23.50%
Syria	75.00%	25.00%	71.14%	28.86%
Tunisia	76.79%	23.21%	74.25%	25.75%
Turkey	75.58%	24.42%	72.88%	27.12%
United Arab Emirates	76.61%	23.39%	76.87%	23.13%
Yemen	74.10%	25.90%	72.29%	27.71%
**South Asia**	71.05%	28.95%	69.43%	30.57%
Afghanistan	72.20%	27.80%	69.03%	30.97%
Bangladesh	69.19%	30.81%	68.81%	31.19%
Bhutan	69.74%	30.26%	68.88%	31.12%
India	71.36%	28.64%	69.71%	30.29%
Nepal	70.28%	29.72%	69.08%	30.92%
Pakistan	70.89%	29.11%	68.25%	31.75%
**Sub-Saharan Africa**	67.48%	32.52%	66.21%	33.79%
**Central Sub-Saharan Africa**	64.92%	35.08%	64.95%	35.05%
Angola	65.98%	34.02%	65.88%	34.12%
**Central African Republic**	66.47%	33.53%	62.41%	37.59%
Congo	66.52%	33.48%	67.31%	32.69%
Democratic Republic of the Congo	64.22%	35.78%	64.67%	35.33%
Equatorial Guinea	70.62%	29.38%	69.14%	30.86%
Gabon	67.30%	32.70%	66.23%	33.77%
**Eastern Sub-Saharan Africa**	65.43%	34.57%	65.42%	34.58%
Burundi	63.22%	36.78%	63.89%	36.11%

Comoros	67.16%	32.84%	67.25%	32.75%
Djibouti	69.94%	30.06%	66.90%	33.10%
Eritrea	65.64%	34.36%	65.24%	34.76%
Ethiopia	62.66%	37.34%	63.37%	36.63%
Kenya	68.26%	31.74%	67.04%	32.96%
Madagascar	63.12%	36.88%	61.81%	38.19%
Malawi	68.90%	31.10%	69.22%	30.78%
Mauritius	62.58%	37.42%	61.77%	38.23%
Mozambique	67.70%	32.30%	66.02%	33.98%
Rwanda	66.72%	33.28%	68.27%	31.73%
Seychelles	64.76%	35.24%	65.62%	34.38%
Somalia	63.77%	36.23%	65.52%	34.48%
Tanzania	66.95%	33.05%	68.48%	31.52%
Uganda	65.70%	34.30%	64.43%	35.57%
Zambia	68.45%	31.55%	66.12%	33.88%
**Southern Sub-Saharan Africa**	69.53%	30.47%	66.47%	33.53%
Botswana	71.18%	28.82%	67.47%	32.53%
Lesotho	65.58%	34.42%	59.32%	40.68%
Namibia	70.06%	29.94%	65.92%	34.08%
South Africa	69.99%	30.01%	67.14%	32.86%
Swaziland	71.23%	28.77%	65.80%	34.20%
Zimbabwe	67.72%	32.28%	64.87%	35.13%
**Western Sub-Saharan Africa**	69.67%	30.33%	67.40%	32.60%
Benin	67.58%	32.42%	64.55%	35.45%
Burkina Faso	67.10%	32.90%	63.77%	36.23%
Cameroon	67.97%	32.03%	64.17%	35.83%
Cape Verde	70.24%	29.76%	64.64%	35.36%
Chad	68.66%	31.34%	65.46%	34.54%
Cote d'Ivoire	67.56%	32.44%	63.41%	36.59%
The Gambia	69.24%	30.76%	66.18%	33.82%
Ghana	68.88%	31.12%	68.14%	31.86%
Guinea	67.89%	32.11%	64.52%	35.48%
Guinea-Bissau	67.39%	32.61%	62.71%	37.29%
Liberia	66.80%	33.20%	65.26%	34.74%
Mali	67.43%	32.57%	66.47%	33.53%
Mauritania	72.52%	27.48%	70.38%	29.62%
Niger	68.34%	31.66%	66.55%	33.45%
Nigeria	71.80%	28.20%	70.00%	30.00%
Sao Tome and Principe	69.61%	30.39%	66.94%	33.06%
Senegal	69.07%	30.93%	65.73%	34.27%
Sierra Leone	67.55%	32.45%	65.63%	34.37%
Togo	68.54%	31.46%	66.08%	33.92%
American Samoa	61.42%	38.58%	65.15%	34.85%
Bermuda	69.12%	30.88%	67.88%	32.12%
Greenland	68.55%	31.45%	66.19%	33.81%
Guam	64.95%	35.05%	67.08%	32.92%
Northern Mariana Islands	59.77%	40.23%	62.95%	37.05%
Puerto Rico	67.57%	32.43%	67.27%	32.73%
Virgin Islands, U.S.	67.11%	32.89%	65.91%	34.09%
South Sudan	66.49%	33.51%	66.75%	33.25%
Sudan	75.02%	24.98%	72.99%	27.01%

Table S3. Lifetime risk of stroke (LTR in %) (with 95% UI) globally and regionally (21 GBD regions and 7 super regions) in 2016 and its percentage change (with 95% UI) from 1990 to 2016 by pathological type of stroke and sex

GBD super regions	GBD regions	Stroke types	Men		Women		Both sexes	
			LTR (95% UI)	Percentage change (95% CI) 1990-2015	LTR (95% UI)	Percentage change (95% CI) 1990-2015	LTR (95% UI)	Percentage change (95% CI) 1990-2015
Global		Ischemic stroke	18.2 (16.7,19.7)	21.2 (17.1, 25.0)	18.5 (17.0, 20.0)	5.5 (1.3, 9.7)	18.3 (16.9, 19.8)	12.7 (8.9, 16.3)
		Hemorrhagic stroke	8.2 (7.5, 8.8)	7.5 (3.8, 11.1)	8.3 (7.6, 9.0)	0.8 (-3.4, 5.0)	8.2 (7.5, 8.9)	4.0 (0.2, 7.6)
		Total strokes	24.7 (23.3,26.0)	15.4 (12.5, 18.2)	25.1 (23.7, 26.5)	3.2 (0.2, 6.1)	24.9 (23.5, 26.2)	8.9 (6.2, 11.5)
High-income	Southern Latin America	Ischemic stroke	12.7 (11.3,14.1)	-20.5 (-27.4, -13.1)	15.6 (14.0,17.3)	-18.0 (-25.2, -10.5)	14.2 (12.8, 15.6)	-18.6 (-24.9, -12.3)
		Hemorrhagic stroke	6.0 (5.4, 6.7)	4.7 (-4.8, 14.2)	6.0 (5.4, 6.7)	-2.4 (-10.2, 5.8)	6.0 (5.4, 6.6)	0.8 (-6.0, 7.9)
		Total strokes	17.8 (16.3, 19.3)	-14.2 (-20.4, -7.6)	20.6 (18.9, 22.3)	-14.5 (-20.7, -8.4)	19.2 (17.8, 20.5)	-14.1 (-19.0, -8.7)
	Western Europe	Ischemic stroke	15.9 (14.5, 17.2)	3.1 (-2.3, 8.4)	17.2 (15.8, 18.5)	-3.7 (-8.6, 1.6)	16.5 (15.2, 17.8)	-0.5 (-5.3, 4.3)
		Hemorrhagic stroke	8.1 (7.5, 8.7)	8.8 (3.6, 14.2)	7.8 (7.2, 8.5)	-4.9 (-10.2, 0.4)	7.9 (7.3, 8.5)	1.2 (-3.8, 6.1)
		Total strokes	22.2 (20.9, 23.4)	4.2 (0.3, 8.2)	23.3 (21.9, 24.6)	-4.3 (-7.9, -0.4)	22.7 (21.4, 23.9)	-0.4 (-3.6, 3.1)
	High-income North America	Ischemic stroke	17.4 (15.9, 18.8)	1.0 (-3.3, 5.9)	20.1 (18.5, 21.5)	-1.5 (-5.6, 2.8)	18.8 (17.3, 20.1)	-0.1 (-4.0, 4.1)
		Hemorrhagic stroke	6.8 (6.2, 7.3)	19.3 (13.9, 24.6)	6.7 (6.2, 7.3)	9.4 (4.6, 14.1)	6.7 (6.2, 7.3)	14.2 (9.6, 18.9)
		Total strokes	22.4 (21.1, 23.7)	4.9 (1.7, 8.7)	25.1 (23.6, 26.4)	0.5 (-2.8, 3.8)	23.8 (22.4, 25.0)	2.7 (-0.3, 5.9)
	Australasia	Ischemic stroke	15.0 (13.5, 16.4)	6.0 (-2.7, 14.8)	16.1 (14.7,17.8)	1.7 (-6.3, 10.1)	15.6 (14.2, 17.0)	3.9 (-2.8, 11.6)
		Hemorrhagic stroke	7.5 (6.8, 8.3)	15.3 (5.4, 25.5)	8.7 (7.9, 9.6)	2.3 (-6.1, 11.7)	8.1 (7.4, 8.9)	7.8 (0.4, 16.0)
		Total strokes	20.9 (19.4, 22.4)	8.1 (1.1, 14.8)	23.0 (21.5, 24.7)	1.4 (-4.6, 7.9)	21.9 (20.6, 23.4)	4.7 (-0.5, 10.1)
	High-income Asia Pacific	Ischemic stroke	16.2 (14.6, 17.8)	-13.4 (-19.4, -7.6)	17.4 (15.8, 19.2)	-15.2 (-21.0, -9.6)	16.8 (15.2, 18.3)	-14.3 (-19.4, -9.5)
		Hemorrhagic stroke	7.5 (6.8, 8.2)	-5.6 (-11.9, 0.8)	7.6 (6.8, 8.4)	-14.1 (-20.2, -7.8)	7.5 (6.8, 8.2)	-10.8 (-16.6, -4.9)
		Total strokes	22.2 (20.6, 23.8)	-11.4 (-16.3, -6.6)	23.5 (21.8, 25.2)	-15.1 (-19.7, -10.6)	22.8 (21.2, 24.3)	-13.5 (-17.4, -9.4)
Latin America and Caribbean	Caribbean	Ischemic stroke	12.8 (11.5,14.1)	-0.1 (-7.2, 6.6)	14.8 (13.2,16.3)	-0.4 (-7.6, 7.3)	13.8 (12.4,15.1)	-0.2 (-6.3, 5.9)
		Hemorrhagic stroke	6.0 (5.5, 6.6)	5.6 (-0.7, 12.8)	7.1 (6.4, 7.8)	1.3 (-5.4, 8.3)	6.5 (6.0, 7.2)	3.3 (-1.9, 9.0)
		Total strokes	18.0 (16.6,19.3)	1.3 (-4.5, 6.8)	20.8 (19.3, 22.3)	-0.3 (-6.1, 5.9)	19.4 (18.0,20.7)	0.5 (-4.1, 5.4)
	Central Latin America	Ischemic stroke	10.0 (9.0,11.0)	-0.2 (-5.9, 5.5)	11.5 (10.3, 12.7)	-1.8 (-7.3, 3.8)	10.7 (9.7, 11.8)	-1.0 (-6.1, 4.1)
		Hemorrhagic stroke	4.7 (4.2, 5.1)	1.1 (-4.3, 6.2)	5.7 (5.2, 6.2)	-3.0 (-8.0, 2.3)	5.2 (4.7, 5.7)	-1.1 (-6.1, 3.7)
		Total strokes	14.1 (13.1, 15.1)	0.0 (-4.3, 4.3)	16.4 (15.2, 17.6)	-2.4 (-6.4, 1.7)	15.2 (14.2, 16.4)	-1.3 (-4.8, 2.6)
	Tropical Latin America	Ischemic stroke	14.9 (13.6, 16.3)	-9.6 (-14.1, -4.8)	14.7 (13.3, 16.1)	-17.3 (-22.1,-12.0)	14.7 (13.4, 16.2)	-13.6 (-17.8, -9.2)
		Hemorrhagic stroke	4.8 (4.3, 5.2)	-10.3 (-14.9, -5.5)	5.8 (5.2, 6.3)	-6.9 (-11.5, -1.7)	5.3 (4.8, 5.7)	-8.3 (-12.4, -3.8)
		Total strokes	18.9 (17.6, 20.2)	-10.4 (-13.9, -6.6)	19.5 (18.1, 20.9)	-15.1 (-18.8, -11.0)	19.1 (17.9,20.5)	-12.8 (-16.0, -9.2)
	Andean Latin America	Ischemic stroke	11.5 (10.0, 12.9)	-1.5 (-11.2, 8.5)	13.1 (11.5, 14.7)	-0.7 (-9.7, 8.9)	12.3 (10.9,13.6)	-1.0 (-8.5, 7.4)
		Hemorrhagic stroke	4.7 (4.1, 5.2)	2.4 (-6.7, 12.2)	5.7 (5.1, 6.4)	3.7 (-5.7, 13.9)	5.2 (4.6, 5.7)	3.3 (-4.2, 11.3)
		Total strokes	15.5 (14.0, 17.0)	-0.9 (-9.0, 8.1)	17.9 (16.2, 19.6)	-0.1 (-7.8, 8.1)	16.7 (15.2,18.2)	-0.3 (-6.7, 6.5)
Sub-Saharan Africa	Central Sub-Saharan Africa	Ischemic stroke	7.7 (6.7, 8.7)	18.9 (8.4, 29.8)	9.1 (8.0, 10.3)	4.8 (-4.7, 15.0)	8.4 (7.4, 9.4)	10.6 (2.1, 19.1)
		Hemorrhagic stroke	4.2 (3.7, 4.8)	2.4 (-7.0, 11.6)	5.1 (4.5, 5.6)	-4.6 (-13.9, 4.9)	4.7 (4.2, 5.2)	-1.5 (-9.7, 6.3)
		Total strokes	11.6 (10.6, 12.7)	12.4 (3.8, 20.8)	13.8 (12.6, 15.1)	1.4 (-6.9, 9.4)	12.8 (11.7, 13.8)	6.1 (-0.9, 12.9)
	Eastern Sub-Saharan Africa	Ischemic stroke	7.4 (6.5, 8.4)	23.2 (13.4, 34.4)	8.2 (7.3, 9.2)	19.3 (10.0, 28.5)	7.9 (6.9, 8.8)	20.9 (12.7, 29.1)
		Hemorrhagic stroke	4.0 (3.6, 4.5)	1.0 (-7.2, 9.4)	4.5 (4.0, 5.0)	-9.3 (-15.5, -2.0)	4.3 (3.8, 4.8)	-4.9 (-10.7, 1.8)
		Total strokes	11.2 (10.3, 12.3)	13.8 (5.4, 22.5)	12.5 (11.4, 13.6)	6.7 (-0.1, 13.9)	11.8 (10.9, 12.8)	9.8 (3.8, 16.1)
	Southern Sub-Saharan Africa	Ischemic stroke	6.8 (6.1, 7.6)	-16.0 (-22.7, -9.3)	10.7 (9.6,11.8)	-10.3 (-15.8, -4.5)	8.8 (7.9, 9.7)	-12.3 (-17.3, -7.1)
		Hemorrhagic stroke	3.5 (3.1, 3.9)	-22.7 (-29.2, -16.9)	4.9 (4.4, 5.4)	-20.9 (-26.1, -15.5)	4.2 (3.8, 4.6)	-21.5 (-26.3, -16.7)
		Total strokes	10.0 (9.2, 10.9)	-18.1 (-23.8, -12.3)	14.9 (13.7, 16.1)	-14.0 (-18.9, -9.0)	12.5 (11.6, 13.5)	-15.4 (-19.9, -11.1)
	Western Sub-Saharan Africa	Ischemic stroke	9.0 (8.0, 10.1)	13.8 (4.6, 23.9)	11.4 (10.1,12.7)	9.6 (0.5, 19.6)	10.2 (9.1, 11.4)	10.5 (3.7, 18.6)
		Hemorrhagic stroke	4.4 (3.9, 4.9)	5.1 (-3.8, 13.6)	5.0 (4.4, 5.6)	2.7 (-5.8, 12.2)	4.7 (4.2, 5.2)	3.4 (-3.3, 10.5)
		Total strokes	13.0 (11.9, 14.2)	10.5 (2.3, 19.1)	15.8 (14.5, 17.3)	7.0 (-0.6, 15.8)	14.4 (13.3, 15.7)	7.9 (2.0, 14.4)
North Africa and Middle East	North Africa and Middle East	Ischemic stroke	15.0 (13.5, 16.5)	12.8 (6.4, 19.3)	18.3 (16.5, 20.1)	6.5 (1.0, 11.9)	16.6 (15.0, 18.2)	9.1 (4.2, 14.1)
		Hemorrhagic stroke	5.2 (4.7, 5.8)	3.8 (-2.4, 10.1)	5.9 (5.3, 6.6)	-3.2 (-8.6, 2.5)	5.6 (5.0, 6.1)	-0.1 (-4.8, 4.9)
		Total strokes	19.4 (17.8, 20.9)	10.2 (4.7, 15.7)	23.1 (21.4, 24.8)	3.7 (-0.8, 8.0)	21.2 (19.6, 22.8)	6.4 (2.5, 10.5)
South Asia	South Asia	Ischemic stroke	9.6 (8.6, 10.6)	23.6 (17.7, 29.0)	11.6 (10.4, 12.9)	27.8 (21.6, 34.2)	10.6 (9.5, 11.6)	25.7 (20.4, 30.7)
		Hemorrhagic stroke	4.2 (3.8, 4.7)	1.9 (-3.2, 6.9)	4.7 (4.3, 5.3)	5.7 (0.7, 10.8)	4.5 (4.0, 4.9)	3.7 (-0.9, 8.2)
		Total strokes	13.5 (12.5, 14.5)	15.6 (11.0, 20.0)	15.9 (14.7, 17.1)	19.6 (14.5, 24.6)	14.6 (13.6, 15.7)	17.6 (13.6, 21.3)
Southeast Asia, East Asia, and Oceania	East Asia	Ischemic stroke	30.5 (28.4, 32.6)	59.0 (53.3, 65.0)	24.6 (22.6, 26.4)	33.9 (28.8, 38.9)	28.0 (26.0, 30.0)	49.6 (44.7, 54.2)
		Hemorrhagic stroke	14.9 (13.9, 16.1)	13.2 (8.2, 18.6)	15.8 (14.6, 17.0)	10.4 (4.9, 15.8)	15.3 (14.2, 16.4)	11.4 (6.8, 15.8)
		Total Strokes	40.6 (38.7, 42.3)	35.9 (31.9, 39.8)	36.3 (34.5, 38.1)	20.7 (16.6, 24.4)	38.8 (37.0, 40.6)	29.7 (26.1, 33.0)
	Southeast Asia	Ischemic stroke	12.4 (11.1, 13.7)	14.6 (8.8, 20.7)	12.8 (11.6, 14.1)	19.6 (13.9, 25.5)	12.6 (11.4, 13.8)	17.0 (12.0, 21.9)
		Hemorrhagic stroke	7.9 (7.2, 8.7)	-3.8 (-8.7, 1.5)	8.2 (7.5, 8.9)	7.9 (1.8, 13.9)	8.1 (7.4, 8.8)	1.9 (-2.8, 6.6)
		Total Strokes	19.6 (18.3, 20.9)	6.9 (2.7, 11.5)	20.0 (18.8, 21.4)	14.2 (9.7, 18.9)	19.8 (18.6, 21.1)	10.4 (6.7, 14.2)
	Oceania	Ischemic stroke	10.3 (8.9, 11.8)	6.1 (-6.2, 20.4)	9.9 (8.5, 11.4)	6.0 (-6.8, 19.6)	10.1 (8.7, 11.5)	5.8 (-6.4, 19.2)
		Hemorrhagic stroke	5.9 (5.1, 6.7)	-4.3 (-15.7, 6.9)	7.3 (6.3, 8.3)	-3.1 (-15.1, 9.6)	6.6 (5.8, 7.5)	-3.0 (-14.4, 8.3)
		Total strokes	15.5 (13.8,17.2)	1.7 (-9.0, 13.0)	16.5 (14.6, 18.3)	1.6 (-9.2, 12.8)	16.0 (14.2, 17.6)	1.8 (-8.8, 12.7)
Central Europe,	Central Asia	Ischemic stroke	15.2 (13.6,16.7)	-0.9 (-7.8, 7.1)	18.1 (16.4, 19.8)	-8.2 (-14.1, -2.2)	16.6 (15.1, 18.1)	-5.7 (-11.0, -0.1)
		Hemorrhagic stroke	9.1 (8.3, 10.0)	-5.7 (-11.8, 1.4)	9.7 (8.9, 10.7)	-16.7 (-22.1, -11.3)	9.4 (8.6,10.3)	-12.3 (-17.0, -7.3)
		Total strokes	22.7 (21.1, 24.4)	-2.4 (-8.1, 3.9)	26.1 (24.4, 27.9)	-10.8 (-15.2, -6.2)	24.4 (22.8, 25.9)	-7.7 (-11.7, -3.6)
		Ischemic stroke	22.1 (17.6, 26.8)	-7.3 (-25.3, 13.0)	31.5 (26.3, 36.7)	-7.8 (-22.2, 6.1)	26.8 (23.0, 30.6)	-8.5 (-20.8, 4.3)

**Eastern Europe, and Central Asia**	Eastern Europe	Hemorrhagic stroke	6.4 (5.2, 7.7)	-7.8 (-23.7, 10.6)	7.5 (6.2, 8.8)	-14.3 (-28.8, -0.5)	6.9 (6.0, 7.9)	-12.3 (-24.3, 0.1)
		Total strokes	26.8 (22.0, 31.6)	-6.9 (-22.9, 11.0)	36.5 (31.2, 41.9)	-8.7 (-21.5, 3.7)	31.6 (27.6, 35.6)	-8.8 (-19.7, 2.7)
	Central Europe	Ischemic stroke	24.5 (22.6, 26.3)	20.8 (14.6, 27.9)	28.5 (26.4, 30.4)	9.9 (4.1, 16.1)	26.5 (24.6, 28.2)	14.9 (9.6, 20.5)
		Hemorrhagic stroke	7.6 (7.1, 8.1)	-2.4 (-6.7, 2.2)	7.5 (7.0, 7.9)	-12.7 (-16.5, -8.9)	7.5 (7.0, 8.0)	-7.9 (-11.2, -4.6)
		Total strokes	29.8 (28.0, 31.5)	13.9 (9.2, 18.9)	33.7 (31.8, 35.5)	4.2 (-0.2, 8.7)	31.7 (30.0, 33.3)	8.7 (4.8, 12.8)

Table S4. Lifetime risk of stroke in 2016 and percent change in lifetime risk of stroke from 1990 to 2016, by country, region, and sex						
**Cause**	**Location**	**Sex**	**Mean lifetime risk (%)**	**95% UI Lower**	**95% UI Upper**	**Mean percent change 1990-2016 (%)**
Stroke	Global	males	24.7	23.3	26.0	3.3
Hemorrhagic stroke	Global	males	8.2	7.5	8.8	0.6
Ischemic stroke	Global	males	18.2	16.7	19.7	3.2
Stroke	Global	females	25.1	23.7	26.5	0.8
Hemorrhagic stroke	Global	females	8.3	7.6	9.0	0.1
Ischemic stroke	Global	females	18.5	17.0	20.0	1.0
Stroke	Global	both sexes combined	24.9	23.5	26.2	2.0
Hemorrhagic stroke	Global	both sexes combined	8.2	7.5	8.9	0.3
Ischemic stroke	Global	both sexes combined	18.3	16.9	19.8	2.1
Stroke	Southeast Asia, East Asia, and Oceania	males	36.2	34.5	37.9	8.8
Hemorrhagic stroke	Southeast Asia, East Asia, and Oceania	males	13.3	12.4	14.4	1.3
Ischemic stroke	Southeast Asia, East Asia, and Oceania	males	26.6	24.6	28.5	9.2
Stroke	Southeast Asia, East Asia, and Oceania	females	32.1	30.5	33.8	5.1
Hemorrhagic stroke	Southeast Asia, East Asia, and Oceania	females	13.8	12.7	14.8	1.2
Ischemic stroke	Southeast Asia, East Asia, and Oceania	females	21.5	19.7	23.2	5.0
Stroke	Southeast Asia, East Asia, and Oceania	both sexes combined	34.4	32.8	36.1	7.2
Hemorrhagic stroke	Southeast Asia, East Asia, and Oceania	both sexes combined	13.5	12.5	14.5	1.2
Ischemic stroke	Southeast Asia, East Asia, and Oceania	both sexes combined	24.4	22.5	26.2	7.5
Stroke	East Asia	males	40.6	38.7	42.3	10.7
Hemorrhagic stroke	East Asia	males	14.9	13.9	16.1	1.7
Ischemic stroke	East Asia	males	30.5	28.4	32.6	11.3
Stroke	East Asia	females	36.3	34.5	38.1	6.2
Hemorrhagic stroke	East Asia	females	15.8	14.6	17.0	1.5
Ischemic stroke	East Asia	females	24.6	22.6	26.4	6.2
Stroke	East Asia	both sexes combined	38.8	37.0	40.6	8.9
Hemorrhagic stroke	East Asia	both sexes combined	15.3	14.2	16.4	1.6
Ischemic stroke	East Asia	both sexes combined	28.0	26.0	30.0	9.3
Stroke	China	males	41.1	39.2	42.9	11.1
Hemorrhagic stroke	China	males	15.1	14.1	16.3	1.9
Ischemic stroke	China	males	30.9	28.8	33.1	11.7
Stroke	China	females	36.7	35.0	38.6	6.6
Hemorrhagic stroke	China	females	16.0	14.8	17.2	1.6
Ischemic stroke	China	females	25.0	23.0	26.9	6.5
Stroke	China	both sexes combined	39.3	37.5	41.1	9.3
Hemorrhagic stroke	China	both sexes combined	15.5	14.4	16.6	1.7
Ischemic stroke	China	both sexes combined	28.5	26.4	30.5	9.7
Stroke	North Korea	males	28.0	26.2	30.2	1.0
Hemorrhagic stroke	North Korea	males	12.2	11.0	13.6	0.6
Ischemic stroke	North Korea	males	17.5	15.8	19.5	0.2
Stroke	North Korea	females	27.8	25.8	29.9	1.4
Hemorrhagic stroke	North Korea	females	14.0	12.6	15.6	0.7
Ischemic stroke	North Korea	females	15.7	13.9	17.4	0.6
Stroke	North Korea	both sexes combined	27.8	26.0	29.6	1.3
Hemorrhagic stroke	North Korea	both sexes combined	13.3	12.1	14.6	0.6
Ischemic stroke	North Korea	both sexes combined	16.3	14.6	17.9	0.6
Stroke	Taiwan	males	20.1	18.2	22.2	-2.9
Hemorrhagic stroke	Taiwan	males	6.8	6.0	7.7	-1.6
Ischemic stroke	Taiwan	males	14.5	12.8	16.5	-1.5
Stroke	Taiwan	females	20.7	18.9	22.8	-4.5
Hemorrhagic stroke	Taiwan	females	7.9	6.9	9.1	-2.7
Ischemic stroke	Taiwan	females	14.2	12.5	16.0	-2.1
Stroke	Taiwan	both sexes combined	20.3	18.8	22.1	-3.7
Hemorrhagic stroke	Taiwan	both sexes combined	7.3	6.5	8.1	-2.1
Ischemic stroke	Taiwan	both sexes combined	14.3	12.9	16.0	-1.8
Stroke	Southeast Asia	males	19.6	18.3	20.9	1.3
Hemorrhagic stroke	Southeast Asia	males	7.9	7.2	8.7	-0.3
Ischemic stroke	Southeast Asia	males	12.4	11.1	13.7	1.6
Stroke	Southeast Asia	females	20.0	18.8	21.4	2.5
Hemorrhagic stroke	Southeast Asia	females	8.2	7.5	8.9	0.6
Ischemic stroke	Southeast Asia	females	12.8	11.6	14.1	2.1
Stroke	Southeast Asia	both sexes combined	19.8	18.6	21.1	1.9
Hemorrhagic stroke	Southeast Asia	both sexes combined	8.1	7.4	8.8	0.2
Ischemic stroke	Southeast Asia	both sexes combined	12.6	11.4	13.8	1.8
Stroke	Cambodia	males	16.2	14.8	17.7	2.4
Hemorrhagic stroke	Cambodia	males	6.6	5.9	7.4	0.2
Ischemic stroke	Cambodia	males	10.1	8.8	11.5	2.3
Stroke	Cambodia	females	21.0	19.5	22.7	3.3
Hemorrhagic stroke	Cambodia	females	9.7	8.8	10.7	0.7
Ischemic stroke	Cambodia	females	12.0	10.6	13.4	2.8
Stroke	Cambodia	both sexes combined	18.7	17.3	20.1	2.6
Hemorrhagic stroke	Cambodia	both sexes combined	8.3	7.5	9.1	0.3
Ischemic stroke	Cambodia	both sexes combined	11.1	9.9	12.4	2.5
Stroke	Indonesia	males	22.7	21.0	24.4	3.7
Hemorrhagic stroke	Indonesia	males	9.2	8.2	10.1	0.7
Ischemic stroke	Indonesia	males	14.5	12.9	16.1	3.1
Stroke	Indonesia	females	21.3	19.9	22.8	6.3
Hemorrhagic stroke	Indonesia	females	8.7	7.8	9.4	2.8
Ischemic stroke	Indonesia	females	13.8	12.5	15.2	4.0
Stroke	Indonesia	both sexes combined	22.0	20.6	23.4	5.0

Hemorrhagic stroke	Indonesia	both sexes combined	8.9	8.1	9.8	1.8
Ischemic stroke	Indonesia	both sexes combined	14.1	12.8	15.5	3.5
Stroke	Laos	males	20.0	18.4	21.6	3.9
Hemorrhagic stroke	Laos	males	8.1	7.3	9.1	0.9
Ischemic stroke	Laos	males	12.6	11.2	14.0	3.1
Stroke	Laos	females	20.7	19.0	22.5	3.3
Hemorrhagic stroke	Laos	females	8.8	7.8	9.8	0.5
Ischemic stroke	Laos	females	12.8	11.3	14.5	3.2
Stroke	Laos	both sexes combined	20.4	18.9	21.8	3.5
Hemorrhagic stroke	Laos	both sexes combined	8.5	7.7	9.3	0.7
Ischemic stroke	Laos	both sexes combined	12.7	11.4	14.1	3.1
Stroke	Malaysia	males	17.9	16.5	19.3	-0.4
Hemorrhagic stroke	Malaysia	males	7.1	6.4	7.8	-0.8
Ischemic stroke	Malaysia	males	11.7	10.3	13.1	0.6
Stroke	Malaysia	females	20.8	19.3	22.3	-1.5
Hemorrhagic stroke	Malaysia	females	8.8	7.9	9.7	-1.7
Ischemic stroke	Malaysia	females	13.3	11.9	14.7	0.4
Stroke	Malaysia	both sexes combined	19.2	17.9	20.5	-0.9
Hemorrhagic stroke	Malaysia	both sexes combined	7.8	7.1	8.5	-1.3
Ischemic stroke	Malaysia	both sexes combined	12.5	11.1	13.7	0.5
Stroke	Maldives	males	18.2	15.8	20.8	1.6
Hemorrhagic stroke	Maldives	males	6.6	5.6	7.7	-0.1
Ischemic stroke	Maldives	males	12.5	10.5	14.6	1.9
Stroke	Maldives	females	17.7	15.2	20.1	2.0
Hemorrhagic stroke	Maldives	females	6.6	5.4	7.8	0.0
Ischemic stroke	Maldives	females	12.0	10.1	14.0	2.3
Stroke	Maldives	both sexes combined	17.9	16.1	19.9	1.8
Hemorrhagic stroke	Maldives	both sexes combined	6.5	5.7	7.4	-0.1
Ischemic stroke	Maldives	both sexes combined	12.3	10.6	13.9	2.1
Stroke	Myanmar	males	18.9	17.2	20.5	2.5
Hemorrhagic stroke	Myanmar	males	7.7	6.9	8.6	0.2
Ischemic stroke	Myanmar	males	11.9	10.4	13.5	2.4
Stroke	Myanmar	females	18.4	16.9	19.9	1.8
Hemorrhagic stroke	Myanmar	females	7.2	6.4	8.0	-0.4
Ischemic stroke	Myanmar	females	12.0	10.6	13.5	2.4
Stroke	Myanmar	both sexes combined	18.6	17.1	20.2	2.1
Hemorrhagic stroke	Myanmar	both sexes combined	7.4	6.7	8.2	-0.1
Ischemic stroke	Myanmar	both sexes combined	12.0	10.5	13.5	2.4
Stroke	Philippines	males	16.4	14.5	18.3	2.0
Hemorrhagic stroke	Philippines	males	7.4	6.4	8.5	0.6
Ischemic stroke	Philippines	males	9.6	8.2	11.2	1.4
Stroke	Philippines	females	19.1	17.2	21.3	2.5
Hemorrhagic stroke	Philippines	females	8.5	7.4	9.7	1.3
Ischemic stroke	Philippines	females	11.5	9.9	13.2	1.4
Stroke	Philippines	both sexes combined	17.6	16.1	19.3	2.2
Hemorrhagic stroke	Philippines	both sexes combined	7.9	7.0	8.9	0.9
Ischemic stroke	Philippines	both sexes combined	10.5	9.2	11.9	1.4
Stroke	Sri Lanka	males	17.8	15.2	20.6	0.9
Hemorrhagic stroke	Sri Lanka	males	5.5	4.6	6.5	0.4
Ischemic stroke	Sri Lanka	males	13.1	10.8	15.5	0.6
Stroke	Sri Lanka	females	20.6	17.9	23.4	0.3
Hemorrhagic stroke	Sri Lanka	females	6.8	5.6	8.0	0.2
Ischemic stroke	Sri Lanka	females	14.9	12.6	17.3	0.2
Stroke	Sri Lanka	both sexes combined	19.2	17.1	21.2	0.7
Hemorrhagic stroke	Sri Lanka	both sexes combined	6.1	5.3	7.0	0.4
Ischemic stroke	Sri Lanka	both sexes combined	14.0	12.1	15.9	0.4
Stroke	Thailand	males	16.2	14.8	17.6	-0.5
Hemorrhagic stroke	Thailand	males	6.3	5.6	7.0	-1.6
Ischemic stroke	Thailand	males	10.6	9.4	11.9	1.1
Stroke	Thailand	females	18.5	17.1	20.1	0.4
Hemorrhagic stroke	Thailand	females	7.5	6.7	8.4	-1.0
Ischemic stroke	Thailand	females	12.1	10.7	13.5	1.6
Stroke	Thailand	both sexes combined	17.4	16.1	18.7	0.0
Hemorrhagic stroke	Thailand	both sexes combined	6.9	6.2	7.6	-1.3
Ischemic stroke	Thailand	both sexes combined	11.3	10.1	12.5	1.3
Stroke	Timor-Leste	males	20.4	17.7	23.3	3.3
Hemorrhagic stroke	Timor-Leste	males	8.3	7.0	9.7	0.4
Ischemic stroke	Timor-Leste	males	13.0	11.0	15.3	3.1
Stroke	Timor-Leste	females	20.9	17.7	24.1	3.9
Hemorrhagic stroke	Timor-Leste	females	9.2	7.5	10.9	0.9
Ischemic stroke	Timor-Leste	females	12.8	10.5	15.2	3.6
Stroke	Timor-Leste	both sexes combined	20.7	18.3	23.1	3.5
Hemorrhagic stroke	Timor-Leste	both sexes combined	8.8	7.6	10.1	0.6
Ischemic stroke	Timor-Leste	both sexes combined	12.9	11.1	14.8	3.3
Stroke	Vietnam	males	20.5	18.6	22.5	-1.5
Hemorrhagic stroke	Vietnam	males	8.5	7.6	9.6	-1.9
Ischemic stroke	Vietnam	males	12.6	11.0	14.2	0.2
Stroke	Vietnam	females	20.9	19.3	22.6	-0.9
Hemorrhagic stroke	Vietnam	females	8.8	7.9	9.8	-1.4
Ischemic stroke	Vietnam	females	13.0	11.5	14.5	0.4
Stroke	Vietnam	both sexes combined	20.6	19.2	22.1	-1.2

Hemorrhagic stroke	Vietnam	both sexes combined	8.7	7.9	9.6	-1.6
Ischemic stroke	Vietnam	both sexes combined	12.7	11.4	14.0	0.3
Stroke	Oceania	males	15.5	13.8	17.2	0.2
Hemorrhagic stroke	Oceania	males	5.9	5.1	6.7	-0.3
Ischemic stroke	Oceania	males	10.3	8.9	11.8	0.6
Stroke	Oceania	females	16.5	14.6	18.3	0.2
Hemorrhagic stroke	Oceania	females	7.3	6.3	8.3	-0.2
Ischemic stroke	Oceania	females	9.9	8.5	11.4	0.5
Stroke	Oceania	both sexes combined	16.0	14.2	17.6	0.3
Hemorrhagic stroke	Oceania	both sexes combined	6.6	5.8	7.5	-0.2
Ischemic stroke	Oceania	both sexes combined	10.1	8.7	11.5	0.5
Stroke	Fiji	males	14.5	11.4	17.9	-0.2
Hemorrhagic stroke	Fiji	males	4.9	3.8	6.3	-0.3
Ischemic stroke	Fiji	males	10.2	7.8	13.0	0.2
Stroke	Fiji	females	16.4	13.0	20.0	-1.0
Hemorrhagic stroke	Fiji	females	6.8	5.2	8.7	-1.0
Ischemic stroke	Fiji	females	10.4	8.1	12.8	0.0
Stroke	Fiji	both sexes combined	15.3	12.9	17.9	-0.5
Hemorrhagic stroke	Fiji	both sexes combined	5.8	4.8	7.0	-0.5
Ischemic stroke	Fiji	both sexes combined	10.2	8.5	12.2	0.0
Stroke	Kiribati	males	17.9	15.6	20.1	-0.6
Hemorrhagic stroke	Kiribati	males	6.7	5.7	7.8	-0.6
Ischemic stroke	Kiribati	males	11.6	9.8	13.5	-0.2
Stroke	Kiribati	females	16.4	14.6	18.6	0.6
Hemorrhagic stroke	Kiribati	females	6.5	5.6	7.6	0.0
Ischemic stroke	Kiribati	females	10.6	9.1	12.3	0.8
Stroke	Kiribati	both sexes combined	17.1	15.5	18.8	0.0
Hemorrhagic stroke	Kiribati	both sexes combined	6.6	5.8	7.5	-0.3
Ischemic stroke	Kiribati	both sexes combined	11.1	9.7	12.6	0.3
Stroke	Marshall Islands	males	15.0	12.9	17.2	-1.7
Hemorrhagic stroke	Marshall Islands	males	5.6	4.7	6.5	-1.1
Ischemic stroke	Marshall Islands	males	9.9	8.3	11.8	-0.6
Stroke	Marshall Islands	females	15.2	12.9	17.7	-1.8
Hemorrhagic stroke	Marshall Islands	females	6.4	5.3	7.6	-1.2
Ischemic stroke	Marshall Islands	females	9.5	7.8	11.3	-0.7
Stroke	Marshall Islands	both sexes combined	15.1	13.3	16.9	-1.7
Hemorrhagic stroke	Marshall Islands	both sexes combined	6.0	5.2	6.9	-1.1
Ischemic stroke	Marshall Islands	both sexes combined	9.7	8.3	11.2	-0.7
Stroke	Federated States of Micronesia	males	16.8	14.1	19.5	-1.3
Hemorrhagic stroke	Federated States of Micronesia	males	6.5	5.4	7.7	-0.9
Ischemic stroke	Federated States of Micronesia	males	10.9	8.8	13.0	-0.4
Stroke	Federated States of Micronesia	females	18.1	14.9	21.2	-1.2
Hemorrhagic stroke	Federated States of Micronesia	females	7.9	6.5	9.6	-1.1
Ischemic stroke	Federated States of Micronesia	females	10.9	8.7	13.0	-0.2
Stroke	Federated States of Micronesia	both sexes combined	17.4	14.6	20.2	-1.2
Hemorrhagic stroke	Federated States of Micronesia	both sexes combined	7.2	6.0	8.6	-0.9
Ischemic stroke	Federated States of Micronesia	both sexes combined	10.9	8.8	13.0	-0.3
Stroke	Papua New Guinea	males	15.4	13.3	17.7	0.7
Hemorrhagic stroke	Papua New Guinea	males	5.9	5.0	6.9	-0.1
Ischemic stroke	Papua New Guinea	males	10.0	8.4	11.8	0.9
Stroke	Papua New Guinea	females	16.4	14.1	18.7	0.6
Hemorrhagic stroke	Papua New Guinea	females	7.4	6.2	8.6	-0.1
Ischemic stroke	Papua New Guinea	females	9.5	7.9	11.2	0.8
Stroke	Papua New Guinea	both sexes combined	15.9	13.8	18.0	0.7
Hemorrhagic stroke	Papua New Guinea	both sexes combined	6.7	5.7	7.8	-0.1
Ischemic stroke	Papua New Guinea	both sexes combined	9.7	8.1	11.4	0.8
Stroke	Samoa	males	20.0	17.8	22.5	-0.8
Hemorrhagic stroke	Samoa	males	7.8	6.8	9.0	-0.9
Ischemic stroke	Samoa	males	13.2	11.4	15.2	0.2
Stroke	Samoa	females	20.8	18.5	23.2	-0.4
Hemorrhagic stroke	Samoa	females	9.3	8.0	10.6	-0.4
Ischemic stroke	Samoa	females	12.8	11.0	14.7	0.1
Stroke	Samoa	both sexes combined	20.4	18.6	22.4	-0.7
Hemorrhagic stroke	Samoa	both sexes combined	8.6	7.6	9.6	-0.6
Ischemic stroke	Samoa	both sexes combined	12.9	11.4	14.6	0.0
Stroke	Solomon Islands	males	17.0	14.6	19.3	0.2
Hemorrhagic stroke	Solomon Islands	males	6.7	5.6	7.7	-0.3
Ischemic stroke	Solomon Islands	males	10.9	9.1	12.8	0.6
Stroke	Solomon Islands	females	17.7	15.1	20.3	-0.1
Hemorrhagic stroke	Solomon Islands	females	8.0	6.6	9.3	-0.3
Ischemic stroke	Solomon Islands	females	10.2	8.4	12.0	0.2
Stroke	Solomon Islands	both sexes combined	17.3	14.9	19.7	0.1
Hemorrhagic stroke	Solomon Islands	both sexes combined	7.3	6.2	8.4	-0.2
Ischemic stroke	Solomon Islands	both sexes combined	10.6	8.8	12.4	0.3
Stroke	Tonga	males	15.5	13.7	17.5	0.7
Hemorrhagic stroke	Tonga	males	5.4	4.6	6.2	0.2
Ischemic stroke	Tonga	males	10.8	9.3	12.5	0.6
Stroke	Tonga	females	18.0	15.8	20.3	1.0
Hemorrhagic stroke	Tonga	females	7.7	6.4	8.9	0.2
Ischemic stroke	Tonga	females	11.4	9.7	13.2	1.0
Stroke	Tonga	both sexes combined	16.8	15.1	18.5	0.9

Hemorrhagic stroke	Tonga	both sexes combined	6.6	5.7	7.5	0.2
Ischemic stroke	Tonga	both sexes combined	11.1	9.6	12.6	0.8
Stroke	Vanuatu	males	18.2	15.8	20.4	-0.7
Hemorrhagic stroke	Vanuatu	males	7.0	6.0	8.1	-0.5
Ischemic stroke	Vanuatu	males	11.8	10.0	13.8	-0.3
Stroke	Vanuatu	females	19.3	16.4	22.0	-0.2
Hemorrhagic stroke	Vanuatu	females	8.5	7.0	9.9	-0.3
Ischemic stroke	Vanuatu	females	11.6	9.5	13.5	0.1
Stroke	Vanuatu	both sexes combined	18.7	16.3	21.1	-0.5
Hemorrhagic stroke	Vanuatu	both sexes combined	7.7	6.5	8.9	-0.3
Ischemic stroke	Vanuatu	both sexes combined	11.7	9.8	13.6	-0.2
Stroke	Central Europe, Eastern Europe, and Central Asia	males	27.3	24.3	30.2	-0.1
Hemorrhagic stroke	Central Europe, Eastern Europe, and Central Asia	males	7.1	6.2	8.0	-0.4
Ischemic stroke	Central Europe, Eastern Europe, and Central Asia	males	22.1	19.3	24.9	0.3
Stroke	Central Europe, Eastern Europe, and Central Asia	females	34.6	31.1	37.9	-2.3
Hemorrhagic stroke	Central Europe, Eastern Europe, and Central Asia	females	7.7	6.8	8.6	-1.3
Ischemic stroke	Central Europe, Eastern Europe, and Central Asia	females	29.3	25.8	32.6	-1.3
Stroke	Central Europe, Eastern Europe, and Central Asia	both sexes combined	31.0	28.3	33.6	-1.3
Hemorrhagic stroke	Central Europe, Eastern Europe, and Central Asia	both sexes combined	7.4	6.6	8.1	-0.9
Ischemic stroke	Central Europe, Eastern Europe, and Central Asia	both sexes combined	25.7	23.1	28.2	-0.7
Stroke	Central Asia	males	22.7	21.1	24.4	-0.6
Hemorrhagic stroke	Central Asia	males	9.1	8.3	10.0	-0.6
Ischemic stroke	Central Asia	males	15.2	13.6	16.7	-0.1
Stroke	Central Asia	females	26.1	24.4	27.9	-3.1
Hemorrhagic stroke	Central Asia	females	9.7	8.9	10.7	-2.0
Ischemic stroke	Central Asia	females	18.1	16.4	19.8	-1.6
Stroke	Central Asia	both sexes combined	24.4	22.8	25.9	-2.0
Hemorrhagic stroke	Central Asia	both sexes combined	9.4	8.6	10.3	-1.3
Ischemic stroke	Central Asia	both sexes combined	16.6	15.1	18.1	-1.0
Stroke	Armenia	males	20.4	18.5	22.4	-1.9
Hemorrhagic stroke	Armenia	males	6.7	5.9	7.5	-0.7
Ischemic stroke	Armenia	males	15.0	13.2	16.9	-1.4
Stroke	Armenia	females	24.0	21.9	26.3	-3.7
Hemorrhagic stroke	Armenia	females	6.9	6.1	7.7	-1.5
Ischemic stroke	Armenia	females	18.6	16.5	20.8	-2.5
Stroke	Armenia	both sexes combined	22.3	20.4	24.0	-2.9
Hemorrhagic stroke	Armenia	both sexes combined	6.7	6.0	7.5	-1.1
Ischemic stroke	Armenia	both sexes combined	16.9	15.1	18.6	-2.0
Stroke	Azerbaijan	males	22.8	19.7	26.5	2.6
Hemorrhagic stroke	Azerbaijan	males	10.2	8.6	12.0	-0.3
Ischemic stroke	Azerbaijan	males	14.4	12.0	17.3	3.2
Stroke	Azerbaijan	females	26.6	23.5	29.6	1.0
Hemorrhagic stroke	Azerbaijan	females	11.4	9.8	13.1	-1.3
Ischemic stroke	Azerbaijan	females	17.2	14.7	19.8	2.4
Stroke	Azerbaijan	both sexes combined	24.6	22.2	27.1	1.6
Hemorrhagic stroke	Azerbaijan	both sexes combined	10.7	9.4	12.2	-0.9
Ischemic stroke	Azerbaijan	both sexes combined	15.8	13.7	18.0	2.7
Stroke	Georgia	males	27.7	23.9	31.5	0.1
Hemorrhagic stroke	Georgia	males	11.4	9.7	13.4	-1.3
Ischemic stroke	Georgia	males	18.4	15.5	21.5	1.4
Stroke	Georgia	females	31.9	28.9	35.1	-0.7
Hemorrhagic stroke	Georgia	females	12.9	11.2	14.7	-2.6
Ischemic stroke	Georgia	females	21.7	19.3	24.2	1.7
Stroke	Georgia	both sexes combined	29.7	27.3	32.1	-0.5
Hemorrhagic stroke	Georgia	both sexes combined	12.1	10.8	13.5	-2.1
Ischemic stroke	Georgia	both sexes combined	20.0	18.0	21.9	1.5
Stroke	Kazakhstan	males	23.3	19.7	27.0	0.0
Hemorrhagic stroke	Kazakhstan	males	7.6	6.4	8.8	-0.6
Ischemic stroke	Kazakhstan	males	17.3	14.2	20.5	0.5
Stroke	Kazakhstan	females	29.6	26.4	33.1	-2.7
Hemorrhagic stroke	Kazakhstan	females	8.8	7.6	10.0	-1.8
Ischemic stroke	Kazakhstan	females	22.8	19.8	26.2	-1.4
Stroke	Kazakhstan	both sexes combined	26.5	23.7	29.1	-1.6
Hemorrhagic stroke	Kazakhstan	both sexes combined	8.2	7.1	9.2	-1.3
Ischemic stroke	Kazakhstan	both sexes combined	20.1	17.4	22.7	-0.6
Stroke	Kyrgyzstan	males	23.3	21.3	25.5	-2.3
Hemorrhagic stroke	Kyrgyzstan	males	8.2	7.3	9.1	-1.7
Ischemic stroke	Kyrgyzstan	males	16.4	14.4	18.3	-1.1
Stroke	Kyrgyzstan	females	26.3	24.1	28.6	-7.1
Hemorrhagic stroke	Kyrgyzstan	females	9.0	8.0	10.0	-3.5
Ischemic stroke	Kyrgyzstan	females	18.7	16.7	21.0	-4.4
Stroke	Kyrgyzstan	both sexes combined	24.8	22.9	26.6	-4.9
Hemorrhagic stroke	Kyrgyzstan	both sexes combined	8.6	7.8	9.4	-2.7
Ischemic stroke	Kyrgyzstan	both sexes combined	17.5	15.8	19.3	-2.9
Stroke	Mongolia	males	21.5	18.9	24.2	4.8
Hemorrhagic stroke	Mongolia	males	13.3	11.5	15.2	3.4
Ischemic stroke	Mongolia	males	9.6	7.9	11.2	1.9
Stroke	Mongolia	females	25.8	23.2	28.7	5.8
Hemorrhagic stroke	Mongolia	females	14.9	13.1	16.9	4.0
Ischemic stroke	Mongolia	females	12.8	10.9	15.0	2.4
Stroke	Mongolia	both sexes combined	23.5	21.6	25.7	5.3

Hemorrhagic stroke	Mongolia	both sexes combined	14.0	12.6	15.5	3.6
Ischemic stroke	Mongolia	both sexes combined	11.1	9.5	12.6	2.1
Stroke	Tajikistan	males	26.3	23.3	29.0	1.0
Hemorrhagic stroke	Tajikistan	males	10.0	8.7	11.4	-0.8
Ischemic stroke	Tajikistan	males	18.0	15.5	20.5	1.6
Stroke	Tajikistan	females	28.5	25.7	31.2	-0.1
Hemorrhagic stroke	Tajikistan	females	10.8	9.4	12.1	-1.2
Ischemic stroke	Tajikistan	females	19.5	16.9	22.0	0.8
Stroke	Tajikistan	both sexes combined	27.3	24.9	29.5	0.3
Hemorrhagic stroke	Tajikistan	both sexes combined	10.4	9.2	11.5	-1.0
Ischemic stroke	Tajikistan	both sexes combined	18.7	16.5	20.8	1.1
Stroke	Turkmenistan	males	21.6	20.1	23.2	3.2
Hemorrhagic stroke	Turkmenistan	males	13.7	12.5	15.1	1.0
Ischemic stroke	Turkmenistan	males	9.1	8.0	10.3	2.5
Stroke	Turkmenistan	females	23.0	21.4	24.7	1.7
Hemorrhagic stroke	Turkmenistan	females	13.2	12.0	14.5	-0.7
Ischemic stroke	Turkmenistan	females	11.2	9.9	12.7	2.6
Stroke	Turkmenistan	both sexes combined	22.3	20.9	23.8	2.4
Hemorrhagic stroke	Turkmenistan	both sexes combined	13.4	12.3	14.7	0.1
Ischemic stroke	Turkmenistan	both sexes combined	10.2	9.0	11.4	2.5
Stroke	Uzbekistan	males	20.7	18.7	22.8	-2.4
Hemorrhagic stroke	Uzbekistan	males	8.4	7.4	9.4	-0.6
Ischemic stroke	Uzbekistan	males	13.7	12.0	15.6	-2.0
Stroke	Uzbekistan	females	21.8	19.8	23.9	-4.6
Hemorrhagic stroke	Uzbekistan	females	8.5	7.5	9.6	-2.3
Ischemic stroke	Uzbekistan	females	14.7	12.9	16.6	-2.9
Stroke	Uzbekistan	both sexes combined	21.3	19.5	23.1	-3.6
Hemorrhagic stroke	Uzbekistan	both sexes combined	8.4	7.6	9.4	-1.5
Ischemic stroke	Uzbekistan	both sexes combined	14.2	12.6	15.8	-2.5
Stroke	Central Europe	males	29.8	28.0	31.5	3.6
Hemorrhagic stroke	Central Europe	males	7.6	7.1	8.1	-0.2
Ischemic stroke	Central Europe	males	24.5	22.6	26.3	4.2
Stroke	Central Europe	females	33.7	31.8	35.5	1.3
Hemorrhagic stroke	Central Europe	females	7.5	7.0	7.9	-1.1
Ischemic stroke	Central Europe	females	28.5	26.4	30.4	2.5
Stroke	Central Europe	both sexes combined	31.7	30.0	33.3	2.5
Hemorrhagic stroke	Central Europe	both sexes combined	7.5	7.0	8.0	-0.6
Ischemic stroke	Central Europe	both sexes combined	26.5	24.6	28.2	3.4
Stroke	Albania	males	32.0	28.9	35.2	3.3
Hemorrhagic stroke	Albania	males	16.5	14.4	18.7	-0.1
Ischemic stroke	Albania	males	19.2	16.8	21.9	4.1
Stroke	Albania	females	35.0	32.1	37.9	2.3
Hemorrhagic stroke	Albania	females	18.0	16.1	20.1	-1.1
Ischemic stroke	Albania	females	20.7	18.3	23.1	3.8
Stroke	Albania	both sexes combined	33.4	31.0	35.9	2.8
Hemorrhagic stroke	Albania	both sexes combined	17.1	15.4	19.0	-0.6
Ischemic stroke	Albania	both sexes combined	19.9	17.7	22.2	3.9
Stroke	Bosnia and Herzegovina	males	34.2	30.6	37.7	7.7
Hemorrhagic stroke	Bosnia and Herzegovina	males	7.0	6.1	8.1	-1.2
Ischemic stroke	Bosnia and Herzegovina	males	29.7	26.2	33.2	9.6
Stroke	Bosnia and Herzegovina	females	37.5	33.6	41.4	4.8
Hemorrhagic stroke	Bosnia and Herzegovina	females	6.7	5.8	7.5	-2.9
Ischemic stroke	Bosnia and Herzegovina	females	33.1	29.2	37.1	8.0
Stroke	Bosnia and Herzegovina	both sexes combined	35.7	32.7	38.7	6.1
Hemorrhagic stroke	Bosnia and Herzegovina	both sexes combined	6.8	6.0	7.7	-2.1
Ischemic stroke	Bosnia and Herzegovina	both sexes combined	31.3	28.3	34.5	8.7
Stroke	Bulgaria	males	31.8	28.4	35.6	0.5
Hemorrhagic stroke	Bulgaria	males	9.5	8.1	10.9	-2.4
Ischemic stroke	Bulgaria	males	24.9	21.7	28.4	2.8
Stroke	Bulgaria	females	35.2	31.3	39.2	-0.1
Hemorrhagic stroke	Bulgaria	females	9.4	8.1	10.8	-3.1
Ischemic stroke	Bulgaria	females	28.5	24.7	32.3	2.8
Stroke	Bulgaria	both sexes combined	33.4	30.4	36.5	0.2
Hemorrhagic stroke	Bulgaria	both sexes combined	9.4	8.4	10.6	-2.7
Ischemic stroke	Bulgaria	both sexes combined	26.6	23.6	29.6	2.8
Stroke	Croatia	males	32.3	29.3	35.2	4.9
Hemorrhagic stroke	Croatia	males	7.5	6.8	8.3	-1.4
Ischemic stroke	Croatia	males	27.2	24.4	30.0	6.6
Stroke	Croatia	females	33.8	31.1	36.5	1.0
Hemorrhagic stroke	Croatia	females	7.1	6.4	7.8	-2.8
Ischemic stroke	Croatia	females	28.7	26.0	31.5	3.4
Stroke	Croatia	both sexes combined	33.0	30.7	35.1	2.9
Hemorrhagic stroke	Croatia	both sexes combined	7.2	6.7	7.8	-2.1
Ischemic stroke	Croatia	both sexes combined	27.9	25.7	30.0	5.0
Stroke	Czech Republic	males	29.2	26.8	31.5	1.7
Hemorrhagic stroke	Czech Republic	males	7.0	6.3	7.9	-0.2
Ischemic stroke	Czech Republic	males	24.5	22.0	26.9	2.3
Stroke	Czech Republic	females	32.6	30.1	35.5	-1.6
Hemorrhagic stroke	Czech Republic	females	7.0	6.3	7.9	-1.2
Ischemic stroke	Czech Republic	females	28.0	25.3	31.0	-0.3
Stroke	Czech Republic	both sexes combined	30.9	28.7	33.1	0.2

Hemorrhagic stroke	Czech Republic	both sexes combined	7.0	6.3	7.8	-0.7
Ischemic stroke	Czech Republic	both sexes combined	26.3	23.9	28.7	1.1
Stroke	Hungary	males	26.9	24.1	30.0	1.0
Hemorrhagic stroke	Hungary	males	6.0	5.3	6.8	-0.4
Ischemic stroke	Hungary	males	22.8	19.9	25.7	1.6
Stroke	Hungary	females	30.1	27.5	33.1	-2.4
Hemorrhagic stroke	Hungary	females	5.7	5.1	6.4	-1.3
Ischemic stroke	Hungary	females	26.2	23.5	29.2	-1.2
Stroke	Hungary	both sexes combined	28.5	26.2	31.0	-0.6
Hemorrhagic stroke	Hungary	both sexes combined	5.8	5.2	6.5	-0.8
Ischemic stroke	Hungary	both sexes combined	24.5	22.1	27.1	0.3
Stroke	Macedonia	males	34.1	31.4	36.7	0.2
Hemorrhagic stroke	Macedonia	males	10.3	9.2	11.4	-0.4
Ischemic stroke	Macedonia	males	26.6	23.9	29.3	0.5
Stroke	Macedonia	females	36.4	33.4	39.2	-1.2
Hemorrhagic stroke	Macedonia	females	10.0	9.0	11.1	-1.5
Ischemic stroke	Macedonia	females	28.7	25.7	31.7	-0.1
Stroke	Macedonia	both sexes combined	35.2	32.6	37.6	-0.5
Hemorrhagic stroke	Macedonia	both sexes combined	10.1	9.2	11.1	-0.9
Ischemic stroke	Macedonia	both sexes combined	27.6	24.9	30.2	0.2
Stroke	Montenegro	males	32.8	30.0	35.6	0.2
Hemorrhagic stroke	Montenegro	males	16.1	14.2	17.9	-3.5
Ischemic stroke	Montenegro	males	20.4	17.8	22.9	3.4
Stroke	Montenegro	females	39.4	36.6	42.7	-1.2
Hemorrhagic stroke	Montenegro	females	21.3	19.2	23.6	-5.4
Ischemic stroke	Montenegro	females	22.5	19.8	25.4	3.4
Stroke	Montenegro	both sexes combined	36.0	33.7	38.5	-0.8
Hemorrhagic stroke	Montenegro	both sexes combined	18.6	17.0	20.4	-4.7
Ischemic stroke	Montenegro	both sexes combined	21.4	19.0	23.8	3.4
Stroke	Poland	males	26.1	23.6	28.4	7.1
Hemorrhagic stroke	Poland	males	5.7	5.3	6.2	0.7
Ischemic stroke	Poland	males	22.0	19.5	24.4	7.1
Stroke	Poland	females	30.8	28.2	33.2	4.4
Hemorrhagic stroke	Poland	females	5.7	5.3	6.0	0.0
Ischemic stroke	Poland	females	26.9	24.2	29.3	4.9
Stroke	Poland	both sexes combined	28.4	26.3	30.4	5.8
Hemorrhagic stroke	Poland	both sexes combined	5.7	5.4	5.9	0.4
Ischemic stroke	Poland	both sexes combined	24.5	22.2	26.5	6.1
Stroke	Romania	males	33.6	30.6	36.9	2.6
Hemorrhagic stroke	Romania	males	9.7	8.6	10.8	-0.1
Ischemic stroke	Romania	males	26.9	23.8	30.1	2.9
Stroke	Romania	females	38.9	36.0	42.1	1.6
Hemorrhagic stroke	Romania	females	10.2	9.1	11.3	-0.6
Ischemic stroke	Romania	females	31.7	28.7	34.8	2.3
Stroke	Romania	both sexes combined	36.2	33.7	38.7	2.1
Hemorrhagic stroke	Romania	both sexes combined	9.9	8.9	10.9	-0.4
Ischemic stroke	Romania	both sexes combined	29.2	26.6	31.9	2.6
Stroke	Serbia	males	31.6	29.8	33.4	-0.5
Hemorrhagic stroke	Serbia	males	7.3	6.8	7.7	-1.9
Ischemic stroke	Serbia	males	26.6	24.9	28.4	1.1
Stroke	Serbia	females	36.0	34.2	37.7	-1.1
Hemorrhagic stroke	Serbia	females	7.2	6.9	7.5	-2.9
Ischemic stroke	Serbia	females	31.0	29.2	32.6	1.4
Stroke	Serbia	both sexes combined	33.8	32.2	35.2	-0.8
Hemorrhagic stroke	Serbia	both sexes combined	7.2	6.9	7.5	-2.4
Ischemic stroke	Serbia	both sexes combined	28.8	27.2	30.2	1.3
Stroke	Slovakia	males	30.3	27.5	33.1	6.9
Hemorrhagic stroke	Slovakia	males	7.5	6.6	8.5	1.0
Ischemic stroke	Slovakia	males	25.3	22.5	28.1	6.9
Stroke	Slovakia	females	34.2	30.7	38.1	4.1
Hemorrhagic stroke	Slovakia	females	6.7	6.1	7.3	-0.7
Ischemic stroke	Slovakia	females	29.9	26.2	34.0	5.2
Stroke	Slovakia	both sexes combined	32.2	29.5	34.8	5.7
Hemorrhagic stroke	Slovakia	both sexes combined	7.0	6.5	7.7	0.1
Ischemic stroke	Slovakia	both sexes combined	27.6	24.8	30.3	6.2
Stroke	Slovenia	males	26.2	23.9	28.5	-0.1
Hemorrhagic stroke	Slovenia	males	7.3	6.7	7.9	0.6
Ischemic stroke	Slovenia	males	20.8	18.8	23.0	-0.4
Stroke	Slovenia	females	27.1	24.9	29.5	-4.0
Hemorrhagic stroke	Slovenia	females	6.4	5.8	7.1	-0.7
Ischemic stroke	Slovenia	females	22.3	20.1	24.8	-3.6
Stroke	Slovenia	both sexes combined	26.6	24.9	28.5	-2.1
Hemorrhagic stroke	Slovenia	both sexes combined	6.8	6.4	7.4	-0.1
Ischemic stroke	Slovenia	both sexes combined	21.6	19.9	23.5	-2.0
Stroke	Eastern Europe	males	26.8	22.0	31.6	-2.0
Hemorrhagic stroke	Eastern Europe	males	6.4	5.2	7.7	-0.6
Ischemic stroke	Eastern Europe	males	22.1	17.6	26.8	-1.8
Stroke	Eastern Europe	females	36.5	31.2	41.9	-3.5
Hemorrhagic stroke	Eastern Europe	females	7.5	6.2	8.8	-1.3
Ischemic stroke	Eastern Europe	females	31.5	26.3	36.7	-2.7
Stroke	Eastern Europe	both sexes combined	31.6	27.6	35.6	-3.1

Hemorrhagic stroke	Eastern Europe	both sexes combined	6.9	6.0	7.9	-1.0
Ischemic stroke	Eastern Europe	both sexes combined	26.8	23.0	30.6	-2.5
Stroke	Belarus	males	25.1	21.5	28.5	-1.4
Hemorrhagic stroke	Belarus	males	6.2	5.3	7.2	-0.6
Ischemic stroke	Belarus	males	20.4	17.2	23.8	-0.9
Stroke	Belarus	females	33.4	29.7	37.1	-2.0
Hemorrhagic stroke	Belarus	females	6.9	6.0	7.9	-0.9
Ischemic stroke	Belarus	females	28.6	24.8	32.3	-1.3
Stroke	Belarus	both sexes combined	29.3	26.6	32.3	-1.9
Hemorrhagic stroke	Belarus	both sexes combined	6.6	5.8	7.3	-0.8
Ischemic stroke	Belarus	both sexes combined	24.6	21.9	27.6	-1.3
Stroke	Estonia	males	23.5	21.1	26.1	-2.5
Hemorrhagic stroke	Estonia	males	5.1	4.4	5.8	-0.6
Ischemic stroke	Estonia	males	19.7	17.4	22.3	-2.0
Stroke	Estonia	females	28.5	25.9	31.7	-7.6
Hemorrhagic stroke	Estonia	females	5.5	4.8	6.2	-1.5
Ischemic stroke	Estonia	females	24.7	21.9	27.8	-6.7
Stroke	Estonia	both sexes combined	26.1	23.8	28.5	-5.1
Hemorrhagic stroke	Estonia	both sexes combined	5.3	4.7	5.9	-1.1
Ischemic stroke	Estonia	both sexes combined	22.3	19.9	24.7	-4.4
Stroke	Latvia	males	32.0	28.5	36.1	2.0
Hemorrhagic stroke	Latvia	males	7.2	6.2	8.3	-1.1
Ischemic stroke	Latvia	males	27.5	24.1	31.7	3.0
Stroke	Latvia	females	41.7	37.7	45.4	-0.3
Hemorrhagic stroke	Latvia	females	8.0	7.0	9.1	-2.7
Ischemic stroke	Latvia	females	37.1	33.0	40.9	1.8
Stroke	Latvia	both sexes combined	37.0	34.0	40.1	0.7
Hemorrhagic stroke	Latvia	both sexes combined	7.6	6.7	8.6	-2.0
Ischemic stroke	Latvia	both sexes combined	32.4	29.3	35.5	2.3
Stroke	Lithuania	males	28.2	25.8	30.6	3.7
Hemorrhagic stroke	Lithuania	males	6.0	5.2	6.8	0.8
Ischemic stroke	Lithuania	males	24.2	21.8	26.6	3.4
Stroke	Lithuania	females	37.1	34.4	39.9	2.8
Hemorrhagic stroke	Lithuania	females	6.7	5.9	7.6	0.3
Ischemic stroke	Lithuania	females	33.0	30.2	35.8	2.9
Stroke	Lithuania	both sexes combined	32.7	30.3	35.0	3.1
Hemorrhagic stroke	Lithuania	both sexes combined	6.3	5.6	7.1	0.5
Ischemic stroke	Lithuania	both sexes combined	28.7	26.3	31.0	3.1
Stroke	Moldova	males	25.8	23.1	28.7	0.0
Hemorrhagic stroke	Moldova	males	7.4	6.5	8.4	-1.2
Ischemic stroke	Moldova	males	20.0	17.6	22.7	0.9
Stroke	Moldova	females	29.4	26.8	32.1	-1.6
Hemorrhagic stroke	Moldova	females	7.3	6.4	8.2	-1.9
Ischemic stroke	Moldova	females	23.8	21.2	26.6	-0.1
Stroke	Moldova	both sexes combined	27.6	25.3	29.7	-0.9
Hemorrhagic stroke	Moldova	both sexes combined	7.3	6.5	8.1	-1.6
Ischemic stroke	Moldova	both sexes combined	21.9	19.7	24.0	0.3
Stroke	Russia	males	27.6	20.9	34.7	-2.0
Hemorrhagic stroke	Russia	males	6.6	5.0	8.4	-0.4
Ischemic stroke	Russia	males	22.9	16.7	29.7	-1.9
Stroke	Russia	females	38.2	30.5	45.8	-3.3
Hemorrhagic stroke	Russia	females	7.9	6.1	9.8	-1.2
Ischemic stroke	Russia	females	33.1	25.7	40.6	-2.5
Stroke	Russia	both sexes combined	32.8	27.2	38.3	-3.0
Hemorrhagic stroke	Russia	both sexes combined	7.2	5.9	8.6	-0.9
Ischemic stroke	Russia	both sexes combined	27.9	22.6	33.1	-2.6
Stroke	Ukraine	males	25.3	20.6	30.6	-2.9
Hemorrhagic stroke	Ukraine	males	6.0	4.9	7.4	-1.1
Ischemic stroke	Ukraine	males	20.9	16.4	25.6	-2.3
Stroke	Ukraine	females	33.1	27.7	38.4	-5.0
Hemorrhagic stroke	Ukraine	females	6.6	5.4	8.0	-1.6
Ischemic stroke	Ukraine	females	28.5	23.5	33.7	-4.0
Stroke	Ukraine	both sexes combined	29.2	25.4	33.1	-4.3
Hemorrhagic stroke	Ukraine	both sexes combined	6.3	5.3	7.3	-1.4
Ischemic stroke	Ukraine	both sexes combined	24.7	21.0	28.4	-3.5
Stroke	High-income	males	22.0	20.7	23.3	0.2
Hemorrhagic stroke	High-income	males	7.5	6.9	8.0	0.5
Ischemic stroke	High-income	males	16.3	14.9	17.6	-0.2
Stroke	High-income	females	23.7	22.3	25.0	-1.2
Hemorrhagic stroke	High-income	females	7.4	6.8	8.0	-0.3
Ischemic stroke	High-income	females	18.0	16.6	19.4	-1.0
Stroke	High-income	both sexes combined	22.8	21.5	24.1	-0.5
Hemorrhagic stroke	High-income	both sexes combined	7.4	6.8	8.0	0.1
Ischemic stroke	High-income	both sexes combined	17.1	15.7	18.4	-0.5
Stroke	High-income Asia Pacific	males	22.2	20.6	23.8	-2.9
Hemorrhagic stroke	High-income Asia Pacific	males	7.5	6.8	8.2	-0.4
Ischemic stroke	High-income Asia Pacific	males	16.2	14.6	17.8	-2.5
Stroke	High-income Asia Pacific	females	23.5	21.8	25.1	-4.2
Hemorrhagic stroke	High-income Asia Pacific	females	7.6	6.8	8.4	-1.2
Ischemic stroke	High-income Asia Pacific	females	17.4	15.8	19.2	-3.1
Stroke	High-income Asia Pacific	both sexes combined	22.8	21.2	24.3	-3.5

Hemorrhagic stroke	High-income Asia Pacific	both sexes combined	7.5	6.8	8.2	-0.9
Ischemic stroke	High-income Asia Pacific	both sexes combined	16.8	15.2	18.3	-2.8
Stroke	Brunei	males	20.1	18.0	22.7	-2.4
Hemorrhagic stroke	Brunei	males	6.8	5.9	7.8	-1.3
Ischemic stroke	Brunei	males	14.6	12.6	16.8	-1.4
Stroke	Brunei	females	21.0	19.0	23.3	-2.7
Hemorrhagic stroke	Brunei	females	7.2	6.4	8.2	-1.7
Ischemic stroke	Brunei	females	15.0	13.1	17.1	-1.2
Stroke	Brunei	both sexes combined	20.5	18.7	22.4	-2.5
Hemorrhagic stroke	Brunei	both sexes combined	7.0	6.2	7.8	-1.5
Ischemic stroke	Brunei	both sexes combined	14.7	13.0	16.6	-1.3
Stroke	Japan	males	22.5	21.2	23.9	-2.7
Hemorrhagic stroke	Japan	males	7.7	7.0	8.3	-0.2
Ischemic stroke	Japan	males	16.4	14.9	17.8	-2.7
Stroke	Japan	females	23.6	22.0	25.2	-3.4
Hemorrhagic stroke	Japan	females	7.6	6.9	8.4	-0.8
Ischemic stroke	Japan	females	17.5	16.0	19.2	-2.8
Stroke	Japan	both sexes combined	23.0	21.6	24.4	-3.1
Hemorrhagic stroke	Japan	both sexes combined	7.6	6.9	8.3	-0.5
Ischemic stroke	Japan	both sexes combined	16.9	15.5	18.4	-2.8
Stroke	South Korea	males	21.8	18.1	25.8	-3.7
Hemorrhagic stroke	South Korea	males	6.9	5.5	8.4	-1.8
Ischemic stroke	South Korea	males	16.3	13.1	19.7	-2.0
Stroke	South Korea	females	23.7	19.8	27.4	-7.7
Hemorrhagic stroke	South Korea	females	7.7	6.3	9.1	-3.8
Ischemic stroke	South Korea	females	17.5	14.4	20.8	-4.4
Stroke	South Korea	both sexes combined	22.7	19.8	25.9	-5.7
Hemorrhagic stroke	South Korea	both sexes combined	7.3	6.2	8.5	-2.8
Ischemic stroke	South Korea	both sexes combined	16.8	14.3	19.6	-3.2
Stroke	Singapore	males	20.4	18.0	23.0	-1.0
Hemorrhagic stroke	Singapore	males	6.3	5.4	7.2	-0.4
Ischemic stroke	Singapore	males	15.3	13.1	17.7	-0.6
Stroke	Singapore	females	21.1	18.7	23.6	-3.5
Hemorrhagic stroke	Singapore	females	6.5	5.6	7.4	-1.3
Ischemic stroke	Singapore	females	15.8	13.7	18.3	-2.4
Stroke	Singapore	both sexes combined	20.8	18.8	23.1	-2.2
Hemorrhagic stroke	Singapore	both sexes combined	6.4	5.6	7.2	-0.9
Ischemic stroke	Singapore	both sexes combined	15.6	13.8	17.8	-1.5
Stroke	Australasia	males	20.9	19.4	22.4	1.6
Hemorrhagic stroke	Australasia	males	7.5	6.8	8.3	1.0
Ischemic stroke	Australasia	males	15.0	13.5	16.4	0.8
Stroke	Australasia	females	23.0	21.5	24.7	0.3
Hemorrhagic stroke	Australasia	females	8.7	7.9	9.6	0.2
Ischemic stroke	Australasia	females	16.1	14.7	17.8	0.3
Stroke	Australasia	both sexes combined	21.9	20.6	23.4	1.0
Hemorrhagic stroke	Australasia	both sexes combined	8.1	7.4	8.9	0.6
Ischemic stroke	Australasia	both sexes combined	15.6	14.2	17.0	0.6
Stroke	Australia	males	20.0	18.4	21.6	0.5
Hemorrhagic stroke	Australia	males	7.3	6.5	8.2	0.8
Ischemic stroke	Australia	males	14.2	12.6	15.7	-0.2
Stroke	Australia	females	22.1	20.5	24.0	-0.6
Hemorrhagic stroke	Australia	females	8.6	7.7	9.6	0.2
Ischemic stroke	Australia	females	15.2	13.6	17.0	-0.8
Stroke	Australia	both sexes combined	21.0	19.5	22.6	0.0
Hemorrhagic stroke	Australia	both sexes combined	8.0	7.2	8.8	0.5
Ischemic stroke	Australia	both sexes combined	14.7	13.2	16.2	-0.5
Stroke	New Zealand	males	25.4	23.0	27.6	6.9
Hemorrhagic stroke	New Zealand	males	8.7	7.8	9.6	2.2
Ischemic stroke	New Zealand	males	19.2	16.8	21.3	6.0
Stroke	New Zealand	females	27.4	25.2	29.5	5.0
Hemorrhagic stroke	New Zealand	females	9.2	8.3	10.2	0.3
Ischemic stroke	New Zealand	females	20.8	18.5	22.6	5.5
Stroke	New Zealand	both sexes combined	26.4	24.5	28.2	5.9
Hemorrhagic stroke	New Zealand	both sexes combined	8.9	8.1	9.6	1.2
Ischemic stroke	New Zealand	both sexes combined	20.0	17.9	21.7	5.7
Stroke	Western Europe	males	22.2	20.9	23.4	0.9
Hemorrhagic stroke	Western Europe	males	8.1	7.5	8.7	0.7
Ischemic stroke	Western Europe	males	15.9	14.5	17.2	0.5
Stroke	Western Europe	females	23.3	21.9	24.6	-1.0
Hemorrhagic stroke	Western Europe	females	7.8	7.2	8.5	-0.4
Ischemic stroke	Western Europe	females	17.2	15.8	18.5	-0.7
Stroke	Western Europe	both sexes combined	22.7	21.4	23.9	-0.1
Hemorrhagic stroke	Western Europe	both sexes combined	7.9	7.3	8.5	0.1
Ischemic stroke	Western Europe	both sexes combined	16.5	15.2	17.8	-0.1
Stroke	Andorra	males	22.2	19.7	25.3	-0.2
Hemorrhagic stroke	Andorra	males	9.0	7.7	10.5	0.2
Ischemic stroke	Andorra	males	15.2	13.0	17.7	-0.5
Stroke	Andorra	females	24.1	20.7	27.0	-1.6
Hemorrhagic stroke	Andorra	females	8.8	7.4	10.2	-0.6
Ischemic stroke	Andorra	females	17.2	14.5	19.8	-1.2
Stroke	Andorra	both sexes combined	23.0	20.7	25.3	-1.0

Hemorrhagic stroke	Andorra	both sexes combined	8.8	7.7	10.0	-0.2
Ischemic stroke	Andorra	both sexes combined	16.2	14.1	18.2	-0.9
Stroke	Austria	males	24.8	23.0	26.6	-1.2
Hemorrhagic stroke	Austria	males	8.6	7.7	9.5	-1.1
Ischemic stroke	Austria	males	18.5	16.7	20.3	-0.6
Stroke	Austria	females	24.8	23.0	26.8	-4.0
Hemorrhagic stroke	Austria	females	8.1	7.3	9.0	-1.9
Ischemic stroke	Austria	females	18.7	17.0	20.8	-2.9
Stroke	Austria	both sexes combined	24.8	23.0	26.4	-2.7
Hemorrhagic stroke	Austria	both sexes combined	8.3	7.5	9.1	-1.5
Ischemic stroke	Austria	both sexes combined	18.6	16.9	20.4	-1.7
Stroke	Belgium	males	22.7	20.7	24.8	2.2
Hemorrhagic stroke	Belgium	males	8.1	7.4	8.8	0.8
Ischemic stroke	Belgium	males	16.6	14.5	18.7	1.8
Stroke	Belgium	females	23.9	21.7	26.2	-0.6
Hemorrhagic stroke	Belgium	females	7.4	6.9	8.1	-0.9
Ischemic stroke	Belgium	females	18.1	16.0	20.5	0.1
Stroke	Belgium	both sexes combined	23.2	21.4	25.0	0.7
Hemorrhagic stroke	Belgium	both sexes combined	7.7	7.2	8.2	-0.1
Ischemic stroke	Belgium	both sexes combined	17.3	15.4	19.2	0.9
Stroke	Cyprus	males	20.1	18.6	21.6	-0.1
Hemorrhagic stroke	Cyprus	males	9.0	8.0	10.0	0.7
Ischemic stroke	Cyprus	males	12.7	11.3	14.1	-0.6
Stroke	Cyprus	females	20.8	19.3	22.6	-1.2
Hemorrhagic stroke	Cyprus	females	8.7	7.7	9.6	-0.2
Ischemic stroke	Cyprus	females	13.7	12.1	15.2	-1.1
Stroke	Cyprus	both sexes combined	20.4	19.1	21.9	-0.6
Hemorrhagic stroke	Cyprus	both sexes combined	8.8	7.9	9.7	0.2
Ischemic stroke	Cyprus	both sexes combined	13.2	11.8	14.6	-0.8
Stroke	Denmark	males	21.7	19.5	23.8	0.8
Hemorrhagic stroke	Denmark	males	8.2	7.2	9.2	0.5
Ischemic stroke	Denmark	males	15.3	13.3	17.2	0.4
Stroke	Denmark	females	22.1	20.1	24.4	0.0
Hemorrhagic stroke	Denmark	females	7.9	7.0	8.9	-0.2
Ischemic stroke	Denmark	females	15.9	13.9	18.0	0.3
Stroke	Denmark	both sexes combined	21.9	20.1	23.7	0.4
Hemorrhagic stroke	Denmark	both sexes combined	8.0	7.1	8.9	0.1
Ischemic stroke	Denmark	both sexes combined	15.6	13.8	17.4	0.4
Stroke	Finland	males	27.9	25.8	30.1	5.2
Hemorrhagic stroke	Finland	males	8.5	7.6	9.5	2.2
Ischemic stroke	Finland	males	21.9	19.8	24.1	4.1
Stroke	Finland	females	30.8	28.2	33.3	2.1
Hemorrhagic stroke	Finland	females	8.7	7.8	9.7	1.1
Ischemic stroke	Finland	females	24.7	22.1	27.4	1.6
Stroke	Finland	both sexes combined	29.3	27.3	31.2	3.6
Hemorrhagic stroke	Finland	both sexes combined	8.6	7.7	9.4	1.6
Ischemic stroke	Finland	both sexes combined	23.3	21.2	25.5	2.8
Stroke	France	males	19.6	18.1	21.1	0.5
Hemorrhagic stroke	France	males	7.2	6.5	8.1	0.2
Ischemic stroke	France	males	13.8	12.3	15.2	0.4
Stroke	France	females	21.7	20.0	23.5	-0.9
Hemorrhagic stroke	France	females	7.4	6.6	8.1	-0.5
Ischemic stroke	France	females	15.8	14.1	17.6	-0.4
Stroke	France	both sexes combined	20.6	19.2	22.1	-0.2
Hemorrhagic stroke	France	both sexes combined	7.3	6.6	7.9	-0.2
Ischemic stroke	France	both sexes combined	14.8	13.3	16.2	0.0
Stroke	Germany	males	23.4	21.5	25.4	2.7
Hemorrhagic stroke	Germany	males	7.4	6.6	8.3	0.5
Ischemic stroke	Germany	males	17.9	15.9	19.8	2.5
Stroke	Germany	females	24.1	22.0	26.2	0.8
Hemorrhagic stroke	Germany	females	7.1	6.3	7.9	-0.3
Ischemic stroke	Germany	females	18.7	16.7	20.8	1.3
Stroke	Germany	both sexes combined	23.7	21.9	25.5	1.7
Hemorrhagic stroke	Germany	both sexes combined	7.2	6.5	8.0	0.1
Ischemic stroke	Germany	both sexes combined	18.3	16.4	20.1	1.9
Stroke	Greece	males	24.6	22.7	26.7	-2.1
Hemorrhagic stroke	Greece	males	10.0	8.9	11.0	-1.1
Ischemic stroke	Greece	males	16.9	15.0	18.8	-1.3
Stroke	Greece	females	27.3	25.3	29.3	-4.9
Hemorrhagic stroke	Greece	females	10.3	9.2	11.4	-2.4
Ischemic stroke	Greece	females	19.3	17.2	21.4	-3.0
Stroke	Greece	both sexes combined	25.9	24.2	27.7	-3.5
Hemorrhagic stroke	Greece	both sexes combined	10.1	9.2	11.0	-1.8
Ischemic stroke	Greece	both sexes combined	18.1	16.3	19.9	-2.1
Stroke	Iceland	males	23.6	21.7	25.7	1.3
Hemorrhagic stroke	Iceland	males	7.8	7.0	8.7	0.4
Ischemic stroke	Iceland	males	17.7	15.8	19.7	1.1
Stroke	Iceland	females	23.1	21.1	25.2	0.0
Hemorrhagic stroke	Iceland	females	7.2	6.3	8.0	-0.6
Ischemic stroke	Iceland	females	17.6	15.6	19.5	0.5
Stroke	Iceland	both sexes combined	23.4	21.7	25.1	0.7

Hemorrhagic stroke	Iceland	both sexes combined	7.5	6.7	8.2	-0.1
Ischemic stroke	Iceland	both sexes combined	17.6	15.8	19.3	0.9
Stroke	Ireland	males	20.5	18.4	22.7	0.8
Hemorrhagic stroke	Ireland	males	7.7	6.7	8.7	0.8
Ischemic stroke	Ireland	males	14.5	12.5	16.4	0.2
Stroke	Ireland	females	21.5	19.2	23.8	-1.2
Hemorrhagic stroke	Ireland	females	7.7	6.7	8.8	-0.2
Ischemic stroke	Ireland	females	15.3	13.3	17.5	-1.1
Stroke	Ireland	both sexes combined	21.0	19.2	23.0	-0.1
Hemorrhagic stroke	Ireland	both sexes combined	7.7	6.8	8.6	0.3
Ischemic stroke	Ireland	both sexes combined	14.9	13.2	16.7	-0.4
Stroke	Israel	males	22.0	19.4	24.8	-0.7
Hemorrhagic stroke	Israel	males	8.2	6.9	9.6	0.3
Ischemic stroke	Israel	males	15.5	13.2	17.9	-1.0
Stroke	Israel	females	21.2	18.5	23.5	-1.3
Hemorrhagic stroke	Israel	females	7.2	6.2	8.3	-0.4
Ischemic stroke	Israel	females	15.4	13.1	17.5	-1.0
Stroke	Israel	both sexes combined	21.5	19.3	23.6	-1.0
Hemorrhagic stroke	Israel	both sexes combined	7.7	6.8	8.6	-0.1
Ischemic stroke	Israel	both sexes combined	15.4	13.4	17.4	-1.0
Stroke	Italy	males	23.1	21.8	24.3	1.0
Hemorrhagic stroke	Italy	males	9.1	8.5	9.8	2.0
Ischemic stroke	Italy	males	16.1	14.9	17.2	-0.5
Stroke	Italy	females	23.4	22.2	24.7	-0.6
Hemorrhagic stroke	Italy	females	8.1	7.4	8.8	0.2
Ischemic stroke	Italy	females	17.1	15.9	18.3	-0.6
Stroke	Italy	both sexes combined	23.3	22.2	24.2	0.2
Hemorrhagic stroke	Italy	both sexes combined	8.5	8.0	9.1	1.0
Ischemic stroke	Italy	both sexes combined	16.6	15.6	17.5	-0.5
Stroke	Luxembourg	males	23.7	21.8	25.7	-0.2
Hemorrhagic stroke	Luxembourg	males	8.5	7.6	9.6	-0.7
Ischemic stroke	Luxembourg	males	17.2	15.3	18.9	0.4
Stroke	Luxembourg	females	23.0	21.2	24.9	-5.0
Hemorrhagic stroke	Luxembourg	females	8.3	7.4	9.3	-2.2
Ischemic stroke	Luxembourg	females	16.4	14.8	18.2	-3.4
Stroke	Luxembourg	both sexes combined	23.3	21.8	24.9	-2.6
Hemorrhagic stroke	Luxembourg	both sexes combined	8.4	7.6	9.3	-1.5
Ischemic stroke	Luxembourg	both sexes combined	16.8	15.3	18.2	-1.5
Stroke	Malta	males	22.0	19.4	24.6	0.3
Hemorrhagic stroke	Malta	males	9.7	8.4	11.3	1.4
Ischemic stroke	Malta	males	14.2	12.2	16.3	-0.8
Stroke	Malta	females	21.5	19.2	24.1	-1.8
Hemorrhagic stroke	Malta	females	8.5	7.3	9.7	-0.1
Ischemic stroke	Malta	females	14.6	12.5	16.9	-1.8
Stroke	Malta	both sexes combined	21.7	19.7	23.6	-0.8
Hemorrhagic stroke	Malta	both sexes combined	9.0	7.9	10.1	0.6
Ischemic stroke	Malta	both sexes combined	14.4	12.6	16.1	-1.4
Stroke	Netherlands	males	21.0	19.2	22.9	2.3
Hemorrhagic stroke	Netherlands	males	7.7	6.8	8.6	0.8
Ischemic stroke	Netherlands	males	14.9	13.2	16.7	1.7
Stroke	Netherlands	females	22.4	20.4	24.3	0.0
Hemorrhagic stroke	Netherlands	females	7.9	7.1	8.8	-0.2
Ischemic stroke	Netherlands	females	16.1	14.2	18.0	0.2
Stroke	Netherlands	both sexes combined	21.7	20.0	23.4	1.1
Hemorrhagic stroke	Netherlands	both sexes combined	7.8	7.0	8.5	0.3
Ischemic stroke	Netherlands	both sexes combined	15.5	13.9	17.2	1.0
Stroke	Norway	males	25.7	23.5	28.2	2.0
Hemorrhagic stroke	Norway	males	9.5	8.4	10.6	1.0
Ischemic stroke	Norway	males	18.7	16.6	21.0	1.4
Stroke	Norway	females	25.7	23.4	28.3	-1.2
Hemorrhagic stroke	Norway	females	8.7	7.7	9.8	-0.8
Ischemic stroke	Norway	females	19.2	16.9	21.9	-0.6
Stroke	Norway	both sexes combined	25.7	23.8	27.8	0.5
Hemorrhagic stroke	Norway	both sexes combined	9.1	8.2	10.0	0.0
Ischemic stroke	Norway	both sexes combined	18.9	17.0	21.1	0.5
Stroke	Portugal	males	22.9	21.2	24.5	-8.5
Hemorrhagic stroke	Portugal	males	9.0	8.1	10.0	-3.4
Ischemic stroke	Portugal	males	15.6	14.1	17.1	-6.3
Stroke	Portugal	females	24.9	23.1	26.6	-11.2
Hemorrhagic stroke	Portugal	females	8.8	7.8	9.7	-4.7
Ischemic stroke	Portugal	females	17.8	16.1	19.7	-8.0
Stroke	Portugal	both sexes combined	23.8	22.4	25.3	-9.9
Hemorrhagic stroke	Portugal	both sexes combined	8.8	8.0	9.6	-4.0
Ischemic stroke	Portugal	both sexes combined	16.7	15.3	18.2	-7.1
Stroke	Spain	males	21.0	19.5	22.6	-1.4
Hemorrhagic stroke	Spain	males	8.1	7.3	9.0	0.4
Ischemic stroke	Spain	males	14.5	13.0	16.2	-1.9
Stroke	Spain	females	22.6	20.9	24.3	-3.6
Hemorrhagic stroke	Spain	females	8.0	7.2	8.9	-0.4
Ischemic stroke	Spain	females	16.2	14.5	18.0	-3.5
Stroke	Spain	both sexes combined	21.8	20.3	23.3	-2.5

Hemorrhagic stroke	Spain	both sexes combined	8.0	7.3	8.8	0.0
Ischemic stroke	Spain	both sexes combined	15.4	13.8	16.9	-2.6
Stroke	Sweden	males	24.5	22.1	26.8	3.2
Hemorrhagic stroke	Sweden	males	8.5	7.4	9.6	1.3
Ischemic stroke	Sweden	males	18.1	16.0	20.4	2.4
Stroke	Sweden	females	24.5	22.2	26.9	0.8
Hemorrhagic stroke	Sweden	females	8.0	7.0	9.1	0.1
Ischemic stroke	Sweden	females	18.4	16.2	20.8	0.8
Stroke	Sweden	both sexes combined	24.5	22.5	26.6	2.0
Hemorrhagic stroke	Sweden	both sexes combined	8.2	7.3	9.1	0.7
Ischemic stroke	Sweden	both sexes combined	18.3	16.3	20.3	1.6
Stroke	Switzerland	males	25.6	21.5	29.3	3.3
Hemorrhagic stroke	Switzerland	males	9.6	7.8	11.5	1.0
Ischemic stroke	Switzerland	males	18.6	15.1	21.9	2.8
Stroke	Switzerland	females	24.5	21.3	27.5	0.2
Hemorrhagic stroke	Switzerland	females	8.7	7.5	9.8	-0.3
Ischemic stroke	Switzerland	females	17.8	15.2	20.4	0.6
Stroke	Switzerland	both sexes combined	24.9	22.3	27.5	1.7
Hemorrhagic stroke	Switzerland	both sexes combined	9.0	7.9	10.1	0.3
Ischemic stroke	Switzerland	both sexes combined	18.1	15.8	20.5	1.7
Stroke	United Kingdom	males	20.5	19.2	21.8	0.7
Hemorrhagic stroke	United Kingdom	males	7.9	7.1	8.6	0.8
Ischemic stroke	United Kingdom	males	14.2	12.9	15.5	0.1
Stroke	United Kingdom	females	21.9	20.5	23.2	-1.5
Hemorrhagic stroke	United Kingdom	females	8.1	7.4	8.9	-0.2
Ischemic stroke	United Kingdom	females	15.4	13.9	16.8	-1.4
Stroke	United Kingdom	both sexes combined	21.2	19.9	22.4	-0.4
Hemorrhagic stroke	United Kingdom	both sexes combined	8.0	7.3	8.7	0.2
Ischemic stroke	United Kingdom	both sexes combined	14.8	13.4	16.1	-0.6
Stroke	Southern Latin America	males	17.8	16.3	19.3	-3.0
Hemorrhagic stroke	Southern Latin America	males	6.0	5.4	6.7	0.3
Ischemic stroke	Southern Latin America	males	12.7	11.3	14.1	-3.3
Stroke	Southern Latin America	females	20.6	18.9	22.3	-3.5
Hemorrhagic stroke	Southern Latin America	females	6.0	5.4	6.7	-0.1
Ischemic stroke	Southern Latin America	females	15.6	14.0	17.3	-3.4
Stroke	Southern Latin America	both sexes combined	19.2	17.8	20.5	-3.1
Hemorrhagic stroke	Southern Latin America	both sexes combined	6.0	5.4	6.6	0.0
Ischemic stroke	Southern Latin America	both sexes combined	14.2	12.8	15.6	-3.3
Stroke	Argentina	males	16.4	15.0	17.8	-4.2
Hemorrhagic stroke	Argentina	males	5.6	4.9	6.2	-0.1
Ischemic stroke	Argentina	males	11.7	10.4	13.0	-4.2
Stroke	Argentina	females	18.9	17.3	20.5	-4.6
Hemorrhagic stroke	Argentina	females	5.6	5.0	6.1	-0.4
Ischemic stroke	Argentina	females	14.2	12.8	15.9	-4.4
Stroke	Argentina	both sexes combined	17.6	16.3	19.0	-4.3
Hemorrhagic stroke	Argentina	both sexes combined	5.5	5.0	6.1	-0.3
Ischemic stroke	Argentina	both sexes combined	13.0	11.7	14.3	-4.2
Stroke	Chile	males	21.1	17.9	24.4	0.0
Hemorrhagic stroke	Chile	males	7.3	6.0	8.6	1.2
Ischemic stroke	Chile	males	15.2	12.5	18.0	-1.0
Stroke	Chile	females	24.2	20.4	27.8	0.0
Hemorrhagic stroke	Chile	females	7.2	6.0	8.5	0.8
Ischemic stroke	Chile	females	18.5	15.1	21.8	-0.5
Stroke	Chile	both sexes combined	22.6	19.9	25.5	0.0
Hemorrhagic stroke	Chile	both sexes combined	7.2	6.2	8.2	1.0
Ischemic stroke	Chile	both sexes combined	16.9	14.4	19.4	-0.7
Stroke	Uruguay	males	18.9	17.3	20.5	-2.3
Hemorrhagic stroke	Uruguay	males	6.0	5.4	6.7	0.2
Ischemic stroke	Uruguay	males	13.9	12.4	15.5	-2.6
Stroke	Uruguay	females	24.7	22.8	26.8	-3.2
Hemorrhagic stroke	Uruguay	females	7.0	6.3	7.7	-0.2
Ischemic stroke	Uruguay	females	19.2	17.3	21.3	-3.1
Stroke	Uruguay	both sexes combined	21.8	20.3	23.5	-2.6
Hemorrhagic stroke	Uruguay	both sexes combined	6.5	5.9	7.1	0.0
Ischemic stroke	Uruguay	both sexes combined	16.7	15.1	18.4	-2.7
Stroke	High-income North America	males	22.4	21.1	23.7	1.1
Hemorrhagic stroke	High-income North America	males	6.8	6.2	7.3	1.1
Ischemic stroke	High-income North America	males	17.4	15.9	18.8	0.2
Stroke	High-income North America	females	25.0	23.6	26.4	0.1
Hemorrhagic stroke	High-income North America	females	6.7	6.2	7.3	0.6
Ischemic stroke	High-income North America	females	20.1	18.5	21.5	-0.3
Stroke	High-income North America	both sexes combined	23.8	22.3	25.0	0.6
Hemorrhagic stroke	High-income North America	both sexes combined	6.7	6.2	7.3	0.8
Ischemic stroke	High-income North America	both sexes combined	18.8	17.3	20.1	0.0
Stroke	Canada	males	23.2	21.5	25.0	1.6
Hemorrhagic stroke	Canada	males	6.8	6.1	7.6	1.1
Ischemic stroke	Canada	males	18.2	16.3	20.1	0.8
Stroke	Canada	females	25.5	23.7	27.4	-0.2
Hemorrhagic stroke	Canada	females	6.9	6.2	7.6	0.6
Ischemic stroke	Canada	females	20.4	18.5	22.6	-0.7
Stroke	Canada	both sexes combined	24.4	22.7	26.1	0.8

Hemorrhagic stroke	Canada	both sexes combined	6.8	6.1	7.5	0.9
Ischemic stroke	Canada	both sexes combined	19.3	17.5	21.1	0.1
Stroke	United States	males	22.3	21.0	23.6	1.0
Hemorrhagic stroke	United States	males	6.8	6.2	7.3	1.1
Ischemic stroke	United States	males	17.3	15.9	18.7	0.1
Stroke	United States	females	25.0	23.5	26.3	0.1
Hemorrhagic stroke	United States	females	6.7	6.2	7.3	0.6
Ischemic stroke	United States	females	20.0	18.5	21.5	-0.3
Stroke	United States	both sexes combined	23.7	22.3	24.9	0.6
Hemorrhagic stroke	United States	both sexes combined	6.7	6.2	7.2	0.8
Ischemic stroke	United States	both sexes combined	18.7	17.2	20.1	-0.1
Stroke	Latin America and Caribbean	males	16.6	15.5	17.7	-0.8
Hemorrhagic stroke	Latin America and Caribbean	males	4.9	4.4	5.3	-0.2
Ischemic stroke	Latin America and Caribbean	males	12.4	11.3	13.6	-0.6
Stroke	Latin America and Caribbean	females	18.3	17.0	19.6	-1.6
Hemorrhagic stroke	Latin America and Caribbean	females	5.9	5.3	6.4	-0.3
Ischemic stroke	Latin America and Caribbean	females	13.3	12.0	14.6	-1.3
Stroke	Latin America and Caribbean	both sexes combined	17.4	16.3	18.6	-1.2
Hemorrhagic stroke	Latin America and Caribbean	both sexes combined	5.4	4.9	5.9	-0.2
Ischemic stroke	Latin America and Caribbean	both sexes combined	12.9	11.6	14.1	-0.9
Stroke	Caribbean	males	18.0	16.6	19.3	0.2
Hemorrhagic stroke	Caribbean	males	6.0	5.5	6.6	0.3
Ischemic stroke	Caribbean	males	12.8	11.5	14.1	0.0
Stroke	Caribbean	females	20.8	19.3	22.3	-0.1
Hemorrhagic stroke	Caribbean	females	7.1	6.4	7.8	0.1
Ischemic stroke	Caribbean	females	14.8	13.2	16.3	-0.1
Stroke	Caribbean	both sexes combined	19.4	18.0	20.7	0.1
Hemorrhagic stroke	Caribbean	both sexes combined	6.5	6.0	7.2	0.2
Ischemic stroke	Caribbean	both sexes combined	13.8	12.4	15.1	0.0
Stroke	Antigua and Barbuda	males	17.9	16.1	19.8	-2.7
Hemorrhagic stroke	Antigua and Barbuda	males	6.8	6.0	7.8	-0.6
Ischemic stroke	Antigua and Barbuda	males	12.0	10.4	13.7	-2.1
Stroke	Antigua and Barbuda	females	21.5	19.4	23.7	-2.8
Hemorrhagic stroke	Antigua and Barbuda	females	7.8	6.8	8.8	-0.5
Ischemic stroke	Antigua and Barbuda	females	15.1	13.2	17.1	-2.3
Stroke	Antigua and Barbuda	both sexes combined	19.8	18.3	21.6	-2.7
Hemorrhagic stroke	Antigua and Barbuda	both sexes combined	7.3	6.6	8.2	-0.5
Ischemic stroke	Antigua and Barbuda	both sexes combined	13.6	12.1	15.2	-2.2
Stroke	The Bahamas	males	16.2	14.6	17.9	-0.3
Hemorrhagic stroke	The Bahamas	males	5.9	5.2	6.6	0.4
Ischemic stroke	The Bahamas	males	11.1	9.6	12.6	-0.7
Stroke	The Bahamas	females	19.7	17.7	22.0	-1.2
Hemorrhagic stroke	The Bahamas	females	7.0	6.1	8.1	0.2
Ischemic stroke	The Bahamas	females	13.8	12.0	15.7	-1.5
Stroke	The Bahamas	both sexes combined	18.1	16.4	19.7	-0.6
Hemorrhagic stroke	The Bahamas	both sexes combined	6.5	5.8	7.3	0.3
Ischemic stroke	The Bahamas	both sexes combined	12.5	11.1	14.0	-1.0
Stroke	Barbados	males	18.3	16.7	20.1	-2.0
Hemorrhagic stroke	Barbados	males	6.6	5.9	7.4	0.1
Ischemic stroke	Barbados	males	12.7	11.2	14.3	-2.2
Stroke	Barbados	females	21.8	19.8	23.8	-2.3
Hemorrhagic stroke	Barbados	females	7.8	6.9	8.7	0.2
Ischemic stroke	Barbados	females	15.4	13.6	17.3	-2.6
Stroke	Barbados	both sexes combined	20.2	18.6	21.9	-2.2
Hemorrhagic stroke	Barbados	both sexes combined	7.2	6.6	8.0	0.2
Ischemic stroke	Barbados	both sexes combined	14.1	12.6	15.6	-2.4
Stroke	Belize	males	14.7	13.0	16.7	-2.9
Hemorrhagic stroke	Belize	males	5.2	4.5	6.1	-0.7
Ischemic stroke	Belize	males	10.1	8.7	11.7	-2.4
Stroke	Belize	females	17.7	15.8	19.8	-1.5
Hemorrhagic stroke	Belize	females	6.2	5.4	7.2	-0.2
Ischemic stroke	Belize	females	12.4	10.9	14.2	-1.3
Stroke	Belize	both sexes combined	16.0	14.6	17.6	-2.3
Hemorrhagic stroke	Belize	both sexes combined	5.7	5.0	6.4	-0.5
Ischemic stroke	Belize	both sexes combined	11.1	9.9	12.5	-1.9
Stroke	Cuba	males	18.7	17.2	20.3	0.8
Hemorrhagic stroke	Cuba	males	5.7	5.1	6.3	0.2
Ischemic stroke	Cuba	males	13.9	12.4	15.5	0.7
Stroke	Cuba	females	21.7	19.9	23.6	0.5
Hemorrhagic stroke	Cuba	females	6.5	5.8	7.2	-0.1
Ischemic stroke	Cuba	females	16.3	14.6	18.2	0.6
Stroke	Cuba	both sexes combined	20.1	18.7	21.7	0.7
Hemorrhagic stroke	Cuba	both sexes combined	6.1	5.5	6.7	0.0
Ischemic stroke	Cuba	both sexes combined	15.1	13.6	16.5	0.7
Stroke	Dominica	males	15.1	13.2	17.0	-0.6
Hemorrhagic stroke	Dominica	males	5.5	4.7	6.3	0.3
Ischemic stroke	Dominica	males	10.3	8.8	11.9	-0.9
Stroke	Dominica	females	19.1	16.9	21.1	0.1
Hemorrhagic stroke	Dominica	females	6.7	5.8	7.6	0.7
Ischemic stroke	Dominica	females	13.4	11.5	15.2	-0.5
Stroke	Dominica	both sexes combined	17.0	15.4	18.6	-0.4

Hemorrhagic stroke	Dominica	both sexes combined	6.0	5.3	6.7	0.5
Ischemic stroke	Dominica	both sexes combined	11.8	10.3	13.3	-0.8
Stroke	Dominican Republic	males	19.6	17.8	21.6	-0.6
Hemorrhagic stroke	Dominican Republic	males	7.0	6.1	7.9	0.3
Ischemic stroke	Dominican Republic	males	13.6	12.0	15.6	-0.8
Stroke	Dominican Republic	females	22.2	19.8	24.9	-0.4
Hemorrhagic stroke	Dominican Republic	females	7.7	6.6	8.9	0.1
Ischemic stroke	Dominican Republic	females	15.8	13.4	18.1	-0.4
Stroke	Dominican Republic	both sexes combined	20.8	19.0	22.8	-0.5
Hemorrhagic stroke	Dominican Republic	both sexes combined	7.3	6.5	8.2	0.2
Ischemic stroke	Dominican Republic	both sexes combined	14.7	12.9	16.4	-0.6
Stroke	Grenada	males	16.8	14.9	18.9	-2.9
Hemorrhagic stroke	Grenada	males	6.0	5.1	6.9	-0.4
Ischemic stroke	Grenada	males	11.5	9.9	13.3	-2.6
Stroke	Grenada	females	20.7	18.4	23.2	-4.0
Hemorrhagic stroke	Grenada	females	7.6	6.5	8.8	-0.5
Ischemic stroke	Grenada	females	14.2	12.2	16.3	-3.6
Stroke	Grenada	both sexes combined	18.8	17.1	20.7	-3.6
Hemorrhagic stroke	Grenada	both sexes combined	6.8	6.0	7.7	-0.5
Ischemic stroke	Grenada	both sexes combined	12.9	11.4	14.6	-3.3
Stroke	Guyana	males	16.6	14.9	18.5	-4.2
Hemorrhagic stroke	Guyana	males	6.4	5.6	7.3	-1.3
Ischemic stroke	Guyana	males	10.8	9.4	12.3	-2.8
Stroke	Guyana	females	20.7	18.5	23.0	-3.8
Hemorrhagic stroke	Guyana	females	8.1	7.2	9.2	-1.0
Ischemic stroke	Guyana	females	13.5	11.6	15.3	-2.8
Stroke	Guyana	both sexes combined	18.5	16.9	20.4	-4.0
Hemorrhagic stroke	Guyana	both sexes combined	7.2	6.4	8.1	-1.1
Ischemic stroke	Guyana	both sexes combined	12.0	10.6	13.5	-2.8
Stroke	Haiti	males	16.4	13.7	19.2	0.0
Hemorrhagic stroke	Haiti	males	5.5	4.5	6.6	0.1
Ischemic stroke	Haiti	males	11.5	9.2	13.7	-0.1
Stroke	Haiti	females	18.2	15.5	21.6	0.0
Hemorrhagic stroke	Haiti	females	6.9	5.6	8.3	0.0
Ischemic stroke	Haiti	females	11.8	9.6	14.4	0.1
Stroke	Haiti	both sexes combined	17.4	15.2	19.9	-0.1
Hemorrhagic stroke	Haiti	both sexes combined	6.3	5.3	7.3	0.0
Ischemic stroke	Haiti	both sexes combined	11.7	9.8	13.6	0.0
Stroke	Jamaica	males	21.2	18.5	24.0	-3.1
Hemorrhagic stroke	Jamaica	males	7.8	6.6	9.0	-0.3
Ischemic stroke	Jamaica	males	14.6	12.3	16.9	-3.0
Stroke	Jamaica	females	24.6	21.6	27.7	-3.6
Hemorrhagic stroke	Jamaica	females	9.3	7.9	10.8	-0.5
Ischemic stroke	Jamaica	females	16.8	14.3	19.6	-3.3
Stroke	Jamaica	both sexes combined	22.8	20.6	25.2	-3.6
Hemorrhagic stroke	Jamaica	both sexes combined	8.5	7.5	9.7	-0.5
Ischemic stroke	Jamaica	both sexes combined	15.6	13.6	17.8	-3.3
Stroke	Saint Lucia	males	18.3	16.9	19.8	-1.6
Hemorrhagic stroke	Saint Lucia	males	6.8	6.2	7.6	0.1
Ischemic stroke	Saint Lucia	males	12.3	10.9	13.8	-1.7
Stroke	Saint Lucia	females	22.3	20.7	23.9	-2.0
Hemorrhagic stroke	Saint Lucia	females	8.4	7.6	9.3	0.0
Ischemic stroke	Saint Lucia	females	15.2	13.6	16.7	-1.9
Stroke	Saint Lucia	both sexes combined	20.2	18.9	21.7	-1.9
Hemorrhagic stroke	Saint Lucia	both sexes combined	7.6	6.9	8.3	0.0
Ischemic stroke	Saint Lucia	both sexes combined	13.7	12.3	15.1	-1.9
Stroke	Saint Vincent and the Grenadines	males	16.5	14.9	17.9	-0.8
Hemorrhagic stroke	Saint Vincent and the Grenadines	males	6.2	5.5	6.9	0.3
Ischemic stroke	Saint Vincent and the Grenadines	males	11.0	9.6	12.3	-1.2
Stroke	Saint Vincent and the Grenadines	females	20.0	18.3	22.0	-1.5
Hemorrhagic stroke	Saint Vincent and the Grenadines	females	7.3	6.5	8.3	0.1
Ischemic stroke	Saint Vincent and the Grenadines	females	13.7	12.1	15.5	-1.6
Stroke	Saint Vincent and the Grenadines	both sexes combined	18.1	16.7	19.5	-1.4
Hemorrhagic stroke	Saint Vincent and the Grenadines	both sexes combined	6.7	6.1	7.5	0.1
Ischemic stroke	Saint Vincent and the Grenadines	both sexes combined	12.3	10.9	13.7	-1.6
Stroke	Suriname	males	19.6	18.0	21.3	-1.0
Hemorrhagic stroke	Suriname	males	7.7	7.0	8.8	0.4
Ischemic stroke	Suriname	males	12.8	11.3	14.3	-1.6
Stroke	Suriname	females	23.5	21.5	25.4	-1.0
Hemorrhagic stroke	Suriname	females	9.4	8.4	10.4	0.5
Ischemic stroke	Suriname	females	15.5	13.9	17.1	-1.5
Stroke	Suriname	both sexes combined	21.5	20.0	23.1	-1.0
Hemorrhagic stroke	Suriname	both sexes combined	8.6	7.7	9.5	0.5
Ischemic stroke	Suriname	both sexes combined	14.1	12.7	15.6	-1.6
Stroke	Trinidad and Tobago	males	17.6	16.1	19.2	-1.7
Hemorrhagic stroke	Trinidad and Tobago	males	6.4	5.7	7.1	0.1
Ischemic stroke	Trinidad and Tobago	males	12.2	10.7	13.7	-1.8
Stroke	Trinidad and Tobago	females	21.6	19.9	23.5	-1.2
Hemorrhagic stroke	Trinidad and Tobago	females	7.8	7.0	8.7	0.3
Ischemic stroke	Trinidad and Tobago	females	15.2	13.5	16.9	-1.4
Stroke	Trinidad and Tobago	both sexes combined	19.5	18.0	21.0	-1.5

Hemorrhagic stroke	Trinidad and Tobago	both sexes combined	7.1	6.4	7.8	0.2
Ischemic stroke	Trinidad and Tobago	both sexes combined	13.6	12.1	15.0	-1.6
Stroke	Andean Latin America	males	15.5	14.0	17.0	-0.1
Hemorrhagic stroke	Andean Latin America	males	4.7	4.1	5.2	0.1
Ischemic stroke	Andean Latin America	males	11.5	10.0	12.9	-0.2
Stroke	Andean Latin America	females	17.9	16.2	19.6	0.0
Hemorrhagic stroke	Andean Latin America	females	5.7	5.1	6.4	0.2
Ischemic stroke	Andean Latin America	females	13.1	11.5	14.7	-0.1
Stroke	Andean Latin America	both sexes combined	16.7	15.2	18.2	-0.1
Hemorrhagic stroke	Andean Latin America	both sexes combined	5.2	4.6	5.7	0.2
Ischemic stroke	Andean Latin America	both sexes combined	12.3	10.9	13.6	-0.1
Stroke	Bolivia	males	16.2	14.2	18.5	1.2
Hemorrhagic stroke	Bolivia	males	5.0	4.3	5.7	0.2
Ischemic stroke	Bolivia	males	11.9	10.1	13.9	1.2
Stroke	Bolivia	females	18.0	15.0	20.9	1.7
Hemorrhagic stroke	Bolivia	females	6.0	4.9	7.1	0.3
Ischemic stroke	Bolivia	females	13.0	10.4	15.5	1.6
Stroke	Bolivia	both sexes combined	17.1	15.2	19.2	1.5
Hemorrhagic stroke	Bolivia	both sexes combined	5.5	4.8	6.2	0.3
Ischemic stroke	Bolivia	both sexes combined	12.4	10.5	14.3	1.4
Stroke	Ecuador	males	15.0	13.7	16.3	-0.7
Hemorrhagic stroke	Ecuador	males	4.7	4.1	5.2	0.1
Ischemic stroke	Ecuador	males	11.0	9.8	12.2	-0.8
Stroke	Ecuador	females	17.6	16.1	19.1	-0.4
Hemorrhagic stroke	Ecuador	females	5.7	5.1	6.4	0.3
Ischemic stroke	Ecuador	females	12.8	11.2	14.2	-0.6
Stroke	Ecuador	both sexes combined	16.3	15.1	17.6	-0.6
Hemorrhagic stroke	Ecuador	both sexes combined	5.2	4.7	5.7	0.2
Ischemic stroke	Ecuador	both sexes combined	11.9	10.7	13.0	-0.7
Stroke	Peru	males	15.5	13.5	17.6	-0.4
Hemorrhagic stroke	Peru	males	4.5	3.9	5.2	0.0
Ischemic stroke	Peru	males	11.6	9.8	13.4	-0.4
Stroke	Peru	females	18.0	15.9	20.2	-0.6
Hemorrhagic stroke	Peru	females	5.6	4.9	6.5	0.1
Ischemic stroke	Peru	females	13.3	11.4	15.3	-0.7
Stroke	Peru	both sexes combined	16.8	15.0	18.5	-0.5
Hemorrhagic stroke	Peru	both sexes combined	5.1	4.5	5.8	0.1
Ischemic stroke	Peru	both sexes combined	12.5	10.8	14.1	-0.5
Stroke	Central Latin America	males	14.1	13.1	15.1	0.0
Hemorrhagic stroke	Central Latin America	males	4.7	4.2	5.1	0.1
Ischemic stroke	Central Latin America	males	10.0	9.0	11.0	0.0
Stroke	Central Latin America	females	16.4	15.2	17.6	-0.4
Hemorrhagic stroke	Central Latin America	females	5.7	5.2	6.2	-0.2
Ischemic stroke	Central Latin America	females	11.5	10.3	12.7	-0.2
Stroke	Central Latin America	both sexes combined	15.2	14.2	16.4	-0.2
Hemorrhagic stroke	Central Latin America	both sexes combined	5.2	4.7	5.7	-0.1
Ischemic stroke	Central Latin America	both sexes combined	10.7	9.7	11.8	-0.1
Stroke	Colombia	males	14.7	13.4	16.2	-0.4
Hemorrhagic stroke	Colombia	males	5.2	4.5	5.8	-0.4
Ischemic stroke	Colombia	males	10.2	9.0	11.5	0.0
Stroke	Colombia	females	17.0	15.6	18.5	-1.6
Hemorrhagic stroke	Colombia	females	6.3	5.6	7.0	-0.9
Ischemic stroke	Colombia	females	11.6	10.2	13.0	-0.8
Stroke	Colombia	both sexes combined	15.9	14.6	17.2	-1.0
Hemorrhagic stroke	Colombia	both sexes combined	5.7	5.1	6.3	-0.6
Ischemic stroke	Colombia	both sexes combined	10.9	9.7	12.1	-0.4
Stroke	Costa Rica	males	15.4	14.1	16.9	0.1
Hemorrhagic stroke	Costa Rica	males	5.0	4.5	5.6	0.6
Ischemic stroke	Costa Rica	males	11.1	9.9	12.4	-0.4
Stroke	Costa Rica	females	17.6	16.1	19.1	0.0
Hemorrhagic stroke	Costa Rica	females	5.8	5.2	6.5	0.5
Ischemic stroke	Costa Rica	females	12.7	11.2	14.2	-0.4
Stroke	Costa Rica	both sexes combined	16.5	15.2	17.9	0.1
Hemorrhagic stroke	Costa Rica	both sexes combined	5.4	4.9	6.0	0.5
Ischemic stroke	Costa Rica	both sexes combined	11.9	10.6	13.2	-0.4
Stroke	El Salvador	males	12.0	10.7	13.5	-1.7
Hemorrhagic stroke	El Salvador	males	3.8	3.3	4.3	-0.6
Ischemic stroke	El Salvador	males	8.7	7.6	10.0	-1.1
Stroke	El Salvador	females	14.5	13.1	16.0	-2.2
Hemorrhagic stroke	El Salvador	females	4.9	4.3	5.5	-0.6
Ischemic stroke	El Salvador	females	10.3	9.0	11.6	-1.6
Stroke	El Salvador	both sexes combined	13.4	12.2	14.6	-1.9
Hemorrhagic stroke	El Salvador	both sexes combined	4.4	3.9	4.9	-0.6
Ischemic stroke	El Salvador	both sexes combined	9.6	8.5	10.7	-1.3
Stroke	Guatemala	males	13.0	10.5	15.8	2.2
Hemorrhagic stroke	Guatemala	males	4.2	3.3	5.1	1.1
Ischemic stroke	Guatemala	males	9.4	7.4	11.6	1.2
Stroke	Guatemala	females	15.1	12.5	18.1	2.8
Hemorrhagic stroke	Guatemala	females	5.0	4.1	6.0	1.2
Ischemic stroke	Guatemala	females	10.8	8.5	13.4	1.8
Stroke	Guatemala	both sexes combined	14.0	12.1	16.3	2.5

Hemorrhagic stroke	Guatemala	both sexes combined	4.6	3.8	5.3	1.2
Ischemic stroke	Guatemala	both sexes combined	10.1	8.3	12.0	1.5
Stroke	Honduras	males	16.3	12.8	19.6	0.4
Hemorrhagic stroke	Honduras	males	4.7	3.6	5.7	0.0
Ischemic stroke	Honduras	males	12.4	9.5	15.2	0.6
Stroke	Honduras	females	14.1	11.7	17.1	0.9
Hemorrhagic stroke	Honduras	females	4.1	3.3	5.0	0.0
Ischemic stroke	Honduras	females	10.6	8.6	13.1	1.1
Stroke	Honduras	both sexes combined	15.1	12.8	17.4	0.8
Hemorrhagic stroke	Honduras	both sexes combined	4.3	3.6	5.1	0.0
Ischemic stroke	Honduras	both sexes combined	11.4	9.5	13.4	0.9
Stroke	Mexico	males	13.3	12.3	14.3	0.3
Hemorrhagic stroke	Mexico	males	4.3	3.9	4.7	0.1
Ischemic stroke	Mexico	males	9.5	8.5	10.5	0.2
Stroke	Mexico	females	15.8	14.7	16.9	-0.1
Hemorrhagic stroke	Mexico	females	5.3	4.8	5.9	-0.1
Ischemic stroke	Mexico	females	11.2	10.1	12.3	0.0
Stroke	Mexico	both sexes combined	14.5	13.5	15.6	0.1
Hemorrhagic stroke	Mexico	both sexes combined	4.8	4.4	5.3	0.0
Ischemic stroke	Mexico	both sexes combined	10.3	9.3	11.4	0.1
Stroke	Nicaragua	males	16.7	14.6	18.8	-2.5
Hemorrhagic stroke	Nicaragua	males	5.7	4.9	6.6	-0.4
Ischemic stroke	Nicaragua	males	11.8	10.0	13.5	-2.2
Stroke	Nicaragua	females	18.9	17.0	21.2	-2.3
Hemorrhagic stroke	Nicaragua	females	6.6	5.8	7.6	-0.5
Ischemic stroke	Nicaragua	females	13.3	11.6	15.4	-1.9
Stroke	Nicaragua	both sexes combined	17.8	16.1	19.6	-2.4
Hemorrhagic stroke	Nicaragua	both sexes combined	6.2	5.4	7.0	-0.4
Ischemic stroke	Nicaragua	both sexes combined	12.6	11.0	14.3	-2.1
Stroke	Panama	males	18.8	16.7	20.8	-1.8
Hemorrhagic stroke	Panama	males	6.7	5.8	7.6	0.1
Ischemic stroke	Panama	males	13.1	11.4	14.9	-2.0
Stroke	Panama	females	20.5	18.5	22.5	-2.4
Hemorrhagic stroke	Panama	females	7.3	6.4	8.2	-0.2
Ischemic stroke	Panama	females	14.4	12.6	16.3	-2.3
Stroke	Panama	both sexes combined	19.6	17.9	21.3	-2.1
Hemorrhagic stroke	Panama	both sexes combined	6.9	6.2	7.7	0.0
Ischemic stroke	Panama	both sexes combined	13.7	12.1	15.3	-2.1
Stroke	Venezuela	males	15.8	13.6	17.9	-0.9
Hemorrhagic stroke	Venezuela	males	5.8	4.9	6.7	0.3
Ischemic stroke	Venezuela	males	10.8	9.0	12.5	-1.2
Stroke	Venezuela	females	18.8	16.5	21.1	-0.8
Hemorrhagic stroke	Venezuela	females	7.0	6.0	8.0	0.2
Ischemic stroke	Venezuela	females	12.8	10.9	14.7	-1.0
Stroke	Venezuela	both sexes combined	17.2	15.5	19.0	-0.9
Hemorrhagic stroke	Venezuela	both sexes combined	6.4	5.5	7.2	0.2
Ischemic stroke	Venezuela	both sexes combined	11.7	10.2	13.3	-1.1
Stroke	Tropical Latin America	males	18.9	17.6	20.2	-2.2
Hemorrhagic stroke	Tropical Latin America	males	4.8	4.3	5.2	-0.5
Ischemic stroke	Tropical Latin America	males	14.9	13.6	16.3	-1.6
Stroke	Tropical Latin America	females	19.5	18.1	20.9	-3.5
Hemorrhagic stroke	Tropical Latin America	females	5.8	5.2	6.3	-0.4
Ischemic stroke	Tropical Latin America	females	14.7	13.3	16.1	-3.1
Stroke	Tropical Latin America	both sexes combined	19.1	17.9	20.5	-2.8
Hemorrhagic stroke	Tropical Latin America	both sexes combined	5.3	4.8	5.7	-0.5
Ischemic stroke	Tropical Latin America	both sexes combined	14.7	13.4	16.2	-2.3
Stroke	Brazil	males	18.9	17.6	20.2	-2.1
Hemorrhagic stroke	Brazil	males	4.7	4.3	5.1	-0.5
Ischemic stroke	Brazil	males	15.0	13.7	16.3	-1.6
Stroke	Brazil	females	19.5	18.1	20.9	-3.5
Hemorrhagic stroke	Brazil	females	5.7	5.1	6.2	-0.4
Ischemic stroke	Brazil	females	14.7	13.3	16.1	-3.1
Stroke	Brazil	both sexes combined	19.1	17.9	20.5	-2.8
Hemorrhagic stroke	Brazil	both sexes combined	5.2	4.7	5.6	-0.5
Ischemic stroke	Brazil	both sexes combined	14.8	13.5	16.2	-2.3
Stroke	Paraguay	males	18.8	16.8	20.6	-4.1
Hemorrhagic stroke	Paraguay	males	7.8	6.9	8.9	-2.1
Ischemic stroke	Paraguay	males	12.0	10.4	13.5	-2.5
Stroke	Paraguay	females	20.4	18.5	22.6	-3.4
Hemorrhagic stroke	Paraguay	females	9.4	8.3	10.6	-1.9
Ischemic stroke	Paraguay	females	12.3	10.7	14.0	-1.7
Stroke	Paraguay	both sexes combined	19.5	17.9	21.2	-3.8
Hemorrhagic stroke	Paraguay	both sexes combined	8.6	7.7	9.5	-2.0
Ischemic stroke	Paraguay	both sexes combined	12.1	10.6	13.5	-2.1
Stroke	North Africa and Middle East	males	19.4	17.8	20.9	1.8
Hemorrhagic stroke	North Africa and Middle East	males	5.2	4.7	5.8	0.2
Ischemic stroke	North Africa and Middle East	males	15.0	13.5	16.5	1.7
Stroke	North Africa and Middle East	females	23.1	21.4	24.8	0.8
Hemorrhagic stroke	North Africa and Middle East	females	5.9	5.3	6.6	-0.2
Ischemic stroke	North Africa and Middle East	females	18.3	16.5	20.1	1.1
Stroke	North Africa and Middle East	both sexes combined	21.2	19.6	22.8	1.3

Hemorrhagic stroke	North Africa and Middle East	both sexes combined	5.6	5.0	6.1	0.0
Ischemic stroke	North Africa and Middle East	both sexes combined	16.7	15.0	18.2	1.4
Stroke	North Africa and Middle East	males	19.4	17.8	20.9	1.8
Hemorrhagic stroke	North Africa and Middle East	males	5.2	4.7	5.8	0.2
Ischemic stroke	North Africa and Middle East	males	15.0	13.5	16.5	1.7
Stroke	North Africa and Middle East	females	23.1	21.4	24.8	0.8
Hemorrhagic stroke	North Africa and Middle East	females	5.9	5.3	6.6	-0.2
Ischemic stroke	North Africa and Middle East	females	18.3	16.5	20.1	1.1
Stroke	North Africa and Middle East	both sexes combined	21.2	19.6	22.8	1.3
Hemorrhagic stroke	North Africa and Middle East	both sexes combined	5.6	5.0	6.1	0.0
Ischemic stroke	North Africa and Middle East	both sexes combined	16.6	15.0	18.2	1.4
Stroke	Algeria	males	21.7	19.6	23.7	1.3
Hemorrhagic stroke	Algeria	males	6.2	5.4	7.1	-0.1
Ischemic stroke	Algeria	males	16.6	14.5	18.6	1.5
Stroke	Algeria	females	24.0	21.9	26.2	0.3
Hemorrhagic stroke	Algeria	females	6.3	5.6	7.1	-0.3
Ischemic stroke	Algeria	females	18.9	16.8	21.1	0.7
Stroke	Algeria	both sexes combined	22.8	21.0	24.7	0.8
Hemorrhagic stroke	Algeria	both sexes combined	6.3	5.6	7.0	-0.2
Ischemic stroke	Algeria	both sexes combined	17.8	15.9	19.7	1.1
Stroke	Bahrain	males	16.2	13.8	18.6	2.7
Hemorrhagic stroke	Bahrain	males	4.1	3.4	4.8	0.0
Ischemic stroke	Bahrain	males	12.8	10.7	15.0	2.8
Stroke	Bahrain	females	18.6	16.2	21.1	1.1
Hemorrhagic stroke	Bahrain	females	4.5	3.7	5.3	-0.5
Ischemic stroke	Bahrain	females	14.9	12.7	17.2	1.8
Stroke	Bahrain	both sexes combined	17.3	15.3	19.3	2.0
Hemorrhagic stroke	Bahrain	both sexes combined	4.3	3.7	4.9	-0.2
Ischemic stroke	Bahrain	both sexes combined	13.8	11.9	15.6	2.4
Stroke	Egypt	males	18.6	16.4	21.0	1.6
Hemorrhagic stroke	Egypt	males	5.1	4.4	6.0	0.0
Ischemic stroke	Egypt	males	14.3	12.3	16.4	1.7
Stroke	Egypt	females	24.3	21.8	27.1	0.9
Hemorrhagic stroke	Egypt	females	6.2	5.4	7.2	-0.2
Ischemic stroke	Egypt	females	19.3	16.9	21.9	1.2
Stroke	Egypt	both sexes combined	21.4	19.5	23.5	1.1
Hemorrhagic stroke	Egypt	both sexes combined	5.6	5.0	6.4	-0.1
Ischemic stroke	Egypt	both sexes combined	16.7	15.0	18.8	1.4
Stroke	Iran	males	19.7	17.2	22.4	-1.0
Hemorrhagic stroke	Iran	males	3.7	3.2	4.4	0.3
Ischemic stroke	Iran	males	16.7	14.4	19.2	-1.3
Stroke	Iran	females	22.9	20.2	25.9	-1.8
Hemorrhagic stroke	Iran	females	4.2	3.7	4.9	0.0
Ischemic stroke	Iran	females	19.6	17.0	22.4	-1.9
Stroke	Iran	both sexes combined	21.1	19.0	23.3	-1.4
Hemorrhagic stroke	Iran	both sexes combined	3.9	3.5	4.5	0.2
Ischemic stroke	Iran	both sexes combined	18.0	15.9	20.2	-1.5
Stroke	Iraq	males	20.1	16.9	24.0	-1.0
Hemorrhagic stroke	Iraq	males	5.7	4.6	7.1	-0.6
Ischemic stroke	Iraq	males	15.3	12.7	18.5	-0.5
Stroke	Iraq	females	23.5	20.1	27.3	-0.3
Hemorrhagic stroke	Iraq	females	6.5	5.4	7.7	-0.5
Ischemic stroke	Iraq	females	18.2	15.3	21.7	0.2
Stroke	Iraq	both sexes combined	21.8	19.4	24.7	-0.7
Hemorrhagic stroke	Iraq	both sexes combined	6.1	5.2	7.1	-0.6
Ischemic stroke	Iraq	both sexes combined	16.7	14.6	19.2	-0.2
Stroke	Jordan	males	22.4	18.6	26.4	-0.6
Hemorrhagic stroke	Jordan	males	5.0	4.0	6.1	-0.1
Ischemic stroke	Jordan	males	18.5	15.0	22.1	-0.4
Stroke	Jordan	females	25.4	21.9	29.2	-1.1
Hemorrhagic stroke	Jordan	females	5.4	4.5	6.3	-0.1
Ischemic stroke	Jordan	females	21.3	18.0	24.9	-0.9
Stroke	Jordan	both sexes combined	23.8	21.0	26.8	-0.8
Hemorrhagic stroke	Jordan	both sexes combined	5.2	4.4	6.0	-0.1
Ischemic stroke	Jordan	both sexes combined	19.8	17.2	22.8	-0.7
Stroke	Kuwait	males	22.4	18.8	25.8	5.4
Hemorrhagic stroke	Kuwait	males	5.0	4.1	6.0	1.1
Ischemic stroke	Kuwait	males	18.5	15.2	21.7	4.7
Stroke	Kuwait	females	24.4	20.6	28.4	5.4
Hemorrhagic stroke	Kuwait	females	4.8	4.0	5.9	0.9
Ischemic stroke	Kuwait	females	20.8	17.3	24.5	4.9
Stroke	Kuwait	both sexes combined	23.2	20.5	26.1	5.2
Hemorrhagic stroke	Kuwait	both sexes combined	4.9	4.2	5.7	1.0
Ischemic stroke	Kuwait	both sexes combined	19.4	16.9	22.2	4.6
Stroke	Lebanon	males	15.0	13.2	16.7	4.0
Hemorrhagic stroke	Lebanon	males	3.4	2.9	3.9	0.7
Ischemic stroke	Lebanon	males	12.2	10.5	13.9	3.6
Stroke	Lebanon	females	23.9	21.5	26.4	-0.8
Hemorrhagic stroke	Lebanon	females	6.3	5.4	7.3	-0.7
Ischemic stroke	Lebanon	females	19.1	16.7	21.5	0.0
Stroke	Lebanon	both sexes combined	19.5	17.6	21.5	1.9

Hemorrhagic stroke	Lebanon	both sexes combined	4.8	4.2	5.5	0.1
Ischemic stroke	Lebanon	both sexes combined	15.7	13.8	17.5	2.0
Stroke	Libya	males	20.0	17.9	22.5	1.8
Hemorrhagic stroke	Libya	males	5.5	4.8	6.3	0.1
Ischemic stroke	Libya	males	15.6	13.5	17.8	1.8
Stroke	Libya	females	23.9	21.5	26.1	1.5
Hemorrhagic stroke	Libya	females	6.0	5.3	6.8	0.0
Ischemic stroke	Libya	females	19.2	16.9	21.4	1.7
Stroke	Libya	both sexes combined	21.9	20.0	23.9	1.7
Hemorrhagic stroke	Libya	both sexes combined	5.8	5.1	6.5	0.1
Ischemic stroke	Libya	both sexes combined	17.4	15.4	19.4	1.8
Stroke	Morocco	males	20.0	18.3	21.9	2.8
Hemorrhagic stroke	Morocco	males	5.8	5.1	6.4	0.5
Ischemic stroke	Morocco	males	15.3	13.5	17.1	2.6
Stroke	Morocco	females	22.8	20.5	25.5	2.8
Hemorrhagic stroke	Morocco	females	6.0	5.1	6.8	0.4
Ischemic stroke	Morocco	females	18.1	15.9	20.7	2.6
Stroke	Morocco	both sexes combined	21.5	19.7	23.5	2.8
Hemorrhagic stroke	Morocco	both sexes combined	5.9	5.2	6.6	0.4
Ischemic stroke	Morocco	both sexes combined	16.7	14.9	18.7	2.6
Stroke	Palestine	males	19.3	17.8	21.0	0.6
Hemorrhagic stroke	Palestine	males	5.2	4.7	5.8	-5.1
Ischemic stroke	Palestine	males	14.9	13.3	16.6	5.3
Stroke	Palestine	females	24.3	22.3	26.2	1.4
Hemorrhagic stroke	Palestine	females	6.0	5.3	6.7	-7.2
Ischemic stroke	Palestine	females	19.4	17.3	21.4	8.2
Stroke	Palestine	both sexes combined	21.8	20.1	23.5	0.9
Hemorrhagic stroke	Palestine	both sexes combined	5.6	5.0	6.2	-6.2
Ischemic stroke	Palestine	both sexes combined	17.2	15.5	18.9	6.7
Stroke	Oman	males	17.6	16.1	19.1	1.2
Hemorrhagic stroke	Oman	males	7.4	6.6	8.2	-0.5
Ischemic stroke	Oman	males	11.2	9.8	12.6	1.9
Stroke	Oman	females	20.1	18.4	21.8	1.5
Hemorrhagic stroke	Oman	females	7.4	6.6	8.3	-1.7
Ischemic stroke	Oman	females	13.9	12.3	15.5	3.3
Stroke	Oman	both sexes combined	18.4	17.0	19.9	1.0
Hemorrhagic stroke	Oman	both sexes combined	7.4	6.6	8.2	-1.1
Ischemic stroke	Oman	both sexes combined	12.1	10.8	13.5	2.2
Stroke	Qatar	males	19.8	16.5	23.7	0.0
Hemorrhagic stroke	Qatar	males	5.0	4.0	6.1	-0.7
Ischemic stroke	Qatar	males	15.8	12.9	19.2	0.7
Stroke	Qatar	females	23.9	20.6	27.8	-0.8
Hemorrhagic stroke	Qatar	females	6.2	5.1	7.5	-2.1
Ischemic stroke	Qatar	females	19.2	16.3	22.8	1.1
Stroke	Qatar	both sexes combined	21.2	18.3	24.1	-0.7
Hemorrhagic stroke	Qatar	both sexes combined	5.5	4.6	6.4	-1.4
Ischemic stroke	Qatar	both sexes combined	16.9	14.3	19.5	0.5
Stroke	Saudi Arabia	males	21.3	19.5	23.1	-0.7
Hemorrhagic stroke	Saudi Arabia	males	5.3	4.7	6.0	-0.9
Ischemic stroke	Saudi Arabia	males	16.9	15.1	18.7	0.1
Stroke	Saudi Arabia	females	25.1	23.1	27.3	-0.6
Hemorrhagic stroke	Saudi Arabia	females	6.2	5.5	6.9	-0.9
Ischemic stroke	Saudi Arabia	females	20.3	18.2	22.4	0.3
Stroke	Saudi Arabia	both sexes combined	23.0	21.2	24.8	-0.8
Hemorrhagic stroke	Saudi Arabia	both sexes combined	5.7	5.1	6.4	-0.9
Ischemic stroke	Saudi Arabia	both sexes combined	18.4	16.5	20.3	0.1
Stroke	Syria	males	18.6	16.9	20.3	-0.4
Hemorrhagic stroke	Syria	males	5.7	5.0	6.4	-0.8
Ischemic stroke	Syria	males	13.8	12.2	15.4	0.4
Stroke	Syria	females	20.9	19.3	22.7	-2.0
Hemorrhagic stroke	Syria	females	5.8	5.2	6.5	-1.4
Ischemic stroke	Syria	females	16.2	14.4	18.1	-0.7
Stroke	Syria	both sexes combined	19.8	18.2	21.3	-1.2
Hemorrhagic stroke	Syria	both sexes combined	5.7	5.1	6.3	-1.1
Ischemic stroke	Syria	both sexes combined	15.0	13.3	16.6	-0.1
Stroke	Tunisia	males	21.0	18.2	24.2	1.3
Hemorrhagic stroke	Tunisia	males	5.9	4.9	7.0	-0.2
Ischemic stroke	Tunisia	males	16.2	13.7	19.0	1.6
Stroke	Tunisia	females	23.8	21.1	26.8	1.4
Hemorrhagic stroke	Tunisia	females	6.3	5.4	7.5	0.0
Ischemic stroke	Tunisia	females	18.8	16.2	21.6	1.6
Stroke	Tunisia	both sexes combined	22.3	20.0	24.6	1.4
Hemorrhagic stroke	Tunisia	both sexes combined	6.1	5.3	7.0	-0.1
Ischemic stroke	Tunisia	both sexes combined	17.5	15.2	19.6	1.6
Stroke	Turkey	males	18.9	16.7	21.3	5.2
Hemorrhagic stroke	Turkey	males	5.6	4.8	6.4	1.3
Ischemic stroke	Turkey	males	14.3	12.3	16.4	4.3
Stroke	Turkey	females	23.4	21.0	25.9	2.6
Hemorrhagic stroke	Turkey	females	6.6	5.7	7.6	0.3
Ischemic stroke	Turkey	females	18.2	15.9	20.4	2.6
Stroke	Turkey	both sexes combined	21.2	19.2	23.2	4.0

Hemorrhagic stroke	Turkey	both sexes combined	6.1	5.4	6.9	0.8
Ischemic stroke	Turkey	both sexes combined	16.3	14.4	18.1	3.5
Stroke	United Arab Emirates	males	23.1	20.4	25.9	-2.9
Hemorrhagic stroke	United Arab Emirates	males	5.7	4.8	6.6	-1.0
Ischemic stroke	United Arab Emirates	males	18.6	16.1	21.2	-2.3
Stroke	United Arab Emirates	females	26.8	23.6	29.6	-3.0
Hemorrhagic stroke	United Arab Emirates	females	6.7	5.8	7.6	-1.2
Ischemic stroke	United Arab Emirates	females	21.6	18.7	24.2	-2.1
Stroke	United Arab Emirates	both sexes combined	24.0	21.5	26.6	-3.4
Hemorrhagic stroke	United Arab Emirates	both sexes combined	6.0	5.2	6.8	-1.2
Ischemic stroke	United Arab Emirates	both sexes combined	19.3	16.9	21.7	-2.7
Stroke	Yemen	males	19.7	17.6	21.9	1.9
Hemorrhagic stroke	Yemen	males	5.8	5.0	6.7	0.0
Ischemic stroke	Yemen	males	14.7	12.9	16.6	1.9
Stroke	Yemen	females	22.7	20.8	24.6	1.2
Hemorrhagic stroke	Yemen	females	6.2	5.5	6.9	-0.5
Ischemic stroke	Yemen	females	17.4	15.5	19.2	1.8
Stroke	Yemen	both sexes combined	21.3	19.4	23.3	1.3
Hemorrhagic stroke	Yemen	both sexes combined	6.0	5.3	6.8	-0.3
Ischemic stroke	Yemen	both sexes combined	16.1	14.4	17.9	1.7
Stroke	South Asia	males	13.5	12.5	14.5	1.8
Hemorrhagic stroke	South Asia	males	4.2	3.8	4.7	0.1
Ischemic stroke	South Asia	males	9.6	8.6	10.6	1.8
Stroke	South Asia	females	15.9	14.7	17.1	2.6
Hemorrhagic stroke	South Asia	females	4.7	4.3	5.3	0.3
Ischemic stroke	South Asia	females	11.6	10.4	12.9	2.5
Stroke	South Asia	both sexes combined	14.6	13.6	15.7	2.2
Hemorrhagic stroke	South Asia	both sexes combined	4.5	4.0	4.9	0.2
Ischemic stroke	South Asia	both sexes combined	10.6	9.5	11.6	2.2
Stroke	South Asia	males	13.5	12.5	14.5	1.8
Hemorrhagic stroke	South Asia	males	4.2	3.8	4.7	0.1
Ischemic stroke	South Asia	males	9.6	8.6	10.6	1.8
Stroke	South Asia	females	15.9	14.7	17.1	2.6
Hemorrhagic stroke	South Asia	females	4.7	4.3	5.3	0.3
Ischemic stroke	South Asia	females	11.6	10.4	12.9	2.5
Stroke	South Asia	both sexes combined	14.6	13.6	15.7	2.2
Hemorrhagic stroke	South Asia	both sexes combined	4.5	4.0	4.9	0.2
Ischemic stroke	South Asia	both sexes combined	10.6	9.5	11.6	2.2
Stroke	Afghanistan	males	15.6	13.9	17.3	1.0
Hemorrhagic stroke	Afghanistan	males	5.1	4.4	5.9	0.0
Ischemic stroke	Afghanistan	males	10.9	9.4	12.4	0.9
Stroke	Afghanistan	females	17.6	15.5	19.6	0.9
Hemorrhagic stroke	Afghanistan	females	5.1	4.4	5.9	-0.3
Ischemic stroke	Afghanistan	females	12.8	11.0	14.6	1.1
Stroke	Afghanistan	both sexes combined	16.5	14.9	18.1	0.9
Hemorrhagic stroke	Afghanistan	both sexes combined	5.1	4.5	5.8	-0.1
Ischemic stroke	Afghanistan	both sexes combined	11.8	10.4	13.2	1.0
Stroke	Bangladesh	males	19.8	18.0	21.8	4.6
Hemorrhagic stroke	Bangladesh	males	6.2	5.5	7.1	0.7
Ischemic stroke	Bangladesh	males	14.1	12.5	15.9	3.9
Stroke	Bangladesh	females	24.5	22.2	26.7	4.8
Hemorrhagic stroke	Bangladesh	females	7.8	6.8	8.7	0.6
Ischemic stroke	Bangladesh	females	17.7	15.6	19.8	4.6
Stroke	Bangladesh	both sexes combined	21.9	20.3	23.6	4.6
Hemorrhagic stroke	Bangladesh	both sexes combined	6.9	6.1	7.6	0.6
Ischemic stroke	Bangladesh	both sexes combined	15.7	14.1	17.3	4.2
Stroke	Bhutan	males	15.9	14.1	17.9	1.6
Hemorrhagic stroke	Bhutan	males	5.1	4.4	5.9	-0.2
Ischemic stroke	Bhutan	males	11.3	9.8	13.0	1.9
Stroke	Bhutan	females	18.1	16.1	20.0	1.8
Hemorrhagic stroke	Bhutan	females	5.8	4.9	6.6	-0.5
Ischemic stroke	Bhutan	females	13.1	11.3	14.8	2.6
Stroke	Bhutan	both sexes combined	16.8	15.2	18.4	1.5
Hemorrhagic stroke	Bhutan	both sexes combined	5.4	4.7	6.1	-0.4
Ischemic stroke	Bhutan	both sexes combined	12.0	10.6	13.5	2.1
Stroke	India	males	12.5	11.6	13.4	1.9
Hemorrhagic stroke	India	males	3.9	3.5	4.3	0.1
Ischemic stroke	India	males	8.9	8.0	9.9	1.8
Stroke	India	females	14.8	13.7	15.9	2.6
Hemorrhagic stroke	India	females	4.4	3.9	4.8	0.3
Ischemic stroke	India	females	10.9	9.7	12.1	2.5
Stroke	India	both sexes combined	13.6	12.6	14.6	2.2
Hemorrhagic stroke	India	both sexes combined	4.1	3.7	4.5	0.2
Ischemic stroke	India	both sexes combined	9.9	8.8	10.9	2.1
Stroke	Nepal	males	16.1	14.5	17.8	2.6
Hemorrhagic stroke	Nepal	males	5.1	4.5	5.8	0.1
Ischemic stroke	Nepal	males	11.5	10.0	13.0	2.7
Stroke	Nepal	females	16.3	14.8	17.8	3.1
Hemorrhagic stroke	Nepal	females	5.1	4.5	5.7	0.4
Ischemic stroke	Nepal	females	11.8	10.4	13.2	3.0
Stroke	Nepal	both sexes combined	16.2	14.8	17.5	2.9

Hemorrhagic stroke	Nepal	both sexes combined	5.1	4.6	5.6	0.2
Ischemic stroke	Nepal	both sexes combined	11.6	10.3	12.9	2.9
Stroke	Pakistan	males	16.8	14.8	18.7	-2.0
Hemorrhagic stroke	Pakistan	males	5.5	4.7	6.3	-1.5
Ischemic stroke	Pakistan	males	11.8	10.1	13.4	-0.6
Stroke	Pakistan	females	19.4	16.7	22.1	0.2
Hemorrhagic stroke	Pakistan	females	5.9	5.0	6.9	-0.8
Ischemic stroke	Pakistan	females	14.2	11.9	16.5	1.0
Stroke	Pakistan	both sexes combined	18.0	16.0	19.8	-1.0
Hemorrhagic stroke	Pakistan	both sexes combined	5.7	5.0	6.4	-1.2
Ischemic stroke	Pakistan	both sexes combined	12.9	11.2	14.4	0.1
Stroke	Sub-Saharan Africa	males	11.8	10.9	12.7	0.9
Hemorrhagic stroke	Sub-Saharan Africa	males	4.2	3.7	4.6	0.0
Ischemic stroke	Sub-Saharan Africa	males	8.0	7.1	8.9	0.9
Stroke	Sub-Saharan Africa	females	14.1	13.1	15.3	0.5
Hemorrhagic stroke	Sub-Saharan Africa	females	4.8	4.3	5.3	-0.3
Ischemic stroke	Sub-Saharan Africa	females	9.7	8.7	10.8	0.8
Stroke	Sub-Saharan Africa	both sexes combined	13.0	12.1	14.0	0.7
Hemorrhagic stroke	Sub-Saharan Africa	both sexes combined	4.5	4.1	4.9	-0.2
Ischemic stroke	Sub-Saharan Africa	both sexes combined	8.9	8.0	9.8	0.9
Stroke	Central Sub-Saharan Africa	males	11.6	10.6	12.7	1.3
Hemorrhagic stroke	Central Sub-Saharan Africa	males	4.2	3.7	4.8	0.1
Ischemic stroke	Central Sub-Saharan Africa	males	7.7	6.7	8.7	1.2
Stroke	Central Sub-Saharan Africa	females	13.8	12.6	15.1	0.2
Hemorrhagic stroke	Central Sub-Saharan Africa	females	5.1	4.5	5.6	-0.2
Ischemic stroke	Central Sub-Saharan Africa	females	9.1	8.0	10.3	0.4
Stroke	Central Sub-Saharan Africa	both sexes combined	12.8	11.7	13.8	0.7
Hemorrhagic stroke	Central Sub-Saharan Africa	both sexes combined	4.7	4.2	5.2	-0.1
Ischemic stroke	Central Sub-Saharan Africa	both sexes combined	8.4	7.4	9.4	0.8
Stroke	Angola	males	12.9	10.4	15.2	2.6
Hemorrhagic stroke	Angola	males	4.6	3.6	5.5	0.5
Ischemic stroke	Angola	males	8.7	6.9	10.6	2.3
Stroke	Angola	females	14.8	11.9	17.6	1.2
Hemorrhagic stroke	Angola	females	5.3	4.2	6.3	-0.1
Ischemic stroke	Angola	females	10.0	7.7	12.2	1.4
Stroke	Angola	both sexes combined	14.0	11.3	16.4	2.0
Hemorrhagic stroke	Angola	both sexes combined	5.0	4.0	6.0	0.2
Ischemic stroke	Angola	both sexes combined	9.4	7.3	11.4	1.9
Stroke	Central African Republic	males	7.8	6.1	9.9	-0.5
Hemorrhagic stroke	Central African Republic	males	3.1	2.4	4.0	-0.6
Ischemic stroke	Central African Republic	males	4.8	3.6	6.2	0.0
Stroke	Central African Republic	females	10.4	8.0	13.7	-1.8
Hemorrhagic stroke	Central African Republic	females	3.7	2.8	4.9	-1.2
Ischemic stroke	Central African Republic	females	6.9	5.1	9.4	-0.7
Stroke	Central African Republic	both sexes combined	9.0	7.6	10.9	-1.1
Hemorrhagic stroke	Central African Republic	both sexes combined	3.4	2.7	4.2	-0.9
Ischemic stroke	Central African Republic	both sexes combined	5.8	4.7	7.2	-0.3
Stroke	Congo	males	12.2	9.9	14.6	2.8
Hemorrhagic stroke	Congo	males	4.2	3.3	5.1	0.4
Ischemic stroke	Congo	males	8.4	6.7	10.3	2.6
Stroke	Congo	females	13.0	10.6	16.0	0.0
Hemorrhagic stroke	Congo	females	4.5	3.6	5.6	-0.4
Ischemic stroke	Congo	females	8.8	7.0	11.1	0.5
Stroke	Congo	both sexes combined	12.7	10.8	14.7	1.5
Hemorrhagic stroke	Congo	both sexes combined	4.4	3.6	5.1	0.0
Ischemic stroke	Congo	both sexes combined	8.6	7.1	10.3	1.6
Stroke	Democratic Republic of the Congo	males	11.6	10.5	12.7	1.0
Hemorrhagic stroke	Democratic Republic of the Congo	males	4.3	3.8	4.8	0.1
Ischemic stroke	Democratic Republic of the Congo	males	7.6	6.6	8.6	0.9
Stroke	Democratic Republic of the Congo	females	13.9	12.6	15.2	0.1
Hemorrhagic stroke	Democratic Republic of the Congo	females	5.2	4.6	5.8	-0.2
Ischemic stroke	Democratic Republic of the Congo	females	9.0	7.9	10.2	0.3
Stroke	Democratic Republic of the Congo	both sexes combined	12.8	11.8	13.9	0.5
Hemorrhagic stroke	Democratic Republic of the Congo	both sexes combined	4.8	4.3	5.3	-0.1
Ischemic stroke	Democratic Republic of the Congo	both sexes combined	8.4	7.4	9.4	0.5
Stroke	Equatorial Guinea	males	13.4	10.5	16.6	3.7
Hemorrhagic stroke	Equatorial Guinea	males	4.4	3.4	5.6	0.4
Ischemic stroke	Equatorial Guinea	males	9.6	7.3	12.2	3.8
Stroke	Equatorial Guinea	females	14.8	11.2	19.0	2.2
Hemorrhagic stroke	Equatorial Guinea	females	4.6	3.4	6.1	-0.5
Ischemic stroke	Equatorial Guinea	females	10.9	7.9	14.2	3.1
Stroke	Equatorial Guinea	both sexes combined	14.1	10.9	17.7	2.9
Hemorrhagic stroke	Equatorial Guinea	both sexes combined	4.5	3.4	5.8	-0.1
Ischemic stroke	Equatorial Guinea	both sexes combined	10.2	7.7	13.0	3.4
Stroke	Gabon	males	12.4	9.9	14.9	0.9
Hemorrhagic stroke	Gabon	males	4.4	3.5	5.3	-0.2
Ischemic stroke	Gabon	males	8.5	6.6	10.4	1.2
Stroke	Gabon	females	14.7	12.7	17.3	-0.9
Hemorrhagic stroke	Gabon	females	5.0	4.2	5.9	-0.7
Ischemic stroke	Gabon	females	10.2	8.5	12.3	-0.3
Stroke	Gabon	both sexes combined	13.5	11.7	15.5	0.1

Hemorrhagic stroke	Gabon	both sexes combined	4.7	4.0	5.5	-0.4
Ischemic stroke	Gabon	both sexes combined	9.3	7.8	10.9	0.5
Stroke	Eastern Sub-Saharan Africa	males	11.2	10.3	12.3	1.4
Hemorrhagic stroke	Eastern Sub-Saharan Africa	males	4.0	3.6	4.5	0.0
Ischemic stroke	Eastern Sub-Saharan Africa	males	7.4	6.5	8.4	1.4
Stroke	Eastern Sub-Saharan Africa	females	12.5	11.4	13.6	0.8
Hemorrhagic stroke	Eastern Sub-Saharan Africa	females	4.5	4.0	5.0	-0.5
Ischemic stroke	Eastern Sub-Saharan Africa	females	8.2	7.3	9.2	1.3
Stroke	Eastern Sub-Saharan Africa	both sexes combined	11.8	10.9	12.8	1.1
Hemorrhagic stroke	Eastern Sub-Saharan Africa	both sexes combined	4.3	3.8	4.8	-0.2
Ischemic stroke	Eastern Sub-Saharan Africa	both sexes combined	7.9	6.9	8.8	1.4
Stroke	Burundi	males	10.4	8.7	12.1	1.1
Hemorrhagic stroke	Burundi	males	3.9	3.2	4.7	-0.2
Ischemic stroke	Burundi	males	6.7	5.5	8.0	1.4
Stroke	Burundi	females	11.6	9.9	13.2	-0.1
Hemorrhagic stroke	Burundi	females	4.4	3.7	5.2	-0.7
Ischemic stroke	Burundi	females	7.3	6.1	8.6	0.7
Stroke	Burundi	both sexes combined	11.0	9.7	12.4	0.5
Hemorrhagic stroke	Burundi	both sexes combined	4.2	3.6	4.8	-0.4
Ischemic stroke	Burundi	both sexes combined	7.0	6.0	8.1	1.0
Stroke	Comoros	males	12.7	11.2	14.3	-0.7
Hemorrhagic stroke	Comoros	males	4.4	3.8	5.0	-0.9
Ischemic stroke	Comoros	males	8.8	7.5	10.1	0.2
Stroke	Comoros	females	13.1	11.7	15.0	-1.9
Hemorrhagic stroke	Comoros	females	4.5	3.9	5.3	-1.4
Ischemic stroke	Comoros	females	9.0	7.7	10.5	-0.4
Stroke	Comoros	both sexes combined	12.9	11.7	14.3	-1.4
Hemorrhagic stroke	Comoros	both sexes combined	4.4	3.9	5.0	-1.2
Ischemic stroke	Comoros	both sexes combined	8.9	7.8	10.0	-0.1
Stroke	Djibouti	males	12.4	10.7	14.1	-0.9
Hemorrhagic stroke	Djibouti	males	4.3	3.6	5.0	-0.9
Ischemic stroke	Djibouti	males	8.5	7.2	9.9	0.0
Stroke	Djibouti	females	13.9	11.6	16.6	-1.5
Hemorrhagic stroke	Djibouti	females	4.4	3.6	5.4	-1.2
Ischemic stroke	Djibouti	females	9.9	8.1	12.2	-0.3
Stroke	Djibouti	both sexes combined	13.1	11.4	15.1	-1.2
Hemorrhagic stroke	Djibouti	both sexes combined	4.3	3.7	5.1	-1.0
Ischemic stroke	Djibouti	both sexes combined	9.2	7.8	10.8	-0.2
Stroke	Eritrea	males	10.9	9.4	12.5	1.0
Hemorrhagic stroke	Eritrea	males	4.0	3.3	4.7	-0.1
Ischemic stroke	Eritrea	males	7.2	6.0	8.5	1.3
Stroke	Eritrea	females	11.8	10.4	13.5	-0.6
Hemorrhagic stroke	Eritrea	females	4.2	3.6	4.9	-1.2
Ischemic stroke	Eritrea	females	7.7	6.5	9.1	0.7
Stroke	Eritrea	both sexes combined	11.4	10.1	12.8	0.0
Hemorrhagic stroke	Eritrea	both sexes combined	4.1	3.6	4.7	-0.8
Ischemic stroke	Eritrea	both sexes combined	7.5	6.5	8.6	0.9
Stroke	Ethiopia	males	11.4	9.6	13.4	3.3
Hemorrhagic stroke	Ethiopia	males	4.4	3.6	5.2	0.6
Ischemic stroke	Ethiopia	males	7.3	6.0	8.9	2.8
Stroke	Ethiopia	females	12.3	10.5	14.3	2.7
Hemorrhagic stroke	Ethiopia	females	4.8	4.0	5.7	0.4
Ischemic stroke	Ethiopia	females	7.8	6.5	9.3	2.5
Stroke	Ethiopia	both sexes combined	11.8	10.4	13.4	3.0
Hemorrhagic stroke	Ethiopia	both sexes combined	4.6	4.0	5.2	0.5
Ischemic stroke	Ethiopia	both sexes combined	7.5	6.4	8.8	2.6
Stroke	Kenya	males	11.6	10.6	12.7	0.6
Hemorrhagic stroke	Kenya	males	4.0	3.6	4.5	0.0
Ischemic stroke	Kenya	males	8.0	7.0	8.9	0.6
Stroke	Kenya	females	12.7	11.5	13.9	0.8
Hemorrhagic stroke	Kenya	females	4.2	3.7	4.7	-0.3
Ischemic stroke	Kenya	females	8.8	7.7	9.9	1.1
Stroke	Kenya	both sexes combined	12.1	11.1	13.2	0.7
Hemorrhagic stroke	Kenya	both sexes combined	4.1	3.6	4.6	-0.2
Ischemic stroke	Kenya	both sexes combined	8.4	7.4	9.4	0.9
Stroke	Madagascar	males	14.7	12.3	17.1	-0.8
Hemorrhagic stroke	Madagascar	males	5.8	4.7	6.9	-0.9
Ischemic stroke	Madagascar	males	9.1	7.3	11.0	0.0
Stroke	Madagascar	females	16.4	13.7	20.2	-1.0
Hemorrhagic stroke	Madagascar	females	6.3	5.1	7.7	-1.4
Ischemic stroke	Madagascar	females	10.2	8.1	13.1	0.2
Stroke	Madagascar	both sexes combined	15.5	13.5	17.7	-0.9
Hemorrhagic stroke	Madagascar	both sexes combined	6.0	5.1	7.0	-1.1
Ischemic stroke	Madagascar	both sexes combined	9.7	8.1	11.3	0.1
Stroke	Malawi	males	9.8	8.1	11.5	0.2
Hemorrhagic stroke	Malawi	males	3.1	2.6	3.8	-0.1
Ischemic stroke	Malawi	males	6.9	5.6	8.4	0.2
Stroke	Malawi	females	11.9	9.9	14.7	-0.1
Hemorrhagic stroke	Malawi	females	3.9	3.2	5.0	-0.7
Ischemic stroke	Malawi	females	8.3	6.7	10.4	0.6
Stroke	Malawi	both sexes combined	10.8	9.3	12.5	-0.1

Hemorrhagic stroke	Malawi	both sexes combined	3.5	3.0	4.2		-0.5
Ischemic stroke	Malawi	both sexes combined	7.6	6.4	8.9		0.4
Stroke	Mauritius	males	15.9	14.2	17.8		-2.7
Hemorrhagic stroke	Mauritius	males	6.5	5.6	7.3		-2.5
Ischemic stroke	Mauritius	males	10.2	8.7	11.7		-0.3
Stroke	Mauritius	females	17.6	15.6	19.4		-4.2
Hemorrhagic stroke	Mauritius	females	7.4	6.4	8.4		-2.8
Ischemic stroke	Mauritius	females	11.2	9.6	12.7		-1.7
Stroke	Mauritius	both sexes combined	16.7	15.2	18.3		-3.4
Hemorrhagic stroke	Mauritius	both sexes combined	6.9	6.1	7.8		-2.6
Ischemic stroke	Mauritius	both sexes combined	10.6	9.3	11.9		-0.9
Stroke	Mozambique	males	11.7	10.1	13.4		-1.4
Hemorrhagic stroke	Mozambique	males	4.2	3.5	4.9		-1.2
Ischemic stroke	Mozambique	males	7.8	6.5	9.1		-0.2
Stroke	Mozambique	females	14.1	11.8	16.4		-1.4
Hemorrhagic stroke	Mozambique	females	4.8	4.0	5.8		-1.6
Ischemic stroke	Mozambique	females	9.7	7.9	11.5		0.3
Stroke	Mozambique	both sexes combined	12.9	11.3	14.6		-1.5
Hemorrhagic stroke	Mozambique	both sexes combined	4.5	3.9	5.2		-1.4
Ischemic stroke	Mozambique	both sexes combined	8.8	7.4	10.1		0.0
Stroke	Rwanda	males	11.6	10.1	13.2		2.7
Hemorrhagic stroke	Rwanda	males	3.8	3.3	4.4		0.6
Ischemic stroke	Rwanda	males	8.1	6.8	9.5		2.3
Stroke	Rwanda	females	12.8	11.2	15.0		1.4
Hemorrhagic stroke	Rwanda	females	4.5	3.8	5.3		-0.6
Ischemic stroke	Rwanda	females	8.7	7.2	10.4		2.2
Stroke	Rwanda	both sexes combined	12.3	10.9	13.7		2.0
Hemorrhagic stroke	Rwanda	both sexes combined	4.2	3.6	4.8		0.0
Ischemic stroke	Rwanda	both sexes combined	8.4	7.3	9.7		2.3
Stroke	Seychelles	males	15.1	13.2	16.9		1.1
Hemorrhagic stroke	Seychelles	males	5.4	4.7	6.2		-0.4
Ischemic stroke	Seychelles	males	10.3	8.8	11.9		1.5
Stroke	Seychelles	females	17.3	15.7	18.8		1.1
Hemorrhagic stroke	Seychelles	females	6.7	6.0	7.6		0.1
Ischemic stroke	Seychelles	females	11.5	10.1	12.9		1.2
Stroke	Seychelles	both sexes combined	16.1	14.7	17.6		1.1
Hemorrhagic stroke	Seychelles	both sexes combined	6.1	5.4	6.8		-0.1
Ischemic stroke	Seychelles	both sexes combined	10.9	9.6	12.2		1.3
Stroke	Somalia	males	10.7	9.1	12.1		-1.0
Hemorrhagic stroke	Somalia	males	3.9	3.2	4.5		-0.9
Ischemic stroke	Somalia	males	7.1	5.8	8.2		-0.1
Stroke	Somalia	females	10.8	9.5	12.2		-1.8
Hemorrhagic stroke	Somalia	females	4.1	3.5	4.7		-1.3
Ischemic stroke	Somalia	females	6.9	5.8	7.9		-0.4
Stroke	Somalia	both sexes combined	10.8	9.3	12.1		-1.5
Hemorrhagic stroke	Somalia	both sexes combined	4.0	3.4	4.6		-1.1
Ischemic stroke	Somalia	both sexes combined	7.0	5.9	8.1		-0.3
Stroke	Tanzania	males	11.1	9.6	12.4		0.7
Hemorrhagic stroke	Tanzania	males	3.7	3.1	4.2		0.0
Ischemic stroke	Tanzania	males	7.7	6.6	9.0		0.9
Stroke	Tanzania	females	11.9	10.5	13.8		0.3
Hemorrhagic stroke	Tanzania	females	4.1	3.5	4.9		-0.6
Ischemic stroke	Tanzania	females	8.1	6.8	9.6		0.9
Stroke	Tanzania	both sexes combined	11.5	10.3	12.8		0.5
Hemorrhagic stroke	Tanzania	both sexes combined	3.9	3.4	4.4		-0.3
Ischemic stroke	Tanzania	both sexes combined	7.9	6.9	9.1		0.9
Stroke	Uganda	males	9.8	8.5	11.0		2.0
Hemorrhagic stroke	Uganda	males	3.6	3.1	4.2		0.7
Ischemic stroke	Uganda	males	6.4	5.5	7.5		1.4
Stroke	Uganda	females	11.6	10.2	13.6		0.6
Hemorrhagic stroke	Uganda	females	4.2	3.5	4.9		-0.5
Ischemic stroke	Uganda	females	7.7	6.5	9.3		1.2
Stroke	Uganda	both sexes combined	10.7	9.5	12.0		1.3
Hemorrhagic stroke	Uganda	both sexes combined	3.9	3.4	4.4		0.1
Ischemic stroke	Uganda	both sexes combined	7.1	6.1	8.2		1.3
Stroke	Zambia	males	8.7	7.0	11.2		-0.9
Hemorrhagic stroke	Zambia	males	3.1	2.4	4.0		-0.3
Ischemic stroke	Zambia	males	5.8	4.5	7.6		-0.8
Stroke	Zambia	females	11.4	9.0	14.9		-0.3
Hemorrhagic stroke	Zambia	females	3.8	2.9	5.1		-0.9
Ischemic stroke	Zambia	females	7.9	6.1	10.6		0.5
Stroke	Zambia	both sexes combined	9.9	8.3	11.8		-0.9
Hemorrhagic stroke	Zambia	both sexes combined	3.4	2.8	4.1		-0.7
Ischemic stroke	Zambia	both sexes combined	6.8	5.5	8.3		-0.3
Stroke	Southern Sub-Saharan Africa	males	10.0	9.2	10.9		-2.2
Hemorrhagic stroke	Southern Sub-Saharan Africa	males	3.5	3.1	3.9		-1.0
Ischemic stroke	Southern Sub-Saharan Africa	males	6.8	6.1	7.6		-1.3
Stroke	Southern Sub-Saharan Africa	females	14.9	13.7	16.1		-2.4
Hemorrhagic stroke	Southern Sub-Saharan Africa	females	4.9	4.4	5.4		-1.3
Ischemic stroke	Southern Sub-Saharan Africa	females	10.7	9.6	11.8		-1.2
Stroke	Southern Sub-Saharan Africa	both sexes combined	12.5	11.6	13.5		-2.3

Hemorrhagic stroke	Southern Sub-Saharan Africa	both sexes combined	4.2	3.8	4.6	-1.2
Ischemic stroke	Southern Sub-Saharan Africa	both sexes combined	8.8	7.9	9.7	-1.2
Stroke	Botswana	males	10.7	8.2	14.4	-1.7
Hemorrhagic stroke	Botswana	males	3.6	2.7	5.0	-0.9
Ischemic stroke	Botswana	males	7.4	5.5	10.0	-0.9
Stroke	Botswana	females	16.7	12.0	26.1	-1.5
Hemorrhagic stroke	Botswana	females	5.2	3.5	8.7	-1.4
Ischemic stroke	Botswana	females	12.2	8.6	19.4	-0.1
Stroke	Botswana	both sexes combined	13.4	10.2	18.6	-2.0
Hemorrhagic stroke	Botswana	both sexes combined	4.4	3.2	6.4	-1.3
Ischemic stroke	Botswana	both sexes combined	9.6	7.2	13.6	-0.8
Stroke	Lesotho	males	5.8	4.6	7.3	-4.6
Hemorrhagic stroke	Lesotho	males	2.5	1.9	3.1	-2.4
Ischemic stroke	Lesotho	males	3.4	2.6	4.4	-2.4
Stroke	Lesotho	females	10.3	7.4	14.6	-6.3
Hemorrhagic stroke	Lesotho	females	3.7	2.6	5.4	-3.4
Ischemic stroke	Lesotho	females	6.8	4.7	9.9	-3.1
Stroke	Lesotho	both sexes combined	7.9	6.3	9.7	-5.5
Hemorrhagic stroke	Lesotho	both sexes combined	3.1	2.4	3.8	-2.9
Ischemic stroke	Lesotho	both sexes combined	5.0	3.9	6.4	-2.9
Stroke	Namibia	males	11.3	9.5	13.4	-1.8
Hemorrhagic stroke	Namibia	males	4.0	3.3	4.9	-1.1
Ischemic stroke	Namibia	males	7.6	6.3	9.3	-0.8
Stroke	Namibia	females	17.7	13.9	24.0	-2.2
Hemorrhagic stroke	Namibia	females	5.8	4.2	8.3	-1.9
Ischemic stroke	Namibia	females	12.7	9.7	17.6	-0.3
Stroke	Namibia	both sexes combined	14.4	12.0	17.7	-2.1
Hemorrhagic stroke	Namibia	both sexes combined	4.9	3.9	6.2	-1.5
Ischemic stroke	Namibia	both sexes combined	10.0	8.2	12.7	-0.6
Stroke	South Africa	males	10.6	9.8	11.5	-2.3
Hemorrhagic stroke	South Africa	males	3.7	3.3	4.0	-1.2
Ischemic stroke	South Africa	males	7.3	6.5	8.1	-1.3
Stroke	South Africa	females	15.6	14.3	16.8	-2.4
Hemorrhagic stroke	South Africa	females	5.1	4.6	5.6	-1.4
Ischemic stroke	South Africa	females	11.2	10.1	12.5	-1.1
Stroke	South Africa	both sexes combined	13.2	12.3	14.2	-2.3
Hemorrhagic stroke	South Africa	both sexes combined	4.4	4.0	4.8	-1.3
Ischemic stroke	South Africa	both sexes combined	9.4	8.4	10.3	-1.1
Stroke	Swaziland	males	8.2	6.3	11.0	-3.1
Hemorrhagic stroke	Swaziland	males	2.9	2.2	4.1	-1.6
Ischemic stroke	Swaziland	males	5.5	4.1	7.5	-1.6
Stroke	Swaziland	females	14.4	10.0	20.4	-2.9
Hemorrhagic stroke	Swaziland	females	4.5	2.9	6.7	-2.2
Ischemic stroke	Swaziland	females	10.5	7.1	15.2	-0.7
Stroke	Swaziland	both sexes combined	11.0	8.3	14.7	-3.4
Hemorrhagic stroke	Swaziland	both sexes combined	3.6	2.6	5.0	-2.0
Ischemic stroke	Swaziland	both sexes combined	7.7	5.6	10.5	-1.5
Stroke	Zimbabwe	males	8.0	6.6	9.8	-1.4
Hemorrhagic stroke	Zimbabwe	males	2.8	2.4	3.5	-0.1
Ischemic stroke	Zimbabwe	males	5.4	4.3	6.8	-1.4
Stroke	Zimbabwe	females	11.7	9.6	14.5	-2.6
Hemorrhagic stroke	Zimbabwe	females	3.9	3.1	4.9	-0.5
Ischemic stroke	Zimbabwe	females	8.2	6.5	10.3	-2.1
Stroke	Zimbabwe	both sexes combined	9.8	8.3	11.5	-2.1
Hemorrhagic stroke	Zimbabwe	both sexes combined	3.3	2.8	4.0	-0.3
Ischemic stroke	Zimbabwe	both sexes combined	6.7	5.5	8.1	-1.8
Stroke	Western Sub-Saharan Africa	males	13.0	11.9	14.2	1.2
Hemorrhagic stroke	Western Sub-Saharan Africa	males	4.4	3.9	4.9	0.2
Ischemic stroke	Western Sub-Saharan Africa	males	9.0	8.0	10.1	1.1
Stroke	Western Sub-Saharan Africa	females	15.8	14.5	17.3	1.0
Hemorrhagic stroke	Western Sub-Saharan Africa	females	5.0	4.4	5.6	0.1
Ischemic stroke	Western Sub-Saharan Africa	females	11.4	10.1	12.7	1.0
Stroke	Western Sub-Saharan Africa	both sexes combined	14.4	13.3	15.7	1.0
Hemorrhagic stroke	Western Sub-Saharan Africa	both sexes combined	4.7	4.2	5.2	0.2
Ischemic stroke	Western Sub-Saharan Africa	both sexes combined	10.2	9.1	11.4	1.0
Stroke	Benin	males	13.1	11.9	14.3	0.4
Hemorrhagic stroke	Benin	males	4.8	4.3	5.3	0.2
Ischemic stroke	Benin	males	8.7	7.6	9.8	0.3
Stroke	Benin	females	15.8	14.0	17.8	0.8
Hemorrhagic stroke	Benin	females	5.3	4.6	6.2	0.3
Ischemic stroke	Benin	females	11.0	9.4	12.6	0.5
Stroke	Benin	both sexes combined	14.5	13.3	15.8	0.6
Hemorrhagic stroke	Benin	both sexes combined	5.1	4.5	5.6	0.2
Ischemic stroke	Benin	both sexes combined	9.9	8.8	11.1	0.4
Stroke	Burkina Faso	males	11.1	10.2	12.1	2.7
Hemorrhagic stroke	Burkina Faso	males	4.2	3.7	4.7	1.0
Ischemic stroke	Burkina Faso	males	7.3	6.4	8.1	1.8
Stroke	Burkina Faso	females	12.5	11.4	13.7	2.6
Hemorrhagic stroke	Burkina Faso	females	4.3	3.8	4.8	0.9
Ischemic stroke	Burkina Faso	females	8.6	7.5	9.7	1.9
Stroke	Burkina Faso	both sexes combined	11.9	10.9	13.0	2.7

Hemorrhagic stroke	Burkina Faso	both sexes combined	4.2	3.8	4.8	0.9
Ischemic stroke	Burkina Faso	both sexes combined	8.1	7.0	9.0	1.9
Stroke	Cameroon	males	11.0	9.7	12.4	-1.1
Hemorrhagic stroke	Cameroon	males	4.1	3.5	4.7	-0.7
Ischemic stroke	Cameroon	males	7.3	6.2	8.4	-0.5
Stroke	Cameroon	females	13.4	11.0	16.1	-0.8
Hemorrhagic stroke	Cameroon	females	4.5	3.6	5.6	-0.4
Ischemic stroke	Cameroon	females	9.3	7.4	11.5	-0.5
Stroke	Cameroon	both sexes combined	12.2	10.6	13.8	-1.1
Hemorrhagic stroke	Cameroon	both sexes combined	4.3	3.6	5.0	-0.6
Ischemic stroke	Cameroon	both sexes combined	8.3	7.0	9.6	-0.6
Stroke	Cape Verde	males	14.8	12.9	16.8	-0.7
Hemorrhagic stroke	Cape Verde	males	5.5	4.7	6.3	-0.8
Ischemic stroke	Cape Verde	males	9.9	8.4	11.5	0.1
Stroke	Cape Verde	females	19.8	18.0	22.1	-0.8
Hemorrhagic stroke	Cape Verde	females	6.3	5.5	7.2	-1.0
Ischemic stroke	Cape Verde	females	14.5	12.8	16.6	0.3
Stroke	Cape Verde	both sexes combined	17.2	15.7	19.0	-0.8
Hemorrhagic stroke	Cape Verde	both sexes combined	5.8	5.1	6.6	-0.9
Ischemic stroke	Cape Verde	both sexes combined	12.1	10.7	13.8	0.1
Stroke	Chad	males	12.4	11.2	13.8	0.4
Hemorrhagic stroke	Chad	males	4.4	3.9	5.1	0.1
Ischemic stroke	Chad	males	8.4	7.3	9.6	0.3
Stroke	Chad	females	14.7	13.0	16.4	0.7
Hemorrhagic stroke	Chad	females	4.8	4.2	5.5	0.1
Ischemic stroke	Chad	females	10.4	8.9	12.0	0.6
Stroke	Chad	both sexes combined	13.6	12.3	14.8	0.4
Hemorrhagic stroke	Chad	both sexes combined	4.6	4.1	5.2	0.1
Ischemic stroke	Chad	both sexes combined	9.4	8.3	10.5	0.4
Stroke	Cote d'Ivoire	males	12.1	10.7	13.5	0.1
Hemorrhagic stroke	Cote d'Ivoire	males	4.6	4.0	5.2	-0.1
Ischemic stroke	Cote d'Ivoire	males	7.9	6.8	9.0	0.1
Stroke	Cote d'Ivoire	females	14.8	13.1	16.6	-0.3
Hemorrhagic stroke	Cote d'Ivoire	females	5.0	4.2	5.8	-0.1
Ischemic stroke	Cote d'Ivoire	females	10.2	8.8	11.8	-0.3
Stroke	Cote d'Ivoire	both sexes combined	13.3	12.0	14.6	-0.1
Hemorrhagic stroke	Cote d'Ivoire	both sexes combined	4.8	4.2	5.3	-0.1
Ischemic stroke	Cote d'Ivoire	both sexes combined	8.9	7.8	10.0	-0.1
Stroke	The Gambia	males	13.8	12.4	15.3	0.0
Hemorrhagic stroke	The Gambia	males	4.9	4.2	5.5	0.2
Ischemic stroke	The Gambia	males	9.5	8.3	10.8	-0.1
Stroke	The Gambia	females	16.8	14.8	19.2	1.2
Hemorrhagic stroke	The Gambia	females	5.4	4.7	6.4	0.3
Ischemic stroke	The Gambia	females	12.0	10.3	14.0	0.9
Stroke	The Gambia	both sexes combined	15.2	13.8	16.8	0.5
Hemorrhagic stroke	The Gambia	both sexes combined	5.1	4.5	5.8	0.2
Ischemic stroke	The Gambia	both sexes combined	10.7	9.4	12.0	0.4
Stroke	Ghana	males	12.4	11.1	13.9	2.0
Hemorrhagic stroke	Ghana	males	4.0	3.5	4.6	0.3
Ischemic stroke	Ghana	males	8.8	7.6	10.1	1.8
Stroke	Ghana	females	19.5	17.5	22.1	0.0
Hemorrhagic stroke	Ghana	females	6.2	5.4	7.2	-0.8
Ischemic stroke	Ghana	females	13.8	12.0	15.9	0.8
Stroke	Ghana	both sexes combined	16.2	14.7	17.8	1.1
Hemorrhagic stroke	Ghana	both sexes combined	5.2	4.6	5.9	-0.2
Ischemic stroke	Ghana	both sexes combined	11.5	10.1	13.0	1.3
Stroke	Guinea	males	12.5	11.1	14.0	-0.4
Hemorrhagic stroke	Guinea	males	4.5	3.9	5.2	0.0
Ischemic stroke	Guinea	males	8.3	7.1	9.5	-0.4
Stroke	Guinea	females	14.5	12.5	16.8	0.3
Hemorrhagic stroke	Guinea	females	4.8	4.0	5.7	0.0
Ischemic stroke	Guinea	females	10.0	8.4	11.9	0.2
Stroke	Guinea	both sexes combined	13.5	12.1	14.9	-0.2
Hemorrhagic stroke	Guinea	both sexes combined	4.7	4.1	5.3	0.0
Ischemic stroke	Guinea	both sexes combined	9.2	8.0	10.4	-0.2
Stroke	Guinea-Bissau	males	11.0	9.6	12.6	-0.2
Hemorrhagic stroke	Guinea-Bissau	males	4.3	3.6	4.9	-0.1
Ischemic stroke	Guinea-Bissau	males	6.9	5.9	8.2	-0.1
Stroke	Guinea-Bissau	females	14.1	12.3	16.0	0.0
Hemorrhagic stroke	Guinea-Bissau	females	4.8	4.0	5.5	-0.1
Ischemic stroke	Guinea-Bissau	females	9.7	8.2	11.3	0.2
Stroke	Guinea-Bissau	both sexes combined	12.5	11.2	13.8	-0.2
Hemorrhagic stroke	Guinea-Bissau	both sexes combined	4.5	3.9	5.1	-0.1
Ischemic stroke	Guinea-Bissau	both sexes combined	8.2	7.1	9.3	-0.1
Stroke	Liberia	males	13.2	12.0	14.5	0.5
Hemorrhagic stroke	Liberia	males	4.7	4.2	5.3	0.1
Ischemic stroke	Liberia	males	8.9	7.8	10.0	0.4
Stroke	Liberia	females	14.5	13.1	16.1	0.7
Hemorrhagic stroke	Liberia	females	5.0	4.5	5.6	0.2
Ischemic stroke	Liberia	females	9.9	8.7	11.2	0.5
Stroke	Liberia	both sexes combined	13.9	12.8	15.1	0.6

Hemorrhagic stroke	Liberia	both sexes combined	4.9	4.4	5.4	0.1
Ischemic stroke	Liberia	both sexes combined	9.5	8.4	10.5	0.5
Stroke	Mali	males	13.3	11.2	15.4	2.2
Hemorrhagic stroke	Mali	males	4.6	3.9	5.5	0.5
Ischemic stroke	Mali	males	9.1	7.5	10.8	1.8
Stroke	Mali	females	15.5	12.9	17.9	1.7
Hemorrhagic stroke	Mali	females	5.2	4.2	6.2	0.4
Ischemic stroke	Mali	females	10.8	8.7	12.8	1.5
Stroke	Mali	both sexes combined	14.4	12.6	16.3	1.8
Hemorrhagic stroke	Mali	both sexes combined	4.9	4.2	5.7	0.4
Ischemic stroke	Mali	both sexes combined	10.0	8.5	11.5	1.6
Stroke	Mauritania	males	15.6	13.4	18.3	1.9
Hemorrhagic stroke	Mauritania	males	4.8	4.0	5.7	0.5
Ischemic stroke	Mauritania	males	11.5	9.6	13.7	1.6
Stroke	Mauritania	females	17.5	14.8	20.7	1.1
Hemorrhagic stroke	Mauritania	females	5.0	4.1	6.1	0.5
Ischemic stroke	Mauritania	females	13.2	10.8	16.0	0.9
Stroke	Mauritania	both sexes combined	16.7	14.8	18.9	1.5
Hemorrhagic stroke	Mauritania	both sexes combined	4.9	4.2	5.7	0.5
Ischemic stroke	Mauritania	both sexes combined	12.5	10.7	14.5	1.2
Stroke	Niger	males	13.0	11.4	14.9	1.4
Hemorrhagic stroke	Niger	males	4.5	3.8	5.2	0.2
Ischemic stroke	Niger	males	8.9	7.5	10.5	1.2
Stroke	Niger	females	15.5	12.9	18.0	1.1
Hemorrhagic stroke	Niger	females	5.1	4.2	6.0	0.4
Ischemic stroke	Niger	females	10.8	8.8	13.0	0.8
Stroke	Niger	both sexes combined	14.2	12.6	15.9	1.1
Hemorrhagic stroke	Niger	both sexes combined	4.8	4.2	5.5	0.3
Ischemic stroke	Niger	both sexes combined	9.9	8.4	11.4	0.9
Stroke	Nigeria	males	13.7	11.8	15.6	1.8
Hemorrhagic stroke	Nigeria	males	4.3	3.6	5.0	0.3
Ischemic stroke	Nigeria	males	9.9	8.3	11.6	1.7
Stroke	Nigeria	females	16.2	14.0	18.9	1.3
Hemorrhagic stroke	Nigeria	females	4.9	4.1	5.9	0.1
Ischemic stroke	Nigeria	females	12.0	10.2	14.3	1.4
Stroke	Nigeria	both sexes combined	14.9	13.2	16.8	1.4
Hemorrhagic stroke	Nigeria	both sexes combined	4.6	4.0	5.3	0.2
Ischemic stroke	Nigeria	both sexes combined	11.0	9.4	12.7	1.4
Stroke	Sao Tome and Principe	males	16.3	14.0	18.9	0.9
Hemorrhagic stroke	Sao Tome and Principe	males	5.6	4.7	6.5	0.0
Ischemic stroke	Sao Tome and Principe	males	11.4	9.5	13.6	0.9
Stroke	Sao Tome and Principe	females	20.0	18.1	22.1	-0.2
Hemorrhagic stroke	Sao Tome and Principe	females	6.3	5.5	7.2	-0.3
Ischemic stroke	Sao Tome and Principe	females	14.4	12.6	16.2	0.0
Stroke	Sao Tome and Principe	both sexes combined	18.3	16.5	20.2	0.3
Hemorrhagic stroke	Sao Tome and Principe	both sexes combined	6.0	5.2	6.8	-0.2
Ischemic stroke	Sao Tome and Principe	both sexes combined	13.0	11.4	14.6	0.4
Stroke	Senegal	males	13.0	11.8	14.4	1.3
Hemorrhagic stroke	Senegal	males	4.6	4.1	5.2	0.5
Ischemic stroke	Senegal	males	8.8	7.7	10.0	0.9
Stroke	Senegal	females	16.5	15.0	18.0	1.2
Hemorrhagic stroke	Senegal	females	5.3	4.7	5.9	0.5
Ischemic stroke	Senegal	females	11.7	10.3	13.1	0.8
Stroke	Senegal	both sexes combined	14.9	13.7	16.2	1.4
Hemorrhagic stroke	Senegal	both sexes combined	5.0	4.5	5.5	0.5
Ischemic stroke	Senegal	both sexes combined	10.4	9.2	11.6	0.9
Stroke	Sierra Leone	males	13.0	11.7	14.5	0.4
Hemorrhagic stroke	Sierra Leone	males	4.6	4.1	5.2	0.2
Ischemic stroke	Sierra Leone	males	8.8	7.7	10.1	0.2
Stroke	Sierra Leone	females	15.0	13.2	17.0	0.4
Hemorrhagic stroke	Sierra Leone	females	5.1	4.3	5.9	0.3
Ischemic stroke	Sierra Leone	females	10.4	8.8	12.2	0.2
Stroke	Sierra Leone	both sexes combined	14.0	12.7	15.4	0.5
Hemorrhagic stroke	Sierra Leone	both sexes combined	4.8	4.3	5.4	0.3
Ischemic stroke	Sierra Leone	both sexes combined	9.6	8.4	10.8	0.3
Stroke	Togo	males	12.4	10.9	13.8	-0.6
Hemorrhagic stroke	Togo	males	4.4	3.7	5.0	-0.2
Ischemic stroke	Togo	males	8.4	7.2	9.6	-0.5
Stroke	Togo	females	15.2	13.6	17.3	0.1
Hemorrhagic stroke	Togo	females	5.0	4.3	5.8	-0.1
Ischemic stroke	Togo	females	10.7	9.2	12.5	0.2
Stroke	Togo	both sexes combined	13.8	12.5	15.2	-0.3
Hemorrhagic stroke	Togo	both sexes combined	4.7	4.1	5.2	-0.2
Ischemic stroke	Togo	both sexes combined	9.5	8.4	10.9	-0.2
Stroke	American Samoa	males	18.9	16.5	21.5	-1.5
Hemorrhagic stroke	American Samoa	males	7.1	6.0	8.2	-1.4
Ischemic stroke	American Samoa	males	12.9	11.0	15.1	-0.2
Stroke	American Samoa	females	20.4	17.8	23.1	-1.8
Hemorrhagic stroke	American Samoa	females	8.7	7.4	10.2	-1.2
Ischemic stroke	American Samoa	females	13.1	11.1	15.1	-0.8
Stroke	American Samoa	both sexes combined	19.7	17.7	21.9	-1.5

Hemorrhagic stroke	American Samoa	both sexes combined	8.0	7.0	9.1	-1.0
Ischemic stroke	American Samoa	both sexes combined	12.9	11.2	14.7	-0.5
Stroke	Bermuda	males	16.7	14.8	18.5	2.2
Hemorrhagic stroke	Bermuda	males	5.7	5.0	6.5	1.0
Ischemic stroke	Bermuda	males	11.9	10.3	13.4	1.4
Stroke	Bermuda	females	20.3	17.8	23.1	0.1
Hemorrhagic stroke	Bermuda	females	6.7	5.7	7.8	0.2
Ischemic stroke	Bermuda	females	14.7	12.6	17.1	-0.1
Stroke	Bermuda	both sexes combined	18.3	16.5	20.2	1.2
Hemorrhagic stroke	Bermuda	both sexes combined	6.1	5.4	6.9	0.6
Ischemic stroke	Bermuda	both sexes combined	13.2	11.6	14.8	0.7
Stroke	Greenland	males	21.1	17.6	24.6	2.5
Hemorrhagic stroke	Greenland	males	7.9	6.4	9.4	0.7
Ischemic stroke	Greenland	males	14.9	12.1	17.9	2.0
Stroke	Greenland	females	21.4	18.0	24.9	-1.4
Hemorrhagic stroke	Greenland	females	7.3	5.9	8.8	-1.7
Ischemic stroke	Greenland	females	15.4	12.7	18.5	-0.1
Stroke	Greenland	both sexes combined	21.2	18.6	23.9	0.8
Hemorrhagic stroke	Greenland	both sexes combined	7.6	6.5	8.8	-0.3
Ischemic stroke	Greenland	both sexes combined	15.1	12.8	17.5	1.1
Stroke	Guam	males	18.1	16.1	20.4	-0.9
Hemorrhagic stroke	Guam	males	6.3	5.4	7.1	-0.2
Ischemic stroke	Guam	males	12.8	11.0	14.7	-0.6
Stroke	Guam	females	21.2	19.1	23.3	0.1
Hemorrhagic stroke	Guam	females	8.1	7.0	9.2	0.0
Ischemic stroke	Guam	females	14.5	12.6	16.4	0.2
Stroke	Guam	both sexes combined	19.5	17.8	21.1	-0.4
Hemorrhagic stroke	Guam	both sexes combined	7.1	6.3	7.9	-0.2
Ischemic stroke	Guam	both sexes combined	13.6	12.0	15.2	-0.2
Stroke	Northern Mariana Islands	males	21.8	19.4	24.2	-2.2
Hemorrhagic stroke	Northern Mariana Islands	males	8.8	7.6	10.0	-1.2
Ischemic stroke	Northern Mariana Islands	males	14.4	12.4	16.4	-1.3
Stroke	Northern Mariana Islands	females	20.9	18.3	23.7	-2.4
Hemorrhagic stroke	Northern Mariana Islands	females	9.5	8.0	11.2	-1.8
Ischemic stroke	Northern Mariana Islands	females	12.8	10.8	14.8	-0.7
Stroke	Northern Mariana Islands	both sexes combined	21.3	19.2	23.2	-2.3
Hemorrhagic stroke	Northern Mariana Islands	both sexes combined	9.2	8.1	10.4	-1.5
Ischemic stroke	Northern Mariana Islands	both sexes combined	13.5	11.8	15.0	-1.0
Stroke	Puerto Rico	males	15.8	14.3	17.3	2.6
Hemorrhagic stroke	Puerto Rico	males	5.6	4.9	6.2	1.3
Ischemic stroke	Puerto Rico	males	11.1	9.7	12.5	1.5
Stroke	Puerto Rico	females	20.0	18.3	21.8	2.9
Hemorrhagic stroke	Puerto Rico	females	7.0	6.2	7.9	1.6
Ischemic stroke	Puerto Rico	females	14.2	12.5	15.9	1.6
Stroke	Puerto Rico	both sexes combined	18.0	16.5	19.5	2.9
Hemorrhagic stroke	Puerto Rico	both sexes combined	6.3	5.7	7.0	1.5
Ischemic stroke	Puerto Rico	both sexes combined	12.7	11.3	14.1	1.7
Stroke	Virgin Islands, U.S.	males	15.1	13.2	17.1	-0.5
Hemorrhagic stroke	Virgin Islands, U.S.	males	5.4	4.6	6.2	-0.2
Ischemic stroke	Virgin Islands, U.S.	males	10.4	9.0	12.2	-0.3
Stroke	Virgin Islands, U.S.	females	19.3	17.6	21.2	0.5
Hemorrhagic stroke	Virgin Islands, U.S.	females	6.8	6.0	7.6	0.0
Ischemic stroke	Virgin Islands, U.S.	females	13.7	12.0	15.4	0.6
Stroke	Virgin Islands, U.S.	both sexes combined	17.1	15.5	18.8	-0.1
Hemorrhagic stroke	Virgin Islands, U.S.	both sexes combined	6.0	5.3	6.8	-0.1
Ischemic stroke	Virgin Islands, U.S.	both sexes combined	12.0	10.5	13.6	0.1
Stroke	South Sudan	males	11.2	9.5	13.0	-0.9
Hemorrhagic stroke	South Sudan	males	3.9	3.2	4.6	-0.6
Ischemic stroke	South Sudan	males	7.6	6.3	9.1	-0.4
Stroke	South Sudan	females	12.0	9.8	14.5	-1.7
Hemorrhagic stroke	South Sudan	females	4.2	3.4	5.2	-1.1
Ischemic stroke	South Sudan	females	8.1	6.4	10.0	-0.6
Stroke	South Sudan	both sexes combined	11.6	9.7	13.7	-1.4
Hemorrhagic stroke	South Sudan	both sexes combined	4.1	3.3	4.9	-0.9
Ischemic stroke	South Sudan	both sexes combined	7.8	6.4	9.6	-0.5
Stroke	Sudan	males	19.5	17.8	21.1	2.8
Hemorrhagic stroke	Sudan	males	5.6	5.0	6.3	0.2
Ischemic stroke	Sudan	males	14.8	13.1	16.3	2.7
Stroke	Sudan	females	23.0	20.9	25.0	2.9
Hemorrhagic stroke	Sudan	females	6.1	5.4	6.9	0.2
Ischemic stroke	Sudan	females	18.0	16.0	19.9	3.0
Stroke	Sudan	both sexes combined	21.2	19.6	22.8	2.8
Hemorrhagic stroke	Sudan	both sexes combined	5.9	5.2	6.5	0.2
Ischemic stroke	Sudan	both sexes combined	16.4	14.7	18.0	2.8
Stroke	High-middle SDI	males	29.5	27.4	31.5	2.8
Hemorrhagic stroke	High-middle SDI	males	8.4	7.6	9.2	0.2
Ischemic stroke	High-middle SDI	males	23.4	21.3	25.5	3.0
Stroke	High-middle SDI	females	32.8	30.2	35.0	-1.7
Hemorrhagic stroke	High-middle SDI	females	8.9	8.0	9.8	-0.8
Ischemic stroke	High-middle SDI	females	26.5	24.0	28.8	-1.0
Stroke	High-middle SDI	both sexes combined	31.1	29.0	33.0	0.4

Hemorrhagic stroke	High-middle SDI	both sexes combined	8.6	7.8	9.4	-0.3
Ischemic stroke	High-middle SDI	both sexes combined	24.9	22.8	26.9	0.9
Stroke	High SDI	males	22.6	21.3	23.8	0.8
Hemorrhagic stroke	High SDI	males	7.4	6.8	7.9	0.6
Ischemic stroke	High SDI	males	17.0	15.6	18.3	0.4
Stroke	High SDI	females	24.5	23.1	25.8	-0.7
Hemorrhagic stroke	High SDI	females	7.3	6.7	7.9	-0.2
Ischemic stroke	High SDI	females	18.9	17.5	20.3	-0.4
Stroke	High SDI	both sexes combined	23.5	22.2	24.8	0.1
Hemorrhagic stroke	High SDI	both sexes combined	7.3	6.7	7.9	0.2
Ischemic stroke	High SDI	both sexes combined	17.9	16.5	19.3	0.0
Stroke	Low-middle SDI	males	16.0	14.9	17.1	2.4
Hemorrhagic stroke	Low-middle SDI	males	5.4	4.9	5.9	0.3
Ischemic stroke	Low-middle SDI	males	11.1	10.0	12.2	2.3
Stroke	Low-middle SDI	females	17.6	16.4	18.9	2.8
Hemorrhagic stroke	Low-middle SDI	females	5.9	5.3	6.4	0.4
Ischemic stroke	Low-middle SDI	females	12.5	11.2	13.7	2.7
Stroke	Low-middle SDI	both sexes combined	16.8	15.6	17.9	2.6
Hemorrhagic stroke	Low-middle SDI	both sexes combined	5.6	5.1	6.2	0.3
Ischemic stroke	Low-middle SDI	both sexes combined	11.8	10.6	12.9	2.4
Stroke	Low SDI	males	12.4	11.4	13.4	1.7
Hemorrhagic stroke	Low SDI	males	4.3	3.9	4.8	0.2
Ischemic stroke	Low SDI	males	8.4	7.5	9.3	1.6
Stroke	Low SDI	females	14.0	13.0	15.1	1.2
Hemorrhagic stroke	Low SDI	females	4.8	4.3	5.3	-0.1
Ischemic stroke	Low SDI	females	9.5	8.5	10.6	1.4
Stroke	Low SDI	both sexes combined	13.2	12.3	14.2	1.4
Hemorrhagic stroke	Low SDI	both sexes combined	4.6	4.1	5.1	0.1
Ischemic stroke	Low SDI	both sexes combined	9.0	8.0	10.0	1.5
Stroke	Middle SDI	males	30.6	29.0	32.2	6.1
Hemorrhagic stroke	Middle SDI	males	11.0	10.2	11.8	0.7
Ischemic stroke	Middle SDI	males	22.2	20.4	23.9	6.5
Stroke	Middle SDI	females	27.6	26.2	29.2	2.9
Hemorrhagic stroke	Middle SDI	females	11.3	10.4	12.2	0.3
Ischemic stroke	Middle SDI	females	18.5	17.0	20.1	3.2
Stroke	Middle SDI	both sexes combined	29.3	27.8	30.8	4.7
Hemorrhagic stroke	Middle SDI	both sexes combined	11.1	10.3	11.9	0.5
Ischemic stroke	Middle SDI	both sexes combined	20.6	18.9	22.1	5.0

Table S5. Lifetime risk of stroke counterfactual, 2016								
Cause	Location	Sex	Mean lifetime risk (%)	95% UI Lower	95% UI Upper	Mean lifetime risk counterfactual (%)	95% UI Lower	95% UI Upper
Cerebrovascular disease	East Asia	males	40.6	38.7	42.3	44.2	42.1	46.0
Hemorrhagic stroke	East Asia	males	14.9	13.9	16.1	17.1	16.0	18.3
Ischemic stroke	East Asia	males	30.5	28.4	32.6	35.0	32.5	37.2
Cerebrovascular disease	East Asia	females	36.3	34.5	38.1	38.6	36.8	40.4
Hemorrhagic stroke	East Asia	females	15.8	14.6	17.0	17.6	16.3	18.9
Ischemic stroke	East Asia	females	24.6	22.6	26.4	27.0	25.0	28.9
Cerebrovascular disease	East Asia	both sexes combined	38.8	37.0	40.6	41.9	40.1	43.7
Hemorrhagic stroke	East Asia	both sexes combined	15.3	14.2	16.4	17.5	16.3	18.7
Ischemic stroke	East Asia	both sexes combined	28.0	26.0	30.0	31.6	29.4	33.7
Cerebrovascular disease	Southeast Asia	males	19.6	18.3	20.9	23.9	22.4	25.3
Hemorrhagic stroke	Southeast Asia	males	7.9	7.2	8.7	9.9	9.1	10.8
Ischemic stroke	Southeast Asia	males	12.4	11.1	13.7	16.1	14.5	17.6
Cerebrovascular disease	Southeast Asia	females	20.0	18.8	21.4	25.4	23.9	27.0
Hemorrhagic stroke	Southeast Asia	females	8.2	7.5	8.9	11.0	10.0	11.9
Ischemic stroke	Southeast Asia	females	12.8	11.6	14.1	16.8	15.3	18.3
Cerebrovascular disease	Southeast Asia	both sexes combined	19.8	18.6	21.1	24.6	23.2	26.1
Hemorrhagic stroke	Southeast Asia	both sexes combined	8.1	7.4	8.8	10.4	9.6	11.3
Ischemic stroke	Southeast Asia	both sexes combined	12.6	11.4	13.8	16.4	14.9	17.9
Cerebrovascular disease	Oceania	males	15.5	13.8	17.2	27.4	25.7	29.1
Hemorrhagic stroke	Oceania	males	5.9	5.1	6.7	10.8	9.9	11.8
Ischemic stroke	Oceania	males	10.3	8.9	11.8	19.4	17.5	21.2
Cerebrovascular disease	Oceania	females	16.5	14.6	18.3	32.4	30.7	34.3
Hemorrhagic stroke	Oceania	females	7.3	6.3	8.3	15.9	14.5	17.3
Ischemic stroke	Oceania	females	9.9	8.5	11.4	20.7	18.8	22.6
Cerebrovascular disease	Oceania	both sexes combined	16.0	14.2	17.6	29.9	28.3	31.6
Hemorrhagic stroke	Oceania	both sexes combined	6.6	5.8	7.5	13.5	12.3	14.6
Ischemic stroke	Oceania	both sexes combined	10.1	8.7	11.5	20.0	18.1	21.8
Cerebrovascular disease	Central Asia	males	22.7	21.1	24.4	31.4	29.7	33.0
Hemorrhagic stroke	Central Asia	males	9.1	8.3	10.0	12.9	11.9	14.0
Ischemic stroke	Central Asia	males	15.2	13.6	16.7	22.5	20.6	24.2
Cerebrovascular disease	Central Asia	females	26.1	24.4	27.9	32.7	30.9	34.4
Hemorrhagic stroke	Central Asia	females	9.7	8.9	10.7	12.9	11.8	13.9
Ischemic stroke	Central Asia	females	18.1	16.4	19.8	23.7	21.6	25.6
Cerebrovascular disease	Central Asia	both sexes combined	24.4	22.8	25.9	31.8	30.1	33.5
Hemorrhagic stroke	Central Asia	both sexes combined	9.4	8.6	10.3	12.8	11.8	13.8
Ischemic stroke	Central Asia	both sexes combined	16.6	15.1	18.1	22.9	21.0	24.7
Cerebrovascular disease	Central Europe	males	29.8	28.0	31.5	35.6	33.6	37.3
Hemorrhagic stroke	Central Europe	males	7.6	7.1	8.1	9.3	8.8	9.9
Ischemic stroke	Central Europe	males	24.5	22.6	26.3	30.0	27.8	32.0
Cerebrovascular disease	Central Europe	females	33.7	31.8	35.5	37.5	35.5	39.2
Hemorrhagic stroke	Central Europe	females	7.5	7.0	7.9	8.6	8.1	9.1
Ischemic stroke	Central Europe	females	28.5	26.4	30.4	32.3	30.2	34.3
Cerebrovascular disease	Central Europe	both sexes combined	31.7	30.0	33.3	36.3	34.5	38.0
Hemorrhagic stroke	Central Europe	both sexes combined	7.5	7.0	8.0	8.9	8.4	9.4
Ischemic stroke	Central Europe	both sexes combined	26.5	24.6	28.2	31.0	29.0	32.9
Cerebrovascular disease	Eastern Europe	males	26.8	22.0	31.6	40.7	38.1	43.2
Hemorrhagic stroke	Eastern Europe	males	6.4	5.2	7.7	10.0	9.1	11.0
Ischemic stroke	Eastern Europe	males	22.1	17.6	26.8	35.2	32.2	38.0
Cerebrovascular disease	Eastern Europe	females	36.5	31.2	41.9	44.0	41.2	46.7
Hemorrhagic stroke	Eastern Europe	females	7.5	6.2	8.8	9.7	8.8	10.7
Ischemic stroke	Eastern Europe	females	31.5	26.3	36.7	39.0	35.6	41.9
Cerebrovascular disease	Eastern Europe	both sexes combined	31.6	27.6	35.6	42.0	39.2	44.4
Hemorrhagic stroke	Eastern Europe	both sexes combined	6.9	6.0	7.9	9.7	8.8	10.6
Ischemic stroke	Eastern Europe	both sexes combined	26.8	23.0	30.6	36.7	33.8	39.5
Cerebrovascular disease	High-income Asia Pacific	males	22.2	20.6	23.8	20.4	19.1	21.7
Hemorrhagic stroke	High-income Asia Pacific	males	7.5	6.8	8.2	6.9	6.3	7.5
Ischemic stroke	High-income Asia Pacific	males	16.2	14.6	17.8	14.9	13.6	16.4
Cerebrovascular disease	High-income Asia Pacific	females	23.5	21.8	25.1	20.7	19.3	22.0
Hemorrhagic stroke	High-income Asia Pacific	females	7.6	6.8	8.4	6.7	6.0	7.3
Ischemic stroke	High-income Asia Pacific	females	17.4	15.8	19.2	15.2	13.8	16.6
Cerebrovascular disease	High-income Asia Pacific	both sexes combined	22.8	21.2	24.3	20.5	19.2	21.7
Hemorrhagic stroke	High-income Asia Pacific	both sexes combined	7.5	6.8	8.2	6.7	6.1	7.3
Ischemic stroke	High-income Asia Pacific	both sexes combined	16.8	15.2	18.3	15.0	13.6	16.4
Cerebrovascular disease	Australasia	males	20.9	19.4	22.4	19.0	17.8	20.2
Hemorrhagic stroke	Australasia	males	7.5	6.8	8.3	6.7	6.1	7.3
Ischemic stroke	Australasia	males	15.0	13.5	16.4	13.6	12.4	14.9
Cerebrovascular disease	Australasia	females	23.0	21.5	24.7	22.0	20.8	23.5
Hemorrhagic stroke	Australasia	females	8.7	7.9	9.6	8.3	7.6	9.1
Ischemic stroke	Australasia	females	16.1	14.7	17.8	15.5	14.2	17.0
Cerebrovascular disease	Australasia	both sexes combined	21.9	20.6	23.4	20.6	19.4	21.8
Hemorrhagic stroke	Australasia	both sexes combined	8.1	7.4	8.9	7.5	6.9	8.2
Ischemic stroke	Australasia	both sexes combined	15.6	14.2	17.0	14.6	13.4	16.0
Cerebrovascular disease	Western Europe	males	22.2	20.9	23.4	21.6	20.4	22.8
Hemorrhagic stroke	Western Europe	males	8.1	7.5	8.7	7.9	7.3	8.4
Ischemic stroke	Western Europe	males	15.9	14.5	17.2	15.5	14.2	16.8
Cerebrovascular disease	Western Europe	females	23.3	21.9	24.6	23.0	21.6	24.2
Hemorrhagic stroke	Western Europe	females	7.8	7.2	8.5	7.7	7.1	8.3
Ischemic stroke	Western Europe	females	17.2	15.8	18.5	16.9	15.6	18.3
Cerebrovascular disease	Western Europe	both sexes combined	22.7	21.4	23.9	22.3	21.0	23.4
Hemorrhagic stroke	Western Europe	both sexes combined	7.9	7.3	8.5	7.7	7.1	8.3
Ischemic stroke	Western Europe	both sexes combined	16.5	15.2	17.8	16.2	14.9	17.5
Cerebrovascular disease	Southern Latin America	males	17.8	16.3	19.3	20.4	19.1	21.7

Hemorrhagic stroke	Southern Latin America	males	6.0	5.4	6.7	7.0	6.3	7.6
Ischemic stroke	Southern Latin America	males	12.7	11.3	14.1	14.8	13.5	16.1
Cerebrovascular disease	Southern Latin America	females	20.6	18.9	22.3	22.3	20.8	23.8
Hemorrhagic stroke	Southern Latin America	females	6.0	5.4	6.7	6.6	5.9	7.1
Ischemic stroke	Southern Latin America	females	15.6	14.0	17.3	17.1	15.5	18.7
Cerebrovascular disease	Southern Latin America	both sexes combined	19.2	17.8	20.5	21.3	19.9	22.5
Hemorrhagic stroke	Southern Latin America	both sexes combined	6.0	5.4	6.6	6.7	6.0	7.3
Ischemic stroke	Southern Latin America	both sexes combined	14.2	12.8	15.6	15.9	14.5	17.4
Cerebrovascular disease	High-income North America	males	22.4	21.1	23.7	23.3	21.9	24.6
Hemorrhagic stroke	High-income North America	males	6.8	6.2	7.3	7.0	6.4	7.6
Ischemic stroke	High-income North America	males	17.4	15.9	18.8	18.0	16.5	19.5
Cerebrovascular disease	High-income North America	females	25.0	23.6	26.4	26.8	25.2	28.2
Hemorrhagic stroke	High-income North America	females	6.7	6.2	7.3	7.2	6.6	7.8
Ischemic stroke	High-income North America	females	20.1	18.5	21.5	21.5	19.8	23.0
Cerebrovascular disease	High-income North America	both sexes combined	23.8	22.3	25.0	25.1	23.6	26.4
Hemorrhagic stroke	High-income North America	both sexes combined	6.7	6.2	7.3	7.1	6.5	7.7
Ischemic stroke	High-income North America	both sexes combined	18.8	17.3	20.1	19.8	18.2	21.2
Cerebrovascular disease	Caribbean	males	18.0	16.6	19.3	20.7	19.4	22.1
Hemorrhagic stroke	Caribbean	males	6.0	5.5	6.6	7.1	6.5	7.7
Ischemic stroke	Caribbean	males	12.8	11.5	14.1	15.1	13.6	16.6
Cerebrovascular disease	Caribbean	females	20.8	19.3	22.3	24.9	23.4	26.5
Hemorrhagic stroke	Caribbean	females	7.1	6.4	7.8	8.7	7.9	9.5
Ischemic stroke	Caribbean	females	14.8	13.2	16.3	18.2	16.5	19.8
Cerebrovascular disease	Caribbean	both sexes combined	19.4	18.0	20.7	22.9	21.4	24.3
Hemorrhagic stroke	Caribbean	both sexes combined	6.5	6.0	7.2	7.9	7.2	8.6
Ischemic stroke	Caribbean	both sexes combined	13.8	12.4	15.1	16.7	15.1	18.2
Cerebrovascular disease	Andean Latin America	males	15.5	14.0	17.0	15.6	14.4	16.7
Hemorrhagic stroke	Andean Latin America	males	4.7	4.1	5.2	4.7	4.2	5.1
Ischemic stroke	Andean Latin America	males	11.5	10.0	12.9	11.5	10.3	12.8
Cerebrovascular disease	Andean Latin America	females	17.9	16.2	19.6	19.6	18.2	21.0
Hemorrhagic stroke	Andean Latin America	females	5.7	5.1	6.4	6.2	5.6	6.9
Ischemic stroke	Andean Latin America	females	13.1	11.5	14.7	14.4	12.9	15.8
Cerebrovascular disease	Andean Latin America	both sexes combined	16.7	15.2	18.2	17.6	16.4	18.8
Hemorrhagic stroke	Andean Latin America	both sexes combined	5.2	4.6	5.7	5.5	4.9	6.0
Ischemic stroke	Andean Latin America	both sexes combined	12.3	10.9	13.6	13.0	11.6	14.2
Cerebrovascular disease	Central Latin America	males	14.1	13.1	15.1	15.7	14.6	16.8
Hemorrhagic stroke	Central Latin America	males	4.7	4.2	5.1	5.2	4.7	5.7
Ischemic stroke	Central Latin America	males	10.0	9.0	11.0	11.2	10.0	12.2
Cerebrovascular disease	Central Latin America	females	16.4	15.2	17.6	18.4	17.2	19.7
Hemorrhagic stroke	Central Latin America	females	5.7	5.2	6.2	6.4	5.8	7.0
Ischemic stroke	Central Latin America	females	11.5	10.3	12.7	13.0	11.7	14.3
Cerebrovascular disease	Central Latin America	both sexes combined	15.2	14.2	16.4	17.1	15.9	18.3
Hemorrhagic stroke	Central Latin America	both sexes combined	5.2	4.7	5.7	5.8	5.3	6.3
Ischemic stroke	Central Latin America	both sexes combined	10.7	9.7	11.8	12.1	10.9	13.3
Cerebrovascular disease	Tropical Latin America	males	18.9	17.6	20.2	22.6	21.2	24.1
Hemorrhagic stroke	Tropical Latin America	males	4.8	4.3	5.2	5.8	5.3	6.4
Ischemic stroke	Tropical Latin America	males	14.9	13.6	16.3	18.1	16.6	19.7
Cerebrovascular disease	Tropical Latin America	females	19.5	18.1	20.9	22.2	20.8	23.7
Hemorrhagic stroke	Tropical Latin America	females	5.8	5.2	6.3	6.6	6.0	7.2
Ischemic stroke	Tropical Latin America	females	14.7	13.3	16.1	16.9	15.4	18.4
Cerebrovascular disease	Tropical Latin America	both sexes combined	19.1	17.9	20.5	22.3	20.9	23.7
Hemorrhagic stroke	Tropical Latin America	both sexes combined	5.3	4.8	5.7	6.2	5.7	6.8
Ischemic stroke	Tropical Latin America	both sexes combined	14.7	13.4	16.2	17.4	15.9	18.9
Cerebrovascular disease	North Africa and Middle East	males	19.4	17.8	20.9	22.7	21.1	24.2
Hemorrhagic stroke	North Africa and Middle East	males	5.2	4.7	5.8	6.3	5.7	6.9
Ischemic stroke	North Africa and Middle East	males	15.0	13.5	16.5	17.8	16.1	19.5
Cerebrovascular disease	North Africa and Middle East	females	23.1	21.4	24.8	27.8	25.8	29.7
Hemorrhagic stroke	North Africa and Middle East	females	5.9	5.3	6.6	7.6	6.8	8.3
Ischemic stroke	North Africa and Middle East	females	18.3	16.5	20.1	22.3	20.2	24.3
Cerebrovascular disease	North Africa and Middle East	both sexes combined	21.2	19.6	22.8	25.3	23.5	27.1
Hemorrhagic stroke	North Africa and Middle East	both sexes combined	5.6	5.0	6.1	7.0	6.3	7.6
Ischemic stroke	North Africa and Middle East	both sexes combined	16.6	15.0	18.2	20.1	18.2	21.9
Cerebrovascular disease	South Asia	males	13.5	12.5	14.5	18.9	17.6	20.1
Hemorrhagic stroke	South Asia	males	4.2	3.8	4.7	6.0	5.5	6.6
Ischemic stroke	South Asia	males	9.6	8.6	10.6	13.9	12.5	15.2
Cerebrovascular disease	South Asia	females	15.9	14.7	17.1	23.7	22.1	25.3
Hemorrhagic stroke	South Asia	females	4.7	4.3	5.3	7.3	6.6	8.0
Ischemic stroke	South Asia	females	11.6	10.4	12.9	17.9	16.3	19.6
Cerebrovascular disease	South Asia	both sexes combined	14.6	13.6	15.7	21.3	19.9	22.7
Hemorrhagic stroke	South Asia	both sexes combined	4.5	4.0	4.9	6.7	6.1	7.3
Ischemic stroke	South Asia	both sexes combined	10.6	9.5	11.6	15.9	14.4	17.3
Cerebrovascular disease	Central Sub-Saharan Africa	males	11.6	10.6	12.7	19.4	18.0	20.9
Hemorrhagic stroke	Central Sub-Saharan Africa	males	4.2	3.7	4.8	7.2	6.5	8.0
Ischemic stroke	Central Sub-Saharan Africa	males	7.7	6.7	8.7	13.5	12.0	14.9
Cerebrovascular disease	Central Sub-Saharan Africa	females	13.8	12.6	15.1	26.9	25.1	28.7
Hemorrhagic stroke	Central Sub-Saharan Africa	females	5.1	4.5	5.6	10.3	9.4	11.3
Ischemic stroke	Central Sub-Saharan Africa	females	9.1	8.0	10.3	19.1	17.1	20.8
Cerebrovascular disease	Central Sub-Saharan Africa	both sexes combined	12.8	11.7	13.8	23.3	21.8	24.9
Hemorrhagic stroke	Central Sub-Saharan Africa	both sexes combined	4.7	4.2	5.2	8.8	8.0	9.7
Ischemic stroke	Central Sub-Saharan Africa	both sexes combined	8.4	7.4	9.4	16.4	14.7	17.9
Cerebrovascular disease	Eastern Sub-Saharan Africa	males	11.2	10.3	12.3	18.3	17.0	19.6
Hemorrhagic stroke	Eastern Sub-Saharan Africa	males	4.0	3.6	4.5	6.8	6.1	7.5
Ischemic stroke	Eastern Sub-Saharan Africa	males	7.4	6.5	8.4	12.7	11.3	14.0
Cerebrovascular disease	Eastern Sub-Saharan Africa	females	12.5	11.4	13.6	22.5	20.9	24.1

Hemorrhagic stroke	Eastern Sub-Saharan Africa	females	4.5	4.0	5.0	8.5	7.6	9.3
Ischemic stroke	Eastern Sub-Saharan Africa	females	8.2	7.3	9.2	15.7	14.1	17.3
Cerebrovascular disease	Eastern Sub-Saharan Africa	both sexes combined	11.8	10.9	12.8	20.5	19.0	21.9
Hemorrhagic stroke	Eastern Sub-Saharan Africa	both sexes combined	4.3	3.8	4.8	7.6	6.9	8.4
Ischemic stroke	Eastern Sub-Saharan Africa	both sexes combined	7.9	6.9	8.8	14.2	12.7	15.6
Cerebrovascular disease	Southern Sub-Saharan Africa	males	10.0	9.2	10.9	19.7	18.5	21.0
Hemorrhagic stroke	Southern Sub-Saharan Africa	males	3.5	3.1	3.9	7.1	6.5	7.7
Ischemic stroke	Southern Sub-Saharan Africa	males	6.8	6.1	7.6	14.0	12.6	15.3
Cerebrovascular disease	Southern Sub-Saharan Africa	females	14.9	13.7	16.1	24.9	23.3	26.5
Hemorrhagic stroke	Southern Sub-Saharan Africa	females	4.9	4.4	5.4	8.6	7.8	9.4
Ischemic stroke	Southern Sub-Saharan Africa	females	10.7	9.6	11.8	18.3	16.6	19.9
Cerebrovascular disease	Southern Sub-Saharan Africa	both sexes combined	12.5	11.6	13.5	22.4	21.0	23.8
Hemorrhagic stroke	Southern Sub-Saharan Africa	both sexes combined	4.2	3.8	4.6	7.9	7.2	8.5
Ischemic stroke	Southern Sub-Saharan Africa	both sexes combined	8.8	7.9	9.7	16.2	14.7	17.7
Cerebrovascular disease	Western Sub-Saharan Africa	males	13.0	11.9	14.2	18.5	17.2	19.7
Hemorrhagic stroke	Western Sub-Saharan Africa	males	4.4	3.9	4.9	6.3	5.7	6.9
Ischemic stroke	Western Sub-Saharan Africa	males	9.0	8.0	10.1	13.2	11.8	14.5
Cerebrovascular disease	Western Sub-Saharan Africa	females	15.8	14.5	17.3	25.5	23.9	27.3
Hemorrhagic stroke	Western Sub-Saharan Africa	females	5.0	4.4	5.6	8.6	7.8	9.5
Ischemic stroke	Western Sub-Saharan Africa	females	11.4	10.1	12.7	19.0	17.2	20.7
Cerebrovascular disease	Western Sub-Saharan Africa	both sexes combined	14.4	13.3	15.7	22.0	20.6	23.5
Hemorrhagic stroke	Western Sub-Saharan Africa	both sexes combined	4.7	4.2	5.2	7.5	6.8	8.2
Ischemic stroke	Western Sub-Saharan Africa	both sexes combined	10.2	9.1	11.4	16.1	14.5	17.6

Table S6. Lifetime Risk of Stroke in 2016, by Sociodemographic Index Quintiles				
**Cause**	**Location**	**Mean lifetime risk (%)**	**95% UI Lower**	**95% UI Upper**
Stroke	High SDI	23.53	22.19	24.75
Stroke	High-middle SDI	31.07	29.05	33.01
Stroke	Middle SDI	29.30	27.77	30.77
Stroke	Low-middle SDI	16.76	15.62	17.88
Stroke	Low SDI	13.24	12.27	14.23
Hemorrhagic stroke	High SDI	7.31	6.74	7.89
Hemorrhagic stroke	High-middle SDI	8.63	7.83	9.39
Hemorrhagic stroke	Middle SDI	11.11	10.27	11.94
Hemorrhagic stroke	Low-middle SDI	5.61	5.08	6.17
Hemorrhagic stroke	Low SDI	4.58	4.14	5.06
Ischemic stroke	High SDI	17.95	16.54	19.29
Ischemic stroke	High-middle SDI	24.91	22.77	26.91
Ischemic stroke	Middle SDI	20.56	18.91	22.14
Ischemic stroke	Low-middle SDI	11.79	10.62	12.93
Ischemic stroke	Low SDI	9.00	8.03	9.97

Table S7. Lifetime Risk of Stroke in 2016, by Age				
**Cause**	**Age**	**Mean lifetime risk (%)**	**95% UI Lower**	**95% UI Upper**
Stroke	25 to 29	24.88	23.47	26.22
Stroke	30 to 34	25.03	23.63	26.39
Stroke	35 to 39	25.19	23.79	26.55
Stroke	40 to 44	25.33	23.90	26.68
Stroke	45 to 49	25.35	23.87	26.71
Stroke	50 to 54	25.16	23.67	26.55
Stroke	55 to 59	24.79	23.28	26.23
Stroke	60 to 64	24.23	22.70	25.69
Stroke	65 to 69	23.39	21.85	24.92
Stroke	70 to 74	22.40	20.75	23.93
Stroke	75 to 79	21.19	19.47	22.80
Stroke	80 to 84	19.31	17.87	20.73
Stroke	85 to 89	17.43	16.08	18.86
Stroke	90 to 94	15.47	13.94	17.03
Stroke	95 plus	13.40	11.75	15.14
Hemorrhagic stroke	25 to 29	8.21	7.54	8.86
Hemorrhagic stroke	30 to 34	8.26	7.59	8.91
Hemorrhagic stroke	35 to 39	8.30	7.62	8.96
Hemorrhagic stroke	40 to 44	8.33	7.65	9.01
Hemorrhagic stroke	45 to 49	8.31	7.62	8.99
Hemorrhagic stroke	50 to 54	8.19	7.48	8.88
Hemorrhagic stroke	55 to 59	8.01	7.29	8.71
Hemorrhagic stroke	60 to 64	7.79	7.07	8.48
Hemorrhagic stroke	65 to 69	7.49	6.76	8.19
Hemorrhagic stroke	70 to 74	7.13	6.42	7.85
Hemorrhagic stroke	75 to 79	6.71	5.97	7.41
Hemorrhagic stroke	80 to 84	6.19	5.55	6.86
Hemorrhagic stroke	85 to 89	5.64	4.96	6.30
Hemorrhagic stroke	90 to 94	5.02	4.28	5.75
Hemorrhagic stroke	95 plus	4.17	3.45	4.88
Ischemic stroke	25 to 29	18.34	16.86	19.80
Ischemic stroke	30 to 34	18.46	16.97	19.94
Ischemic stroke	35 to 39	18.58	17.08	20.07
Ischemic stroke	40 to 44	18.70	17.17	20.21
Ischemic stroke	45 to 49	18.74	17.16	20.25
Ischemic stroke	50 to 54	18.64	17.01	20.14
Ischemic stroke	55 to 59	18.40	16.74	19.88
Ischemic stroke	60 to 64	17.99	16.39	19.53
Ischemic stroke	65 to 69	17.37	15.69	18.94
Ischemic stroke	70 to 74	16.65	14.98	18.29
Ischemic stroke	75 to 79	15.79	14.00	17.52
Ischemic stroke	80 to 84	14.29	12.78	15.82
Ischemic stroke	85 to 89	12.85	11.51	14.25
Ischemic stroke	90 to 94	11.33	9.89	12.91
Ischemic stroke	95 plus	9.63	8.09	11.28

Table S8. Citations by cause	
**Cause Name**	**Citation**
Stroke	Arauz A, Villarreal-Careaga J, Rangel-Guerra R, Ramos-Moreno A, Barinagarrementeria F, PREMIER Investigators. Acute care and one-year outcome of Mexican patients with first-ever acute ischemic stroke: the PREMIER study. Rev Neurol. 2010; 51(11): 641–9
Stroke	Leonards CO, Ipsen N, Malzahn U, Fiebach JB, Endres M, Ebinger M. White matter lesion severity in mild acute ischemic stroke patients and functional outcome after 1 year. Stroke. 2012; 43(11): 3046–51
Stroke	Vemmos KN, Bots ML, Tsibouris PK, Zis VP, Takis CE, Grobbee DE, Stamatelopoulos S. Prognosis of stroke in the south of Greece: 1 year mortality, functional outcome and its determinants: the Arcadia Stroke Registry. J Neurol Neurosurg Psychiatr. 2000; 69(5): 595-600
Stroke	Heikinheimo T, Chimbayo D, Kumwenda JJ, Kampondeni S, Allain TJ. Stroke outcomes in Malawi, a country with high prevalence of HIV: a prospective follow-up study. PLoS One. 2012; 7(3): e33765
Stroke	Unnikrishnan JP, Sylaja S, Nayak SD, Radhakrishnan K. India - Trivandrum Stroke Registry 2005. [Unpublished]
Stroke	Kulshrestha M, Vidyanand. An analysis of the risk factors and the outcomes of cerebrovascular diseases in northern India. J Clin Diagn Res. 2013; 7(1): 127–31
Stroke	Appelros P, Terent A. Characteristics of the National Institute of Health Stroke Scale: results from a population-based stroke cohort at baseline and after one year. Cerebrovasc Dis. 2004; 17(1): 21–7
Stroke	Counsell C, Dennis M, McDowall M. Predicting functional outcome in acute stroke: comparison of a simple six variable model with other predictive systems and informal clinical prediction. J Neurol Neurosurg Psychiatry. 2004; 75(3): 401–5
Stroke	Abubakar SA, Okubadejo NU, Ojo OO, Oladipo O, Ojini FI, Danesi MA. Relationship between admission serum C-reactive protein and short term outcome following acute ischaemic stroke at a tertiary health institution in Nigeria. Niger J Clin Pract. 2013; 16(3): 320–4
Stroke	Rivero-Arias O, Ouellet M, Gray A, Wolstenholme J, Rothwell PM, Luengo-Fernandez R. Mapping the modified Rankin scale (mRS) measurement into the generic EuroQol (EQ-5D) health outcome. Med Decis Mak. 2010; 30(3): 341–54
Stroke	Luengo-Fernandez R, Paul NLM, Gray AM, Pendlebury ST, Bull LM, Welch SJV, Cuthbertson FC, Rothwell PM, Oxford Vascular Study. Population-based study of disability and institutionalization after transient ischemic attack and stroke: 10-year results of the Oxford Vascular Study. Stroke. 2013; 44(10): 2854–611
Stroke	Fernandes TG, Goulart AC, Santos-Junior WR, Alencar AP, Benseñor IM, Lotufo PA. Educational levels and the functional dependence of ischemic stroke survivors. Cad Saude Publica. 2012; 28(8): 1581–90
Stroke	Corso G, Bottacchi E, Giardini G, Di Giovanni M, Meloni T, Pesenti Campagnoni M, Veronese Morosini M. Epidemiology of stroke in northern Italy: the Cerebrovascular Aosta Registry, 2004-2008. Neurol Sci. 2013; 34(7): 1071–81
Stroke	Kong F-Y, Tao W-D, Hao Z-L, Liu M. Predictors of one-year disability and death in Chinese hospitalized women after ischemic stroke. Cerebrovasc Dis. 2010; 29(3): 255–62
Stroke	Hattori N, Hirayama T, Katayama Y. Medical care for chronic-phase stroke in Japan. Neurol Med Chir (Tokyo). 2012; 52(4): 175–80
Stroke	Kim J-M, Stewart R, Park M-S, Kang H-J, Kim S-W, Shin I-S, Kim H-R, Shin M-G, Cho K-H, Yoon J-S. Associations of BDNF genotype and promoter methylation with acute and long-term stroke outcomes in an East Asian cohort. PLoS One. 2012; 7(12): e51280
Stroke	Vibo R, Korv J, Roose M. One-year outcome after first-ever stroke according to stroke subtype, severity, risk factors and pre-stroke treatment. A population-based study from Tartu, Estonia. Eur J Neurol. 2007; 14(4): 435–9
Stroke	Kang H-J, Stewart R, Park M-S, Bae K-Y, Kim S-W, Kim J-M, Shin I-S, Cho K-H, Yoon J-S. White matter hyperintensities and functional outcomes at 2 weeks and 1 year after stroke. Cerebrovasc Dis. 2013; 35(2): 138–45
Stroke	World Health Organization Regional Office for Europe (WHO/Europe). European Hospital Morbidity Database 1999-2007. Copenhagen, Denmark: World Health Organization Regional Office for Europe (WHO/Europe)
Stroke	Center for Research and Teaching in Economics (CIDE) (Mexico), National Institute of Perinatology (Mexico), National Institute of Statistics and Geography (INEGI) (Mexico), Universidad Iberoamericana. Mexico Family Life Survey 2002
Stroke	National Institute of Public Health (Mexico). Mexico National Health Survey 1999-2000
Stroke	National Institute of Public Health (Mexico). Mexico National Survey of Health and Nutrition 2005-2006. Cuernavaca, Mexico: National Institute of Public Health (Mexico)
Stroke	World Health Organization (WHO). Congo World Health Survey 2003. Geneva, Switzerland: World Health Organization (WHO), 2005
Stroke	World Health Organization (WHO). France World Health Survey 2003. Geneva, Switzerland: World Health Organization (WHO), 2005
Stroke	World Health Organization (WHO). Kenya World Health Survey 2004. Geneva, Switzerland: World Health Organization (WHO), 2005
Stroke	World Health Organization (WHO). Mexico World Health Survey 2002-2003. Geneva, Switzerland: World Health Organization (WHO), 2005
Stroke	World Health Organization (WHO). Norway World Health Survey 2003. Geneva, Switzerland: World Health Organization (WHO), 2005
Stroke	World Health Organization (WHO). Vietnam World Health Survey 2002-2003. Geneva, Switzerland: World Health Organization (WHO), 2005
Stroke	General Statistics Office (Viet Nam), United Nations Development Programme (UNDP), World Bank (WB). Viet Nam Living Standards Measurement Survey 2008. Ha N?i, Viet Nam: General Statistics Office (Viet Nam)
Stroke	General Statistics Office (Viet Nam), United Nations Development Programme (UNDP), World Bank. Vietnam Living Standards Measurement Survey 2006
Stroke	National Institute of Statistics (Cambodia), Statistics Sweden. Cambodia Socio-Economic Survey 2003-2005. Phnom Penh, Cambodia: National Institute of Statistics (Cambodia)
Stroke	National Institute of Statistics (Cambodia), Statistics Sweden. Cambodia Socio-Economic Survey 2006-2007. Phnom Penh, Cambodia: National Institute of Statistics (Cambodia)
Stroke	University of Wisconsin-Madison, Inter-University Consortium for Political and Social Research (ICPSR), College of the Northern Border (COLEF), Research in Health and Demographics (INSAD), National Institute of Medical Sciences and Nutrition Salvador Zubirán. Mexico - Mexico City Survey on Health, Well-Being, and Aging in Latin
Stroke	Management Research (IIHMR), Ministry of Public Health (Afghanistan), World Health Organization Regional Office for the Eastern Mediterranean (EMRO-WHO). Afghanistan Special Demographic and Health Survey 2010. Fairfax, United States: ICF International
Stroke	California Center for Population Research (CCPR), University of California Los Angeles (UCLA), Center for Research and Teaching in Economics (CIDE) (Mexico), National Institute of Public Health (Mexico), Universidad Iberoamericana. Mexico Family Life Survey 2005-2006
Stroke	Organization for Economic Co-operation and Development (OECD). OECD Health Statistics. Paris, France: Organization for Economic Co-operation and Development (OECD)
Stroke	National Institute of Public Health (Mexico). Mexico National Survey of Health and Nutrition 2011-2012. Cuernavaca, Mexico: National Institute of Public Health (Mexico)
Stroke	Ellekjær H, Holmen J, Indredavik B, Terent A. Epidemiology of Stroke in Innherred, Norway, 1994 to 1996: Incidence and 30-Day Case-Fatality Rate. Stroke. 1997; 28(11): 2180-4
Stroke	Bejot Y, Rouaud O, Durier J, Caillier M, Marie C, Freysz M, Yeguiayan J-M, Chantegret A, Osseby G, Moreau T, Giroud M. Decrease in the Stroke Case Fatality Rates in a French Population-Based Twenty-Year Study. Cerebrovasc Dis. 2007; 24(5): 439-44
Stroke	Giroud M, Lemesle M, Gouyon JB, Nivelon JL, Milan C, Dumas R. Cerebrovascular disease in children under 16 years of age in the city of Dijon, France: a study of incidence and clinical features from 1985 to 1993. J Clin Epidemiol. 1995; 48(11): 1343-8

Stroke	Smadja D, Cabre P, May F, Fanon J-L, René-Corail P, Riocreux C, Charpentier J-C, Fournerie P, Saint-Vil M, Ketterlé J. ERMANCIA: Epidemiology of Stroke in Martinique, French West Indies. Stroke. 2001; 32(12): 2741-7
Stroke	Wolfe CDA, Giroud M, Kolominsky-Rabas P, Dundas R, Lemesle M, Heuschmann P, Rudd A. Variations in Stroke Incidence and Survival in 3 Areas of Europe. Stroke. 2000; 31(9): 2074-9
Stroke	National Institute of Statistics, Geography, and Informatics (Mexico), Population Studies Center, University of Pennsylvania, University of Maryland, University of Wisconsin. Mexico Health and Aging Study 2001
Stroke	National Institute of Statistics, Geography, and Informatics (Mexico), Population Studies Center, University of Pennsylvania, University of Maryland, University of Wisconsin. Mexico Health and Aging Study 2003
Stroke	National Institute of Statistics, Geography, and Informatics (Mexico), Population Studies Center, University of Pennsylvania, University of Maryland, University of Wisconsin. Mexico Health and Aging Study 2012. Mexico City, México: National Institute of Statistics, Geography, and Informatics (Mexico)
Stroke	National Institute of Public Health (Mexico), World Health Organization (WHO). Mexico WHO Study on Global AGEing and Adult Health 2009-2010. Geneva, Switzerland: World Health Organization (WHO), 2011
Stroke	Béjot Y, Benzenine E, Lorgis L, Zeller M, Aubé H, Giroud M, Cottin Y, Quantin C. Comparative analysis of patients with acute coronary and cerebrovascular syndromes from the national French hospitalization health care system database. Neuroepidemiology. 2011; 37(3-4): 143-52
Stroke	Cantu-Brito C, Majersik JJ, Sánchez BN, Ruano A, Becerra-Mendoza D, Wing JJ, Morgenstern LB. Door-to-Door Capture of Incident and Prevalent Stroke Cases in Durango, Mexico The Brain Attack Surveillance in Durango Study. Stroke. 2011; 42(3): 601-6
Stroke	Cossi M-J, Gobron C, Preux P-M, Niama D, Chabriat H, Houinato D. Stroke: prevalence and disability in Cotonou, Benin. Cerebrovasc Dis. 2012; 33(2): 166-72
Stroke	Mohammad QD, Habib M, Hoque A, Alam B, Haque B, Hossain S, Rahman KM, Khan SU. Prevalence of stroke above forty years. Mymensingh Med J. 2011; 20(4): 640-4
Stroke	Börsch-Supan, A. (2013). Survey of Health, Ageing and Retirement in Europe (SHARE) Wave 1. Release version: 2.6.0. SHARE-ERIC. Data set. DOI: 10.6103/SHARE.w1.260
Stroke	Börsch-Supan, A. (2013). Survey of Health, Ageing and Retirement in Europe (SHARE) Wave 2. Release version: 2.6.0. SHARE-ERIC. Data set. DOI: 10.6103/SHARE.w2.260
Stroke	Börsch-Supan, A. (2013). Survey of Health, Ageing and Retirement in Europe (SHARE) Wave 4. Release version: 1.1.1. SHARE-ERIC. Data set. DOI: 10.6103/SHARE.w4.111
Stroke	(KEMRI), Kenya National Bureau of Statistics, Ministry of Public Health and Sanitation (Kenya), National AIDS Control Council (Kenya), National AIDS and STI Control Program (Kenya), National Coordinating Agency for Population and Development (Kenya), National Public Health Laboratory Services, Ministry of Public Health and
Stroke	Norwegian Directorate of Health. Norway Patient Register 2009
Stroke	Norwegian Directorate of Health. Norway Patient Register 2010
Stroke	Norwegian Directorate of Health. Norway Patient Register 2011
Stroke	Norwegian Directorate of Health. Norway Patient Register 2012
Stroke	National Institute of Public Health (Mexico), World Health Organization (WHO). Mexico WHO Multi-country Survey Study on Health and Health System Responsiveness 2000-2001. Geneva, Switzerland: World Health Organization (WHO)
Stroke	Abt Associates Inc., Kenya National Bureau of Statistics, Ministry of Health (Kenya). Kenya Household Health Expenditure and Utilization Survey 2007. Nairobi, Kenya: Kenya National Bureau of Statistics
Stroke	Center for Research and Teaching in Economics (CIDE) (Mexico), Duke University, National Institute of Public Health (Mexico), Universidad Iberoamericana, University of California, Los Angeles (UCLA). Mexico Family Life Survey 2008-2013
Stroke	Action Africa Help International (AAH-I), Institute for Health Metrics and Evaluation (IHME), Ministry of Medical Services (Kenya), Ministry of Public Health and Sanitation (Kenya). Access, Bottlenecks, Costs, and Equity (ABCE) project in Kenya, 2012. Seattle, United States: Institute for Health Metrics and Evaluation (IHME), 2015
Stroke	Infectious Diseases Research Collaboration (IDRC), Institute for Health Metrics and Evaluation (IHME), Makerere University, Ministry of Health (Uganda). Access, Bottlenecks, Costs, and Equity (ABCE) project in Uganda, 2012. Seattle, United States: Institute for Health Metrics and Evaluation (IHME), 2015
Stroke	National Institute of Statistics (Cambodia), Statistics Sweden. Cambodia Socio-Economic Survey 2007-2008. Phnom Penh, Cambodia: National Institute of Statistics (Cambodia)
Stroke	Gallup Europe, World Health Organization (WHO). Bahrain WHO Multi-country Survey Study on Health and Health System Responsiveness 2000-2001. Geneva, Switzerland: World Health Organization (WHO)
Stroke	Erik Consulting, International Research Associates (INRA) Europe, World Health Organization (WHO). France WHO Multi-country Survey Study on Health and Health System Responsiveness 2000-2001. Geneva, Switzerland: World Health Organization (WHO)
Stroke	Biomedical Engineering Institute, Kaunas University of Technology, Statistics Lithuania, World Health Organization (WHO). Lithuania WHO Multi-country Survey Study on Health and Health System Responsiveness 2000-2001. Geneva, Switzerland: World Health Organization (WHO)
Stroke	Gallup Europe, World Health Organization (WHO). Oman WHO Multi-country Survey Study on Health and Health System Responsiveness 2000-2001. Geneva, Switzerland: World Health Organization (WHO)
Stroke	Börsch-Supan, A. (2015). Survey of Health, Ageing and Retirement in Europe (SHARE) Wave 5. Release version: 1.0.0. SHARE-ERIC. Data set. DOI: 10.6103/SHARE.w5.100
Stroke	Norwegian Directorate of Health. Norway Patient Register 2008-2012
Stroke	Norwegian Directorate of Health. Norway Patient Register 2002
Stroke	Norwegian Directorate of Health. Norway Patient Register 2003
Stroke	Ministry of Health (Vietnam). Vietnam Hospital Data 2013
Stroke	World Health Organization Regional Office for Europe (WHO/Europe). European Health for All Database - Inpatient Care Discharges Per 100. Copenhagen, Denmark: World Health Organization Regional Office for Europe (WHO/Europe)
Stroke	Norwegian Directorate of Health. Norway Patient Register 2013
Stroke	Norwegian Directorate of Health. Norway Patient Register 2014
Stroke	Norwegian Directorate of Health. Norway Patient Register 2001
Stroke	Norwegian Directorate of Health. Norway Patient Register 2000
Stroke	Norwegian Directorate of Health. Norway Patient Register 1999
Stroke	Norwegian Directorate of Health. Norway Patient Register 1998
Stroke	Norwegian Directorate of Health. Norway Patient Register 1997
Stroke	Norwegian Directorate of Health. Norway Patient Register 1996
Stroke	Norwegian Directorate of Health. Norway Patient Register 1995
Stroke	Norwegian Directorate of Health. Norway Patient Register 1994
Stroke	Norwegian Directorate of Health. Norway Patient Register 1993

Stroke	Norwegian Directorate of Health. Norway Patient Register 1992
Stroke	Norwegian Directorate of Health. Norway Patient Register 1991
Stroke	Norwegian Directorate of Health. Norway Patient Register 1990
Stroke	Norwegian Directorate of Health. Norway Patient Register 1989
Stroke	Norwegian Directorate of Health. Norway Patient Register 1988
Stroke	Norwegian Directorate of Health. Norway Patient Register 1987
Stroke	Norwegian Directorate of Health. Norway Patient Register 1986
Stroke	Norwegian Directorate of Health. Norway Patient Register 1985
Stroke	Norwegian Directorate of Health. Norway Patient Register 1984
Stroke	Norwegian Directorate of Health. Norway Patient Register 1983
Stroke	Norwegian Directorate of Health. Norway Patient Register 1982
Stroke	Norwegian Directorate of Health. Norway Patient Register 1981
Stroke	Norwegian Directorate of Health. Norway Patient Register 1980
Stroke	Norwegian Directorate of Health. Norway Patient Register 1979
Stroke	Norwegian Directorate of Health. Norway Patient Register 1978
Stroke	Norwegian Directorate of Health. Norway Patient Register 1977
Stroke	Norwegian Directorate of Health. Norway Patient Register 1975
Stroke	Norwegian Directorate of Health. Norway Patient Register 1973
Stroke	Statistics Canada. Canada Community Health Survey 2000-2001. Ottawa, Canada: Statistics Canada, 2003
Stroke	Statistics Indonesia. Indonesia National Socioeconomic Survey 2005
Stroke	RAND Corporation, University of Indonesia. Indonesia Family Life Survey 1993-1994. Santa Monica, United States: RAND Corporation
Stroke	Center for Population and Policy Studies, Gadjah Mada University (Indonesia), RAND Corporation, SurveyMETER. Indonesia Family Life Survey 2007-2008. Santa Monica, United States: RAND Corporation
Stroke	Central Bureau of Statistics (Indonesia). Indonesia National Socioeconomic Survey 1992
Stroke	Central Bureau of Statistics (Indonesia). Indonesia National Socioeconomic Survey 1993
Stroke	Central Bureau of Statistics (Indonesia). Indonesia National Socioeconomic Survey 1994
Stroke	Central Bureau of Statistics (Indonesia), Ministry of Health (Indonesia), United Nations Children's Fund (UNICEF). Indonesia National Socioeconomic Survey 1995
Stroke	Central Bureau of Statistics (Indonesia), Ministry of Health (Indonesia), United Nations Children's Fund (UNICEF). Indonesia National Socioeconomic Survey 1996
Stroke	Central Bureau of Statistics (Indonesia), Ministry of Health (Indonesia), United Nations Children's Fund (UNICEF). Indonesia National Socioeconomic Survey 1997
Stroke	Central Bureau of Statistics (Indonesia), Ministry of Health (Indonesia), World Bank. Indonesia National Socioeconomic Survey 2000
Stroke	Central Bureau of Statistics (Indonesia), Ministry of Health (Indonesia), World Bank. Indonesia National Socioeconomic Survey 2001
Stroke	Statistics Indonesia. Indonesia National Socioeconomic Survey 2004
Stroke	National Institute of Statistics and Censuses (Nicaragua), World Bank. Nicaragua Living Standards Measurement Survey 1993
Stroke	National Institute of Statistics and Censuses (Nicaragua), World Bank. Nicaragua Living Standards Measurement Survey 1998-1999
Stroke	Central Statistical Office (Zambia). Zambia Living Conditions Monitoring Survey 2002-2003. Lusaka, Zambia: Central Statistical Office (Zambia)
Stroke	Central Statistical Office (Zambia). Zambia Living Conditions Monitoring Survey 2004-2005. Lusaka, Zambia: Central Statistical Office (Zambia)
Stroke	Australian Bureau of Statistics. Australia National Health Survey 1995. Canberra, Australia: Australian Bureau of Statistics
Stroke	Analytical and Information Center of the Ministry of Health of Uzbekistan, Macro International, Inc, Ministry of Macroeconomics and Statistics (Uzbekistan). Uzbekistan Special Demographic and Health Survey 2002. Calverton, United States: Macro International, Inc
Stroke	World Health Organization (WHO). Bangladesh World Health Survey 2003. Geneva, Switzerland: World Health Organization (WHO), 2005
Stroke	World Health Organization (WHO). Burkina Faso World Health Survey 2002-2003. Geneva, Switzerland: World Health Organization (WHO), 2005
Stroke	World Health Organization (WHO). Dominican Republic World Health Survey 2003. Geneva, Switzerland: World Health Organization (WHO), 2005
Stroke	World Health Organization (WHO). Germany World Health Survey 2004. Geneva, Switzerland: World Health Organization (WHO), 2005
Stroke	World Health Organization (WHO). Mali World Health Survey 2003. Geneva, Switzerland: World Health Organization (WHO), 2005
Stroke	World Health Organization (WHO). Portugal World Health Survey 2003. Geneva, Switzerland: World Health Organization (WHO), 2006
Stroke	World Health Organization (WHO). Zambia World Health Survey 2003. Geneva, Switzerland: World Health Organization (WHO), 2005
Stroke	Federal Environment Agency (Germany), Federal Institute for Drugs and Medical Devices (Germany), Max Planck Institute of Psychiatry, Robert Koch Institute. Germany National Health Interview and Examination Survey 1997-1999. Berlin, Germany: Robert Koch Institute, 2000
Stroke	Centre for Health Promotion Studies, National University of Ireland, Galway, Health Promotion Unit, Department of Health and Children (Ireland). Ireland Survey of Lifestyle Attitudes and Nutrition 1998. Dublin, Ireland: Health Promotion Unit, Department of Health and Children (Ireland)
Stroke	Statistics Indonesia. Indonesia National Socioeconomic Survey 2010
Stroke	Family Health International, Ministry of Health (Indonesia), National AIDS Commission (KPA), Statistics Indonesia. Indonesia Behavioral Surveillance Survey 2007
Stroke	Statistics Indonesia. Indonesia National Socioeconomic Survey 2002
Stroke	Statistics Indonesia. Indonesia National Socioeconomic Survey 2008
Stroke	Statistics Indonesia. Indonesia National Socioeconomic Survey - Poverty Program Evaluation 2006. Jakarta, Indonesia: Statistics Indonesia
Stroke	Statistics Indonesia. Indonesia National Socioeconomic Survey - Poverty Program Evaluation 2008-2009. Jakarta, Indonesia: Statistics Indonesia
Stroke	Federal Statistical Office (Germany). Germany Hospital Discharges by Diagnosis 2009. Wiesbaden, Germany: Federal Statistical Office (Germany), 2011
Stroke	Hamad Medical Corporation (Qatar). Qatar - Annual Inpatients Discharge Abstract: Hamad General Hospital 2002. Doha, Qatar: Hamad Medical Corporation (Qatar)
Stroke	Hamad Medical Corporation (Qatar). Qatar - Annual Inpatients Discharge Abstract: Hamad General Hospital and Women's Hospital 2003. Doha, Qatar: Hamad Medical Corporation (Qatar)
Stroke	Statistics Indonesia. Indonesia National Socioeconomic Survey 2011
Stroke	Health Care International, World Health Organization (WHO). Egypt WHO Multi-country Survey Study on Health and Health System Responsiveness 2000-2001. Geneva, Switzerland: World Health Organization (WHO)
Stroke	Institute of Health Systems (India), World Health Organization (WHO). India - Andhra Pradesh WHO Multi-country Survey Study on Health and Health System Responsiveness 2000-2001
Stroke	Thrift AG, Dewey HM, Sturm JW, Srikanth VK, Gilligan AK, Gall SL, Macdonell RAL, McNeil JJ, Donnan GA. Incidence of stroke subtypes in the North East Melbourne Stroke Incidence Study (NEMESIS): differences between men and women. Neuroepidemiology. 2009; 32(1): 11-8
Stroke	Islam MS, Anderson CS, Hankey GJ, Hardie K, Carter K, Broadhurst R, Jamrozik K. Trends in Incidence and Outcome of Stroke in Perth, Western Australia During 1989 to 2001. Stroke. 2008; 39(3): 776-82

Stroke	Kolominsky-Rabas PL, Sarti C, Heuschmann PU, Graf C, Siemonsen S, Neundoerfer B, Katalinic A, Lang E, Gassmann K-G, von Stockert TR. A Prospective Community-Based Study of Stroke in Germany-The Erlangen Stroke Project (ESPro): Incidence and Case Fatality at 1, 3, and 12 Months. Stroke. 1998; 29(12): 2501-6
Stroke	Thrift AG, Dewey HM, Macdonell RAL, McNeil JJ, Donnan GA. Incidence of the Major Stroke Subtypes: Initial Findings From the North East Melbourne Stroke Incidence Study (NEMESIS). Stroke. 2001; 32(8): 1732-8
Stroke	Correia M, Silva MR, Matos I, Magalhães R, Lopes JC, Ferro JM, Silva MC. Prospective Community-Based Study of Stroke in Northern Portugal: Incidence and Case Fatality in Rural and Urban Populations. Stroke. 2004; 35(9): 2048-53
Stroke	Hamad A, Hamad A, Sokrab TEO, Momeni S, Mesraoua B, Lingren A. Stroke in Qatar: A one-year, hospital-based study. J Stroke Cerebrovasc Dis. 2001; 10(5): 236-41
Stroke	Thrift AG, Dewey HM, Macdonell RAL, McNeil JJ, Donnan GA. Stroke Incidence on the East Coast of Australia: The North East Melbourne Stroke Incidence Study (NEMESIS). Stroke. 2000; 31(9): 2087-92
Stroke	Palm F, Urbanek C, Rose S, Buggle F, Bode B, Hennerici MG, Schmieder K, Inselmann G, Reiter R, Fleischer R, Piplack K-O, Safer A, Becher H, Grau AJ. Stroke Incidence and Survival in Ludwigshafen am Rhein, Germany: the Ludwigshafen Stroke Study (LuSSt). Stroke. 2010; 41(9): 1865-70
Stroke	Jungehülsing GJ, Müller-Nordhorn J, Nolte CH, Roll S, Rossnagel K, Reich A, Wagner A, Einhäupl KM, Willich SN, Villringer A. Prevalence of stroke and stroke symptoms: a population-based survey of 28,090 participants. Neuroepidemiology. 2008; 30(1): 51-7
Stroke	Nicoletti A, Sofia V, Giuffrida S, Bartoloni A, Bartalesi F, Bartolo MLL, Fermo SL, Cocuzza V, Gamboa H, Salazar E, Reggio A. Prevalence of Stroke: A Door-to-Door Survey in Rural Bolivia. Stroke. 2000; 31(4): 882-5
Stroke	Salonen JT, Puska P, Tuomilehto J. Physical activity and risk of myocardial infarction, cerebral stroke and death: a longitudinal study in Eastern Finland. Am J Epidemiol. 1982; 115(4): 526-37
Stroke	Bijnen FC, Caspersen CJ, Feskens EJ, Saris WH, Mosterd WL, Kromhout D. Physical activity and 10-year mortality from cardiovascular diseases and all causes: The Zutphen Elderly Study. Arch Intern Med. 1998; 158(14): 1499-505
Stroke	Lee IM, Hennekens CH, Berger K, Buring JE, Manson JE. Exercise and risk of stroke in male physicians. Stroke. 1999; 30(1): 1-6
Stroke	Abbott RD, Rodriguez BL, Burchfiel CM, Curb JD. Physical activity in older middle-aged men and reduced risk of stroke: the Honolulu Heart Program. Am J Epidemiol. 1994; 139(9): 881-93
Stroke	Agnarsson U, Thorgeirsson G, Sigvaldason H, Sigfusson N. Effects of leisure-time physical activity and ventilatory function on risk for stroke in men: the Reykjavík Study. Ann Intern Med. 1999; 130(12): 987-90
Stroke	Okada H, Horibe H, Yoshiyuki O, Hayakawa N, Aoki N. A prospective study of cerebrovascular disease in Japanese rural communities, Akabane and Asahi. Part 1: evaluation of risk factors in the occurrence of cerebral hemorrhage and thrombosis. Stroke. 1976; 7(6): 599-607
Stroke	Paganini-Hill A, Perez Barreto M. Stroke risk in older men and women: aspirin, estrogen, exercise, vitamins, and other factors. J Gend Specif Med. 2001; 4(2): 18-28
Stroke	Paffenbarger RS Jr, Brand RJ, Sholtz RI, Jung DL. Energy expenditure, cigarette smoking, and blood pressure level as related to death from specific diseases. Am J Epidemiol. 1978; 108(1): 12-8
Stroke	Hu FB, Stampfer MJ, Colditz GA, Ascherio A, Rexrode KM, Willett WC, Manson JE. Physical activity and risk of stroke in women. JAMA. 2000; 283(22): 2961-7
Stroke	Ellekjaer H, Holmen J, Ellekjaer E, Vatten L. Physical activity and stroke mortality in women. Ten-year follow-up of the Nord-Trondelag health survey, 1984-1986. Stroke. 2000; 31(1): 14-8
Stroke	Håheim LL, Holme I, Hjermann I, Leren P. Risk factors of stroke incidence and mortality. A 12-year follow-up of the Oslo Study. Stroke. 1993; 24(10): 1484-9
Stroke	Lindenstrøm E, Boysen G, Nyboe J. Lifestyle factors and risk of cerebrovascular disease in women. The Copenhagen City Heart Study. Stroke. 1993; 24(10): 1468-72
Stroke	Wannamethee G, Shaper AG. Physical activity and stroke in British middle aged men. BMJ. 1992; 304(6827): 597-601
Stroke	Lapidus L, Bengtsson C. Socioeconomic factors and physical activity in relation to cardiovascular disease and death. A 12 year follow up of participants in a population study of women in Gothenburg, Sweden. Br Heart J. 1986; 55(3): 295-301
Stroke	Simonsick EM, Lafferty ME, Phillips CL, Mendes de Leon CF, Kasl SV, Seeman TE, Fillenbaum G, Hebert P, Lemke JH. Risk due to inactivity in physically capable older adults. Am J Public Health. 1993; 83(10): 1443-50
Stroke	Lee IM, Paffenbarger RS Jr. Physical activity and stroke incidence: the Harvard Alumni Health Study. Stroke. 1998; 29(10): 2049-54
Stroke	Palm F, Dos Santos M, Urbanek C, Greulich M, Zimmer K, Safer A, Grau AJ, Becher H. Stroke seasonality associations with subtype, etiology and laboratory results in the Ludwigshafen Stroke Study (LuSSt). Eur J Epidemiol. 2013; 28(5): 373-81
Stroke	Ferri CP, Schoenborn C, Kalra L, Acosta D, Guerra M, Huang Y, Jacob KS, Rodriguez JJL, Salas A, Sosa AL, Williams JD, Liu Z, Moriyama T, Valhuerdi A, Prince MJ. Prevalence of stroke and related burden among older people living in Latin America, India and China. J Neurol Neurosurg Psychiatr. 2011; 82(10): 1074-82
Stroke	Sienkiewicz-Jarosz H, Gluszkiewicz M, Pniewski J, Niewada M, Czlonkowska A, Wolfe C, Ryglewicz D. Incidence and case fatality rates of first-ever stroke - comparison of data from two prospective population-based studies conducted in Warsaw. Neurol Neurochir Pol. 2011; 45(3): 207-12
Stroke	Wawrzynczyk M, Pierzchala K, Braczkowska B, Manka-Gaca I, Kumor K, Borowski D, Grodzicka-Zawisza L, Zejda J. Estimates of stroke incidence and case fatality in Zabrze, 2005-2006. Neurol Neurochir Pol. 2011; 45(1): 3-10
Stroke	Leyden JM, Kleinig TJ, Newbury J, Castle S, Cranefield J, Anderson CS, Crotty M, Whitford D, Jannes J, Lee A, Greenhill J. Adelaide stroke incidence study: declining stroke rates but many preventable cardioembolic strokes. Stroke. 2013; 44(5): 1226-31
Stroke	Ministry of Health (Nicaragua), National Institute for Development Information (Nicaragua). Nicaragua National Demographic and Health Survey 2011-2012. Managua, Nicaragua: National Institute for Development Information (Nicaragua)
Stroke	Robert Koch Institute. Germany Health Update 2009-2010. Berlin, Germany: Robert Koch Institute
Stroke	Jucha R. Stroke incidence and casefatality rates in population of Krosno County. Przegl Lek. 2013; 70(4): 191-4
Stroke	Statistics Canada. Canada Community Health Survey 2005. Ottawa, Canada: Statistics Canada
Stroke	Statistics Canada. Canada Community Health Survey 2007-2008. Ottawa, Canada: Statistics Canada, 2009
Stroke	Katzenellenbogen JM, Vos T, Somerford P, Begg S, Semmens JB, Codde JP. Excess Mortality Rates for Estimating the Non-Fatal Burden of Stroke in Western Australia: A Data Linkage Study. Cerebrovasc Dis. 2010; 30(1): 57-64
Stroke	Chiuve SE, Rexrode KM, Spiegelman D, Logroscino G, Manson JE, Rimm EB. Primary prevention of stroke by healthy lifestyle. Circulation. 2008; 118(9): 947–954
Stroke	Autenrieth CS, Evenson KR, Yatsuya H, Shahar E, Baggett C, Rosamond WD. Association between physical activity and risk of stroke subtypes: the atherosclerosis risk in communities study. Neuroepidemiology. 2013; 40(2): 109–116
Stroke	Myint PK, Luben RN, Wareham NJ, Welch AA, Bingham SA, Day NE, Khaw K-T. Combined work and leisure physical activity and risk of stroke in men and women in the European prospective investigation into Cancer-Norfolk Prospective Population Study. Neuroepidemiology. 2006; 27(3): 122–129
Stroke	Sattelmair JR, Kurth T, Buring JE, Lee I-M. Physical activity and risk of stroke in women. Stroke. 2010; 41(6): 1243–50
Stroke	Willey JZ, Moon YP, Paik MC, Boden-Albala B, Sacco RL, Elkind MSV. Physical activity and risk of ischemic stroke in the Northern Manhattan Study. Neurology. 2009; 73(21): 1774–1779

Stroke	Zhang Q, Zhou Y, Gao X, Wang C, Zhang S, Wang A, Li N, Bian L, Wu J, Jia Q, Wu S, Zhao X. Ideal cardiovascular health metrics and the risks of ischemic and intracerebral hemorrhagic stroke. Stroke. 2013; 44(9): 2451–2456
Stroke	Clinton Health Access Initiative (CHAI), Institute for Health Metrics and Evaluation (IHME), Ministry of Health (Zambia), University of Zambia. Access, Bottlenecks, Costs, and Equity (ABCE) project in Zambia, 2011-2012. Seattle, United States: Institute for Health Metrics and Evaluation (IHME), 2015
Stroke	Hu G, Sarti C, Jousilahti P, Silventoinen K, Barengo NC, Tuomilehto J. Leisure time, occupational, and commuting physical activity and the risk of stroke. Stroke. 2005; 36(9): 1994–9
Stroke	TQA Research, World Health Organization (WHO). Australia WHO Multi-country Survey Study on Health and Health System Responsiveness 2000-2001. Geneva, Switzerland: World Health Organization (WHO)
Stroke	Environics Research Group, World Health Organization (WHO). Canada WHO Multi-country Survey Study on Health and Health System Responsiveness 2000-2001. Geneva, Switzerland: World Health Organization (WHO)
Stroke	International Research Associates (INRA) Europe, World Health Organization (WHO). Germany WHO Multi-country Survey Study on Health and Health System Responsiveness 2000-2001. Geneva, Switzerland: World Health Organization (WHO)
Stroke	Public Opinion Research Center (CBOS) (Poland), World Health Organization (WHO). Poland WHO Multi-country Survey Study on Health and Health System Responsiveness 2000-2001. Geneva, Switzerland: World Health Organization (WHO)
Stroke	International Research Associates (INRA) Europe, World Health Organization (WHO). Portugal WHO Multi-country Survey Study on Health and Health System Responsiveness 2000-2001. Geneva, Switzerland: World Health Organization (WHO)
Stroke	Börsch-Supan, A. (2015). Survey of Health, Ageing and Retirement in Europe (SHARE) Wave 5. Release version: 1.0.0. SHARE-ERIC. Data set. DOI: 10.6103/SHARE.w5.100
Stroke	National Team for the Acceleration of Poverty Reduction (TNP2K) (Indonesia), SurveyMETER, University of Southern California, World Bank. Indonesia Family Life Survey East 2012
Stroke	Concluzia-Prim Center for Survey Methodology (Moldova), Independent Sociology and Information Service (OPINIA) (Moldova), Institute for Advanced Studies (Austria), London School of Hygiene and Tropical Medicine, University of Aberdeen. Moldova Health in Times of Transition Household Survey 2010
Stroke	Calling S, Hedblad B, Engström G, Berglund G, Janzon L. Effects of body fatness and physical activity on cardiovascular risk: risk prediction using the bioelectrical impedance method. Scand J Public Health. 2006; 34(6): 568-75
Stroke	Gulsvik AK, Thelle DS, Samuelsen SO, Myrstad M, Mowé M, Wyller TB. Ageing, physical activity and mortality—a 42-year follow-up study. Int J Epidemiol. 2012-41(2): 521-30
Stroke	Ministry of Rural Development (Mali), National Institute of Statistics (INSTAT) (Mali), World Bank. Mali Agricultural Integrated Economic Survey 2014-2015. Washington DC, United States: World Bank
Stroke	RAND Corporation, SurveyMETER. Indonesia Family Life Survey 2014-2015. Santa Monica, United States: RAND Corporation, 2016
Stroke	Ministry of Health (Portugal). Portugal Hospital Inpatient Discharges 2015
Stroke	Ministry of Health (Poland), National Institute of Public Health-National Institute of Hygiene (NIPH-NIH) (Poland). Poland Hospital Inpatient Discharges 2003
Stroke	Ministry of Health (Poland), National Institute of Public Health-National Institute of Hygiene (NIPH-NIH) (Poland). Poland Hospital Inpatient Discharges 2004
Stroke	Federal Statistical Office (Germany). Germany Federal Health Reporting Hospital Discharges 2000
Stroke	Federal Statistical Office (Germany). Germany Federal Health Reporting Hospital Discharges 2001
Stroke	Federal Statistical Office (Germany). Germany Federal Health Reporting Hospital Discharges 2002
Stroke	Federal Statistical Office (Germany). Germany Federal Health Reporting Hospital Discharges 1990
Stroke	Federal Statistical Office (Germany). Germany Federal Health Reporting Hospital Discharges 1991
Stroke	Federal Statistical Office (Germany). Germany Federal Health Reporting Hospital Discharges 1992
Stroke	Federal Statistical Office (Germany). Germany Federal Health Reporting Hospital Discharges 1993
Stroke	Federal Statistical Office (Germany). Germany Federal Health Reporting Hospital Discharges 1994
Stroke	Federal Statistical Office (Germany). Germany Federal Health Reporting Hospital Discharges 1995
Stroke	Federal Statistical Office (Germany). Germany Federal Health Reporting Hospital Discharges 1996
Stroke	Federal Statistical Office (Germany). Germany Federal Health Reporting Hospital Discharges 1997
Stroke	Federal Statistical Office (Germany). Germany Federal Health Reporting Hospital Discharges 1998
Stroke	Federal Statistical Office (Germany). Germany Federal Health Reporting Hospital Discharges 1999
Stroke	Federal Statistical Office (Germany). Germany Federal Health Reporting Hospital Discharges 2013
Stroke	Federal Statistical Office (Germany). Germany Federal Health Reporting Hospital Discharges 2014
Stroke	Statistics Portugal. Portugal Hospital Inpatient Discharges 1985
Stroke	Statistics Portugal. Portugal Hospital Inpatient Discharges 1986
Stroke	Statistics Portugal. Portugal Hospital Inpatient Discharges 1987
Stroke	Statistics Portugal. Portugal Hospital Inpatient Discharges 1988
Stroke	Statistics Portugal. Portugal Hospital Inpatient Discharges 1989
Stroke	Statistics Portugal. Portugal Hospital Inpatient Discharges 1990
Stroke	Statistics Portugal. Portugal Hospital Inpatient Discharges 1991
Stroke	Statistics Portugal. Portugal Hospital Inpatient Discharges 1992
Stroke	Statistics Portugal. Portugal Hospital Inpatient Discharges 1993
Stroke	Statistics Portugal. Portugal Hospital Inpatient Discharges 1994
Stroke	Statistics Portugal. Portugal Hospital Inpatient Discharges 1995
Stroke	Statistics Portugal. Portugal Hospital Inpatient Discharges 1996
Stroke	Statistics Portugal. Portugal Hospital Inpatient Discharges 1997
Stroke	Statistics Portugal. Portugal Hospital Inpatient Discharges 1998
Stroke	Statistics Portugal. Portugal Hospital Inpatient Discharges 1999
Stroke	Statistics Portugal. Portugal Hospital Inpatient Discharges 2000
Stroke	Statistics Portugal. Portugal Hospital Inpatient Discharges 2001
Stroke	Statistics Portugal. Portugal Hospital Inpatient Discharges 2002
Stroke	Statistics Portugal. Portugal Hospital Inpatient Discharges 2003
Stroke	Statistics Portugal. Portugal Hospital Inpatient Discharges 2004
Stroke	Statistics Portugal. Portugal Hospital Inpatient Discharges 2005
Stroke	Statistics Portugal. Portugal Hospital Inpatient Discharges 2006
Stroke	Statistics Portugal. Portugal Hospital Inpatient Discharges 2007
Stroke	Statistics Portugal. Portugal Hospital Inpatient Discharges 2008
Stroke	Statistics Portugal. Portugal Hospital Inpatient Discharges 2009

Stroke	Statistics Portugal. Portugal Hospital Inpatient Discharges 2010
Stroke	Statistics Portugal. Portugal Hospital Inpatient Discharges 2011
Stroke	Statistics Portugal. Portugal Hospital Inpatient Discharges 2012
Stroke	Statistics Portugal. Portugal Hospital Inpatient Discharges 2013
Stroke	Statistics Portugal. Portugal Hospital Inpatient Discharges 2014
Stroke	Ministry of Health (Poland). Poland Hospital Inpatient Discharges 2002
Stroke	Ministry of Health (Poland). Poland Hospital Inpatient Discharges 2001
Stroke	Ministry of Health (Poland). Poland Hospital Inpatient Discharges 2000
Stroke	Ministry of Health (Poland). Poland Hospital Inpatient Discharges 1999
Stroke	Ministry of Health (Poland). Poland Hospital Inpatient Discharges 1998
Stroke	Ministry of Health (Poland). Poland Hospital Inpatient Discharges 1997
Stroke	Ministry of Health (Poland). Poland Hospital Inpatient Discharges 1996
Stroke	Ministry of Health (Poland). Poland Hospital Inpatient Discharges 1995
Stroke	Ministry of Health (Poland). Poland Hospital Inpatient Discharges 1994
Stroke	Ministry of Health (Poland). Poland Hospital Inpatient Discharges 1993
Stroke	Ministry of Health (Poland). Poland Hospital Inpatient Discharges 1992
Stroke	Ministry of Health (Poland). Poland Hospital Inpatient Discharges 1991
Stroke	Ministry of Health (Poland). Poland Hospital Inpatient Discharges 1990
Stroke	Ministry of Health (Poland). Poland Hospital Inpatient Discharges 1989
Stroke	Ministry of Health (Poland). Poland Hospital Inpatient Discharges 1988
Stroke	Ministry of Health (Poland). Poland Hospital Inpatient Discharges 1987
Stroke	Ministry of Health (Poland). Poland Hospital Inpatient Discharges 1986
Stroke	Ministry of Health (Poland). Poland Hospital Inpatient Discharges 1985
Stroke	Ministry of Health (Poland). Poland Hospital Inpatient Discharges 1984
Stroke	Ministry of Health (Poland). Poland Hospital Inpatient Discharges 1983
Stroke	Ministry of Health (Poland). Poland Hospital Inpatient Discharges 1982
Stroke	Ministry of Health (Poland). Poland Hospital Inpatient Discharges 1981
Stroke	Ministry of Health (Poland). Poland Hospital Inpatient Discharges 1980
Stroke	Ministry of Health (Poland). Poland Hospital Inpatient Discharges 2013
Stroke	Ministry of Health (Poland). Poland Hospital Inpatient Discharges 2014
Stroke	Census and Statistics Directorate (Panama), Ministry of Economy and Finance (Panama), World Bank. Panama Living Standard Measurement Survey 2003. Washington DC, United States: World Bank
Stroke	Carolina Population Center, University of North Carolina at Chapel Hill, Institute of Sociology, Russian Academy of Sciences, National Research University Higher School of Economics (Russia), ZAO Demoscope. Russia Longitudinal Monitoring Survey of HSE, Round II 1992-1993
Stroke	Carolina Population Center, University of North Carolina at Chapel Hill, Institute of Sociology, Russian Academy of Sciences, National Research University Higher School of Economics (Russia), ZAO Demoscope. Russia Longitudinal Monitoring Survey of HSE, Round IV 1993-1994
Stroke	Russia Longitudinal Monitoring Survey (RLMS-HSE), Round VII 1996. National Research University Higher School of Economics, ZAO Demoscope, Carolina Population Center, Univeristy of North Carolina at Chapel Hill, Institute of Sociology, Russian Academy of Sciences
Stroke	Russia Longitudinal Monitoring Survey (RLMS-HSE), Round VIII 1998-1999. National Research University Higher School of Economics, ZAO Demoscope, Carolina Population Center, Univeristy of North Carolina at Chapel Hill, Institute of Sociology, Russian Academy of Sciences
Stroke	Russia Longitudinal Monitoring Survey (RLMS-HSE), Round X 2001. National Research University Higher School of Economics, ZAO Demoscope, Carolina Population Center, Univeristy of North Carolina at Chapel Hill, Institute of Sociology, Russian Academy of Sciences
Stroke	Russia Longitudinal Monitoring Survey (RLMS-HSE), Round XI 2002. National Research University Higher School of Economics, ZAO Demoscope, Carolina Population Center, Univeristy of North Carolina at Chapel Hill, Institute of Sociology, Russian Academy of Sciences
Stroke	Russia Longitudinal Monitoring Survey (RLMS-HSE), Round XII 2003. National Research University Higher School of Economics, ZAO Demoscope, Carolina Population Center, Univeristy of North Carolina at Chapel Hill, Institute of Sociology, Russian Academy of Sciences
Stroke	Russia Longitudinal Monitoring Survey (RLMS-HSE), Round XIII 2004. National Research University Higher School of Economics, ZAO Demoscope, Carolina Population Center, Univeristy of North Carolina at Chapel Hill, Institute of Sociology, Russian Academy of Sciences
Stroke	Russia Longitudinal Monitoring Survey (RLMS-HSE), Round XIV 2005. National Research University Higher School of Economics, ZAO Demoscope, Carolina Population Center, Univeristy of North Carolina at Chapel Hill, Institute of Sociology, Russian Academy of Sciences
Stroke	Ministry of Health, Social Services and Equality (Spain), National Statistics Institute (Spain). Spain National Health Survey 2006-2007
Stroke	Health and Retirement Study, (Biennial 1992) public use dataset. Produced and distributed by the University of Michigan with funding from the National Institute on Aging (grant number NIA U01AG009740). Ann Arbor, MI, (2011)
Stroke	and Prevention. National Center for Health Statistics. National Health Interview Survey, 1994: Second Longitudinal Study on Aging, Wave 2, 1997. ICPSR03526-v2. Ann Arbor, MI: Inter-university Consortium for Political and Social Research [distributor], 2007-03-01. http://doi.org/10.3886/ICPSR03526.v2
Stroke	World Health Organization (WHO). Ecuador World Health Survey 2003. Geneva, Switzerland: World Health Organization (WHO), 2005
Stroke	World Health Organization (WHO). Greece World Health Survey 2003. Geneva, Switzerland: World Health Organization (WHO), 2005
Stroke	World Health Organization (WHO). Laos World Health Survey 2003
Stroke	World Health Organization (WHO). Malawi World Health Survey 2003. Geneva, Switzerland: World Health Organization (WHO), 2005
Stroke	World Health Organization (WHO). Mauritania World Health Survey 2003. Geneva, Switzerland: World Health Organization (WHO), 2005
Stroke	World Health Organization (WHO). Russia World Health Survey 2003. Geneva, Switzerland: World Health Organization (WHO), 2005
Stroke	World Health Organization (WHO). Spain World Health Survey 2002-2003. Geneva, Switzerland: World Health Organization (WHO), 2005
Stroke	National Center for Health Statistics (NCHS), Centers for Disease Control and Prevention (CDC). United States National Health and Nutrition Examination Survey 2007-2008. Hyattsville, United States: National Center for Health Statistics (NCHS), Centers for Disease Control and Prevention (CDC), 2009
Stroke	National Center for Health Statistics (NCHS), Centers for Disease Control and Prevention (CDC), US Census Bureau. United States National Health Interview Survey 1990. Hyattsville, United States: National Center for Health Statistics (NCHS), Centers for Disease Control and Prevention (CDC)
Stroke	National Center for Health Statistics (NCHS), Centers for Disease Control and Prevention (CDC), US Census Bureau. United States National Health Interview Survey 1991. Hyattsville, United States: National Center for Health Statistics (NCHS), Centers for Disease Control and Prevention (CDC)
Stroke	National Center for Health Statistics (NCHS), Centers for Disease Control and Prevention (CDC), US Census Bureau. United States National Health Interview Survey 1992. Hyattsville, United States: National Center for Health Statistics (NCHS), Centers for Disease Control and Prevention (CDC)
Stroke	National Center for Health Statistics (NCHS), Centers for Disease Control and Prevention (CDC), US Census Bureau. United States National Health Interview Survey 1993. Hyattville, United States: National Center for Health Statistics (NCHS), Centers for Disease Control and Prevention (CDC)

Stroke	National Center for Health Statistics (NCHS), Centers for Disease Control and Prevention (CDC), US Census Bureau. United States National Health Interview Survey 1997. Hyattsville, United States: National Center for Health Statistics (NCHS), Centers for Disease Control and Prevention (CDC)
Stroke	National Center for Health Statistics (NCHS), Centers for Disease Control and Prevention (CDC), US Census Bureau. United States National Health Interview Survey 1998. Hyattsville, United States: National Center for Health Statistics (NCHS), Centers for Disease Control and Prevention (CDC)
Stroke	National Center for Health Statistics (NCHS), Centers for Disease Control and Prevention (CDC), US Census Bureau. United States National Health Interview Survey 1999. Hyattsville, United States: National Center for Health Statistics (NCHS), Centers for Disease Control and Prevention (CDC)
Stroke	National Center for Health Statistics (NCHS), Centers for Disease Control and Prevention (CDC), US Census Bureau. United States National Health Interview Survey 2000. Hyattsville, United States: National Center for Health Statistics (NCHS), Centers for Disease Control and Prevention (CDC)
Stroke	National Center for Health Statistics (NCHS), Centers for Disease Control and Prevention (CDC), US Census Bureau. United States National Health Interview Survey 2001. Hyattsville, United States: National Center for Health Statistics (NCHS), Centers for Disease Control and Prevention (CDC)
Stroke	National Center for Health Statistics (NCHS), Centers for Disease Control and Prevention (CDC), US Census Bureau. United States National Health Interview Survey 2002. Hyattsville, United States: National Center for Health Statistics (NCHS), Centers for Disease Control and Prevention (CDC)
Stroke	National Center for Health Statistics (NCHS), Centers for Disease Control and Prevention (CDC), US Census Bureau. United States National Health Interview Survey 2003. Hyattsville, United States: National Center for Health Statistics (NCHS), Centers for Disease Control and Prevention (CDC)
Stroke	National Center for Health Statistics (NCHS), Centers for Disease Control and Prevention (CDC), US Census Bureau. United States National Health Interview Survey 2004. Hyattsville, United States: National Center for Health Statistics (NCHS), Centers for Disease Control and Prevention (CDC)
Stroke	National Center for Health Statistics (NCHS), Centers for Disease Control and Prevention (CDC), US Census Bureau. United States National Health Interview Survey 2005. Hyattsville, United States: National Center for Health Statistics (NCHS), Centers for Disease Control and Prevention (CDC)
Stroke	National Center for Health Statistics (NCHS), Centers for Disease Control and Prevention (CDC), US Census Bureau. United States National Health Interview Survey 2006. Hyattsville, United States: National Center for Health Statistics (NCHS), Centers for Disease Control and Prevention (CDC)
Stroke	National Center for Health Statistics (NCHS), Centers for Disease Control and Prevention (CDC), US Census Bureau. United States National Health Interview Survey 2007. Hyattsville, United States: National Center for Health Statistics (NCHS), Centers for Disease Control and Prevention (CDC)
Stroke	National Center for Health Statistics (NCHS), Centers for Disease Control and Prevention (CDC), US Census Bureau. United States National Health Interview Survey 2008. Hyattsville, United States: National Center for Health Statistics (NCHS), Centers for Disease Control and Prevention (CDC)
Stroke	National Center for Health Statistics (NCHS), Centers for Disease Control and Prevention (CDC), US Census Bureau. United States National Health Interview Survey 2009. Hyattsville, United States: National Center for Health Statistics (NCHS), Centers for Disease Control and Prevention (CDC)
Stroke	National Center for Health Statistics (NCHS), Centers for Disease Control and Prevention (CDC), US Census Bureau. United States National Health Interview Survey 2010. Hyattsville, United States: National Center for Health Statistics (NCHS), Centers for Disease Control and Prevention (CDC)
Stroke	National Statistical Office of Malawi, World Bank. Malawi Living Standards Measurement Survey 2004-2005
Stroke	National Center for Health Statistics (NCHS), Centers for Disease Control and Prevention (CDC). United States National Health and Nutrition Examination Survey 2005-2006. Hyattsville, United States: National Center for Health Statistics (NCHS), Centers for Disease Control and Prevention (CDC), 2007
Stroke	National Center for Health Statistics (NCHS), Centers for Disease Control and Prevention (CDC). United States National Health and Nutrition Examination Survey 2003-2004. Hyattsville, United States: National Center for Health Statistics (NCHS), Centers for Disease Control and Prevention (CDC)
Stroke	National Center for Health Statistics (NCHS), Centers for Disease Control and Prevention (CDC). United States National Health and Nutrition Examination Survey 2009-2010. Hyattsville, United States: National Center for Health Statistics (NCHS), Centers for Disease Control and Prevention (CDC), 2011
Stroke	National Center for Health Statistics (NCHS), Centers for Disease Control and Prevention (CDC). United States National Health and Nutrition Examination Survey 1988-1994. Hyattsville, United States: National Center for Health Statistics (NCHS), Centers for Disease Control and Prevention (CDC)
Stroke	National Center for Health Statistics (NCHS), Centers for Disease Control and Prevention (CDC). United States National Health and Nutrition Examination Survey 2001-2002. Hyattsville, United States: National Center for Health Statistics (NCHS), Centers for Disease Control and Prevention (CDC)
Stroke	National Center for Health Statistics (NCHS), Centers for Disease Control and Prevention (CDC). United States National Health and Nutrition Examination Survey 1999-2000. Hyattsville, United States: National Center for Health Statistics (NCHS), Centers for Disease Control and Prevention (CDC)
Stroke	Health and Retirement Study, (Biennial 1996) public use dataset. Produced and distributed by the University of Michigan with funding from the National Institute on Aging (grant number NIA U01AG009740). Ann Arbor, MI, (2011)
Stroke	Health and Retirement Study, (Biennial 1998) public use dataset. Produced and distributed by the University of Michigan with funding from the National Institute on Aging (grant number NIA U01AG009740). Ann Arbor, MI, (2014)
Stroke	Health and Retirement Study, (Biennial 2000) public use dataset. Produced and distributed by the University of Michigan with funding from the National Institute on Aging (grant number NIA U01AG009740). Ann Arbor, MI, (2011)
Stroke	Health and Retirement Study, (Biennial 2002) public use dataset. Produced and distributed by the University of Michigan with funding from the National Institute on Aging (grant number NIA U01AG009740). Ann Arbor, MI, (2011)
Stroke	Health and Retirement Study, (Biennial 2004) public use dataset. Produced and distributed by the University of Michigan with funding from the National Institute on Aging (grant number NIA U01AG009740). Ann Arbor, MI, (2013)
Stroke	Health and Retirement Study, (Biennial 2006) public use dataset. Produced and distributed by the University of Michigan with funding from the National Institute on Aging (grant number NIA U01AG009740). Ann Arbor, MI, (2014)
Stroke	Health and Retirement Study, (Biennial 2010) public use dataset. Produced and distributed by the University of Michigan with funding from the National Institute on Aging (grant number NIA U01AG009740). Ann Arbor, MI, (2014)
Stroke	Panel Study of Income Dynamics, 2007 public use dataset. Produced and distributed by the University of Michigan with primary funding from the National Science Foundation, the National Institute of Aging, and the National Institute of Child Health and Human Development. Ann Arbor, MI, (2011)
Stroke	Panel Study of Income Dynamics, 2005 public use dataset. Produced and distributed by the University of Michigan with primary funding from the National Science Foundation, the National Institute of Aging, and the National Institute of Child Health and Human Development. Ann Arbor, MI, (2011)
Stroke	National Center for Health Statistics (NCHS), Centers for Disease Control and Prevention (CDC), United States Census Bureau. United States National Hospital Discharge Survey 1996. Hyattsville, United States: National Center for Health Statistics (NCHS), Centers for Disease Control and Prevention (CDC)
Stroke	National Center for Health Statistics (NCHS), Centers for Disease Control and Prevention (CDC), United States Census Bureau. United States National Hospital Discharge Survey 1997. Hyattsville, United States: National Center for Health Statistics (NCHS), Centers for Disease Control and Prevention (CDC)

Stroke	National Center for Health Statistics (NCHS), Centers for Disease Control and Prevention (CDC), United States Census Bureau. United States National Hospital Discharge Survey 1998. Hyattsville, United States: National Center for Health Statistics (NCHS), Centers for Disease Control and Prevention (CDC)
Stroke	National Center for Health Statistics (NCHS), Centers for Disease Control and Prevention (CDC), United States Census Bureau. United States National Hospital Discharge Survey 1999. Hyattsville, United States: National Center for Health Statistics (NCHS), Centers for Disease Control and Prevention (CDC)
Stroke	National Center for Health Statistics (NCHS), Centers for Disease Control and Prevention (CDC), United States Census Bureau. United States National Hospital Discharge Survey 2000. Hyattsville, United States: National Center for Health Statistics (NCHS), Centers for Disease Control and Prevention (CDC)
Stroke	National Center for Health Statistics (NCHS), Centers for Disease Control and Prevention (CDC), United States Census Bureau. United States National Hospital Discharge Survey 2001. Hyattsville, United States: National Center for Health Statistics (NCHS), Centers for Disease Control and Prevention (CDC)
Stroke	National Center for Health Statistics (NCHS), Centers for Disease Control and Prevention (CDC), United States Census Bureau. United States National Hospital Discharge Survey 2002. Hyattsville, United States: National Center for Health Statistics (NCHS), Centers for Disease Control and Prevention (CDC)
Stroke	National Center for Health Statistics (NCHS), Centers for Disease Control and Prevention (CDC), United States Census Bureau. United States National Hospital Discharge Survey 2003. Hyattsville, United States: National Center for Health Statistics (NCHS), Centers for Disease Control and Prevention (CDC)
Stroke	National Center for Health Statistics (NCHS), Centers for Disease Control and Prevention (CDC), United States Census Bureau. United States National Hospital Discharge Survey 2004. Hyattsville, United States: National Center for Health Statistics (NCHS), Centers for Disease Control and Prevention (CDC)
Stroke	National Center for Health Statistics (NCHS), Centers for Disease Control and Prevention (CDC), United States Census Bureau. United States National Hospital Discharge Survey 2005. Hyattsville, United States: National Center for Health Statistics (NCHS), Centers for Disease Control and Prevention (CDC)
Stroke	National Center for Health Statistics (NCHS), Centers for Disease Control and Prevention (CDC), United States Census Bureau. United States National Hospital Discharge Survey 2006. Hyattsville, United States: National Center for Health Statistics (NCHS), Centers for Disease Control and Prevention (CDC)
Stroke	National Center for Health Statistics (NCHS), Centers for Disease Control and Prevention (CDC), United States Census Bureau. United States National Hospital Discharge Survey 2007. Hyattsville, United States: National Center for Health Statistics (NCHS), Centers for Disease Control and Prevention (CDC)
Stroke	National Center for Health Statistics (NCHS), Centers for Disease Control and Prevention (CDC), United States Census Bureau. United States National Hospital Discharge Survey 2008. Hyattsville, United States: National Center for Health Statistics (NCHS), Centers for Disease Control and Prevention (CDC)
Stroke	National Center for Health Statistics (NCHS), Centers for Disease Control and Prevention (CDC), United States Census Bureau. United States National Hospital Discharge Survey 2009. Hyattsville, United States: National Center for Health Statistics (NCHS), Centers for Disease Control and Prevention (CDC)
Stroke	National Center for Health Statistics (NCHS), Centers for Disease Control and Prevention (CDC), United States Census Bureau. United States National Hospital Discharge Survey 2010. Hyattsville, United States: National Center for Health Statistics (NCHS), Centers for Disease Control and Prevention (CDC)
Stroke	National Center for Health Statistics (NCHS), Centers for Disease Control and Prevention (CDC), United States Census Bureau. United States National Hospital Discharge Survey 1979. Hyattsville, United States: National Center for Health Statistics (NCHS), Centers for Disease Control and Prevention (CDC)
Stroke	National Center for Health Statistics (NCHS), Centers for Disease Control and Prevention (CDC), United States Census Bureau. United States National Hospital Discharge Survey 1980. Hyattsville, United States: National Center for Health Statistics (NCHS), Centers for Disease Control and Prevention (CDC)
Stroke	National Center for Health Statistics (NCHS), Centers for Disease Control and Prevention (CDC), United States Census Bureau. United States National Hospital Discharge Survey 1981. Hyattsville, United States: National Center for Health Statistics (NCHS), Centers for Disease Control and Prevention (CDC)
Stroke	National Center for Health Statistics (NCHS), Centers for Disease Control and Prevention (CDC), United States Census Bureau. United States National Hospital Discharge Survey 1982. Hyattsville, United States: National Center for Health Statistics (NCHS), Centers for Disease Control and Prevention (CDC)
Stroke	National Center for Health Statistics (NCHS), Centers for Disease Control and Prevention (CDC), United States Census Bureau. United States National Hospital Discharge Survey 1983. Hyattsville, United States: National Center for Health Statistics (NCHS), Centers for Disease Control and Prevention (CDC)
Stroke	National Center for Health Statistics (NCHS), Centers for Disease Control and Prevention (CDC), United States Census Bureau. United States National Hospital Discharge Survey 1984. Hyattsville, United States: National Center for Health Statistics (NCHS), Centers for Disease Control and Prevention (CDC)
Stroke	National Center for Health Statistics (NCHS), Centers for Disease Control and Prevention (CDC), United States Census Bureau. United States National Hospital Discharge Survey 1985. Hyattsville, United States: National Center for Health Statistics (NCHS), Centers for Disease Control and Prevention (CDC)
Stroke	National Center for Health Statistics (NCHS), Centers for Disease Control and Prevention (CDC), United States Census Bureau. United States National Hospital Discharge Survey 1986. Hyattsville, United States: National Center for Health Statistics (NCHS), Centers for Disease Control and Prevention (CDC)
Stroke	National Center for Health Statistics (NCHS), Centers for Disease Control and Prevention (CDC), United States Census Bureau. United States National Hospital Discharge Survey 1987. Hyattsville, United States: National Center for Health Statistics (NCHS), Centers for Disease Control and Prevention (CDC)
Stroke	National Center for Health Statistics (NCHS), Centers for Disease Control and Prevention (CDC), United States Census Bureau. United States National Hospital Discharge Survey 1988. Hyattsville, United States: National Center for Health Statistics (NCHS), Centers for Disease Control and Prevention (CDC)
Stroke	National Center for Health Statistics (NCHS), Centers for Disease Control and Prevention (CDC), United States Census Bureau. United States National Hospital Discharge Survey 1989. Hyattsville, United States: National Center for Health Statistics (NCHS), Centers for Disease Control and Prevention (CDC)
Stroke	National Center for Health Statistics (NCHS), Centers for Disease Control and Prevention (CDC), United States Census Bureau. United States National Hospital Discharge Survey 1990. Hyattsville, United States: National Center for Health Statistics (NCHS), Centers for Disease Control and Prevention (CDC)

Stroke	National Center for Health Statistics (NCHS), Centers for Disease Control and Prevention (CDC), United States Census Bureau. United States National Hospital Discharge Survey 1991. Hyattsville, United States: National Center for Health Statistics (NCHS), Centers for Disease Control and Prevention (CDC)
Stroke	National Center for Health Statistics (NCHS), Centers for Disease Control and Prevention (CDC), United States Census Bureau. United States National Hospital Discharge Survey 1992. Hyattsville, United States: National Center for Health Statistics (NCHS), Centers for Disease Control and Prevention (CDC)
Stroke	National Center for Health Statistics (NCHS), Centers for Disease Control and Prevention (CDC), United States Census Bureau. United States National Hospital Discharge Survey 1993. Hyattsville, United States: National Center for Health Statistics (NCHS), Centers for Disease Control and Prevention (CDC)
Stroke	National Center for Health Statistics (NCHS), Centers for Disease Control and Prevention (CDC), United States Census Bureau. United States National Hospital Discharge Survey 1994. Hyattsville, United States: National Center for Health Statistics (NCHS), Centers for Disease Control and Prevention (CDC)
Stroke	National Center for Health Statistics (NCHS), Centers for Disease Control and Prevention (CDC), United States Census Bureau. United States National Hospital Discharge Survey 1995. Hyattsville, United States: National Center for Health Statistics (NCHS), Centers for Disease Control and Prevention (CDC)
Stroke	National Institute of Statistics and Censuses (Ecuador). Ecuador Hospital Inpatient Discharges 1997. Quito, Ecuador: National Institute of Statistics and Censuses (Ecuador)
Stroke	National Institute of Statistics and Censuses (Ecuador). Ecuador Hospital Inpatient Discharges 1998. Quito, Ecuador: National Institute of Statistics and Censuses (Ecuador)
Stroke	National Institute of Statistics and Censuses (Ecuador). Ecuador Hospital Inpatient Discharges 1999. Quito, Ecuador: National Institute of Statistics and Censuses (Ecuador)
Stroke	National Institute of Statistics and Censuses (Ecuador). Ecuador Hospital Inpatient Discharges 2000. Quito, Ecuador: National Institute of Statistics and Censuses (Ecuador)
Stroke	National Institute of Statistics and Censuses (Ecuador). Ecuador Hospital Inpatient Discharges 2001. Quito, Ecuador: National Institute of Statistics and Censuses (Ecuador)
Stroke	National Institute of Statistics and Censuses (Ecuador). Ecuador Hospital Inpatient Discharges 2002. Quito, Ecuador: National Institute of Statistics and Censuses (Ecuador)
Stroke	National Institute of Statistics and Censuses (Ecuador). Ecuador Hospital Inpatient Discharges 2003. Quito, Ecuador: National Institute of Statistics and Censuses (Ecuador)
Stroke	National Institute of Statistics and Censuses (Ecuador). Ecuador Hospital Inpatient Discharges 2004. Quito, Ecuador: National Institute of Statistics and Censuses (Ecuador)
Stroke	National Institute of Statistics and Censuses (Ecuador). Ecuador Hospital Inpatient Discharges 2005. Quito, Ecuador: National Institute of Statistics and Censuses (Ecuador)
Stroke	National Institute of Statistics and Censuses (Ecuador). Ecuador Hospital Inpatient Discharges 2006. Quito, Ecuador: National Institute of Statistics and Censuses (Ecuador)
Stroke	National Institute of Statistics and Censuses (Ecuador). Ecuador Hospital Inpatient Discharges 2007. Quito, Ecuador: National Institute of Statistics and Censuses (Ecuador)
Stroke	National Institute of Statistics and Censuses (Ecuador). Ecuador Hospital Inpatient Discharges 2008. Quito, Ecuador: National Institute of Statistics and Censuses (Ecuador)
Stroke	National Institute of Statistics and Censuses (Ecuador). Ecuador Hospital Inpatient Discharges 2009. Quito, Ecuador: National Institute of Statistics and Censuses (Ecuador)
Stroke	National Institute of Statistics and Censuses (Ecuador). Ecuador Hospital Inpatient Discharges 2010. Quito, Ecuador: National Institute of Statistics and Censuses (Ecuador)
Stroke	National Institute of Statistics and Censuses (Ecuador). Ecuador Hospital Inpatient Discharges 2011. Quito, Ecuador: National Institute of Statistics and Censuses (Ecuador)
Stroke	Healthcare Cost and Utilization Project (HCUP), Agency for Healthcare Research and Quality (AHRQ). United States State Inpatient Databases 2003. Rockville, United States: Healthcare Cost and Utilization Project (HCUP), Agency for Healthcare Research and Quality (AHRQ)
Stroke	Healthcare Cost and Utilization Project (HCUP), Agency for Healthcare Research and Quality (AHRQ). United States State Inpatient Databases 2004. Rockville, United States: Healthcare Cost and Utilization Project (HCUP), Agency for Healthcare Research and Quality (AHRQ)
Stroke	Healthcare Cost and Utilization Project (HCUP), Agency for Healthcare Research and Quality (AHRQ). United States State Inpatient Databases 2005. Rockville, United States: Healthcare Cost and Utilization Project (HCUP), Agency for Healthcare Research and Quality (AHRQ)
Stroke	Healthcare Cost and Utilization Project (HCUP), Agency for Healthcare Research and Quality (AHRQ). United States State Inpatient Databases 2006. Rockville, United States: Healthcare Cost and Utilization Project (HCUP), Agency for Healthcare Research and Quality (AHRQ)
Stroke	Healthcare Cost and Utilization Project (HCUP), Agency for Healthcare Research and Quality (AHRQ). United States State Inpatient Databases 2007. Rockville, United States: Healthcare Cost and Utilization Project (HCUP), Agency for Healthcare Research and Quality (AHRQ)
Stroke	Healthcare Cost and Utilization Project (HCUP), Agency for Healthcare Research and Quality (AHRQ). United States State Inpatient Databases 2008. Rockville, United States: Healthcare Cost and Utilization Project (HCUP), Agency for Healthcare Research and Quality (AHRQ)
Stroke	Healthcare Cost and Utilization Project (HCUP), Agency for Healthcare Research and Quality (AHRQ). United States State Inpatient Databases 2009. Rockville, United States: Healthcare Cost and Utilization Project (HCUP), Agency for Healthcare Research and Quality (AHRQ)
Stroke	Health Measurement Research Group, University of Wisconsin. United States National Health Measurement Study 2005-2006. Data and Information Sciences Center, University of Wisconsin-Madison [distributor]
Stroke	National Center for Health Statistics (NCHS), Centers for Disease Control and Prevention (CDC), US Census Bureau. United States National Health Interview Survey 2011. Hyattsville, United States: National Center for Health Statistics (NCHS), Centers for Disease Control and Prevention (CDC)
Stroke	Ministry of Health and Medical Education (Iran), World Health Organization (WHO). Iran WHO Multi-country Survey Study on Health and Health System Responsiveness 2000-2001. Geneva, Switzerland: World Health Organization (WHO)
Stroke	National Statistical Office of Malawi, World Bank. Malawi Integrated Household Survey 2010-2011. Washington DC, United States: World Bank
Stroke	Agency for Healthcare Research and Quality. United States Medical Expenditure Panel Survey 2002-2009. Rockville, United States: Agency for Healthcare Research and Quality
Stroke	Feigin V, Carter K, Hackett M, Barber PA, McNaughton H, Dyall L, Chen M, Anderson C. Ethnic disparities in incidence of stroke subtypes: Auckland Regional Community Stroke Study, 2002-2003. Lancet Neurol. 2006; 5(2): 130-9
Stroke	Feigin VL, Wiebers DO, Nikitin YP, O'Fallon WM, Whisnant JP. Stroke Epidemiology in Novosibirsk, Russia: A Population-Based Study. Mayo Clin Proc. 1995; 70(9): 847-52
Stroke	Azarpazhooh MR, Etemadi MM, Donnan GA, Mokhber N, Majdi MR, Ghayour-Mobarhan M, Ghandehary K, Farzadfard MT, Kiani R, Panahandeh M, Thrift AG. Excessive Incidence of Stroke in Iran. Stroke. 2010; 41(1): e3-e10

Stroke	Vemmos KN, Bots ML, Tsibouris PK, Zis VP, Grobbee DE, Stranjalis GS, Stamatelopoulos S. Stroke Incidence and Case Fatality in Southern Greece: The Arcadia Stroke Registry. Stroke. 1999; 30(2): 363-70
Stroke	al-Rajeh S, Larbi EB, Bademosi O, Awada A, Yousef A, al-Freihi H, Miniawi H. Stroke register: experience from the eastern province of Saudi Arabia. Cerebrovasc Dis. 1998; 8(2): 86-9
Stroke	Al Rajeh S. Stroke in the Elderly Aged 75 Years and Above. Cerebrovasc Dis. 1994; 4(6): 402-6
Stroke	Anderson CS, Carter KN, Hackett ML, Feigin V, Barber PA, Broad JB, Bonita R. Trends in Stroke Incidence in Auckland, New Zealand, During 1981 to 2003. Stroke. 2005; 36(10): 2087-93
Stroke	Awada A. Stroke in Saudi Arabian young adults: a study of 120 cases. Acta Neurol Scand. 1994; 89(5): 323-8
Stroke	Bonita R, Broad JB, Beaglehole R. Changes in stroke incidence and case-fatality in Auckland, New Zealand, 1981-91. Lancet. 1993; 342(8885): 1470-3
Stroke	Earley CJ, Kittner SJ, Feeser BR, Gardner J, Epstein A, Wozniak MA, Wityk R, Stern BJ, Price TR, Macko RF, Johnson C, Sloan MA, Buchholz D. Stroke in children and sickle-cell disease: Baltimore-Washington Cooperative Young Stroke Study. Neurology. 1998; 51(1): 169-76
Stroke	Alzamora MT, Sorribes M, Heras A, Vila N, Vicheto M, Forés R, Sánchez-Ojanguren J, Sancho A, the, Pera G. Ischemic stroke incidence in Santa Coloma de Gramenet (ISISCOG), Spain. A community-based study. BMC Neurol. 2008; 8(1): 5
Stroke	Ghandehari K, Moud ZI. Incidence and etiology of ischemic stroke in Persian young adults. Acta Neurol Scand. 2006; 113(2): 121-4
Stroke	National Center for Health Statistics (NCHS) Centers for Disease Control and Prevention (CDC). United States National Health and Nutrition Examination Survey 2011-2012. Hyattsville, United States: National Center for Health Statistics (NCHS) Centers for Disease Control and Prevention (CDC), 2013
Stroke	National Center for Health Statistics (NCHS), Centers for Disease Control and Prevention (CDC), United States Census Bureau. United States National Health Interview Survey 2012. Hyattsville, United States: National Center for Health Statistics (NCHS), Centers for Disease Control and Prevention (CDC), 2013
Stroke	National Institute of Statistics and Censuses (Ecuador). Ecuador Hospital Inpatient Discharges 2012. Quito, Ecuador: National Institute of Statistics and Censuses (Ecuador), 2013
Stroke	Talaei M, Sarrafzadegan N, Sadeghi M, Oveisgharan S, Marshall T, Thomas GN, Iranipour R. Incidence of cardiovascular diseases in an Iranian population: the Isfahan Cohort Study. Arch Iran Med. 2013; 16(3): 138-44
Stroke	Russia Longitudinal Monitoring Survey (RLMS-HSE), Round XVIII 2009. National Research University Higher School of Economics, ZAO Demoscope, Carolina Population Center, Univeristy of North Carolina at Chapel Hill, Institute of Sociology, Russian Academy of Sciences
Stroke	Russia Longitudinal Monitoring Survey (RLMS-HSE), Round XVII 2008. National Research University Higher School of Economics, ZAO Demoscope, Carolina Population Center, Univeristy of North Carolina at Chapel Hill, Institute of Sociology, Russian Academy of Sciences
Stroke	Russia Longitudinal Monitoring Survey (RLMS-HSE), Round XVI 2007. National Research University Higher School of Economics, ZAO Demoscope, Carolina Population Center, Univeristy of North Carolina at Chapel Hill, Institute of Sociology, Russian Academy of Sciences
Stroke	Russia Longitudinal Monitoring Survey (RLMS-HSE), Round XV 2006. National Research University Higher School of Economics, ZAO Demoscope, Carolina Population Center, Univeristy of North Carolina at Chapel Hill, Institute of Sociology, Russian Academy of Sciences
Stroke	Russia Longitudinal Monitoring Survey (RLMS-HSE), Round VI 1995. National Research University Higher School of Economics, ZAO Demoscope, Carolina Population Center, Univeristy of North Carolina at Chapel Hill, Institute of Sociology, Russian Academy of Sciences
Stroke	Russia Longitudinal Monitoring Survey (RLMS-HSE), Round V 1994. National Research University Higher School of Economics, ZAO Demoscope, Carolina Population Center, Univeristy of North Carolina at Chapel Hill, Institute of Sociology, Russian Academy of Sciences
Stroke	Zahuranec DB, Brown DL, Lisabeth LD, Morgenstern LB. Is it time for a large, collaborative study of pediatric stroke?. Stroke. 2005; 36(9): 1825-9
Stroke	Bonita R, Solomon N, Broad JB. Prevalence of Stroke and Stroke-Related Disability: Estimates From the Auckland Stroke Studies. Stroke. 1997; 28(10): 1898-902
Stroke	Díaz-Guzmán J, Bermejo-Pareja F, Benito-León J, Vega S, Gabriel R, Medrano MJ. Prevalence of stroke and transient ischemic attack in three elderly populations of central Spain. Neuroepidemiology. 2008; 30(4): 247-53
Stroke	Delbari A, Salman Roghani R, Tabatabaei SS, Rahgozar M, Lokk J. Stroke epidemiology and one-month fatality among an urban population in Iran. Int J Stroke. 2011; 6(3): 195-200
Stroke	Delbari A, Salman Roghani R, Tabatabaei SS, Lökk J. A Stroke Study of an Urban Area of Iran: Risk Factors, Length of Stay, Case Fatality, and Discharge Destination. J Stroke Cerebrovasc Dis. 2010; 19(2): 104-9
Stroke	Institute for Health Metrics and Evaluation (IHME), Ministry of Health (Saudi Arabia). Saudi Arabia Health Interview Survey 2013
Stroke	Health and Retirement Study, (Biennial 2012) public use dataset. Produced and distributed by the University of Michigan with funding from the National Institute on Aging (grant number NIA U01AG009740). Ann Arbor, MI, (2015)
Stroke	Ministry of Health, Social Services and Equality (Spain), Sociological Research Center (Spain). Spain Health Barometer Survey 2004. Madrid, Spain: Ministry of Health, Social Services and Equality (Spain), 2004
Stroke	National Center for Health Statistics (NCHS), Centers for Disease Control and Prevention (CDC), United States Census Bureau. United States National Health Interview Survey 2013. Hyattsville, United States: National Center for Health Statistics (NCHS), Centers for Disease Control and Prevention (CDC), 2014
Stroke	National Institute of Statistics and Censuses (Ecuador). Ecuador Hospital Inpatient Discharges 2013. Quito, Ecuador: National Institute of Statistics and Censuses (Ecuador)
Stroke	National Center for Health Statistics (NCHS), Centers for Disease Control and Prevention (CDC). United States National Health and Nutrition Examination Survey 2013-2014. Hyattsville, United States: National Center for Health Statistics (NCHS), Centers for Disease Control and Prevention (CDC)
Stroke	International Research Associates (INRA) Europe, World Health Organization (WHO). Spain WHO Multi-country Survey Study on Health and Health System Responsiveness 2000-2001. Geneva, Switzerland: World Health Organization (WHO)
Stroke	University of Otago (New Zealand), World Health Organization (WHO). New Zealand WHO Multi-country Survey Study on Health and Health System Responsiveness 2000-2001. Geneva, Switzerland: World Health Organization (WHO)
Stroke	International Research Associates (INRA) Europe, World Health Organization (WHO). Romania WHO Multi-country Survey Study on Health and Health System Responsiveness 2000-2001. Geneva, Switzerland: World Health Organization (WHO)
Stroke	International Research Associates (INRA) Europe, World Health Organization (WHO). Russia WHO Multi-country Survey Study on Health and Health System Responsiveness 2000-2001. Geneva, Switzerland: World Health Organization (WHO)
Stroke	Washington State University, World Health Organization (WHO). United States WHO Multi-country Survey Study on Health and Health System Responsiveness 2000-2001. Geneva, Switzerland: World Health Organization (WHO)
Stroke	Börsch-Supan, A. (2015). Survey of Health, Ageing and Retirement in Europe (SHARE) Wave 5. Release version: 1.0.0. SHARE-ERIC. Data set. DOI: 10.6103/SHARE.w5.100
Stroke	National Center for Health Statistics (NCHS), Centers for Disease Control and Prevention (CDC). United States National Health Interview Survey 2014. Hyattsville, United States: National Center for Health Statistics (NCHS), Centers for Disease Control and Prevention (CDC), 2015
Stroke	Ministry of Health (New Zealand). New Zealand National Minimum Dataset 2000
Stroke	Ministry of Health (New Zealand). New Zealand National Minimum Dataset 2001
Stroke	Ministry of Health (New Zealand). New Zealand National Minimum Dataset 2002

Stroke	Ministry of Health (New Zealand). New Zealand National Minimum Dataset 2003
Stroke	Ministry of Health (New Zealand). New Zealand National Minimum Dataset 2004
Stroke	Ministry of Health (New Zealand). New Zealand National Minimum Dataset 2005
Stroke	Ministry of Health (New Zealand). New Zealand National Minimum Dataset 2006
Stroke	Ministry of Health (New Zealand). New Zealand National Minimum Dataset 2007. Wellington, New Zealand: Ministry of Health (New Zealand)
Stroke	Ministry of Health (New Zealand). New Zealand National Minimum Dataset 2008. Wellington, New Zealand: Ministry of Health (New Zealand)
Stroke	Ministry of Health (New Zealand). New Zealand National Minimum Dataset 2009. Wellington, New Zealand: Ministry of Health (New Zealand)
Stroke	Ministry of Health (New Zealand). New Zealand National Minimum Dataset 2010. Wellington, New Zealand: Ministry of Health (New Zealand)
Stroke	Ministry of Health (New Zealand). New Zealand National Minimum Dataset 2011. Wellington, New Zealand: Ministry of Health (New Zealand)
Stroke	Ministry of Health (New Zealand). New Zealand National Minimum Dataset 2012. Wellington, New Zealand: Ministry of Health (New Zealand)
Stroke	Ministry of Health (New Zealand). New Zealand National Minimum Dataset 2013. Wellington, New Zealand: Ministry of Health (New Zealand)
Stroke	Ministry of Health (New Zealand). New Zealand National Minimum Dataset 2014. Wellington, New Zealand: Ministry of Health (New Zealand)
Stroke	National Statistical Office of Malawi. Malawi Integrated Household Survey 2013. Washington DC, United States: World Bank, 2015
Stroke	Truven Health Analytics. United States MarketScan Commercial Claims and Encounters Database 2010. Ann Arbor, United States: Truven Health Analytics
Stroke	Center for Sociological Studies, Lomonosov Moscow State University, Concluzia-Prim Center for Survey Methodology (Moldova), Institute for Advanced Studies (Austria), London School of Hygiene and Tropical Medicine, University of Aberdeen. Russia Health in Times of Transition Household Survey 2010
Stroke	National Center for Health Statistics (NCHS), Centers for Disease Control and Prevention (CDC). United States National Hospital Discharge Survey 1988-1992
Stroke	National Center for Health Statistics (NCHS), Centers for Disease Control and Prevention (CDC). United States National Hospital Discharge Survey 1993-1997
Stroke	National Center for Health Statistics (NCHS), Centers for Disease Control and Prevention (CDC). United States National Hospital Discharge Survey 1998-2002
Stroke	National Center for Health Statistics (NCHS), Centers for Disease Control and Prevention (CDC). United States National Hospital Discharge Survey 2003-2007
Stroke	National Center for Health Statistics (NCHS), Centers for Disease Control and Prevention (CDC). United States National Hospital Discharge Survey 2008-2010
Stroke	National Institute of Statistics and Censuses (Ecuador). Ecuador Hospital Inpatient Discharges 2014. Quito, Ecuador: National Institute of Statistics and Censuses (Ecuador)
Stroke	Healthcare Cost and Utilization Project (HCUP), Agency for Healthcare Research and Quality (AHRQ). United States Nationwide Inpatient Sample 2013. Rockville, United States: Healthcare Cost and Utilization Project (HCUP), Agency for Healthcare Research and Quality (AHRQ)
Stroke	National Center for Health Statistics (NCHS), Centers for Disease Control and Prevention (CDC). United States National Health Interview Survey 2015. Hyattsville, United States: National Center for Health Statistics (NCHS), Centers for Disease Control and Prevention (CDC), 2016
Stroke	and Prevention. National Center for Health Statistics. National Health Interview Survey, 1994: Second Supplement on Aging. ICPSR02563-v3. Ann Arbor, MI: Inter-university Consortium for Political and Social Research [distributor], 2007-02-12. http://doi.org/10.3886/ICPSR02563.v3
Stroke	Healthcare Cost and Utilization Project (HCUP), Agency for Healthcare Research and Quality (AHRQ). United States State Inpatient Databases 2003-2007
Stroke	Healthcare Cost and Utilization Project (HCUP), Agency for Healthcare Research and Quality (AHRQ). United States State Inpatient Databases 2008-2009
Stroke	Ministry of Health (New Zealand). New Zealand National Minimum Dataset 2000-2002
Stroke	Ministry of Health (New Zealand). New Zealand National Minimum Dataset 2003-2007
Stroke	Ministry of Health (New Zealand). New Zealand National Minimum Dataset 2008-2012
Stroke	Ministry of Health (New Zealand). New Zealand National Minimum Dataset 2013-2014
Stroke	Ministry of Health (New Zealand). New Zealand National Minimum Dataset 2015. Wellington, New Zealand: Ministry of Health (New Zealand)
Stroke	Ministry of Health (Romania). Romania Hospital Inpatient Discharges 2000
Stroke	Ministry of Health (Romania). Romania Hospital Inpatient Discharges 2001
Stroke	Ministry of Health (Romania). Romania Hospital Inpatient Discharges 2002
Stroke	Ministry of Health (Romania). Romania Hospital Inpatient Discharges 2003
Stroke	Ministry of Health (Romania). Romania Hospital Inpatient Discharges 2004
Stroke	Ministry of Health (Romania). Romania Hospital Inpatient Discharges 2005
Stroke	National School of Public Health (Greece), World Health Organization (WHO). Greece WHO Multi-country Survey Study on Health and Health System Responsiveness 2000-2001
Stroke	Healthcare Cost and Utilization Project (HCUP), Agency for Healthcare Research and Quality (AHRQ). United States Nationwide Inpatient Sample 2014. Rockville, United States: Healthcare Cost and Utilization Project (HCUP), Agency for Healthcare Research and Quality (AHRQ)
Stroke	Healthcare Cost and Utilization Project (HCUP), Agency for Healthcare Research and Quality (AHRQ). United States State Inpatient Databases 2011. Rockville, United States: Healthcare Cost and Utilization Project (HCUP), Agency for Healthcare Research and Quality (AHRQ)
Stroke	Healthcare Cost and Utilization Project (HCUP), Agency for Healthcare Research and Quality (AHRQ). United States State Inpatient Databases 2012. Rockville, United States: Healthcare Cost and Utilization Project (HCUP), Agency for Healthcare Research and Quality (AHRQ)
Stroke	Healthcare Cost and Utilization Project (HCUP), Agency for Healthcare Research and Quality (AHRQ). United States State Inpatient Databases 2013. Rockville, United States: Healthcare Cost and Utilization Project (HCUP), Agency for Healthcare Research and Quality (AHRQ)
Stroke	Healthcare Cost and Utilization Project (HCUP), Agency for Healthcare Research and Quality (AHRQ). United States State Inpatient Databases 2014. Rockville, United States: Healthcare Cost and Utilization Project (HCUP), Agency for Healthcare Research and Quality (AHRQ)
Stroke	Healthcare Cost and Utilization Project (HCUP), Agency for Healthcare Research and Quality (AHRQ). United States Nationwide Inpatient Sample 2015. Rockville, United States: Healthcare Cost and Utilization Project (HCUP), Agency for Healthcare Research and Quality (AHRQ)
Stroke	Ministry of Health (Romania). Romania Hospital Inpatient Discharges 1980
Stroke	Ministry of Health and Social Security (Spain), National Statistics Institute (Spain). Spain Statistics on Health Establishments Providing Inpatient Care 1972
Stroke	Ministry of Health and Social Security (Spain), National Statistics Institute (Spain). Spain Statistics on Health Establishments Providing Inpatient Care 1973
Stroke	Ministry of Health and Social Security (Spain), National Statistics Institute (Spain). Spain Statistics on Health Establishments Providing Inpatient Care 1974
Stroke	Ministry of Health and Social Security (Spain), National Statistics Institute (Spain). Spain Statistics on Health Establishments Providing Inpatient Care 1975

Stroke	Ministry of Health and Social Security (Spain), National Statistics Institute (Spain). Spain Statistics on Health Establishments Providing Inpatient Care 1976
Stroke	Ministry of Health and Social Security (Spain), National Statistics Institute (Spain). Spain Statistics on Health Establishments Providing Inpatient Care 1977
Stroke	Ministry of Health and Social Security (Spain), National Statistics Institute (Spain). Spain Statistics on Health Establishments Providing Inpatient Care 1978
Stroke	Ministry of Health and Social Security (Spain), National Statistics Institute (Spain). Spain Statistics on Health Establishments Providing Inpatient Care 1979
Stroke	Ministry of Health and Social Security (Spain), National Statistics Institute (Spain). Spain Statistics on Health Establishments Providing Inpatient Care 1980
Stroke	Ministry of Health and Social Security (Spain), National Statistics Institute (Spain). Spain Statistics on Health Establishments Providing Inpatient Care 1981
Stroke	Ministry of Health and Consumer Affairs (Spain), National Statistics Institute (Spain). Spain Statistics on Health Establishments Providing Inpatient Care 1982
Stroke	Ministry of Health and Consumer Affairs (Spain), National Statistics Institute (Spain). Spain Statistics on Health Establishments Providing Inpatient Care 1983
Stroke	Ministry of Health and Consumer Affairs (Spain), National Statistics Institute (Spain). Spain Statistics on Health Establishments Providing Inpatient Care 1984
Stroke	Ministry of Health and Consumer Affairs (Spain), National Statistics Institute (Spain). Spain Statistics on Health Establishments Providing Inpatient Care 1985
Stroke	Ministry of Health and Consumer Affairs (Spain), National Statistics Institute (Spain). Spain Statistics on Health Establishments Providing Inpatient Care 1986
Stroke	Ministry of Health and Consumer Affairs (Spain), National Statistics Institute (Spain). Spain Statistics on Health Establishments Providing Inpatient Care 1987
Stroke	Ministry of Health and Consumer Affairs (Spain), National Statistics Institute (Spain). Spain Statistics on Health Establishments Providing Inpatient Care 1988
Stroke	Ministry of Health and Consumer Affairs (Spain), National Statistics Institute (Spain). Spain Statistics on Health Establishments Providing Inpatient Care 1989
Stroke	Ministry of Health and Consumer Affairs (Spain), National Statistics Institute (Spain). Spain Statistics on Health Establishments Providing Inpatient Care 1990
Stroke	Ministry of Health and Consumer Affairs (Spain), National Statistics Institute (Spain). Spain Statistics on Health Establishments Providing Inpatient Care 1991
Stroke	Ministry of Health and Consumer Affairs (Spain), National Statistics Institute (Spain). Spain Statistics on Health Establishments Providing Inpatient Care 1992
Stroke	Ministry of Health and Consumer Affairs (Spain), National Statistics Institute (Spain). Spain Statistics on Health Establishments Providing Inpatient Care 1993
Stroke	Ministry of Health and Consumer Affairs (Spain), National Statistics Institute (Spain). Spain Statistics on Health Establishments Providing Inpatient Care 1994
Stroke	Ministry of Health and Consumer Affairs (Spain), National Statistics Institute (Spain). Spain Statistics on Health Establishments Providing Inpatient Care 1995
Stroke	Ministry of Health and Consumer Affairs (Spain), National Statistics Institute (Spain). Spain Statistics on Health Establishments Providing Inpatient Care 1996
Stroke	Ministry of Health and Consumer Affairs (Spain). Spain Statistics on Health Establishments Providing Inpatient Care 1997. Madrid, Spain: Ministry of Health, Social Services and Equality (Spain)
Stroke	Ministry of Health and Consumer Affairs (Spain). Spain Statistics on Health Establishments Providing Inpatient Care 1998. Madrid, Spain: Ministry of Health, Social Services and Equality (Spain)
Stroke	Ministry of Health and Consumer Affairs (Spain). Spain Statistics on Health Establishments Providing Inpatient Care 1999. Madrid, Spain: Ministry of Health, Social Services and Equality (Spain)
Stroke	Ministry of Health and Consumer Affairs (Spain). Spain Statistics on Health Establishments Providing Inpatient Care 2000. Madrid, Spain: Ministry of Health, Social Services and Equality (Spain)
Stroke	Ministry of Health and Consumer Affairs (Spain). Spain Statistics on Health Establishments Providing Inpatient Care 2001. Madrid, Spain: Ministry of Health, Social Services and Equality (Spain)
Stroke	Ministry of Health and Consumer Affairs (Spain). Spain Statistics on Health Establishments Providing Inpatient Care 2002. Madrid, Spain: Ministry of Health, Social Services and Equality (Spain)
Stroke	Ministry of Health and Consumer Affairs (Spain). Spain Statistics on Health Establishments Providing Inpatient Care 2003. Madrid, Spain: Ministry of Health, Social Services and Equality (Spain)
Stroke	Ministry of Health and Consumer Affairs (Spain). Spain Statistics on Health Establishments Providing Inpatient Care 2004. Madrid, Spain: Ministry of Health, Social Services and Equality (Spain)
Stroke	Ministry of Health and Consumer Affairs (Spain). Spain Statistics on Health Establishments Providing Inpatient Care 2005. Madrid, Spain: Ministry of Health, Social Services and Equality (Spain)
Stroke	Ministry of Health and Consumer Affairs (Spain). Spain Statistics on Health Establishments Providing Inpatient Care 2006. Madrid, Spain: Ministry of Health, Social Services and Equality (Spain)
Stroke	Ministry of Health and Consumer Affairs (Spain). Spain Statistics on Health Establishments Providing Inpatient Care 2007. Madrid, Spain: Ministry of Health, Social Services and Equality (Spain)
Stroke	Ministry of Health and Consumer Affairs (Spain). Spain Statistics on Health Establishments Providing Inpatient Care 2008. Madrid, Spain: Ministry of Health, Social Services and Equality (Spain)
Stroke	Ministry of Health and Consumer Affairs (Spain). Spain Statistics on Health Establishments Providing Inpatient Care 2009. Madrid, Spain: Ministry of Health, Social Services and Equality (Spain)
Stroke	Ministry of Health, Social Services and Equality (Spain). Spain Statistics on Specialized Healthcare Centers 2010. Madrid, Spain: Ministry of Health, Social Services and Equality (Spain)
Stroke	Ministry of Health, Social Services and Equality (Spain). Spain Statistics on Specialized Healthcare Centers 2011. Madrid, Spain: Ministry of Health, Social Services and Equality (Spain)
Stroke	Ministry of Health, Social Services and Equality (Spain). Spain Statistics on Specialized Healthcare Centers 2012. Madrid, Spain: Ministry of Health, Social Services and Equality (Spain)

Stroke	Ministry of Health, Social Services and Equality (Spain). Spain Statistics on Specialized Healthcare Centers 2013. Madrid, Spain: Ministry of Health, Social Services and Equality (Spain)
Stroke	Ministry of Health, Social Services and Equality (Spain). Spain Statistics on Specialized Healthcare Centers 2014. Madrid, Spain: Ministry of Health, Social Services and Equality (Spain)
Stroke	Ministry of Health (Romania). Romania Hospital Inpatient Discharges 1985
Stroke	Ministry of Health (Romania). Romania Hospital Inpatient Discharges 1986
Stroke	Ministry of Health (Romania). Romania Hospital Inpatient Discharges 1987
Stroke	Ministry of Health (Romania). Romania Hospital Inpatient Discharges 1988
Stroke	Ministry of Health (Romania). Romania Hospital Inpatient Discharges 1989
Stroke	Ministry of Health (Romania). Romania Hospital Inpatient Discharges 1990
Stroke	Ministry of Health (Romania). Romania Hospital Inpatient Discharges 1991
Stroke	Ministry of Health (Romania). Romania Hospital Inpatient Discharges 1992
Stroke	Ministry of Health (Romania). Romania Hospital Inpatient Discharges 1993
Stroke	Ministry of Health (Romania). Romania Hospital Inpatient Discharges 1994
Stroke	Ministry of Health (Romania). Romania Hospital Inpatient Discharges 1995
Stroke	Ministry of Health (Romania). Romania Hospital Inpatient Discharges 1996
Stroke	Ministry of Health (Romania). Romania Hospital Inpatient Discharges 1997
Stroke	Ministry of Health (Romania). Romania Hospital Inpatient Discharges 1998
Stroke	Ministry of Health (Romania). Romania Hospital Inpatient Discharges 1999
Stroke	Ministry of Health (Romania). Romania Hospital Inpatient Discharges 2013
Stroke	Bureau of Statistics (Guyana), World Bank. Guyana Living Standards Measurement Survey 1992-1993
Stroke	Ministry of Statistics and Programme Implementation (India). India National Sample Survey Round 52 1995-1996. New Delhi, India: Ministry of Statistics and Programme Implementation (India)
Stroke	Ministry of Statistics and Programme Implementation (India). India National Sample Survey Round 60 2004. New Delhi, India: Ministry of Statistics and Programme Implementation (India)
Stroke	National Institute of Statistics and Informatics (Peru), World Bank (WB). Peru Living Standards Measurement Survey 1990
Stroke	National Institute of Statistics and Informatics (INEI) (Peru), World Bank. Peru Living Standards Measurement Survey 1991. Washington DC, United States: World Bank
Stroke	National Institute of Statistics and Informatics (Peru), World Bank (WB). Peru Living Standards Measurement Survey 1994
Stroke	Ministry of Social Affairs (Serbia), World Bank. Yugoslavia, Federal Republic - Serbia Living Standards Measurement Survey 2002. Washington DC, United States: World Bank
Stroke	Ministry of Social Affairs (Serbia), World Bank. Serbia and Montenegro - Serbia Living Standards Measurement Survey 2003. Washington DC, United States: World Bank
Stroke	International Institute for Population Sciences (India), World Health Organization (WHO). India World Health Survey 2003. Geneva, Switzerland: World Health Organization (WHO), 2005
Stroke	World Health Organization (WHO). Malaysia World Health Survey 2003. Geneva, Switzerland: World Health Organization (WHO), 2005
Stroke	World Health Organization (WHO). Mauritius World Health Survey 2003. Geneva, Switzerland: World Health Organization (WHO), 2005
Stroke	World Health Organization (WHO). Sweden World Health Survey 2003. Geneva, Switzerland: World Health Organization (WHO), 2005
Stroke	World Health Organization (WHO). Ukraine World Health Survey 2002-2003. Geneva, Switzerland: World Health Organization (WHO), 2005
Stroke	National Institute of Statistics and Informatics (INEI) (Peru), United Nations Economic Commission for Latin America and the Caribbean (CEPAL), Institute of Research for Development (France). Peru National Household Survey 2007. Lima, Peru: National Institute of Statistics and Informatics (INEI) (Peru)
Stroke	National Institute of Statistics and Informatics (INEI) (Peru), United Nations Economic Commission for Latin America and the Caribbean (CEPAL), Institute of Research for Development (France). Peru National Household Survey 2008. Lima, Peru: National Institute of Statistics and Informatics (INEI) (Peru)
Stroke	National Institute of Statistics and Informatics (INEI) (Peru). Peru National Household Survey 2009. Lima, Peru: National Institute of Statistics and Informatics (INEI) (Peru)
Stroke	National Institute of Statistics and Informatics (INEI) (Peru). Peru National Household Survey 2010. Lima, Peru: National Institute of Statistics and Informatics (INEI) (Peru)
Stroke	National Institute of Statistics and Informatics (INEI) (Peru). Peru National Household Survey, Second Quarter 1998. Lima, Peru: National Institute of Statistics and Informatics (INEI) (Peru)
Stroke	National Institute of Statistics and Informatics (INEI) (Peru). Peru National Household Survey, Second Quarter 1999. Lima, Peru: National Institute of Statistics and Informatics (INEI) (Peru)
Stroke	National Institute of Statistics (Albania), World Bank (WB). Albania Living Standards Measurement Survey 2002. Washington DC, United States: World Bank (WB)
Stroke	National Institute of Statistics (Albania), World Bank (WB). Albania Living Standards Measurement Survey 2005. Washington DC, United States: World Bank (WB)
Stroke	National Institute of Statistics and Informatics (INEI) (Peru). Peru National Household Survey, Second Quarter 2000. Lima, Peru: National Institute of Statistics and Informatics (INEI) (Peru)
Stroke	Ministry of Social Affairs (Serbia), World Bank. Serbia Living Standards Measurement Survey 2007. Washington DC, United States: World Bank
Stroke	National Institute of Statistics and Informatics (INEI) (Peru). Peru National Household Survey 2005. Lima, Peru: National Institute of Statistics and Informatics (INEI) (Peru)
Stroke	National Institute of Statistics and Informatics (INEI) (Peru). Peru National Household Survey 2006. Lima, Peru: National Institute of Statistics and Informatics (INEI) (Peru)
Stroke	International Institute for Population Sciences (India), World Health Organization (WHO). India WHO Study on Global Ageing and Adult Health 2007. Geneva, Switzerland: World Health Organization (WHO), 2007
Stroke	Ministry of Health (Syria), World Health Organization (WHO). Syria WHO Multi-country Survey Study on Health and Health System Responsiveness 2000-2001
Stroke	National Institute of Statistics (Niger), World Bank. Niger National Survey on Household Living Conditions and Agriculture 2011-2012
Stroke	Sridharan SE, Unnikrishnan JP, Sukumaran S, Sylaja PN, Nayak SD, Sarma PS, Radhakrishnan K. Incidence, Types, Risk Factors, and Outcome of Stroke in a Developing Country. Stroke. 2009; 40(4): 1212-8
Stroke	Appelros P, Nydevik I, Seiger Åke, Terént A. High Incidence Rates of Stroke in Orebro, Sweden: Further Support for Regional Incidence Differences within Scandinavia. Cerebrovasc Dis. 2002; 14(3-4): 161-8
Stroke	Hallström B, Jönsson A-C, Nerbrand C, Norrving B, Lindgren A. Stroke Incidence and Survival in the Beginning of the 21st Century in Southern Sweden: Comparisons With the Late 20th Century and Projections Into the Future. Stroke. 2008; 39(1): 10-5

Stroke	Dalal PM, Malik S, Bhattacharjee M, Trivedi ND, Vairale J, Bhat P, Deshmukh S, Khandelwal K, Mathur VD. Population-based stroke survey in Mumbai, India: incidence and 28-day case fatality. Neuroepidemiology. 2008; 31(4): 254-61
Stroke	Mihálka L, Smolanka V, Bulecza B, Mulesa S, Bereczki D. A Population Study of Stroke in West Ukraine: Incidence, Stroke Services, and 30-Day Case Fatality. Stroke. 2001; 32(10): 2227-31
Stroke	Terént A. Trends in Stroke Incidence and 10-Year Survival in Söderhamn, Sweden, 1975-2001. Stroke. 2003; 34(6): 1353-8
Stroke	Statistics Sweden, Swedish National Institute of Public Health. Sweden National Survey of Public Health 2009
Stroke	Statistics Sweden, Swedish National Institute of Public Health. Sweden National Survey of Public Health 2007
Stroke	Institute for Public Health, Ministry of Health (Malaysia). Malaysia National Health And Morbidity Survey 2006. Kuala Lumpur, Malaysia: Institute for Public Health, Ministry of Health (Malaysia)
Stroke	Northern Ireland Statistics and Research Agency. Central Survey Unit, Northern Ireland Health and Social Wellbeing Survey, 2001 [computer file]. Colchester, Essex: UK Data Archive [distributor], October 2002. SN: 4590 , http://dx.doi.org/10.5255/UKDA-SN-4590-1
Stroke	Northern Ireland Statistics and Research Agency. Central Survey Unit, Northern Ireland Health and Social Wellbeing Survey, 2005-2006 [computer file]. Colchester, Essex: UK Data Archive [distributor], October 2007. SN: 5710, http://dx.doi.org/10.5255/UKDA-SN-5710-1
Stroke	Northern Ireland Statistics and Research Agency. Central Survey Unit, Northern Ireland Health and Social Wellbeing Survey, 1997 [computer file]. Colchester, Essex: UK Data Archive [distributor], October 2002. SN: 4589, http://dx.doi.org/10.5255/UKDA-SN-4589-1
Stroke	Northern Ireland Statistics and Research Agency (NISRA). United Kingdom - Northern Ireland Health Survey 2010-2011 - UK Data Service
Stroke	Das SK, Banerjee TK, Biswas A, Roy T, Raut DK, Mukherjee CS, Chaudhuri A, Hazra A, Roy J. A Prospective Community-Based Study of Stroke in Kolkata, India. Stroke. 2007; 38(3): 906-10
Stroke	Banerjee TK, Mukherjee CS, Sarkhel A. Stroke in the urban population of Calcutta – an epidemiological study. Neuroepidemiology. 2001; 20(3): 201-7
Stroke	Das S, Sanyal K, Moitra A. A pilot study on neuroepidemiology in urban Bengal. Indian J Public Health. 1998; 42(2): 34-6
Stroke	Dhamija RK, Dhamija SB. Prevalence of stroke in rural community – an overview of Indian experience. J Assoc Physicians India. 1998; 46(4): 351-4
Stroke	Koul R, Motta A, Razdan S. Epidemiology of young strokes in rural Kashmir, India. Acta Neurol Scand. 1990; 82(1): 1-3
Stroke	Saha SP, Bhattacharya S, Das SK, Maity B, Roy T, Raut DK. Epidemiological study of neurological disorders in a rural population of Eastern India. J Indian Med Assoc. 2003; 101(5): 299-304
Stroke	National Board of Health and Welfare (Sweden). Sweden National Patient Register 1998. Stockholm, Sweden: National Board of Health and Welfare (Sweden)
Stroke	National Board of Health and Welfare (Sweden). Sweden National Patient Register 1999. Stockholm, Sweden: National Board of Health and Welfare (Sweden)
Stroke	National Board of Health and Welfare (Sweden). Sweden National Patient Register 2011. Stockholm, Sweden: National Board of Health and Welfare (Sweden)
Stroke	National Board of Health and Welfare (Sweden). Sweden National Patient Register 2012. Stockholm, Sweden: National Board of Health and Welfare (Sweden)
Stroke	Statistics Sweden, Swedish National Institute of Public Health. Sweden National Survey of Public Health 2008
Stroke	Hilmarsson A, Kjartansson O, Olafsson E. Incidence of First Stroke A Population Study in Iceland. Stroke. 2013; 44(6): 1714-6
Stroke	Pilot Study, 2005 (Data Set 27-28, Cunningham, Shayna.) [machine-readable data file and documentation]. Geneva, Switzerland: World Health Organization (Producer). Los Altos, CA: Sociometrics Corporation, Data Archive of Social Research on Aging (Producer & Distributor)
Stroke	Statistics Sweden, Swedish National Institute of Public Health. Sweden National Survey of Public Health 2012
Stroke	Statistics Sweden, Swedish National Institute of Public Health. Sweden National Survey of Public Health 2010
Stroke	Statistics Sweden, Swedish National Institute of Public Health. Sweden National Survey of Public Health 2004
Stroke	Statistics Sweden, Swedish National Institute of Public Health. Sweden National Survey of Public Health 2005
Stroke	Statistics Sweden, Swedish National Institute of Public Health. Sweden National Survey of Public Health 2011
Stroke	Lindmark A, Glader E-L, Asplund K, Norrving B, Eriksson M, Riks-Stroke Collaboration. Socioeconomic disparities in stroke case fatality--Observations from Riks-Stroke, the Swedish stroke register. Int J Stroke. 2014; 9(4): 429–36
Stroke	Trinity College Dublin. Ireland Longitudinal Study on Ageing 2009-2011. Dublin, Ireland: Irish Social Science Data Archive, University College Dublin
Stroke	Statistics Sweden, Swedish National Institute of Public Health. Sweden National Survey of Public Health 2013
Stroke	Public Health Agency of Sweden, Statistics Sweden. Sweden National Survey of Public Health 2014
Stroke	International Research Associates (INRA) Europe, World Health Organization (WHO). Iceland WHO Multi-country Survey Study on Health and Health System Responsiveness 2000-2001. Geneva, Switzerland: World Health Organization (WHO)
Stroke	International Research Associates (INRA) Europe, World Health Organization (WHO). Sweden WHO Multi-country Survey Study on Health and Health System Responsiveness 2000-2001. Geneva, Switzerland: World Health Organization (WHO)
Stroke	Kiev International Institute of Sociology, World Health Organization (WHO). Ukraine WHO Multi-country Survey Study on Health and Health System Responsiveness 2000-2001. Geneva, Switzerland: World Health Organization (WHO)
Stroke	Gallup Europe, World Health Organization (WHO). Venezuela WHO Multi-country Survey Study on Health and Health System Responsiveness 2000-2001. Geneva, Switzerland: World Health Organization (WHO)
Stroke	Börsch-Supan, A. (2013). Survey of Health, Ageing and Retirement in Europe (SHARE) Wave 2. Release version: 2.6.0. SHARE-ERIC. Data set. DOI: 10.6103/SHARE.w2.260
Stroke	Börsch-Supan, A. (2013). Survey of Health, Ageing and Retirement in Europe (SHARE) Wave 4. Release version: 1.1.1. SHARE-ERIC. Data set. DOI: 10.6103/SHARE.w4.111
Stroke	Börsch-Supan, A. (2015). Survey of Health, Ageing and Retirement in Europe (SHARE) Wave 5. Release version: 1.0.0. SHARE-ERIC. Data set. DOI: 10.6103/SHARE.w5.100
Stroke	Government of India, Ministry of Statistics and Programme Implementation (India). India National Sample Survey Round 71 2014. New Delhi, India: Ministry of Statistics and Programme Implementation (India)
Stroke	Armenian Sociological Association, Concluzia-Prim Center for Survey Methodology (Moldova), Institute for Advanced Studies (Austria), London School of Hygiene and Tropical Medicine, University of Aberdeen. Armenia Health in Times of Transition Household Survey 2010
Stroke	Zhi X, Joas E, Waern M, Östling S, Börjesson-Hanson A, Skoog I. Prevalence of cardiovascular disorders and risk factors in two 75-year-old birth cohorts examined in 1976-1977 and 2005-2006. Aging Clin Exp Res. 2013; 25(4): 377-83
Stroke	Concluzia-Prim Center for Survey Methodology (Moldova), East-Ukranian Foundation For Social Research, Institute for Advanced Studies (Austria), London School of Hygiene and Tropical Medicine, University of Aberdeen. Ukraine Health in Times of Transition Household Survey 2010
Stroke	National Board of Health and Welfare (Sweden). Sweden National Patient Register 1998-2002
Stroke	National Board of Health and Welfare (Sweden). Sweden National Patient Register 2003-2007
Stroke	National Board of Health and Welfare (Sweden). Sweden National Patient Register 2008-2012
Stroke	All India Institute of Medical Sciences, New Delhi (AIIMS), Bangur Institute of Neurology (India), Indian Statistical Institute, National Neurosciences Center, Calcutta (India). India - Kolkata Study for Epidemiology of Neurological Disorders 2003-2004
Stroke	Institute for Public Health, Ministry of Health (Malaysia). Malaysia National Health and Morbidity Survey 2011
Stroke	St. John's National Academy of Health Sciences. INdian Stroke ProspectIve REgistry (INSPIRE) Data 2009-2014

Stroke	Kalkonde YV, Sahane V, Deshmukh MD, Nila S, Mandava P, Bang A. High Prevalence of Stroke in Rural Gadchiroli, India: A Community-Based Study. Neuroepidemiology. 2016; 46(4): 235-9
Stroke	Moodbidri P, Mehmi G, Sharma A, Arora OP, Dhanuka AK, Sobti MK, Sehgal H, Kaur M, Grewal SS, Jhawar SS, Shadangi TN, Arora T, Saxena A, Sachdeva G, Gill JS, Brar RS, Gill A, Bakshi SS, Pawar SS, Singh G, Sikka P, Litoria PK, Sharma M. Incidence, short-term outcome, and spatial distribution of stroke patients in Ludhiana, India.
Stroke	Zhu L, Fratiglioni L, Guo Z, Aguero-Torres H, Winblad B, Viitanen M. Association of stroke with dementia, cognitive impairment, and functional disability in the very old: a population-based study. Stroke. 1998; 29(10): 2094–9
Stroke	Hornsten C, Molander L, Gustafson Y. The prevalence of stroke and the association between stroke and depression among a very old population. Arch Gerontol Geriatr. 2012; 55(3): 555–9
Stroke	Institute of Public Health of Serbia. Serbia National Hospital Discharge Database 2012
Stroke	Department of Health, Social Services and Public Safety (Northern Ireland), Information Centre for Health and Social Care, NHS, NHS England, NHS Health Scotland, NHS Wales. United Kingdom Hospital Patient and Discharge Data 2006
Stroke	Directorate of Health (Iceland). Iceland Hospital Data Registry 2008
Stroke	Ministry of Health (Albania). Albania Inpatient Care Discharges per 100 1993
Stroke	Ministry of Health (Albania). Albania Inpatient Care Discharges per 100 1994
Stroke	Ministry of Health (Albania). Albania Inpatient Care Discharges per 100 1995
Stroke	Ministry of Health (Albania). Albania Inpatient Care Discharges per 100 1996
Stroke	Ministry of Health (Albania). Albania Inpatient Care Discharges per 100 1997
Stroke	Ministry of Health (Albania). Albania Inpatient Care Discharges per 100 1998
Stroke	Ministry of Health (Albania). Albania Inpatient Care Discharges per 100 1999
Stroke	Ministry of Health (Albania). Albania Inpatient Care Discharges per 100 2000
Stroke	Ministry of Health (Albania). Albania Inpatient Care Discharges per 100 2001
Stroke	Ministry of Health (Albania). Albania Inpatient Care Discharges per 100 2002
Stroke	Ministry of Health (Albania). Albania Inpatient Care Discharges per 100 2003
Stroke	Ministry of Health (Albania). Albania Inpatient Care Discharges per 100 2004
Stroke	Ministry of Health (Albania). Albania Inpatient Care Discharges per 100 2005
Stroke	Ministry of Health (Albania). Albania Inpatient Care Discharges per 100 2006
Stroke	Ministry of Health (Albania). Albania Inpatient Care Discharges per 100 2007
Stroke	Ministry of Health (Albania). Albania Inpatient Care Discharges per 100 2008
Stroke	Ministry of Health (Albania). Albania Inpatient Care Discharges per 100 2009
Stroke	Ministry of Health (Albania). Albania Inpatient Care Discharges per 100 2010
Stroke	Ministry of Health (Albania). Albania Inpatient Care Discharges per 100 2011
Stroke	Ministry of Health (Albania). Albania Inpatient Care Discharges per 100 2012
Stroke	Ministry of Health (Albania). Albania Inpatient Care Discharges per 100 2013
Stroke	Directorate of Health (Iceland). Iceland Hospital Data Registry 2010
Stroke	Directorate of Health (Iceland). Iceland Hospital Data Registry 2011
Stroke	Directorate of Health (Iceland). Iceland Hospital Data Registry 2012
Stroke	Directorate of Health (Iceland). Iceland Hospital Data Registry 2013
Stroke	Directorate of Health (Iceland). Iceland Hospital Data Registry 2014
Stroke	Directorate of Health (Iceland). Iceland Hospital Data Registry 1999
Stroke	Directorate of Health (Iceland). Iceland Hospital Data Registry 1995
Stroke	Directorate of Health (Iceland). Iceland Hospital Data Registry 1994
Stroke	Directorate of Health (Iceland). Iceland Hospital Data Registry 1993
Stroke	Directorate of Health (Iceland). Iceland Hospital Data Registry 1992
Stroke	Directorate of Health (Iceland). Iceland Hospital Data Registry 1991
Stroke	Directorate of Health (Iceland). Iceland Hospital Data Registry 1990
Stroke	Directorate of Health (Iceland). Iceland Hospital Data Registry 1989
Stroke	Directorate of Health (Iceland). Iceland Hospital Data Registry 1988
Stroke	Ministry of Health (Armenia). Armenia Inpatient Care Discharges per 100 1980
Stroke	Ministry of Health (Armenia). Armenia Inpatient Care Discharges per 100 1981
Stroke	Ministry of Health (Armenia). Armenia Inpatient Care Discharges per 100 1982
Stroke	Ministry of Health (Armenia). Armenia Inpatient Care Discharges per 100 1983
Stroke	Ministry of Health (Armenia). Armenia Inpatient Care Discharges per 100 1984
Stroke	Ministry of Health (Armenia). Armenia Inpatient Care Discharges per 100 1985
Stroke	Ministry of Health (Armenia). Armenia Inpatient Care Discharges per 100 1986
Stroke	Ministry of Health (Armenia). Armenia Inpatient Care Discharges per 100 1987
Stroke	Ministry of Health (Armenia). Armenia Inpatient Care Discharges per 100 1988
Stroke	Ministry of Health (Armenia). Armenia Inpatient Care Discharges per 100 1989
Stroke	Ministry of Health (Armenia). Armenia Inpatient Care Discharges per 100 1990
Stroke	Ministry of Health (Armenia). Armenia Inpatient Care Discharges per 100 1991
Stroke	Ministry of Health (Armenia). Armenia Inpatient Care Discharges per 100 1992
Stroke	Ministry of Health (Armenia). Armenia Inpatient Care Discharges per 100 1993
Stroke	Ministry of Health (Armenia). Armenia Inpatient Care Discharges per 100 1994
Stroke	Ministry of Health (Armenia). Armenia Inpatient Care Discharges per 100 1995
Stroke	Ministry of Health (Armenia). Armenia Inpatient Care Discharges per 100 1996
Stroke	Ministry of Health (Armenia). Armenia Inpatient Care Discharges per 100 1997
Stroke	Ministry of Health (Armenia). Armenia Inpatient Care Discharges per 100 1998
Stroke	Ministry of Health (Armenia). Armenia Inpatient Care Discharges per 100 1999
Stroke	Ministry of Health (Armenia). Armenia Inpatient Care Discharges per 100 2000
Stroke	Ministry of Health (Armenia). Armenia Inpatient Care Discharges per 100 2001
Stroke	Ministry of Health (Armenia). Armenia Inpatient Care Discharges per 100 2002
Stroke	Ministry of Health (Armenia). Armenia Inpatient Care Discharges per 100 2003
Stroke	Ministry of Health (Armenia). Armenia Inpatient Care Discharges per 100 2004
Stroke	Ministry of Health (Armenia). Armenia Inpatient Care Discharges per 100 2005
Stroke	Ministry of Health (Armenia). Armenia Inpatient Care Discharges per 100 2006

Stroke	Ministry of Health (Armenia). Armenia Inpatient Care Discharges per 100 2007
Stroke	Ministry of Health (Armenia). Armenia Inpatient Care Discharges per 100 2008
Stroke	Ministry of Health (Armenia). Armenia Inpatient Care Discharges per 100 2009
Stroke	Ministry of Health (Armenia). Armenia Inpatient Care Discharges per 100 2010
Stroke	Ministry of Health (Armenia). Armenia Inpatient Care Discharges per 100 2011
Stroke	Ministry of Health (Armenia). Armenia Inpatient Care Discharges per 100 2012
Stroke	Ministry of Health (Armenia). Armenia Inpatient Care Discharges per 100 2013
Stroke	Ministry of Health (Armenia). Armenia Inpatient Care Discharges per 100 2014
Stroke	Center for Health Statistics, Ministry of Health (Ukraine). Ukraine Inpatient Care Discharges per 100 1980
Stroke	Center for Health Statistics, Ministry of Health (Ukraine). Ukraine Inpatient Care Discharges per 100 1981
Stroke	Center for Health Statistics, Ministry of Health (Ukraine). Ukraine Inpatient Care Discharges per 100 1982
Stroke	Center for Health Statistics, Ministry of Health (Ukraine). Ukraine Inpatient Care Discharges per 100 1983
Stroke	Center for Health Statistics, Ministry of Health (Ukraine). Ukraine Inpatient Care Discharges per 100 1984
Stroke	Center for Health Statistics, Ministry of Health (Ukraine). Ukraine Inpatient Care Discharges per 100 1985
Stroke	Center for Health Statistics, Ministry of Health (Ukraine). Ukraine Inpatient Care Discharges per 100 1986
Stroke	Center for Health Statistics, Ministry of Health (Ukraine). Ukraine Inpatient Care Discharges per 100 1987
Stroke	Center for Health Statistics, Ministry of Health (Ukraine). Ukraine Inpatient Care Discharges per 100 1988
Stroke	Center for Health Statistics, Ministry of Health (Ukraine). Ukraine Inpatient Care Discharges per 100 1989
Stroke	Center for Health Statistics, Ministry of Health (Ukraine). Ukraine Inpatient Care Discharges per 100 1990
Stroke	Center for Health Statistics, Ministry of Health (Ukraine). Ukraine Inpatient Care Discharges per 100 1991
Stroke	Center for Health Statistics, Ministry of Health (Ukraine). Ukraine Inpatient Care Discharges per 100 1992
Stroke	Center for Health Statistics, Ministry of Health (Ukraine). Ukraine Inpatient Care Discharges per 100 1993
Stroke	Center for Health Statistics, Ministry of Health (Ukraine). Ukraine Inpatient Care Discharges per 100 1994
Stroke	Center for Health Statistics, Ministry of Health (Ukraine). Ukraine Inpatient Care Discharges per 100 1995
Stroke	Center for Health Statistics, Ministry of Health (Ukraine). Ukraine Inpatient Care Discharges per 100 1996
Stroke	Center for Health Statistics, Ministry of Health (Ukraine). Ukraine Inpatient Care Discharges per 100 1997
Stroke	Center for Health Statistics, Ministry of Health (Ukraine). Ukraine Inpatient Care Discharges per 100 1998
Stroke	Center for Health Statistics, Ministry of Health (Ukraine). Ukraine Inpatient Care Discharges per 100 1999
Stroke	Center for Health Statistics, Ministry of Health (Ukraine). Ukraine Inpatient Care Discharges per 100 2000
Stroke	Center for Health Statistics, Ministry of Health (Ukraine). Ukraine Inpatient Care Discharges per 100 2001
Stroke	Center for Health Statistics, Ministry of Health (Ukraine). Ukraine Inpatient Care Discharges per 100 2002
Stroke	Center for Health Statistics, Ministry of Health (Ukraine). Ukraine Inpatient Care Discharges per 100 2003
Stroke	Center for Health Statistics, Ministry of Health (Ukraine). Ukraine Inpatient Care Discharges per 100 2004
Stroke	Center for Health Statistics, Ministry of Health (Ukraine). Ukraine Inpatient Care Discharges per 100 2005
Stroke	Center for Health Statistics, Ministry of Health (Ukraine). Ukraine Inpatient Care Discharges per 100 2006
Stroke	Center for Health Statistics, Ministry of Health (Ukraine). Ukraine Inpatient Care Discharges per 100 2007
Stroke	Center for Health Statistics, Ministry of Health (Ukraine). Ukraine Inpatient Care Discharges per 100 2008
Stroke	Center for Health Statistics, Ministry of Health (Ukraine). Ukraine Inpatient Care Discharges per 100 2009
Stroke	Center for Health Statistics, Ministry of Health (Ukraine). Ukraine Inpatient Care Discharges per 100 2010
Stroke	Center for Health Statistics, Ministry of Health (Ukraine). Ukraine Inpatient Care Discharges per 100 2011
Stroke	Center for Health Statistics, Ministry of Health (Ukraine). Ukraine Inpatient Care Discharges per 100 2012
Stroke	Center for Health Statistics, Ministry of Health (Ukraine). Ukraine Inpatient Care Discharges per 100 2013
Stroke	Institute of Public Health of Serbia. Serbia Inpatient Care Discharges Per 100 2010
Stroke	Institute of Public Health of Serbia. Serbia Inpatient Care Discharges Per 100 2011
Stroke	Institute of Public Health of Serbia. Serbia Inpatient Care Discharges Per 100 2013
Stroke	Institute of Public Health of Serbia. Serbia Inpatient Care Discharges Per 100 2014
Stroke	Department of Health, Social Services and Public Safety (Northern Ireland), Information Centre for Health and Social Care, NHS, NHS England, NHS Health Scotland, NHS Wales. United Kingdom Hospital Patient and Discharge Data 2012
Stroke	Department of Health, Social Services and Public Safety (Northern Ireland), Information Centre for Health and Social Care, NHS, NHS England, NHS Health Scotland, NHS Wales. United Kingdom Hospital Patient and Discharge Data 2013
Stroke	Department of Health, Social Services and Public Safety (Northern Ireland), Information Centre for Health and Social Care, NHS, NHS England, NHS Health Scotland, NHS Wales. United Kingdom Hospital Patient and Discharge Data 2014
Stroke	National Board of Health and Welfare (Sweden). Sweden National Patient Register 1997
Stroke	National Board of Health and Welfare (Sweden). Sweden National Patient Register 1996. Stockholm, Sweden: National Board of Health and Welfare (Sweden)
Stroke	National Board of Health and Welfare (Sweden). Sweden National Patient Register 1995. Stockholm, Sweden: National Board of Health and Welfare (Sweden)
Stroke	National Board of Health and Welfare (Sweden). Sweden National Patient Register 1994. Stockholm, Sweden: National Board of Health and Welfare (Sweden)
Stroke	National Board of Health and Welfare (Sweden). Sweden National Patient Register 1993. Stockholm, Sweden: National Board of Health and Welfare (Sweden)
Stroke	National Board of Health and Welfare (Sweden). Sweden National Patient Register 1992. Stockholm, Sweden: National Board of Health and Welfare (Sweden)
Stroke	National Board of Health and Welfare (Sweden). Sweden National Patient Register 1991. Stockholm, Sweden: National Board of Health and Welfare (Sweden)
Stroke	National Board of Health and Welfare (Sweden). Sweden National Patient Register 1990. Stockholm, Sweden: National Board of Health and Welfare (Sweden)
Stroke	National Board of Health and Welfare (Sweden). Sweden National Patient Register 1989. Stockholm, Sweden: National Board of Health and Welfare (Sweden)
Stroke	National Board of Health and Welfare (Sweden). Sweden National Patient Register 1988. Stockholm, Sweden: National Board of Health and Welfare (Sweden)
Stroke	National Board of Health and Welfare (Sweden). Sweden National Patient Register 1987. Stockholm, Sweden: National Board of Health and Welfare (Sweden)
Stroke	National Board of Health and Welfare (Sweden). Sweden National Patient Register 2013
Stroke	National Board of Health and Welfare (Sweden). Sweden National Patient Register 2014

Stroke	Joint Health Surveys Unit of Social and Community Planning Research and University College London, Scottish Health Survey, 1998 [computer file]. Colchester, Essex: UK Data Archive [distributor], July 2001. SN: 4379 , http://dx.doi.org/10.5255/UKDA-SN-4379-1
Stroke	Joint Health Surveys Unit, University College London and Medical Research Council. Social and Public Health Sciences Unit, Scottish Health Survey, 2003 [computer file]. Colchester, Essex: UK Data Archive [distributor], February 2006. SN: 5318
Stroke	World Health Organization (WHO). Bosnia and Herzegovina World Health Survey 2003. Geneva, Switzerland: World Health Organization (WHO), 2005
Stroke	World Health Organization (WHO). Chad World Health Survey 2003. Geneva, Switzerland: World Health Organization (WHO), 2005
Stroke	World Health Organization (WHO). Ireland World Health Survey 2003. Geneva, Switzerland: World Health Organization (WHO), 2005
Stroke	World Health Organization (WHO). Nepal World Health Survey 2003. Geneva, Switzerland: World Health Organization (WHO), 2005
Stroke	World Health Organization (WHO). Slovakia World Health Survey 2003. Geneva, Switzerland: World Health Organization (WHO), 2005
Stroke	World Health Organization (WHO). Tunisia World Health Survey 2003. Geneva, Switzerland: World Health Organization (WHO), 2005
Stroke	Centers for Disease Control and Prevention (CDC), Ministry of Health (Jordan), World Health Organization (WHO). Jordan STEPS Noncommunicable Disease Risk Factors Survey 2007
Stroke	Srpska), Federal Office of Statistics (Bosnia and Herzegovina), Swedish International Development Agency (SIDA), UK Department for International Development (DFID), United Nations Development Programme (UNDP), European Commission (EC), Government of Japan, World Bank (WB). Bosnia and Herzegovina Living Standards
Stroke	Srpska), Federal Office of Statistics (Bosnia and Herzegovina), Independent Bureau for Humanitarian Issues (IBHI), Birks Sinclair and Associates, LTD, Institute for Social and Economic Research, University of Essex. Bosnia and Herzegovina Living Standards Measurement Survey 2002. Washington, DC, United States: World Bank (WB)
Stroke	Federal Office of Statistics (Federation of Bosnia and Herzegovina), Independent Bureau for Humanitarian Issues (IBHI), Institute for Social and Economic Research, University of Essex, Institute of Statistics (Republic of Srpska). Bosnia and Herzegovina Living Standards Measurement Survey 2004-2005
Stroke	Public Health Authority of the Slovak Republic, World Health Organization (WHO). Slovakia WHO Multi-country Survey Study on Health and Health System Responsiveness 2000
Stroke	University of Ibadan (Nigeria), World Health Organization (WHO). Nigeria WHO Multi-country Survey Study on Health and Health System Responsiveness 2000-2001
Stroke	Scottish Centre for Social Research and University College London. Department of Epidemiology and Public Health, Scottish Health Survey, 2008 [computer file]. 2nd Edition. Colchester, Essex: UK Data Archive [distributor], April 2013. SN: 6383, http://dx.doi.org/10.5255/UKDA-SN-6383-2
Stroke	Scottish Centre for Social Research and University College London. Department of Epidemiology and Public Health, Scottish Health Survey, 2009 [computer file]. 4th Edition. Colchester, Essex: UK Data Archive [distributor], November 2011. SN: 6713, http://dx.doi.org/10.5255/UKDA-SN-6713-2
Stroke	ScotCen Social Research and University College London. Department of Epidemiology and Public Health, Scottish Health Survey, 2010 [computer file]. Colchester, Essex: UK Data Archive [distributor], April 2012. SN: 6987, http://dx.doi.org/10.5255/UKDA-SN-6987-1
Stroke	and Public Health and University of Glasgow. MRC/CSO Social and Public Health Sciences Unit, Scottish Health Survey, 2011 [computer file]. 2nd Edition. Colchester, Essex: UK Data Archive [distributor], August 2013. SN: 7247, http://dx.doi.org/10.5255/UKDA-SN-7247-2
Stroke	Longe AC, Osuntokun BO. Prevalence of neurological disorders in Udo, a rural community in southern Nigeria. Trop Geogr Med. 1989; 41(1): 36-40
Stroke	Mrabet A, Attia-Romdhane N, Ben Hamida M, Gharbi N, Le Noan H, Hentati R, Ben Mansour J, Srairi I. Epidemiologic aspects of cerebrovascular accidents in Tunisia. Rev Neurol (Paris). 1990; 146(4): 297-301
Stroke	Danesi M, Okubadejo N, Ojini F. Prevalence of stroke in an urban, mixed-income community in Lagos, Nigeria. Neuroepidemiology. 2007; 28(4): 216-23
Stroke	Flynn RWV, MacDonald TM, Murray GD, Ferguson C, Shah K, Doney ASF. The Tayside Stroke Cohort: exploiting advanced regional medical informatics to create a region-wide database for studying the pharmacoepidemiology of stroke. Pharmacoepidemiol Drug Saf. 2010; 19(7): 737-44
Stroke	Maheswaran R, Pearson T, Smeeton NC, Beevers SD, Campbell MJ, Wolfe CD. Outdoor air pollution and incidence of ischemic and hemorrhagic stroke: a small-area level ecological study. Stroke. 2012; 43(1): 22-7
Stroke	Damasceno A, Gomes J, Azevedo A, Carrilho C, Lobo V, Lopes H, Madede T, Pravinrai P, Silva-Matos C, Jalla S, Stewart S, Lunet N. An Epidemiological Study of Stroke Hospitalizations in Maputo, Mozambique A High Burden of Disease in a Resource-Poor Country. Stroke. 2010; 41(11): 2463-9
Stroke	National Bureau of Statistics (Nigeria). Nigeria Living Standards Survey 2008-2010. Abuja, Nigeria: National Bureau of Statistics (Nigeria)
Stroke	Trinity College Dublin. Ireland Longitudinal Study on Ageing 2012-2013. Dublin, Ireland: Irish Social Science Data Archive, University College Dublin
Stroke	ScotCen Social Research, Scottish Health Survey, 2013 [computer file]. Colchester, Essex: UK Data Archive [distributor], December 2014. SN: 7594, http://dx.doi.org/10.5255/UKDA-SN-7594-1
Stroke	Department of Epidemiology and Public Health, University College London, Scottish Centre for Social Research (ScotCen), University of Glasgow. United Kingdom - Scottish Health Survey 2012 - Scottish Government
Stroke	LINK Institute for Market and Social Research (Switzerland), World Health Organization (WHO). Switzerland WHO Multi-country Survey Study on Health and Health System Responsiveness 2000-2001. Geneva, Switzerland: World Health Organization (WHO)
Stroke	International Research Associates (INRA) Europe, World Health Organization (WHO). Ireland WHO Multi-country Survey Study on Health and Health System Responsiveness 2000-2001. Geneva, Switzerland: World Health Organization (WHO)
Stroke	Gallup Europe, World Health Organization (WHO). Jordan WHO Multi-country Survey Study on Health and Health System Responsiveness 2000-2001. Geneva, Switzerland: World Health Organization (WHO)
Stroke	Börsch-Supan, A. (2015). Survey of Health, Ageing and Retirement in Europe (SHARE) Wave 5. Release version: 1.0.0. SHARE-ERIC. Data set. DOI: 10.6103/SHARE.w5.100
Stroke	Concluzia-Prim Center for Survey Methodology (Moldova), Institute for Advanced Studies (Austria), London School of Hygiene and Tropical Medicine, SIAR Research and Consulting (Azerbaijan), University of Aberdeen. Azerbaijan Health in Times of Transition Household Survey 2010
Stroke	ScotCen Social Research. (2015). Scottish Health Survey, 2014. [data collection]. UK Data Service. SN: 7851, http://dx.doi.org/10.5255/UKDA-SN-7851-1
Stroke	Department of Health and Children (Ireland), Economic and Social Research Institute (ESRI) (Ireland). Ireland Hospital Inpatient Enquiry 2000
Stroke	Department of Health (Ireland), Economic and Social Research Institute (ESRI) (Ireland). Ireland Hospital Inpatient Enquiry 1980
Stroke	Department of Health (Ireland), Economic and Social Research Institute (ESRI) (Ireland). Ireland Hospital Inpatient Enquiry 1981
Stroke	Department of Health (Ireland), Economic and Social Research Institute (ESRI) (Ireland). Ireland Hospital Inpatient Enquiry 1982
Stroke	Department of Health (Ireland), Economic and Social Research Institute (ESRI) (Ireland). Ireland Hospital Inpatient Enquiry 1983
Stroke	Department of Health (Ireland), Economic and Social Research Institute (ESRI) (Ireland). Ireland Hospital Inpatient Enquiry 1984
Stroke	Department of Health (Ireland), Economic and Social Research Institute (ESRI) (Ireland). Ireland Hospital Inpatient Enquiry 1985
Stroke	Department of Health (Ireland), Economic and Social Research Institute (ESRI) (Ireland). Ireland Hospital Inpatient Enquiry 1986
Stroke	Department of Health (Ireland), Economic and Social Research Institute (ESRI) (Ireland). Ireland Hospital Inpatient Enquiry 1987
Stroke	Department of Health (Ireland), Economic and Social Research Institute (ESRI) (Ireland). Ireland Hospital Inpatient Enquiry 1988
Stroke	Department of Health (Ireland), Economic and Social Research Institute (ESRI) (Ireland). Ireland Hospital Inpatient Enquiry 1989
Stroke	Department of Health (Ireland), Economic and Social Research Institute (ESRI) (Ireland). Ireland Hospital Inpatient Enquiry 1990
Stroke	Department of Health (Ireland), Economic and Social Research Institute (ESRI) (Ireland). Ireland Hospital Inpatient Enquiry 1991
Stroke	Department of Health (Ireland), Economic and Social Research Institute (ESRI) (Ireland). Ireland Hospital Inpatient Enquiry 1992

Stroke	Department of Health (Ireland), Economic and Social Research Institute (ESRI) (Ireland). Ireland Hospital Inpatient Enquiry 1993
Stroke	Department of Health (Ireland), Economic and Social Research Institute (ESRI) (Ireland). Ireland Hospital Inpatient Enquiry 1994
Stroke	Department of Health (Ireland), Economic and Social Research Institute (ESRI) (Ireland). Ireland Hospital Inpatient Enquiry 1995
Stroke	Department of Health (Ireland), Economic and Social Research Institute (ESRI) (Ireland). Ireland Hospital Inpatient Enquiry 1996
Stroke	Department of Health and Children (Ireland), Economic and Social Research Institute (ESRI) (Ireland). Ireland Hospital Inpatient Enquiry 1997
Stroke	Department of Health and Children (Ireland), Economic and Social Research Institute (ESRI) (Ireland). Ireland Hospital Inpatient Enquiry 1998
Stroke	Department of Health and Children (Ireland), Economic and Social Research Institute (ESRI) (Ireland). Ireland Hospital Inpatient Enquiry 1999
Stroke	Economic and Social Research Institute (ESRI) (Ireland), Health Service Executive (HSE) (Ireland). Ireland Hospital Inpatient Enquiry 2013
Stroke	Economic and Social Research Institute (ESRI) (Ireland), Health Service Executive (HSE) (Ireland). Ireland Hospital Inpatient Enquiry 2014
Stroke	Federal Statistical Office (Switzerland). Switzerland Medical Statistics of Hospitals 1997
Stroke	Federal Statistical Office (Switzerland). Switzerland Medical Statistics of Hospitals 1998
Stroke	Federal Statistical Office (Switzerland). Switzerland Medical Statistics of Hospitals 2013
Stroke	Federal Statistical Office (Switzerland). Switzerland Medical Statistics of Hospitals 2014
Stroke	National Statistical Institute of Bulgaria. Bulgaria Living Standards Measurement Survey 2003. Washington DC, United States: World Bank
Stroke	Administrative Department of Science, Technology, and Innovation (Colombia), Center for Development Projects, Pontifical Xavierian University, Ministry of Social Protection (Colombia), Specialized Information Systems. Colombia National Health Survey 2007-2008
Stroke	Planning Institute of Jamaica, Statistical Institute of Jamaica. Jamaica Survey of Living Conditions 1988. Kingston, Jamaica: Planning Institute of Jamaica
Stroke	Statistical Institute of Jamaica. Jamaica Survey of Living Conditions 1990. Washington DC, United States: World Bank
Stroke	Planning Institute of Jamaica, Statistical Institute of Jamaica. Jamaica Survey of Living Conditions 1992
Stroke	Planning Institute of Jamaica, Statistical Institute of Jamaica. Jamaica Survey of Living Conditions 1991
Stroke	World Health Organization (WHO). Austria World Health Survey 2003. Geneva, Switzerland: World Health Organization (WHO), 2005
Stroke	Center for Scientific and Technological Information, Oswaldo Cruz Foundation and World Health Organization (WHO). Brazil World Health Survey 2003. Geneva, Switzerland: World Health Organization (WHO), 2005
Stroke	World Health Organization (WHO). Côte d'Ivoire World Health Survey 2003. Geneva, Switzerland: World Health Organization (WHO), 2005
Stroke	World Health Organization (WHO). Georgia World Health Survey 2003. Geneva, Switzerland: World Health Organization (WHO), 2005
Stroke	World Health Organization (WHO). Israel World Health Survey 2003. Geneva, Switzerland: World Health Organization (WHO), 2005
Stroke	World Health Organization (WHO). Myanmar World Health Survey 2003. Geneva, Switzerland: World Health Organization (WHO), 2005
Stroke	World Health Organization (WHO). Namibia World Health Survey 2003. Geneva, Switzerland: World Health Organization (WHO), 2005
Stroke	World Health Organization (WHO). Pakistan World Health Survey 2003-2004. Geneva, Switzerland: World Health Organization (WHO), 2005
Stroke	World Health Organization (WHO). Slovenia World Health Survey 2003. Geneva, Switzerland: World Health Organization (WHO), 2005
Stroke	World Health Organization (WHO). United Kingdom World Health Survey 2004. Geneva, Switzerland: World Health Organization (WHO), 2005
Stroke	Office of Population Censuses and Surveys. Social Survey Division, Health Survey for England, 1993 [Computer file]. Colchester, Essex: UK Data Archive [distributor], April 1995. SN: 3316, http://dx.doi.org/10.5255/UKDA-SN-3316-1
Stroke	Joint Health Surveys Unit of Social and Community Planning Research and University College London, Health Survey for England, 1994 [computer file]. 4th ed. Colchester, Essex: UK Data Archive [distributor], 26 March 2001. SN: 3640
Stroke	National Centre for Social Research, University College London Department of Epidemiology and Public Health, Health Survey for England, 1998 [computer file]. 4th ed. Colchester, Essex: UK Data Archive [distributor], 30 November 2002. SN: 4150
Stroke	National Centre for Social Research, University College London Department of Epidemiology and Public Health, Health Survey for England, 2000 [computer file]. Colchester, Essex: UK Data Archive [distributor], 23 April 2002. SN: 4487
Stroke	National Centre for Social Research and University College London. Department of Epidemiology and Public Health, Health Survey for England, 2003 [computer file]. Colchester, Essex: UK Data Archive [distributor], March 2005. SN: 5098
Stroke	National Centre for Social Research and University College London. Department of Epidemiology and Public Health, Health Survey for England, 2005 [computer file]. Colchester, Essex: UK Data Archive [distributor], July 2007. SN: 5675
Stroke	National Centre for Social Research and University College London. Department of Epidemiology and Public Health, Health Survey for England, 2006 [computer file]. 4th Edition. Colchester, Essex: UK Data Archive [distributor], July 2011. SN: 5809, http://dx.doi.org/10.5255/UKDA-SN-5809-1
Stroke	University of Wisconsin-Madison, Inter-University Consortium for Political and Social Research (ICPSR), University of São Paulo. Brazil - São Paulo Survey on Health, Well-Being, and Aging in Latin America and the Caribbean 1999-2000. Ann Arbor, United States: Inter-University Consortium for Political and Social Research (ICPSR)
Stroke	Planning Institute of Jamaica, Statistical Institute of Jamaica. Jamaica Survey of Living Conditions 1993
Stroke	Planning Institute of Jamaica, Statistical Institute of Jamaica. Jamaica Survey of Living Conditions 1994
Stroke	Planning Institute of Jamaica, Statistical Institute of Jamaica. Jamaica Survey of Living Conditions 1995
Stroke	Planning Institute of Jamaica, Statistical Institute of Jamaica. Jamaica Survey of Living Conditions 1996
Stroke	Planning Institute of Jamaica, Statistical Institute of Jamaica. Jamaica Survey of Living Conditions 1997
Stroke	Planning Institute of Jamaica, Statistical Institute of Jamaica. Jamaica Survey of Living Conditions 1999
Stroke	Planning Institute of Jamaica, Statistical Institute of Jamaica. Jamaica Survey of Living Conditions 2000
Stroke	TNS BBSS, World Bank. Bulgaria Living Standards Measurement Survey 2001. Washington DC, United States: World Bank
Stroke	National Administrative Department of Statistics (Colombia). Colombia National Quality of Life Survey 1997. Bogotá, Colombia: National Administrative Department of Statistics (Colombia)
Stroke	National Administrative Department of Statistics (Colombia). Colombia National Quality of Life Survey 2008. Bogotá, Colombia: National Administrative Department of Statistics (Colombia)
Stroke	National Administrative Department of Statistics (Colombia). Colombia National Quality of Life Survey 2010. Bogotá, Colombia: National Administrative Department of Statistics (Colombia), 2012
Stroke	Pontificia Universidad Javeriana (Colombia), World Health Organization (WHO). Colombia WHO Multi-country Survey Study on Health and Health System Responsiveness 2000-2001. Geneva, Switzerland: World Health Organization (WHO)
Stroke	AMATEM (Turkey), Plaza Ltd. Research, World Health Organization (WHO). Turkey WHO Multi-country Survey Study on Health and Health System Responsiveness 2000-2001. Geneva, Switzerland: World Health Organization (WHO)
Stroke	Institute for Polling and Marketing (Georgia), World Health Organization (WHO). Georgia WHO Multi-country Survey Study on Health and Health System Responsiveness 2000-2001
Stroke	Turkish Statistical Institute. Turkey Health Interview Survey 2010. Ankara, Turkey: Turkish Statistical Institute
Stroke	NatCen Social Research and University College London. Department of Epidemiology and Public Health, Health Survey for England, 2011 [computer file]. Colchester, Essex: UK Data Archive [distributor], April 2013. SN: 7260, http://dx.doi.org/10.5255/UKDA-SN-7260-1
Stroke	Minelli C, Fu Fen L, Camara Minelli DP. Stroke Incidence, Prognosis, 30-Day, and 1-Year Case Fatality Rates in Matão, Brazil. Stroke. 2007; 38(11): 2906-11
Stroke	Cabral NL, Gonçalves ARR, Longo AL, Moro CHC, Costa G, Amaral CH, Fonseca L a M, Eluf-Neto J. Incidence of stroke subtypes, prognosis and prevalence of risk factors in Joinville, Brazil: a 2 year community based study. J Neurol Neurosurg Psychiatr. 2009; 80(7): 755-61

Stroke	Abdul-Ghaffar NU, el-Sonbaty MR, el-Din Abdul-Baky MS, Marafie AA, al-Said AM. Stroke in Kuwait: a three-year prospective study. Neuroepidemiology. 1997; 16(1): 40-7
Stroke	Börü UT, Oztürk E, Ta?demir M, Sur H. Living alone following first-ever stroke: a prospective study in Turkey identifying the risk factors and evaluating their effects. N Z Med J. 2007; 120(1255): U2559
Stroke	Du X, Sourbutts J, Cruickshank K, Alison Summers, Roberts N, Walton E, Holmes S. A Community Based Stroke Register in a High Risk Area for Stroke in North West England. J Epidemiol Community Health. 1997; 51(5): 472-8
Stroke	Kumral E, Ozkaya B, Sagduyu A, Sirin H, Vardarli E, Pehlivan M. The Ege Stroke Registry: A Hospital-Based Study in the Aegean Region, Izmir, Turkey. Cerebrovasc Dis. 1998; 8(5): 278-88
Stroke	Tsiskaridze A, Djibuti M, van Melle G, Lomidze G, Apridonidze S, Gauarashvili I, Piechowski-Jóźwiak B, Shakarishvili R, Bogousslavsky J. Stroke Incidence and 30-Day Case-Fatality in a Suburb of Tbilisi: Results of the First Prospective Population-Based Study in Georgia. Stroke. 2004; 35(11): 2523-8
Stroke	Al-Shammri S, Shahid Z, Ghali A, Mehndiratta MM, Swaminathan TR, Chadha G, Sharma PN, Akanji AO. Risk Factors, Subtypes and Outcome of Ischaemic Stroke in Kuwait - A Hospital-Based Study. Med Princ Pract. 2003; 12(4): 218-23
Stroke	Statistics Austria, World Health Organization (WHO). Austria WHO Multi-country Survey Study on Health and Health System Responsiveness 2000-2001. Geneva, Switzerland: World Health Organization (WHO)
Stroke	National Assembly for Wales, Welsh Health Survey, 1998 [computer file]. 2nd Edition. Colchester, Essex: UK Data Archive [distributor], February 2011. SN: 4176 , http://dx.doi.org/10.5255/UKDA-SN-4176-1
Stroke	National Centre for Social Research, Beaufort Research Limited and University College London. Department of Epidemiology and Public Health, Welsh Health Survey, 2003-2004 [computer file]. 2nd Edition. Colchester, Essex: UK Data Archive [distributor], February 2011. SN: 5692 , http://dx.doi.org/10.5255/UKDA-SN-5692-1
Stroke	National Centre for Social Research, Welsh Health Survey, 2005-2006 [computer file]. 2nd Edition. Colchester, Essex: UK Data Archive [distributor], February 2011. SN: 5750 , http://dx.doi.org/10.5255/UKDA-SN-5750-1
Stroke	National Centre for Social Research, Welsh Health Survey, 2007 [computer file]. 2nd Edition. Colchester, Essex: UK Data Archive [distributor], February 2011. SN: 6052, http://dx.doi.org/10.5255/UKDA-SN-6052-1
Stroke	National Centre for Social Research, Welsh Health Survey, 2008 [computer file]. 2nd Edition. Colchester, Essex: UK Data Archive [distributor], February 2011. SN: 6372
Stroke	National Centre for Social Research, Welsh Health Survey, 2009 [computer file]. 2nd Edition. Colchester, Essex: UK Data Archive [distributor], February 2011. SN: 6589, http://dx.doi.org/10.5255/UKDA-SN-6589-1
Stroke	National Centre for Social Research, Welsh Health Survey, 2010 [computer file]. Colchester, Essex: UK Data Archive [distributor], November 2011. SN: 6895, http://dx.doi.org/10.5255/UKDA-SN-6895-1
Stroke	NatCen Social Research, Welsh Health Survey, 2011 [computer file]. Colchester, Essex: UK Data Archive [distributor], January 2013. SN: 7188, http://dx.doi.org/10.5255/UKDA-SN-7188-1
Stroke	NatCen Social Research, Welsh Health Survey, 2012 [computer file]. Colchester, Essex: UK Data Archive [distributor], February 2014. SN: 7459, http://dx.doi.org/10.5255/UKDA-SN-7459-1
Stroke	Federal Ministry of Health (Austria), Statistics Austria. Austria Hospital Inpatient Discharges 1989. Vienna, Austria: Statistics Austria
Stroke	National Institute of Statistics of Rwanda. Rwanda Integrated Household Living Conditions Survey 2010-2011. Kigali, Rwanda: National Institute of Statistics of Rwanda
Stroke	Health Institute (São Paulo, Brazil), State University of Campinas, São Paulo Municipal Health Department, São Paulo State University, University of São Paulo. Brazil - São Paulo Health Survey 2008-2009
Stroke	NatCen Social Research, Welsh Health Survey, 2013 [computer file]. Colchester, Essex: UK Data Archive [distributor], January 2015. SN: 7632, http://dx.doi.org/10.5255/UKDA-SN-7632-1
Stroke	Brazilian Institute of Geography and Statistics (IBGE), Ministry of Health (Brazil), Ministry of Planning, Budget, and Management (Brazil). Brazil National Health Survey 2013. Rio de Janeiro, Brazil: Brazilian Institute of Geography and Statistics (IBGE)
Stroke	Federal Ministry of Health (Austria), Statistics Austria. Austria Hospital Inpatient Discharges 2013. Vienna, Austria: Statistics Austria
Stroke	International Research Associates (INRA) Europe, World Health Organization (WHO). Bulgaria WHO Multi-country Survey Study on Health and Health System Responsiveness 2000-2001. Geneva, Switzerland: World Health Organization (WHO)
Stroke	National Centre for Social Research (NatCen), World Health Organization (WHO). United Kingdom WHO Multi-country Survey Study on Health and Health System Responsiveness 2000-2001. Geneva, Switzerland: World Health Organization (WHO)
Stroke	Börsch-Supan, A. (2015). Survey of Health, Ageing and Retirement in Europe (SHARE) Wave 5. Release version: 1.0.0. SHARE-ERIC. Data set. DOI: 10.6103/SHARE.w5.100
Stroke	Börsch-Supan, A. (2015). Survey of Health, Ageing and Retirement in Europe (SHARE) Wave 5. Release version: 1.0.0. SHARE-ERIC. Data set. DOI: 10.6103/SHARE.w5.100
Stroke	Börsch-Supan, A. (2015). Survey of Health, Ageing and Retirement in Europe (SHARE) Wave 5. Release version: 1.0.0. SHARE-ERIC. Data set. DOI: 10.6103/SHARE.w5.100
Stroke	National Center for Disease Control and Public Health (Georgia). Georgia Hospital Data 2014
Stroke	Concluzia-Prim Center for Survey Methodology (Moldova), Georgia Opinion Research Business International (GORBI), Institute for Advanced Studies (Austria), London School of Hygiene and Tropical Medicine, University of Aberdeen. Georgia Health in Times of Transition Household Survey 2010
Stroke	Federal Ministry of Health (Austria), Statistics Austria. Austria Hospital Inpatient Discharges 1989-1992
Stroke	Federal Ministry of Health (Austria), Statistics Austria. Austria Hospital Inpatient Discharges 1993-1997
Stroke	Federal Ministry of Health (Austria), Statistics Austria. Austria Hospital Inpatient Discharges 1998-2002
Stroke	Federal Ministry of Health (Austria), Statistics Austria. Austria Hospital Inpatient Discharges 2003-2007
Stroke	Federal Ministry of Health (Austria), Statistics Austria. Austria Hospital Inpatient Discharges 2008-2012
Stroke	Ministry of Labor and Social Policy (Bulgaria), National Statistical Institute of Bulgaria, TNS Gallup, World Bank. Bulgaria Multitopic Household Survey 2007. Washington DC, United States: World Bank
Stroke	Federal Ministry of Health (Austria), Statistics Austria. Austria Hospital Inpatient Discharges 2014. Vienna, Austria: Statistics Austria
Stroke	Federal Ministry of Health (Austria), Statistics Austria. Austria Hospital Inpatient Discharges 2013-2014
Stroke	National Institute of Public Health (Slovenia). Slovenia National Hospital Health Care Statistics Database 2004
Stroke	National Institute of Public Health (Slovenia). Slovenia National Hospital Health Care Statistics Database 2005
Stroke	National Institute of Public Health (Slovenia). Slovenia National Hospital Health Care Statistics Database 2006
Stroke	National Institute of Public Health (Slovenia). Slovenia National Hospital Health Care Statistics Database 2007
Stroke	National Institute of Public Health (Slovenia). Slovenia National Hospital Health Care Statistics Database 2008
Stroke	General Directorate of Curative Services, Ministry of Health (Turkey). Turkey Hospital Inpatient Discharges 2007
Stroke	General Directorate of Curative Services, Ministry of Health (Turkey). Turkey Hospital Inpatient Discharges 2008
Stroke	General Directorate of Curative Services, Ministry of Health (Turkey). Turkey Hospital Inpatient Discharges 2009
Stroke	General Directorate of Curative Services, Ministry of Health (Turkey). Turkey Hospital Inpatient Discharges 2010
Stroke	General Directorate of Curative Services, Ministry of Health (Turkey). Turkey Hospital Inpatient Discharges 2011

Stroke	National Center for Disease Control and Public Health (Georgia). Georgia Inpatient Care Discharges per 100 1991
Stroke	National Center for Disease Control and Public Health (Georgia). Georgia Inpatient Care Discharges per 100 1992
Stroke	National Center for Disease Control and Public Health (Georgia). Georgia Inpatient Care Discharges per 100 1993
Stroke	National Center for Disease Control and Public Health (Georgia). Georgia Inpatient Care Discharges per 100 1994
Stroke	National Center for Disease Control and Public Health (Georgia). Georgia Inpatient Care Discharges per 100 1995
Stroke	National Center for Disease Control and Public Health (Georgia). Georgia Inpatient Care Discharges per 100 1996
Stroke	National Center for Disease Control and Public Health (Georgia). Georgia Inpatient Care Discharges per 100 1997
Stroke	National Center for Disease Control and Public Health (Georgia). Georgia Inpatient Care Discharges per 100 1998
Stroke	National Center for Disease Control and Public Health (Georgia). Georgia Inpatient Care Discharges per 100 1999
Stroke	National Center for Disease Control and Public Health (Georgia). Georgia Inpatient Care Discharges per 100 2000
Stroke	National Center for Disease Control and Public Health (Georgia). Georgia Inpatient Care Discharges per 100 2001
Stroke	National Center for Disease Control and Public Health (Georgia). Georgia Inpatient Care Discharges per 100 2002
Stroke	National Center for Disease Control and Public Health (Georgia). Georgia Inpatient Care Discharges per 100 2003
Stroke	National Center for Disease Control and Public Health (Georgia). Georgia Inpatient Care Discharges per 100 2004
Stroke	National Center for Disease Control and Public Health (Georgia). Georgia Inpatient Care Discharges per 100 2005
Stroke	National Center for Disease Control and Public Health (Georgia). Georgia Inpatient Care Discharges per 100 2006
Stroke	National Center for Disease Control and Public Health (Georgia). Georgia Inpatient Care Discharges per 100 2007
Stroke	National Center for Disease Control and Public Health (Georgia). Georgia Inpatient Care Discharges per 100 2008
Stroke	National Center for Disease Control and Public Health (Georgia). Georgia Inpatient Care Discharges per 100 2009
Stroke	National Center for Disease Control and Public Health (Georgia). Georgia Inpatient Care Discharges per 100 2010
Stroke	National Center for Disease Control and Public Health (Georgia). Georgia Inpatient Care Discharges per 100 2011
Stroke	National Center for Disease Control and Public Health (Georgia). Georgia Inpatient Care Discharges per 100 2012
Stroke	National Center for Disease Control and Public Health (Georgia). Georgia Inpatient Care Discharges per 100 2013
Stroke	National Center for Disease Control and Public Health (Georgia). Georgia Inpatient Care Discharges per 100 2014
Stroke	Ministry of Health (Israel). Israel National Hospital Discharge Database 2011
Stroke	Ministry of Health (Israel). Israel National Hospital Discharge Database 2012
Stroke	Ministry of Health (Israel). Israel National Hospital Discharge Database 2013
Stroke	Ministry of Health (Israel). Israel National Hospital Discharge Database 2014
Stroke	National Center for Disease Control and Public Health (Georgia). Georgia Inpatient Care Discharges per 100 1980
Stroke	National Center for Disease Control and Public Health (Georgia). Georgia Inpatient Care Discharges per 100 1985
Stroke	National Center for Disease Control and Public Health (Georgia). Georgia Inpatient Care Discharges per 100 1986
Stroke	National Center for Disease Control and Public Health (Georgia). Georgia Inpatient Care Discharges per 100 1987
Stroke	National Center for Disease Control and Public Health (Georgia). Georgia Inpatient Care Discharges per 100 1988
Stroke	National Center for Disease Control and Public Health (Georgia). Georgia Inpatient Care Discharges per 100 1989
Stroke	National Center for Disease Control and Public Health (Georgia). Georgia Inpatient Care Discharges per 100 1990
Stroke	General Directorate of Curative Services, Ministry of Health (Turkey). Turkey Inpatient Care Discharges per 100 1980
Stroke	General Directorate of Curative Services, Ministry of Health (Turkey). Turkey Inpatient Care Discharges per 100 1981
Stroke	General Directorate of Curative Services, Ministry of Health (Turkey). Turkey Inpatient Care Discharges per 100 1982
Stroke	General Directorate of Curative Services, Ministry of Health (Turkey). Turkey Inpatient Care Discharges per 100 1983
Stroke	General Directorate of Curative Services, Ministry of Health (Turkey). Turkey Inpatient Care Discharges per 100 1984
Stroke	General Directorate of Curative Services, Ministry of Health (Turkey). Turkey Inpatient Care Discharges per 100 1985
Stroke	General Directorate of Curative Services, Ministry of Health (Turkey). Turkey Inpatient Care Discharges per 100 1986
Stroke	General Directorate of Curative Services, Ministry of Health (Turkey). Turkey Inpatient Care Discharges per 100 1987
Stroke	General Directorate of Curative Services, Ministry of Health (Turkey). Turkey Inpatient Care Discharges per 100 1988
Stroke	General Directorate of Curative Services, Ministry of Health (Turkey). Turkey Inpatient Care Discharges per 100 1989
Stroke	General Directorate of Curative Services, Ministry of Health (Turkey). Turkey Inpatient Care Discharges per 100 1990
Stroke	General Directorate of Curative Services, Ministry of Health (Turkey). Turkey Inpatient Care Discharges per 100 1991
Stroke	General Directorate of Curative Services, Ministry of Health (Turkey). Turkey Inpatient Care Discharges per 100 1992
Stroke	General Directorate of Curative Services, Ministry of Health (Turkey). Turkey Inpatient Care Discharges per 100 1993
Stroke	General Directorate of Curative Services, Ministry of Health (Turkey). Turkey Inpatient Care Discharges per 100 1994
Stroke	General Directorate of Curative Services, Ministry of Health (Turkey). Turkey Inpatient Care Discharges per 100 1995
Stroke	General Directorate of Curative Services, Ministry of Health (Turkey). Turkey Inpatient Care Discharges per 100 1996
Stroke	General Directorate of Curative Services, Ministry of Health (Turkey). Turkey Inpatient Care Discharges per 100 1997
Stroke	General Directorate of Curative Services, Ministry of Health (Turkey). Turkey Inpatient Care Discharges per 100 1998
Stroke	General Directorate of Curative Services, Ministry of Health (Turkey). Turkey Inpatient Care Discharges per 100 1999
Stroke	General Directorate of Curative Services, Ministry of Health (Turkey). Turkey Inpatient Care Discharges per 100 2000
Stroke	General Directorate of Curative Services, Ministry of Health (Turkey). Turkey Inpatient Care Discharges per 100 2001
Stroke	General Directorate of Curative Services, Ministry of Health (Turkey). Turkey Inpatient Care Discharges per 100 2002
Stroke	General Directorate of Curative Services, Ministry of Health (Turkey). Turkey Inpatient Care Discharges per 100 2003
Stroke	General Directorate of Curative Services, Ministry of Health (Turkey). Turkey Inpatient Care Discharges per 100 2004
Stroke	General Directorate of Curative Services, Ministry of Health (Turkey). Turkey Inpatient Care Discharges per 100 2005
Stroke	General Directorate of Curative Services, Ministry of Health (Turkey). Turkey Inpatient Care Discharges per 100 2006
Stroke	General Directorate of Curative Services, Ministry of Health (Turkey). Turkey Inpatient Care Discharges per 100 2012
Stroke	General Directorate of Curative Services, Ministry of Health (Turkey). Turkey Inpatient Care Discharges per 100 2013
Stroke	General Directorate of Curative Services, Ministry of Health (Turkey). Turkey Inpatient Care Discharges per 100 2014
Stroke	National Institute of Public Health (Slovenia). Slovenia National Hospital Health Care Statistics Database 1980
Stroke	National Institute of Public Health (Slovenia). Slovenia National Hospital Health Care Statistics Database 1986
Stroke	National Institute of Public Health (Slovenia). Slovenia National Hospital Health Care Statistics Database 1985
Stroke	National Institute of Public Health (Slovenia). Slovenia National Hospital Health Care Statistics Database 1987
Stroke	National Institute of Public Health (Slovenia). Slovenia National Hospital Health Care Statistics Database 1988
Stroke	National Institute of Public Health (Slovenia). Slovenia National Hospital Health Care Statistics Database 1989
Stroke	National Institute of Public Health (Slovenia). Slovenia National Hospital Health Care Statistics Database 1990
Stroke	National Institute of Public Health (Slovenia). Slovenia National Hospital Health Care Statistics Database 1991
Stroke	National Institute of Public Health (Slovenia). Slovenia National Hospital Health Care Statistics Database 1992
Stroke	National Institute of Public Health (Slovenia). Slovenia National Hospital Health Care Statistics Database 1993

Stroke	National Institute of Public Health (Slovenia). Slovenia National Hospital Health Care Statistics Database 1994
Stroke	National Institute of Public Health (Slovenia). Slovenia National Hospital Health Care Statistics Database 1995
Stroke	National Institute of Public Health (Slovenia). Slovenia National Hospital Health Care Statistics Database 1996
Stroke	National Institute of Public Health (Slovenia). Slovenia National Hospital Health Care Statistics Database 1997
Stroke	National Institute of Public Health (Slovenia). Slovenia National Hospital Health Care Statistics Database 1998
Stroke	National Institute of Public Health (Slovenia). Slovenia National Hospital Health Care Statistics Database 1999
Stroke	National Institute of Public Health (Slovenia). Slovenia National Hospital Health Care Statistics Database 2000
Stroke	National Institute of Public Health (Slovenia). Slovenia National Hospital Health Care Statistics Database 2001
Stroke	National Institute of Public Health (Slovenia). Slovenia National Hospital Health Care Statistics Database 2002
Stroke	National Institute of Public Health (Slovenia). Slovenia National Hospital Health Care Statistics Database 2003
Stroke	National Institute of Public Health (Slovenia). Slovenia National Hospital Health Care Statistics Database 2013
Stroke	National Institute of Public Health (Slovenia). Slovenia National Hospital Health Care Statistics Database 2014
Stroke	Bahamas Department of Statistics, Ministry of Health (The Bahamas). Bahamas Living Conditions Survey 2001. Nassau, The Bahamas: Bahamas Department of Statistics
Stroke	Agency of the Republic of Kazakhstan on Statistics, World Bank. Kazakhstan Living Standards Measurement Survey 1996. Washington DC, United States: World Bank
Stroke	Central Statistical Service (South Africa). South Africa October Household Survey 1996
Stroke	World Health Organization (WHO). Belgium World Health Survey 2002. Geneva, Switzerland: World Health Organization (WHO), 2005
Stroke	World Health Organization (WHO). China World Health Survey 2002. Geneva, Switzerland: World Health Organization (WHO), 2005
Stroke	World Health Organization (WHO). Comoros World Health Survey 2003. Geneva, Switzerland: World Health Organization (WHO), 2005
Stroke	World Health Organization (WHO). Croatia World Health Survey 2003. Geneva, Switzerland: World Health Organization (WHO), 2005
Stroke	World Health Organization (WHO). Italy World Health Survey 2003. Geneva, Switzerland: World Health Organization (WHO), 2005
Stroke	World Health Organization (WHO). Kazakhstan World Health Survey 2002-2003. Geneva, Switzerland: World Health Organization (WHO), 2005
Stroke	World Health Organization (WHO). Paraguay World Health Survey 2002-2003. Geneva, Switzerland: World Health Organization (WHO), 2005
Stroke	World Health Organization (WHO). Philippines World Health Survey 2003. Geneva, Switzerland: World Health Organization (WHO), 2005
Stroke	World Health Organization (WHO). Senegal World Health Survey 2003. Geneva, Switzerland: World Health Organization (WHO), 2005
Stroke	World Health Organization (WHO). United Arab Emirates World Health Survey 2003. Geneva, Switzerland: World Health Organization (WHO), 2005
Stroke	Ministry of Health (China), National Center for Chronic and Noncommunicable Disease Control and Prevention (China), World Health Organization (WHO). China WHO Study on Global AGEing and Adult Health 2007-2010
Stroke	Carolina Population Center, University of North Carolina at Chapel Hill, Chinese Center for Disease Control and Prevention (CCDC). China Health and Nutrition Survey. Chapel Hill, United States: Carolina Population Center, University of North Carolina at Chapel Hill
Stroke	Ministry of Public Health (Lebanon), World Health Organization (WHO). Lebanon WHO Multi-country Survey Study on Health and Health System Responsiveness 2000-2001
Stroke	Institute of Social Medicine and Health Policy, Shandong University, Shandong University School of Medicine, World Health Organization (WHO). China WHO Multi-country Survey Study on Health and Health System Responsiveness 2000-2001
Stroke	Carolei A, Marini C, Di Napoli M, Di Gianfilippo G, Santalucia P, Baldassarre M, Giorgio De Matteis M, di Orio F. High Stroke Incidence in the Prospective Community-Based L'Aquila Registry (1994-1998): First Year's Results. Stroke. 1997; 28(12): 2500-6
Stroke	Di Carlo A, Inzitari D, Galati F, Baldereschi M, Giunta V, Grillo G, Furchì A, Manno V, Naso F, Vecchio A, Consoli D. A Prospective Community-Based Study of Stroke in Southern Italy: The Vibo Valentia Incidence of Stroke Study (VISS). Cerebrovasc Dis. 2003; 16(4): 410-7
Stroke	Manobianca G, Zoccolella S, Petruzzellis A, Miccoli A, Logroscino G. The incidence of major stroke subtypes in Southern Italy: a population based study. Eur J Neurol. 2010; 17(9): 1148-55
Stroke	Corso G, Bottacchi E, Giardini G, De la Pierre F, Meloni T, Pesenti Campagnoni M, Ponzetti C, Veronese Morosini M. Community-based study of stroke incidence in the Valley of Aosta, Italy. CARe-cerebrovascular Aosta Registry: years 2004-2005. Neuroepidemiology. 2009; 32(3): 186-95
Stroke	Lauria G, Gentile M, Fassetta G, Casetta I, Agnoli F, Andreotta G, Barp C, Caneve G, Cavallaro A, Cielo R, Mongillo D, Mosca M, Olivieri P. Incidence and Prognosis of Stroke in the Belluno Province, Italy: First-Year Results of a Community-Based Study. Stroke. 1995; 26(10): 1787-93
Stroke	D'Alessandro G, Bottacchi E, Di Giovanni M, Martinazzo C, Sironi L, Lia C, Carenini L, Corso G, Gerbaz V, Polillo C, Pesenti Compagnoni M. Temporal trends of stroke in Valle d'Aosta, Italy. Incidence and 30-day fatality rates. Neurol Sci. 2000; 21(1): 13-8
Stroke	Manobianca G, Zoccolella S, Petruzzellis A, Miccoli A, Logroscino G. Low Incidence of Stroke in Southern Italy: A Population-Based Study. Stroke. 2008; 39(11): 2923-8
Stroke	Hong Y, Bots ML, Pan X, Hofman A, Grobbee DE, Chen H. Stroke Incidence and Mortality in Rural and Urban Shanghai From 1984 Through 1991: Findings From a Community-Based Registry. Stroke. 1994; 25(6): 1165-9
Stroke	Zhao D, Liu J, Wang W, Zeng Z, Cheng J, Liu J, Sun J, Wu Z. Epidemiological Transition of Stroke in China Twenty-One-Year Observational Study From the Sino-MONICA-Beijing Project. Stroke. 2008; 39(6): 1668-74
Stroke	Central Statistical Service (South Africa). South Africa October Household Survey 1994
Stroke	Musolino R LSP. First-ever stroke incidence and 30-day case fatality in the Sicilian Aeolian archipelago, Italy. Stroke. 2005; 36(12): 2738-41
Stroke	Orlandi G, Gelli A, Fanucchi S, Tognoni G, Acerbi G, Murri L. Prevalence of stroke and transient ischaemic attack in the elderly population of an Italian rural community. Eur J Epidemiol. 2003; 18(9): 879-82
Stroke	Prencipe M, Ferretti C, Casini AR, Santini M, Giubilei F, Culasso F. Stroke, disability, and dementia: results of a population survey. Stroke. 1997; 28(3): 531-6
Stroke	Bonzini M, Ferrario MM, Bertù L, Bono G, Vidale S, Veronesi G, Chambless L, Cesana GC. Temporal trends in ischemic and hemorrhagic strokes in Northern Italy: results from the cardiovascular monitoring unit in Northern Italy population-based register, 1998-2004. Neuroepidemiology. 2012; 39(1): 35-42
Stroke	Pikija S, Cvetko D, Malojcic B, Trkanjec Z, Pavlicek I, Lukic A, Kopjar A, Hajduk M, Androvic A, Bilic-Genter M, Trkulja V. A population-based prospective 24-month study of stroke: incidence and 30-day case-fatality rates of first-ever strokes in Croatia. Neuroepidemiology. 2012; 38(3): 164-71
Stroke	D'Alessandro G, Gallo F, Vitaliano A, Col PD, Gorraz F, Cristofaro RD, Boaretto G. Prevalence of stroke and stroke-related disability in Valle d'Aosta, Italy. Neurol Sci. 2010; 31(2): 137-41
Stroke	Zhao Y, Yao Z, D'Souza W, Zhu C, Chun H, Zhuoga C, Zhang Q, Hu X, Zhou D. An Epidemiological Survey of Stroke in Lhasa, Tibet, China. Stroke. 2010; 41(12): 2739-43
Stroke	China Center for Economic Research, Peking University. China Health and Retirement Longitudinal Study Pilot Resurvey 2012. Beijing, China: China Center for Economic Research, Peking University
Stroke	Sacco S, Stracci F, Cerone D, Ricci S, Carolei A. Epidemiology of stroke in Italy. Int J Stroke. 2011; 6(3): 219-27
Stroke	Human Sciences Research Council, South African Medical Research Council. South Africa National Health and Nutrition Examination Survey 2012
Stroke	Wu GX, Wu ZS, He BL. [Epidemiological characteristics of stroke in 16 provinces of China]. Nat Med J Chin. 1994; 74: 281–283
Stroke	Central American Population Center, University of Costa Rica. Costa Rica Survey of Family Health Services and Expenses 2008. San José, Costa Rica: Central American Population Center, University of Costa Rica

Stroke	Gallup Europe, World Health Organization (WHO). United Arab Emirates WHO Multi-country Survey Study on Health and Health System Responsiveness 2000-2001. Geneva, Switzerland: World Health Organization (WHO)
Stroke	International Research Associates (INRA) Europe, World Health Organization (WHO). Belgium WHO Multi-country Survey Study on Health and Health System Responsiveness 2000-2001. Geneva, Switzerland: World Health Organization (WHO)
Stroke	Gallup Europe, World Health Organization (WHO). Costa Rica WHO Multi-country Survey Study on Health and Health System Responsiveness 2000-2001. Geneva, Switzerland: World Health Organization (WHO)
Stroke	Market, Media, and Public Opinion Research (Croatia), World Health Organization (WHO). Croatia WHO Multi-country Survey Study on Health and Health System Responsiveness 2000-2001. Geneva, Switzerland: World Health Organization (WHO)
Stroke	International Research Associates (INRA) Europe, World Health Organization (WHO). Italy WHO Multi-country Survey Study on Health and Health System Responsiveness 2000-2001. Geneva, Switzerland: World Health Organization (WHO)
Stroke	Börsch-Supan, A. (2015). Survey of Health, Ageing and Retirement in Europe (SHARE) Wave 5. Release version: 1.0.0. SHARE-ERIC. Data set. DOI: 10.6103/SHARE.w5.100
Stroke	Börsch-Supan, A. (2015). Survey of Health, Ageing and Retirement in Europe (SHARE) Wave 5. Release version: 1.0.0. SHARE-ERIC. Data set. DOI: 10.6103/SHARE.w5.100
Stroke	Center for Study of Public Opinion (Kazakhstan), Concluzia-Prim Center for Survey Methodology (Moldova), Institute for Advanced Studies (Austria), London School of Hygiene and Tropical Medicine, University of Aberdeen. Kazakhstan Health in Times of Transition Household Survey 2010
Stroke	Wang W, Jiang B, Sun H, Ru X, Sun D, Wang L, Wang L, Jiang Y, Li Y, Wang Y, Chen Z, Wu S, Zhang Y, Wang D, Wang Y, Feigin VL; NESS-China investigators. Prevalence, Incidence and Mortality of Stroke in China: Results from a Nationwide Population-Based Survey of 480,687 Adults. Circulation. 2017; 135(8): 759-71
Stroke	Center for Health Statistics and Information, National Health and Family Planning Commission (China). China National Health Statistical Information Reporting System 2013-2015
Stroke	Institute for Maternal and Child Health - IRCCS “Burlo Garofolo”. Italy - Friuli Venezia Giulia Hospital Inpatient Discharges 2010-2012
Stroke	Institute for Maternal and Child Health - IRCCS “Burlo Garofolo”. Italy - Friuli Venezia Giulia Hospital Inpatient Discharges 2013-2015
Stroke	Ricci S, Celani MG, La Rosa F, Vitali R, Duca E, Ferraguzzi R, Paolotti M, Seppoloni D, Caputo N, Chiurulla C, Scaroni R, Signorini E. SEPIVAC: a Community-based Study of Stroke Incidence in Umbria, Italy. J Neurol Neurosurg Psychiatry. 1991; 54(8): 695–8
Stroke	Ministry of Health (Italy). Italy National Hospital Discharge Database 2012
Stroke	Federal Public Service Health, Food Chain Safety, and Environment (Belgium). Belgium Minimum Clinical Summary 2000
Stroke	Federal Public Service Health, Food Chain Safety, and Environment (Belgium). Belgium Minimum Clinical Summary 2001
Stroke	Federal Public Service Health, Food Chain Safety, and Environment (Belgium). Belgium Minimum Clinical Summary 2002
Stroke	Federal Public Service Health, Food Chain Safety, and Environment (Belgium). Belgium Minimum Clinical Summary 2005
Stroke	Federal Public Service Health, Food Chain Safety, and Environment (Belgium). Belgium Minimum Clinical Summary 2006
Stroke	Federal Public Service Health, Food Chain Safety, and Environment (Belgium). Belgium Minimum Clinical Summary 2007
Stroke	Federal Public Service Health, Food Chain Safety, and Environment (Belgium). Belgium Minimum Hospital Summary 2010
Stroke	Federal Public Service Health, Food Chain Safety, and Environment (Belgium). Belgium Minimum Hospital Summary 2011
Stroke	Federal Public Service Health, Food Chain Safety, and Environment (Belgium). Belgium Minimum Hospital Summary 2012
Stroke	Federal Public Service Health, Food Chain Safety, and Environment (Belgium). Belgium Minimum Hospital Summary 2013
Stroke	Ministry of Health (Italy). Italy National Hospital Discharge Database 1970
Stroke	Ministry of Health (Italy). Italy National Hospital Discharge Database 1971
Stroke	Ministry of Health (Italy). Italy National Hospital Discharge Database 1972
Stroke	Ministry of Health (Italy). Italy National Hospital Discharge Database 1973
Stroke	Ministry of Health (Italy). Italy National Hospital Discharge Database 1974
Stroke	Ministry of Health (Italy). Italy National Hospital Discharge Database 1975
Stroke	Ministry of Health (Italy). Italy National Hospital Discharge Database 1976
Stroke	Ministry of Health (Italy). Italy National Hospital Discharge Database 1977
Stroke	Ministry of Health (Italy). Italy National Hospital Discharge Database 1978
Stroke	Ministry of Health (Italy). Italy National Hospital Discharge Database 1979
Stroke	Ministry of Health (Italy). Italy National Hospital Discharge Database 1980
Stroke	Ministry of Health (Italy). Italy National Hospital Discharge Database 1981
Stroke	Ministry of Health (Italy). Italy National Hospital Discharge Database 1982
Stroke	Ministry of Health (Italy). Italy National Hospital Discharge Database 1983
Stroke	Ministry of Health (Italy). Italy National Hospital Discharge Database 1984
Stroke	Ministry of Health (Italy). Italy National Hospital Discharge Database 1985
Stroke	Ministry of Health (Italy). Italy National Hospital Discharge Database 1986
Stroke	Ministry of Health (Italy). Italy National Hospital Discharge Database 1987
Stroke	Ministry of Health (Italy). Italy National Hospital Discharge Database 1988
Stroke	Ministry of Health (Italy). Italy National Hospital Discharge Database 1989
Stroke	Ministry of Health (Italy). Italy National Hospital Discharge Database 1990
Stroke	Ministry of Health (Italy). Italy National Hospital Discharge Database 1991
Stroke	Ministry of Health (Italy). Italy National Hospital Discharge Database 1992
Stroke	Ministry of Health (Italy). Italy National Hospital Discharge Database 1993
Stroke	Ministry of Health (Italy). Italy National Hospital Discharge Database 1994
Stroke	Ministry of Health (Italy). Italy National Hospital Discharge Database 1995
Stroke	Ministry of Health (Italy). Italy National Hospital Discharge Database 1996
Stroke	Ministry of Health (Italy). Italy National Hospital Discharge Database 1997
Stroke	Ministry of Health (Italy). Italy National Hospital Discharge Database 1998
Stroke	Ministry of Health (Italy). Italy National Hospital Discharge Database 1999
Stroke	Ministry of Health (Italy). Italy National Hospital Discharge Database 2000
Stroke	Ministry of Health (Italy). Italy National Hospital Discharge Database 2013
Stroke	Ministry of Health (Italy). Italy National Hospital Discharge Database 2014
Stroke	National Bureau of Statistics of China. China Statistical Yearbook 2015. Beijing, China: National Bureau of Statistics of China
Stroke	Ghana Statistical Service. Ghana Living Standards Measurement Survey 1991-1992. Accra, Ghana: Ghana Statistical Service
Stroke	Ghana Statistical Service. Ghana Living Standards Survey 1998-1999
Stroke	Ghana Statistical Service. Ghana Living Standards Measurement Survey 2005-2006. Accra, Ghana: Ghana Statistical Service
Stroke	Institute of Sociology, Russian Academy of Sciences, Paragon Research, University of North Carolina, World Bank. Kyrgyzstan Living Standards Measurement Survey 1993. Washington DC, United States: World Bank

Stroke	World Health Organization (WHO). Czech Republic World Health Survey 2002-2003. Geneva, Switzerland: World Health Organization (WHO), 2005
Stroke	World Health Organization (WHO). Ghana World Health Survey 2003. Geneva, Switzerland: World Health Organization (WHO), 2005
Stroke	World Health Organization (WHO). Luxembourg World Health Survey 2003. Geneva, Switzerland: World Health Organization (WHO), 2005
Stroke	World Health Organization (WHO). Sri Lanka World Health Survey 2003. Geneva, Switzerland: World Health Organization (WHO), 2005
Stroke	World Health Organization (WHO). Swaziland World Health Survey 2003. Geneva, Switzerland: World Health Organization (WHO), 2005
Stroke	University of Wisconsin-Madison, Inter-University Consortium for Political and Social Research (ICPSR), Chronic Disease Research Centre (CDRC), University of the West Indies. Barbados - Bridgetown Survey on Health, Well-Being, and Aging in Latin America and the Caribbean 1999-2000. Ann Arbor, United States: Inter-University
Stroke	National Statistical Committee of the Kyrgyz Republic, Research Triangle Institute, Inc. (RTI), World Bank. Kyrgyzstan Living Standards Measurement Survey 1997. Washington DC, United States: World Bank
Stroke	National Statistical Committee of the Kyrgyz Republic, Research Triangle Institute, Inc. (RTI), World Bank. Kyrgyzstan Living Standards Measurement Survey 1998. Washington DC, United States: World Bank
Stroke	Ashok PP, Radhakrishnan K, Sridharan R, el-Mangoush MA. Incidence and pattern of cerebrovascular diseases in Benghazi, Libya. J Neurol Neurosurg Psychiatr. 1986; 49(5): 519-23
Stroke	Morikawa Y, Nakagawa H, Naruse Y, Nishijo M, Miura K, Tabata M, Hirokawa W, Kagamimori S, Honda M, Yoshita K, Hayashi K. Trends in Stroke Incidence and Acute Case Fatality in a Japanese Rural Area: The Oyabe Study. Stroke. 2000; 31(7): 1583-7
Stroke	Kita Y, Okayama A, Ueshima H, Wada M, Nozaki A, Choudhary SR, Bonita R, Inamoto Y, Kasamatsu T. Stroke incidence and case fatality in Shiga, Japan 1989-1993. Int J Epidemiol. 1999; 28(6): 1059-65
Stroke	Gallup Europe, World Health Organization (WHO). Argentina WHO Multi-country Survey Study on Health and Health System Responsiveness 2000-2001. Geneva, Switzerland: World Health Organization (WHO)
Stroke	Kita Y, Turin TC, Ichikawa M, Sugihara H, Morita Y, Tomioka N, Rumana N, Okayama A, Nakamura Y, Abbott RD, Ueshima H. Trend of stroke incidence in a Japanese population: Takashima stroke registry, 1990-2001. Int J Stroke. 2009; 4(4): 241-9
Stroke	Kulesh SD, Filina NA, Frantava NM, Zhytko NL, Kastsinevich TM, Kliatskova LA, Shumskas MS, Hilz MJ, Schwab S, Kolominsky-Rabas PL. Incidence and Case-Fatality of Stroke on the East Border of the European Union The Grodno Stroke Study. Stroke. 2010; 41(12): 2726-30
Stroke	Melcon CM, Melcon MO. Prevalence of stroke in an Argentine community. Neuroepidemiology. 2006; 27(2): 81-8
Stroke	Turin TC, Kita Y, Rumana N, Nakamura Y, Takashima N, Ichikawa M, Sugihara H, Morita Y, Hirose K, Okayama A, Miura K, Ueshima H. Ischemic Stroke Subtypes in a Japanese Population Takashima Stroke Registry, 1988-2004. Stroke. 2010; 41(9): 1871-6
Stroke	General Administration of Statistics and Censuses (El Salvador), Ministry of Economy (El Salvador). El Salvador Multipurpose Household Survey 2013. San Salvador, El Salvador: General Administration of Statistics and Censuses (El Salvador)
Stroke	Ghana Statistical Service, World Bank. Ghana Living Standards Measurement Survey 2012-2013. Accra, Ghana: Ghana Statistical Service
Stroke	MEMRB International, World Health Organization (WHO). Cyprus WHO Multi-country Survey Study on Health and Health System Responsiveness 2000-2001. Geneva, Switzerland: World Health Organization (WHO)
Stroke	Institute of Health Information and Statistics of the Czech Republic, International Research Associates (INRA) Europe, World Health Organization (WHO). Czech Republic WHO Multi-country Survey Study on Health and Health System Responsiveness 2000-2001. Geneva, Switzerland: World Health Organization (WHO)
Stroke	National Statistical Committee of the Kyrgyz Republic, SIAR Research and Consulting (Kyrgyzstan), World Health Organization (WHO). Kyrgyzstan WHO Multi-country Survey Study on Health and Health System Responsiveness 2000-2001. Geneva, Switzerland: World Health Organization (WHO)
Stroke	International Research Associates (INRA) Europe, World Health Organization (WHO). Luxembourg WHO Multi-country Survey Study on Health and Health System Responsiveness 2000-2001. Geneva, Switzerland: World Health Organization (WHO)
Stroke	Börsch-Supan, A. (2015). Survey of Health, Ageing and Retirement in Europe (SHARE) Wave 5. Release version: 1.0.0. SHARE-ERIC. Data set. DOI: 10.6103/SHARE.w5.100
Stroke	Börsch-Supan, A. (2015). Survey of Health, Ageing and Retirement in Europe (SHARE) Wave 5. Release version: 1.0.0. SHARE-ERIC. Data set. DOI: 10.6103/SHARE.w5.100
Stroke	Belarusian State University, Concluzia-Prim Center for Survey Methodology (Moldova), Institute for Advanced Studies (Austria), London School of Hygiene and Tropical Medicine, University of Aberdeen. Belarus Health in Times of Transition Household Survey 2010
Stroke	Studies (Austria), International Centre for Sociological, Political and Social Psychological Research (Kyrgyzstan), London School of Hygiene and Tropical Medicine, University of Aberdeen. Kyrgyzstan Health in Times of Transition Household Survey 2011
Stroke	General Administration of Statistics and Censuses (El Salvador), Ministry of Economy (El Salvador). El Salvador Multipurpose Household Survey 2014. San Salvador, El Salvador: General Administration of Statistics and Censuses (El Salvador)
Stroke	Department of Economics, University of Chile, Ministry of Planning (Chile). Chile National Socioeconomic Characterization Survey 1992
Stroke	Department of Economics, University of Chile, Ministry of Planning (Chile). Chile National Socioeconomic Characterization Survey 1994. Santiago, Chile: Ministry of Social Development (Chile)
Stroke	Department of Economics, University of Chile, Ministry of Planning (Chile). Chile National Socioeconomic Characterization Survey 1996. Santiago, Chile: Ministry of Social Development (Chile)
Stroke	Department of Economics, University of Chile, Ministry of Planning (Chile). Chile National Socioeconomic Characterization Survey 1998
Stroke	Department of Economics, University of Chile, Ministry of Planning (Chile). Chile National Socioeconomic Characterization Survey 2000. Santiago, Chile: Ministry of Social Development (Chile)
Stroke	Ministry of Public Health (Thailand). Thailand National Health and Examination Survey 2003-2004
Stroke	World Health Organization (WHO). Denmark World Health Survey 2003. Geneva, Switzerland: World Health Organization (WHO), 2005
Stroke	World Health Organization (WHO). Estonia World Health Survey 2003. Geneva, Switzerland: World Health Organization (WHO), 2005
Stroke	World Health Organization (WHO). Guatemala World Health Survey 2003. Geneva, Switzerland: World Health Organization (WHO), 2005
Stroke	World Health Organization (WHO). Hungary World Health Survey 2003. Geneva, Switzerland: World Health Organization (WHO), 2005
Stroke	World Health Organization (WHO). Morocco World Health Survey 2003. Geneva, Switzerland: World Health Organization (WHO), 2005
Stroke	World Health Organization (WHO). Zimbabwe World Health Survey 2003. Geneva, Switzerland: World Health Organization (WHO), 2005
Stroke	Department of Economics, University of Chile, Ministry of Planning (Chile). Chile National Socioeconomic Characterization Survey 2003. Santiago, Chile: Ministry of Social Development (Chile)
Stroke	University of Wisconsin-Madison, Inter-University Consortium for Political and Social Research (ICPSR), Institute of Nutrition and Food Technology (INTA), University of Chile, Center for Geriatrics and Gerontology, Pontifical Catholic University of Chile. Chile - Santiago Survey on Health, Well-Being, and Aging in Latin America and the
Stroke	Directorate of Statistics of the High Commission for Planning (Morocco), World Bank. Morocco Living Standards Measurement Survey 1990-1991
Stroke	Department of Economics, University of Chile, Ministry of Planning (Chile). Chile National Socioeconomic Characterization Survey 2006. Santiago, Chile: Ministry of Social Development (Chile)
Stroke	Jorgensen HS, Plesner AM, Hubbe P, Larsen K. Marked increase of stroke incidence in men between 1972 and 1990 in Frederiksberg, Denmark. Stroke. 1992; 23(12): 1701-4

Stroke	Lavados PM, Sacks C, Prina L, Escobar A, Tossi C, Araya F, Feuerhake W, Galvez M, Salinas R, Alvarez G. Incidence, 30-day case-fatality rate, and prognosis of stroke in Iquique, Chile: a 2-year community-based prospective study (PISCIS project). Lancet. 2005; 365(9478): 2206-15
Stroke	Vibo R, Kõrv J, Roose M. The Third Stroke Registry in Tartu, Estonia: Decline of Stroke Incidence and 28-Day Case-Fatality Rate Since 1991. Stroke. 2005; 36(12): 2544-8
Stroke	Health Promotion Research Institute (Hungary), Hungarian Gallup Institute. Hungary National Population Health Survey 2000
Stroke	Kôrv J, Roose M, Kaasik A-E. Changed Incidence and Case-Fatality Rates of First-Ever Stroke Between 1970 and 1993 in Tartu, Estonia. Stroke. 1996; 27(2): 199-203
Stroke	Matenga J. Stroke incidence rates among black residents of Harare - a prospective community-based study. S Afr Med J. 1997; 87(5): 606-8
Stroke	Fuh JL, Wang SJ, Larson EB, Liu HC. Prevalence of stroke in Kinmen. Stroke. 1996; 27(8): 1338-41
Stroke	Huang Z-S, Chiang T-L, Lee T-K. Stroke Prevalence in Taiwan: Findings From the 1994 National Health Interview Survey. Stroke. 1997; 28(8): 1579-84
Stroke	Ministry of Health (Chile). Chile Hospital Discharges 2001. Santiago, Chile: Ministry of Health (Chile)
Stroke	Ministry of Health (Chile). Chile Hospital Discharges 2002. Santiago, Chile: Ministry of Health (Chile)
Stroke	Ministry of Health (Chile). Chile Hospital Discharges 2003. Santiago, Chile: Ministry of Health (Chile)
Stroke	Ministry of Health (Chile). Chile Hospital Discharges 2004. Santiago, Chile: Ministry of Health (Chile)
Stroke	Ministry of Health (Chile). Chile Hospital Discharges 2005. Santiago, Chile: Ministry of Health (Chile)
Stroke	Ministry of Health (Chile). Chile Hospital Discharges 2006. Santiago, Chile: Ministry of Health (Chile)
Stroke	Ministry of Health (Chile). Chile Hospital Discharges 2007. Santiago, Chile: Ministry of Health (Chile)
Stroke	Ministry of Health (Chile). Chile Hospital Discharges 2008. Santiago, Chile: Ministry of Health (Chile)
Stroke	Ministry of Health (Chile). Chile Hospital Discharges 2009. Santiago, Chile: Ministry of Health (Chile)
Stroke	Ministry of Health (Chile). Chile Hospital Discharges 2010. Santiago, Chile: Ministry of Health (Chile)
Stroke	Ministry of Health (Chile). Chile Hospital Discharges 2011. Santiago, Chile: Ministry of Health (Chile)
Stroke	Ministry of Planning (Chile), Social Observatory, Alberto Hurtado University. Chile National Socioeconomic Characterization Survey 2009. Santiago, Chile: Ministry of Social Development (Chile)
Stroke	Korea Centers for Disease Control and Prevention. South Korea National Health and Nutrition Examination Survey 2012
Stroke	Competence Centre for Clinical Quality and Health Informatics West (Denmark). Danish Stroke Registry Data 2009. [Unpublished]
Stroke	Hu HH, Sheng WY, Chu FL, Lan CF, Chiang BN. Incidence of stroke in Taiwan. Stroke. 1992; 23: 1237–1241
Stroke	Ministry of Health (Chile). Chile Hospital Discharges 2012. Santiago, Chile: Ministry of Health (Chile)
Stroke	University of Concepcion (Chile), World Health Organization (WHO). Chile WHO Multi-country Survey Study on Health and Health System Responsiveness 2000-2001. Geneva, Switzerland: World Health Organization (WHO)
Stroke	Statistics Denmark, World Health Organization (WHO). Denmark WHO Multi-country Survey Study on Health and Health System Responsiveness 2000-2001. Geneva, Switzerland: World Health Organization (WHO)
Stroke	International Research Associates (INRA) Europe, World Health Organization (WHO). Estonia WHO Multi-country Survey Study on Health and Health System Responsiveness 2000-2001. Geneva, Switzerland: World Health Organization (WHO)
Stroke	Szonda Ipsos, World Health Organization (WHO). Hungary WHO Multi-country Survey Study on Health and Health System Responsiveness 2000-2001. Geneva, Switzerland: World Health Organization (WHO)
Stroke	Graduate School of Public Health, Seoul National University, World Health Organization (WHO). South Korea WHO Multi-country Survey Study on Health and Health System Responsiveness 2000-2001. Geneva, Switzerland: World Health Organization (WHO)
Stroke	Gallup Europe, World Health Organization (WHO). Morocco WHO Multi-country Survey Study on Health and Health System Responsiveness 2000-2001. Geneva, Switzerland: World Health Organization (WHO)
Stroke	International Research Associates (INRA) Europe, World Health Organization (WHO). Malta WHO Multi-country Survey Study on Health and Health System Responsiveness 2000-2001. Geneva, Switzerland: World Health Organization (WHO)
Stroke	Mahidol University, World Health Organization (WHO). Thailand WHO Multi-country Survey Study on Health and Health System Responsiveness 2000-2001. Geneva, Switzerland: World Health Organization (WHO)
Stroke	Börsch-Supan, A. (2015). Survey of Health, Ageing and Retirement in Europe (SHARE) Wave 5. Release version: 1.0.0. SHARE-ERIC. Data set. DOI: 10.6103/SHARE.w5.100
Stroke	Börsch-Supan, A. (2015). Survey of Health, Ageing and Retirement in Europe (SHARE) Wave 5. Release version: 1.0.0. SHARE-ERIC. Data set. DOI: 10.6103/SHARE.w5.100
Stroke	National Office of Statistics (Cuba). Cuba Statistical Yearbook 2012. Havana, Cuba: National Office of Statistics (Cuba)
Stroke	Truelsen T, Gronbaek M, Schnohr P, Boysen G. Stroke case fatality in Denmark from 1977 to 1992: the Copenhagen City Heart Study. Neuroepidemiology. 2002; 21(1): 22–7
Stroke	Danish Health and Medicines Authority. Denmark National Patient Registry 2003
Stroke	Danish Health and Medicines Authority. Denmark National Patient Registry 2004
Stroke	Danish Health and Medicines Authority. Denmark National Patient Registry 2005
Stroke	Danish Health and Medicines Authority. Denmark National Patient Registry 2006
Stroke	Danish Health and Medicines Authority. Denmark National Patient Registry 2007
Stroke	Danish Health and Medicines Authority. Denmark National Patient Registry 2008
Stroke	Danish Health and Medicines Authority. Denmark National Patient Registry 2009
Stroke	Ministry of Social Affairs (Estonia), National Institute for Health Development (Estonia). Estonia Hospital Inpatient Discharges 2003
Stroke	Ministry of Social Affairs (Estonia), National Institute for Health Development (Estonia). Estonia Hospital Inpatient Discharges 2004
Stroke	Ministry of Social Affairs (Estonia), National Institute for Health Development (Estonia). Estonia Hospital Inpatient Discharges 2005
Stroke	Ministry of Social Affairs (Estonia), National Institute for Health Development (Estonia). Estonia Hospital Inpatient Discharges 2006
Stroke	Ministry of Social Affairs (Estonia), National Institute for Health Development (Estonia). Estonia Hospital Inpatient Discharges 2007
Stroke	Ministry of Social Affairs (Estonia), National Institute for Health Development (Estonia). Estonia Hospital Inpatient Discharges 2008
Stroke	Ministry of Social Affairs (Estonia), National Institute for Health Development (Estonia). Estonia Hospital Inpatient Discharges 2009
Stroke	Ministry of Social Affairs (Estonia), National Institute for Health Development (Estonia). Estonia Hospital Inpatient Discharges 2010
Stroke	Ministry of Social Affairs (Estonia), National Institute for Health Development (Estonia). Estonia Hospital Inpatient Discharges 2011
Stroke	Center for Health Care Information (GYOGYINFOK) (Hungary). Hungary Hospital Inpatient Discharges 1990
Stroke	National Institute for Health Development (Estonia). Estonia Hospital Inpatient Discharges 2012
Stroke	National Institute for Health Development (Estonia). Estonia Hospital Inpatient Discharges 2013
Stroke	National Institute for Health Development (Estonia). Estonia Hospital Inpatient Discharges 2014
Stroke	Institute of Experimental and Clinical Medicine (Estonia). Estonia Hospital Inpatient Discharges 1999
Stroke	Institute of Experimental and Clinical Medicine (Estonia). Estonia Hospital Inpatient Discharges 1998
Stroke	Institute of Experimental and Clinical Medicine (Estonia). Estonia Hospital Inpatient Discharges 1997
Stroke	Institute of Experimental and Clinical Medicine (Estonia). Estonia Hospital Inpatient Discharges 1996
Stroke	Institute of Experimental and Clinical Medicine (Estonia). Estonia Hospital Inpatient Discharges 1995

Stroke	Institute of Experimental and Clinical Medicine (Estonia). Estonia Hospital Inpatient Discharges 1994
Stroke	Institute of Experimental and Clinical Medicine (Estonia). Estonia Hospital Inpatient Discharges 1993
Stroke	Institute of Experimental and Clinical Medicine (Estonia). Estonia Hospital Inpatient Discharges 1992
Stroke	Institute of Experimental and Clinical Medicine (Estonia). Estonia Hospital Inpatient Discharges 1991
Stroke	Institute of Experimental and Clinical Medicine (Estonia). Estonia Hospital Inpatient Discharges 1990
Stroke	Institute of Experimental and Clinical Medicine (Estonia). Estonia Hospital Inpatient Discharges 1989
Stroke	Institute of Experimental and Clinical Medicine (Estonia). Estonia Hospital Inpatient Discharges 1988
Stroke	Institute of Experimental and Clinical Medicine (Estonia). Estonia Hospital Inpatient Discharges 1987
Stroke	Institute of Experimental and Clinical Medicine (Estonia). Estonia Hospital Inpatient Discharges 1986
Stroke	Institute of Experimental and Clinical Medicine (Estonia). Estonia Hospital Inpatient Discharges 1985
Stroke	Institute of Experimental and Clinical Medicine (Estonia). Estonia Hospital Inpatient Discharges 1980
Stroke	Center for Health Care Information (GYOGYINFOK) (Hungary). Hungary Hospital Inpatient Discharges 1991
Stroke	Center for Health Care Information (GYOGYINFOK) (Hungary). Hungary Hospital Inpatient Discharges 1992
Stroke	Center for Health Care Information (GYOGYINFOK) (Hungary). Hungary Hospital Inpatient Discharges 1993
Stroke	Center for Health Care Information (GYOGYINFOK) (Hungary). Hungary Hospital Inpatient Discharges 1994
Stroke	Center for Health Care Information (GYOGYINFOK) (Hungary). Hungary Hospital Inpatient Discharges 1995
Stroke	Center for Health Care Information (GYOGYINFOK) (Hungary). Hungary Hospital Inpatient Discharges 1996
Stroke	Center for Health Care Information (GYOGYINFOK) (Hungary). Hungary Hospital Inpatient Discharges 1997
Stroke	Center for Health Care Information (GYOGYINFOK) (Hungary). Hungary Hospital Inpatient Discharges 1998
Stroke	Center for Health Care Information (GYOGYINFOK) (Hungary). Hungary Hospital Inpatient Discharges 1999
Stroke	Center for Health Care Information (GYOGYINFOK) (Hungary). Hungary Hospital Inpatient Discharges 2000
Stroke	Center for Health Care Information (GYOGYINFOK) (Hungary). Hungary Hospital Inpatient Discharges 2001
Stroke	Center for Health Care Information (GYOGYINFOK) (Hungary). Hungary Hospital Inpatient Discharges 2002
Stroke	Center for Health Care Information (GYOGYINFOK) (Hungary). Hungary Hospital Inpatient Discharges 2003
Stroke	National Institute for Strategic Health Research (ESKI) (Hungary). Hungary Hospital Inpatient Discharges 2013
Stroke	National Institute for Strategic Health Research (ESKI) (Hungary). Hungary Hospital Inpatient Discharges 2014
Stroke	Danish Health and Medicines Authority. Denmark National Patient Registry 1979
Stroke	Danish Health and Medicines Authority. Denmark National Patient Registry 1980
Stroke	Danish Health and Medicines Authority. Denmark National Patient Registry 1981
Stroke	Danish Health and Medicines Authority. Denmark National Patient Registry 1982
Stroke	Danish Health and Medicines Authority. Denmark National Patient Registry 1983
Stroke	Danish Health and Medicines Authority. Denmark National Patient Registry 1984
Stroke	Danish Health and Medicines Authority. Denmark National Patient Registry 1985
Stroke	Danish Health and Medicines Authority. Denmark National Patient Registry 1986
Stroke	Danish Health and Medicines Authority. Denmark National Patient Registry 1987
Stroke	Danish Health and Medicines Authority. Denmark National Patient Registry 1988
Stroke	Danish Health and Medicines Authority. Denmark National Patient Registry 1989
Stroke	Danish Health and Medicines Authority. Denmark National Patient Registry 1990
Stroke	Danish Health and Medicines Authority. Denmark National Patient Registry 1991
Stroke	Danish Health and Medicines Authority. Denmark National Patient Registry 1992
Stroke	Danish Health and Medicines Authority. Denmark National Patient Registry 1993
Stroke	Danish Health and Medicines Authority. Denmark National Patient Registry 1994
Stroke	Danish Health and Medicines Authority. Denmark National Patient Registry 1995
Stroke	Danish Health and Medicines Authority. Denmark National Patient Registry 1996
Stroke	Danish Health and Medicines Authority. Denmark National Patient Registry 1997
Stroke	Danish Health and Medicines Authority. Denmark National Patient Registry 1998
Stroke	Danish Health and Medicines Authority. Denmark National Patient Registry 1999
Stroke	Danish Health and Medicines Authority. Denmark National Patient Registry 2000
Stroke	Danish Health and Medicines Authority. Denmark National Patient Registry 2001
Stroke	Danish Health and Medicines Authority. Denmark National Patient Registry 2002
Stroke	Danish Health and Medicines Authority. Denmark National Patient Registry 2010
Stroke	Danish Health and Medicines Authority. Denmark National Patient Registry 2011
Stroke	Danish Health and Medicines Authority. Denmark National Patient Registry 2012
Stroke	Danish Health and Medicines Authority. Denmark National Patient Registry 2013
Stroke	National State Statistical Agency (Tajikistan), World Bank. Tajikistan Living Standards Measurement Survey 2003
Stroke	National State Statistical Agency (Tajikistan), World Bank. Tajikistan Living Standards Measurement Survey 2007
Stroke	Planning Commission (Tanzania), University of Dar es Salaam, World Bank. Tanzania Living Standards Measurement Study 1993-1994. Washington DC, United States: World Bank
Stroke	National Statistics Directorate (Timor-Leste), World Bank. Timor-Leste Living Standards and Measurement Survey 2001. Washington DC, United States: World Bank
Stroke	Palestinian Central Bureau of Statistics. Palestine Demographic and Health Survey 2004
Stroke	World Health Organization (WHO). Ethiopia World Health Survey 2003. Geneva, Switzerland: World Health Organization (WHO), 2005
Stroke	World Health Organization (WHO). Finland World Health Survey 2004. Geneva, Switzerland: World Health Organization (WHO), 2005
Stroke	World Health Organization (WHO). Latvia World Health Survey 2003. Geneva, Switzerland: World Health Organization (WHO), 2005
Stroke	World Health Organization (WHO). Netherlands World Health Survey 2004. Geneva, Switzerland: World Health Organization (WHO), 2005
Stroke	World Health Organization (WHO). Uruguay World Health Survey 2002-2003. Geneva, Switzerland: World Health Organization (WHO), 2005
Stroke	National State Statistical Agency (Tajikistan), World Bank. Tajikistan Living Standards Measurement Survey 2009
Stroke	National Statistics Directorate (Timor-Leste), World Bank. Timor-Leste Living Standards and Measurement Survey 2007-2008. Washington DC, United States: World Bank
Stroke	National Bureau of Statistics (Tanzania). Tanzania Living Standards Measurement Study - Integrated Surveys on Agriculture 2010-2011. Dar es Salaam, Tanzania: National Bureau of Statistics (Tanzania)
Stroke	Numminen H, Kotila M, Waltimo O, Aho K, Kaste M. Declining Incidence and Mortality Rates of Stroke in Finland From 1972 to 1991: Results of Three Population-Based Stroke Registers. Stroke. 1996; 27(9): 1487-91
Stroke	Immonen-Räihä P, Mähönen M, Tuomilehto J, Salomaa V, Kaarsalo E, Narva EV, Salmi K, Sarti C, Sivenius J, Alhainen K, Torppa J. Trends in Case-Fatality of Stroke in Finland During 1983 to 1992. Stroke. 1997; 28(12): 2493-9

Stroke	Vaartjes I, Reitsma JB, de Bruin A, Berger-van Sijl M, Bos MJ, Breteler MM, Grobbee DE, Bots ML. Nationwide incidence of first stroke and TIA in the Netherlands. Eur J Neurol. 2008; 15(12): 1315-23
Stroke	Walker R, Unwin N, Mugusi F, Swai M, Aris E, Jusabani A, Kabadi G, Gray W, Lewanga M, Alberti G, Whiting D. Stroke incidence in rural and urban Tanzania: a prospective, community-based study. Lancet Neurol. 2010; 9(8): 786-92
Stroke	Sweileh WM, Sawalha AF, Al-Aqad SM, Zyoud SH, Al-Jabi SW. The Epidemiology of Stroke in Northern Palestine: A 1-Year, Hospital-Based Study. J Stroke Cerebrovasc Dis. 2008; 17(6): 406-11
Stroke	De Jesús Llibre J, Valhuerdi A, Fernández O, Llibre JC, Porto R, López AM, Marcheco B, Moreno C. Prevalence of stroke and associated risk factors in older adults in Havana City and Matanzas Provinces, Cuba (10/66 population-based study). MEDICC Rev. 2010; 12(3): 20-6
Stroke	Tekle-Haimanot R, Abebe M, Gebre-Mariam A, Forsgren L, Heijbel J, Holmgren G, Ekstedt J. Community-based study of neurological disorders in rural central Ethiopia. Neuroepidemiology. 1990; 9(5): 263-77
Stroke	Venketasubramanian N, Tan LCS, Sahadevan S, Chin JJ, Krishnamoorthy ES, Hong CY, Saw SM. Prevalence of Stroke Among Chinese, Malay, and Indian Singaporeans. Stroke. 2005; 36(3): 551-6
Stroke	Walker R, McLarty D, Masuki G, Kitange H, Whiting D, Moshi A, Massawe J, Amaro R, Mhina A, Alberti K. Age specific prevalence of impairment and disability relating to hemiplegic stroke in the Hai District of northern Tanzania. J Neurol Neurosurg Psychiatr. 2000; 68(6): 744-9
Stroke	Wieberdink RG, Ikram MA, Hofman A, Koudstaal PJ, Breteler MMB. Trends in stroke incidence rates and stroke risk factors in Rotterdam, the Netherlands from 1990 to 2008. Eur J Epidemiol. 2012; 27(4): 287-95
Stroke	Dutch Hospital Data (DHD). Netherlands National Medical Registry 1998
Stroke	Dutch Hospital Data (DHD). Netherlands National Medical Registry 1999
Stroke	Dutch Hospital Data (DHD). Netherlands National Medical Registry 2000
Stroke	Dutch Hospital Data (DHD). Netherlands National Medical Registry 2001
Stroke	Dutch Hospital Data (DHD). Netherlands National Medical Registry 2002
Stroke	Dutch Hospital Data (DHD). Netherlands National Medical Registry 2003
Stroke	Dutch Hospital Data (DHD). Netherlands National Medical Registry 2004
Stroke	Dutch Hospital Data (DHD). Netherlands National Medical Registry 2006
Stroke	Dutch Hospital Data (DHD). Netherlands National Medical Registry 2007
Stroke	Dutch Hospital Data (DHD). Netherlands National Medical Registry 2010
Stroke	Development Center for Welfare and Health (STAKES) (Finland), World Health Organization (WHO). Finland WHO Multi-country Survey Study on Health and Health System Responsiveness 2000-2001. Geneva, Switzerland: World Health Organization (WHO)
Stroke	Gallup Europe, World Health Organization (WHO). Latvia WHO Multi-country Survey Study on Health and Health System Responsiveness 2000-2001. Geneva, Switzerland: World Health Organization (WHO)
Stroke	International Research Associates (INRA) Europe, Netherlands Organisation for Applied Scientific Research (TNO), World Health Organization (WHO). Netherlands WHO Multi-country Survey Study on Health and Health System Responsiveness 2000-2001. Geneva, Switzerland: World Health Organization (WHO)
Stroke	University of the West Indies, World Health Organization (WHO). Trinidad and Tobago WHO Multi-country Survey Study on Health and Health System Responsiveness 2000-2001. Geneva, Switzerland: World Health Organization (WHO)
Stroke	Börsch-Supan, A. (2015). Survey of Health, Ageing and Retirement in Europe (SHARE) Wave 5. Release version: 1.0.0. SHARE-ERIC. Data set. DOI: 10.6103/SHARE.w5.100
Stroke	Centre for Disease Prevention and Control (Latvia), Riga Stradi?š University. Latvia Health Behavior Among the Adult Population 2014
Stroke	Rastas S, Verkkoniemi A, Polvikoski T, Juva K, Niinisto L, Mattila K, Lansimies E, Pirttila T, Sulkava R. Atrial fibrillation, stroke, and cognition: a longitudinal population-based study of people aged 85 and older. Stroke. 2007; 38(5): 1454–60
Stroke	Ministry of Health of the Republic of Latvia. Latvia Hospital Inpatient Discharges 2004
Stroke	Ministry of Health of the Republic of Latvia. Latvia Hospital Inpatient Discharges 2006
Stroke	Ministry of Health of the Republic of Latvia. Latvia Hospital Inpatient Discharges 2007
Stroke	Ministry of Health of the Republic of Latvia. Latvia Hospital Inpatient Discharges 2008
Stroke	Ministry of Health of the Republic of Latvia. Latvia Hospital Inpatient Discharges 2010
Stroke	Ministry of Health of the Republic of Latvia, National Health Service (Latvia). Latvia Hospital Inpatient Discharges 2011
Stroke	Ministry of Health of the Republic of Latvia, National Health Service (Latvia). Latvia Hospital Inpatient Discharges 2012
Stroke	Institute of Public Health (Macedonia). Macedonia Hospital Inpatient Discharges 2000
Stroke	Institute of Public Health (Macedonia). Macedonia Hospital Inpatient Discharges 2001
Stroke	Institute of Public Health (Macedonia). Macedonia Hospital Inpatient Discharges 2002
Stroke	Institute of Public Health (Macedonia). Macedonia Hospital Inpatient Discharges 2004
Stroke	National Public Health Institute (Finland). Finland Hospital Discharge Register 1988
Stroke	National Public Health Institute (Finland). Finland Hospital Discharge Register 1989
Stroke	National Public Health Institute (Finland). Finland Hospital Discharge Register 1990
Stroke	National Public Health Institute (Finland). Finland Hospital Discharge Register 1991
Stroke	National Public Health Institute (Finland). Finland Hospital Discharge Register 1992
Stroke	National Public Health Institute (Finland). Finland Hospital Discharge Register 1993
Stroke	Ministry of Health of the Republic of Latvia. Latvia Hospital Inpatient Discharges 1980
Stroke	Institute of Public Health (Macedonia). Macedonia Hospital Inpatient Discharges 1980
Stroke	Dutch Hospital Data (DHD). Netherlands National Medical Registry 1990
Stroke	Dutch Hospital Data (DHD). Netherlands National Medical Registry 1991
Stroke	Dutch Hospital Data (DHD). Netherlands National Medical Registry 1992
Stroke	Dutch Hospital Data (DHD). Netherlands National Medical Registry 1993
Stroke	Dutch Hospital Data (DHD). Netherlands National Medical Registry 1994
Stroke	Dutch Hospital Data (DHD). Netherlands National Medical Registry 1995
Stroke	Dutch Hospital Data (DHD). Netherlands National Medical Registry 1996
Stroke	Dutch Hospital Data (DHD). Netherlands National Medical Registry 1997
Stroke	National Public Health Institute (Finland). Finland Hospital Discharge Register 1994
Stroke	National Public Health Institute (Finland). Finland Hospital Discharge Register 1995
Stroke	National Public Health Institute (Finland). Finland Hospital Discharge Register 1996
Stroke	National Public Health Institute (Finland). Finland Hospital Discharge Register 1997
Stroke	National Public Health Institute (Finland). Finland Hospital Discharge Register 1998
Stroke	National Public Health Institute (Finland). Finland Hospital Discharge Register 1999
Stroke	National Institute for Health and Welfare (THL) (Finland). Finland Hospital Discharge Register 2013
Stroke	National Institute for Health and Welfare (THL) (Finland). Finland Hospital Discharge Register 2014

Stroke	Ministry of Health of the Republic of Latvia. Latvia Hospital Inpatient Discharges 1981
Stroke	Ministry of Health of the Republic of Latvia. Latvia Hospital Inpatient Discharges 1982
Stroke	Ministry of Health of the Republic of Latvia. Latvia Hospital Inpatient Discharges 1983
Stroke	Ministry of Health of the Republic of Latvia. Latvia Hospital Inpatient Discharges 1984
Stroke	Ministry of Health of the Republic of Latvia. Latvia Hospital Inpatient Discharges 1985
Stroke	Ministry of Health of the Republic of Latvia. Latvia Hospital Inpatient Discharges 1986
Stroke	Ministry of Health of the Republic of Latvia. Latvia Hospital Inpatient Discharges 1987
Stroke	Ministry of Health of the Republic of Latvia. Latvia Hospital Inpatient Discharges 1988
Stroke	Ministry of Health of the Republic of Latvia. Latvia Hospital Inpatient Discharges 1989
Stroke	Ministry of Health of the Republic of Latvia. Latvia Hospital Inpatient Discharges 1990
Stroke	Ministry of Health of the Republic of Latvia. Latvia Hospital Inpatient Discharges 1991
Stroke	Ministry of Health of the Republic of Latvia. Latvia Hospital Inpatient Discharges 1992
Stroke	Ministry of Health of the Republic of Latvia. Latvia Hospital Inpatient Discharges 1993
Stroke	Ministry of Health of the Republic of Latvia. Latvia Hospital Inpatient Discharges 1994
Stroke	Ministry of Health of the Republic of Latvia. Latvia Hospital Inpatient Discharges 1995
Stroke	Ministry of Health of the Republic of Latvia. Latvia Hospital Inpatient Discharges 1996
Stroke	Ministry of Health of the Republic of Latvia. Latvia Hospital Inpatient Discharges 1997
Stroke	Ministry of Health of the Republic of Latvia. Latvia Hospital Inpatient Discharges 1998
Stroke	Ministry of Health of the Republic of Latvia. Latvia Hospital Inpatient Discharges 1999
Stroke	Ministry of Health of the Republic of Latvia. Latvia Hospital Inpatient Discharges 2000
Stroke	Ministry of Health of the Republic of Latvia. Latvia Hospital Inpatient Discharges 2001
Stroke	Ministry of Health of the Republic of Latvia. Latvia Hospital Inpatient Discharges 2002
Stroke	Ministry of Health of the Republic of Latvia. Latvia Hospital Inpatient Discharges 2003
Stroke	Ministry of Health of the Republic of Latvia, National Health Service (Latvia). Latvia Hospital Inpatient Discharges 2013
Stroke	Ministry of Health of the Republic of Latvia, National Health Service (Latvia). Latvia Hospital Inpatient Discharges 2014
Stroke	Institute of Public Health (Macedonia). Macedonia Hospital Inpatient Discharges 1982
Stroke	Institute of Public Health (Macedonia). Macedonia Hospital Inpatient Discharges 1984
Stroke	Institute of Public Health (Macedonia). Macedonia Hospital Inpatient Discharges 1985
Stroke	Institute of Public Health (Macedonia). Macedonia Hospital Inpatient Discharges 1986
Stroke	Institute of Public Health (Macedonia). Macedonia Hospital Inpatient Discharges 1987
Stroke	Institute of Public Health (Macedonia). Macedonia Hospital Inpatient Discharges 1989
Stroke	Institute of Public Health (Macedonia). Macedonia Hospital Inpatient Discharges 1990
Stroke	Institute of Public Health (Macedonia). Macedonia Hospital Inpatient Discharges 1991
Stroke	Institute of Public Health (Macedonia). Macedonia Hospital Inpatient Discharges 1992
Stroke	Institute of Public Health (Macedonia). Macedonia Hospital Inpatient Discharges 1993
Stroke	Institute of Public Health (Macedonia). Macedonia Hospital Inpatient Discharges 1995
Stroke	Institute of Public Health (Macedonia). Macedonia Hospital Inpatient Discharges 1996
Stroke	Institute of Public Health (Macedonia). Macedonia Hospital Inpatient Discharges 1997
Stroke	Institute of Public Health (Macedonia). Macedonia Hospital Inpatient Discharges 2012
Stroke	Béjot Y, Cordonnier C, Durier J, Aboa-Eboulé C, Rouaud O, Giroud M. Intracerebral haemorrhage profiles are changing: results from the Dijon population-based study. Brain. 2013; 136(Pt 2): 658-64
Stroke	Tveiten A, Ljøstad U, Mygland A, Thomassen L, Pripp AH, Naess H. Intracerebral hemorrhage in southern Norway – a hospital-based incidence study. Eur Neurol. 2012; 67(4): 240-5
Stroke	Awada A, Russell N, Al Rajeh S, Omojola M. Non-traumatic cerebral hemorrage in Saudi Arabs: a hospital-based study of 243 cases. J Neurol Sci. 1996; 144(1-2): 198-203
Stroke	Irwin J, Wright P, Reeve P. Temporal trends and clinical characteristics of spontaneous intracerebral haemorrhage in the Waikato region of New Zealand: a hospital-based analysis. N Z Med J. 2011; 124(1345): 16-25
Stroke	Howard G, Cushman M, Howard VJ, Kissela BM, Kleindorfer DO, Moy CS, Switzer J, Woo D. Risk factors for intracerebral hemorrhage: the REasons for geographic and racial differences in stroke (REGARDS) study. Stroke. 2013; 44(5): 1282–7
Stroke	Sacco S, Marini C, Toni D, Olivieri L, Carolei A. Incidence and 10-Year Survival of Intracerebral Hemorrhage in a Population-Based Registry. Stroke. 2009; 40(2): 394-9
Ischemic Stroke	World Health Organization Regional Office for Europe (WHO/Europe). European Hospital Morbidity Database 1999-2007. Copenhagen, Denmark: World Health Organization Regional Office for Europe (WHO/Europe)
Ischemic Stroke	Center for Research and Teaching in Economics (CIDE) (Mexico), National Institute of Perinatology (Mexico), National Institute of Statistics and Geography (INEGI) (Mexico), Universidad Iberoamericana. Mexico Family Life Survey 2002
Ischemic Stroke	National Institute of Public Health (Mexico). Mexico National Health Survey 1999-2000
Ischemic Stroke	National Institute of Public Health (Mexico). Mexico National Survey of Health and Nutrition 2005-2006. Cuernavaca, Mexico: National Institute of Public Health (Mexico)
Ischemic Stroke	World Health Organization (WHO). Congo World Health Survey 2003. Geneva, Switzerland: World Health Organization (WHO), 2005
Ischemic Stroke	World Health Organization (WHO). France World Health Survey 2003. Geneva, Switzerland: World Health Organization (WHO), 2005
Ischemic Stroke	World Health Organization (WHO). Kenya World Health Survey 2004. Geneva, Switzerland: World Health Organization (WHO), 2005
Ischemic Stroke	World Health Organization (WHO). Mexico World Health Survey 2002-2003. Geneva, Switzerland: World Health Organization (WHO), 2005
Ischemic Stroke	World Health Organization (WHO). Norway World Health Survey 2003. Geneva, Switzerland: World Health Organization (WHO), 2005
Ischemic Stroke	World Health Organization (WHO). Vietnam World Health Survey 2002-2003. Geneva, Switzerland: World Health Organization (WHO), 2005
Ischemic Stroke	General Statistics Office (Viet Nam), United Nations Development Programme (UNDP), World Bank (WB). Viet Nam Living Standards Measurement Survey 2008. Ha N?i, Viet Nam: General Statistics Office (Viet Nam)
Ischemic Stroke	General Statistics Office (Viet Nam), United Nations Development Programme (UNDP), World Bank. Vietnam Living Standards Measurement Survey 2006
Ischemic Stroke	National Institute of Statistics (Cambodia), Statistics Sweden. Cambodia Socio-Economic Survey 2003-2005. Phnom Penh, Cambodia: National Institute of Statistics (Cambodia)
Ischemic Stroke	National Institute of Statistics (Cambodia), Statistics Sweden. Cambodia Socio-Economic Survey 2006-2007. Phnom Penh, Cambodia: National Institute of Statistics (Cambodia)
Ischemic Stroke	University of Wisconsin-Madison, Inter-University Consortium for Political and Social Research (ICPSR), College of the Northern Border (COLEF), Research in Health and Demographics (INSAD), National Institute of Medical Sciences and Nutrition Salvador Zubirán. Mexico - Mexico City Survey on Health, Well-Being, and Aging in Latin

Ischemic Stroke	Management Research (IIHMR), Ministry of Public Health (Afghanistan), World Health Organization Regional Office for the Eastern Mediterranean (EMRO-WHO). Afghanistan Special Demographic and Health Survey 2010. Fairfax, United States: ICF International
Ischemic Stroke	California Center for Population Research (CCPR), University of California Los Angeles (UCLA), Center for Research and Teaching in Economics (CIDE) (Mexico), National Institute of Public Health (Mexico), Universidad Iberoamericana. Mexico Family Life Survey 2005-2006
Ischemic Stroke	Organization for Economic Co-operation and Development (OECD). OECD Health Statistics. Paris, France: Organization for Economic Co-operation and Development (OECD)
Ischemic Stroke	National Institute of Public Health (Mexico). Mexico National Survey of Health and Nutrition 2011-2012. Cuernavaca, Mexico: National Institute of Public Health (Mexico)
Ischemic Stroke	Ellekjær H, Holmen J, Indredavik B, Terent A. Epidemiology of Stroke in Innherred, Norway, 1994 to 1996: Incidence and 30-Day Case-Fatality Rate. Stroke. 1997; 28(11): 2180-4
Ischemic Stroke	Bejot Y, Rouaud O, Durier J, Caillier M, Marie C, Freysz M, Yeguiayan J-M, Chantegret A, Osseby G, Moreau T, Giroud M. Decrease in the Stroke Case Fatality Rates in a French Population-Based Twenty-Year Study. Cerebrovasc Dis. 2007; 24(5): 439-44
Ischemic Stroke	Giroud M, Lemesle M, Gouyon JB, Nivelon JL, Milan C, Dumas R. Cerebrovascular disease in children under 16 years of age in the city of Dijon, France: a study of incidence and clinical features from 1985 to 1993. J Clin Epidemiol. 1995; 48(11): 1343-8
Ischemic Stroke	Smadja D, Cabre P, May F, Fanon J-L, René-Corail P, Riocreux C, Charpentier J-C, Fournerie P, Saint-Vil M, Ketterlé J. ERMANCIA: Epidemiology of Stroke in Martinique, French West Indies. Stroke. 2001; 32(12): 2741-7
Ischemic Stroke	Wolfe CDA, Giroud M, Kolominsky-Rabas P, Dundas R, Lemesle M, Heuschmann P, Rudd A. Variations in Stroke Incidence and Survival in 3 Areas of Europe. Stroke. 2000; 31(9): 2074-9
Ischemic Stroke	National Institute of Statistics, Geography, and Informatics (Mexico), Population Studies Center, University of Pennsylvania, University of Maryland, University of Wisconsin. Mexico Health and Aging Study 2001
Ischemic Stroke	National Institute of Statistics, Geography, and Informatics (Mexico), Population Studies Center, University of Pennsylvania, University of Maryland, University of Wisconsin. Mexico Health and Aging Study 2003
Ischemic Stroke	National Institute of Statistics, Geography, and Informatics (Mexico), Population Studies Center, University of Pennsylvania, University of Maryland, University of Wisconsin. Mexico Health and Aging Study 2012. Mexico City, México: National Institute of Statistics, Geography, and Informatics (Mexico)
Ischemic Stroke	National Institute of Public Health (Mexico), World Health Organization (WHO). Mexico WHO Study on Global AGEing and Adult Health 2009-2010. Geneva, Switzerland: World Health Organization (WHO), 2011
Ischemic Stroke	Béjot Y, Benzenine E, Lorgis L, Zeller M, Aubé H, Giroud M, Cottin Y, Quantin C. Comparative analysis of patients with acute coronary and cerebrovascular syndromes from the national French hospitalization health care system database. Neuroepidemiology. 2011; 37(3-4): 143-52
Ischemic Stroke	Cantu-Brito C, Majersik JJ, Sánchez BN, Ruano A, Becerra-Mendoza D, Wing JJ, Morgenstern LB. Door-to-Door Capture of Incident and Prevalent Stroke Cases in Durango, Mexico The Brain Attack Surveillance in Durango Study. Stroke. 2011; 42(3): 601-6
Ischemic Stroke	Cossi M-J, Gobron C, Preux P-M, Niama D, Chabriat H, Houinato D. Stroke: prevalence and disability in Cotonou, Benin. Cerebrovasc Dis. 2012; 33(2): 166-72
Ischemic Stroke	Mohammad QD, Habib M, Hoque A, Alam B, Haque B, Hossain S, Rahman KM, Khan SU. Prevalence of stroke above forty years. Mymensingh Med J. 2011; 20(4): 640-4
Ischemic Stroke	Börsch-Supan, A. (2013). Survey of Health, Ageing and Retirement in Europe (SHARE) Wave 1. Release version: 2.6.0. SHARE-ERIC. Data set. DOI: 10.6103/SHARE.w1.260
Ischemic Stroke	Börsch-Supan, A. (2013). Survey of Health, Ageing and Retirement in Europe (SHARE) Wave 2. Release version: 2.6.0. SHARE-ERIC. Data set. DOI: 10.6103/SHARE.w2.260
Ischemic Stroke	Börsch-Supan, A. (2013). Survey of Health, Ageing and Retirement in Europe (SHARE) Wave 4. Release version: 1.1.1. SHARE-ERIC. Data set. DOI: 10.6103/SHARE.w4.111
Ischemic Stroke	(KEMRI), Kenya National Bureau of Statistics, Ministry of Public Health and Sanitation (Kenya), National AIDS Control Council (Kenya), National AIDS and STI Control Program (Kenya), National Coordinating Agency for Population and Development (Kenya), National Public Health Laboratory Services, Ministry of Public Health and
Ischemic Stroke	Norwegian Directorate of Health. Norway Patient Register 2009
Ischemic Stroke	Norwegian Directorate of Health. Norway Patient Register 2010
Ischemic Stroke	Norwegian Directorate of Health. Norway Patient Register 2011
Ischemic Stroke	Norwegian Directorate of Health. Norway Patient Register 2012
Ischemic Stroke	National Institute of Public Health (Mexico), World Health Organization (WHO). Mexico WHO Multi-country Survey Study on Health and Health System Responsiveness 2000-2001. Geneva, Switzerland: World Health Organization (WHO)
Ischemic Stroke	Abt Associates Inc., Kenya National Bureau of Statistics, Ministry of Health (Kenya). Kenya Household Health Expenditure and Utilization Survey 2007. Nairobi, Kenya: Kenya National Bureau of Statistics
Ischemic Stroke	Center for Research and Teaching in Economics (CIDE) (Mexico), Duke University, National Institute of Public Health (Mexico), Universidad Iberoamericana, University of California, Los Angeles (UCLA). Mexico Family Life Survey 2008-2013
Ischemic Stroke	Action Africa Help International (AAH-I), Institute for Health Metrics and Evaluation (IHME), Ministry of Medical Services (Kenya), Ministry of Public Health and Sanitation (Kenya). Access, Bottlenecks, Costs, and Equity (ABCE) project in Kenya, 2012. Seattle, United States: Institute for Health Metrics and Evaluation (IHME), 2015
Ischemic Stroke	Infectious Diseases Research Collaboration (IDRC), Institute for Health Metrics and Evaluation (IHME), Makerere University, Ministry of Health (Uganda). Access, Bottlenecks, Costs, and Equity (ABCE) project in Uganda, 2012. Seattle, United States: Institute for Health Metrics and Evaluation (IHME), 2015
Ischemic Stroke	National Institute of Statistics (Cambodia), Statistics Sweden. Cambodia Socio-Economic Survey 2007-2008. Phnom Penh, Cambodia: National Institute of Statistics (Cambodia)
Ischemic Stroke	Gallup Europe, World Health Organization (WHO). Bahrain WHO Multi-country Survey Study on Health and Health System Responsiveness 2000-2001. Geneva, Switzerland: World Health Organization (WHO)
Ischemic Stroke	Erik Consulting, International Research Associates (INRA) Europe, World Health Organization (WHO). France WHO Multi-country Survey Study on Health and Health System Responsiveness 2000-2001. Geneva, Switzerland: World Health Organization (WHO)
Ischemic Stroke	Biomedical Engineering Institute, Kaunas University of Technology, Statistics Lithuania, World Health Organization (WHO). Lithuania WHO Multi-country Survey Study on Health and Health System Responsiveness 2000-2001. Geneva, Switzerland: World Health Organization (WHO)
Ischemic Stroke	Gallup Europe, World Health Organization (WHO). Oman WHO Multi-country Survey Study on Health and Health System Responsiveness 2000-2001. Geneva, Switzerland: World Health Organization (WHO)
Ischemic Stroke	Börsch-Supan, A. (2015). Survey of Health, Ageing and Retirement in Europe (SHARE) Wave 5. Release version: 1.0.0. SHARE-ERIC. Data set. DOI: 10.6103/SHARE.w5.100
Ischemic Stroke	Norwegian Directorate of Health. Norway Patient Register 2008-2012
Ischemic Stroke	Arauz A, Villarreal-Careaga J, Rangel-Guerra R, Ramos-Moreno A, Barinagarrementeria F, PREMIER Investigators. Acute care and one-year outcome of Mexican patients with first-ever acute ischemic stroke: the PREMIER study. Rev Neurol. 2010; 51(11): 641–9

Ischemic Stroke	Norwegian Directorate of Health. Norway Patient Register 2002
Ischemic Stroke	Norwegian Directorate of Health. Norway Patient Register 2003
Ischemic Stroke	Ministry of Health (Vietnam). Vietnam Hospital Data 2013
Ischemic Stroke	World Health Organization Regional Office for Europe (WHO/Europe). European Health for All Database - Inpatient Care Discharges Per 100. Copenhagen, Denmark: World Health Organization Regional Office for Europe (WHO/Europe)
Ischemic Stroke	Norwegian Directorate of Health. Norway Patient Register 2013
Ischemic Stroke	Norwegian Directorate of Health. Norway Patient Register 2014
Ischemic Stroke	Norwegian Directorate of Health. Norway Patient Register 2001
Ischemic Stroke	Norwegian Directorate of Health. Norway Patient Register 2000
Ischemic Stroke	Norwegian Directorate of Health. Norway Patient Register 1999
Ischemic Stroke	Norwegian Directorate of Health. Norway Patient Register 1998
Ischemic Stroke	Norwegian Directorate of Health. Norway Patient Register 1997
Ischemic Stroke	Norwegian Directorate of Health. Norway Patient Register 1996
Ischemic Stroke	Norwegian Directorate of Health. Norway Patient Register 1995
Ischemic Stroke	Norwegian Directorate of Health. Norway Patient Register 1994
Ischemic Stroke	Norwegian Directorate of Health. Norway Patient Register 1993
Ischemic Stroke	Norwegian Directorate of Health. Norway Patient Register 1992
Ischemic Stroke	Norwegian Directorate of Health. Norway Patient Register 1991
Ischemic Stroke	Norwegian Directorate of Health. Norway Patient Register 1990
Ischemic Stroke	Norwegian Directorate of Health. Norway Patient Register 1989
Ischemic Stroke	Norwegian Directorate of Health. Norway Patient Register 1988
Ischemic Stroke	Norwegian Directorate of Health. Norway Patient Register 1987
Ischemic Stroke	Norwegian Directorate of Health. Norway Patient Register 1986
Ischemic Stroke	Norwegian Directorate of Health. Norway Patient Register 1985
Ischemic Stroke	Norwegian Directorate of Health. Norway Patient Register 1984
Ischemic Stroke	Norwegian Directorate of Health. Norway Patient Register 1983
Ischemic Stroke	Norwegian Directorate of Health. Norway Patient Register 1982
Ischemic Stroke	Norwegian Directorate of Health. Norway Patient Register 1981
Ischemic Stroke	Norwegian Directorate of Health. Norway Patient Register 1980
Ischemic Stroke	Norwegian Directorate of Health. Norway Patient Register 1979
Ischemic Stroke	Norwegian Directorate of Health. Norway Patient Register 1978
Ischemic Stroke	Norwegian Directorate of Health. Norway Patient Register 1977
Ischemic Stroke	Norwegian Directorate of Health. Norway Patient Register 1975
Ischemic Stroke	Norwegian Directorate of Health. Norway Patient Register 1973
Ischemic Stroke	Statistics Canada. Canada Community Health Survey 2000-2001. Ottawa, Canada: Statistics Canada, 2003
Ischemic Stroke	Statistics Indonesia. Indonesia National Socioeconomic Survey 2005
Ischemic Stroke	RAND Corporation, University of Indonesia. Indonesia Family Life Survey 1993-1994. Santa Monica, United States: RAND Corporation
Ischemic Stroke	Center for Population and Policy Studies, Gadjah Mada University (Indonesia), RAND Corporation, SurveyMETER. Indonesia Family Life Survey 2007-2008. Santa Monica, United States: RAND Corporation
Ischemic Stroke	Central Bureau of Statistics (Indonesia). Indonesia National Socioeconomic Survey 1992
Ischemic Stroke	Central Bureau of Statistics (Indonesia). Indonesia National Socioeconomic Survey 1993
Ischemic Stroke	Central Bureau of Statistics (Indonesia). Indonesia National Socioeconomic Survey 1994
Ischemic Stroke	Central Bureau of Statistics (Indonesia), Ministry of Health (Indonesia), United Nations Children's Fund (UNICEF). Indonesia National Socioeconomic Survey 1995
Ischemic Stroke	Central Bureau of Statistics (Indonesia), Ministry of Health (Indonesia), United Nations Children's Fund (UNICEF). Indonesia National Socioeconomic Survey 1996
Ischemic Stroke	Central Bureau of Statistics (Indonesia), Ministry of Health (Indonesia), United Nations Children's Fund (UNICEF). Indonesia National Socioeconomic Survey 1997
Ischemic Stroke	Central Bureau of Statistics (Indonesia), Ministry of Health (Indonesia), World Bank. Indonesia National Socioeconomic Survey 2000
Ischemic Stroke	Central Bureau of Statistics (Indonesia), Ministry of Health (Indonesia), World Bank. Indonesia National Socioeconomic Survey 2001
Ischemic Stroke	Statistics Indonesia. Indonesia National Socioeconomic Survey 2004
Ischemic Stroke	National Institute of Statistics and Censuses (Nicaragua), World Bank. Nicaragua Living Standards Measurement Survey 1993
Ischemic Stroke	National Institute of Statistics and Censuses (Nicaragua), World Bank. Nicaragua Living Standards Measurement Survey 1998-1999
Ischemic Stroke	Central Statistical Office (Zambia). Zambia Living Conditions Monitoring Survey 2002-2003. Lusaka, Zambia: Central Statistical Office (Zambia)
Ischemic Stroke	Central Statistical Office (Zambia). Zambia Living Conditions Monitoring Survey 2004-2005. Lusaka, Zambia: Central Statistical Office (Zambia)
Ischemic Stroke	Australian Bureau of Statistics. Australia National Health Survey 1995. Canberra, Australia: Australian Bureau of Statistics
Ischemic Stroke	Analytical and Information Center of the Ministry of Health of Uzbekistan, Macro International, Inc, Ministry of Macroeconomics and Statistics (Uzbekistan). Uzbekistan Special Demographic and Health Survey 2002. Calverton, United States: Macro International, Inc
Ischemic Stroke	World Health Organization (WHO). Bangladesh World Health Survey 2003. Geneva, Switzerland: World Health Organization (WHO), 2005
Ischemic Stroke	World Health Organization (WHO). Burkina Faso World Health Survey 2002-2003. Geneva, Switzerland: World Health Organization (WHO), 2005
Ischemic Stroke	World Health Organization (WHO). Dominican Republic World Health Survey 2003. Geneva, Switzerland: World Health Organization (WHO), 2005
Ischemic Stroke	World Health Organization (WHO). Germany World Health Survey 2004. Geneva, Switzerland: World Health Organization (WHO), 2005
Ischemic Stroke	World Health Organization (WHO). Mali World Health Survey 2003. Geneva, Switzerland: World Health Organization (WHO), 2005
Ischemic Stroke	World Health Organization (WHO). Portugal World Health Survey 2003. Geneva, Switzerland: World Health Organization (WHO), 2006
Ischemic Stroke	World Health Organization (WHO). Zambia World Health Survey 2003. Geneva, Switzerland: World Health Organization (WHO), 2005
Ischemic Stroke	Federal Environment Agency (Germany), Federal Institute for Drugs and Medical Devices (Germany), Max Planck Institute of Psychiatry, Robert Koch Institute. Germany National Health Interview and Examination Survey 1997-1999. Berlin, Germany: Robert Koch Institute, 2000
Ischemic Stroke	Centre for Health Promotion Studies, National University of Ireland, Galway, Health Promotion Unit, Department of Health and Children (Ireland). Ireland Survey of Lifestyle Attitudes and Nutrition 1998. Dublin, Ireland: Health Promotion Unit, Department of Health and Children (Ireland)
Ischemic Stroke	Statistics Indonesia. Indonesia National Socioeconomic Survey 2010
Ischemic Stroke	Family Health International, Ministry of Health (Indonesia), National AIDS Commission (KPA), Statistics Indonesia. Indonesia Behavioral Surveillance Survey 2007
Ischemic Stroke	Statistics Indonesia. Indonesia National Socioeconomic Survey 2002
Ischemic Stroke	Statistics Indonesia. Indonesia National Socioeconomic Survey 2008
Ischemic Stroke	Statistics Indonesia. Indonesia National Socioeconomic Survey - Poverty Program Evaluation 2006. Jakarta, Indonesia: Statistics Indonesia

Ischemic Stroke	Statistics Indonesia. Indonesia National Socioeconomic Survey - Poverty Program Evaluation 2008-2009. Jakarta, Indonesia: Statistics Indonesia
Ischemic Stroke	Federal Statistical Office (Germany). Germany Hospital Discharges by Diagnosis 2009. Wiesbaden, Germany: Federal Statistical Office (Germany), 2011
Ischemic Stroke	Hamad Medical Corporation (Qatar). Qatar - Annual Inpatients Discharge Abstract: Hamad General Hospital 2002. Doha, Qatar: Hamad Medical Corporation (Qatar)
Ischemic Stroke	Hamad Medical Corporation (Qatar). Qatar - Annual Inpatients Discharge Abstract: Hamad General Hospital and Women's Hospital 2003. Doha, Qatar: Hamad Medical Corporation (Qatar)
Ischemic Stroke	Statistics Indonesia. Indonesia National Socioeconomic Survey 2011
Ischemic Stroke	Health Care International, World Health Organization (WHO). Egypt WHO Multi-country Survey Study on Health and Health System Responsiveness 2000-2001. Geneva, Switzerland: World Health Organization (WHO)
Ischemic Stroke	Institute of Health Systems (India), World Health Organization (WHO). India - Andhra Pradesh WHO Multi-country Survey Study on Health and Health System Responsiveness 2000-2001
Ischemic Stroke	Thrift AG, Dewey HM, Sturm JW, Srikanth VK, Gilligan AK, Gall SL, Macdonell RAL, McNeil JJ, Donnan GA. Incidence of stroke subtypes in the North East Melbourne Stroke Incidence Study (NEMESIS): differences between men and women. Neuroepidemiology. 2009; 32(1): 11-8
Ischemic Stroke	Islam MS, Anderson CS, Hankey GJ, Hardie K, Carter K, Broadhurst R, Jamrozik K. Trends in Incidence and Outcome of Stroke in Perth, Western Australia During 1989 to 2001. Stroke. 2008; 39(3): 776-82
Ischemic Stroke	Kolominsky-Rabas PL, Sarti C, Heuschmann PU, Graf C, Siemonsen S, Neundoerfer B, Katalinic A, Lang E, Gassmann K-G, von Stockert TR. A Prospective Community-Based Study of Stroke in Germany-The Erlangen Stroke Project (ESPro): Incidence and Case Fatality at 1, 3, and 12 Months. Stroke. 1998; 29(12): 2501-6
Ischemic Stroke	Thrift AG, Dewey HM, Macdonell RAL, McNeil JJ, Donnan GA. Incidence of the Major Stroke Subtypes: Initial Findings From the North East Melbourne Stroke Incidence Study (NEMESIS). Stroke. 2001; 32(8): 1732-8
Ischemic Stroke	Correia M, Silva MR, Matos I, Magalhães R, Lopes JC, Ferro JM, Silva MC. Prospective Community-Based Study of Stroke in Northern Portugal: Incidence and Case Fatality in Rural and Urban Populations. Stroke. 2004; 35(9): 2048-53
Ischemic Stroke	Hamad A, Hamad A, Sokrab TEO, Momeni S, Mesraoua B, Lingren A. Stroke in Qatar: A one-year, hospital-based study. J Stroke Cerebrovasc Dis. 2001; 10(5): 236-41
Ischemic Stroke	Thrift AG, Dewey HM, Macdonell RAL, McNeil JJ, Donnan GA. Stroke Incidence on the East Coast of Australia: The North East Melbourne Stroke Incidence Study (NEMESIS). Stroke. 2000; 31(9): 2087-92
Ischemic Stroke	Palm F, Urbanek C, Rose S, Buggle F, Bode B, Hennerici MG, Schmieder K, Inselmann G, Reiter R, Fleischer R, Piplack K-O, Safer A, Becher H, Grau AJ. Stroke Incidence and Survival in Ludwigshafen am Rhein, Germany: the Ludwigshafen Stroke Study (LuSSt). Stroke. 2010; 41(9): 1865-70
Ischemic Stroke	Jungehülsing GJ, Müller-Nordhorn J, Nolte CH, Roll S, Rossnagel K, Reich A, Wagner A, Einhäupl KM, Willich SN, Villringer A. Prevalence of stroke and stroke symptoms: a population-based survey of 28,090 participants. Neuroepidemiology. 2008; 30(1): 51-7
Ischemic Stroke	Nicoletti A, Sofia V, Giuffrida S, Bartoloni A, Bartalesi F, Bartolo MLL, Fermo SL, Cocuzza V, Gamboa H, Salazar E, Reggio A. Prevalence of Stroke: A Door-to-Door Survey in Rural Bolivia. Stroke. 2000; 31(4): 882-5
Ischemic Stroke	Salonen JT, Puska P, Tuomilehto J. Physical activity and risk of myocardial infarction, cerebral stroke and death: a longitudinal study in Eastern Finland. Am J Epidemiol. 1982; 115(4): 526-37
Ischemic Stroke	Bijnen FC, Caspersen CJ, Feskens EJ, Saris WH, Mosterd WL, Kromhout D. Physical activity and 10-year mortality from cardiovascular diseases and all causes: The Zutphen Elderly Study. Arch Intern Med. 1998; 158(14): 1499-505
Ischemic Stroke	Lee IM, Hennekens CH, Berger K, Buring JE, Manson JE. Exercise and risk of stroke in male physicians. Stroke. 1999; 30(1): 1-6
Ischemic Stroke	Abbott RD, Rodriguez BL, Burchfiel CM, Curb JD. Physical activity in older middle-aged men and reduced risk of stroke: the Honolulu Heart Program. Am J Epidemiol. 1994; 139(9): 881-93
Ischemic Stroke	Agnarsson U, Thorgeirsson G, Sigvaldason H, Sigfusson N. Effects of leisure-time physical activity and ventilatory function on risk for stroke in men: the Reykjavík Study. Ann Intern Med. 1999; 130(12): 987-90
Ischemic Stroke	Okada H, Horibe H, Yoshiyuki O, Hayakawa N, Aoki N. A prospective study of cerebrovascular disease in Japanese rural communities, Akabane and Asahi. Part 1: evaluation of risk factors in the occurrence of cerebral hemorrhage and thrombosis. Stroke. 1976; 7(6): 599-607
Ischemic Stroke	Paganini-Hill A, Perez Barreto M. Stroke risk in older men and women: aspirin, estrogen, exercise, vitamins, and other factors. J Gend Specif Med. 2001; 4(2): 18-28
Ischemic Stroke	Paffenbarger RS Jr, Brand RJ, Sholtz RI, Jung DL. Energy expenditure, cigarette smoking, and blood pressure level as related to death from specific diseases. Am J Epidemiol. 1978; 108(1): 12-8
Ischemic Stroke	Hu FB, Stampfer MJ, Colditz GA, Ascherio A, Rexrode KM, Willett WC, Manson JE. Physical activity and risk of stroke in women. JAMA. 2000; 283(22): 2961-7
Ischemic Stroke	Ellekjaer H, Holmen J, Ellekjaer E, Vatten L. Physical activity and stroke mortality in women. Ten-year follow-up of the Nord-Trondelag health survey, 1984-1986. Stroke. 2000; 31(1): 14-8
Ischemic Stroke	Håheim LL, Holme I, Hjermann I, Leren P. Risk factors of stroke incidence and mortality. A 12-year follow-up of the Oslo Study. Stroke. 1993; 24(10): 1484-9
Ischemic Stroke	Lindenstrøm E, Boysen G, Nyboe J. Lifestyle factors and risk of cerebrovascular disease in women. The Copenhagen City Heart Study. Stroke. 1993; 24(10): 1468-72
Ischemic Stroke	Wannamethee G, Shaper AG. Physical activity and stroke in British middle aged men. BMJ. 1992; 304(6827): 597-601
Ischemic Stroke	Lapidus L, Bengtsson C. Socioeconomic factors and physical activity in relation to cardiovascular disease and death. A 12 year follow up of participants in a population study of women in Gothenburg, Sweden. Br Heart J. 1986; 55(3): 295-301
Ischemic Stroke	Simonsick EM, Lafferty ME, Phillips CL, Mendes de Leon CF, Kasl SV, Seeman TE, Fillenbaum G, Hebert P, Lemke JH. Risk due to inactivity in physically capable older adults. Am J Public Health. 1993; 83(10): 1443-50
Ischemic Stroke	Lee IM, Paffenbarger RS Jr. Physical activity and stroke incidence: the Harvard Alumni Health Study. Stroke. 1998; 29(10): 2049-54
Ischemic Stroke	Palm F, Dos Santos M, Urbanek C, Greulich M, Zimmer K, Safer A, Grau AJ, Becher H. Stroke seasonality associations with subtype, etiology and laboratory results in the Ludwigshafen Stroke Study (LuSSt). Eur J Epidemiol. 2013; 28(5): 373-81
Ischemic Stroke	Ferri CP, Schoenborn C, Kalra L, Acosta D, Guerra M, Huang Y, Jacob KS, Rodriguez JJL, Salas A, Sosa AL, Williams JD, Liu Z, Moriyama T, Valhuerdi A, Prince MJ. Prevalence of stroke and related burden among older people living in Latin America, India and China. J Neurol Neurosurg Psychiatr. 2011; 82(10): 1074-82
Ischemic Stroke	Sienkiewicz-Jarosz H, Gluszkiewicz M, Pniewski J, Niewada M, Czlonkowska A, Wolfe C, Ryglewicz D. Incidence and case fatality rates of first-ever stroke - comparison of data from two prospective population-based studies conducted in Warsaw. Neurol Neurochir Pol. 2011; 45(3): 207-12
Ischemic Stroke	Wawrzynczyk M, Pierzchala K, Braczkowska B, Manka-Gaca I, Kumor K, Borowski D, Grodzicka-Zawisza L, Zejda J. Estimates of stroke incidence and case fatality in Zabrze, 2005-2006. Neurol Neurochir Pol. 2011; 45(1): 3-10
Ischemic Stroke	Leyden JM, Kleinig TJ, Newbury J, Castle S, Cranefield J, Anderson CS, Crotty M, Whitford D, Jannes J, Lee A, Greenhill J. Adelaide stroke incidence study: declining stroke rates but many preventable cardioembolic strokes. Stroke. 2013; 44(5): 1226-31
Ischemic Stroke	Ministry of Health (Nicaragua), National Institute for Development Information (Nicaragua). Nicaragua National Demographic and Health Survey 2011-2012. Managua, Nicaragua: National Institute for Development Information (Nicaragua)
Ischemic Stroke	Robert Koch Institute. Germany Health Update 2009-2010. Berlin, Germany: Robert Koch Institute

Ischemic Stroke	Jucha R. Stroke incidence and casefatality rates in population of Krosno County. Przegl Lek. 2013; 70(4): 191-4
Ischemic Stroke	Statistics Canada. Canada Community Health Survey 2005. Ottawa, Canada: Statistics Canada
Ischemic Stroke	Statistics Canada. Canada Community Health Survey 2007-2008. Ottawa, Canada: Statistics Canada, 2009
Ischemic Stroke	Katzenellenbogen JM, Vos T, Somerford P, Begg S, Semmens JB, Codde JP. Excess Mortality Rates for Estimating the Non-Fatal Burden of Stroke in Western Australia: A Data Linkage Study. Cerebrovasc Dis. 2010; 30(1): 57-64
Ischemic Stroke	Chiuve SE, Rexrode KM, Spiegelman D, Logroscino G, Manson JE, Rimm EB. Primary prevention of stroke by healthy lifestyle. Circulation. 2008; 118(9): 947–954
Ischemic Stroke	Autenrieth CS, Evenson KR, Yatsuya H, Shahar E, Baggett C, Rosamond WD. Association between physical activity and risk of stroke subtypes: the atherosclerosis risk in communities study. Neuroepidemiology. 2013; 40(2): 109–116
Ischemic Stroke	Myint PK, Luben RN, Wareham NJ, Welch AA, Bingham SA, Day NE, Khaw K-T. Combined work and leisure physical activity and risk of stroke in men and women in the European prospective investigation into Cancer-Norfolk Prospective Population Study. Neuroepidemiology. 2006; 27(3): 122–129
Ischemic Stroke	Sattelmair JR, Kurth T, Buring JE, Lee I-M. Physical activity and risk of stroke in women. Stroke. 2010; 41(6): 1243–50
Ischemic Stroke	Willey JZ, Moon YP, Paik MC, Boden-Albala B, Sacco RL, Elkind MSV. Physical activity and risk of ischemic stroke in the Northern Manhattan Study. Neurology. 2009; 73(21): 1774–1779
Ischemic Stroke	Zhang Q, Zhou Y, Gao X, Wang C, Zhang S, Wang A, Li N, Bian L, Wu J, Jia Q, Wu S, Zhao X. Ideal cardiovascular health metrics and the risks of ischemic and intracerebral hemorrhagic stroke. Stroke. 2013; 44(9): 2451–2456
Ischemic Stroke	Clinton Health Access Initiative (CHAI), Institute for Health Metrics and Evaluation (IHME), Ministry of Health (Zambia), University of Zambia. Access, Bottlenecks, Costs, and Equity (ABCE) project in Zambia, 2011-2012. Seattle, United States: Institute for Health Metrics and Evaluation (IHME), 2015
Ischemic Stroke	Hu G, Sarti C, Jousilahti P, Silventoinen K, Barengo NC, Tuomilehto J. Leisure time, occupational, and commuting physical activity and the risk of stroke. Stroke. 2005; 36(9): 1994–9
Ischemic Stroke	TQA Research, World Health Organization (WHO). Australia WHO Multi-country Survey Study on Health and Health System Responsiveness 2000-2001. Geneva, Switzerland: World Health Organization (WHO)
Ischemic Stroke	Environics Research Group, World Health Organization (WHO). Canada WHO Multi-country Survey Study on Health and Health System Responsiveness 2000-2001. Geneva, Switzerland: World Health Organization (WHO)
Ischemic Stroke	International Research Associates (INRA) Europe, World Health Organization (WHO). Germany WHO Multi-country Survey Study on Health and Health System Responsiveness 2000-2001. Geneva, Switzerland: World Health Organization (WHO)
Ischemic Stroke	Public Opinion Research Center (CBOS) (Poland), World Health Organization (WHO). Poland WHO Multi-country Survey Study on Health and Health System Responsiveness 2000-2001. Geneva, Switzerland: World Health Organization (WHO)
Ischemic Stroke	International Research Associates (INRA) Europe, World Health Organization (WHO). Portugal WHO Multi-country Survey Study on Health and Health System Responsiveness 2000-2001. Geneva, Switzerland: World Health Organization (WHO)
Ischemic Stroke	Börsch-Supan, A. (2015). Survey of Health, Ageing and Retirement in Europe (SHARE) Wave 5. Release version: 1.0.0. SHARE-ERIC. Data set. DOI: 10.6103/SHARE.w5.100
Ischemic Stroke	National Team for the Acceleration of Poverty Reduction (TNP2K) (Indonesia), SurveyMETER, University of Southern California, World Bank. Indonesia Family Life Survey East 2012
Ischemic Stroke	Concluzia-Prim Center for Survey Methodology (Moldova), Independent Sociology and Information Service (OPINIA) (Moldova), Institute for Advanced Studies (Austria), London School of Hygiene and Tropical Medicine, University of Aberdeen. Moldova Health in Times of Transition Household Survey 2010
Ischemic Stroke	Calling S, Hedblad B, Engström G, Berglund G, Janzon L. Effects of body fatness and physical activity on cardiovascular risk: risk prediction using the bioelectrical impedance method. Scand J Public Health. 2006; 34(6): 568-75
Ischemic Stroke	Gulsvik AK, Thelle DS, Samuelsen SO, Myrstad M, Mowé M, Wyller TB. Ageing, physical activity and mortality—a 42-year follow-up study. Int J Epidemiol. 2012-41(2): 521-30
Ischemic Stroke	Ministry of Rural Development (Mali), National Institute of Statistics (INSTAT) (Mali), World Bank. Mali Agricultural Integrated Economic Survey 2014-2015. Washington DC, United States: World Bank
Ischemic Stroke	RAND Corporation, SurveyMETER. Indonesia Family Life Survey 2014-2015. Santa Monica, United States: RAND Corporation, 2016
Ischemic Stroke	Leonards CO, Ipsen N, Malzahn U, Fiebach JB, Endres M, Ebinger M. White matter lesion severity in mild acute ischemic stroke patients and functional outcome after 1 year. Stroke. 2012; 43(11): 3046–51
Ischemic Stroke	Ministry of Health (Portugal). Portugal Hospital Inpatient Discharges 2015
Ischemic Stroke	Ministry of Health (Poland), National Institute of Public Health-National Institute of Hygiene (NIPH-NIH) (Poland). Poland Hospital Inpatient Discharges 2003
Ischemic Stroke	Ministry of Health (Poland), National Institute of Public Health-National Institute of Hygiene (NIPH-NIH) (Poland). Poland Hospital Inpatient Discharges 2004
Ischemic Stroke	Federal Statistical Office (Germany). Germany Federal Health Reporting Hospital Discharges 2000
Ischemic Stroke	Federal Statistical Office (Germany). Germany Federal Health Reporting Hospital Discharges 2001
Ischemic Stroke	Federal Statistical Office (Germany). Germany Federal Health Reporting Hospital Discharges 2002
Ischemic Stroke	Federal Statistical Office (Germany). Germany Federal Health Reporting Hospital Discharges 1990
Ischemic Stroke	Federal Statistical Office (Germany). Germany Federal Health Reporting Hospital Discharges 1991
Ischemic Stroke	Federal Statistical Office (Germany). Germany Federal Health Reporting Hospital Discharges 1992
Ischemic Stroke	Federal Statistical Office (Germany). Germany Federal Health Reporting Hospital Discharges 1993
Ischemic Stroke	Federal Statistical Office (Germany). Germany Federal Health Reporting Hospital Discharges 1994
Ischemic Stroke	Federal Statistical Office (Germany). Germany Federal Health Reporting Hospital Discharges 1995
Ischemic Stroke	Federal Statistical Office (Germany). Germany Federal Health Reporting Hospital Discharges 1996
Ischemic Stroke	Federal Statistical Office (Germany). Germany Federal Health Reporting Hospital Discharges 1997
Ischemic Stroke	Federal Statistical Office (Germany). Germany Federal Health Reporting Hospital Discharges 1998
Ischemic Stroke	Federal Statistical Office (Germany). Germany Federal Health Reporting Hospital Discharges 1999
Ischemic Stroke	Federal Statistical Office (Germany). Germany Federal Health Reporting Hospital Discharges 2013
Ischemic Stroke	Federal Statistical Office (Germany). Germany Federal Health Reporting Hospital Discharges 2014
Ischemic Stroke	Statistics Portugal. Portugal Hospital Inpatient Discharges 1985
Ischemic Stroke	Statistics Portugal. Portugal Hospital Inpatient Discharges 1986
Ischemic Stroke	Statistics Portugal. Portugal Hospital Inpatient Discharges 1987
Ischemic Stroke	Statistics Portugal. Portugal Hospital Inpatient Discharges 1988
Ischemic Stroke	Statistics Portugal. Portugal Hospital Inpatient Discharges 1989
Ischemic Stroke	Statistics Portugal. Portugal Hospital Inpatient Discharges 1990
Ischemic Stroke	Statistics Portugal. Portugal Hospital Inpatient Discharges 1991
Ischemic Stroke	Statistics Portugal. Portugal Hospital Inpatient Discharges 1992
Ischemic Stroke	Statistics Portugal. Portugal Hospital Inpatient Discharges 1993

Ischemic Stroke	Statistics Portugal. Portugal Hospital Inpatient Discharges 1994
Ischemic Stroke	Statistics Portugal. Portugal Hospital Inpatient Discharges 1995
Ischemic Stroke	Statistics Portugal. Portugal Hospital Inpatient Discharges 1996
Ischemic Stroke	Statistics Portugal. Portugal Hospital Inpatient Discharges 1997
Ischemic Stroke	Statistics Portugal. Portugal Hospital Inpatient Discharges 1998
Ischemic Stroke	Statistics Portugal. Portugal Hospital Inpatient Discharges 1999
Ischemic Stroke	Statistics Portugal. Portugal Hospital Inpatient Discharges 2000
Ischemic Stroke	Statistics Portugal. Portugal Hospital Inpatient Discharges 2001
Ischemic Stroke	Statistics Portugal. Portugal Hospital Inpatient Discharges 2002
Ischemic Stroke	Statistics Portugal. Portugal Hospital Inpatient Discharges 2003
Ischemic Stroke	Statistics Portugal. Portugal Hospital Inpatient Discharges 2004
Ischemic Stroke	Statistics Portugal. Portugal Hospital Inpatient Discharges 2005
Ischemic Stroke	Statistics Portugal. Portugal Hospital Inpatient Discharges 2006
Ischemic Stroke	Statistics Portugal. Portugal Hospital Inpatient Discharges 2007
Ischemic Stroke	Statistics Portugal. Portugal Hospital Inpatient Discharges 2008
Ischemic Stroke	Statistics Portugal. Portugal Hospital Inpatient Discharges 2009
Ischemic Stroke	Statistics Portugal. Portugal Hospital Inpatient Discharges 2010
Ischemic Stroke	Statistics Portugal. Portugal Hospital Inpatient Discharges 2011
Ischemic Stroke	Statistics Portugal. Portugal Hospital Inpatient Discharges 2012
Ischemic Stroke	Statistics Portugal. Portugal Hospital Inpatient Discharges 2013
Ischemic Stroke	Statistics Portugal. Portugal Hospital Inpatient Discharges 2014
Ischemic Stroke	Ministry of Health (Poland). Poland Hospital Inpatient Discharges 2002
Ischemic Stroke	Ministry of Health (Poland). Poland Hospital Inpatient Discharges 2001
Ischemic Stroke	Ministry of Health (Poland). Poland Hospital Inpatient Discharges 2000
Ischemic Stroke	Ministry of Health (Poland). Poland Hospital Inpatient Discharges 1999
Ischemic Stroke	Ministry of Health (Poland). Poland Hospital Inpatient Discharges 1998
Ischemic Stroke	Ministry of Health (Poland). Poland Hospital Inpatient Discharges 1997
Ischemic Stroke	Ministry of Health (Poland). Poland Hospital Inpatient Discharges 1996
Ischemic Stroke	Ministry of Health (Poland). Poland Hospital Inpatient Discharges 1995
Ischemic Stroke	Ministry of Health (Poland). Poland Hospital Inpatient Discharges 1994
Ischemic Stroke	Ministry of Health (Poland). Poland Hospital Inpatient Discharges 1993
Ischemic Stroke	Ministry of Health (Poland). Poland Hospital Inpatient Discharges 1992
Ischemic Stroke	Ministry of Health (Poland). Poland Hospital Inpatient Discharges 1991
Ischemic Stroke	Ministry of Health (Poland). Poland Hospital Inpatient Discharges 1990
Ischemic Stroke	Ministry of Health (Poland). Poland Hospital Inpatient Discharges 1989
Ischemic Stroke	Ministry of Health (Poland). Poland Hospital Inpatient Discharges 1988
Ischemic Stroke	Ministry of Health (Poland). Poland Hospital Inpatient Discharges 1987
Ischemic Stroke	Ministry of Health (Poland). Poland Hospital Inpatient Discharges 1986
Ischemic Stroke	Ministry of Health (Poland). Poland Hospital Inpatient Discharges 1985
Ischemic Stroke	Ministry of Health (Poland). Poland Hospital Inpatient Discharges 1984
Ischemic Stroke	Ministry of Health (Poland). Poland Hospital Inpatient Discharges 1983
Ischemic Stroke	Ministry of Health (Poland). Poland Hospital Inpatient Discharges 1982
Ischemic Stroke	Ministry of Health (Poland). Poland Hospital Inpatient Discharges 1981
Ischemic Stroke	Ministry of Health (Poland). Poland Hospital Inpatient Discharges 1980
Ischemic Stroke	Ministry of Health (Poland). Poland Hospital Inpatient Discharges 2013
Ischemic Stroke	Ministry of Health (Poland). Poland Hospital Inpatient Discharges 2014
Ischemic Stroke	Census and Statistics Directorate (Panama), Ministry of Economy and Finance (Panama), World Bank. Panama Living Standard Measurement Survey 2003. Washington DC, United States: World Bank
Ischemic Stroke	Carolina Population Center, University of North Carolina at Chapel Hill, Institute of Sociology, Russian Academy of Sciences, National Research University Higher School of Economics (Russia), ZAO Demoscope. Russia Longitudinal Monitoring Survey of HSE, Round II 1992-1993
Ischemic Stroke	Carolina Population Center, University of North Carolina at Chapel Hill, Institute of Sociology, Russian Academy of Sciences, National Research University Higher School of Economics (Russia), ZAO Demoscope. Russia Longitudinal Monitoring Survey of HSE, Round IV 1993-1994
Ischemic Stroke	Russia Longitudinal Monitoring Survey (RLMS-HSE), Round VII 1996. National Research University Higher School of Economics, ZAO Demoscope, Carolina Population Center, Univeristy of North Carolina at Chapel Hill, Institute of Sociology, Russian Academy of Sciences
Ischemic Stroke	Russia Longitudinal Monitoring Survey (RLMS-HSE), Round VIII 1998-1999. National Research University Higher School of Economics, ZAO Demoscope, Carolina Population Center, Univeristy of North Carolina at Chapel Hill, Institute of Sociology, Russian Academy of Sciences
Ischemic Stroke	Russia Longitudinal Monitoring Survey (RLMS-HSE), Round X 2001. National Research University Higher School of Economics, ZAO Demoscope, Carolina Population Center, Univeristy of North Carolina at Chapel Hill, Institute of Sociology, Russian Academy of Sciences
Ischemic Stroke	Russia Longitudinal Monitoring Survey (RLMS-HSE), Round XI 2002. National Research University Higher School of Economics, ZAO Demoscope, Carolina Population Center, Univeristy of North Carolina at Chapel Hill, Institute of Sociology, Russian Academy of Sciences
Ischemic Stroke	Russia Longitudinal Monitoring Survey (RLMS-HSE), Round XII 2003. National Research University Higher School of Economics, ZAO Demoscope, Carolina Population Center, Univeristy of North Carolina at Chapel Hill, Institute of Sociology, Russian Academy of Sciences
Ischemic Stroke	Russia Longitudinal Monitoring Survey (RLMS-HSE), Round XIII 2004. National Research University Higher School of Economics, ZAO Demoscope, Carolina Population Center, Univeristy of North Carolina at Chapel Hill, Institute of Sociology, Russian Academy of Sciences
Ischemic Stroke	Russia Longitudinal Monitoring Survey (RLMS-HSE), Round XIV 2005. National Research University Higher School of Economics, ZAO Demoscope, Carolina Population Center, Univeristy of North Carolina at Chapel Hill, Institute of Sociology, Russian Academy of Sciences
Ischemic Stroke	Ministry of Health, Social Services and Equality (Spain), National Statistics Institute (Spain). Spain National Health Survey 2006-2007
Ischemic Stroke	Health and Retirement Study, (Biennial 1992) public use dataset. Produced and distributed by the University of Michigan with funding from the National Institute on Aging (grant number NIA U01AG009740). Ann Arbor, MI, (2011)
Ischemic Stroke	and Prevention. National Center for Health Statistics. National Health Interview Survey, 1994: Second Longitudinal Study on Aging, Wave 2, 1997. ICPSR03526-v2. Ann Arbor, MI: Inter-university Consortium for Political and Social Research [distributor], 2007-03-01. http://doi.org/10.3886/ICPSR03526.v2
Ischemic Stroke	World Health Organization (WHO). Ecuador World Health Survey 2003. Geneva, Switzerland: World Health Organization (WHO), 2005
Ischemic Stroke	World Health Organization (WHO). Greece World Health Survey 2003. Geneva, Switzerland: World Health Organization (WHO), 2005
Ischemic Stroke	World Health Organization (WHO). Laos World Health Survey 2003

Ischemic Stroke	World Health Organization (WHO). Malawi World Health Survey 2003. Geneva, Switzerland: World Health Organization (WHO), 2005
Ischemic Stroke	World Health Organization (WHO). Mauritania World Health Survey 2003. Geneva, Switzerland: World Health Organization (WHO), 2005
Ischemic Stroke	World Health Organization (WHO). Russia World Health Survey 2003. Geneva, Switzerland: World Health Organization (WHO), 2005
Ischemic Stroke	World Health Organization (WHO). Spain World Health Survey 2002-2003. Geneva, Switzerland: World Health Organization (WHO), 2005
Ischemic Stroke	National Center for Health Statistics (NCHS), Centers for Disease Control and Prevention (CDC). United States National Health and Nutrition Examination Survey 2007-2008. Hyattsville, United States: National Center for Health Statistics (NCHS), Centers for Disease Control and Prevention (CDC), 2009
Ischemic Stroke	National Center for Health Statistics (NCHS), Centers for Disease Control and Prevention (CDC), US Census Bureau. United States National Health Interview Survey 1990. Hyattsville, United States: National Center for Health Statistics (NCHS), Centers for Disease Control and Prevention (CDC)
Ischemic Stroke	National Center for Health Statistics (NCHS), Centers for Disease Control and Prevention (CDC), US Census Bureau. United States National Health Interview Survey 1991. Hyattsville, United States: National Center for Health Statistics (NCHS), Centers for Disease Control and Prevention (CDC)
Ischemic Stroke	National Center for Health Statistics (NCHS), Centers for Disease Control and Prevention (CDC), US Census Bureau. United States National Health Interview Survey 1992. Hyattsville, United States: National Center for Health Statistics (NCHS), Centers for Disease Control and Prevention (CDC)
Ischemic Stroke	National Center for Health Statistics (NCHS), Centers for Disease Control and Prevention (CDC), US Census Bureau. United States National Health Interview Survey 1993. Hyattville, United States: National Center for Health Statistics (NCHS), Centers for Disease Control and Prevention (CDC)
Ischemic Stroke	National Center for Health Statistics (NCHS), Centers for Disease Control and Prevention (CDC), US Census Bureau. United States National Health Interview Survey 1997. Hyattsville, United States: National Center for Health Statistics (NCHS), Centers for Disease Control and Prevention (CDC)
Ischemic Stroke	National Center for Health Statistics (NCHS), Centers for Disease Control and Prevention (CDC), US Census Bureau. United States National Health Interview Survey 1998. Hyattsville, United States: National Center for Health Statistics (NCHS), Centers for Disease Control and Prevention (CDC)
Ischemic Stroke	National Center for Health Statistics (NCHS), Centers for Disease Control and Prevention (CDC), US Census Bureau. United States National Health Interview Survey 1999. Hyattsville, United States: National Center for Health Statistics (NCHS), Centers for Disease Control and Prevention (CDC)
Ischemic Stroke	National Center for Health Statistics (NCHS), Centers for Disease Control and Prevention (CDC), US Census Bureau. United States National Health Interview Survey 2000. Hyattsville, United States: National Center for Health Statistics (NCHS), Centers for Disease Control and Prevention (CDC)
Ischemic Stroke	National Center for Health Statistics (NCHS), Centers for Disease Control and Prevention (CDC), US Census Bureau. United States National Health Interview Survey 2001. Hyattsville, United States: National Center for Health Statistics (NCHS), Centers for Disease Control and Prevention (CDC)
Ischemic Stroke	National Center for Health Statistics (NCHS), Centers for Disease Control and Prevention (CDC), US Census Bureau. United States National Health Interview Survey 2002. Hyattsville, United States: National Center for Health Statistics (NCHS), Centers for Disease Control and Prevention (CDC)
Ischemic Stroke	National Center for Health Statistics (NCHS), Centers for Disease Control and Prevention (CDC), US Census Bureau. United States National Health Interview Survey 2003. Hyattsville, United States: National Center for Health Statistics (NCHS), Centers for Disease Control and Prevention (CDC)
Ischemic Stroke	National Center for Health Statistics (NCHS), Centers for Disease Control and Prevention (CDC), US Census Bureau. United States National Health Interview Survey 2004. Hyattsville, United States: National Center for Health Statistics (NCHS), Centers for Disease Control and Prevention (CDC)
Ischemic Stroke	National Center for Health Statistics (NCHS), Centers for Disease Control and Prevention (CDC), US Census Bureau. United States National Health Interview Survey 2005. Hyattsville, United States: National Center for Health Statistics (NCHS), Centers for Disease Control and Prevention (CDC)
Ischemic Stroke	National Center for Health Statistics (NCHS), Centers for Disease Control and Prevention (CDC), US Census Bureau. United States National Health Interview Survey 2006. Hyattsville, United States: National Center for Health Statistics (NCHS), Centers for Disease Control and Prevention (CDC)
Ischemic Stroke	National Center for Health Statistics (NCHS), Centers for Disease Control and Prevention (CDC), US Census Bureau. United States National Health Interview Survey 2007. Hyattsville, United States: National Center for Health Statistics (NCHS), Centers for Disease Control and Prevention (CDC)
Ischemic Stroke	National Center for Health Statistics (NCHS), Centers for Disease Control and Prevention (CDC), US Census Bureau. United States National Health Interview Survey 2008. Hyattsville, United States: National Center for Health Statistics (NCHS), Centers for Disease Control and Prevention (CDC)
Ischemic Stroke	National Center for Health Statistics (NCHS), Centers for Disease Control and Prevention (CDC), US Census Bureau. United States National Health Interview Survey 2009. Hyattsville, United States: National Center for Health Statistics (NCHS), Centers for Disease Control and Prevention (CDC)
Ischemic Stroke	National Center for Health Statistics (NCHS), Centers for Disease Control and Prevention (CDC), US Census Bureau. United States National Health Interview Survey 2010. Hyattsville, United States: National Center for Health Statistics (NCHS), Centers for Disease Control and Prevention (CDC)
Ischemic Stroke	National Statistical Office of Malawi, World Bank. Malawi Living Standards Measurement Survey 2004-2005
Ischemic Stroke	National Center for Health Statistics (NCHS), Centers for Disease Control and Prevention (CDC). United States National Health and Nutrition Examination Survey 2005-2006. Hyattsville, United States: National Center for Health Statistics (NCHS), Centers for Disease Control and Prevention (CDC), 2007
Ischemic Stroke	National Center for Health Statistics (NCHS), Centers for Disease Control and Prevention (CDC). United States National Health and Nutrition Examination Survey 2003-2004. Hyattsville, United States: National Center for Health Statistics (NCHS), Centers for Disease Control and Prevention (CDC)
Ischemic Stroke	National Center for Health Statistics (NCHS), Centers for Disease Control and Prevention (CDC). United States National Health and Nutrition Examination Survey 2009-2010. Hyattsville, United States: National Center for Health Statistics (NCHS), Centers for Disease Control and Prevention (CDC), 2011
Ischemic Stroke	National Center for Health Statistics (NCHS), Centers for Disease Control and Prevention (CDC). United States National Health and Nutrition Examination Survey 1988-1994. Hyattsville, United States: National Center for Health Statistics (NCHS), Centers for Disease Control and Prevention (CDC)
Ischemic Stroke	National Center for Health Statistics (NCHS), Centers for Disease Control and Prevention (CDC). United States National Health and Nutrition Examination Survey 2001-2002. Hyattsville, United States: National Center for Health Statistics (NCHS), Centers for Disease Control and Prevention (CDC)
Ischemic Stroke	National Center for Health Statistics (NCHS), Centers for Disease Control and Prevention (CDC). United States National Health and Nutrition Examination Survey 1999-2000. Hyattsville, United States: National Center for Health Statistics (NCHS), Centers for Disease Control and Prevention (CDC)
Ischemic Stroke	Health and Retirement Study, (Biennial 1996) public use dataset. Produced and distributed by the University of Michigan with funding from the National Institute on Aging (grant number NIA U01AG009740). Ann Arbor, MI, (2011)
Ischemic Stroke	Health and Retirement Study, (Biennial 1998) public use dataset. Produced and distributed by the University of Michigan with funding from the National Institute on Aging (grant number NIA U01AG009740). Ann Arbor, MI, (2014)
Ischemic Stroke	Health and Retirement Study, (Biennial 2000) public use dataset. Produced and distributed by the University of Michigan with funding from the National Institute on Aging (grant number NIA U01AG009740). Ann Arbor, MI, (2011)
Ischemic Stroke	Health and Retirement Study, (Biennial 2002) public use dataset. Produced and distributed by the University of Michigan with funding from the National Institute on Aging (grant number NIA U01AG009740). Ann Arbor, MI, (2011)
Ischemic Stroke	Health and Retirement Study, (Biennial 2004) public use dataset. Produced and distributed by the University of Michigan with funding from the National Institute on Aging (grant number NIA U01AG009740). Ann Arbor, MI, (2013)
Ischemic Stroke	Health and Retirement Study, (Biennial 2006) public use dataset. Produced and distributed by the University of Michigan with funding from the National Institute on Aging (grant number NIA U01AG009740). Ann Arbor, MI, (2014)

Ischemic Stroke	Health and Retirement Study, (Biennial 2010) public use dataset. Produced and distributed by the University of Michigan with funding from the National Institute on Aging (grant number NIA U01AG009740). Ann Arbor, MI, (2014)
Ischemic Stroke	Panel Study of Income Dynamics, 2007 public use dataset. Produced and distributed by the University of Michigan with primary funding from the National Science Foundation, the National Institute of Aging, and the National Institute of Child Health and Human Development. Ann Arbor, MI, (2011)
Ischemic Stroke	Panel Study of Income Dynamics, 2005 public use dataset. Produced and distributed by the University of Michigan with primary funding from the National Science Foundation, the National Institute of Aging, and the National Institute of Child Health and Human Development. Ann Arbor, MI, (2011)
Ischemic Stroke	National Center for Health Statistics (NCHS), Centers for Disease Control and Prevention (CDC), United States Census Bureau. United States National Hospital Discharge Survey 1996. Hyattsville, United States: National Center for Health Statistics (NCHS), Centers for Disease Control and Prevention (CDC)
Ischemic Stroke	National Center for Health Statistics (NCHS), Centers for Disease Control and Prevention (CDC), United States Census Bureau. United States National Hospital Discharge Survey 1997. Hyattsville, United States: National Center for Health Statistics (NCHS), Centers for Disease Control and Prevention (CDC)
Ischemic Stroke	National Center for Health Statistics (NCHS), Centers for Disease Control and Prevention (CDC), United States Census Bureau. United States National Hospital Discharge Survey 1998. Hyattsville, United States: National Center for Health Statistics (NCHS), Centers for Disease Control and Prevention (CDC)
Ischemic Stroke	National Center for Health Statistics (NCHS), Centers for Disease Control and Prevention (CDC), United States Census Bureau. United States National Hospital Discharge Survey 1999. Hyattsville, United States: National Center for Health Statistics (NCHS), Centers for Disease Control and Prevention (CDC)
Ischemic Stroke	National Center for Health Statistics (NCHS), Centers for Disease Control and Prevention (CDC), United States Census Bureau. United States National Hospital Discharge Survey 2000. Hyattsville, United States: National Center for Health Statistics (NCHS), Centers for Disease Control and Prevention (CDC)
Ischemic Stroke	National Center for Health Statistics (NCHS), Centers for Disease Control and Prevention (CDC), United States Census Bureau. United States National Hospital Discharge Survey 2001. Hyattsville, United States: National Center for Health Statistics (NCHS), Centers for Disease Control and Prevention (CDC)
Ischemic Stroke	National Center for Health Statistics (NCHS), Centers for Disease Control and Prevention (CDC), United States Census Bureau. United States National Hospital Discharge Survey 2002. Hyattsville, United States: National Center for Health Statistics (NCHS), Centers for Disease Control and Prevention (CDC)
Ischemic Stroke	National Center for Health Statistics (NCHS), Centers for Disease Control and Prevention (CDC), United States Census Bureau. United States National Hospital Discharge Survey 2003. Hyattsville, United States: National Center for Health Statistics (NCHS), Centers for Disease Control and Prevention (CDC)
Ischemic Stroke	National Center for Health Statistics (NCHS), Centers for Disease Control and Prevention (CDC), United States Census Bureau. United States National Hospital Discharge Survey 2004. Hyattsville, United States: National Center for Health Statistics (NCHS), Centers for Disease Control and Prevention (CDC)
Ischemic Stroke	National Center for Health Statistics (NCHS), Centers for Disease Control and Prevention (CDC), United States Census Bureau. United States National Hospital Discharge Survey 2005. Hyattsville, United States: National Center for Health Statistics (NCHS), Centers for Disease Control and Prevention (CDC)
Ischemic Stroke	National Center for Health Statistics (NCHS), Centers for Disease Control and Prevention (CDC), United States Census Bureau. United States National Hospital Discharge Survey 2006. Hyattsville, United States: National Center for Health Statistics (NCHS), Centers for Disease Control and Prevention (CDC)
Ischemic Stroke	National Center for Health Statistics (NCHS), Centers for Disease Control and Prevention (CDC), United States Census Bureau. United States National Hospital Discharge Survey 2007. Hyattsville, United States: National Center for Health Statistics (NCHS), Centers for Disease Control and Prevention (CDC)
Ischemic Stroke	National Center for Health Statistics (NCHS), Centers for Disease Control and Prevention (CDC), United States Census Bureau. United States National Hospital Discharge Survey 2008. Hyattsville, United States: National Center for Health Statistics (NCHS), Centers for Disease Control and Prevention (CDC)
Ischemic Stroke	National Center for Health Statistics (NCHS), Centers for Disease Control and Prevention (CDC), United States Census Bureau. United States National Hospital Discharge Survey 2009. Hyattsville, United States: National Center for Health Statistics (NCHS), Centers for Disease Control and Prevention (CDC)
Ischemic Stroke	National Center for Health Statistics (NCHS), Centers for Disease Control and Prevention (CDC), United States Census Bureau. United States National Hospital Discharge Survey 2010. Hyattsville, United States: National Center for Health Statistics (NCHS), Centers for Disease Control and Prevention (CDC)
Ischemic Stroke	National Center for Health Statistics (NCHS), Centers for Disease Control and Prevention (CDC), United States Census Bureau. United States National Hospital Discharge Survey 1979. Hyattsville, United States: National Center for Health Statistics (NCHS), Centers for Disease Control and Prevention (CDC)
Ischemic Stroke	National Center for Health Statistics (NCHS), Centers for Disease Control and Prevention (CDC), United States Census Bureau. United States National Hospital Discharge Survey 1980. Hyattsville, United States: National Center for Health Statistics (NCHS), Centers for Disease Control and Prevention (CDC)
Ischemic Stroke	National Center for Health Statistics (NCHS), Centers for Disease Control and Prevention (CDC), United States Census Bureau. United States National Hospital Discharge Survey 1981. Hyattsville, United States: National Center for Health Statistics (NCHS), Centers for Disease Control and Prevention (CDC)
Ischemic Stroke	National Center for Health Statistics (NCHS), Centers for Disease Control and Prevention (CDC), United States Census Bureau. United States National Hospital Discharge Survey 1982. Hyattsville, United States: National Center for Health Statistics (NCHS), Centers for Disease Control and Prevention (CDC)
Ischemic Stroke	National Center for Health Statistics (NCHS), Centers for Disease Control and Prevention (CDC), United States Census Bureau. United States National Hospital Discharge Survey 1983. Hyattsville, United States: National Center for Health Statistics (NCHS), Centers for Disease Control and Prevention (CDC)
Ischemic Stroke	National Center for Health Statistics (NCHS), Centers for Disease Control and Prevention (CDC), United States Census Bureau. United States National Hospital Discharge Survey 1984. Hyattsville, United States: National Center for Health Statistics (NCHS), Centers for Disease Control and Prevention (CDC)
Ischemic Stroke	National Center for Health Statistics (NCHS), Centers for Disease Control and Prevention (CDC), United States Census Bureau. United States National Hospital Discharge Survey 1985. Hyattsville, United States: National Center for Health Statistics (NCHS), Centers for Disease Control and Prevention (CDC)

Ischemic Stroke	National Center for Health Statistics (NCHS), Centers for Disease Control and Prevention (CDC), United States Census Bureau. United States National Hospital Discharge Survey 1986. Hyattsville, United States: National Center for Health Statistics (NCHS), Centers for Disease Control and Prevention (CDC)
Ischemic Stroke	National Center for Health Statistics (NCHS), Centers for Disease Control and Prevention (CDC), United States Census Bureau. United States National Hospital Discharge Survey 1987. Hyattsville, United States: National Center for Health Statistics (NCHS), Centers for Disease Control and Prevention (CDC)
Ischemic Stroke	National Center for Health Statistics (NCHS), Centers for Disease Control and Prevention (CDC), United States Census Bureau. United States National Hospital Discharge Survey 1988. Hyattsville, United States: National Center for Health Statistics (NCHS), Centers for Disease Control and Prevention (CDC)
Ischemic Stroke	National Center for Health Statistics (NCHS), Centers for Disease Control and Prevention (CDC), United States Census Bureau. United States National Hospital Discharge Survey 1989. Hyattsville, United States: National Center for Health Statistics (NCHS), Centers for Disease Control and Prevention (CDC)
Ischemic Stroke	National Center for Health Statistics (NCHS), Centers for Disease Control and Prevention (CDC), United States Census Bureau. United States National Hospital Discharge Survey 1990. Hyattsville, United States: National Center for Health Statistics (NCHS), Centers for Disease Control and Prevention (CDC)
Ischemic Stroke	National Center for Health Statistics (NCHS), Centers for Disease Control and Prevention (CDC), United States Census Bureau. United States National Hospital Discharge Survey 1991. Hyattsville, United States: National Center for Health Statistics (NCHS), Centers for Disease Control and Prevention (CDC)
Ischemic Stroke	National Center for Health Statistics (NCHS), Centers for Disease Control and Prevention (CDC), United States Census Bureau. United States National Hospital Discharge Survey 1992. Hyattsville, United States: National Center for Health Statistics (NCHS), Centers for Disease Control and Prevention (CDC)
Ischemic Stroke	National Center for Health Statistics (NCHS), Centers for Disease Control and Prevention (CDC), United States Census Bureau. United States National Hospital Discharge Survey 1993. Hyattsville, United States: National Center for Health Statistics (NCHS), Centers for Disease Control and Prevention (CDC)
Ischemic Stroke	National Center for Health Statistics (NCHS), Centers for Disease Control and Prevention (CDC), United States Census Bureau. United States National Hospital Discharge Survey 1994. Hyattsville, United States: National Center for Health Statistics (NCHS), Centers for Disease Control and Prevention (CDC)
Ischemic Stroke	National Center for Health Statistics (NCHS), Centers for Disease Control and Prevention (CDC), United States Census Bureau. United States National Hospital Discharge Survey 1995. Hyattsville, United States: National Center for Health Statistics (NCHS), Centers for Disease Control and Prevention (CDC)
Ischemic Stroke	National Institute of Statistics and Censuses (Ecuador). Ecuador Hospital Inpatient Discharges 1997. Quito, Ecuador: National Institute of Statistics and Censuses (Ecuador)
Ischemic Stroke	National Institute of Statistics and Censuses (Ecuador). Ecuador Hospital Inpatient Discharges 1998. Quito, Ecuador: National Institute of Statistics and Censuses (Ecuador)
Ischemic Stroke	National Institute of Statistics and Censuses (Ecuador). Ecuador Hospital Inpatient Discharges 1999. Quito, Ecuador: National Institute of Statistics and Censuses (Ecuador)
Ischemic Stroke	National Institute of Statistics and Censuses (Ecuador). Ecuador Hospital Inpatient Discharges 2000. Quito, Ecuador: National Institute of Statistics and Censuses (Ecuador)
Ischemic Stroke	National Institute of Statistics and Censuses (Ecuador). Ecuador Hospital Inpatient Discharges 2001. Quito, Ecuador: National Institute of Statistics and Censuses (Ecuador)
Ischemic Stroke	National Institute of Statistics and Censuses (Ecuador). Ecuador Hospital Inpatient Discharges 2002. Quito, Ecuador: National Institute of Statistics and Censuses (Ecuador)
Ischemic Stroke	National Institute of Statistics and Censuses (Ecuador). Ecuador Hospital Inpatient Discharges 2003. Quito, Ecuador: National Institute of Statistics and Censuses (Ecuador)
Ischemic Stroke	National Institute of Statistics and Censuses (Ecuador). Ecuador Hospital Inpatient Discharges 2004. Quito, Ecuador: National Institute of Statistics and Censuses (Ecuador)
Ischemic Stroke	National Institute of Statistics and Censuses (Ecuador). Ecuador Hospital Inpatient Discharges 2005. Quito, Ecuador: National Institute of Statistics and Censuses (Ecuador)
Ischemic Stroke	National Institute of Statistics and Censuses (Ecuador). Ecuador Hospital Inpatient Discharges 2006. Quito, Ecuador: National Institute of Statistics and Censuses (Ecuador)
Ischemic Stroke	National Institute of Statistics and Censuses (Ecuador). Ecuador Hospital Inpatient Discharges 2007. Quito, Ecuador: National Institute of Statistics and Censuses (Ecuador)
Ischemic Stroke	National Institute of Statistics and Censuses (Ecuador). Ecuador Hospital Inpatient Discharges 2008. Quito, Ecuador: National Institute of Statistics and Censuses (Ecuador)
Ischemic Stroke	National Institute of Statistics and Censuses (Ecuador). Ecuador Hospital Inpatient Discharges 2009. Quito, Ecuador: National Institute of Statistics and Censuses (Ecuador)
Ischemic Stroke	National Institute of Statistics and Censuses (Ecuador). Ecuador Hospital Inpatient Discharges 2010. Quito, Ecuador: National Institute of Statistics and Censuses (Ecuador)
Ischemic Stroke	National Institute of Statistics and Censuses (Ecuador). Ecuador Hospital Inpatient Discharges 2011. Quito, Ecuador: National Institute of Statistics and Censuses (Ecuador)
Ischemic Stroke	Healthcare Cost and Utilization Project (HCUP), Agency for Healthcare Research and Quality (AHRQ). United States State Inpatient Databases 2003. Rockville, United States: Healthcare Cost and Utilization Project (HCUP), Agency for Healthcare Research and Quality (AHRQ)
Ischemic Stroke	Healthcare Cost and Utilization Project (HCUP), Agency for Healthcare Research and Quality (AHRQ). United States State Inpatient Databases 2004. Rockville, United States: Healthcare Cost and Utilization Project (HCUP), Agency for Healthcare Research and Quality (AHRQ)
Ischemic Stroke	Healthcare Cost and Utilization Project (HCUP), Agency for Healthcare Research and Quality (AHRQ). United States State Inpatient Databases 2005. Rockville, United States: Healthcare Cost and Utilization Project (HCUP), Agency for Healthcare Research and Quality (AHRQ)
Ischemic Stroke	Healthcare Cost and Utilization Project (HCUP), Agency for Healthcare Research and Quality (AHRQ). United States State Inpatient Databases 2006. Rockville, United States: Healthcare Cost and Utilization Project (HCUP), Agency for Healthcare Research and Quality (AHRQ)
Ischemic Stroke	Healthcare Cost and Utilization Project (HCUP), Agency for Healthcare Research and Quality (AHRQ). United States State Inpatient Databases 2007. Rockville, United States: Healthcare Cost and Utilization Project (HCUP), Agency for Healthcare Research and Quality (AHRQ)
Ischemic Stroke	Healthcare Cost and Utilization Project (HCUP), Agency for Healthcare Research and Quality (AHRQ). United States State Inpatient Databases 2008. Rockville, United States: Healthcare Cost and Utilization Project (HCUP), Agency for Healthcare Research and Quality (AHRQ)
Ischemic Stroke	Healthcare Cost and Utilization Project (HCUP), Agency for Healthcare Research and Quality (AHRQ). United States State Inpatient Databases 2009. Rockville, United States: Healthcare Cost and Utilization Project (HCUP), Agency for Healthcare Research and Quality (AHRQ)

Ischemic Stroke	Health Measurement Research Group, University of Wisconsin. United States National Health Measurement Study 2005-2006. Data and Information Sciences Center, University of Wisconsin-Madison [distributor]
Ischemic Stroke	National Center for Health Statistics (NCHS), Centers for Disease Control and Prevention (CDC), US Census Bureau. United States National Health Interview Survey 2011. Hyattsville, United States: National Center for Health Statistics (NCHS), Centers for Disease Control and Prevention (CDC)
Ischemic Stroke	Ministry of Health and Medical Education (Iran), World Health Organization (WHO). Iran WHO Multi-country Survey Study on Health and Health System Responsiveness 2000-2001. Geneva, Switzerland: World Health Organization (WHO)
Ischemic Stroke	National Statistical Office of Malawi, World Bank. Malawi Integrated Household Survey 2010-2011. Washington DC, United States: World Bank
Ischemic Stroke	Agency for Healthcare Research and Quality. United States Medical Expenditure Panel Survey 2002-2009. Rockville, United States: Agency for Healthcare Research and Quality
Ischemic Stroke	Feigin V, Carter K, Hackett M, Barber PA, McNaughton H, Dyall L, Chen M, Anderson C. Ethnic disparities in incidence of stroke subtypes: Auckland Regional Community Stroke Study, 2002-2003. Lancet Neurol. 2006; 5(2): 130-9
Ischemic Stroke	Feigin VL, Wiebers DO, Nikitin YP, O'Fallon WM, Whisnant JP. Stroke Epidemiology in Novosibirsk, Russia: A Population-Based Study. Mayo Clin Proc. 1995; 70(9): 847-52
Ischemic Stroke	Azarpazhooh MR, Etemadi MM, Donnan GA, Mokhber N, Majdi MR, Ghayour-Mobarhan M, Ghandehary K, Farzadfard MT, Kiani R, Panahandeh M, Thrift AG. Excessive Incidence of Stroke in Iran. Stroke. 2010; 41(1): e3-e10
Ischemic Stroke	Vemmos KN, Bots ML, Tsibouris PK, Zis VP, Grobbee DE, Stranjalis GS, Stamatelopoulos S. Stroke Incidence and Case Fatality in Southern Greece: The Arcadia Stroke Registry. Stroke. 1999; 30(2): 363-70
Ischemic Stroke	al-Rajeh S, Larbi EB, Bademosi O, Awada A, Yousef A, al-Freihi H, Miniawi H. Stroke register: experience from the eastern province of Saudi Arabia. Cerebrovasc Dis. 1998; 8(2): 86-9
Ischemic Stroke	Al Rajeh S. Stroke in the Elderly Aged 75 Years and Above. Cerebrovasc Dis. 1994; 4(6): 402-6
Ischemic Stroke	Anderson CS, Carter KN, Hackett ML, Feigin V, Barber PA, Broad JB, Bonita R. Trends in Stroke Incidence in Auckland, New Zealand, During 1981 to 2003. Stroke. 2005; 36(10): 2087-93
Ischemic Stroke	Awada A. Stroke in Saudi Arabian young adults: a study of 120 cases. Acta Neurol Scand. 1994; 89(5): 323-8
Ischemic Stroke	Bonita R, Broad JB, Beaglehole R. Changes in stroke incidence and case-fatality in Auckland, New Zealand, 1981-91. Lancet. 1993; 342(8885): 1470-3
Ischemic Stroke	Earley CJ, Kittner SJ, Feeser BR, Gardner J, Epstein A, Wozniak MA, Wityk R, Stern BJ, Price TR, Macko RF, Johnson C, Sloan MA, Buchholz D. Stroke in children and sickle-cell disease: Baltimore-Washington Cooperative Young Stroke Study. Neurology. 1998; 51(1): 169-76
Ischemic Stroke	Alzamora MT, Sorribes M, Heras A, Vila N, Vicheto M, Forés R, Sánchez-Ojanguren J, Sancho A, the, Pera G. Ischemic stroke incidence in Santa Coloma de Gramenet (ISISCOG), Spain. A community-based study. BMC Neurol. 2008; 8(1): 5
Ischemic Stroke	Ghandehari K, Moud ZI. Incidence and etiology of ischemic stroke in Persian young adults. Acta Neurol Scand. 2006; 113(2): 121-4
Ischemic Stroke	National Center for Health Statistics (NCHS) Centers for Disease Control and Prevention (CDC). United States National Health and Nutrition Examination Survey 2011-2012. Hyattsville, United States: National Center for Health Statistics (NCHS) Centers for Disease Control and Prevention (CDC), 2013
Ischemic Stroke	National Center for Health Statistics (NCHS), Centers for Disease Control and Prevention (CDC), United States Census Bureau. United States National Health Interview Survey 2012. Hyattsville, United States: National Center for Health Statistics (NCHS), Centers for Disease Control and Prevention (CDC), 2013
Ischemic Stroke	National Institute of Statistics and Censuses (Ecuador). Ecuador Hospital Inpatient Discharges 2012. Quito, Ecuador: National Institute of Statistics and Censuses (Ecuador), 2013
Ischemic Stroke	Talaei M, Sarrafzadegan N, Sadeghi M, Oveisgharan S, Marshall T, Thomas GN, Iranipour R. Incidence of cardiovascular diseases in an Iranian population: the Isfahan Cohort Study. Arch Iran Med. 2013; 16(3): 138-44
Ischemic Stroke	Russia Longitudinal Monitoring Survey (RLMS-HSE), Round XVIII 2009. National Research University Higher School of Economics, ZAO Demoscope, Carolina Population Center, Univeristy of North Carolina at Chapel Hill, Institute of Sociology, Russian Academy of Sciences
Ischemic Stroke	Russia Longitudinal Monitoring Survey (RLMS-HSE), Round XVII 2008. National Research University Higher School of Economics, ZAO Demoscope, Carolina Population Center, Univeristy of North Carolina at Chapel Hill, Institute of Sociology, Russian Academy of Sciences
Ischemic Stroke	Russia Longitudinal Monitoring Survey (RLMS-HSE), Round XVI 2007. National Research University Higher School of Economics, ZAO Demoscope, Carolina Population Center, Univeristy of North Carolina at Chapel Hill, Institute of Sociology, Russian Academy of Sciences
Ischemic Stroke	Russia Longitudinal Monitoring Survey (RLMS-HSE), Round XV 2006. National Research University Higher School of Economics, ZAO Demoscope, Carolina Population Center, Univeristy of North Carolina at Chapel Hill, Institute of Sociology, Russian Academy of Sciences
Ischemic Stroke	Russia Longitudinal Monitoring Survey (RLMS-HSE), Round VI 1995. National Research University Higher School of Economics, ZAO Demoscope, Carolina Population Center, Univeristy of North Carolina at Chapel Hill, Institute of Sociology, Russian Academy of Sciences
Ischemic Stroke	Russia Longitudinal Monitoring Survey (RLMS-HSE), Round V 1994. National Research University Higher School of Economics, ZAO Demoscope, Carolina Population Center, Univeristy of North Carolina at Chapel Hill, Institute of Sociology, Russian Academy of Sciences
Ischemic Stroke	Zahuranec DB, Brown DL, Lisabeth LD, Morgenstern LB. Is it time for a large, collaborative study of pediatric stroke?. Stroke. 2005; 36(9): 1825-9
Ischemic Stroke	Bonita R, Solomon N, Broad JB. Prevalence of Stroke and Stroke-Related Disability: Estimates From the Auckland Stroke Studies. Stroke. 1997; 28(10): 1898-902
Ischemic Stroke	Díaz-Guzmán J, Bermejo-Pareja F, Benito-León J, Vega S, Gabriel R, Medrano MJ. Prevalence of stroke and transient ischemic attack in three elderly populations of central Spain. Neuroepidemiology. 2008; 30(4): 247-53
Ischemic Stroke	Delbari A, Salman Roghani R, Tabatabaei SS, Rahgozar M, Lokk J. Stroke epidemiology and one-month fatality among an urban population in Iran. Int J Stroke. 2011; 6(3): 195-200
Ischemic Stroke	Delbari A, Salman Roghani R, Tabatabaei SS, Lökk J. A Stroke Study of an Urban Area of Iran: Risk Factors, Length of Stay, Case Fatality, and Discharge Destination. J Stroke Cerebrovasc Dis. 2010; 19(2): 104-9
Ischemic Stroke	Institute for Health Metrics and Evaluation (IHME), Ministry of Health (Saudi Arabia). Saudi Arabia Health Interview Survey 2013
Ischemic Stroke	Health and Retirement Study, (Biennial 2012) public use dataset. Produced and distributed by the University of Michigan with funding from the National Institute on Aging (grant number NIA U01AG009740). Ann Arbor, MI, (2015)
Ischemic Stroke	Ministry of Health, Social Services and Equality (Spain), Sociological Research Center (Spain). Spain Health Barometer Survey 2004. Madrid, Spain: Ministry of Health, Social Services and Equality (Spain), 2004
Ischemic Stroke	Vemmos KN, Bots ML, Tsibouris PK, Zis VP, Takis CE, Grobbee DE, Stamatelopoulos S. Prognosis of stroke in the south of Greece: 1 year mortality, functional outcome and its determinants: the Arcadia Stroke Registry. J Neurol Neurosurg Psychiatr. 2000; 69(5): 595-600
Ischemic Stroke	National Center for Health Statistics (NCHS), Centers for Disease Control and Prevention (CDC), United States Census Bureau. United States National Health Interview Survey 2013. Hyattsville, United States: National Center for Health Statistics (NCHS), Centers for Disease Control and Prevention (CDC), 2014
Ischemic Stroke	National Institute of Statistics and Censuses (Ecuador). Ecuador Hospital Inpatient Discharges 2013. Quito, Ecuador: National Institute of Statistics and Censuses (Ecuador)
Ischemic Stroke	National Center for Health Statistics (NCHS), Centers for Disease Control and Prevention (CDC). United States National Health and Nutrition Examination Survey 2013-2014. Hyattsville, United States: National Center for Health Statistics (NCHS), Centers for Disease Control and Prevention (CDC)

Ischemic Stroke	International Research Associates (INRA) Europe, World Health Organization (WHO). Spain WHO Multi-country Survey Study on Health and Health System Responsiveness 2000-2001. Geneva, Switzerland: World Health Organization (WHO)
Ischemic Stroke	University of Otago (New Zealand), World Health Organization (WHO). New Zealand WHO Multi-country Survey Study on Health and Health System Responsiveness 2000-2001. Geneva, Switzerland: World Health Organization (WHO)
Ischemic Stroke	International Research Associates (INRA) Europe, World Health Organization (WHO). Romania WHO Multi-country Survey Study on Health and Health System Responsiveness 2000-2001. Geneva, Switzerland: World Health Organization (WHO)
Ischemic Stroke	International Research Associates (INRA) Europe, World Health Organization (WHO). Russia WHO Multi-country Survey Study on Health and Health System Responsiveness 2000-2001. Geneva, Switzerland: World Health Organization (WHO)
Ischemic Stroke	Washington State University, World Health Organization (WHO). United States WHO Multi-country Survey Study on Health and Health System Responsiveness 2000-2001. Geneva, Switzerland: World Health Organization (WHO)
Ischemic Stroke	Börsch-Supan, A. (2015). Survey of Health, Ageing and Retirement in Europe (SHARE) Wave 5. Release version: 1.0.0. SHARE-ERIC. Data set. DOI: 10.6103/SHARE.w5.100
Ischemic Stroke	National Center for Health Statistics (NCHS), Centers for Disease Control and Prevention (CDC). United States National Health Interview Survey 2014. Hyattsville, United States: National Center for Health Statistics (NCHS), Centers for Disease Control and Prevention (CDC), 2015
Ischemic Stroke	Ministry of Health (New Zealand). New Zealand National Minimum Dataset 2000
Ischemic Stroke	Ministry of Health (New Zealand). New Zealand National Minimum Dataset 2001
Ischemic Stroke	Ministry of Health (New Zealand). New Zealand National Minimum Dataset 2002
Ischemic Stroke	Ministry of Health (New Zealand). New Zealand National Minimum Dataset 2003
Ischemic Stroke	Ministry of Health (New Zealand). New Zealand National Minimum Dataset 2004
Ischemic Stroke	Ministry of Health (New Zealand). New Zealand National Minimum Dataset 2005
Ischemic Stroke	Ministry of Health (New Zealand). New Zealand National Minimum Dataset 2006
Ischemic Stroke	Ministry of Health (New Zealand). New Zealand National Minimum Dataset 2007. Wellington, New Zealand: Ministry of Health (New Zealand)
Ischemic Stroke	Ministry of Health (New Zealand). New Zealand National Minimum Dataset 2008. Wellington, New Zealand: Ministry of Health (New Zealand)
Ischemic Stroke	Ministry of Health (New Zealand). New Zealand National Minimum Dataset 2009. Wellington, New Zealand: Ministry of Health (New Zealand)
Ischemic Stroke	Ministry of Health (New Zealand). New Zealand National Minimum Dataset 2010. Wellington, New Zealand: Ministry of Health (New Zealand)
Ischemic Stroke	Ministry of Health (New Zealand). New Zealand National Minimum Dataset 2011. Wellington, New Zealand: Ministry of Health (New Zealand)
Ischemic Stroke	Ministry of Health (New Zealand). New Zealand National Minimum Dataset 2012. Wellington, New Zealand: Ministry of Health (New Zealand)
Ischemic Stroke	Ministry of Health (New Zealand). New Zealand National Minimum Dataset 2013. Wellington, New Zealand: Ministry of Health (New Zealand)
Ischemic Stroke	Ministry of Health (New Zealand). New Zealand National Minimum Dataset 2014. Wellington, New Zealand: Ministry of Health (New Zealand)
Ischemic Stroke	National Statistical Office of Malawi. Malawi Integrated Household Survey 2013. Washington DC, United States: World Bank, 2015
Ischemic Stroke	Truven Health Analytics. United States MarketScan Commercial Claims and Encounters Database 2010. Ann Arbor, United States: Truven Health Analytics
Ischemic Stroke	Center for Sociological Studies, Lomonosov Moscow State University, Concluzia-Prim Center for Survey Methodology (Moldova), Institute for Advanced Studies (Austria), London School of Hygiene and Tropical Medicine, University of Aberdeen. Russia Health in Times of Transition Household Survey 2010
Ischemic Stroke	National Center for Health Statistics (NCHS), Centers for Disease Control and Prevention (CDC). United States National Hospital Discharge Survey 1988-1992
Ischemic Stroke	National Center for Health Statistics (NCHS), Centers for Disease Control and Prevention (CDC). United States National Hospital Discharge Survey 1993-1997
Ischemic Stroke	National Center for Health Statistics (NCHS), Centers for Disease Control and Prevention (CDC). United States National Hospital Discharge Survey 1998-2002
Ischemic Stroke	National Center for Health Statistics (NCHS), Centers for Disease Control and Prevention (CDC). United States National Hospital Discharge Survey 2003-2007
Ischemic Stroke	National Center for Health Statistics (NCHS), Centers for Disease Control and Prevention (CDC). United States National Hospital Discharge Survey 2008-2010
Ischemic Stroke	National Institute of Statistics and Censuses (Ecuador). Ecuador Hospital Inpatient Discharges 2014. Quito, Ecuador: National Institute of Statistics and Censuses (Ecuador)
Ischemic Stroke	Healthcare Cost and Utilization Project (HCUP), Agency for Healthcare Research and Quality (AHRQ). United States Nationwide Inpatient Sample 2013. Rockville, United States: Healthcare Cost and Utilization Project (HCUP), Agency for Healthcare Research and Quality (AHRQ)
Ischemic Stroke	National Center for Health Statistics (NCHS), Centers for Disease Control and Prevention (CDC). United States National Health Interview Survey 2015. Hyattsville, United States: National Center for Health Statistics (NCHS), Centers for Disease Control and Prevention (CDC), 2016
Ischemic Stroke	and Prevention. National Center for Health Statistics. National Health Interview Survey, 1994: Second Supplement on Aging. ICPSR02563-v3. Ann Arbor, MI: Inter-university Consortium for Political and Social Research [distributor], 2007-02-12. http://doi.org/10.3886/ICPSR02563.v3
Ischemic Stroke	Heikinheimo T, Chimbayo D, Kumwenda JJ, Kampondeni S, Allain TJ. Stroke outcomes in Malawi, a country with high prevalence of HIV: a prospective follow-up study. PLoS One. 2012; 7(3): e33765
Ischemic Stroke	Healthcare Cost and Utilization Project (HCUP), Agency for Healthcare Research and Quality (AHRQ). United States State Inpatient Databases 2003-2007
Ischemic Stroke	Healthcare Cost and Utilization Project (HCUP), Agency for Healthcare Research and Quality (AHRQ). United States State Inpatient Databases 2008-2009
Ischemic Stroke	Ministry of Health (New Zealand). New Zealand National Minimum Dataset 2000-2002
Ischemic Stroke	Ministry of Health (New Zealand). New Zealand National Minimum Dataset 2003-2007
Ischemic Stroke	Ministry of Health (New Zealand). New Zealand National Minimum Dataset 2008-2012
Ischemic Stroke	Ministry of Health (New Zealand). New Zealand National Minimum Dataset 2013-2014
Ischemic Stroke	Ministry of Health (New Zealand). New Zealand National Minimum Dataset 2015. Wellington, New Zealand: Ministry of Health (New Zealand)
Ischemic Stroke	Ministry of Health (Romania). Romania Hospital Inpatient Discharges 2000
Ischemic Stroke	Ministry of Health (Romania). Romania Hospital Inpatient Discharges 2001
Ischemic Stroke	Ministry of Health (Romania). Romania Hospital Inpatient Discharges 2002
Ischemic Stroke	Ministry of Health (Romania). Romania Hospital Inpatient Discharges 2003
Ischemic Stroke	Ministry of Health (Romania). Romania Hospital Inpatient Discharges 2004
Ischemic Stroke	Ministry of Health (Romania). Romania Hospital Inpatient Discharges 2005
Ischemic Stroke	National School of Public Health (Greece), World Health Organization (WHO). Greece WHO Multi-country Survey Study on Health and Health System Responsiveness 2000-2001
Ischemic Stroke	Healthcare Cost and Utilization Project (HCUP), Agency for Healthcare Research and Quality (AHRQ). United States Nationwide Inpatient Sample 2014. Rockville, United States: Healthcare Cost and Utilization Project (HCUP), Agency for Healthcare Research and Quality (AHRQ)

Ischemic Stroke	Healthcare Cost and Utilization Project (HCUP), Agency for Healthcare Research and Quality (AHRQ). United States State Inpatient Databases 2011. Rockville, United States: Healthcare Cost and Utilization Project (HCUP), Agency for Healthcare Research and Quality (AHRQ)
Ischemic Stroke	Healthcare Cost and Utilization Project (HCUP), Agency for Healthcare Research and Quality (AHRQ). United States State Inpatient Databases 2012. Rockville, United States: Healthcare Cost and Utilization Project (HCUP), Agency for Healthcare Research and Quality (AHRQ)
Ischemic Stroke	Healthcare Cost and Utilization Project (HCUP), Agency for Healthcare Research and Quality (AHRQ). United States State Inpatient Databases 2013. Rockville, United States: Healthcare Cost and Utilization Project (HCUP), Agency for Healthcare Research and Quality (AHRQ)
Ischemic Stroke	Healthcare Cost and Utilization Project (HCUP), Agency for Healthcare Research and Quality (AHRQ). United States State Inpatient Databases 2014. Rockville, United States: Healthcare Cost and Utilization Project (HCUP), Agency for Healthcare Research and Quality (AHRQ)
Ischemic Stroke	Healthcare Cost and Utilization Project (HCUP), Agency for Healthcare Research and Quality (AHRQ). United States Nationwide Inpatient Sample 2015. Rockville, United States: Healthcare Cost and Utilization Project (HCUP), Agency for Healthcare Research and Quality (AHRQ)
Ischemic Stroke	Ministry of Health (Romania). Romania Hospital Inpatient Discharges 1980
Ischemic Stroke	Ministry of Health and Social Security (Spain), National Statistics Institute (Spain). Spain Statistics on Health Establishments Providing Inpatient Care 1972
Ischemic Stroke	Ministry of Health and Social Security (Spain), National Statistics Institute (Spain). Spain Statistics on Health Establishments Providing Inpatient Care 1973
Ischemic Stroke	Ministry of Health and Social Security (Spain), National Statistics Institute (Spain). Spain Statistics on Health Establishments Providing Inpatient Care 1974
Ischemic Stroke	Ministry of Health and Social Security (Spain), National Statistics Institute (Spain). Spain Statistics on Health Establishments Providing Inpatient Care 1975
Ischemic Stroke	Ministry of Health and Social Security (Spain), National Statistics Institute (Spain). Spain Statistics on Health Establishments Providing Inpatient Care 1976
Ischemic Stroke	Ministry of Health and Social Security (Spain), National Statistics Institute (Spain). Spain Statistics on Health Establishments Providing Inpatient Care 1977
Ischemic Stroke	Ministry of Health and Social Security (Spain), National Statistics Institute (Spain). Spain Statistics on Health Establishments Providing Inpatient Care 1978
Ischemic Stroke	Ministry of Health and Social Security (Spain), National Statistics Institute (Spain). Spain Statistics on Health Establishments Providing Inpatient Care 1979
Ischemic Stroke	Ministry of Health and Social Security (Spain), National Statistics Institute (Spain). Spain Statistics on Health Establishments Providing Inpatient Care 1980
Ischemic Stroke	Ministry of Health and Social Security (Spain), National Statistics Institute (Spain). Spain Statistics on Health Establishments Providing Inpatient Care 1981
Ischemic Stroke	Ministry of Health and Consumer Affairs (Spain), National Statistics Institute (Spain). Spain Statistics on Health Establishments Providing Inpatient Care 1982
Ischemic Stroke	Ministry of Health and Consumer Affairs (Spain), National Statistics Institute (Spain). Spain Statistics on Health Establishments Providing Inpatient Care 1983
Ischemic Stroke	Ministry of Health and Consumer Affairs (Spain), National Statistics Institute (Spain). Spain Statistics on Health Establishments Providing Inpatient Care 1984
Ischemic Stroke	Ministry of Health and Consumer Affairs (Spain), National Statistics Institute (Spain). Spain Statistics on Health Establishments Providing Inpatient Care 1985
Ischemic Stroke	Ministry of Health and Consumer Affairs (Spain), National Statistics Institute (Spain). Spain Statistics on Health Establishments Providing Inpatient Care 1986
Ischemic Stroke	Ministry of Health and Consumer Affairs (Spain), National Statistics Institute (Spain). Spain Statistics on Health Establishments Providing Inpatient Care 1987
Ischemic Stroke	Ministry of Health and Consumer Affairs (Spain), National Statistics Institute (Spain). Spain Statistics on Health Establishments Providing Inpatient Care 1988
Ischemic Stroke	Ministry of Health and Consumer Affairs (Spain), National Statistics Institute (Spain). Spain Statistics on Health Establishments Providing Inpatient Care 1989
Ischemic Stroke	Ministry of Health and Consumer Affairs (Spain), National Statistics Institute (Spain). Spain Statistics on Health Establishments Providing Inpatient Care 1990
Ischemic Stroke	Ministry of Health and Consumer Affairs (Spain), National Statistics Institute (Spain). Spain Statistics on Health Establishments Providing Inpatient Care 1991
Ischemic Stroke	Ministry of Health and Consumer Affairs (Spain), National Statistics Institute (Spain). Spain Statistics on Health Establishments Providing Inpatient Care 1992
Ischemic Stroke	Ministry of Health and Consumer Affairs (Spain), National Statistics Institute (Spain). Spain Statistics on Health Establishments Providing Inpatient Care 1993
Ischemic Stroke	Ministry of Health and Consumer Affairs (Spain), National Statistics Institute (Spain). Spain Statistics on Health Establishments Providing Inpatient Care 1994
Ischemic Stroke	Ministry of Health and Consumer Affairs (Spain), National Statistics Institute (Spain). Spain Statistics on Health Establishments Providing Inpatient Care 1995
Ischemic Stroke	Ministry of Health and Consumer Affairs (Spain), National Statistics Institute (Spain). Spain Statistics on Health Establishments Providing Inpatient Care 1996
Ischemic Stroke	Ministry of Health and Consumer Affairs (Spain). Spain Statistics on Health Establishments Providing Inpatient Care 1997. Madrid, Spain: Ministry of Health, Social Services and Equality (Spain)
Ischemic Stroke	Ministry of Health and Consumer Affairs (Spain). Spain Statistics on Health Establishments Providing Inpatient Care 1998. Madrid, Spain: Ministry of Health, Social Services and Equality (Spain)
Ischemic Stroke	Ministry of Health and Consumer Affairs (Spain). Spain Statistics on Health Establishments Providing Inpatient Care 1999. Madrid, Spain: Ministry of Health, Social Services and Equality (Spain)
Ischemic Stroke	Ministry of Health and Consumer Affairs (Spain). Spain Statistics on Health Establishments Providing Inpatient Care 2000. Madrid, Spain: Ministry of Health, Social Services and Equality (Spain)
Ischemic Stroke	Ministry of Health and Consumer Affairs (Spain). Spain Statistics on Health Establishments Providing Inpatient Care 2001. Madrid, Spain: Ministry of Health, Social Services and Equality (Spain)
Ischemic Stroke	Ministry of Health and Consumer Affairs (Spain). Spain Statistics on Health Establishments Providing Inpatient Care 2002. Madrid, Spain: Ministry of Health, Social Services and Equality (Spain)
Ischemic Stroke	Ministry of Health and Consumer Affairs (Spain). Spain Statistics on Health Establishments Providing Inpatient Care 2003. Madrid, Spain: Ministry of Health, Social Services and Equality (Spain)

Ischemic Stroke	Ministry of Health and Consumer Affairs (Spain). Spain Statistics on Health Establishments Providing Inpatient Care 2004. Madrid, Spain: Ministry of Health, Social Services and Equality (Spain)
Ischemic Stroke	Ministry of Health and Consumer Affairs (Spain). Spain Statistics on Health Establishments Providing Inpatient Care 2005. Madrid, Spain: Ministry of Health, Social Services and Equality (Spain)
Ischemic Stroke	Ministry of Health and Consumer Affairs (Spain). Spain Statistics on Health Establishments Providing Inpatient Care 2006. Madrid, Spain: Ministry of Health, Social Services and Equality (Spain)
Ischemic Stroke	Ministry of Health and Consumer Affairs (Spain). Spain Statistics on Health Establishments Providing Inpatient Care 2007. Madrid, Spain: Ministry of Health, Social Services and Equality (Spain)
Ischemic Stroke	Ministry of Health and Consumer Affairs (Spain). Spain Statistics on Health Establishments Providing Inpatient Care 2008. Madrid, Spain: Ministry of Health, Social Services and Equality (Spain)
Ischemic Stroke	Ministry of Health and Consumer Affairs (Spain). Spain Statistics on Health Establishments Providing Inpatient Care 2009. Madrid, Spain: Ministry of Health, Social Services and Equality (Spain)
Ischemic Stroke	Ministry of Health, Social Services and Equality (Spain). Spain Statistics on Specialized Healthcare Centers 2010. Madrid, Spain: Ministry of Health, Social Services and Equality (Spain)
Ischemic Stroke	Ministry of Health, Social Services and Equality (Spain). Spain Statistics on Specialized Healthcare Centers 2011. Madrid, Spain: Ministry of Health, Social Services and Equality (Spain)
Ischemic Stroke	Ministry of Health, Social Services and Equality (Spain). Spain Statistics on Specialized Healthcare Centers 2012. Madrid, Spain: Ministry of Health, Social Services and Equality (Spain)
Ischemic Stroke	Ministry of Health, Social Services and Equality (Spain). Spain Statistics on Specialized Healthcare Centers 2013. Madrid, Spain: Ministry of Health, Social Services and Equality (Spain)
Ischemic Stroke	Ministry of Health, Social Services and Equality (Spain). Spain Statistics on Specialized Healthcare Centers 2014. Madrid, Spain: Ministry of Health, Social Services and Equality (Spain)
Ischemic Stroke	Ministry of Health (Romania). Romania Hospital Inpatient Discharges 1985
Ischemic Stroke	Ministry of Health (Romania). Romania Hospital Inpatient Discharges 1986
Ischemic Stroke	Ministry of Health (Romania). Romania Hospital Inpatient Discharges 1987
Ischemic Stroke	Ministry of Health (Romania). Romania Hospital Inpatient Discharges 1988
Ischemic Stroke	Ministry of Health (Romania). Romania Hospital Inpatient Discharges 1989
Ischemic Stroke	Ministry of Health (Romania). Romania Hospital Inpatient Discharges 1990
Ischemic Stroke	Ministry of Health (Romania). Romania Hospital Inpatient Discharges 1991
Ischemic Stroke	Ministry of Health (Romania). Romania Hospital Inpatient Discharges 1992
Ischemic Stroke	Ministry of Health (Romania). Romania Hospital Inpatient Discharges 1993
Ischemic Stroke	Ministry of Health (Romania). Romania Hospital Inpatient Discharges 1994
Ischemic Stroke	Ministry of Health (Romania). Romania Hospital Inpatient Discharges 1995
Ischemic Stroke	Ministry of Health (Romania). Romania Hospital Inpatient Discharges 1996
Ischemic Stroke	Ministry of Health (Romania). Romania Hospital Inpatient Discharges 1997
Ischemic Stroke	Ministry of Health (Romania). Romania Hospital Inpatient Discharges 1998
Ischemic Stroke	Ministry of Health (Romania). Romania Hospital Inpatient Discharges 1999
Ischemic Stroke	Ministry of Health (Romania). Romania Hospital Inpatient Discharges 2013
Ischemic Stroke	Bureau of Statistics (Guyana), World Bank. Guyana Living Standards Measurement Survey 1992-1993
Ischemic Stroke	Ministry of Statistics and Programme Implementation (India). India National Sample Survey Round 52 1995-1996. New Delhi, India: Ministry of Statistics and Programme Implementation (India)
Ischemic Stroke	Ministry of Statistics and Programme Implementation (India). India National Sample Survey Round 60 2004. New Delhi, India: Ministry of Statistics and Programme Implementation (India)
Ischemic Stroke	National Institute of Statistics and Informatics (Peru), World Bank (WB). Peru Living Standards Measurement Survey 1990
Ischemic Stroke	National Institute of Statistics and Informatics (INEI) (Peru), World Bank. Peru Living Standards Measurement Survey 1991. Washington DC, United States: World Bank
Ischemic Stroke	National Institute of Statistics and Informatics (Peru), World Bank (WB). Peru Living Standards Measurement Survey 1994
Ischemic Stroke	Ministry of Social Affairs (Serbia), World Bank. Yugoslavia, Federal Republic - Serbia Living Standards Measurement Survey 2002. Washington DC, United States: World Bank
Ischemic Stroke	Ministry of Social Affairs (Serbia), World Bank. Serbia and Montenegro - Serbia Living Standards Measurement Survey 2003. Washington DC, United States: World Bank
Ischemic Stroke	International Institute for Population Sciences (India), World Health Organization (WHO). India World Health Survey 2003. Geneva, Switzerland: World Health Organization (WHO), 2005
Ischemic Stroke	World Health Organization (WHO). Malaysia World Health Survey 2003. Geneva, Switzerland: World Health Organization (WHO), 2005
Ischemic Stroke	World Health Organization (WHO). Mauritius World Health Survey 2003. Geneva, Switzerland: World Health Organization (WHO), 2005
Ischemic Stroke	World Health Organization (WHO). Sweden World Health Survey 2003. Geneva, Switzerland: World Health Organization (WHO), 2005
Ischemic Stroke	World Health Organization (WHO). Ukraine World Health Survey 2002-2003. Geneva, Switzerland: World Health Organization (WHO), 2005
Ischemic Stroke	National Institute of Statistics and Informatics (INEI) (Peru), United Nations Economic Commission for Latin America and the Caribbean (CEPAL), Institute of Research for Development (France). Peru National Household Survey 2007. Lima, Peru: National Institute of Statistics and Informatics (INEI) (Peru)
Ischemic Stroke	National Institute of Statistics and Informatics (INEI) (Peru), United Nations Economic Commission for Latin America and the Caribbean (CEPAL), Institute of Research for Development (France). Peru National Household Survey 2008. Lima, Peru: National Institute of Statistics and Informatics (INEI) (Peru)
Ischemic Stroke	National Institute of Statistics and Informatics (INEI) (Peru). Peru National Household Survey 2009. Lima, Peru: National Institute of Statistics and Informatics (INEI) (Peru)
Ischemic Stroke	National Institute of Statistics and Informatics (INEI) (Peru). Peru National Household Survey 2010. Lima, Peru: National Institute of Statistics and Informatics (INEI) (Peru)
Ischemic Stroke	National Institute of Statistics and Informatics (INEI) (Peru). Peru National Household Survey, Second Quarter 1998. Lima, Peru: National Institute of Statistics and Informatics (INEI) (Peru)
Ischemic Stroke	National Institute of Statistics and Informatics (INEI) (Peru). Peru National Household Survey, Second Quarter 1999. Lima, Peru: National Institute of Statistics and Informatics (INEI) (Peru)
Ischemic Stroke	National Institute of Statistics (Albania), World Bank (WB). Albania Living Standards Measurement Survey 2002. Washington DC, United States: World Bank (WB)
Ischemic Stroke	National Institute of Statistics (Albania), World Bank (WB). Albania Living Standards Measurement Survey 2005. Washington DC, United States: World Bank (WB)

Ischemic Stroke	National Institute of Statistics and Informatics (INEI) (Peru). Peru National Household Survey, Second Quarter 2000. Lima, Peru: National Institute of Statistics and Informatics (INEI) (Peru)
Ischemic Stroke	Ministry of Social Affairs (Serbia), World Bank. Serbia Living Standards Measurement Survey 2007. Washington DC, United States: World Bank
Ischemic Stroke	National Institute of Statistics and Informatics (INEI) (Peru). Peru National Household Survey 2005. Lima, Peru: National Institute of Statistics and Informatics (INEI) (Peru)
Ischemic Stroke	National Institute of Statistics and Informatics (INEI) (Peru). Peru National Household Survey 2006. Lima, Peru: National Institute of Statistics and Informatics (INEI) (Peru)
Ischemic Stroke	International Institute for Population Sciences (India), World Health Organization (WHO). India WHO Study on Global Ageing and Adult Health 2007. Geneva, Switzerland: World Health Organization (WHO), 2007
Ischemic Stroke	Ministry of Health (Syria), World Health Organization (WHO). Syria WHO Multi-country Survey Study on Health and Health System Responsiveness 2000-2001
Ischemic Stroke	National Institute of Statistics (Niger), World Bank. Niger National Survey on Household Living Conditions and Agriculture 2011-2012
Ischemic Stroke	Sridharan SE, Unnikrishnan JP, Sukumaran S, Sylaja PN, Nayak SD, Sarma PS, Radhakrishnan K. Incidence, Types, Risk Factors, and Outcome of Stroke in a Developing Country. Stroke. 2009; 40(4): 1212-8
Ischemic Stroke	Appelros P, Nydevik I, Seiger Åke, Terént A. High Incidence Rates of Stroke in Orebro, Sweden: Further Support for Regional Incidence Differences within Scandinavia. Cerebrovasc Dis. 2002; 14(3-4): 161-8
Ischemic Stroke	Hallström B, Jönsson A-C, Nerbrand C, Norrving B, Lindgren A. Stroke Incidence and Survival in the Beginning of the 21st Century in Southern Sweden: Comparisons With the Late 20th Century and Projections Into the Future. Stroke. 2008; 39(1): 10-5
Ischemic Stroke	Dalal PM, Malik S, Bhattacharjee M, Trivedi ND, Vairale J, Bhat P, Deshmukh S, Khandelwal K, Mathur VD. Population-based stroke survey in Mumbai, India: incidence and 28-day case fatality. Neuroepidemiology. 2008; 31(4): 254-61
Ischemic Stroke	Mihálka L, Smolanka V, Bulecza B, Mulesa S, Bereczki D. A Population Study of Stroke in West Ukraine: Incidence, Stroke Services, and 30-Day Case Fatality. Stroke. 2001; 32(10): 2227-31
Ischemic Stroke	Terént A. Trends in Stroke Incidence and 10-Year Survival in Söderhamn, Sweden, 1975-2001. Stroke. 2003; 34(6): 1353-8
Ischemic Stroke	Statistics Sweden, Swedish National Institute of Public Health. Sweden National Survey of Public Health 2009
Ischemic Stroke	Statistics Sweden, Swedish National Institute of Public Health. Sweden National Survey of Public Health 2007
Ischemic Stroke	Institute for Public Health, Ministry of Health (Malaysia). Malaysia National Health And Morbidity Survey 2006. Kuala Lumpur, Malaysia: Institute for Public Health, Ministry of Health (Malaysia)
Ischemic Stroke	Northern Ireland Statistics and Research Agency. Central Survey Unit, Northern Ireland Health and Social Wellbeing Survey, 2001 [computer file]. Colchester, Essex: UK Data Archive [distributor], October 2002. SN: 4590 , http://dx.doi.org/10.5255/UKDA-SN-4590-1
Ischemic Stroke	Northern Ireland Statistics and Research Agency. Central Survey Unit, Northern Ireland Health and Social Wellbeing Survey, 2005-2006 [computer file]. Colchester, Essex: UK Data Archive [distributor], October 2007. SN: 5710, http://dx.doi.org/10.5255/UKDA-SN-5710-1
Ischemic Stroke	Northern Ireland Statistics and Research Agency. Central Survey Unit, Northern Ireland Health and Social Wellbeing Survey, 1997 [computer file]. Colchester, Essex: UK Data Archive [distributor], October 2002. SN: 4589, http://dx.doi.org/10.5255/UKDA-SN-4589-1
Ischemic Stroke	Northern Ireland Statistics and Research Agency (NISRA). United Kingdom - Northern Ireland Health Survey 2010-2011 - UK Data Service
Ischemic Stroke	Das SK, Banerjee TK, Biswas A, Roy T, Raut DK, Mukherjee CS, Chaudhuri A, Hazra A, Roy J. A Prospective Community-Based Study of Stroke in Kolkata, India. Stroke. 2007; 38(3): 906-10
Ischemic Stroke	Banerjee TK, Mukherjee CS, Sarkhel A. Stroke in the urban population of Calcutta – an epidemiological study. Neuroepidemiology. 2001; 20(3): 201-7
Ischemic Stroke	Das S, Sanyal K, Moitra A. A pilot study on neuroepidemiology in urban Bengal. Indian J Public Health. 1998; 42(2): 34-6
Ischemic Stroke	Dhamija RK, Dhamija SB. Prevalence of stroke in rural community – an overview of Indian experience. J Assoc Physicians India. 1998; 46(4): 351-4
Ischemic Stroke	Koul R, Motta A, Razdan S. Epidemiology of young strokes in rural Kashmir, India. Acta Neurol Scand. 1990; 82(1): 1-3
Ischemic Stroke	Saha SP, Bhattacharya S, Das SK, Maity B, Roy T, Raut DK. Epidemiological study of neurological disorders in a rural population of Eastern India. J Indian Med Assoc. 2003; 101(5): 299-304
Ischemic Stroke	National Board of Health and Welfare (Sweden). Sweden National Patient Register 1998. Stockholm, Sweden: National Board of Health and Welfare (Sweden)
Ischemic Stroke	National Board of Health and Welfare (Sweden). Sweden National Patient Register 1999. Stockholm, Sweden: National Board of Health and Welfare (Sweden)
Ischemic Stroke	National Board of Health and Welfare (Sweden). Sweden National Patient Register 2011. Stockholm, Sweden: National Board of Health and Welfare (Sweden)
Ischemic Stroke	National Board of Health and Welfare (Sweden). Sweden National Patient Register 2012. Stockholm, Sweden: National Board of Health and Welfare (Sweden)
Ischemic Stroke	Statistics Sweden, Swedish National Institute of Public Health. Sweden National Survey of Public Health 2008
Ischemic Stroke	Hilmarsson A, Kjartansson O, Olafsson E. Incidence of First Stroke A Population Study in Iceland. Stroke. 2013; 44(6): 1714-6
Ischemic Stroke	Pilot Study, 2005 (Data Set 27-28, Cunningham, Shayna.) [machine-readable data file and documentation]. Geneva, Switzerland: World Health Organization (Producer). Los Altos, CA: Sociometrics Corporation, Data Archive of Social Research on Aging (Producer & Distributor)
Ischemic Stroke	Statistics Sweden, Swedish National Institute of Public Health. Sweden National Survey of Public Health 2012
Ischemic Stroke	Statistics Sweden, Swedish National Institute of Public Health. Sweden National Survey of Public Health 2010
Ischemic Stroke	Statistics Sweden, Swedish National Institute of Public Health. Sweden National Survey of Public Health 2004
Ischemic Stroke	Statistics Sweden, Swedish National Institute of Public Health. Sweden National Survey of Public Health 2005
Ischemic Stroke	Statistics Sweden, Swedish National Institute of Public Health. Sweden National Survey of Public Health 2011
Ischemic Stroke	Lindmark A, Glader E-L, Asplund K, Norrving B, Eriksson M, Riks-Stroke Collaboration. Socioeconomic disparities in stroke case fatality--Observations from Riks-Stroke, the Swedish stroke register. Int J Stroke. 2014; 9(4): 429–36
Ischemic Stroke	Trinity College Dublin. Ireland Longitudinal Study on Ageing 2009-2011. Dublin, Ireland: Irish Social Science Data Archive, University College Dublin
Ischemic Stroke	Statistics Sweden, Swedish National Institute of Public Health. Sweden National Survey of Public Health 2013
Ischemic Stroke	Public Health Agency of Sweden, Statistics Sweden. Sweden National Survey of Public Health 2014
Ischemic Stroke	International Research Associates (INRA) Europe, World Health Organization (WHO). Iceland WHO Multi-country Survey Study on Health and Health System Responsiveness 2000-2001. Geneva, Switzerland: World Health Organization (WHO)
Ischemic Stroke	International Research Associates (INRA) Europe, World Health Organization (WHO). Sweden WHO Multi-country Survey Study on Health and Health System Responsiveness 2000-2001. Geneva, Switzerland: World Health Organization (WHO)
Ischemic Stroke	Kiev International Institute of Sociology, World Health Organization (WHO). Ukraine WHO Multi-country Survey Study on Health and Health System Responsiveness 2000-2001. Geneva, Switzerland: World Health Organization (WHO)
Ischemic Stroke	Gallup Europe, World Health Organization (WHO). Venezuela WHO Multi-country Survey Study on Health and Health System Responsiveness 2000-2001. Geneva, Switzerland: World Health Organization (WHO)
Ischemic Stroke	Börsch-Supan, A. (2013). Survey of Health, Ageing and Retirement in Europe (SHARE) Wave 2. Release version: 2.6.0. SHARE-ERIC. Data set. DOI: 10.6103/SHARE.w2.260

Ischemic Stroke	Börsch-Supan, A. (2013). Survey of Health, Ageing and Retirement in Europe (SHARE) Wave 4. Release version: 1.1.1. SHARE-ERIC. Data set. DOI: 10.6103/SHARE.w4.111
Ischemic Stroke	Börsch-Supan, A. (2015). Survey of Health, Ageing and Retirement in Europe (SHARE) Wave 5. Release version: 1.0.0. SHARE-ERIC. Data set. DOI: 10.6103/SHARE.w5.100
Ischemic Stroke	Government of India, Ministry of Statistics and Programme Implementation (India). India National Sample Survey Round 71 2014. New Delhi, India: Ministry of Statistics and Programme Implementation (India)
Ischemic Stroke	Armenian Sociological Association, Concluzia-Prim Center for Survey Methodology (Moldova), Institute for Advanced Studies (Austria), London School of Hygiene and Tropical Medicine, University of Aberdeen. Armenia Health in Times of Transition Household Survey 2010
Ischemic Stroke	Zhi X, Joas E, Waern M, Östling S, Börjesson-Hanson A, Skoog I. Prevalence of cardiovascular disorders and risk factors in two 75-year-old birth cohorts examined in 1976-1977 and 2005-2006. Aging Clin Exp Res. 2013; 25(4): 377-83
Ischemic Stroke	Concluzia-Prim Center for Survey Methodology (Moldova), East-Ukranian Foundation For Social Research, Institute for Advanced Studies (Austria), London School of Hygiene and Tropical Medicine, University of Aberdeen. Ukraine Health in Times of Transition Household Survey 2010
Ischemic Stroke	National Board of Health and Welfare (Sweden). Sweden National Patient Register 1998-2002
Ischemic Stroke	National Board of Health and Welfare (Sweden). Sweden National Patient Register 2003-2007
Ischemic Stroke	National Board of Health and Welfare (Sweden). Sweden National Patient Register 2008-2012
Ischemic Stroke	All India Institute of Medical Sciences, New Delhi (AIIMS), Bangur Institute of Neurology (India), Indian Statistical Institute, National Neurosciences Center, Calcutta (India). India - Kolkata Study for Epidemiology of Neurological Disorders 2003-2004
Ischemic Stroke	Institute for Public Health, Ministry of Health (Malaysia). Malaysia National Health and Morbidity Survey 2011
Ischemic Stroke	Unnikrishnan JP, Sylaja S, Nayak SD, Radhakrishnan K. India - Trivandrum Stroke Registry 2005. [Unpublished]
Ischemic Stroke	St. John's National Academy of Health Sciences. INdian Stroke ProspectIve REgistry (INSPIRE) Data 2009-2014
Ischemic Stroke	Kalkonde YV, Sahane V, Deshmukh MD, Nila S, Mandava P, Bang A. High Prevalence of Stroke in Rural Gadchiroli, India: A Community-Based Study. Neuroepidemiology. 2016; 46(4): 235-9
Ischemic Stroke	Moodbidri P, Mehmi G, Sharma A, Arora OP, Dhanuka AK, Sobti MK, Sehgal H, Kaur M, Grewal SS, Jhawar SS, Shadangi TN, Arora T, Saxena A, Sachdeva G, Gill JS, Brar RS, Gill A, Bakshi SS, Pawar SS, Singh G, Sikka P, Litoria PK, Sharma M. Incidence, short-term outcome, and spatial distribution of stroke patients in Ludhiana, India.
Ischemic Stroke	Kulshrestha M, Vidyanand. An analysis of the risk factors and the outcomes of cerebrovascular diseases in northern India. J Clin Diagn Res. 2013; 7(1): 127–31
Ischemic Stroke	Appelros P, Terent A. Characteristics of the National Institute of Health Stroke Scale: results from a population-based stroke cohort at baseline and after one year. Cerebrovasc Dis. 2004; 17(1): 21–7
Ischemic Stroke	Zhu L, Fratiglioni L, Guo Z, Aguero-Torres H, Winblad B, Viitanen M. Association of stroke with dementia, cognitive impairment, and functional disability in the very old: a population-based study. Stroke. 1998; 29(10): 2094–9
Ischemic Stroke	Hornsten C, Molander L, Gustafson Y. The prevalence of stroke and the association between stroke and depression among a very old population. Arch Gerontol Geriatr. 2012; 55(3): 555–9
Ischemic Stroke	Institute of Public Health of Serbia. Serbia National Hospital Discharge Database 2012
Ischemic Stroke	Department of Health, Social Services and Public Safety (Northern Ireland), Information Centre for Health and Social Care, NHS, NHS England, NHS Health Scotland, NHS Wales. United Kingdom Hospital Patient and Discharge Data 2006
Ischemic Stroke	Directorate of Health (Iceland). Iceland Hospital Data Registry 2008
Ischemic Stroke	Ministry of Health (Albania). Albania Inpatient Care Discharges per 100 1993
Ischemic Stroke	Ministry of Health (Albania). Albania Inpatient Care Discharges per 100 1994
Ischemic Stroke	Ministry of Health (Albania). Albania Inpatient Care Discharges per 100 1995
Ischemic Stroke	Ministry of Health (Albania). Albania Inpatient Care Discharges per 100 1996
Ischemic Stroke	Ministry of Health (Albania). Albania Inpatient Care Discharges per 100 1997
Ischemic Stroke	Ministry of Health (Albania). Albania Inpatient Care Discharges per 100 1998
Ischemic Stroke	Ministry of Health (Albania). Albania Inpatient Care Discharges per 100 1999
Ischemic Stroke	Ministry of Health (Albania). Albania Inpatient Care Discharges per 100 2000
Ischemic Stroke	Ministry of Health (Albania). Albania Inpatient Care Discharges per 100 2001
Ischemic Stroke	Ministry of Health (Albania). Albania Inpatient Care Discharges per 100 2002
Ischemic Stroke	Ministry of Health (Albania). Albania Inpatient Care Discharges per 100 2003
Ischemic Stroke	Ministry of Health (Albania). Albania Inpatient Care Discharges per 100 2004
Ischemic Stroke	Ministry of Health (Albania). Albania Inpatient Care Discharges per 100 2005
Ischemic Stroke	Ministry of Health (Albania). Albania Inpatient Care Discharges per 100 2006
Ischemic Stroke	Ministry of Health (Albania). Albania Inpatient Care Discharges per 100 2007
Ischemic Stroke	Ministry of Health (Albania). Albania Inpatient Care Discharges per 100 2008
Ischemic Stroke	Ministry of Health (Albania). Albania Inpatient Care Discharges per 100 2009
Ischemic Stroke	Ministry of Health (Albania). Albania Inpatient Care Discharges per 100 2010
Ischemic Stroke	Ministry of Health (Albania). Albania Inpatient Care Discharges per 100 2011
Ischemic Stroke	Ministry of Health (Albania). Albania Inpatient Care Discharges per 100 2012
Ischemic Stroke	Ministry of Health (Albania). Albania Inpatient Care Discharges per 100 2013
Ischemic Stroke	Directorate of Health (Iceland). Iceland Hospital Data Registry 2010
Ischemic Stroke	Directorate of Health (Iceland). Iceland Hospital Data Registry 2011
Ischemic Stroke	Directorate of Health (Iceland). Iceland Hospital Data Registry 2012
Ischemic Stroke	Directorate of Health (Iceland). Iceland Hospital Data Registry 2013
Ischemic Stroke	Directorate of Health (Iceland). Iceland Hospital Data Registry 2014
Ischemic Stroke	Directorate of Health (Iceland). Iceland Hospital Data Registry 1999
Ischemic Stroke	Directorate of Health (Iceland). Iceland Hospital Data Registry 1995
Ischemic Stroke	Directorate of Health (Iceland). Iceland Hospital Data Registry 1994
Ischemic Stroke	Directorate of Health (Iceland). Iceland Hospital Data Registry 1993
Ischemic Stroke	Directorate of Health (Iceland). Iceland Hospital Data Registry 1992
Ischemic Stroke	Directorate of Health (Iceland). Iceland Hospital Data Registry 1991
Ischemic Stroke	Directorate of Health (Iceland). Iceland Hospital Data Registry 1990
Ischemic Stroke	Directorate of Health (Iceland). Iceland Hospital Data Registry 1989
Ischemic Stroke	Directorate of Health (Iceland). Iceland Hospital Data Registry 1988
Ischemic Stroke	Ministry of Health (Armenia). Armenia Inpatient Care Discharges per 100 1980
Ischemic Stroke	Ministry of Health (Armenia). Armenia Inpatient Care Discharges per 100 1981
Ischemic Stroke	Ministry of Health (Armenia). Armenia Inpatient Care Discharges per 100 1982

Ischemic Stroke	Ministry of Health (Armenia). Armenia Inpatient Care Discharges per 100 1983
Ischemic Stroke	Ministry of Health (Armenia). Armenia Inpatient Care Discharges per 100 1984
Ischemic Stroke	Ministry of Health (Armenia). Armenia Inpatient Care Discharges per 100 1985
Ischemic Stroke	Ministry of Health (Armenia). Armenia Inpatient Care Discharges per 100 1986
Ischemic Stroke	Ministry of Health (Armenia). Armenia Inpatient Care Discharges per 100 1987
Ischemic Stroke	Ministry of Health (Armenia). Armenia Inpatient Care Discharges per 100 1988
Ischemic Stroke	Ministry of Health (Armenia). Armenia Inpatient Care Discharges per 100 1989
Ischemic Stroke	Ministry of Health (Armenia). Armenia Inpatient Care Discharges per 100 1990
Ischemic Stroke	Ministry of Health (Armenia). Armenia Inpatient Care Discharges per 100 1991
Ischemic Stroke	Ministry of Health (Armenia). Armenia Inpatient Care Discharges per 100 1992
Ischemic Stroke	Ministry of Health (Armenia). Armenia Inpatient Care Discharges per 100 1993
Ischemic Stroke	Ministry of Health (Armenia). Armenia Inpatient Care Discharges per 100 1994
Ischemic Stroke	Ministry of Health (Armenia). Armenia Inpatient Care Discharges per 100 1995
Ischemic Stroke	Ministry of Health (Armenia). Armenia Inpatient Care Discharges per 100 1996
Ischemic Stroke	Ministry of Health (Armenia). Armenia Inpatient Care Discharges per 100 1997
Ischemic Stroke	Ministry of Health (Armenia). Armenia Inpatient Care Discharges per 100 1998
Ischemic Stroke	Ministry of Health (Armenia). Armenia Inpatient Care Discharges per 100 1999
Ischemic Stroke	Ministry of Health (Armenia). Armenia Inpatient Care Discharges per 100 2000
Ischemic Stroke	Ministry of Health (Armenia). Armenia Inpatient Care Discharges per 100 2001
Ischemic Stroke	Ministry of Health (Armenia). Armenia Inpatient Care Discharges per 100 2002
Ischemic Stroke	Ministry of Health (Armenia). Armenia Inpatient Care Discharges per 100 2003
Ischemic Stroke	Ministry of Health (Armenia). Armenia Inpatient Care Discharges per 100 2004
Ischemic Stroke	Ministry of Health (Armenia). Armenia Inpatient Care Discharges per 100 2005
Ischemic Stroke	Ministry of Health (Armenia). Armenia Inpatient Care Discharges per 100 2006
Ischemic Stroke	Ministry of Health (Armenia). Armenia Inpatient Care Discharges per 100 2007
Ischemic Stroke	Ministry of Health (Armenia). Armenia Inpatient Care Discharges per 100 2008
Ischemic Stroke	Ministry of Health (Armenia). Armenia Inpatient Care Discharges per 100 2009
Ischemic Stroke	Ministry of Health (Armenia). Armenia Inpatient Care Discharges per 100 2010
Ischemic Stroke	Ministry of Health (Armenia). Armenia Inpatient Care Discharges per 100 2011
Ischemic Stroke	Ministry of Health (Armenia). Armenia Inpatient Care Discharges per 100 2012
Ischemic Stroke	Ministry of Health (Armenia). Armenia Inpatient Care Discharges per 100 2013
Ischemic Stroke	Ministry of Health (Armenia). Armenia Inpatient Care Discharges per 100 2014
Ischemic Stroke	Center for Health Statistics, Ministry of Health (Ukraine). Ukraine Inpatient Care Discharges per 100 1980
Ischemic Stroke	Center for Health Statistics, Ministry of Health (Ukraine). Ukraine Inpatient Care Discharges per 100 1981
Ischemic Stroke	Center for Health Statistics, Ministry of Health (Ukraine). Ukraine Inpatient Care Discharges per 100 1982
Ischemic Stroke	Center for Health Statistics, Ministry of Health (Ukraine). Ukraine Inpatient Care Discharges per 100 1983
Ischemic Stroke	Center for Health Statistics, Ministry of Health (Ukraine). Ukraine Inpatient Care Discharges per 100 1984
Ischemic Stroke	Center for Health Statistics, Ministry of Health (Ukraine). Ukraine Inpatient Care Discharges per 100 1985
Ischemic Stroke	Center for Health Statistics, Ministry of Health (Ukraine). Ukraine Inpatient Care Discharges per 100 1986
Ischemic Stroke	Center for Health Statistics, Ministry of Health (Ukraine). Ukraine Inpatient Care Discharges per 100 1987
Ischemic Stroke	Center for Health Statistics, Ministry of Health (Ukraine). Ukraine Inpatient Care Discharges per 100 1988
Ischemic Stroke	Center for Health Statistics, Ministry of Health (Ukraine). Ukraine Inpatient Care Discharges per 100 1989
Ischemic Stroke	Center for Health Statistics, Ministry of Health (Ukraine). Ukraine Inpatient Care Discharges per 100 1990
Ischemic Stroke	Center for Health Statistics, Ministry of Health (Ukraine). Ukraine Inpatient Care Discharges per 100 1991
Ischemic Stroke	Center for Health Statistics, Ministry of Health (Ukraine). Ukraine Inpatient Care Discharges per 100 1992
Ischemic Stroke	Center for Health Statistics, Ministry of Health (Ukraine). Ukraine Inpatient Care Discharges per 100 1993
Ischemic Stroke	Center for Health Statistics, Ministry of Health (Ukraine). Ukraine Inpatient Care Discharges per 100 1994
Ischemic Stroke	Center for Health Statistics, Ministry of Health (Ukraine). Ukraine Inpatient Care Discharges per 100 1995
Ischemic Stroke	Center for Health Statistics, Ministry of Health (Ukraine). Ukraine Inpatient Care Discharges per 100 1996
Ischemic Stroke	Center for Health Statistics, Ministry of Health (Ukraine). Ukraine Inpatient Care Discharges per 100 1997
Ischemic Stroke	Center for Health Statistics, Ministry of Health (Ukraine). Ukraine Inpatient Care Discharges per 100 1998
Ischemic Stroke	Center for Health Statistics, Ministry of Health (Ukraine). Ukraine Inpatient Care Discharges per 100 1999
Ischemic Stroke	Center for Health Statistics, Ministry of Health (Ukraine). Ukraine Inpatient Care Discharges per 100 2000
Ischemic Stroke	Center for Health Statistics, Ministry of Health (Ukraine). Ukraine Inpatient Care Discharges per 100 2001
Ischemic Stroke	Center for Health Statistics, Ministry of Health (Ukraine). Ukraine Inpatient Care Discharges per 100 2002
Ischemic Stroke	Center for Health Statistics, Ministry of Health (Ukraine). Ukraine Inpatient Care Discharges per 100 2003
Ischemic Stroke	Center for Health Statistics, Ministry of Health (Ukraine). Ukraine Inpatient Care Discharges per 100 2004
Ischemic Stroke	Center for Health Statistics, Ministry of Health (Ukraine). Ukraine Inpatient Care Discharges per 100 2005
Ischemic Stroke	Center for Health Statistics, Ministry of Health (Ukraine). Ukraine Inpatient Care Discharges per 100 2006
Ischemic Stroke	Center for Health Statistics, Ministry of Health (Ukraine). Ukraine Inpatient Care Discharges per 100 2007
Ischemic Stroke	Center for Health Statistics, Ministry of Health (Ukraine). Ukraine Inpatient Care Discharges per 100 2008
Ischemic Stroke	Center for Health Statistics, Ministry of Health (Ukraine). Ukraine Inpatient Care Discharges per 100 2009
Ischemic Stroke	Center for Health Statistics, Ministry of Health (Ukraine). Ukraine Inpatient Care Discharges per 100 2010
Ischemic Stroke	Center for Health Statistics, Ministry of Health (Ukraine). Ukraine Inpatient Care Discharges per 100 2011
Ischemic Stroke	Center for Health Statistics, Ministry of Health (Ukraine). Ukraine Inpatient Care Discharges per 100 2012
Ischemic Stroke	Center for Health Statistics, Ministry of Health (Ukraine). Ukraine Inpatient Care Discharges per 100 2013
Ischemic Stroke	Institute of Public Health of Serbia. Serbia Inpatient Care Discharges Per 100 2010
Ischemic Stroke	Institute of Public Health of Serbia. Serbia Inpatient Care Discharges Per 100 2011
Ischemic Stroke	Institute of Public Health of Serbia. Serbia Inpatient Care Discharges Per 100 2013
Ischemic Stroke	Institute of Public Health of Serbia. Serbia Inpatient Care Discharges Per 100 2014
Ischemic Stroke	Department of Health, Social Services and Public Safety (Northern Ireland), Information Centre for Health and Social Care, NHS, NHS England, NHS Health Scotland, NHS Wales. United Kingdom Hospital Patient and Discharge Data 2012
Ischemic Stroke	Department of Health, Social Services and Public Safety (Northern Ireland), Information Centre for Health and Social Care, NHS, NHS England, NHS Health Scotland, NHS Wales. United Kingdom Hospital Patient and Discharge Data 2013

Ischemic Stroke	Department of Health, Social Services and Public Safety (Northern Ireland), Information Centre for Health and Social Care, NHS, NHS England, NHS Health Scotland, NHS Wales. United Kingdom Hospital Patient and Discharge Data 2014
Ischemic Stroke	National Board of Health and Welfare (Sweden). Sweden National Patient Register 1997
Ischemic Stroke	National Board of Health and Welfare (Sweden). Sweden National Patient Register 1996. Stockholm, Sweden: National Board of Health and Welfare (Sweden)
Ischemic Stroke	National Board of Health and Welfare (Sweden). Sweden National Patient Register 1995. Stockholm, Sweden: National Board of Health and Welfare (Sweden)
Ischemic Stroke	National Board of Health and Welfare (Sweden). Sweden National Patient Register 1994. Stockholm, Sweden: National Board of Health and Welfare (Sweden)
Ischemic Stroke	National Board of Health and Welfare (Sweden). Sweden National Patient Register 1993. Stockholm, Sweden: National Board of Health and Welfare (Sweden)
Ischemic Stroke	National Board of Health and Welfare (Sweden). Sweden National Patient Register 1992. Stockholm, Sweden: National Board of Health and Welfare (Sweden)
Ischemic Stroke	National Board of Health and Welfare (Sweden). Sweden National Patient Register 1991. Stockholm, Sweden: National Board of Health and Welfare (Sweden)
Ischemic Stroke	National Board of Health and Welfare (Sweden). Sweden National Patient Register 1990. Stockholm, Sweden: National Board of Health and Welfare (Sweden)
Ischemic Stroke	National Board of Health and Welfare (Sweden). Sweden National Patient Register 1989. Stockholm, Sweden: National Board of Health and Welfare (Sweden)
Ischemic Stroke	National Board of Health and Welfare (Sweden). Sweden National Patient Register 1988. Stockholm, Sweden: National Board of Health and Welfare (Sweden)
Ischemic Stroke	National Board of Health and Welfare (Sweden). Sweden National Patient Register 1987. Stockholm, Sweden: National Board of Health and Welfare (Sweden)
Ischemic Stroke	National Board of Health and Welfare (Sweden). Sweden National Patient Register 2013
Ischemic Stroke	National Board of Health and Welfare (Sweden). Sweden National Patient Register 2014
Ischemic Stroke	Joint Health Surveys Unit of Social and Community Planning Research and University College London, Scottish Health Survey, 1998 [computer file]. Colchester, Essex: UK Data Archive [distributor], July 2001. SN: 4379 , http://dx.doi.org/10.5255/UKDA-SN-4379-1
Ischemic Stroke	Joint Health Surveys Unit, University College London and Medical Research Council. Social and Public Health Sciences Unit, Scottish Health Survey, 2003 [computer file]. Colchester, Essex: UK Data Archive [distributor], February 2006. SN: 5318
Ischemic Stroke	World Health Organization (WHO). Bosnia and Herzegovina World Health Survey 2003. Geneva, Switzerland: World Health Organization (WHO), 2005
Ischemic Stroke	World Health Organization (WHO). Chad World Health Survey 2003. Geneva, Switzerland: World Health Organization (WHO), 2005
Ischemic Stroke	World Health Organization (WHO). Ireland World Health Survey 2003. Geneva, Switzerland: World Health Organization (WHO), 2005
Ischemic Stroke	World Health Organization (WHO). Nepal World Health Survey 2003. Geneva, Switzerland: World Health Organization (WHO), 2005
Ischemic Stroke	World Health Organization (WHO). Slovakia World Health Survey 2003. Geneva, Switzerland: World Health Organization (WHO), 2005
Ischemic Stroke	World Health Organization (WHO). Tunisia World Health Survey 2003. Geneva, Switzerland: World Health Organization (WHO), 2005
Ischemic Stroke	Centers for Disease Control and Prevention (CDC), Ministry of Health (Jordan), World Health Organization (WHO). Jordan STEPS Noncommunicable Disease Risk Factors Survey 2007
Ischemic Stroke	Srpska), Federal Office of Statistics (Bosnia and Herzegovina), Swedish International Development Agency (SIDA), UK Department for International Development (DFID), United Nations Development Programme (UNDP), European Commission (EC), Government of Japan, World Bank (WB). Bosnia and Herzegovina Living Standards
Ischemic Stroke	Srpska), Federal Office of Statistics (Bosnia and Herzegovina), Independent Bureau for Humanitarian Issues (IBHI), Birks Sinclair and Associates, LTD, Institute for Social and Economic Research, University of Essex. Bosnia and Herzegovina Living Standards Measurement Survey 2002. Washington, DC, United States: World Bank (WB)
Ischemic Stroke	Federal Office of Statistics (Federation of Bosnia and Herzegovina), Independent Bureau for Humanitarian Issues (IBHI), Institute for Social and Economic Research, University of Essex, Institute of Statistics (Republic of Srpska). Bosnia and Herzegovina Living Standards Measurement Survey 2004-2005
Ischemic Stroke	Public Health Authority of the Slovak Republic, World Health Organization (WHO). Slovakia WHO Multi-country Survey Study on Health and Health System Responsiveness 2000
Ischemic Stroke	University of Ibadan (Nigeria), World Health Organization (WHO). Nigeria WHO Multi-country Survey Study on Health and Health System Responsiveness 2000-2001
Ischemic Stroke	Scottish Centre for Social Research and University College London. Department of Epidemiology and Public Health, Scottish Health Survey, 2008 [computer file]. 2nd Edition. Colchester, Essex: UK Data Archive [distributor], April 2013. SN: 6383, http://dx.doi.org/10.5255/UKDA-SN-6383-2
Ischemic Stroke	Scottish Centre for Social Research and University College London. Department of Epidemiology and Public Health, Scottish Health Survey, 2009 [computer file]. 4th Edition. Colchester, Essex: UK Data Archive [distributor], November 2011. SN: 6713, http://dx.doi.org/10.5255/UKDA-SN-6713-2
Ischemic Stroke	ScotCen Social Research and University College London. Department of Epidemiology and Public Health, Scottish Health Survey, 2010 [computer file]. Colchester, Essex: UK Data Archive [distributor], April 2012. SN: 6987, http://dx.doi.org/10.5255/UKDA-SN-6987-1
Ischemic Stroke	and Public Health and University of Glasgow. MRC/CSO Social and Public Health Sciences Unit, Scottish Health Survey, 2011 [computer file]. 2nd Edition. Colchester, Essex: UK Data Archive [distributor], August 2013. SN: 7247, http://dx.doi.org/10.5255/UKDA-SN-7247-2
Ischemic Stroke	Longe AC, Osuntokun BO. Prevalence of neurological disorders in Udo, a rural community in southern Nigeria. Trop Geogr Med. 1989; 41(1): 36-40
Ischemic Stroke	Mrabet A, Attia-Romdhane N, Ben Hamida M, Gharbi N, Le Noan H, Hentati R, Ben Mansour J, Srairi I. Epidemiologic aspects of cerebrovascular accidents in Tunisia. Rev Neurol (Paris). 1990; 146(4): 297-301
Ischemic Stroke	Danesi M, Okubadejo N, Ojini F. Prevalence of stroke in an urban, mixed-income community in Lagos, Nigeria. Neuroepidemiology. 2007; 28(4): 216-23
Ischemic Stroke	Flynn RWV, MacDonald TM, Murray GD, Ferguson C, Shah K, Doney ASF. The Tayside Stroke Cohort: exploiting advanced regional medical informatics to create a region-wide database for studying the pharmacoepidemiology of stroke. Pharmacoepidemiol Drug Saf. 2010; 19(7): 737-44
Ischemic Stroke	Maheswaran R, Pearson T, Smeeton NC, Beevers SD, Campbell MJ, Wolfe CD. Outdoor air pollution and incidence of ischemic and hemorrhagic stroke: a small-area level ecological study. Stroke. 2012; 43(1): 22-7
Ischemic Stroke	Damasceno A, Gomes J, Azevedo A, Carrilho C, Lobo V, Lopes H, Madede T, Pravinrai P, Silva-Matos C, Jalla S, Stewart S, Lunet N. An Epidemiological Study of Stroke Hospitalizations in Maputo, Mozambique A High Burden of Disease in a Resource-Poor Country. Stroke. 2010; 41(11): 2463-9
Ischemic Stroke	National Bureau of Statistics (Nigeria). Nigeria Living Standards Survey 2008-2010. Abuja, Nigeria: National Bureau of Statistics (Nigeria)
Ischemic Stroke	Trinity College Dublin. Ireland Longitudinal Study on Ageing 2012-2013. Dublin, Ireland: Irish Social Science Data Archive, University College Dublin
Ischemic Stroke	ScotCen Social Research, Scottish Health Survey, 2013 [computer file]. Colchester, Essex: UK Data Archive [distributor], December 2014. SN: 7594, http://dx.doi.org/10.5255/UKDA-SN-7594-1
Ischemic Stroke	Department of Epidemiology and Public Health, University College London, Scottish Centre for Social Research (ScotCen), University of Glasgow. United Kingdom - Scottish Health Survey 2012 - Scottish Government

Ischemic Stroke	LINK Institute for Market and Social Research (Switzerland), World Health Organization (WHO). Switzerland WHO Multi-country Survey Study on Health and Health System Responsiveness 2000-2001. Geneva, Switzerland: World Health Organization (WHO)
Ischemic Stroke	International Research Associates (INRA) Europe, World Health Organization (WHO). Ireland WHO Multi-country Survey Study on Health and Health System Responsiveness 2000-2001. Geneva, Switzerland: World Health Organization (WHO)
Ischemic Stroke	Gallup Europe, World Health Organization (WHO). Jordan WHO Multi-country Survey Study on Health and Health System Responsiveness 2000-2001. Geneva, Switzerland: World Health Organization (WHO)
Ischemic Stroke	Börsch-Supan, A. (2015). Survey of Health, Ageing and Retirement in Europe (SHARE) Wave 5. Release version: 1.0.0. SHARE-ERIC. Data set. DOI: 10.6103/SHARE.w5.100
Ischemic Stroke	Concluzia-Prim Center for Survey Methodology (Moldova), Institute for Advanced Studies (Austria), London School of Hygiene and Tropical Medicine, SIAR Research and Consulting (Azerbaijan), University of Aberdeen. Azerbaijan Health in Times of Transition Household Survey 2010
Ischemic Stroke	ScotCen Social Research. (2015). Scottish Health Survey, 2014. [data collection]. UK Data Service. SN: 7851, http://dx.doi.org/10.5255/UKDA-SN-7851-1
Ischemic Stroke	Counsell C, Dennis M, McDowall M. Predicting functional outcome in acute stroke: comparison of a simple six variable model with other predictive systems and informal clinical prediction. J Neurol Neurosurg Psychiatry. 2004; 75(3): 401–5
Ischemic Stroke	Abubakar SA, Okubadejo NU, Ojo OO, Oladipo O, Ojini FI, Danesi MA. Relationship between admission serum C-reactive protein and short term outcome following acute ischaemic stroke at a tertiary health institution in Nigeria. Niger J Clin Pract. 2013; 16(3): 320–4
Ischemic Stroke	Department of Health and Children (Ireland), Economic and Social Research Institute (ESRI) (Ireland). Ireland Hospital Inpatient Enquiry 2000
Ischemic Stroke	Department of Health (Ireland), Economic and Social Research Institute (ESRI) (Ireland). Ireland Hospital Inpatient Enquiry 1980
Ischemic Stroke	Department of Health (Ireland), Economic and Social Research Institute (ESRI) (Ireland). Ireland Hospital Inpatient Enquiry 1981
Ischemic Stroke	Department of Health (Ireland), Economic and Social Research Institute (ESRI) (Ireland). Ireland Hospital Inpatient Enquiry 1982
Ischemic Stroke	Department of Health (Ireland), Economic and Social Research Institute (ESRI) (Ireland). Ireland Hospital Inpatient Enquiry 1983
Ischemic Stroke	Department of Health (Ireland), Economic and Social Research Institute (ESRI) (Ireland). Ireland Hospital Inpatient Enquiry 1984
Ischemic Stroke	Department of Health (Ireland), Economic and Social Research Institute (ESRI) (Ireland). Ireland Hospital Inpatient Enquiry 1985
Ischemic Stroke	Department of Health (Ireland), Economic and Social Research Institute (ESRI) (Ireland). Ireland Hospital Inpatient Enquiry 1986
Ischemic Stroke	Department of Health (Ireland), Economic and Social Research Institute (ESRI) (Ireland). Ireland Hospital Inpatient Enquiry 1987
Ischemic Stroke	Department of Health (Ireland), Economic and Social Research Institute (ESRI) (Ireland). Ireland Hospital Inpatient Enquiry 1988
Ischemic Stroke	Department of Health (Ireland), Economic and Social Research Institute (ESRI) (Ireland). Ireland Hospital Inpatient Enquiry 1989
Ischemic Stroke	Department of Health (Ireland), Economic and Social Research Institute (ESRI) (Ireland). Ireland Hospital Inpatient Enquiry 1990
Ischemic Stroke	Department of Health (Ireland), Economic and Social Research Institute (ESRI) (Ireland). Ireland Hospital Inpatient Enquiry 1991
Ischemic Stroke	Department of Health (Ireland), Economic and Social Research Institute (ESRI) (Ireland). Ireland Hospital Inpatient Enquiry 1992
Ischemic Stroke	Department of Health (Ireland), Economic and Social Research Institute (ESRI) (Ireland). Ireland Hospital Inpatient Enquiry 1993
Ischemic Stroke	Department of Health (Ireland), Economic and Social Research Institute (ESRI) (Ireland). Ireland Hospital Inpatient Enquiry 1994
Ischemic Stroke	Department of Health (Ireland), Economic and Social Research Institute (ESRI) (Ireland). Ireland Hospital Inpatient Enquiry 1995
Ischemic Stroke	Department of Health (Ireland), Economic and Social Research Institute (ESRI) (Ireland). Ireland Hospital Inpatient Enquiry 1996
Ischemic Stroke	Department of Health and Children (Ireland), Economic and Social Research Institute (ESRI) (Ireland). Ireland Hospital Inpatient Enquiry 1997
Ischemic Stroke	Department of Health and Children (Ireland), Economic and Social Research Institute (ESRI) (Ireland). Ireland Hospital Inpatient Enquiry 1998
Ischemic Stroke	Department of Health and Children (Ireland), Economic and Social Research Institute (ESRI) (Ireland). Ireland Hospital Inpatient Enquiry 1999
Ischemic Stroke	Economic and Social Research Institute (ESRI) (Ireland), Health Service Executive (HSE) (Ireland). Ireland Hospital Inpatient Enquiry 2013
Ischemic Stroke	Economic and Social Research Institute (ESRI) (Ireland), Health Service Executive (HSE) (Ireland). Ireland Hospital Inpatient Enquiry 2014
Ischemic Stroke	Federal Statistical Office (Switzerland). Switzerland Medical Statistics of Hospitals 1997
Ischemic Stroke	Federal Statistical Office (Switzerland). Switzerland Medical Statistics of Hospitals 1998
Ischemic Stroke	Federal Statistical Office (Switzerland). Switzerland Medical Statistics of Hospitals 2013
Ischemic Stroke	Federal Statistical Office (Switzerland). Switzerland Medical Statistics of Hospitals 2014
Ischemic Stroke	National Statistical Institute of Bulgaria. Bulgaria Living Standards Measurement Survey 2003. Washington DC, United States: World Bank
Ischemic Stroke	Administrative Department of Science, Technology, and Innovation (Colombia), Center for Development Projects, Pontifical Xavierian University, Ministry of Social Protection (Colombia), Specialized Information Systems. Colombia National Health Survey 2007-2008
Ischemic Stroke	Planning Institute of Jamaica, Statistical Institute of Jamaica. Jamaica Survey of Living Conditions 1988. Kingston, Jamaica: Planning Institute of Jamaica
Ischemic Stroke	Statistical Institute of Jamaica. Jamaica Survey of Living Conditions 1990. Washington DC, United States: World Bank
Ischemic Stroke	Planning Institute of Jamaica, Statistical Institute of Jamaica. Jamaica Survey of Living Conditions 1992
Ischemic Stroke	Planning Institute of Jamaica, Statistical Institute of Jamaica. Jamaica Survey of Living Conditions 1991
Ischemic Stroke	World Health Organization (WHO). Austria World Health Survey 2003. Geneva, Switzerland: World Health Organization (WHO), 2005
Ischemic Stroke	Center for Scientific and Technological Information, Oswaldo Cruz Foundation and World Health Organization (WHO). Brazil World Health Survey 2003. Geneva, Switzerland: World Health Organization (WHO), 2005
Ischemic Stroke	World Health Organization (WHO). Côte d'Ivoire World Health Survey 2003. Geneva, Switzerland: World Health Organization (WHO), 2005
Ischemic Stroke	World Health Organization (WHO). Georgia World Health Survey 2003. Geneva, Switzerland: World Health Organization (WHO), 2005
Ischemic Stroke	World Health Organization (WHO). Israel World Health Survey 2003. Geneva, Switzerland: World Health Organization (WHO), 2005
Ischemic Stroke	World Health Organization (WHO). Myanmar World Health Survey 2003. Geneva, Switzerland: World Health Organization (WHO), 2005
Ischemic Stroke	World Health Organization (WHO). Namibia World Health Survey 2003. Geneva, Switzerland: World Health Organization (WHO), 2005
Ischemic Stroke	World Health Organization (WHO). Pakistan World Health Survey 2003-2004. Geneva, Switzerland: World Health Organization (WHO), 2005
Ischemic Stroke	World Health Organization (WHO). Slovenia World Health Survey 2003. Geneva, Switzerland: World Health Organization (WHO), 2005
Ischemic Stroke	World Health Organization (WHO). United Kingdom World Health Survey 2004. Geneva, Switzerland: World Health Organization (WHO), 2005
Ischemic Stroke	Office of Population Censuses and Surveys. Social Survey Division, Health Survey for England, 1993 [Computer file]. Colchester, Essex: UK Data Archive [distributor], April 1995. SN: 3316, http://dx.doi.org/10.5255/UKDA-SN-3316-1
Ischemic Stroke	Joint Health Surveys Unit of Social and Community Planning Research and University College London, Health Survey for England, 1994 [computer file]. 4th ed. Colchester, Essex: UK Data Archive [distributor], 26 March 2001. SN: 3640
Ischemic Stroke	National Centre for Social Research, University College London Department of Epidemiology and Public Health, Health Survey for England, 1998 [computer file]. 4th ed. Colchester, Essex: UK Data Archive [distributor], 30 November 2002. SN: 4150
Ischemic Stroke	National Centre for Social Research, University College London Department of Epidemiology and Public Health, Health Survey for England, 2000 [computer file]. Colchester, Essex: UK Data Archive [distributor], 23 April 2002. SN: 4487
Ischemic Stroke	National Centre for Social Research and University College London. Department of Epidemiology and Public Health, Health Survey for England, 2003 [computer file]. Colchester, Essex: UK Data Archive [distributor], March 2005. SN: 5098
Ischemic Stroke	National Centre for Social Research and University College London. Department of Epidemiology and Public Health, Health Survey for England, 2005 [computer file]. Colchester, Essex: UK Data Archive [distributor], July 2007. SN: 5675
Ischemic Stroke	National Centre for Social Research and University College London. Department of Epidemiology and Public Health, Health Survey for England, 2006 [computer file]. 4th Edition. Colchester, Essex: UK Data Archive [distributor], July 2011. SN: 5809, http://dx.doi.org/10.5255/UKDA-SN-5809-1

Ischemic Stroke	University of Wisconsin-Madison, Inter-University Consortium for Political and Social Research (ICPSR), University of São Paulo. Brazil - São Paulo Survey on Health, Well-Being, and Aging in Latin America and the Caribbean 1999-2000. Ann Arbor, United States: Inter-University Consortium for Political and Social Research (ICPSR)
Ischemic Stroke	Planning Institute of Jamaica, Statistical Institute of Jamaica. Jamaica Survey of Living Conditions 1993
Ischemic Stroke	Planning Institute of Jamaica, Statistical Institute of Jamaica. Jamaica Survey of Living Conditions 1994
Ischemic Stroke	Planning Institute of Jamaica, Statistical Institute of Jamaica. Jamaica Survey of Living Conditions 1995
Ischemic Stroke	Planning Institute of Jamaica, Statistical Institute of Jamaica. Jamaica Survey of Living Conditions 1996
Ischemic Stroke	Planning Institute of Jamaica, Statistical Institute of Jamaica. Jamaica Survey of Living Conditions 1997
Ischemic Stroke	Planning Institute of Jamaica, Statistical Institute of Jamaica. Jamaica Survey of Living Conditions 1999
Ischemic Stroke	Planning Institute of Jamaica, Statistical Institute of Jamaica. Jamaica Survey of Living Conditions 2000
Ischemic Stroke	TNS BBSS, World Bank. Bulgaria Living Standards Measurement Survey 2001. Washington DC, United States: World Bank
Ischemic Stroke	National Administrative Department of Statistics (Colombia). Colombia National Quality of Life Survey 1997. Bogotá, Colombia: National Administrative Department of Statistics (Colombia)
Ischemic Stroke	National Administrative Department of Statistics (Colombia). Colombia National Quality of Life Survey 2008. Bogotá, Colombia: National Administrative Department of Statistics (Colombia)
Ischemic Stroke	National Administrative Department of Statistics (Colombia). Colombia National Quality of Life Survey 2010. Bogotá, Colombia: National Administrative Department of Statistics (Colombia), 2012
Ischemic Stroke	Pontificia Universidad Javeriana (Colombia), World Health Organization (WHO). Colombia WHO Multi-country Survey Study on Health and Health System Responsiveness 2000-2001. Geneva, Switzerland: World Health Organization (WHO)
Ischemic Stroke	AMATEM (Turkey), Plaza Ltd. Research, World Health Organization (WHO). Turkey WHO Multi-country Survey Study on Health and Health System Responsiveness 2000-2001. Geneva, Switzerland: World Health Organization (WHO)
Ischemic Stroke	Institute for Polling and Marketing (Georgia), World Health Organization (WHO). Georgia WHO Multi-country Survey Study on Health and Health System Responsiveness 2000-2001
Ischemic Stroke	Turkish Statistical Institute. Turkey Health Interview Survey 2010. Ankara, Turkey: Turkish Statistical Institute
Ischemic Stroke	NatCen Social Research and University College London. Department of Epidemiology and Public Health, Health Survey for England, 2011 [computer file]. Colchester, Essex: UK Data Archive [distributor], April 2013. SN: 7260, http://dx.doi.org/10.5255/UKDA-SN-7260-1
Ischemic Stroke	Minelli C, Fu Fen L, Camara Minelli DP. Stroke Incidence, Prognosis, 30-Day, and 1-Year Case Fatality Rates in Matão, Brazil. Stroke. 2007; 38(11): 2906-11
Ischemic Stroke	Cabral NL, Gonçalves ARR, Longo AL, Moro CHC, Costa G, Amaral CH, Fonseca L a M, Eluf-Neto J. Incidence of stroke subtypes, prognosis and prevalence of risk factors in Joinville, Brazil: a 2 year community based study. J Neurol Neurosurg Psychiatr. 2009; 80(7): 755-61
Ischemic Stroke	Abdul-Ghaffar NU, el-Sonbaty MR, el-Din Abdul-Baky MS, Marafie AA, al-Said AM. Stroke in Kuwait: a three-year prospective study. Neuroepidemiology. 1997; 16(1): 40-7
Ischemic Stroke	Börü UT, Oztürk E, Ta?demir M, Sur H. Living alone following first-ever stroke: a prospective study in Turkey identifying the risk factors and evaluating their effects. N Z Med J. 2007; 120(1255): U2559
Ischemic Stroke	Du X, Sourbutts J, Cruickshank K, Alison Summers, Roberts N, Walton E, Holmes S. A Community Based Stroke Register in a High Risk Area for Stroke in North West England. J Epidemiol Community Health. 1997; 51(5): 472-8
Ischemic Stroke	Kumral E, Ozkaya B, Sagduyu A, Sirin H, Vardarli E, Pehlivan M. The Ege Stroke Registry: A Hospital-Based Study in the Aegean Region, Izmir, Turkey. Cerebrovasc Dis. 1998; 8(5): 278-88
Ischemic Stroke	Tsiskaridze A, Djibuti M, van Melle G, Lomidze G, Apridonidze S, Gauarashvili I, Piechowski-Józwiak B, Shakarishvili R, Bogousslavsky J. Stroke Incidence and 30-Day Case-Fatality in a Suburb of Tbilisi: Results of the First Prospective Population-Based Study in Georgia. Stroke. 2004; 35(11): 2523-8
Ischemic Stroke	Al-Shammri S, Shahid Z, Ghali A, Mehndiratta MM, Swaminathan TR, Chadha G, Sharma PN, Akanji AO. Risk Factors, Subtypes and Outcome of Ischaemic Stroke in Kuwait - A Hospital-Based Study. Med Princ Pract. 2003; 12(4): 218-23
Ischemic Stroke	Statistics Austria, World Health Organization (WHO). Austria WHO Multi-country Survey Study on Health and Health System Responsiveness 2000-2001. Geneva, Switzerland: World Health Organization (WHO)
Ischemic Stroke	National Assembly for Wales, Welsh Health Survey, 1998 [computer file]. 2nd Edition. Colchester, Essex: UK Data Archive [distributor], February 2011. SN: 4176 , http://dx.doi.org/10.5255/UKDA-SN-4176-1
Ischemic Stroke	National Centre for Social Research, Beaufort Research Limited and University College London. Department of Epidemiology and Public Health, Welsh Health Survey, 2003-2004 [computer file]. 2nd Edition. Colchester, Essex: UK Data Archive [distributor], February 2011. SN: 5692 , http://dx.doi.org/10.5255/UKDA-SN-5692-1
Ischemic Stroke	National Centre for Social Research, Welsh Health Survey, 2005-2006 [computer file]. 2nd Edition. Colchester, Essex: UK Data Archive [distributor], February 2011. SN: 5750 , http://dx.doi.org/10.5255/UKDA-SN-5750-1
Ischemic Stroke	National Centre for Social Research, Welsh Health Survey, 2007 [computer file]. 2nd Edition. Colchester, Essex: UK Data Archive [distributor], February 2011. SN: 6052, http://dx.doi.org/10.5255/UKDA-SN-6052-1
Ischemic Stroke	National Centre for Social Research, Welsh Health Survey, 2008 [computer file]. 2nd Edition. Colchester, Essex: UK Data Archive [distributor], February 2011. SN: 6372
Ischemic Stroke	National Centre for Social Research, Welsh Health Survey, 2009 [computer file]. 2nd Edition. Colchester, Essex: UK Data Archive [distributor], February 2011. SN: 6589, http://dx.doi.org/10.5255/UKDA-SN-6589-1
Ischemic Stroke	National Centre for Social Research, Welsh Health Survey, 2010 [computer file]. Colchester, Essex: UK Data Archive [distributor], November 2011. SN: 6895, http://dx.doi.org/10.5255/UKDA-SN-6895-1
Ischemic Stroke	NatCen Social Research, Welsh Health Survey, 2011 [computer file]. Colchester, Essex: UK Data Archive [distributor], January 2013. SN: 7188, http://dx.doi.org/10.5255/UKDA-SN-7188-1
Ischemic Stroke	NatCen Social Research, Welsh Health Survey, 2012 [computer file]. Colchester, Essex: UK Data Archive [distributor], February 2014. SN: 7459, http://dx.doi.org/10.5255/UKDA-SN-7459-1
Ischemic Stroke	Federal Ministry of Health (Austria), Statistics Austria. Austria Hospital Inpatient Discharges 1989. Vienna, Austria: Statistics Austria
Ischemic Stroke	National Institute of Statistics of Rwanda. Rwanda Integrated Household Living Conditions Survey 2010-2011. Kigali, Rwanda: National Institute of Statistics of Rwanda
Ischemic Stroke	Health Institute (São Paulo, Brazil), State University of Campinas, São Paulo Municipal Health Department, São Paulo State University, University of São Paulo. Brazil - São Paulo Health Survey 2008-2009
Ischemic Stroke	NatCen Social Research, Welsh Health Survey, 2013 [computer file]. Colchester, Essex: UK Data Archive [distributor], January 2015. SN: 7632, http://dx.doi.org/10.5255/UKDA-SN-7632-1
Ischemic Stroke	Brazilian Institute of Geography and Statistics (IBGE), Ministry of Health (Brazil), Ministry of Planning, Budget, and Management (Brazil). Brazil National Health Survey 2013. Rio de Janeiro, Brazil: Brazilian Institute of Geography and Statistics (IBGE)
Ischemic Stroke	Federal Ministry of Health (Austria), Statistics Austria. Austria Hospital Inpatient Discharges 2013. Vienna, Austria: Statistics Austria
Ischemic Stroke	International Research Associates (INRA) Europe, World Health Organization (WHO). Bulgaria WHO Multi-country Survey Study on Health and Health System Responsiveness 2000-2001. Geneva, Switzerland: World Health Organization (WHO)

Ischemic Stroke	National Centre for Social Research (NatCen), World Health Organization (WHO). United Kingdom WHO Multi-country Survey Study on Health and Health System Responsiveness 2000-2001. Geneva, Switzerland: World Health Organization (WHO)
Ischemic Stroke	Börsch-Supan, A. (2015). Survey of Health, Ageing and Retirement in Europe (SHARE) Wave 5. Release version: 1.0.0. SHARE-ERIC. Data set. DOI: 10.6103/SHARE.w5.100
Ischemic Stroke	Börsch-Supan, A. (2015). Survey of Health, Ageing and Retirement in Europe (SHARE) Wave 5. Release version: 1.0.0. SHARE-ERIC. Data set. DOI: 10.6103/SHARE.w5.100
Ischemic Stroke	Börsch-Supan, A. (2015). Survey of Health, Ageing and Retirement in Europe (SHARE) Wave 5. Release version: 1.0.0. SHARE-ERIC. Data set. DOI: 10.6103/SHARE.w5.100
Ischemic Stroke	National Center for Disease Control and Public Health (Georgia). Georgia Hospital Data 2014
Ischemic Stroke	Concluzia-Prim Center for Survey Methodology (Moldova), Georgia Opinion Research Business International (GORBI), Institute for Advanced Studies (Austria), London School of Hygiene and Tropical Medicine, University of Aberdeen. Georgia Health in Times of Transition Household Survey 2010
Ischemic Stroke	Federal Ministry of Health (Austria), Statistics Austria. Austria Hospital Inpatient Discharges 1989-1992
Ischemic Stroke	Federal Ministry of Health (Austria), Statistics Austria. Austria Hospital Inpatient Discharges 1993-1997
Ischemic Stroke	Federal Ministry of Health (Austria), Statistics Austria. Austria Hospital Inpatient Discharges 1998-2002
Ischemic Stroke	Federal Ministry of Health (Austria), Statistics Austria. Austria Hospital Inpatient Discharges 2003-2007
Ischemic Stroke	Federal Ministry of Health (Austria), Statistics Austria. Austria Hospital Inpatient Discharges 2008-2012
Ischemic Stroke	Ministry of Labor and Social Policy (Bulgaria), National Statistical Institute of Bulgaria, TNS Gallup, World Bank. Bulgaria Multitopic Household Survey 2007. Washington DC, United States: World Bank
Ischemic Stroke	Federal Ministry of Health (Austria), Statistics Austria. Austria Hospital Inpatient Discharges 2014. Vienna, Austria: Statistics Austria
Ischemic Stroke	Rivero-Arias O, Ouellet M, Gray A, Wolstenholme J, Rothwell PM, Luengo-Fernandez R. Mapping the modified Rankin scale (mRS) measurement into the generic EuroQol (EQ-5D) health outcome. Med Decis Mak. 2010; 30(3): 341–54
Ischemic Stroke	Luengo-Fernandez R, Paul NLM, Gray AM, Pendlebury ST, Bull LM, Welch SJV, Cuthbertson FC, Rothwell PM, Oxford Vascular Study. Population-based study of disability and institutionalization after transient ischemic attack and stroke: 10-year results of the Oxford Vascular Study. Stroke. 2013; 44(10): 2854–61
Ischemic Stroke	Federal Ministry of Health (Austria), Statistics Austria. Austria Hospital Inpatient Discharges 2013-2014
Ischemic Stroke	Fernandes TG, Goulart AC, Santos-Junior WR, Alencar AP, Benseñor IM, Lotufo PA. Educational levels and the functional dependence of ischemic stroke survivors. Cad Saude Publica. 2012; 28(8): 1581–90
Ischemic Stroke	National Institute of Public Health (Slovenia). Slovenia National Hospital Health Care Statistics Database 2004
Ischemic Stroke	National Institute of Public Health (Slovenia). Slovenia National Hospital Health Care Statistics Database 2005
Ischemic Stroke	National Institute of Public Health (Slovenia). Slovenia National Hospital Health Care Statistics Database 2006
Ischemic Stroke	National Institute of Public Health (Slovenia). Slovenia National Hospital Health Care Statistics Database 2007
Ischemic Stroke	National Institute of Public Health (Slovenia). Slovenia National Hospital Health Care Statistics Database 2008
Ischemic Stroke	General Directorate of Curative Services, Ministry of Health (Turkey). Turkey Hospital Inpatient Discharges 2007
Ischemic Stroke	General Directorate of Curative Services, Ministry of Health (Turkey). Turkey Hospital Inpatient Discharges 2008
Ischemic Stroke	General Directorate of Curative Services, Ministry of Health (Turkey). Turkey Hospital Inpatient Discharges 2009
Ischemic Stroke	General Directorate of Curative Services, Ministry of Health (Turkey). Turkey Hospital Inpatient Discharges 2010
Ischemic Stroke	General Directorate of Curative Services, Ministry of Health (Turkey). Turkey Hospital Inpatient Discharges 2011
Ischemic Stroke	National Center for Disease Control and Public Health (Georgia). Georgia Inpatient Care Discharges per 100 1991
Ischemic Stroke	National Center for Disease Control and Public Health (Georgia). Georgia Inpatient Care Discharges per 100 1992
Ischemic Stroke	National Center for Disease Control and Public Health (Georgia). Georgia Inpatient Care Discharges per 100 1993
Ischemic Stroke	National Center for Disease Control and Public Health (Georgia). Georgia Inpatient Care Discharges per 100 1994
Ischemic Stroke	National Center for Disease Control and Public Health (Georgia). Georgia Inpatient Care Discharges per 100 1995
Ischemic Stroke	National Center for Disease Control and Public Health (Georgia). Georgia Inpatient Care Discharges per 100 1996
Ischemic Stroke	National Center for Disease Control and Public Health (Georgia). Georgia Inpatient Care Discharges per 100 1997
Ischemic Stroke	National Center for Disease Control and Public Health (Georgia). Georgia Inpatient Care Discharges per 100 1998
Ischemic Stroke	National Center for Disease Control and Public Health (Georgia). Georgia Inpatient Care Discharges per 100 1999
Ischemic Stroke	National Center for Disease Control and Public Health (Georgia). Georgia Inpatient Care Discharges per 100 2000
Ischemic Stroke	National Center for Disease Control and Public Health (Georgia). Georgia Inpatient Care Discharges per 100 2001
Ischemic Stroke	National Center for Disease Control and Public Health (Georgia). Georgia Inpatient Care Discharges per 100 2002
Ischemic Stroke	National Center for Disease Control and Public Health (Georgia). Georgia Inpatient Care Discharges per 100 2003
Ischemic Stroke	National Center for Disease Control and Public Health (Georgia). Georgia Inpatient Care Discharges per 100 2004
Ischemic Stroke	National Center for Disease Control and Public Health (Georgia). Georgia Inpatient Care Discharges per 100 2005
Ischemic Stroke	National Center for Disease Control and Public Health (Georgia). Georgia Inpatient Care Discharges per 100 2006
Ischemic Stroke	National Center for Disease Control and Public Health (Georgia). Georgia Inpatient Care Discharges per 100 2007
Ischemic Stroke	National Center for Disease Control and Public Health (Georgia). Georgia Inpatient Care Discharges per 100 2008
Ischemic Stroke	National Center for Disease Control and Public Health (Georgia). Georgia Inpatient Care Discharges per 100 2009
Ischemic Stroke	National Center for Disease Control and Public Health (Georgia). Georgia Inpatient Care Discharges per 100 2010
Ischemic Stroke	National Center for Disease Control and Public Health (Georgia). Georgia Inpatient Care Discharges per 100 2011
Ischemic Stroke	National Center for Disease Control and Public Health (Georgia). Georgia Inpatient Care Discharges per 100 2012
Ischemic Stroke	National Center for Disease Control and Public Health (Georgia). Georgia Inpatient Care Discharges per 100 2013
Ischemic Stroke	National Center for Disease Control and Public Health (Georgia). Georgia Inpatient Care Discharges per 100 2014
Ischemic Stroke	Ministry of Health (Israel). Israel National Hospital Discharge Database 2011
Ischemic Stroke	Ministry of Health (Israel). Israel National Hospital Discharge Database 2012
Ischemic Stroke	Ministry of Health (Israel). Israel National Hospital Discharge Database 2013
Ischemic Stroke	Ministry of Health (Israel). Israel National Hospital Discharge Database 2014
Ischemic Stroke	National Center for Disease Control and Public Health (Georgia). Georgia Inpatient Care Discharges per 100 1980
Ischemic Stroke	National Center for Disease Control and Public Health (Georgia). Georgia Inpatient Care Discharges per 100 1985
Ischemic Stroke	National Center for Disease Control and Public Health (Georgia). Georgia Inpatient Care Discharges per 100 1986
Ischemic Stroke	National Center for Disease Control and Public Health (Georgia). Georgia Inpatient Care Discharges per 100 1987
Ischemic Stroke	National Center for Disease Control and Public Health (Georgia). Georgia Inpatient Care Discharges per 100 1988
Ischemic Stroke	National Center for Disease Control and Public Health (Georgia). Georgia Inpatient Care Discharges per 100 1989
Ischemic Stroke	National Center for Disease Control and Public Health (Georgia). Georgia Inpatient Care Discharges per 100 1990
Ischemic Stroke	General Directorate of Curative Services, Ministry of Health (Turkey). Turkey Inpatient Care Discharges per 100 1980
Ischemic Stroke	General Directorate of Curative Services, Ministry of Health (Turkey). Turkey Inpatient Care Discharges per 100 1981
Ischemic Stroke	General Directorate of Curative Services, Ministry of Health (Turkey). Turkey Inpatient Care Discharges per 100 1982

Ischemic Stroke	General Directorate of Curative Services, Ministry of Health (Turkey). Turkey Inpatient Care Discharges per 100 1983
Ischemic Stroke	General Directorate of Curative Services, Ministry of Health (Turkey). Turkey Inpatient Care Discharges per 100 1984
Ischemic Stroke	General Directorate of Curative Services, Ministry of Health (Turkey). Turkey Inpatient Care Discharges per 100 1985
Ischemic Stroke	General Directorate of Curative Services, Ministry of Health (Turkey). Turkey Inpatient Care Discharges per 100 1986
Ischemic Stroke	General Directorate of Curative Services, Ministry of Health (Turkey). Turkey Inpatient Care Discharges per 100 1987
Ischemic Stroke	General Directorate of Curative Services, Ministry of Health (Turkey). Turkey Inpatient Care Discharges per 100 1988
Ischemic Stroke	General Directorate of Curative Services, Ministry of Health (Turkey). Turkey Inpatient Care Discharges per 100 1989
Ischemic Stroke	General Directorate of Curative Services, Ministry of Health (Turkey). Turkey Inpatient Care Discharges per 100 1990
Ischemic Stroke	General Directorate of Curative Services, Ministry of Health (Turkey). Turkey Inpatient Care Discharges per 100 1991
Ischemic Stroke	General Directorate of Curative Services, Ministry of Health (Turkey). Turkey Inpatient Care Discharges per 100 1992
Ischemic Stroke	General Directorate of Curative Services, Ministry of Health (Turkey). Turkey Inpatient Care Discharges per 100 1993
Ischemic Stroke	General Directorate of Curative Services, Ministry of Health (Turkey). Turkey Inpatient Care Discharges per 100 1994
Ischemic Stroke	General Directorate of Curative Services, Ministry of Health (Turkey). Turkey Inpatient Care Discharges per 100 1995
Ischemic Stroke	General Directorate of Curative Services, Ministry of Health (Turkey). Turkey Inpatient Care Discharges per 100 1996
Ischemic Stroke	General Directorate of Curative Services, Ministry of Health (Turkey). Turkey Inpatient Care Discharges per 100 1997
Ischemic Stroke	General Directorate of Curative Services, Ministry of Health (Turkey). Turkey Inpatient Care Discharges per 100 1998
Ischemic Stroke	General Directorate of Curative Services, Ministry of Health (Turkey). Turkey Inpatient Care Discharges per 100 1999
Ischemic Stroke	General Directorate of Curative Services, Ministry of Health (Turkey). Turkey Inpatient Care Discharges per 100 2000
Ischemic Stroke	General Directorate of Curative Services, Ministry of Health (Turkey). Turkey Inpatient Care Discharges per 100 2001
Ischemic Stroke	General Directorate of Curative Services, Ministry of Health (Turkey). Turkey Inpatient Care Discharges per 100 2002
Ischemic Stroke	General Directorate of Curative Services, Ministry of Health (Turkey). Turkey Inpatient Care Discharges per 100 2003
Ischemic Stroke	General Directorate of Curative Services, Ministry of Health (Turkey). Turkey Inpatient Care Discharges per 100 2004
Ischemic Stroke	General Directorate of Curative Services, Ministry of Health (Turkey). Turkey Inpatient Care Discharges per 100 2005
Ischemic Stroke	General Directorate of Curative Services, Ministry of Health (Turkey). Turkey Inpatient Care Discharges per 100 2006
Ischemic Stroke	General Directorate of Curative Services, Ministry of Health (Turkey). Turkey Inpatient Care Discharges per 100 2012
Ischemic Stroke	General Directorate of Curative Services, Ministry of Health (Turkey). Turkey Inpatient Care Discharges per 100 2013
Ischemic Stroke	General Directorate of Curative Services, Ministry of Health (Turkey). Turkey Inpatient Care Discharges per 100 2014
Ischemic Stroke	National Institute of Public Health (Slovenia). Slovenia National Hospital Health Care Statistics Database 1980
Ischemic Stroke	National Institute of Public Health (Slovenia). Slovenia National Hospital Health Care Statistics Database 1986
Ischemic Stroke	National Institute of Public Health (Slovenia). Slovenia National Hospital Health Care Statistics Database 1985
Ischemic Stroke	National Institute of Public Health (Slovenia). Slovenia National Hospital Health Care Statistics Database 1987
Ischemic Stroke	National Institute of Public Health (Slovenia). Slovenia National Hospital Health Care Statistics Database 1988
Ischemic Stroke	National Institute of Public Health (Slovenia). Slovenia National Hospital Health Care Statistics Database 1989
Ischemic Stroke	National Institute of Public Health (Slovenia). Slovenia National Hospital Health Care Statistics Database 1990
Ischemic Stroke	National Institute of Public Health (Slovenia). Slovenia National Hospital Health Care Statistics Database 1991
Ischemic Stroke	National Institute of Public Health (Slovenia). Slovenia National Hospital Health Care Statistics Database 1992
Ischemic Stroke	National Institute of Public Health (Slovenia). Slovenia National Hospital Health Care Statistics Database 1993
Ischemic Stroke	National Institute of Public Health (Slovenia). Slovenia National Hospital Health Care Statistics Database 1994
Ischemic Stroke	National Institute of Public Health (Slovenia). Slovenia National Hospital Health Care Statistics Database 1995
Ischemic Stroke	National Institute of Public Health (Slovenia). Slovenia National Hospital Health Care Statistics Database 1996
Ischemic Stroke	National Institute of Public Health (Slovenia). Slovenia National Hospital Health Care Statistics Database 1997
Ischemic Stroke	National Institute of Public Health (Slovenia). Slovenia National Hospital Health Care Statistics Database 1998
Ischemic Stroke	National Institute of Public Health (Slovenia). Slovenia National Hospital Health Care Statistics Database 1999
Ischemic Stroke	National Institute of Public Health (Slovenia). Slovenia National Hospital Health Care Statistics Database 2000
Ischemic Stroke	National Institute of Public Health (Slovenia). Slovenia National Hospital Health Care Statistics Database 2001
Ischemic Stroke	National Institute of Public Health (Slovenia). Slovenia National Hospital Health Care Statistics Database 2002
Ischemic Stroke	National Institute of Public Health (Slovenia). Slovenia National Hospital Health Care Statistics Database 2003
Ischemic Stroke	National Institute of Public Health (Slovenia). Slovenia National Hospital Health Care Statistics Database 2013
Ischemic Stroke	National Institute of Public Health (Slovenia). Slovenia National Hospital Health Care Statistics Database 2014
Ischemic Stroke	Bahamas Department of Statistics, Ministry of Health (The Bahamas). Bahamas Living Conditions Survey 2001. Nassau, The Bahamas: Bahamas Department of Statistics
Ischemic Stroke	Agency of the Republic of Kazakhstan on Statistics, World Bank. Kazakhstan Living Standards Measurement Survey 1996. Washington DC, United States: World Bank
Ischemic Stroke	Central Statistical Service (South Africa). South Africa October Household Survey 1996
Ischemic Stroke	World Health Organization (WHO). Belgium World Health Survey 2002. Geneva, Switzerland: World Health Organization (WHO), 2005
Ischemic Stroke	World Health Organization (WHO). China World Health Survey 2002. Geneva, Switzerland: World Health Organization (WHO), 2005
Ischemic Stroke	World Health Organization (WHO). Comoros World Health Survey 2003. Geneva, Switzerland: World Health Organization (WHO), 2005
Ischemic Stroke	World Health Organization (WHO). Croatia World Health Survey 2003. Geneva, Switzerland: World Health Organization (WHO), 2005
Ischemic Stroke	World Health Organization (WHO). Italy World Health Survey 2003. Geneva, Switzerland: World Health Organization (WHO), 2005
Ischemic Stroke	World Health Organization (WHO). Kazakhstan World Health Survey 2002-2003. Geneva, Switzerland: World Health Organization (WHO), 2005
Ischemic Stroke	World Health Organization (WHO). Paraguay World Health Survey 2002-2003. Geneva, Switzerland: World Health Organization (WHO), 2005
Ischemic Stroke	World Health Organization (WHO). Philippines World Health Survey 2003. Geneva, Switzerland: World Health Organization (WHO), 2005
Ischemic Stroke	World Health Organization (WHO). Senegal World Health Survey 2003. Geneva, Switzerland: World Health Organization (WHO), 2005
Ischemic Stroke	World Health Organization (WHO). United Arab Emirates World Health Survey 2003. Geneva, Switzerland: World Health Organization (WHO), 2005
Ischemic Stroke	Ministry of Health (China), National Center for Chronic and Noncommunicable Disease Control and Prevention (China), World Health Organization (WHO). China WHO Study on Global AGEing and Adult Health 2007-2010
Ischemic Stroke	Carolina Population Center, University of North Carolina at Chapel Hill, Chinese Center for Disease Control and Prevention (CCDC). China Health and Nutrition Survey. Chapel Hill, United States: Carolina Population Center, University of North Carolina at Chapel Hill
Ischemic Stroke	Ministry of Public Health (Lebanon), World Health Organization (WHO). Lebanon WHO Multi-country Survey Study on Health and Health System Responsiveness 2000-2001
Ischemic Stroke	Institute of Social Medicine and Health Policy, Shandong University, Shandong University School of Medicine, World Health Organization (WHO). China WHO Multi-country Survey Study on Health and Health System Responsiveness 2000-2001
Ischemic Stroke	Carolei A, Marini C, Di Napoli M, Di Gianfilippo G, Santalucia P, Baldassarre M, Giorgio De Matteis M, di Orio F. High Stroke Incidence in the Prospective Community-Based L'Aquila Registry (1994-1998): First Year's Results. Stroke. 1997; 28(12): 2500-6

Ischemic Stroke	Di Carlo A, Inzitari D, Galati F, Baldereschi M, Giunta V, Grillo G, Furchì A, Manno V, Naso F, Vecchio A, Consoli D. A Prospective Community-Based Study of Stroke in Southern Italy: The Vibo Valentia Incidence of Stroke Study (VISS). Cerebrovasc Dis. 2003; 16(4): 410-7
Ischemic Stroke	Manobianca G, Zoccolella S, Petruzzellis A, Miccoli A, Logroscino G. The incidence of major stroke subtypes in Southern Italy: a population based study. Eur J Neurol. 2010; 17(9): 1148-55
Ischemic Stroke	Corso G, Bottacchi E, Giardini G, De la Pierre F, Meloni T, Pesenti Campagnoni M, Ponzetti C, Veronese Morosini M. Community-based study of stroke incidence in the Valley of Aosta, Italy. CARe-cerebrovascular Aosta Registry: years 2004-2005. Neuroepidemiology. 2009; 32(3): 186-95
Ischemic Stroke	Lauria G, Gentile M, Fassetta G, Casetta I, Agnoli F, Andreotta G, Barp C, Caneve G, Cavallaro A, Cielo R, Mongillo D, Mosca M, Olivieri P. Incidence and Prognosis of Stroke in the Belluno Province, Italy: First-Year Results of a Community-Based Study. Stroke. 1995; 26(10): 1787-93
Ischemic Stroke	D'Alessandro G, Bottacchi E, Di Giovanni M, Martinazzo C, Sironi L, Lia C, Carenini L, Corso G, Gerbaz V, Polillo C, Pesenti Compagnoni M. Temporal trends of stroke in Valle d'Aosta, Italy. Incidence and 30-day fatality rates. Neurol Sci. 2000; 21(1): 13-8
Ischemic Stroke	Manobianca G, Zoccolella S, Petruzzellis A, Miccoli A, Logroscino G. Low Incidence of Stroke in Southern Italy: A Population-Based Study. Stroke. 2008; 39(11): 2923-8
Ischemic Stroke	Hong Y, Bots ML, Pan X, Hofman A, Grobbee DE, Chen H. Stroke Incidence and Mortality in Rural and Urban Shanghai From 1984 Through 1991: Findings From a Community-Based Registry. Stroke. 1994; 25(6): 1165-9
Ischemic Stroke	Zhao D, Liu J, Wang W, Zeng Z, Cheng J, Liu J, Sun J, Wu Z. Epidemiological Transition of Stroke in China Twenty-One-Year Observational Study From the Sino-MONICA-Beijing Project. Stroke. 2008; 39(6): 1668-74
Ischemic Stroke	Central Statistical Service (South Africa). South Africa October Household Survey 1994
Ischemic Stroke	Musolino R LSP. First-ever stroke incidence and 30-day case fatality in the Sicilian Aeolian archipelago, Italy. Stroke. 2005; 36(12): 2738-41
Ischemic Stroke	Orlandi G, Gelli A, Fanucchi S, Tognoni G, Acerbi G, Murri L. Prevalence of stroke and transient ischaemic attack in the elderly population of an Italian rural community. Eur J Epidemiol. 2003; 18(9): 879-82
Ischemic Stroke	Prencipe M, Ferretti C, Casini AR, Santini M, Giubilei F, Culasso F. Stroke, disability, and dementia: results of a population survey. Stroke. 1997; 28(3): 531-6
Ischemic Stroke	Bonzini M, Ferrario MM, Bertù L, Bono G, Vidale S, Veronesi G, Chambless L, Cesana GC. Temporal trends in ischemic and hemorrhagic strokes in Northern Italy: results from the cardiovascular monitoring unit in Northern Italy population-based register, 1998-2004. Neuroepidemiology. 2012; 39(1): 35-42
Ischemic Stroke	Pikija S, Cvetko D, Malojcic B, Trkanjec Z, Pavlicek I, Lukic A, Kopjar A, Hajduk M, Androvic A, Bilic-Genter M, Trkulja V. A population-based prospective 24-month study of stroke: incidence and 30-day case-fatality rates of first-ever strokes in Croatia. Neuroepidemiology. 2012; 38(3): 164-71
Ischemic Stroke	D'Alessandro G, Gallo F, Vitaliano A, Col PD, Gorraz F, Cristofaro RD, Boaretto G. Prevalence of stroke and stroke-related disability in Valle d'Aosta, Italy. Neurol Sci. 2010; 31(2): 137-41
Ischemic Stroke	Zhao Y, Yao Z, D'Souza W, Zhu C, Chun H, Zhuoga C, Zhang Q, Hu X, Zhou D. An Epidemiological Survey of Stroke in Lhasa, Tibet, China. Stroke. 2010; 41(12): 2739-43
Ischemic Stroke	China Center for Economic Research, Peking University. China Health and Retirement Longitudinal Study Pilot Resurvey 2012. Beijing, China: China Center for Economic Research, Peking University
Ischemic Stroke	Sacco S, Stracci F, Cerone D, Ricci S, Carolei A. Epidemiology of stroke in Italy. Int J Stroke. 2011; 6(3): 219-27
Ischemic Stroke	Human Sciences Research Council, South African Medical Research Council. South Africa National Health and Nutrition Examination Survey 2012
Ischemic Stroke	Wu GX, Wu ZS, He BL. [Epidemiological characteristics of stroke in 16 provinces of China]. Nat Med J Chin. 1994; 74: 281–283
Ischemic Stroke	Central American Population Center, University of Costa Rica. Costa Rica Survey of Family Health Services and Expenses 2008. San José, Costa Rica: Central American Population Center, University of Costa Rica
Ischemic Stroke	Gallup Europe, World Health Organization (WHO). United Arab Emirates WHO Multi-country Survey Study on Health and Health System Responsiveness 2000-2001. Geneva, Switzerland: World Health Organization (WHO)
Ischemic Stroke	International Research Associates (INRA) Europe, World Health Organization (WHO). Belgium WHO Multi-country Survey Study on Health and Health System Responsiveness 2000-2001. Geneva, Switzerland: World Health Organization (WHO)
Ischemic Stroke	Gallup Europe, World Health Organization (WHO). Costa Rica WHO Multi-country Survey Study on Health and Health System Responsiveness 2000-2001. Geneva, Switzerland: World Health Organization (WHO)
Ischemic Stroke	Market, Media, and Public Opinion Research (Croatia), World Health Organization (WHO). Croatia WHO Multi-country Survey Study on Health and Health System Responsiveness 2000-2001. Geneva, Switzerland: World Health Organization (WHO)
Ischemic Stroke	International Research Associates (INRA) Europe, World Health Organization (WHO). Italy WHO Multi-country Survey Study on Health and Health System Responsiveness 2000-2001. Geneva, Switzerland: World Health Organization (WHO)
Ischemic Stroke	Börsch-Supan, A. (2015). Survey of Health, Ageing and Retirement in Europe (SHARE) Wave 5. Release version: 1.0.0. SHARE-ERIC. Data set. DOI: 10.6103/SHARE.w5.100
Ischemic Stroke	Börsch-Supan, A. (2015). Survey of Health, Ageing and Retirement in Europe (SHARE) Wave 5. Release version: 1.0.0. SHARE-ERIC. Data set. DOI: 10.6103/SHARE.w5.100
Ischemic Stroke	Center for Study of Public Opinion (Kazakhstan), Concluzia-Prim Center for Survey Methodology (Moldova), Institute for Advanced Studies (Austria), London School of Hygiene and Tropical Medicine, University of Aberdeen. Kazakhstan Health in Times of Transition Household Survey 2010
Ischemic Stroke	Corso G, Bottacchi E, Giardini G, Di Giovanni M, Meloni T, Pesenti Campagnoni M, Veronese Morosini M. Epidemiology of stroke in northern Italy: the Cerebrovascular Aosta Registry, 2004-2008. Neurol Sci. 2013; 34(7): 1071–81
Ischemic Stroke	Kong F-Y, Tao W-D, Hao Z-L, Liu M. Predictors of one-year disability and death in Chinese hospitalized women after ischemic stroke. Cerebrovasc Dis. 2010; 29(3): 255–62
Ischemic Stroke	Wang W, Jiang B, Sun H, Ru X, Sun D, Wang L, Wang L, Jiang Y, Li Y, Wang Y, Chen Z, Wu S, Zhang Y, Wang D, Wang Y, Feigin VL; NESS-China investigators. Prevalence, Incidence and Mortality of Stroke in China: Results from a Nationwide Population-Based Survey of 480,687 Adults. Circulation. 2017; 135(8): 759-71
Ischemic Stroke	Center for Health Statistics and Information, National Health and Family Planning Commission (China). China National Health Statistical Information Reporting System 2013-2015
Ischemic Stroke	Institute for Maternal and Child Health - IRCCS “Burlo Garofolo”. Italy - Friuli Venezia Giulia Hospital Inpatient Discharges 2010-2012
Ischemic Stroke	Institute for Maternal and Child Health - IRCCS “Burlo Garofolo”. Italy - Friuli Venezia Giulia Hospital Inpatient Discharges 2013-2015
Ischemic Stroke	Ricci S, Celani MG, La Rosa F, Vitali R, Duca E, Ferraguzzi R, Paolotti M, Seppoloni D, Caputo N, Chiurulla C, Scaroni R, Signorini E. SEPIVAC: a Community-based Study of Stroke Incidence in Umbria, Italy. J Neurol Neurosurg Psychiatry. 1991; 54(8): 695–8
Ischemic Stroke	Ministry of Health (Italy). Italy National Hospital Discharge Database 2012
Ischemic Stroke	Federal Public Service Health, Food Chain Safety, and Environment (Belgium). Belgium Minimum Clinical Summary 2000
Ischemic Stroke	Federal Public Service Health, Food Chain Safety, and Environment (Belgium). Belgium Minimum Clinical Summary 2001
Ischemic Stroke	Federal Public Service Health, Food Chain Safety, and Environment (Belgium). Belgium Minimum Clinical Summary 2002
Ischemic Stroke	Federal Public Service Health, Food Chain Safety, and Environment (Belgium). Belgium Minimum Clinical Summary 2005
Ischemic Stroke	Federal Public Service Health, Food Chain Safety, and Environment (Belgium). Belgium Minimum Clinical Summary 2006
Ischemic Stroke	Federal Public Service Health, Food Chain Safety, and Environment (Belgium). Belgium Minimum Clinical Summary 2007
Ischemic Stroke	Federal Public Service Health, Food Chain Safety, and Environment (Belgium). Belgium Minimum Hospital Summary 2010

Ischemic Stroke	Federal Public Service Health, Food Chain Safety, and Environment (Belgium). Belgium Minimum Hospital Summary 2011
Ischemic Stroke	Federal Public Service Health, Food Chain Safety, and Environment (Belgium). Belgium Minimum Hospital Summary 2012
Ischemic Stroke	Federal Public Service Health, Food Chain Safety, and Environment (Belgium). Belgium Minimum Hospital Summary 2013
Ischemic Stroke	Ministry of Health (Italy). Italy National Hospital Discharge Database 1970
Ischemic Stroke	Ministry of Health (Italy). Italy National Hospital Discharge Database 1971
Ischemic Stroke	Ministry of Health (Italy). Italy National Hospital Discharge Database 1972
Ischemic Stroke	Ministry of Health (Italy). Italy National Hospital Discharge Database 1973
Ischemic Stroke	Ministry of Health (Italy). Italy National Hospital Discharge Database 1974
Ischemic Stroke	Ministry of Health (Italy). Italy National Hospital Discharge Database 1975
Ischemic Stroke	Ministry of Health (Italy). Italy National Hospital Discharge Database 1976
Ischemic Stroke	Ministry of Health (Italy). Italy National Hospital Discharge Database 1977
Ischemic Stroke	Ministry of Health (Italy). Italy National Hospital Discharge Database 1978
Ischemic Stroke	Ministry of Health (Italy). Italy National Hospital Discharge Database 1979
Ischemic Stroke	Ministry of Health (Italy). Italy National Hospital Discharge Database 1980
Ischemic Stroke	Ministry of Health (Italy). Italy National Hospital Discharge Database 1981
Ischemic Stroke	Ministry of Health (Italy). Italy National Hospital Discharge Database 1982
Ischemic Stroke	Ministry of Health (Italy). Italy National Hospital Discharge Database 1983
Ischemic Stroke	Ministry of Health (Italy). Italy National Hospital Discharge Database 1984
Ischemic Stroke	Ministry of Health (Italy). Italy National Hospital Discharge Database 1985
Ischemic Stroke	Ministry of Health (Italy). Italy National Hospital Discharge Database 1986
Ischemic Stroke	Ministry of Health (Italy). Italy National Hospital Discharge Database 1987
Ischemic Stroke	Ministry of Health (Italy). Italy National Hospital Discharge Database 1988
Ischemic Stroke	Ministry of Health (Italy). Italy National Hospital Discharge Database 1989
Ischemic Stroke	Ministry of Health (Italy). Italy National Hospital Discharge Database 1990
Ischemic Stroke	Ministry of Health (Italy). Italy National Hospital Discharge Database 1991
Ischemic Stroke	Ministry of Health (Italy). Italy National Hospital Discharge Database 1992
Ischemic Stroke	Ministry of Health (Italy). Italy National Hospital Discharge Database 1993
Ischemic Stroke	Ministry of Health (Italy). Italy National Hospital Discharge Database 1994
Ischemic Stroke	Ministry of Health (Italy). Italy National Hospital Discharge Database 1995
Ischemic Stroke	Ministry of Health (Italy). Italy National Hospital Discharge Database 1996
Ischemic Stroke	Ministry of Health (Italy). Italy National Hospital Discharge Database 1997
Ischemic Stroke	Ministry of Health (Italy). Italy National Hospital Discharge Database 1998
Ischemic Stroke	Ministry of Health (Italy). Italy National Hospital Discharge Database 1999
Ischemic Stroke	Ministry of Health (Italy). Italy National Hospital Discharge Database 2000
Ischemic Stroke	Ministry of Health (Italy). Italy National Hospital Discharge Database 2013
Ischemic Stroke	Ministry of Health (Italy). Italy National Hospital Discharge Database 2014
Ischemic Stroke	National Bureau of Statistics of China. China Statistical Yearbook 2015. Beijing, China: National Bureau of Statistics of China
Ischemic Stroke	Ghana Statistical Service. Ghana Living Standards Measurement Survey 1991-1992. Accra, Ghana: Ghana Statistical Service
Ischemic Stroke	Ghana Statistical Service. Ghana Living Standards Survey 1998-1999
Ischemic Stroke	Ghana Statistical Service. Ghana Living Standards Measurement Survey 2005-2006. Accra, Ghana: Ghana Statistical Service
Ischemic Stroke	Institute of Sociology, Russian Academy of Sciences, Paragon Research, University of North Carolina, World Bank. Kyrgyzstan Living Standards Measurement Survey 1993. Washington DC, United States: World Bank
Ischemic Stroke	World Health Organization (WHO). Czech Republic World Health Survey 2002-2003. Geneva, Switzerland: World Health Organization (WHO), 2005
Ischemic Stroke	World Health Organization (WHO). Ghana World Health Survey 2003. Geneva, Switzerland: World Health Organization (WHO), 2005
Ischemic Stroke	World Health Organization (WHO). Luxembourg World Health Survey 2003. Geneva, Switzerland: World Health Organization (WHO), 2005
Ischemic Stroke	World Health Organization (WHO). Sri Lanka World Health Survey 2003. Geneva, Switzerland: World Health Organization (WHO), 2005
Ischemic Stroke	World Health Organization (WHO). Swaziland World Health Survey 2003. Geneva, Switzerland: World Health Organization (WHO), 2005
Ischemic Stroke	University of Wisconsin-Madison, Inter-University Consortium for Political and Social Research (ICPSR), Chronic Disease Research Centre (CDRC), University of the West Indies. Barbados - Bridgetown Survey on Health, Well-Being, and Aging in Latin America and the Caribbean 1999-2000. Ann Arbor, United States: Inter-University
Ischemic Stroke	National Statistical Committee of the Kyrgyz Republic, Research Triangle Institute, Inc. (RTI), World Bank. Kyrgyzstan Living Standards Measurement Survey 1997. Washington DC, United States: World Bank
Ischemic Stroke	National Statistical Committee of the Kyrgyz Republic, Research Triangle Institute, Inc. (RTI), World Bank. Kyrgyzstan Living Standards Measurement Survey 1998. Washington DC, United States: World Bank
Ischemic Stroke	Ashok PP, Radhakrishnan K, Sridharan R, el-Mangoush MA. Incidence and pattern of cerebrovascular diseases in Benghazi, Libya. J Neurol Neurosurg Psychiatr. 1986; 49(5): 519-23
Ischemic Stroke	Morikawa Y, Nakagawa H, Naruse Y, Nishijo M, Miura K, Tabata M, Hirokawa W, Kagamimori S, Honda M, Yoshita K, Hayashi K. Trends in Stroke Incidence and Acute Case Fatality in a Japanese Rural Area: The Oyabe Study. Stroke. 2000; 31(7): 1583-7
Ischemic Stroke	Kita Y, Okayama A, Ueshima H, Wada M, Nozaki A, Choudhary SR, Bonita R, Inamoto Y, Kasamatsu T. Stroke incidence and case fatality in Shiga, Japan 1989-1993. Int J Epidemiol. 1999; 28(6): 1059-65
Ischemic Stroke	Gallup Europe, World Health Organization (WHO). Argentina WHO Multi-country Survey Study on Health and Health System Responsiveness 2000-2001. Geneva, Switzerland: World Health Organization (WHO)
Ischemic Stroke	Kita Y, Turin TC, Ichikawa M, Sugihara H, Morita Y, Tomioka N, Rumana N, Okayama A, Nakamura Y, Abbott RD, Ueshima H. Trend of stroke incidence in a Japanese population: Takashima stroke registry, 1990-2001. Int J Stroke. 2009; 4(4): 241-9
Ischemic Stroke	Kulesh SD, Filina NA, Frantava NM, Zhytko NL, Kastsinevich TM, Kliatskova LA, Shumskas MS, Hilz MJ, Schwab S, Kolominsky-Rabas PL. Incidence and Case-Fatality of Stroke on the East Border of the European Union The Grodno Stroke Study. Stroke. 2010; 41(12): 2726-30
Ischemic Stroke	Melcon CM, Melcon MO. Prevalence of stroke in an Argentine community. Neuroepidemiology. 2006; 27(2): 81-8
Ischemic Stroke	Turin TC, Kita Y, Rumana N, Nakamura Y, Takashima N, Ichikawa M, Sugihara H, Morita Y, Hirose K, Okayama A, Miura K, Ueshima H. Ischemic Stroke Subtypes in a Japanese Population Takashima Stroke Registry, 1988-2004. Stroke. 2010; 41(9): 1871-6
Ischemic Stroke	General Administration of Statistics and Censuses (El Salvador), Ministry of Economy (El Salvador). El Salvador Multipurpose Household Survey 2013. San Salvador, El Salvador: General Administration of Statistics and Censuses (El Salvador)
Ischemic Stroke	Ghana Statistical Service, World Bank. Ghana Living Standards Measurement Survey 2012-2013. Accra, Ghana: Ghana Statistical Service
Ischemic Stroke	MEMRB International, World Health Organization (WHO). Cyprus WHO Multi-country Survey Study on Health and Health System Responsiveness 2000-2001. Geneva, Switzerland: World Health Organization (WHO)

Ischemic Stroke	Institute of Health Information and Statistics of the Czech Republic, International Research Associates (INRA) Europe, World Health Organization (WHO). Czech Republic WHO Multi-country Survey Study on Health and Health System Responsiveness 2000-2001. Geneva, Switzerland: World Health Organization (WHO)
Ischemic Stroke	National Statistical Committee of the Kyrgyz Republic, SIAR Research and Consulting (Kyrgyzstan), World Health Organization (WHO). Kyrgyzstan WHO Multi-country Survey Study on Health and Health System Responsiveness 2000-2001. Geneva, Switzerland: World Health Organization (WHO)
Ischemic Stroke	International Research Associates (INRA) Europe, World Health Organization (WHO). Luxembourg WHO Multi-country Survey Study on Health and Health System Responsiveness 2000-2001. Geneva, Switzerland: World Health Organization (WHO)
Ischemic Stroke	Börsch-Supan, A. (2015). Survey of Health, Ageing and Retirement in Europe (SHARE) Wave 5. Release version: 1.0.0. SHARE-ERIC. Data set. DOI: 10.6103/SHARE.w5.100
Ischemic Stroke	Börsch-Supan, A. (2015). Survey of Health, Ageing and Retirement in Europe (SHARE) Wave 5. Release version: 1.0.0. SHARE-ERIC. Data set. DOI: 10.6103/SHARE.w5.100
Ischemic Stroke	Belarusian State University, Concluzia-Prim Center for Survey Methodology (Moldova), Institute for Advanced Studies (Austria), London School of Hygiene and Tropical Medicine, University of Aberdeen. Belarus Health in Times of Transition Household Survey 2010
Ischemic Stroke	Studies (Austria), International Centre for Sociological, Political and Social Psychological Research (Kyrgyzstan), London School of Hygiene and Tropical Medicine, University of Aberdeen. Kyrgyzstan Health in Times of Transition Household Survey 2011
Ischemic Stroke	General Administration of Statistics and Censuses (El Salvador), Ministry of Economy (El Salvador). El Salvador Multipurpose Household Survey 2014. San Salvador, El Salvador: General Administration of Statistics and Censuses (El Salvador)
Ischemic Stroke	Hattori N, Hirayama T, Katayama Y. Medical care for chronic-phase stroke in Japan. Neurol Med Chir (Tokyo). 2012; 52(4): 175–80
Ischemic Stroke	Department of Economics, University of Chile, Ministry of Planning (Chile). Chile National Socioeconomic Characterization Survey 1992
Ischemic Stroke	Department of Economics, University of Chile, Ministry of Planning (Chile). Chile National Socioeconomic Characterization Survey 1994. Santiago, Chile: Ministry of Social Development (Chile)
Ischemic Stroke	Department of Economics, University of Chile, Ministry of Planning (Chile). Chile National Socioeconomic Characterization Survey 1996. Santiago, Chile: Ministry of Social Development (Chile)
Ischemic Stroke	Department of Economics, University of Chile, Ministry of Planning (Chile). Chile National Socioeconomic Characterization Survey 1998
Ischemic Stroke	Department of Economics, University of Chile, Ministry of Planning (Chile). Chile National Socioeconomic Characterization Survey 2000. Santiago, Chile: Ministry of Social Development (Chile)
Ischemic Stroke	Ministry of Public Health (Thailand). Thailand National Health and Examination Survey 2003-2004
Ischemic Stroke	World Health Organization (WHO). Denmark World Health Survey 2003. Geneva, Switzerland: World Health Organization (WHO), 2005
Ischemic Stroke	World Health Organization (WHO). Estonia World Health Survey 2003. Geneva, Switzerland: World Health Organization (WHO), 2005
Ischemic Stroke	World Health Organization (WHO). Guatemala World Health Survey 2003. Geneva, Switzerland: World Health Organization (WHO), 2005
Ischemic Stroke	World Health Organization (WHO). Hungary World Health Survey 2003. Geneva, Switzerland: World Health Organization (WHO), 2005
Ischemic Stroke	World Health Organization (WHO). Morocco World Health Survey 2003. Geneva, Switzerland: World Health Organization (WHO), 2005
Ischemic Stroke	World Health Organization (WHO). Zimbabwe World Health Survey 2003. Geneva, Switzerland: World Health Organization (WHO), 2005
Ischemic Stroke	Department of Economics, University of Chile, Ministry of Planning (Chile). Chile National Socioeconomic Characterization Survey 2003. Santiago, Chile: Ministry of Social Development (Chile)
Ischemic Stroke	University of Wisconsin-Madison, Inter-University Consortium for Political and Social Research (ICPSR), Institute of Nutrition and Food Technology (INTA), University of Chile, Center for Geriatrics and Gerontology, Pontifical Catholic University of Chile. Chile - Santiago Survey on Health, Well-Being, and Aging in Latin America and the
Ischemic Stroke	Directorate of Statistics of the High Commission for Planning (Morocco), World Bank. Morocco Living Standards Measurement Survey 1990-1991
Ischemic Stroke	Department of Economics, University of Chile, Ministry of Planning (Chile). Chile National Socioeconomic Characterization Survey 2006. Santiago, Chile: Ministry of Social Development (Chile)
Ischemic Stroke	Jorgensen HS, Plesner AM, Hubbe P, Larsen K. Marked increase of stroke incidence in men between 1972 and 1990 in Frederiksberg, Denmark. Stroke. 1992; 23(12): 1701-4
Ischemic Stroke	Lavados PM, Sacks C, Prina L, Escobar A, Tossi C, Araya F, Feuerhake W, Galvez M, Salinas R, Alvarez G. Incidence, 30-day case-fatality rate, and prognosis of stroke in Iquique, Chile: a 2-year community-based prospective study (PISCIS project). Lancet. 2005; 365(9478): 2206-15
Ischemic Stroke	Vibo R, Kõrv J, Roose M. The Third Stroke Registry in Tartu, Estonia: Decline of Stroke Incidence and 28-Day Case-Fatality Rate Since 1991. Stroke. 2005; 36(12): 2544-8
Ischemic Stroke	Health Promotion Research Institute (Hungary), Hungarian Gallup Institute. Hungary National Population Health Survey 2000
Ischemic Stroke	Kôrv J, Roose M, Kaasik A-E. Changed Incidence and Case-Fatality Rates of First-Ever Stroke Between 1970 and 1993 in Tartu, Estonia. Stroke. 1996; 27(2): 199-203
Ischemic Stroke	Matenga J. Stroke incidence rates among black residents of Harare - a prospective community-based study. S Afr Med J. 1997; 87(5): 606-8
Ischemic Stroke	Fuh JL, Wang SJ, Larson EB, Liu HC. Prevalence of stroke in Kinmen. Stroke. 1996; 27(8): 1338-41
Ischemic Stroke	Huang Z-S, Chiang T-L, Lee T-K. Stroke Prevalence in Taiwan: Findings From the 1994 National Health Interview Survey. Stroke. 1997; 28(8): 1579-84
Ischemic Stroke	Ministry of Health (Chile). Chile Hospital Discharges 2001. Santiago, Chile: Ministry of Health (Chile)
Ischemic Stroke	Ministry of Health (Chile). Chile Hospital Discharges 2002. Santiago, Chile: Ministry of Health (Chile)
Ischemic Stroke	Ministry of Health (Chile). Chile Hospital Discharges 2003. Santiago, Chile: Ministry of Health (Chile)
Ischemic Stroke	Ministry of Health (Chile). Chile Hospital Discharges 2004. Santiago, Chile: Ministry of Health (Chile)
Ischemic Stroke	Ministry of Health (Chile). Chile Hospital Discharges 2005. Santiago, Chile: Ministry of Health (Chile)
Ischemic Stroke	Ministry of Health (Chile). Chile Hospital Discharges 2006. Santiago, Chile: Ministry of Health (Chile)
Ischemic Stroke	Ministry of Health (Chile). Chile Hospital Discharges 2007. Santiago, Chile: Ministry of Health (Chile)
Ischemic Stroke	Ministry of Health (Chile). Chile Hospital Discharges 2008. Santiago, Chile: Ministry of Health (Chile)
Ischemic Stroke	Ministry of Health (Chile). Chile Hospital Discharges 2009. Santiago, Chile: Ministry of Health (Chile)
Ischemic Stroke	Ministry of Health (Chile). Chile Hospital Discharges 2010. Santiago, Chile: Ministry of Health (Chile)
Ischemic Stroke	Ministry of Health (Chile). Chile Hospital Discharges 2011. Santiago, Chile: Ministry of Health (Chile)
Ischemic Stroke	Ministry of Planning (Chile), Social Observatory, Alberto Hurtado University. Chile National Socioeconomic Characterization Survey 2009. Santiago, Chile: Ministry of Social Development (Chile)
Ischemic Stroke	Korea Centers for Disease Control and Prevention. South Korea National Health and Nutrition Examination Survey 2012
Ischemic Stroke	Competence Centre for Clinical Quality and Health Informatics West (Denmark). Danish Stroke Registry Data 2009. [Unpublished]
Ischemic Stroke	Hu HH, Sheng WY, Chu FL, Lan CF, Chiang BN. Incidence of stroke in Taiwan. Stroke. 1992; 23: 1237–1241
Ischemic Stroke	Ministry of Health (Chile). Chile Hospital Discharges 2012. Santiago, Chile: Ministry of Health (Chile)
Ischemic Stroke	University of Concepcion (Chile), World Health Organization (WHO). Chile WHO Multi-country Survey Study on Health and Health System Responsiveness 2000-2001. Geneva, Switzerland: World Health Organization (WHO)
Ischemic Stroke	Statistics Denmark, World Health Organization (WHO). Denmark WHO Multi-country Survey Study on Health and Health System Responsiveness 2000-2001. Geneva, Switzerland: World Health Organization (WHO)

Ischemic Stroke	International Research Associates (INRA) Europe, World Health Organization (WHO). Estonia WHO Multi-country Survey Study on Health and Health System Responsiveness 2000-2001. Geneva, Switzerland: World Health Organization (WHO)
Ischemic Stroke	Szonda Ipsos, World Health Organization (WHO). Hungary WHO Multi-country Survey Study on Health and Health System Responsiveness 2000-2001. Geneva, Switzerland: World Health Organization (WHO)
Ischemic Stroke	Graduate School of Public Health, Seoul National University, World Health Organization (WHO). South Korea WHO Multi-country Survey Study on Health and Health System Responsiveness 2000-2001. Geneva, Switzerland: World Health Organization (WHO)
Ischemic Stroke	Gallup Europe, World Health Organization (WHO). Morocco WHO Multi-country Survey Study on Health and Health System Responsiveness 2000-2001. Geneva, Switzerland: World Health Organization (WHO)
Ischemic Stroke	International Research Associates (INRA) Europe, World Health Organization (WHO). Malta WHO Multi-country Survey Study on Health and Health System Responsiveness 2000-2001. Geneva, Switzerland: World Health Organization (WHO)
Ischemic Stroke	Mahidol University, World Health Organization (WHO). Thailand WHO Multi-country Survey Study on Health and Health System Responsiveness 2000-2001. Geneva, Switzerland: World Health Organization (WHO)
Ischemic Stroke	Börsch-Supan, A. (2015). Survey of Health, Ageing and Retirement in Europe (SHARE) Wave 5. Release version: 1.0.0. SHARE-ERIC. Data set. DOI: 10.6103/SHARE.w5.100
Ischemic Stroke	Börsch-Supan, A. (2015). Survey of Health, Ageing and Retirement in Europe (SHARE) Wave 5. Release version: 1.0.0. SHARE-ERIC. Data set. DOI: 10.6103/SHARE.w5.100
Ischemic Stroke	National Office of Statistics (Cuba). Cuba Statistical Yearbook 2012. Havana, Cuba: National Office of Statistics (Cuba)
Ischemic Stroke	Kim J-M, Stewart R, Park M-S, Kang H-J, Kim S-W, Shin I-S, Kim H-R, Shin M-G, Cho K-H, Yoon J-S. Associations of BDNF genotype and promoter methylation with acute and long-term stroke outcomes in an East Asian cohort. PLoS One. 2012; 7(12): e51280
Ischemic Stroke	Vibo R, Korv J, Roose M. One-year outcome after first-ever stroke according to stroke subtype, severity, risk factors and pre-stroke treatment. A population-based study from Tartu, Estonia. Eur J Neurol. 2007; 14(4): 435–9
Ischemic Stroke	Kang H-J, Stewart R, Park M-S, Bae K-Y, Kim S-W, Kim J-M, Shin I-S, Cho K-H, Yoon J-S. White matter hyperintensities and functional outcomes at 2 weeks and 1 year after stroke. Cerebrovasc Dis. 2013; 35(2): 138–45
Ischemic Stroke	Truelsen T, Gronbaek M, Schnohr P, Boysen G. Stroke case fatality in Denmark from 1977 to 1992: the Copenhagen City Heart Study. Neuroepidemiology. 2002; 21(1): 22–7
Ischemic Stroke	Danish Health and Medicines Authority. Denmark National Patient Registry 2003
Ischemic Stroke	Danish Health and Medicines Authority. Denmark National Patient Registry 2004
Ischemic Stroke	Danish Health and Medicines Authority. Denmark National Patient Registry 2005
Ischemic Stroke	Danish Health and Medicines Authority. Denmark National Patient Registry 2006
Ischemic Stroke	Danish Health and Medicines Authority. Denmark National Patient Registry 2007
Ischemic Stroke	Danish Health and Medicines Authority. Denmark National Patient Registry 2008
Ischemic Stroke	Danish Health and Medicines Authority. Denmark National Patient Registry 2009
Ischemic Stroke	Ministry of Social Affairs (Estonia), National Institute for Health Development (Estonia). Estonia Hospital Inpatient Discharges 2003
Ischemic Stroke	Ministry of Social Affairs (Estonia), National Institute for Health Development (Estonia). Estonia Hospital Inpatient Discharges 2004
Ischemic Stroke	Ministry of Social Affairs (Estonia), National Institute for Health Development (Estonia). Estonia Hospital Inpatient Discharges 2005
Ischemic Stroke	Ministry of Social Affairs (Estonia), National Institute for Health Development (Estonia). Estonia Hospital Inpatient Discharges 2006
Ischemic Stroke	Ministry of Social Affairs (Estonia), National Institute for Health Development (Estonia). Estonia Hospital Inpatient Discharges 2007
Ischemic Stroke	Ministry of Social Affairs (Estonia), National Institute for Health Development (Estonia). Estonia Hospital Inpatient Discharges 2008
Ischemic Stroke	Ministry of Social Affairs (Estonia), National Institute for Health Development (Estonia). Estonia Hospital Inpatient Discharges 2009
Ischemic Stroke	Ministry of Social Affairs (Estonia), National Institute for Health Development (Estonia). Estonia Hospital Inpatient Discharges 2010
Ischemic Stroke	Ministry of Social Affairs (Estonia), National Institute for Health Development (Estonia). Estonia Hospital Inpatient Discharges 2011
Ischemic Stroke	Center for Health Care Information (GYOGYINFOK) (Hungary). Hungary Hospital Inpatient Discharges 1990
Ischemic Stroke	National Institute for Health Development (Estonia). Estonia Hospital Inpatient Discharges 2012
Ischemic Stroke	National Institute for Health Development (Estonia). Estonia Hospital Inpatient Discharges 2013
Ischemic Stroke	National Institute for Health Development (Estonia). Estonia Hospital Inpatient Discharges 2014
Ischemic Stroke	Institute of Experimental and Clinical Medicine (Estonia). Estonia Hospital Inpatient Discharges 1999
Ischemic Stroke	Institute of Experimental and Clinical Medicine (Estonia). Estonia Hospital Inpatient Discharges 1998
Ischemic Stroke	Institute of Experimental and Clinical Medicine (Estonia). Estonia Hospital Inpatient Discharges 1997
Ischemic Stroke	Institute of Experimental and Clinical Medicine (Estonia). Estonia Hospital Inpatient Discharges 1996
Ischemic Stroke	Institute of Experimental and Clinical Medicine (Estonia). Estonia Hospital Inpatient Discharges 1995
Ischemic Stroke	Institute of Experimental and Clinical Medicine (Estonia). Estonia Hospital Inpatient Discharges 1994
Ischemic Stroke	Institute of Experimental and Clinical Medicine (Estonia). Estonia Hospital Inpatient Discharges 1993
Ischemic Stroke	Institute of Experimental and Clinical Medicine (Estonia). Estonia Hospital Inpatient Discharges 1992
Ischemic Stroke	Institute of Experimental and Clinical Medicine (Estonia). Estonia Hospital Inpatient Discharges 1991
Ischemic Stroke	Institute of Experimental and Clinical Medicine (Estonia). Estonia Hospital Inpatient Discharges 1990
Ischemic Stroke	Institute of Experimental and Clinical Medicine (Estonia). Estonia Hospital Inpatient Discharges 1989
Ischemic Stroke	Institute of Experimental and Clinical Medicine (Estonia). Estonia Hospital Inpatient Discharges 1988
Ischemic Stroke	Institute of Experimental and Clinical Medicine (Estonia). Estonia Hospital Inpatient Discharges 1987
Ischemic Stroke	Institute of Experimental and Clinical Medicine (Estonia). Estonia Hospital Inpatient Discharges 1986
Ischemic Stroke	Institute of Experimental and Clinical Medicine (Estonia). Estonia Hospital Inpatient Discharges 1985
Ischemic Stroke	Institute of Experimental and Clinical Medicine (Estonia). Estonia Hospital Inpatient Discharges 1980
Ischemic Stroke	Center for Health Care Information (GYOGYINFOK) (Hungary). Hungary Hospital Inpatient Discharges 1991
Ischemic Stroke	Center for Health Care Information (GYOGYINFOK) (Hungary). Hungary Hospital Inpatient Discharges 1992
Ischemic Stroke	Center for Health Care Information (GYOGYINFOK) (Hungary). Hungary Hospital Inpatient Discharges 1993
Ischemic Stroke	Center for Health Care Information (GYOGYINFOK) (Hungary). Hungary Hospital Inpatient Discharges 1994
Ischemic Stroke	Center for Health Care Information (GYOGYINFOK) (Hungary). Hungary Hospital Inpatient Discharges 1995
Ischemic Stroke	Center for Health Care Information (GYOGYINFOK) (Hungary). Hungary Hospital Inpatient Discharges 1996
Ischemic Stroke	Center for Health Care Information (GYOGYINFOK) (Hungary). Hungary Hospital Inpatient Discharges 1997
Ischemic Stroke	Center for Health Care Information (GYOGYINFOK) (Hungary). Hungary Hospital Inpatient Discharges 1998
Ischemic Stroke	Center for Health Care Information (GYOGYINFOK) (Hungary). Hungary Hospital Inpatient Discharges 1999
Ischemic Stroke	Center for Health Care Information (GYOGYINFOK) (Hungary). Hungary Hospital Inpatient Discharges 2000
Ischemic Stroke	Center for Health Care Information (GYOGYINFOK) (Hungary). Hungary Hospital Inpatient Discharges 2001
Ischemic Stroke	Center for Health Care Information (GYOGYINFOK) (Hungary). Hungary Hospital Inpatient Discharges 2002
Ischemic Stroke	Center for Health Care Information (GYOGYINFOK) (Hungary). Hungary Hospital Inpatient Discharges 2003
Ischemic Stroke	National Institute for Strategic Health Research (ESKI) (Hungary). Hungary Hospital Inpatient Discharges 2013

Ischemic Stroke	National Institute for Strategic Health Research (ESKI) (Hungary). Hungary Hospital Inpatient Discharges 2014
Ischemic Stroke	Danish Health and Medicines Authority. Denmark National Patient Registry 1979
Ischemic Stroke	Danish Health and Medicines Authority. Denmark National Patient Registry 1980
Ischemic Stroke	Danish Health and Medicines Authority. Denmark National Patient Registry 1981
Ischemic Stroke	Danish Health and Medicines Authority. Denmark National Patient Registry 1982
Ischemic Stroke	Danish Health and Medicines Authority. Denmark National Patient Registry 1983
Ischemic Stroke	Danish Health and Medicines Authority. Denmark National Patient Registry 1984
Ischemic Stroke	Danish Health and Medicines Authority. Denmark National Patient Registry 1985
Ischemic Stroke	Danish Health and Medicines Authority. Denmark National Patient Registry 1986
Ischemic Stroke	Danish Health and Medicines Authority. Denmark National Patient Registry 1987
Ischemic Stroke	Danish Health and Medicines Authority. Denmark National Patient Registry 1988
Ischemic Stroke	Danish Health and Medicines Authority. Denmark National Patient Registry 1989
Ischemic Stroke	Danish Health and Medicines Authority. Denmark National Patient Registry 1990
Ischemic Stroke	Danish Health and Medicines Authority. Denmark National Patient Registry 1991
Ischemic Stroke	Danish Health and Medicines Authority. Denmark National Patient Registry 1992
Ischemic Stroke	Danish Health and Medicines Authority. Denmark National Patient Registry 1993
Ischemic Stroke	Danish Health and Medicines Authority. Denmark National Patient Registry 1994
Ischemic Stroke	Danish Health and Medicines Authority. Denmark National Patient Registry 1995
Ischemic Stroke	Danish Health and Medicines Authority. Denmark National Patient Registry 1996
Ischemic Stroke	Danish Health and Medicines Authority. Denmark National Patient Registry 1997
Ischemic Stroke	Danish Health and Medicines Authority. Denmark National Patient Registry 1998
Ischemic Stroke	Danish Health and Medicines Authority. Denmark National Patient Registry 1999
Ischemic Stroke	Danish Health and Medicines Authority. Denmark National Patient Registry 2000
Ischemic Stroke	Danish Health and Medicines Authority. Denmark National Patient Registry 2001
Ischemic Stroke	Danish Health and Medicines Authority. Denmark National Patient Registry 2002
Ischemic Stroke	Danish Health and Medicines Authority. Denmark National Patient Registry 2010
Ischemic Stroke	Danish Health and Medicines Authority. Denmark National Patient Registry 2011
Ischemic Stroke	Danish Health and Medicines Authority. Denmark National Patient Registry 2012
Ischemic Stroke	Danish Health and Medicines Authority. Denmark National Patient Registry 2013
Ischemic Stroke	National State Statistical Agency (Tajikistan), World Bank. Tajikistan Living Standards Measurement Survey 2003
Ischemic Stroke	National State Statistical Agency (Tajikistan), World Bank. Tajikistan Living Standards Measurement Survey 2007
Ischemic Stroke	Planning Commission (Tanzania), University of Dar es Salaam, World Bank. Tanzania Living Standards Measurement Study 1993-1994. Washington DC, United States: World Bank
Ischemic Stroke	National Statistics Directorate (Timor-Leste), World Bank. Timor-Leste Living Standards and Measurement Survey 2001. Washington DC, United States: World Bank
Ischemic Stroke	Palestinian Central Bureau of Statistics. Palestine Demographic and Health Survey 2004
Ischemic Stroke	World Health Organization (WHO). Ethiopia World Health Survey 2003. Geneva, Switzerland: World Health Organization (WHO), 2005
Ischemic Stroke	World Health Organization (WHO). Finland World Health Survey 2004. Geneva, Switzerland: World Health Organization (WHO), 2005
Ischemic Stroke	World Health Organization (WHO). Latvia World Health Survey 2003. Geneva, Switzerland: World Health Organization (WHO), 2005
Ischemic Stroke	World Health Organization (WHO). Netherlands World Health Survey 2004. Geneva, Switzerland: World Health Organization (WHO), 2005
Ischemic Stroke	World Health Organization (WHO). Uruguay World Health Survey 2002-2003. Geneva, Switzerland: World Health Organization (WHO), 2005
Ischemic Stroke	National State Statistical Agency (Tajikistan), World Bank. Tajikistan Living Standards Measurement Survey 2009
Ischemic Stroke	National Statistics Directorate (Timor-Leste), World Bank. Timor-Leste Living Standards and Measurement Survey 2007-2008. Washington DC, United States: World Bank
Ischemic Stroke	National Bureau of Statistics (Tanzania). Tanzania Living Standards Measurement Study - Integrated Surveys on Agriculture 2010-2011. Dar es Salaam, Tanzania: National Bureau of Statistics (Tanzania)
Ischemic Stroke	Numminen H, Kotila M, Waltimo O, Aho K, Kaste M. Declining Incidence and Mortality Rates of Stroke in Finland From 1972 to 1991: Results of Three Population-Based Stroke Registers. Stroke. 1996; 27(9): 1487-91
Ischemic Stroke	Immonen-Räihä P, Mähönen M, Tuomilehto J, Salomaa V, Kaarsalo E, Narva EV, Salmi K, Sarti C, Sivenius J, Alhainen K, Torppa J. Trends in Case-Fatality of Stroke in Finland During 1983 to 1992. Stroke. 1997; 28(12): 2493-9
Ischemic Stroke	Vaartjes I, Reitsma JB, de Bruin A, Berger-van Sijl M, Bos MJ, Breteler MM, Grobbee DE, Bots ML. Nationwide incidence of first stroke and TIA in the Netherlands. Eur J Neurol. 2008; 15(12): 1315-23
Ischemic Stroke	Walker R, Unwin N, Mugusi F, Swai M, Aris E, Jusabani A, Kabadi G, Gray W, Lewanga M, Alberti G, Whiting D. Stroke incidence in rural and urban Tanzania: a prospective, community-based study. Lancet Neurol. 2010; 9(8): 786-92
Ischemic Stroke	Sweileh WM, Sawalha AF, Al-Aqad SM, Zyoud SH, Al-Jabi SW. The Epidemiology of Stroke in Northern Palestine: A 1-Year, Hospital-Based Study. J Stroke Cerebrovasc Dis. 2008; 17(6): 406-11
Ischemic Stroke	De Jesús Llibre J, Valhuerdi A, Fernández O, Llibre JC, Porto R, López AM, Marcheco B, Moreno C. Prevalence of stroke and associated risk factors in older adults in Havana City and Matanzas Provinces, Cuba (10/66 population-based study). MEDICC Rev. 2010; 12(3): 20-6
Ischemic Stroke	Tekle-Haimanot R, Abebe M, Gebre-Mariam A, Forsgren L, Heijbel J, Holmgren G, Ekstedt J. Community-based study of neurological disorders in rural central Ethiopia. Neuroepidemiology. 1990; 9(5): 263-77
Ischemic Stroke	Venketasubramanian N, Tan LCS, Sahadevan S, Chin JJ, Krishnamoorthy ES, Hong CY, Saw SM. Prevalence of Stroke Among Chinese, Malay, and Indian Singaporeans. Stroke. 2005; 36(3): 551-6
Ischemic Stroke	Walker R, McLarty D, Masuki G, Kitange H, Whiting D, Moshi A, Massawe J, Amaro R, Mhina A, Alberti K. Age specific prevalence of impairment and disability relating to hemiplegic stroke in the Hai District of northern Tanzania. J Neurol Neurosurg Psychiatr. 2000; 68(6): 744-9
Ischemic Stroke	Wieberdink RG, Ikram MA, Hofman A, Koudstaal PJ, Breteler MMB. Trends in stroke incidence rates and stroke risk factors in Rotterdam, the Netherlands from 1990 to 2008. Eur J Epidemiol. 2012; 27(4): 287-95
Ischemic Stroke	Dutch Hospital Data (DHD). Netherlands National Medical Registry 1998
Ischemic Stroke	Dutch Hospital Data (DHD). Netherlands National Medical Registry 1999
Ischemic Stroke	Dutch Hospital Data (DHD). Netherlands National Medical Registry 2000
Ischemic Stroke	Dutch Hospital Data (DHD). Netherlands National Medical Registry 2001
Ischemic Stroke	Dutch Hospital Data (DHD). Netherlands National Medical Registry 2002
Ischemic Stroke	Dutch Hospital Data (DHD). Netherlands National Medical Registry 2003
Ischemic Stroke	Dutch Hospital Data (DHD). Netherlands National Medical Registry 2004
Ischemic Stroke	Dutch Hospital Data (DHD). Netherlands National Medical Registry 2006
Ischemic Stroke	Dutch Hospital Data (DHD). Netherlands National Medical Registry 2007

Ischemic Stroke	Dutch Hospital Data (DHD). Netherlands National Medical Registry 2010
Ischemic Stroke	Development Center for Welfare and Health (STAKES) (Finland), World Health Organization (WHO). Finland WHO Multi-country Survey Study on Health and Health System Responsiveness 2000-2001. Geneva, Switzerland: World Health Organization (WHO)
Ischemic Stroke	Gallup Europe, World Health Organization (WHO). Latvia WHO Multi-country Survey Study on Health and Health System Responsiveness 2000-2001. Geneva, Switzerland: World Health Organization (WHO)
Ischemic Stroke	International Research Associates (INRA) Europe, Netherlands Organisation for Applied Scientific Research (TNO), World Health Organization (WHO). Netherlands WHO Multi-country Survey Study on Health and Health System Responsiveness 2000-2001. Geneva, Switzerland: World Health Organization (WHO)
Ischemic Stroke	University of the West Indies, World Health Organization (WHO). Trinidad and Tobago WHO Multi-country Survey Study on Health and Health System Responsiveness 2000-2001. Geneva, Switzerland: World Health Organization (WHO)
Ischemic Stroke	Börsch-Supan, A. (2015). Survey of Health, Ageing and Retirement in Europe (SHARE) Wave 5. Release version: 1.0.0. SHARE-ERIC. Data set. DOI: 10.6103/SHARE.w5.100
Ischemic Stroke	Centre for Disease Prevention and Control (Latvia), Riga Stradi?š University. Latvia Health Behavior Among the Adult Population 2014
Ischemic Stroke	Rastas S, Verkkoniemi A, Polvikoski T, Juva K, Niinisto L, Mattila K, Lansimies E, Pirttila T, Sulkava R. Atrial fibrillation, stroke, and cognition: a longitudinal population-based study of people aged 85 and older. Stroke. 2007; 38(5): 1454–60
Ischemic Stroke	Ministry of Health of the Republic of Latvia. Latvia Hospital Inpatient Discharges 2004
Ischemic Stroke	Ministry of Health of the Republic of Latvia. Latvia Hospital Inpatient Discharges 2006
Ischemic Stroke	Ministry of Health of the Republic of Latvia. Latvia Hospital Inpatient Discharges 2007
Ischemic Stroke	Ministry of Health of the Republic of Latvia. Latvia Hospital Inpatient Discharges 2008
Ischemic Stroke	Ministry of Health of the Republic of Latvia. Latvia Hospital Inpatient Discharges 2010
Ischemic Stroke	Ministry of Health of the Republic of Latvia, National Health Service (Latvia). Latvia Hospital Inpatient Discharges 2011
Ischemic Stroke	Ministry of Health of the Republic of Latvia, National Health Service (Latvia). Latvia Hospital Inpatient Discharges 2012
Ischemic Stroke	Institute of Public Health (Macedonia). Macedonia Hospital Inpatient Discharges 2000
Ischemic Stroke	Institute of Public Health (Macedonia). Macedonia Hospital Inpatient Discharges 2001
Ischemic Stroke	Institute of Public Health (Macedonia). Macedonia Hospital Inpatient Discharges 2002
Ischemic Stroke	Institute of Public Health (Macedonia). Macedonia Hospital Inpatient Discharges 2004
Ischemic Stroke	National Public Health Institute (Finland). Finland Hospital Discharge Register 1988
Ischemic Stroke	National Public Health Institute (Finland). Finland Hospital Discharge Register 1989
Ischemic Stroke	National Public Health Institute (Finland). Finland Hospital Discharge Register 1990
Ischemic Stroke	National Public Health Institute (Finland). Finland Hospital Discharge Register 1991
Ischemic Stroke	National Public Health Institute (Finland). Finland Hospital Discharge Register 1992
Ischemic Stroke	National Public Health Institute (Finland). Finland Hospital Discharge Register 1993
Ischemic Stroke	Ministry of Health of the Republic of Latvia. Latvia Hospital Inpatient Discharges 1980
Ischemic Stroke	Institute of Public Health (Macedonia). Macedonia Hospital Inpatient Discharges 1980
Ischemic Stroke	Dutch Hospital Data (DHD). Netherlands National Medical Registry 1990
Ischemic Stroke	Dutch Hospital Data (DHD). Netherlands National Medical Registry 1991
Ischemic Stroke	Dutch Hospital Data (DHD). Netherlands National Medical Registry 1992
Ischemic Stroke	Dutch Hospital Data (DHD). Netherlands National Medical Registry 1993
Ischemic Stroke	Dutch Hospital Data (DHD). Netherlands National Medical Registry 1994
Ischemic Stroke	Dutch Hospital Data (DHD). Netherlands National Medical Registry 1995
Ischemic Stroke	Dutch Hospital Data (DHD). Netherlands National Medical Registry 1996
Ischemic Stroke	Dutch Hospital Data (DHD). Netherlands National Medical Registry 1997
Ischemic Stroke	National Public Health Institute (Finland). Finland Hospital Discharge Register 1994
Ischemic Stroke	National Public Health Institute (Finland). Finland Hospital Discharge Register 1995
Ischemic Stroke	National Public Health Institute (Finland). Finland Hospital Discharge Register 1996
Ischemic Stroke	National Public Health Institute (Finland). Finland Hospital Discharge Register 1997
Ischemic Stroke	National Public Health Institute (Finland). Finland Hospital Discharge Register 1998
Ischemic Stroke	National Public Health Institute (Finland). Finland Hospital Discharge Register 1999
Ischemic Stroke	National Institute for Health and Welfare (THL) (Finland). Finland Hospital Discharge Register 2013
Ischemic Stroke	National Institute for Health and Welfare (THL) (Finland). Finland Hospital Discharge Register 2014
Ischemic Stroke	Ministry of Health of the Republic of Latvia. Latvia Hospital Inpatient Discharges 1981
Ischemic Stroke	Ministry of Health of the Republic of Latvia. Latvia Hospital Inpatient Discharges 1982
Ischemic Stroke	Ministry of Health of the Republic of Latvia. Latvia Hospital Inpatient Discharges 1983
Ischemic Stroke	Ministry of Health of the Republic of Latvia. Latvia Hospital Inpatient Discharges 1984
Ischemic Stroke	Ministry of Health of the Republic of Latvia. Latvia Hospital Inpatient Discharges 1985
Ischemic Stroke	Ministry of Health of the Republic of Latvia. Latvia Hospital Inpatient Discharges 1986
Ischemic Stroke	Ministry of Health of the Republic of Latvia. Latvia Hospital Inpatient Discharges 1987
Ischemic Stroke	Ministry of Health of the Republic of Latvia. Latvia Hospital Inpatient Discharges 1988
Ischemic Stroke	Ministry of Health of the Republic of Latvia. Latvia Hospital Inpatient Discharges 1989
Ischemic Stroke	Ministry of Health of the Republic of Latvia. Latvia Hospital Inpatient Discharges 1990
Ischemic Stroke	Ministry of Health of the Republic of Latvia. Latvia Hospital Inpatient Discharges 1991
Ischemic Stroke	Ministry of Health of the Republic of Latvia. Latvia Hospital Inpatient Discharges 1992
Ischemic Stroke	Ministry of Health of the Republic of Latvia. Latvia Hospital Inpatient Discharges 1993
Ischemic Stroke	Ministry of Health of the Republic of Latvia. Latvia Hospital Inpatient Discharges 1994
Ischemic Stroke	Ministry of Health of the Republic of Latvia. Latvia Hospital Inpatient Discharges 1995
Ischemic Stroke	Ministry of Health of the Republic of Latvia. Latvia Hospital Inpatient Discharges 1996
Ischemic Stroke	Ministry of Health of the Republic of Latvia. Latvia Hospital Inpatient Discharges 1997
Ischemic Stroke	Ministry of Health of the Republic of Latvia. Latvia Hospital Inpatient Discharges 1998
Ischemic Stroke	Ministry of Health of the Republic of Latvia. Latvia Hospital Inpatient Discharges 1999
Ischemic Stroke	Ministry of Health of the Republic of Latvia. Latvia Hospital Inpatient Discharges 2000
Ischemic Stroke	Ministry of Health of the Republic of Latvia. Latvia Hospital Inpatient Discharges 2001
Ischemic Stroke	Ministry of Health of the Republic of Latvia. Latvia Hospital Inpatient Discharges 2002
Ischemic Stroke	Ministry of Health of the Republic of Latvia. Latvia Hospital Inpatient Discharges 2003
Ischemic Stroke	Ministry of Health of the Republic of Latvia, National Health Service (Latvia). Latvia Hospital Inpatient Discharges 2013
Ischemic Stroke	Ministry of Health of the Republic of Latvia, National Health Service (Latvia). Latvia Hospital Inpatient Discharges 2014

Ischemic Stroke	Institute of Public Health (Macedonia). Macedonia Hospital Inpatient Discharges 1982
Ischemic Stroke	Institute of Public Health (Macedonia). Macedonia Hospital Inpatient Discharges 1984
Ischemic Stroke	Institute of Public Health (Macedonia). Macedonia Hospital Inpatient Discharges 1985
Ischemic Stroke	Institute of Public Health (Macedonia). Macedonia Hospital Inpatient Discharges 1986
Ischemic Stroke	Institute of Public Health (Macedonia). Macedonia Hospital Inpatient Discharges 1987
Ischemic Stroke	Institute of Public Health (Macedonia). Macedonia Hospital Inpatient Discharges 1989
Ischemic Stroke	Institute of Public Health (Macedonia). Macedonia Hospital Inpatient Discharges 1990
Ischemic Stroke	Institute of Public Health (Macedonia). Macedonia Hospital Inpatient Discharges 1991
Ischemic Stroke	Institute of Public Health (Macedonia). Macedonia Hospital Inpatient Discharges 1992
Ischemic Stroke	Institute of Public Health (Macedonia). Macedonia Hospital Inpatient Discharges 1993
Ischemic Stroke	Institute of Public Health (Macedonia). Macedonia Hospital Inpatient Discharges 1995
Ischemic Stroke	Institute of Public Health (Macedonia). Macedonia Hospital Inpatient Discharges 1996
Ischemic Stroke	Institute of Public Health (Macedonia). Macedonia Hospital Inpatient Discharges 1997
Ischemic Stroke	Institute of Public Health (Macedonia). Macedonia Hospital Inpatient Discharges 2012
Hemorrhagic Stroke	World Health Organization Regional Office for Europe (WHO/Europe). European Hospital Morbidity Database 1999-2007. Copenhagen, Denmark: World Health Organization Regional Office for Europe (WHO/Europe)
Hemorrhagic Stroke	Center for Research and Teaching in Economics (CIDE) (Mexico), National Institute of Perinatology (Mexico), National Institute of Statistics and Geography (INEGI) (Mexico), Universidad Iberoamericana. Mexico Family Life Survey 2002
Hemorrhagic Stroke	National Institute of Public Health (Mexico). Mexico National Health Survey 1999-2000
Hemorrhagic Stroke	National Institute of Public Health (Mexico). Mexico National Survey of Health and Nutrition 2005-2006. Cuernavaca, Mexico: National Institute of Public Health (Mexico)
Hemorrhagic Stroke	World Health Organization (WHO). Congo World Health Survey 2003. Geneva, Switzerland: World Health Organization (WHO), 2005
Hemorrhagic Stroke	World Health Organization (WHO). France World Health Survey 2003. Geneva, Switzerland: World Health Organization (WHO), 2005
Hemorrhagic Stroke	World Health Organization (WHO). Kenya World Health Survey 2004. Geneva, Switzerland: World Health Organization (WHO), 2005
Hemorrhagic Stroke	World Health Organization (WHO). Mexico World Health Survey 2002-2003. Geneva, Switzerland: World Health Organization (WHO), 2005
Hemorrhagic Stroke	World Health Organization (WHO). Norway World Health Survey 2003. Geneva, Switzerland: World Health Organization (WHO), 2005
Hemorrhagic Stroke	World Health Organization (WHO). Vietnam World Health Survey 2002-2003. Geneva, Switzerland: World Health Organization (WHO), 2005
Hemorrhagic Stroke	General Statistics Office (Viet Nam), United Nations Development Programme (UNDP), World Bank (WB). Viet Nam Living Standards Measurement Survey 2008. Ha N?i, Viet Nam: General Statistics Office (Viet Nam)
Hemorrhagic Stroke	General Statistics Office (Viet Nam), United Nations Development Programme (UNDP), World Bank. Vietnam Living Standards Measurement Survey 2006
Hemorrhagic Stroke	National Institute of Statistics (Cambodia), Statistics Sweden. Cambodia Socio-Economic Survey 2003-2005. Phnom Penh, Cambodia: National Institute of Statistics (Cambodia)
Hemorrhagic Stroke	National Institute of Statistics (Cambodia), Statistics Sweden. Cambodia Socio-Economic Survey 2006-2007. Phnom Penh, Cambodia: National Institute of Statistics (Cambodia)
Hemorrhagic Stroke	University of Wisconsin-Madison, Inter-University Consortium for Political and Social Research (ICPSR), College of the Northern Border (COLEF), Research in Health and Demographics (INSAD), National Institute of Medical Sciences and Nutrition Salvador Zubirán. Mexico - Mexico City Survey on Health, Well-Being, and Aging in Latin
Hemorrhagic Stroke	Management Research (IIHMR), Ministry of Public Health (Afghanistan), World Health Organization Regional Office for the Eastern Mediterranean (EMRO-WHO). Afghanistan Special Demographic and Health Survey 2010. Fairfax, United States: ICF International
Hemorrhagic Stroke	California Center for Population Research (CCPR), University of California Los Angeles (UCLA), Center for Research and Teaching in Economics (CIDE) (Mexico), National Institute of Public Health (Mexico), Universidad Iberoamericana. Mexico Family Life Survey 2005-2006
Hemorrhagic Stroke	Organization for Economic Co-operation and Development (OECD). OECD Health Statistics. Paris, France: Organization for Economic Co-operation and Development (OECD)
Hemorrhagic Stroke	National Institute of Public Health (Mexico). Mexico National Survey of Health and Nutrition 2011-2012. Cuernavaca, Mexico: National Institute of Public Health (Mexico)
Hemorrhagic Stroke	Ellekjær H, Holmen J, Indredavik B, Terent A. Epidemiology of Stroke in Innherred, Norway, 1994 to 1996: Incidence and 30-Day Case-Fatality Rate. Stroke. 1997; 28(11): 2180-4
Hemorrhagic Stroke	Bejot Y, Rouaud O, Durier J, Caillier M, Marie C, Freysz M, Yeguiayan J-M, Chantegret A, Osseby G, Moreau T, Giroud M. Decrease in the Stroke Case Fatality Rates in a French Population-Based Twenty-Year Study. Cerebrovasc Dis. 2007; 24(5): 439-44
Hemorrhagic Stroke	Giroud M, Lemesle M, Gouyon JB, Nivelon JL, Milan C, Dumas R. Cerebrovascular disease in children under 16 years of age in the city of Dijon, France: a study of incidence and clinical features from 1985 to 1993. J Clin Epidemiol. 1995; 48(11): 1343-8
Hemorrhagic Stroke	Smadja D, Cabre P, May F, Fanon J-L, René-Corail P, Riocreux C, Charpentier J-C, Fournerie P, Saint-Vil M, Ketterlé J. ERMANCIA: Epidemiology of Stroke in Martinique, French West Indies. Stroke. 2001; 32(12): 2741-7
Hemorrhagic Stroke	Wolfe CDA, Giroud M, Kolominsky-Rabas P, Dundas R, Lemesle M, Heuschmann P, Rudd A. Variations in Stroke Incidence and Survival in 3 Areas of Europe. Stroke. 2000; 31(9): 2074-9
Hemorrhagic Stroke	National Institute of Statistics, Geography, and Informatics (Mexico), Population Studies Center, University of Pennsylvania, University of Maryland, University of Wisconsin. Mexico Health and Aging Study 2001
Hemorrhagic Stroke	National Institute of Statistics, Geography, and Informatics (Mexico), Population Studies Center, University of Pennsylvania, University of Maryland, University of Wisconsin. Mexico Health and Aging Study 2003
Hemorrhagic Stroke	National Institute of Statistics, Geography, and Informatics (Mexico), Population Studies Center, University of Pennsylvania, University of Maryland, University of Wisconsin. Mexico Health and Aging Study 2012. Mexico City, México: National Institute of Statistics, Geography, and Informatics (Mexico)
Hemorrhagic Stroke	National Institute of Public Health (Mexico), World Health Organization (WHO). Mexico WHO Study on Global AGEing and Adult Health 2009-2010. Geneva, Switzerland: World Health Organization (WHO), 2011
Hemorrhagic Stroke	Béjot Y, Benzenine E, Lorgis L, Zeller M, Aubé H, Giroud M, Cottin Y, Quantin C. Comparative analysis of patients with acute coronary and cerebrovascular syndromes from the national French hospitalization health care system database. Neuroepidemiology. 2011; 37(3-4): 143-52
Hemorrhagic Stroke	Cossi M-J, Gobron C, Preux P-M, Niama D, Chabriat H, Houinato D. Stroke: prevalence and disability in Cotonou, Benin. Cerebrovasc Dis. 2012; 33(2): 166-72
Hemorrhagic Stroke	Mohammad QD, Habib M, Hoque A, Alam B, Haque B, Hossain S, Rahman KM, Khan SU. Prevalence of stroke above forty years. Mymensingh Med J. 2011; 20(4): 640-4
Hemorrhagic Stroke	Béjot Y, Cordonnier C, Durier J, Aboa-Eboulé C, Rouaud O, Giroud M. Intracerebral haemorrhage profiles are changing: results from the Dijon population-based study. Brain. 2013; 136(Pt 2): 658-64
Hemorrhagic Stroke	Tveiten A, Ljøstad U, Mygland A, Thomassen L, Pripp AH, Naess H. Intracerebral hemorrhage in southern Norway – a hospital-based incidence study. Eur Neurol. 2012; 67(4): 240-5

Hemorrhagic Stroke	Börsch-Supan, A. (2013). Survey of Health, Ageing and Retirement in Europe (SHARE) Wave 1. Release version: 2.6.0. SHARE-ERIC. Data set. DOI: 10.6103/SHARE.w1.260
Hemorrhagic Stroke	Börsch-Supan, A. (2013). Survey of Health, Ageing and Retirement in Europe (SHARE) Wave 2. Release version: 2.6.0. SHARE-ERIC. Data set. DOI: 10.6103/SHARE.w2.260
Hemorrhagic Stroke	Börsch-Supan, A. (2013). Survey of Health, Ageing and Retirement in Europe (SHARE) Wave 4. Release version: 1.1.1. SHARE-ERIC. Data set. DOI: 10.6103/SHARE.w4.111
Hemorrhagic Stroke	(KEMRI), Kenya National Bureau of Statistics, Ministry of Public Health and Sanitation (Kenya), National AIDS Control Council (Kenya), National AIDS and STI Control Program (Kenya), National Coordinating Agency for Population and Development (Kenya), National Public Health Laboratory Services, Ministry of Public Health and
Hemorrhagic Stroke	Norwegian Directorate of Health. Norway Patient Register 2009
Hemorrhagic Stroke	Norwegian Directorate of Health. Norway Patient Register 2010
Hemorrhagic Stroke	Norwegian Directorate of Health. Norway Patient Register 2011
Hemorrhagic Stroke	Norwegian Directorate of Health. Norway Patient Register 2012
Hemorrhagic Stroke	National Institute of Public Health (Mexico), World Health Organization (WHO). Mexico WHO Multi-country Survey Study on Health and Health System Responsiveness 2000-2001. Geneva, Switzerland: World Health Organization (WHO)
Hemorrhagic Stroke	Abt Associates Inc., Kenya National Bureau of Statistics, Ministry of Health (Kenya). Kenya Household Health Expenditure and Utilization Survey 2007. Nairobi, Kenya: Kenya National Bureau of Statistics
Hemorrhagic Stroke	Center for Research and Teaching in Economics (CIDE) (Mexico), Duke University, National Institute of Public Health (Mexico), Universidad Iberoamericana, University of California, Los Angeles (UCLA). Mexico Family Life Survey 2008-2013
Hemorrhagic Stroke	Action Africa Help International (AAH-I), Institute for Health Metrics and Evaluation (IHME), Ministry of Medical Services (Kenya), Ministry of Public Health and Sanitation (Kenya). Access, Bottlenecks, Costs, and Equity (ABCE) project in Kenya, 2012. Seattle, United States: Institute for Health Metrics and Evaluation (IHME), 2015
Hemorrhagic Stroke	Infectious Diseases Research Collaboration (IDRC), Institute for Health Metrics and Evaluation (IHME), Makerere University, Ministry of Health (Uganda). Access, Bottlenecks, Costs, and Equity (ABCE) project in Uganda, 2012. Seattle, United States: Institute for Health Metrics and Evaluation (IHME), 2015
Hemorrhagic Stroke	National Institute of Statistics (Cambodia), Statistics Sweden. Cambodia Socio-Economic Survey 2007-2008. Phnom Penh, Cambodia: National Institute of Statistics (Cambodia)
Hemorrhagic Stroke	Gallup Europe, World Health Organization (WHO). Bahrain WHO Multi-country Survey Study on Health and Health System Responsiveness 2000-2001. Geneva, Switzerland: World Health Organization (WHO)
Hemorrhagic Stroke	Erik Consulting, International Research Associates (INRA) Europe, World Health Organization (WHO). France WHO Multi-country Survey Study on Health and Health System Responsiveness 2000-2001. Geneva, Switzerland: World Health Organization (WHO)
Hemorrhagic Stroke	Biomedical Engineering Institute, Kaunas University of Technology, Statistics Lithuania, World Health Organization (WHO). Lithuania WHO Multi-country Survey Study on Health and Health System Responsiveness 2000-2001. Geneva, Switzerland: World Health Organization (WHO)
Hemorrhagic Stroke	Gallup Europe, World Health Organization (WHO). Oman WHO Multi-country Survey Study on Health and Health System Responsiveness 2000-2001. Geneva, Switzerland: World Health Organization (WHO)
Hemorrhagic Stroke	Börsch-Supan, A. (2015). Survey of Health, Ageing and Retirement in Europe (SHARE) Wave 5. Release version: 1.0.0. SHARE-ERIC. Data set. DOI: 10.6103/SHARE.w5.100
Hemorrhagic Stroke	Norwegian Directorate of Health. Norway Patient Register 2008-2012
Hemorrhagic Stroke	Arauz A, Villarreal-Careaga J, Rangel-Guerra R, Ramos-Moreno A, Barinagarrementeria F, PREMIER Investigators. Acute care and one-year outcome of Mexican patients with first-ever acute ischemic stroke: the PREMIER study. Rev Neurol. 2010; 51(11): 641–9
Hemorrhagic Stroke	Norwegian Directorate of Health. Norway Patient Register 2002
Hemorrhagic Stroke	Norwegian Directorate of Health. Norway Patient Register 2003
Hemorrhagic Stroke	Ministry of Health (Vietnam). Vietnam Hospital Data 2013
Hemorrhagic Stroke	World Health Organization Regional Office for Europe (WHO/Europe). European Health for All Database - Inpatient Care Discharges Per 100. Copenhagen, Denmark: World Health Organization Regional Office for Europe (WHO/Europe)
Hemorrhagic Stroke	Norwegian Directorate of Health. Norway Patient Register 2013
Hemorrhagic Stroke	Norwegian Directorate of Health. Norway Patient Register 2014
Hemorrhagic Stroke	Norwegian Directorate of Health. Norway Patient Register 2001
Hemorrhagic Stroke	Norwegian Directorate of Health. Norway Patient Register 2000
Hemorrhagic Stroke	Norwegian Directorate of Health. Norway Patient Register 1999
Hemorrhagic Stroke	Norwegian Directorate of Health. Norway Patient Register 1998
Hemorrhagic Stroke	Norwegian Directorate of Health. Norway Patient Register 1997
Hemorrhagic Stroke	Norwegian Directorate of Health. Norway Patient Register 1996
Hemorrhagic Stroke	Norwegian Directorate of Health. Norway Patient Register 1995
Hemorrhagic Stroke	Norwegian Directorate of Health. Norway Patient Register 1994
Hemorrhagic Stroke	Norwegian Directorate of Health. Norway Patient Register 1993
Hemorrhagic Stroke	Norwegian Directorate of Health. Norway Patient Register 1992
Hemorrhagic Stroke	Norwegian Directorate of Health. Norway Patient Register 1991
Hemorrhagic Stroke	Norwegian Directorate of Health. Norway Patient Register 1990
Hemorrhagic Stroke	Norwegian Directorate of Health. Norway Patient Register 1989
Hemorrhagic Stroke	Norwegian Directorate of Health. Norway Patient Register 1988
Hemorrhagic Stroke	Norwegian Directorate of Health. Norway Patient Register 1987
Hemorrhagic Stroke	Norwegian Directorate of Health. Norway Patient Register 1986
Hemorrhagic Stroke	Norwegian Directorate of Health. Norway Patient Register 1985
Hemorrhagic Stroke	Norwegian Directorate of Health. Norway Patient Register 1984
Hemorrhagic Stroke	Norwegian Directorate of Health. Norway Patient Register 1983
Hemorrhagic Stroke	Norwegian Directorate of Health. Norway Patient Register 1982
Hemorrhagic Stroke	Norwegian Directorate of Health. Norway Patient Register 1981
Hemorrhagic Stroke	Norwegian Directorate of Health. Norway Patient Register 1980
Hemorrhagic Stroke	Norwegian Directorate of Health. Norway Patient Register 1979
Hemorrhagic Stroke	Norwegian Directorate of Health. Norway Patient Register 1978
Hemorrhagic Stroke	Norwegian Directorate of Health. Norway Patient Register 1977
Hemorrhagic Stroke	Norwegian Directorate of Health. Norway Patient Register 1975
Hemorrhagic Stroke	Norwegian Directorate of Health. Norway Patient Register 1973
Hemorrhagic Stroke	Statistics Canada. Canada Community Health Survey 2000-2001. Ottawa, Canada: Statistics Canada, 2003

Hemorrhagic Stroke	Statistics Indonesia. Indonesia National Socioeconomic Survey 2005
Hemorrhagic Stroke	RAND Corporation, University of Indonesia. Indonesia Family Life Survey 1993-1994. Santa Monica, United States: RAND Corporation
Hemorrhagic Stroke	Center for Population and Policy Studies, Gadjah Mada University (Indonesia), RAND Corporation, SurveyMETER. Indonesia Family Life Survey 2007-2008. Santa Monica, United States: RAND Corporation
Hemorrhagic Stroke	Central Bureau of Statistics (Indonesia). Indonesia National Socioeconomic Survey 1992
Hemorrhagic Stroke	Central Bureau of Statistics (Indonesia). Indonesia National Socioeconomic Survey 1993
Hemorrhagic Stroke	Central Bureau of Statistics (Indonesia). Indonesia National Socioeconomic Survey 1994
Hemorrhagic Stroke	Central Bureau of Statistics (Indonesia), Ministry of Health (Indonesia), United Nations Children's Fund (UNICEF). Indonesia National Socioeconomic Survey 1995
Hemorrhagic Stroke	Central Bureau of Statistics (Indonesia), Ministry of Health (Indonesia), United Nations Children's Fund (UNICEF). Indonesia National Socioeconomic Survey 1996
Hemorrhagic Stroke	Central Bureau of Statistics (Indonesia), Ministry of Health (Indonesia), United Nations Children's Fund (UNICEF). Indonesia National Socioeconomic Survey 1997
Hemorrhagic Stroke	Central Bureau of Statistics (Indonesia), Ministry of Health (Indonesia), World Bank. Indonesia National Socioeconomic Survey 2000
Hemorrhagic Stroke	Central Bureau of Statistics (Indonesia), Ministry of Health (Indonesia), World Bank. Indonesia National Socioeconomic Survey 2001
Hemorrhagic Stroke	Statistics Indonesia. Indonesia National Socioeconomic Survey 2004
Hemorrhagic Stroke	National Institute of Statistics and Censuses (Nicaragua), World Bank. Nicaragua Living Standards Measurement Survey 1993
Hemorrhagic Stroke	National Institute of Statistics and Censuses (Nicaragua), World Bank. Nicaragua Living Standards Measurement Survey 1998-1999
Hemorrhagic Stroke	Central Statistical Office (Zambia). Zambia Living Conditions Monitoring Survey 2002-2003. Lusaka, Zambia: Central Statistical Office (Zambia)
Hemorrhagic Stroke	Central Statistical Office (Zambia). Zambia Living Conditions Monitoring Survey 2004-2005. Lusaka, Zambia: Central Statistical Office (Zambia)
Hemorrhagic Stroke	Australian Bureau of Statistics. Australia National Health Survey 1995. Canberra, Australia: Australian Bureau of Statistics
Hemorrhagic Stroke	Analytical and Information Center of the Ministry of Health of Uzbekistan, Macro International, Inc, Ministry of Macroeconomics and Statistics (Uzbekistan). Uzbekistan Special Demographic and Health Survey 2002. Calverton, United States: Macro International, Inc
Hemorrhagic Stroke	World Health Organization (WHO). Bangladesh World Health Survey 2003. Geneva, Switzerland: World Health Organization (WHO), 2005
Hemorrhagic Stroke	World Health Organization (WHO). Burkina Faso World Health Survey 2002-2003. Geneva, Switzerland: World Health Organization (WHO), 2005
Hemorrhagic Stroke	World Health Organization (WHO). Dominican Republic World Health Survey 2003. Geneva, Switzerland: World Health Organization (WHO), 2005
Hemorrhagic Stroke	World Health Organization (WHO). Germany World Health Survey 2004. Geneva, Switzerland: World Health Organization (WHO), 2005
Hemorrhagic Stroke	World Health Organization (WHO). Mali World Health Survey 2003. Geneva, Switzerland: World Health Organization (WHO), 2005
Hemorrhagic Stroke	World Health Organization (WHO). Portugal World Health Survey 2003. Geneva, Switzerland: World Health Organization (WHO), 2006
Hemorrhagic Stroke	World Health Organization (WHO). Zambia World Health Survey 2003. Geneva, Switzerland: World Health Organization (WHO), 2005
Hemorrhagic Stroke	Federal Environment Agency (Germany), Federal Institute for Drugs and Medical Devices (Germany), Max Planck Institute of Psychiatry, Robert Koch Institute. Germany National Health Interview and Examination Survey 1997-1999. Berlin, Germany: Robert Koch Institute, 2000
Hemorrhagic Stroke	Centre for Health Promotion Studies, National University of Ireland, Galway, Health Promotion Unit, Department of Health and Children (Ireland). Ireland Survey of Lifestyle Attitudes and Nutrition 1998. Dublin, Ireland: Health Promotion Unit, Department of Health and Children (Ireland)
Hemorrhagic Stroke	Statistics Indonesia. Indonesia National Socioeconomic Survey 2010
Hemorrhagic Stroke	Family Health International, Ministry of Health (Indonesia), National AIDS Commission (KPA), Statistics Indonesia. Indonesia Behavioral Surveillance Survey 2007
Hemorrhagic Stroke	Statistics Indonesia. Indonesia National Socioeconomic Survey 2002
Hemorrhagic Stroke	Statistics Indonesia. Indonesia National Socioeconomic Survey 2008
Hemorrhagic Stroke	Statistics Indonesia. Indonesia National Socioeconomic Survey - Poverty Program Evaluation 2006. Jakarta, Indonesia: Statistics Indonesia
Hemorrhagic Stroke	Statistics Indonesia. Indonesia National Socioeconomic Survey - Poverty Program Evaluation 2008-2009. Jakarta, Indonesia: Statistics Indonesia
Hemorrhagic Stroke	Federal Statistical Office (Germany). Germany Hospital Discharges by Diagnosis 2009. Wiesbaden, Germany: Federal Statistical Office (Germany), 2011
Hemorrhagic Stroke	Hamad Medical Corporation (Qatar). Qatar - Annual Inpatients Discharge Abstract: Hamad General Hospital 2002. Doha, Qatar: Hamad Medical Corporation (Qatar)
Hemorrhagic Stroke	Hamad Medical Corporation (Qatar). Qatar - Annual Inpatients Discharge Abstract: Hamad General Hospital and Women's Hospital 2003. Doha, Qatar: Hamad Medical Corporation (Qatar)
Hemorrhagic Stroke	Statistics Indonesia. Indonesia National Socioeconomic Survey 2011
Hemorrhagic Stroke	Health Care International, World Health Organization (WHO). Egypt WHO Multi-country Survey Study on Health and Health System Responsiveness 2000-2001. Geneva, Switzerland: World Health Organization (WHO)
Hemorrhagic Stroke	Institute of Health Systems (India), World Health Organization (WHO). India - Andhra Pradesh WHO Multi-country Survey Study on Health and Health System Responsiveness 2000-2001
Hemorrhagic Stroke	Thrift AG, Dewey HM, Sturm JW, Srikanth VK, Gilligan AK, Gall SL, Macdonell RAL, McNeil JJ, Donnan GA. Incidence of stroke subtypes in the North East Melbourne Stroke Incidence Study (NEMESIS): differences between men and women. Neuroepidemiology. 2009; 32(1): 11-8
Hemorrhagic Stroke	Islam MS, Anderson CS, Hankey GJ, Hardie K, Carter K, Broadhurst R, Jamrozik K. Trends in Incidence and Outcome of Stroke in Perth, Western Australia During 1989 to 2001. Stroke. 2008; 39(3): 776-82
Hemorrhagic Stroke	Kolominsky-Rabas PL, Sarti C, Heuschmann PU, Graf C, Siemonsen S, Neundoerfer B, Katalinic A, Lang E, Gassmann K-G, von Stockert TR. A Prospective Community-Based Study of Stroke in Germany-The Erlangen Stroke Project (ESPro): Incidence and Case Fatality at 1, 3, and 12 Months. Stroke. 1998; 29(12): 2501-6
Hemorrhagic Stroke	Thrift AG, Dewey HM, Macdonell RAL, McNeil JJ, Donnan GA. Incidence of the Major Stroke Subtypes: Initial Findings From the North East Melbourne Stroke Incidence Study (NEMESIS). Stroke. 2001; 32(8): 1732-8
Hemorrhagic Stroke	Correia M, Silva MR, Matos I, Magalhães R, Lopes JC, Ferro JM, Silva MC. Prospective Community-Based Study of Stroke in Northern Portugal: Incidence and Case Fatality in Rural and Urban Populations. Stroke. 2004; 35(9): 2048-53
Hemorrhagic Stroke	Hamad A, Hamad A, Sokrab TEO, Momeni S, Mesraoua B, Lingren A. Stroke in Qatar: A one-year, hospital-based study. J Stroke Cerebrovasc Dis. 2001; 10(5): 236-41
Hemorrhagic Stroke	Thrift AG, Dewey HM, Macdonell RAL, McNeil JJ, Donnan GA. Stroke Incidence on the East Coast of Australia: The North East Melbourne Stroke Incidence Study (NEMESIS). Stroke. 2000; 31(9): 2087-92
Hemorrhagic Stroke	Palm F, Urbanek C, Rose S, Buggle F, Bode B, Hennerici MG, Schmieder K, Inselmann G, Reiter R, Fleischer R, Piplack K-O, Safer A, Becher H, Grau AJ. Stroke Incidence and Survival in Ludwigshafen am Rhein, Germany: the Ludwigshafen Stroke Study (LuSSt). Stroke. 2010; 41(9): 1865-70
Hemorrhagic Stroke	Jungehülsing GJ, Müller-Nordhorn J, Nolte CH, Roll S, Rossnagel K, Reich A, Wagner A, Einhäupl KM, Willich SN, Villringer A. Prevalence of stroke and stroke symptoms: a population-based survey of 28,090 participants. Neuroepidemiology. 2008; 30(1): 51-7
Hemorrhagic Stroke	Nicoletti A, Sofia V, Giuffrida S, Bartoloni A, Bartalesi F, Bartolo MLL, Fermo SL, Cocuzza V, Gamboa H, Salazar E, Reggio A. Prevalence of Stroke: A Door-to-Door Survey in Rural Bolivia. Stroke. 2000; 31(4): 882-5
Hemorrhagic Stroke	Ferri CP, Schoenborn C, Kalra L, Acosta D, Guerra M, Huang Y, Jacob KS, Rodriguez JJL, Salas A, Sosa AL, Williams JD, Liu Z, Moriyama T, Valhuerdi A, Prince MJ. Prevalence of stroke and related burden among older people living in Latin America, India and China. J Neurol Neurosurg Psychiatr. 2011; 82(10): 1074-82

Hemorrhagic Stroke	Sienkiewicz-Jarosz H, Gluszkiewicz M, Pniewski J, Niewada M, Czlonkowska A, Wolfe C, Ryglewicz D. Incidence and case fatality rates of first-ever stroke - comparison of data from two prospective population-based studies conducted in Warsaw. Neurol Neurochir Pol. 2011; 45(3): 207-12
Hemorrhagic Stroke	Wawrzynczyk M, Pierzchala K, Braczkowska B, Manka-Gaca I, Kumor K, Borowski D, Grodzicka-Zawisza L, Zejda J. Estimates of stroke incidence and case fatality in Zabrze, 2005-2006. Neurol Neurochir Pol. 2011; 45(1): 3-10
Hemorrhagic Stroke	Leyden JM, Kleinig TJ, Newbury J, Castle S, Cranefield J, Anderson CS, Crotty M, Whitford D, Jannes J, Lee A, Greenhill J. Adelaide stroke incidence study: declining stroke rates but many preventable cardioembolic strokes. Stroke. 2013; 44(5): 1226-31
Hemorrhagic Stroke	Ministry of Health (Nicaragua), National Institute for Development Information (Nicaragua). Nicaragua National Demographic and Health Survey 2011-2012. Managua, Nicaragua: National Institute for Development Information (Nicaragua)
Hemorrhagic Stroke	Robert Koch Institute. Germany Health Update 2009-2010. Berlin, Germany: Robert Koch Institute
Hemorrhagic Stroke	Jucha R. Stroke incidence and casefatality rates in population of Krosno County. Przegl Lek. 2013; 70(4): 191-4
Hemorrhagic Stroke	Statistics Canada. Canada Community Health Survey 2005. Ottawa, Canada: Statistics Canada
Hemorrhagic Stroke	Statistics Canada. Canada Community Health Survey 2007-2008. Ottawa, Canada: Statistics Canada, 2009
Hemorrhagic Stroke	Katzenellenbogen JM, Vos T, Somerford P, Begg S, Semmens JB, Codde JP. Excess Mortality Rates for Estimating the Non-Fatal Burden of Stroke in Western Australia: A Data Linkage Study. Cerebrovasc Dis. 2010; 30(1): 57-64
Hemorrhagic Stroke	Clinton Health Access Initiative (CHAI), Institute for Health Metrics and Evaluation (IHME), Ministry of Health (Zambia), University of Zambia. Access, Bottlenecks, Costs, and Equity (ABCE) project in Zambia, 2011-2012. Seattle, United States: Institute for Health Metrics and Evaluation (IHME), 2015
Hemorrhagic Stroke	TQA Research, World Health Organization (WHO). Australia WHO Multi-country Survey Study on Health and Health System Responsiveness 2000-2001. Geneva, Switzerland: World Health Organization (WHO)
Hemorrhagic Stroke	Environics Research Group, World Health Organization (WHO). Canada WHO Multi-country Survey Study on Health and Health System Responsiveness 2000-2001. Geneva, Switzerland: World Health Organization (WHO)
Hemorrhagic Stroke	International Research Associates (INRA) Europe, World Health Organization (WHO). Germany WHO Multi-country Survey Study on Health and Health System Responsiveness 2000-2001. Geneva, Switzerland: World Health Organization (WHO)
Hemorrhagic Stroke	Public Opinion Research Center (CBOS) (Poland), World Health Organization (WHO). Poland WHO Multi-country Survey Study on Health and Health System Responsiveness 2000-2001. Geneva, Switzerland: World Health Organization (WHO)
Hemorrhagic Stroke	International Research Associates (INRA) Europe, World Health Organization (WHO). Portugal WHO Multi-country Survey Study on Health and Health System Responsiveness 2000-2001. Geneva, Switzerland: World Health Organization (WHO)
Hemorrhagic Stroke	Börsch-Supan, A. (2015). Survey of Health, Ageing and Retirement in Europe (SHARE) Wave 5. Release version: 1.0.0. SHARE-ERIC. Data set. DOI: 10.6103/SHARE.w5.100
Hemorrhagic Stroke	National Team for the Acceleration of Poverty Reduction (TNP2K) (Indonesia), SurveyMETER, University of Southern California, World Bank. Indonesia Family Life Survey East 2012
Hemorrhagic Stroke	Concluzia-Prim Center for Survey Methodology (Moldova), Independent Sociology and Information Service (OPINIA) (Moldova), Institute for Advanced Studies (Austria), London School of Hygiene and Tropical Medicine, University of Aberdeen. Moldova Health in Times of Transition Household Survey 2010
Hemorrhagic Stroke	Ministry of Rural Development (Mali), National Institute of Statistics (INSTAT) (Mali), World Bank. Mali Agricultural Integrated Economic Survey 2014-2015. Washington DC, United States: World Bank
Hemorrhagic Stroke	RAND Corporation, SurveyMETER. Indonesia Family Life Survey 2014-2015. Santa Monica, United States: RAND Corporation, 2016
Hemorrhagic Stroke	Leonards CO, Ipsen N, Malzahn U, Fiebach JB, Endres M, Ebinger M. White matter lesion severity in mild acute ischemic stroke patients and functional outcome after 1 year. Stroke. 2012; 43(11): 3046–51
Hemorrhagic Stroke	Ministry of Health (Portugal). Portugal Hospital Inpatient Discharges 2015
Hemorrhagic Stroke	Ministry of Health (Poland), National Institute of Public Health-National Institute of Hygiene (NIPH-NIH) (Poland). Poland Hospital Inpatient Discharges 2003
Hemorrhagic Stroke	Ministry of Health (Poland), National Institute of Public Health-National Institute of Hygiene (NIPH-NIH) (Poland). Poland Hospital Inpatient Discharges 2004
Hemorrhagic Stroke	Federal Statistical Office (Germany). Germany Federal Health Reporting Hospital Discharges 2000
Hemorrhagic Stroke	Federal Statistical Office (Germany). Germany Federal Health Reporting Hospital Discharges 2001
Hemorrhagic Stroke	Federal Statistical Office (Germany). Germany Federal Health Reporting Hospital Discharges 2002
Hemorrhagic Stroke	Federal Statistical Office (Germany). Germany Federal Health Reporting Hospital Discharges 1990
Hemorrhagic Stroke	Federal Statistical Office (Germany). Germany Federal Health Reporting Hospital Discharges 1991
Hemorrhagic Stroke	Federal Statistical Office (Germany). Germany Federal Health Reporting Hospital Discharges 1992
Hemorrhagic Stroke	Federal Statistical Office (Germany). Germany Federal Health Reporting Hospital Discharges 1993
Hemorrhagic Stroke	Federal Statistical Office (Germany). Germany Federal Health Reporting Hospital Discharges 1994
Hemorrhagic Stroke	Federal Statistical Office (Germany). Germany Federal Health Reporting Hospital Discharges 1995
Hemorrhagic Stroke	Federal Statistical Office (Germany). Germany Federal Health Reporting Hospital Discharges 1996
Hemorrhagic Stroke	Federal Statistical Office (Germany). Germany Federal Health Reporting Hospital Discharges 1997
Hemorrhagic Stroke	Federal Statistical Office (Germany). Germany Federal Health Reporting Hospital Discharges 1998
Hemorrhagic Stroke	Federal Statistical Office (Germany). Germany Federal Health Reporting Hospital Discharges 1999
Hemorrhagic Stroke	Federal Statistical Office (Germany). Germany Federal Health Reporting Hospital Discharges 2013
Hemorrhagic Stroke	Federal Statistical Office (Germany). Germany Federal Health Reporting Hospital Discharges 2014
Hemorrhagic Stroke	Statistics Portugal. Portugal Hospital Inpatient Discharges 1985
Hemorrhagic Stroke	Statistics Portugal. Portugal Hospital Inpatient Discharges 1986
Hemorrhagic Stroke	Statistics Portugal. Portugal Hospital Inpatient Discharges 1987
Hemorrhagic Stroke	Statistics Portugal. Portugal Hospital Inpatient Discharges 1988
Hemorrhagic Stroke	Statistics Portugal. Portugal Hospital Inpatient Discharges 1989
Hemorrhagic Stroke	Statistics Portugal. Portugal Hospital Inpatient Discharges 1990
Hemorrhagic Stroke	Statistics Portugal. Portugal Hospital Inpatient Discharges 1991
Hemorrhagic Stroke	Statistics Portugal. Portugal Hospital Inpatient Discharges 1992
Hemorrhagic Stroke	Statistics Portugal. Portugal Hospital Inpatient Discharges 1993
Hemorrhagic Stroke	Statistics Portugal. Portugal Hospital Inpatient Discharges 1994
Hemorrhagic Stroke	Statistics Portugal. Portugal Hospital Inpatient Discharges 1995
Hemorrhagic Stroke	Statistics Portugal. Portugal Hospital Inpatient Discharges 1996
Hemorrhagic Stroke	Statistics Portugal. Portugal Hospital Inpatient Discharges 1997
Hemorrhagic Stroke	Statistics Portugal. Portugal Hospital Inpatient Discharges 1998
Hemorrhagic Stroke	Statistics Portugal. Portugal Hospital Inpatient Discharges 1999
Hemorrhagic Stroke	Statistics Portugal. Portugal Hospital Inpatient Discharges 2000
Hemorrhagic Stroke	Statistics Portugal. Portugal Hospital Inpatient Discharges 2001

Hemorrhagic Stroke	Statistics Portugal. Portugal Hospital Inpatient Discharges 2002
Hemorrhagic Stroke	Statistics Portugal. Portugal Hospital Inpatient Discharges 2003
Hemorrhagic Stroke	Statistics Portugal. Portugal Hospital Inpatient Discharges 2004
Hemorrhagic Stroke	Statistics Portugal. Portugal Hospital Inpatient Discharges 2005
Hemorrhagic Stroke	Statistics Portugal. Portugal Hospital Inpatient Discharges 2006
Hemorrhagic Stroke	Statistics Portugal. Portugal Hospital Inpatient Discharges 2007
Hemorrhagic Stroke	Statistics Portugal. Portugal Hospital Inpatient Discharges 2008
Hemorrhagic Stroke	Statistics Portugal. Portugal Hospital Inpatient Discharges 2009
Hemorrhagic Stroke	Statistics Portugal. Portugal Hospital Inpatient Discharges 2010
Hemorrhagic Stroke	Statistics Portugal. Portugal Hospital Inpatient Discharges 2011
Hemorrhagic Stroke	Statistics Portugal. Portugal Hospital Inpatient Discharges 2012
Hemorrhagic Stroke	Statistics Portugal. Portugal Hospital Inpatient Discharges 2013
Hemorrhagic Stroke	Statistics Portugal. Portugal Hospital Inpatient Discharges 2014
Hemorrhagic Stroke	Ministry of Health (Poland). Poland Hospital Inpatient Discharges 2002
Hemorrhagic Stroke	Ministry of Health (Poland). Poland Hospital Inpatient Discharges 2001
Hemorrhagic Stroke	Ministry of Health (Poland). Poland Hospital Inpatient Discharges 2000
Hemorrhagic Stroke	Ministry of Health (Poland). Poland Hospital Inpatient Discharges 1999
Hemorrhagic Stroke	Ministry of Health (Poland). Poland Hospital Inpatient Discharges 1998
Hemorrhagic Stroke	Ministry of Health (Poland). Poland Hospital Inpatient Discharges 1997
Hemorrhagic Stroke	Ministry of Health (Poland). Poland Hospital Inpatient Discharges 1996
Hemorrhagic Stroke	Ministry of Health (Poland). Poland Hospital Inpatient Discharges 1995
Hemorrhagic Stroke	Ministry of Health (Poland). Poland Hospital Inpatient Discharges 1994
Hemorrhagic Stroke	Ministry of Health (Poland). Poland Hospital Inpatient Discharges 1993
Hemorrhagic Stroke	Ministry of Health (Poland). Poland Hospital Inpatient Discharges 1992
Hemorrhagic Stroke	Ministry of Health (Poland). Poland Hospital Inpatient Discharges 1991
Hemorrhagic Stroke	Ministry of Health (Poland). Poland Hospital Inpatient Discharges 1990
Hemorrhagic Stroke	Ministry of Health (Poland). Poland Hospital Inpatient Discharges 1989
Hemorrhagic Stroke	Ministry of Health (Poland). Poland Hospital Inpatient Discharges 1988
Hemorrhagic Stroke	Ministry of Health (Poland). Poland Hospital Inpatient Discharges 1987
Hemorrhagic Stroke	Ministry of Health (Poland). Poland Hospital Inpatient Discharges 1986
Hemorrhagic Stroke	Ministry of Health (Poland). Poland Hospital Inpatient Discharges 1985
Hemorrhagic Stroke	Ministry of Health (Poland). Poland Hospital Inpatient Discharges 1984
Hemorrhagic Stroke	Ministry of Health (Poland). Poland Hospital Inpatient Discharges 1983
Hemorrhagic Stroke	Ministry of Health (Poland). Poland Hospital Inpatient Discharges 1982
Hemorrhagic Stroke	Ministry of Health (Poland). Poland Hospital Inpatient Discharges 1981
Hemorrhagic Stroke	Ministry of Health (Poland). Poland Hospital Inpatient Discharges 1980
Hemorrhagic Stroke	Ministry of Health (Poland). Poland Hospital Inpatient Discharges 2013
Hemorrhagic Stroke	Ministry of Health (Poland). Poland Hospital Inpatient Discharges 2014
Hemorrhagic Stroke	Census and Statistics Directorate (Panama), Ministry of Economy and Finance (Panama), World Bank. Panama Living Standard Measurement Survey 2003. Washington DC, United States: World Bank
Hemorrhagic Stroke	Carolina Population Center, University of North Carolina at Chapel Hill, Institute of Sociology, Russian Academy of Sciences, National Research University Higher School of Economics (Russia), ZAO Demoscope. Russia Longitudinal Monitoring Survey of HSE, Round II 1992-1993
Hemorrhagic Stroke	Carolina Population Center, University of North Carolina at Chapel Hill, Institute of Sociology, Russian Academy of Sciences, National Research University Higher School of Economics (Russia), ZAO Demoscope. Russia Longitudinal Monitoring Survey of HSE, Round IV 1993-1994
Hemorrhagic Stroke	Russia Longitudinal Monitoring Survey (RLMS-HSE), Round VII 1996. National Research University Higher School of Economics, ZAO Demoscope, Carolina Population Center, Univeristy of North Carolina at Chapel Hill, Institute of Sociology, Russian Academy of Sciences
Hemorrhagic Stroke	Russia Longitudinal Monitoring Survey (RLMS-HSE), Round VIII 1998-1999. National Research University Higher School of Economics, ZAO Demoscope, Carolina Population Center, Univeristy of North Carolina at Chapel Hill, Institute of Sociology, Russian Academy of Sciences
Hemorrhagic Stroke	Russia Longitudinal Monitoring Survey (RLMS-HSE), Round X 2001. National Research University Higher School of Economics, ZAO Demoscope, Carolina Population Center, Univeristy of North Carolina at Chapel Hill, Institute of Sociology, Russian Academy of Sciences
Hemorrhagic Stroke	Russia Longitudinal Monitoring Survey (RLMS-HSE), Round XI 2002. National Research University Higher School of Economics, ZAO Demoscope, Carolina Population Center, Univeristy of North Carolina at Chapel Hill, Institute of Sociology, Russian Academy of Sciences
Hemorrhagic Stroke	Russia Longitudinal Monitoring Survey (RLMS-HSE), Round XII 2003. National Research University Higher School of Economics, ZAO Demoscope, Carolina Population Center, Univeristy of North Carolina at Chapel Hill, Institute of Sociology, Russian Academy of Sciences
Hemorrhagic Stroke	Russia Longitudinal Monitoring Survey (RLMS-HSE), Round XIII 2004. National Research University Higher School of Economics, ZAO Demoscope, Carolina Population Center, Univeristy of North Carolina at Chapel Hill, Institute of Sociology, Russian Academy of Sciences
Hemorrhagic Stroke	Russia Longitudinal Monitoring Survey (RLMS-HSE), Round XIV 2005. National Research University Higher School of Economics, ZAO Demoscope, Carolina Population Center, Univeristy of North Carolina at Chapel Hill, Institute of Sociology, Russian Academy of Sciences
Hemorrhagic Stroke	Ministry of Health, Social Services and Equality (Spain), National Statistics Institute (Spain). Spain National Health Survey 2006-2007
Hemorrhagic Stroke	Health and Retirement Study, (Biennial 1992) public use dataset. Produced and distributed by the University of Michigan with funding from the National Institute on Aging (grant number NIA U01AG009740). Ann Arbor, MI, (2011)
Hemorrhagic Stroke	and Prevention. National Center for Health Statistics. National Health Interview Survey, 1994: Second Longitudinal Study on Aging, Wave 2, 1997. ICPSR03526-v2. Ann Arbor, MI: Inter-university Consortium for Political and Social Research [distributor], 2007-03-01. http://doi.org/10.3886/ICPSR03526.v2
Hemorrhagic Stroke	World Health Organization (WHO). Ecuador World Health Survey 2003. Geneva, Switzerland: World Health Organization (WHO), 2005
Hemorrhagic Stroke	World Health Organization (WHO). Greece World Health Survey 2003. Geneva, Switzerland: World Health Organization (WHO), 2005
Hemorrhagic Stroke	World Health Organization (WHO). Laos World Health Survey 2003
Hemorrhagic Stroke	World Health Organization (WHO). Malawi World Health Survey 2003. Geneva, Switzerland: World Health Organization (WHO), 2005
Hemorrhagic Stroke	World Health Organization (WHO). Mauritania World Health Survey 2003. Geneva, Switzerland: World Health Organization (WHO), 2005
Hemorrhagic Stroke	World Health Organization (WHO). Russia World Health Survey 2003. Geneva, Switzerland: World Health Organization (WHO), 2005
Hemorrhagic Stroke	World Health Organization (WHO). Spain World Health Survey 2002-2003. Geneva, Switzerland: World Health Organization (WHO), 2005
Hemorrhagic Stroke	National Center for Health Statistics (NCHS), Centers for Disease Control and Prevention (CDC). United States National Health and Nutrition Examination Survey 2007-2008. Hyattsville, United States: National Center for Health Statistics (NCHS), Centers for Disease Control and Prevention (CDC), 2009

Hemorrhagic Stroke	National Center for Health Statistics (NCHS), Centers for Disease Control and Prevention (CDC), US Census Bureau. United States National Health Interview Survey 1990. Hyattsville, United States: National Center for Health Statistics (NCHS), Centers for Disease Control and Prevention (CDC)
Hemorrhagic Stroke	National Center for Health Statistics (NCHS), Centers for Disease Control and Prevention (CDC), US Census Bureau. United States National Health Interview Survey 1991. Hyattsville, United States: National Center for Health Statistics (NCHS), Centers for Disease Control and Prevention (CDC)
Hemorrhagic Stroke	National Center for Health Statistics (NCHS), Centers for Disease Control and Prevention (CDC), US Census Bureau. United States National Health Interview Survey 1992. Hyattsville, United States: National Center for Health Statistics (NCHS), Centers for Disease Control and Prevention (CDC)
Hemorrhagic Stroke	National Center for Health Statistics (NCHS), Centers for Disease Control and Prevention (CDC), US Census Bureau. United States National Health Interview Survey 1993. Hyattville, United States: National Center for Health Statistics (NCHS), Centers for Disease Control and Prevention (CDC)
Hemorrhagic Stroke	National Center for Health Statistics (NCHS), Centers for Disease Control and Prevention (CDC), US Census Bureau. United States National Health Interview Survey 1997. Hyattsville, United States: National Center for Health Statistics (NCHS), Centers for Disease Control and Prevention (CDC)
Hemorrhagic Stroke	National Center for Health Statistics (NCHS), Centers for Disease Control and Prevention (CDC), US Census Bureau. United States National Health Interview Survey 1998. Hyattsville, United States: National Center for Health Statistics (NCHS), Centers for Disease Control and Prevention (CDC)
Hemorrhagic Stroke	National Center for Health Statistics (NCHS), Centers for Disease Control and Prevention (CDC), US Census Bureau. United States National Health Interview Survey 1999. Hyattsville, United States: National Center for Health Statistics (NCHS), Centers for Disease Control and Prevention (CDC)
Hemorrhagic Stroke	National Center for Health Statistics (NCHS), Centers for Disease Control and Prevention (CDC), US Census Bureau. United States National Health Interview Survey 2000. Hyattsville, United States: National Center for Health Statistics (NCHS), Centers for Disease Control and Prevention (CDC)
Hemorrhagic Stroke	National Center for Health Statistics (NCHS), Centers for Disease Control and Prevention (CDC), US Census Bureau. United States National Health Interview Survey 2001. Hyattsville, United States: National Center for Health Statistics (NCHS), Centers for Disease Control and Prevention (CDC)
Hemorrhagic Stroke	National Center for Health Statistics (NCHS), Centers for Disease Control and Prevention (CDC), US Census Bureau. United States National Health Interview Survey 2002. Hyattsville, United States: National Center for Health Statistics (NCHS), Centers for Disease Control and Prevention (CDC)
Hemorrhagic Stroke	National Center for Health Statistics (NCHS), Centers for Disease Control and Prevention (CDC), US Census Bureau. United States National Health Interview Survey 2003. Hyattsville, United States: National Center for Health Statistics (NCHS), Centers for Disease Control and Prevention (CDC)
Hemorrhagic Stroke	National Center for Health Statistics (NCHS), Centers for Disease Control and Prevention (CDC), US Census Bureau. United States National Health Interview Survey 2004. Hyattsville, United States: National Center for Health Statistics (NCHS), Centers for Disease Control and Prevention (CDC)
Hemorrhagic Stroke	National Center for Health Statistics (NCHS), Centers for Disease Control and Prevention (CDC), US Census Bureau. United States National Health Interview Survey 2005. Hyattsville, United States: National Center for Health Statistics (NCHS), Centers for Disease Control and Prevention (CDC)
Hemorrhagic Stroke	National Center for Health Statistics (NCHS), Centers for Disease Control and Prevention (CDC), US Census Bureau. United States National Health Interview Survey 2006. Hyattsville, United States: National Center for Health Statistics (NCHS), Centers for Disease Control and Prevention (CDC)
Hemorrhagic Stroke	National Center for Health Statistics (NCHS), Centers for Disease Control and Prevention (CDC), US Census Bureau. United States National Health Interview Survey 2007. Hyattsville, United States: National Center for Health Statistics (NCHS), Centers for Disease Control and Prevention (CDC)
Hemorrhagic Stroke	National Center for Health Statistics (NCHS), Centers for Disease Control and Prevention (CDC), US Census Bureau. United States National Health Interview Survey 2008. Hyattsville, United States: National Center for Health Statistics (NCHS), Centers for Disease Control and Prevention (CDC)
Hemorrhagic Stroke	National Center for Health Statistics (NCHS), Centers for Disease Control and Prevention (CDC), US Census Bureau. United States National Health Interview Survey 2009. Hyattsville, United States: National Center for Health Statistics (NCHS), Centers for Disease Control and Prevention (CDC)
Hemorrhagic Stroke	National Center for Health Statistics (NCHS), Centers for Disease Control and Prevention (CDC), US Census Bureau. United States National Health Interview Survey 2010. Hyattsville, United States: National Center for Health Statistics (NCHS), Centers for Disease Control and Prevention (CDC)
Hemorrhagic Stroke	National Statistical Office of Malawi, World Bank. Malawi Living Standards Measurement Survey 2004-2005
Hemorrhagic Stroke	National Center for Health Statistics (NCHS), Centers for Disease Control and Prevention (CDC). United States National Health and Nutrition Examination Survey 2005-2006. Hyattsville, United States: National Center for Health Statistics (NCHS), Centers for Disease Control and Prevention (CDC), 2007
Hemorrhagic Stroke	National Center for Health Statistics (NCHS), Centers for Disease Control and Prevention (CDC). United States National Health and Nutrition Examination Survey 2003-2004. Hyattsville, United States: National Center for Health Statistics (NCHS), Centers for Disease Control and Prevention (CDC)
Hemorrhagic Stroke	National Center for Health Statistics (NCHS), Centers for Disease Control and Prevention (CDC). United States National Health and Nutrition Examination Survey 2009-2010. Hyattsville, United States: National Center for Health Statistics (NCHS), Centers for Disease Control and Prevention (CDC), 2011
Hemorrhagic Stroke	National Center for Health Statistics (NCHS), Centers for Disease Control and Prevention (CDC). United States National Health and Nutrition Examination Survey 1988-1994. Hyattsville, United States: National Center for Health Statistics (NCHS), Centers for Disease Control and Prevention (CDC)
Hemorrhagic Stroke	National Center for Health Statistics (NCHS), Centers for Disease Control and Prevention (CDC). United States National Health and Nutrition Examination Survey 2001-2002. Hyattsville, United States: National Center for Health Statistics (NCHS), Centers for Disease Control and Prevention (CDC)
Hemorrhagic Stroke	National Center for Health Statistics (NCHS), Centers for Disease Control and Prevention (CDC). United States National Health and Nutrition Examination Survey 1999-2000. Hyattsville, United States: National Center for Health Statistics (NCHS), Centers for Disease Control and Prevention (CDC)
Hemorrhagic Stroke	Health and Retirement Study, (Biennial 1996) public use dataset. Produced and distributed by the University of Michigan with funding from the National Institute on Aging (grant number NIA U01AG009740). Ann Arbor, MI, (2011)
Hemorrhagic Stroke	Health and Retirement Study, (Biennial 1998) public use dataset. Produced and distributed by the University of Michigan with funding from the National Institute on Aging (grant number NIA U01AG009740). Ann Arbor, MI, (2014)
Hemorrhagic Stroke	Health and Retirement Study, (Biennial 2000) public use dataset. Produced and distributed by the University of Michigan with funding from the National Institute on Aging (grant number NIA U01AG009740). Ann Arbor, MI, (2011)
Hemorrhagic Stroke	Health and Retirement Study, (Biennial 2002) public use dataset. Produced and distributed by the University of Michigan with funding from the National Institute on Aging (grant number NIA U01AG009740). Ann Arbor, MI, (2011)
Hemorrhagic Stroke	Health and Retirement Study, (Biennial 2004) public use dataset. Produced and distributed by the University of Michigan with funding from the National Institute on Aging (grant number NIA U01AG009740). Ann Arbor, MI, (2013)
Hemorrhagic Stroke	Health and Retirement Study, (Biennial 2006) public use dataset. Produced and distributed by the University of Michigan with funding from the National Institute on Aging (grant number NIA U01AG009740). Ann Arbor, MI, (2014)
Hemorrhagic Stroke	Health and Retirement Study, (Biennial 2010) public use dataset. Produced and distributed by the University of Michigan with funding from the National Institute on Aging (grant number NIA U01AG009740). Ann Arbor, MI, (2014)
Hemorrhagic Stroke	Panel Study of Income Dynamics, 2007 public use dataset. Produced and distributed by the University of Michigan with primary funding from the National Science Foundation, the National Institute of Aging, and the National Institute of Child Health and Human Development. Ann Arbor, MI, (2011)
Hemorrhagic Stroke	Panel Study of Income Dynamics, 2005 public use dataset. Produced and distributed by the University of Michigan with primary funding from the National Science Foundation, the National Institute of Aging, and the National Institute of Child Health and Human Development. Ann Arbor, MI, (2011)

Hemorrhagic Stroke	National Center for Health Statistics (NCHS), Centers for Disease Control and Prevention (CDC), United States Census Bureau. United States National Hospital Discharge Survey 1996. Hyattsville, United States: National Center for Health Statistics (NCHS), Centers for Disease Control and Prevention (CDC)
Hemorrhagic Stroke	National Center for Health Statistics (NCHS), Centers for Disease Control and Prevention (CDC), United States Census Bureau. United States National Hospital Discharge Survey 1997. Hyattsville, United States: National Center for Health Statistics (NCHS), Centers for Disease Control and Prevention (CDC)
Hemorrhagic Stroke	National Center for Health Statistics (NCHS), Centers for Disease Control and Prevention (CDC), United States Census Bureau. United States National Hospital Discharge Survey 1998. Hyattsville, United States: National Center for Health Statistics (NCHS), Centers for Disease Control and Prevention (CDC)
Hemorrhagic Stroke	National Center for Health Statistics (NCHS), Centers for Disease Control and Prevention (CDC), United States Census Bureau. United States National Hospital Discharge Survey 1999. Hyattsville, United States: National Center for Health Statistics (NCHS), Centers for Disease Control and Prevention (CDC)
Hemorrhagic Stroke	National Center for Health Statistics (NCHS), Centers for Disease Control and Prevention (CDC), United States Census Bureau. United States National Hospital Discharge Survey 2000. Hyattsville, United States: National Center for Health Statistics (NCHS), Centers for Disease Control and Prevention (CDC)
Hemorrhagic Stroke	National Center for Health Statistics (NCHS), Centers for Disease Control and Prevention (CDC), United States Census Bureau. United States National Hospital Discharge Survey 2001. Hyattsville, United States: National Center for Health Statistics (NCHS), Centers for Disease Control and Prevention (CDC)
Hemorrhagic Stroke	National Center for Health Statistics (NCHS), Centers for Disease Control and Prevention (CDC), United States Census Bureau. United States National Hospital Discharge Survey 2002. Hyattsville, United States: National Center for Health Statistics (NCHS), Centers for Disease Control and Prevention (CDC)
Hemorrhagic Stroke	National Center for Health Statistics (NCHS), Centers for Disease Control and Prevention (CDC), United States Census Bureau. United States National Hospital Discharge Survey 2003. Hyattsville, United States: National Center for Health Statistics (NCHS), Centers for Disease Control and Prevention (CDC)
Hemorrhagic Stroke	National Center for Health Statistics (NCHS), Centers for Disease Control and Prevention (CDC), United States Census Bureau. United States National Hospital Discharge Survey 2004. Hyattsville, United States: National Center for Health Statistics (NCHS), Centers for Disease Control and Prevention (CDC)
Hemorrhagic Stroke	National Center for Health Statistics (NCHS), Centers for Disease Control and Prevention (CDC), United States Census Bureau. United States National Hospital Discharge Survey 2005. Hyattsville, United States: National Center for Health Statistics (NCHS), Centers for Disease Control and Prevention (CDC)
Hemorrhagic Stroke	National Center for Health Statistics (NCHS), Centers for Disease Control and Prevention (CDC), United States Census Bureau. United States National Hospital Discharge Survey 2006. Hyattsville, United States: National Center for Health Statistics (NCHS), Centers for Disease Control and Prevention (CDC)
Hemorrhagic Stroke	National Center for Health Statistics (NCHS), Centers for Disease Control and Prevention (CDC), United States Census Bureau. United States National Hospital Discharge Survey 2007. Hyattsville, United States: National Center for Health Statistics (NCHS), Centers for Disease Control and Prevention (CDC)
Hemorrhagic Stroke	National Center for Health Statistics (NCHS), Centers for Disease Control and Prevention (CDC), United States Census Bureau. United States National Hospital Discharge Survey 2008. Hyattsville, United States: National Center for Health Statistics (NCHS), Centers for Disease Control and Prevention (CDC)
Hemorrhagic Stroke	National Center for Health Statistics (NCHS), Centers for Disease Control and Prevention (CDC), United States Census Bureau. United States National Hospital Discharge Survey 2009. Hyattsville, United States: National Center for Health Statistics (NCHS), Centers for Disease Control and Prevention (CDC)
Hemorrhagic Stroke	National Center for Health Statistics (NCHS), Centers for Disease Control and Prevention (CDC), United States Census Bureau. United States National Hospital Discharge Survey 2010. Hyattsville, United States: National Center for Health Statistics (NCHS), Centers for Disease Control and Prevention (CDC)
Hemorrhagic Stroke	National Center for Health Statistics (NCHS), Centers for Disease Control and Prevention (CDC), United States Census Bureau. United States National Hospital Discharge Survey 1979. Hyattsville, United States: National Center for Health Statistics (NCHS), Centers for Disease Control and Prevention (CDC)
Hemorrhagic Stroke	National Center for Health Statistics (NCHS), Centers for Disease Control and Prevention (CDC), United States Census Bureau. United States National Hospital Discharge Survey 1980. Hyattsville, United States: National Center for Health Statistics (NCHS), Centers for Disease Control and Prevention (CDC)
Hemorrhagic Stroke	National Center for Health Statistics (NCHS), Centers for Disease Control and Prevention (CDC), United States Census Bureau. United States National Hospital Discharge Survey 1981. Hyattsville, United States: National Center for Health Statistics (NCHS), Centers for Disease Control and Prevention (CDC)
Hemorrhagic Stroke	National Center for Health Statistics (NCHS), Centers for Disease Control and Prevention (CDC), United States Census Bureau. United States National Hospital Discharge Survey 1982. Hyattsville, United States: National Center for Health Statistics (NCHS), Centers for Disease Control and Prevention (CDC)
Hemorrhagic Stroke	National Center for Health Statistics (NCHS), Centers for Disease Control and Prevention (CDC), United States Census Bureau. United States National Hospital Discharge Survey 1983. Hyattsville, United States: National Center for Health Statistics (NCHS), Centers for Disease Control and Prevention (CDC)
Hemorrhagic Stroke	National Center for Health Statistics (NCHS), Centers for Disease Control and Prevention (CDC), United States Census Bureau. United States National Hospital Discharge Survey 1984. Hyattsville, United States: National Center for Health Statistics (NCHS), Centers for Disease Control and Prevention (CDC)
Hemorrhagic Stroke	National Center for Health Statistics (NCHS), Centers for Disease Control and Prevention (CDC), United States Census Bureau. United States National Hospital Discharge Survey 1985. Hyattsville, United States: National Center for Health Statistics (NCHS), Centers for Disease Control and Prevention (CDC)
Hemorrhagic Stroke	National Center for Health Statistics (NCHS), Centers for Disease Control and Prevention (CDC), United States Census Bureau. United States National Hospital Discharge Survey 1986. Hyattsville, United States: National Center for Health Statistics (NCHS), Centers for Disease Control and Prevention (CDC)
Hemorrhagic Stroke	National Center for Health Statistics (NCHS), Centers for Disease Control and Prevention (CDC), United States Census Bureau. United States National Hospital Discharge Survey 1987. Hyattsville, United States: National Center for Health Statistics (NCHS), Centers for Disease Control and Prevention (CDC)
Hemorrhagic Stroke	National Center for Health Statistics (NCHS), Centers for Disease Control and Prevention (CDC), United States Census Bureau. United States National Hospital Discharge Survey 1988. Hyattsville, United States: National Center for Health Statistics (NCHS), Centers for Disease Control and Prevention (CDC)

Hemorrhagic Stroke	National Center for Health Statistics (NCHS), Centers for Disease Control and Prevention (CDC), United States Census Bureau. United States National Hospital Discharge Survey 1989. Hyattsville, United States: National Center for Health Statistics (NCHS), Centers for Disease Control and Prevention (CDC)
Hemorrhagic Stroke	National Center for Health Statistics (NCHS), Centers for Disease Control and Prevention (CDC), United States Census Bureau. United States National Hospital Discharge Survey 1990. Hyattsville, United States: National Center for Health Statistics (NCHS), Centers for Disease Control and Prevention (CDC)
Hemorrhagic Stroke	National Center for Health Statistics (NCHS), Centers for Disease Control and Prevention (CDC), United States Census Bureau. United States National Hospital Discharge Survey 1991. Hyattsville, United States: National Center for Health Statistics (NCHS), Centers for Disease Control and Prevention (CDC)
Hemorrhagic Stroke	National Center for Health Statistics (NCHS), Centers for Disease Control and Prevention (CDC), United States Census Bureau. United States National Hospital Discharge Survey 1992. Hyattsville, United States: National Center for Health Statistics (NCHS), Centers for Disease Control and Prevention (CDC)
Hemorrhagic Stroke	National Center for Health Statistics (NCHS), Centers for Disease Control and Prevention (CDC), United States Census Bureau. United States National Hospital Discharge Survey 1993. Hyattsville, United States: National Center for Health Statistics (NCHS), Centers for Disease Control and Prevention (CDC)
Hemorrhagic Stroke	National Center for Health Statistics (NCHS), Centers for Disease Control and Prevention (CDC), United States Census Bureau. United States National Hospital Discharge Survey 1994. Hyattsville, United States: National Center for Health Statistics (NCHS), Centers for Disease Control and Prevention (CDC)
Hemorrhagic Stroke	National Center for Health Statistics (NCHS), Centers for Disease Control and Prevention (CDC), United States Census Bureau. United States National Hospital Discharge Survey 1995. Hyattsville, United States: National Center for Health Statistics (NCHS), Centers for Disease Control and Prevention (CDC)
Hemorrhagic Stroke	National Institute of Statistics and Censuses (Ecuador). Ecuador Hospital Inpatient Discharges 1997. Quito, Ecuador: National Institute of Statistics and Censuses (Ecuador)
Hemorrhagic Stroke	National Institute of Statistics and Censuses (Ecuador). Ecuador Hospital Inpatient Discharges 1998. Quito, Ecuador: National Institute of Statistics and Censuses (Ecuador)
Hemorrhagic Stroke	National Institute of Statistics and Censuses (Ecuador). Ecuador Hospital Inpatient Discharges 1999. Quito, Ecuador: National Institute of Statistics and Censuses (Ecuador)
Hemorrhagic Stroke	National Institute of Statistics and Censuses (Ecuador). Ecuador Hospital Inpatient Discharges 2000. Quito, Ecuador: National Institute of Statistics and Censuses (Ecuador)
Hemorrhagic Stroke	National Institute of Statistics and Censuses (Ecuador). Ecuador Hospital Inpatient Discharges 2001. Quito, Ecuador: National Institute of Statistics and Censuses (Ecuador)
Hemorrhagic Stroke	National Institute of Statistics and Censuses (Ecuador). Ecuador Hospital Inpatient Discharges 2002. Quito, Ecuador: National Institute of Statistics and Censuses (Ecuador)
Hemorrhagic Stroke	National Institute of Statistics and Censuses (Ecuador). Ecuador Hospital Inpatient Discharges 2003. Quito, Ecuador: National Institute of Statistics and Censuses (Ecuador)
Hemorrhagic Stroke	National Institute of Statistics and Censuses (Ecuador). Ecuador Hospital Inpatient Discharges 2004. Quito, Ecuador: National Institute of Statistics and Censuses (Ecuador)
Hemorrhagic Stroke	National Institute of Statistics and Censuses (Ecuador). Ecuador Hospital Inpatient Discharges 2005. Quito, Ecuador: National Institute of Statistics and Censuses (Ecuador)
Hemorrhagic Stroke	National Institute of Statistics and Censuses (Ecuador). Ecuador Hospital Inpatient Discharges 2006. Quito, Ecuador: National Institute of Statistics and Censuses (Ecuador)
Hemorrhagic Stroke	National Institute of Statistics and Censuses (Ecuador). Ecuador Hospital Inpatient Discharges 2007. Quito, Ecuador: National Institute of Statistics and Censuses (Ecuador)
Hemorrhagic Stroke	National Institute of Statistics and Censuses (Ecuador). Ecuador Hospital Inpatient Discharges 2008. Quito, Ecuador: National Institute of Statistics and Censuses (Ecuador)
Hemorrhagic Stroke	National Institute of Statistics and Censuses (Ecuador). Ecuador Hospital Inpatient Discharges 2009. Quito, Ecuador: National Institute of Statistics and Censuses (Ecuador)
Hemorrhagic Stroke	National Institute of Statistics and Censuses (Ecuador). Ecuador Hospital Inpatient Discharges 2010. Quito, Ecuador: National Institute of Statistics and Censuses (Ecuador)
Hemorrhagic Stroke	National Institute of Statistics and Censuses (Ecuador). Ecuador Hospital Inpatient Discharges 2011. Quito, Ecuador: National Institute of Statistics and Censuses (Ecuador)
Hemorrhagic Stroke	Healthcare Cost and Utilization Project (HCUP), Agency for Healthcare Research and Quality (AHRQ). United States State Inpatient Databases 2003. Rockville, United States: Healthcare Cost and Utilization Project (HCUP), Agency for Healthcare Research and Quality (AHRQ)
Hemorrhagic Stroke	Healthcare Cost and Utilization Project (HCUP), Agency for Healthcare Research and Quality (AHRQ). United States State Inpatient Databases 2004. Rockville, United States: Healthcare Cost and Utilization Project (HCUP), Agency for Healthcare Research and Quality (AHRQ)
Hemorrhagic Stroke	Healthcare Cost and Utilization Project (HCUP), Agency for Healthcare Research and Quality (AHRQ). United States State Inpatient Databases 2005. Rockville, United States: Healthcare Cost and Utilization Project (HCUP), Agency for Healthcare Research and Quality (AHRQ)
Hemorrhagic Stroke	Healthcare Cost and Utilization Project (HCUP), Agency for Healthcare Research and Quality (AHRQ). United States State Inpatient Databases 2006. Rockville, United States: Healthcare Cost and Utilization Project (HCUP), Agency for Healthcare Research and Quality (AHRQ)
Hemorrhagic Stroke	Healthcare Cost and Utilization Project (HCUP), Agency for Healthcare Research and Quality (AHRQ). United States State Inpatient Databases 2007. Rockville, United States: Healthcare Cost and Utilization Project (HCUP), Agency for Healthcare Research and Quality (AHRQ)
Hemorrhagic Stroke	Healthcare Cost and Utilization Project (HCUP), Agency for Healthcare Research and Quality (AHRQ). United States State Inpatient Databases 2008. Rockville, United States: Healthcare Cost and Utilization Project (HCUP), Agency for Healthcare Research and Quality (AHRQ)
Hemorrhagic Stroke	Healthcare Cost and Utilization Project (HCUP), Agency for Healthcare Research and Quality (AHRQ). United States State Inpatient Databases 2009. Rockville, United States: Healthcare Cost and Utilization Project (HCUP), Agency for Healthcare Research and Quality (AHRQ)
Hemorrhagic Stroke	Health Measurement Research Group, University of Wisconsin. United States National Health Measurement Study 2005-2006. Data and Information Sciences Center, University of Wisconsin-Madison [distributor]
Hemorrhagic Stroke	National Center for Health Statistics (NCHS), Centers for Disease Control and Prevention (CDC), US Census Bureau. United States National Health Interview Survey 2011. Hyattsville, United States: National Center for Health Statistics (NCHS), Centers for Disease Control and Prevention (CDC)
Hemorrhagic Stroke	Ministry of Health and Medical Education (Iran), World Health Organization (WHO). Iran WHO Multi-country Survey Study on Health and Health System Responsiveness 2000-2001. Geneva, Switzerland: World Health Organization (WHO)
Hemorrhagic Stroke	National Statistical Office of Malawi, World Bank. Malawi Integrated Household Survey 2010-2011. Washington DC, United States: World Bank
Hemorrhagic Stroke	Agency for Healthcare Research and Quality. United States Medical Expenditure Panel Survey 2002-2009. Rockville, United States: Agency for Healthcare Research and Quality

Hemorrhagic Stroke	Feigin V, Carter K, Hackett M, Barber PA, McNaughton H, Dyall L, Chen M, Anderson C. Ethnic disparities in incidence of stroke subtypes: Auckland Regional Community Stroke Study, 2002-2003. Lancet Neurol. 2006; 5(2): 130-9
Hemorrhagic Stroke	Feigin VL, Wiebers DO, Nikitin YP, O'Fallon WM, Whisnant JP. Stroke Epidemiology in Novosibirsk, Russia: A Population-Based Study. Mayo Clin Proc. 1995; 70(9): 847-52
Hemorrhagic Stroke	Azarpazhooh MR, Etemadi MM, Donnan GA, Mokhber N, Majdi MR, Ghayour-Mobarhan M, Ghandehary K, Farzadfard MT, Kiani R, Panahandeh M, Thrift AG. Excessive Incidence of Stroke in Iran. Stroke. 2010; 41(1): e3-e10
Hemorrhagic Stroke	Vemmos KN, Bots ML, Tsibouris PK, Zis VP, Grobbee DE, Stranjalis GS, Stamatelopoulos S. Stroke Incidence and Case Fatality in Southern Greece: The Arcadia Stroke Registry. Stroke. 1999; 30(2): 363-70
Hemorrhagic Stroke	al-Rajeh S, Larbi EB, Bademosi O, Awada A, Yousef A, al-Freihi H, Miniawi H. Stroke register: experience from the eastern province of Saudi Arabia. Cerebrovasc Dis. 1998; 8(2): 86-9
Hemorrhagic Stroke	Al Rajeh S. Stroke in the Elderly Aged 75 Years and Above. Cerebrovasc Dis. 1994; 4(6): 402-6
Hemorrhagic Stroke	Anderson CS, Carter KN, Hackett ML, Feigin V, Barber PA, Broad JB, Bonita R. Trends in Stroke Incidence in Auckland, New Zealand, During 1981 to 2003. Stroke. 2005; 36(10): 2087-93
Hemorrhagic Stroke	Awada A, Russell N, Al Rajeh S, Omojola M. Non-traumatic cerebral hemorrage in Saudi Arabs: a hospital-based study of 243 cases. J Neurol Sci. 1996; 144(1-2): 198-203
Hemorrhagic Stroke	Awada A. Stroke in Saudi Arabian young adults: a study of 120 cases. Acta Neurol Scand. 1994; 89(5): 323-8
Hemorrhagic Stroke	Bonita R, Broad JB, Beaglehole R. Changes in stroke incidence and case-fatality in Auckland, New Zealand, 1981-91. Lancet. 1993; 342(8885): 1470-3
Hemorrhagic Stroke	Earley CJ, Kittner SJ, Feeser BR, Gardner J, Epstein A, Wozniak MA, Wityk R, Stern BJ, Price TR, Macko RF, Johnson C, Sloan MA, Buchholz D. Stroke in children and sickle-cell disease: Baltimore-Washington Cooperative Young Stroke Study. Neurology. 1998; 51(1): 169-76
Hemorrhagic Stroke	National Center for Health Statistics (NCHS) Centers for Disease Control and Prevention (CDC). United States National Health and Nutrition Examination Survey 2011-2012. Hyattsville, United States: National Center for Health Statistics (NCHS) Centers for Disease Control and Prevention (CDC), 2013
Hemorrhagic Stroke	National Center for Health Statistics (NCHS), Centers for Disease Control and Prevention (CDC), United States Census Bureau. United States National Health Interview Survey 2012. Hyattsville, United States: National Center for Health Statistics (NCHS), Centers for Disease Control and Prevention (CDC), 2013
Hemorrhagic Stroke	National Institute of Statistics and Censuses (Ecuador). Ecuador Hospital Inpatient Discharges 2012. Quito, Ecuador: National Institute of Statistics and Censuses (Ecuador), 2013
Hemorrhagic Stroke	Talaei M, Sarrafzadegan N, Sadeghi M, Oveisgharan S, Marshall T, Thomas GN, Iranipour R. Incidence of cardiovascular diseases in an Iranian population: the Isfahan Cohort Study. Arch Iran Med. 2013; 16(3): 138-44
Hemorrhagic Stroke	Russia Longitudinal Monitoring Survey (RLMS-HSE), Round XVIII 2009. National Research University Higher School of Economics, ZAO Demoscope, Carolina Population Center, Univeristy of North Carolina at Chapel Hill, Institute of Sociology, Russian Academy of Sciences
Hemorrhagic Stroke	Russia Longitudinal Monitoring Survey (RLMS-HSE), Round XVII 2008. National Research University Higher School of Economics, ZAO Demoscope, Carolina Population Center, Univeristy of North Carolina at Chapel Hill, Institute of Sociology, Russian Academy of Sciences
Hemorrhagic Stroke	Russia Longitudinal Monitoring Survey (RLMS-HSE), Round XVI 2007. National Research University Higher School of Economics, ZAO Demoscope, Carolina Population Center, Univeristy of North Carolina at Chapel Hill, Institute of Sociology, Russian Academy of Sciences
Hemorrhagic Stroke	Russia Longitudinal Monitoring Survey (RLMS-HSE), Round XV 2006. National Research University Higher School of Economics, ZAO Demoscope, Carolina Population Center, Univeristy of North Carolina at Chapel Hill, Institute of Sociology, Russian Academy of Sciences
Hemorrhagic Stroke	Russia Longitudinal Monitoring Survey (RLMS-HSE), Round VI 1995. National Research University Higher School of Economics, ZAO Demoscope, Carolina Population Center, Univeristy of North Carolina at Chapel Hill, Institute of Sociology, Russian Academy of Sciences
Hemorrhagic Stroke	Russia Longitudinal Monitoring Survey (RLMS-HSE), Round V 1994. National Research University Higher School of Economics, ZAO Demoscope, Carolina Population Center, Univeristy of North Carolina at Chapel Hill, Institute of Sociology, Russian Academy of Sciences
Hemorrhagic Stroke	Zahuranec DB, Brown DL, Lisabeth LD, Morgenstern LB. Is it time for a large, collaborative study of pediatric stroke?. Stroke. 2005; 36(9): 1825-9
Hemorrhagic Stroke	Bonita R, Solomon N, Broad JB. Prevalence of Stroke and Stroke-Related Disability: Estimates From the Auckland Stroke Studies. Stroke. 1997; 28(10): 1898-902
Hemorrhagic Stroke	Díaz-Guzmán J, Bermejo-Pareja F, Benito-León J, Vega S, Gabriel R, Medrano MJ. Prevalence of stroke and transient ischemic attack in three elderly populations of central Spain. Neuroepidemiology. 2008; 30(4): 247-53
Hemorrhagic Stroke	Delbari A, Salman Roghani R, Tabatabaei SS, Rahgozar M, Lokk J. Stroke epidemiology and one-month fatality among an urban population in Iran. Int J Stroke. 2011; 6(3): 195-200
Hemorrhagic Stroke	Irwin J, Wright P, Reeve P. Temporal trends and clinical characteristics of spontaneous intracerebral haemorrhage in the Waikato region of New Zealand: a hospital-based analysis. N Z Med J. 2011; 124(1345): 16-25
Hemorrhagic Stroke	Institute for Health Metrics and Evaluation (IHME), Ministry of Health (Saudi Arabia). Saudi Arabia Health Interview Survey 2013
Hemorrhagic Stroke	Health and Retirement Study, (Biennial 2012) public use dataset. Produced and distributed by the University of Michigan with funding from the National Institute on Aging (grant number NIA U01AG009740). Ann Arbor, MI, (2015)
Hemorrhagic Stroke	Ministry of Health, Social Services and Equality (Spain), Sociological Research Center (Spain). Spain Health Barometer Survey 2004. Madrid, Spain: Ministry of Health, Social Services and Equality (Spain), 2004
Hemorrhagic Stroke	Vemmos KN, Bots ML, Tsibouris PK, Zis VP, Takis CE, Grobbee DE, Stamatelopoulos S. Prognosis of stroke in the south of Greece: 1 year mortality, functional outcome and its determinants: the Arcadia Stroke Registry. J Neurol Neurosurg Psychiatr. 2000; 69(5): 595-600
Hemorrhagic Stroke	National Center for Health Statistics (NCHS), Centers for Disease Control and Prevention (CDC), United States Census Bureau. United States National Health Interview Survey 2013. Hyattsville, United States: National Center for Health Statistics (NCHS), Centers for Disease Control and Prevention (CDC), 2014
Hemorrhagic Stroke	National Institute of Statistics and Censuses (Ecuador). Ecuador Hospital Inpatient Discharges 2013. Quito, Ecuador: National Institute of Statistics and Censuses (Ecuador)
Hemorrhagic Stroke	National Center for Health Statistics (NCHS), Centers for Disease Control and Prevention (CDC). United States National Health and Nutrition Examination Survey 2013-2014. Hyattsville, United States: National Center for Health Statistics (NCHS), Centers for Disease Control and Prevention (CDC)
Hemorrhagic Stroke	International Research Associates (INRA) Europe, World Health Organization (WHO). Spain WHO Multi-country Survey Study on Health and Health System Responsiveness 2000-2001. Geneva, Switzerland: World Health Organization (WHO)
Hemorrhagic Stroke	University of Otago (New Zealand), World Health Organization (WHO). New Zealand WHO Multi-country Survey Study on Health and Health System Responsiveness 2000-2001. Geneva, Switzerland: World Health Organization (WHO)
Hemorrhagic Stroke	International Research Associates (INRA) Europe, World Health Organization (WHO). Romania WHO Multi-country Survey Study on Health and Health System Responsiveness 2000-2001. Geneva, Switzerland: World Health Organization (WHO)
Hemorrhagic Stroke	International Research Associates (INRA) Europe, World Health Organization (WHO). Russia WHO Multi-country Survey Study on Health and Health System Responsiveness 2000-2001. Geneva, Switzerland: World Health Organization (WHO)
Hemorrhagic Stroke	Washington State University, World Health Organization (WHO). United States WHO Multi-country Survey Study on Health and Health System Responsiveness 2000-2001. Geneva, Switzerland: World Health Organization (WHO)

Hemorrhagic Stroke	Börsch-Supan, A. (2015). Survey of Health, Ageing and Retirement in Europe (SHARE) Wave 5. Release version: 1.0.0. SHARE-ERIC. Data set. DOI: 10.6103/SHARE.w5.100
Hemorrhagic Stroke	National Center for Health Statistics (NCHS), Centers for Disease Control and Prevention (CDC). United States National Health Interview Survey 2014. Hyattsville, United States: National Center for Health Statistics (NCHS), Centers for Disease Control and Prevention (CDC), 2015
Hemorrhagic Stroke	Ministry of Health (New Zealand). New Zealand National Minimum Dataset 2000
Hemorrhagic Stroke	Ministry of Health (New Zealand). New Zealand National Minimum Dataset 2001
Hemorrhagic Stroke	Ministry of Health (New Zealand). New Zealand National Minimum Dataset 2002
Hemorrhagic Stroke	Ministry of Health (New Zealand). New Zealand National Minimum Dataset 2003
Hemorrhagic Stroke	Ministry of Health (New Zealand). New Zealand National Minimum Dataset 2004
Hemorrhagic Stroke	Ministry of Health (New Zealand). New Zealand National Minimum Dataset 2005
Hemorrhagic Stroke	Ministry of Health (New Zealand). New Zealand National Minimum Dataset 2006
Hemorrhagic Stroke	Ministry of Health (New Zealand). New Zealand National Minimum Dataset 2007. Wellington, New Zealand: Ministry of Health (New Zealand)
Hemorrhagic Stroke	Ministry of Health (New Zealand). New Zealand National Minimum Dataset 2008. Wellington, New Zealand: Ministry of Health (New Zealand)
Hemorrhagic Stroke	Ministry of Health (New Zealand). New Zealand National Minimum Dataset 2009. Wellington, New Zealand: Ministry of Health (New Zealand)
Hemorrhagic Stroke	Ministry of Health (New Zealand). New Zealand National Minimum Dataset 2010. Wellington, New Zealand: Ministry of Health (New Zealand)
Hemorrhagic Stroke	Ministry of Health (New Zealand). New Zealand National Minimum Dataset 2011. Wellington, New Zealand: Ministry of Health (New Zealand)
Hemorrhagic Stroke	Ministry of Health (New Zealand). New Zealand National Minimum Dataset 2012. Wellington, New Zealand: Ministry of Health (New Zealand)
Hemorrhagic Stroke	Ministry of Health (New Zealand). New Zealand National Minimum Dataset 2013. Wellington, New Zealand: Ministry of Health (New Zealand)
Hemorrhagic Stroke	Ministry of Health (New Zealand). New Zealand National Minimum Dataset 2014. Wellington, New Zealand: Ministry of Health (New Zealand)
Hemorrhagic Stroke	National Statistical Office of Malawi. Malawi Integrated Household Survey 2013. Washington DC, United States: World Bank, 2015
Hemorrhagic Stroke	Truven Health Analytics. United States MarketScan Commercial Claims and Encounters Database 2010. Ann Arbor, United States: Truven Health Analytics
Hemorrhagic Stroke	Center for Sociological Studies, Lomonosov Moscow State University, Concluzia-Prim Center for Survey Methodology (Moldova), Institute for Advanced Studies (Austria), London School of Hygiene and Tropical Medicine, University of Aberdeen. Russia Health in Times of Transition Household Survey 2010
Hemorrhagic Stroke	Howard G, Cushman M, Howard VJ, Kissela BM, Kleindorfer DO, Moy CS, Switzer J, Woo D. Risk factors for intracerebral hemorrhage: the REasons for geographic and racial differences in stroke (REGARDS) study. Stroke. 2013; 44(5): 1282–7
Hemorrhagic Stroke	National Center for Health Statistics (NCHS), Centers for Disease Control and Prevention (CDC). United States National Hospital Discharge Survey 1988-1992
Hemorrhagic Stroke	National Center for Health Statistics (NCHS), Centers for Disease Control and Prevention (CDC). United States National Hospital Discharge Survey 1993-1997
Hemorrhagic Stroke	National Center for Health Statistics (NCHS), Centers for Disease Control and Prevention (CDC). United States National Hospital Discharge Survey 1998-2002
Hemorrhagic Stroke	National Center for Health Statistics (NCHS), Centers for Disease Control and Prevention (CDC). United States National Hospital Discharge Survey 2003-2007
Hemorrhagic Stroke	National Center for Health Statistics (NCHS), Centers for Disease Control and Prevention (CDC). United States National Hospital Discharge Survey 2008-2010
Hemorrhagic Stroke	National Institute of Statistics and Censuses (Ecuador). Ecuador Hospital Inpatient Discharges 2014. Quito, Ecuador: National Institute of Statistics and Censuses (Ecuador)
Hemorrhagic Stroke	Healthcare Cost and Utilization Project (HCUP), Agency for Healthcare Research and Quality (AHRQ). United States Nationwide Inpatient Sample 2013. Rockville, United States: Healthcare Cost and Utilization Project (HCUP), Agency for Healthcare Research and Quality (AHRQ)
Hemorrhagic Stroke	National Center for Health Statistics (NCHS), Centers for Disease Control and Prevention (CDC). United States National Health Interview Survey 2015. Hyattsville, United States: National Center for Health Statistics (NCHS), Centers for Disease Control and Prevention (CDC), 2016
Hemorrhagic Stroke	and Prevention. National Center for Health Statistics. National Health Interview Survey, 1994: Second Supplement on Aging. ICPSR02563-v3. Ann Arbor, MI: Inter-university Consortium for Political and Social Research [distributor], 2007-02-12. http://doi.org/10.3886/ICPSR02563.v3
Hemorrhagic Stroke	Heikinheimo T, Chimbayo D, Kumwenda JJ, Kampondeni S, Allain TJ. Stroke outcomes in Malawi, a country with high prevalence of HIV: a prospective follow-up study. PLoS One. 2012; 7(3): e33765
Hemorrhagic Stroke	Healthcare Cost and Utilization Project (HCUP), Agency for Healthcare Research and Quality (AHRQ). United States State Inpatient Databases 2003-2007
Hemorrhagic Stroke	Healthcare Cost and Utilization Project (HCUP), Agency for Healthcare Research and Quality (AHRQ). United States State Inpatient Databases 2008-2009
Hemorrhagic Stroke	Ministry of Health (New Zealand). New Zealand National Minimum Dataset 2000-2002
Hemorrhagic Stroke	Ministry of Health (New Zealand). New Zealand National Minimum Dataset 2003-2007
Hemorrhagic Stroke	Ministry of Health (New Zealand). New Zealand National Minimum Dataset 2008-2012
Hemorrhagic Stroke	Ministry of Health (New Zealand). New Zealand National Minimum Dataset 2013-2014
Hemorrhagic Stroke	Ministry of Health (New Zealand). New Zealand National Minimum Dataset 2015. Wellington, New Zealand: Ministry of Health (New Zealand)
Hemorrhagic Stroke	Ministry of Health (Romania). Romania Hospital Inpatient Discharges 2000
Hemorrhagic Stroke	Ministry of Health (Romania). Romania Hospital Inpatient Discharges 2001
Hemorrhagic Stroke	Ministry of Health (Romania). Romania Hospital Inpatient Discharges 2002
Hemorrhagic Stroke	Ministry of Health (Romania). Romania Hospital Inpatient Discharges 2003
Hemorrhagic Stroke	Ministry of Health (Romania). Romania Hospital Inpatient Discharges 2004
Hemorrhagic Stroke	Ministry of Health (Romania). Romania Hospital Inpatient Discharges 2005
Hemorrhagic Stroke	National School of Public Health (Greece), World Health Organization (WHO). Greece WHO Multi-country Survey Study on Health and Health System Responsiveness 2000-2001
Hemorrhagic Stroke	Healthcare Cost and Utilization Project (HCUP), Agency for Healthcare Research and Quality (AHRQ). United States Nationwide Inpatient Sample 2014. Rockville, United States: Healthcare Cost and Utilization Project (HCUP), Agency for Healthcare Research and Quality (AHRQ)
Hemorrhagic Stroke	Healthcare Cost and Utilization Project (HCUP), Agency for Healthcare Research and Quality (AHRQ). United States State Inpatient Databases 2011. Rockville, United States: Healthcare Cost and Utilization Project (HCUP), Agency for Healthcare Research and Quality (AHRQ)
Hemorrhagic Stroke	Healthcare Cost and Utilization Project (HCUP), Agency for Healthcare Research and Quality (AHRQ). United States State Inpatient Databases 2012. Rockville, United States: Healthcare Cost and Utilization Project (HCUP), Agency for Healthcare Research and Quality (AHRQ)
Hemorrhagic Stroke	Healthcare Cost and Utilization Project (HCUP), Agency for Healthcare Research and Quality (AHRQ). United States State Inpatient Databases 2013. Rockville, United States: Healthcare Cost and Utilization Project (HCUP), Agency for Healthcare Research and Quality (AHRQ)
Hemorrhagic Stroke	Healthcare Cost and Utilization Project (HCUP), Agency for Healthcare Research and Quality (AHRQ). United States State Inpatient Databases 2014. Rockville, United States: Healthcare Cost and Utilization Project (HCUP), Agency for Healthcare Research and Quality (AHRQ)

Hemorrhagic Stroke	Healthcare Cost and Utilization Project (HCUP), Agency for Healthcare Research and Quality (AHRQ). United States Nationwide Inpatient Sample 2015. Rockville, United States: Healthcare Cost and Utilization Project (HCUP), Agency for Healthcare Research and Quality (AHRQ)
Hemorrhagic Stroke	Ministry of Health (Romania). Romania Hospital Inpatient Discharges 1980
Hemorrhagic Stroke	Ministry of Health and Social Security (Spain), National Statistics Institute (Spain). Spain Statistics on Health Establishments Providing Inpatient Care 1972
Hemorrhagic Stroke	Ministry of Health and Social Security (Spain), National Statistics Institute (Spain). Spain Statistics on Health Establishments Providing Inpatient Care 1973
Hemorrhagic Stroke	Ministry of Health and Social Security (Spain), National Statistics Institute (Spain). Spain Statistics on Health Establishments Providing Inpatient Care 1974
Hemorrhagic Stroke	Ministry of Health and Social Security (Spain), National Statistics Institute (Spain). Spain Statistics on Health Establishments Providing Inpatient Care 1975
Hemorrhagic Stroke	Ministry of Health and Social Security (Spain), National Statistics Institute (Spain). Spain Statistics on Health Establishments Providing Inpatient Care 1976
Hemorrhagic Stroke	Ministry of Health and Social Security (Spain), National Statistics Institute (Spain). Spain Statistics on Health Establishments Providing Inpatient Care 1977
Hemorrhagic Stroke	Ministry of Health and Social Security (Spain), National Statistics Institute (Spain). Spain Statistics on Health Establishments Providing Inpatient Care 1978
Hemorrhagic Stroke	Ministry of Health and Social Security (Spain), National Statistics Institute (Spain). Spain Statistics on Health Establishments Providing Inpatient Care 1979
Hemorrhagic Stroke	Ministry of Health and Social Security (Spain), National Statistics Institute (Spain). Spain Statistics on Health Establishments Providing Inpatient Care 1980
Hemorrhagic Stroke	Ministry of Health and Social Security (Spain), National Statistics Institute (Spain). Spain Statistics on Health Establishments Providing Inpatient Care 1981
Hemorrhagic Stroke	Ministry of Health and Consumer Affairs (Spain), National Statistics Institute (Spain). Spain Statistics on Health Establishments Providing Inpatient Care 1982
Hemorrhagic Stroke	Ministry of Health and Consumer Affairs (Spain), National Statistics Institute (Spain). Spain Statistics on Health Establishments Providing Inpatient Care 1983
Hemorrhagic Stroke	Ministry of Health and Consumer Affairs (Spain), National Statistics Institute (Spain). Spain Statistics on Health Establishments Providing Inpatient Care 1984
Hemorrhagic Stroke	Ministry of Health and Consumer Affairs (Spain), National Statistics Institute (Spain). Spain Statistics on Health Establishments Providing Inpatient Care 1985
Hemorrhagic Stroke	Ministry of Health and Consumer Affairs (Spain), National Statistics Institute (Spain). Spain Statistics on Health Establishments Providing Inpatient Care 1986
Hemorrhagic Stroke	Ministry of Health and Consumer Affairs (Spain), National Statistics Institute (Spain). Spain Statistics on Health Establishments Providing Inpatient Care 1987
Hemorrhagic Stroke	Ministry of Health and Consumer Affairs (Spain), National Statistics Institute (Spain). Spain Statistics on Health Establishments Providing Inpatient Care 1988
Hemorrhagic Stroke	Ministry of Health and Consumer Affairs (Spain), National Statistics Institute (Spain). Spain Statistics on Health Establishments Providing Inpatient Care 1989
Hemorrhagic Stroke	Ministry of Health and Consumer Affairs (Spain), National Statistics Institute (Spain). Spain Statistics on Health Establishments Providing Inpatient Care 1990
Hemorrhagic Stroke	Ministry of Health and Consumer Affairs (Spain), National Statistics Institute (Spain). Spain Statistics on Health Establishments Providing Inpatient Care 1991
Hemorrhagic Stroke	Ministry of Health and Consumer Affairs (Spain), National Statistics Institute (Spain). Spain Statistics on Health Establishments Providing Inpatient Care 1992
Hemorrhagic Stroke	Ministry of Health and Consumer Affairs (Spain), National Statistics Institute (Spain). Spain Statistics on Health Establishments Providing Inpatient Care 1993
Hemorrhagic Stroke	Ministry of Health and Consumer Affairs (Spain), National Statistics Institute (Spain). Spain Statistics on Health Establishments Providing Inpatient Care 1994
Hemorrhagic Stroke	Ministry of Health and Consumer Affairs (Spain), National Statistics Institute (Spain). Spain Statistics on Health Establishments Providing Inpatient Care 1995
Hemorrhagic Stroke	Ministry of Health and Consumer Affairs (Spain), National Statistics Institute (Spain). Spain Statistics on Health Establishments Providing Inpatient Care 1996
Hemorrhagic Stroke	Ministry of Health and Consumer Affairs (Spain). Spain Statistics on Health Establishments Providing Inpatient Care 1997. Madrid, Spain: Ministry of Health, Social Services and Equality (Spain)
Hemorrhagic Stroke	Ministry of Health and Consumer Affairs (Spain). Spain Statistics on Health Establishments Providing Inpatient Care 1998. Madrid, Spain: Ministry of Health, Social Services and Equality (Spain)
Hemorrhagic Stroke	Ministry of Health and Consumer Affairs (Spain). Spain Statistics on Health Establishments Providing Inpatient Care 1999. Madrid, Spain: Ministry of Health, Social Services and Equality (Spain)
Hemorrhagic Stroke	Ministry of Health and Consumer Affairs (Spain). Spain Statistics on Health Establishments Providing Inpatient Care 2000. Madrid, Spain: Ministry of Health, Social Services and Equality (Spain)
Hemorrhagic Stroke	Ministry of Health and Consumer Affairs (Spain). Spain Statistics on Health Establishments Providing Inpatient Care 2001. Madrid, Spain: Ministry of Health, Social Services and Equality (Spain)
Hemorrhagic Stroke	Ministry of Health and Consumer Affairs (Spain). Spain Statistics on Health Establishments Providing Inpatient Care 2002. Madrid, Spain: Ministry of Health, Social Services and Equality (Spain)
Hemorrhagic Stroke	Ministry of Health and Consumer Affairs (Spain). Spain Statistics on Health Establishments Providing Inpatient Care 2003. Madrid, Spain: Ministry of Health, Social Services and Equality (Spain)
Hemorrhagic Stroke	Ministry of Health and Consumer Affairs (Spain). Spain Statistics on Health Establishments Providing Inpatient Care 2004. Madrid, Spain: Ministry of Health, Social Services and Equality (Spain)
Hemorrhagic Stroke	Ministry of Health and Consumer Affairs (Spain). Spain Statistics on Health Establishments Providing Inpatient Care 2005. Madrid, Spain: Ministry of Health, Social Services and Equality (Spain)
Hemorrhagic Stroke	Ministry of Health and Consumer Affairs (Spain). Spain Statistics on Health Establishments Providing Inpatient Care 2006. Madrid, Spain: Ministry of Health, Social Services and Equality (Spain)
Hemorrhagic Stroke	Ministry of Health and Consumer Affairs (Spain). Spain Statistics on Health Establishments Providing Inpatient Care 2007. Madrid, Spain: Ministry of Health, Social Services and Equality (Spain)

Hemorrhagic Stroke	Ministry of Health and Consumer Affairs (Spain). Spain Statistics on Health Establishments Providing Inpatient Care 2008. Madrid, Spain: Ministry of Health, Social Services and Equality (Spain)
Hemorrhagic Stroke	Ministry of Health and Consumer Affairs (Spain). Spain Statistics on Health Establishments Providing Inpatient Care 2009. Madrid, Spain: Ministry of Health, Social Services and Equality (Spain)
Hemorrhagic Stroke	Ministry of Health, Social Services and Equality (Spain). Spain Statistics on Specialized Healthcare Centers 2010. Madrid, Spain: Ministry of Health, Social Services and Equality (Spain)
Hemorrhagic Stroke	Ministry of Health, Social Services and Equality (Spain). Spain Statistics on Specialized Healthcare Centers 2011. Madrid, Spain: Ministry of Health, Social Services and Equality (Spain)
Hemorrhagic Stroke	Ministry of Health, Social Services and Equality (Spain). Spain Statistics on Specialized Healthcare Centers 2012. Madrid, Spain: Ministry of Health, Social Services and Equality (Spain)
Hemorrhagic Stroke	Ministry of Health, Social Services and Equality (Spain). Spain Statistics on Specialized Healthcare Centers 2013. Madrid, Spain: Ministry of Health, Social Services and Equality (Spain)
Hemorrhagic Stroke	Ministry of Health, Social Services and Equality (Spain). Spain Statistics on Specialized Healthcare Centers 2014. Madrid, Spain: Ministry of Health, Social Services and Equality (Spain)
Hemorrhagic Stroke	Ministry of Health (Romania). Romania Hospital Inpatient Discharges 1985
Hemorrhagic Stroke	Ministry of Health (Romania). Romania Hospital Inpatient Discharges 1986
Hemorrhagic Stroke	Ministry of Health (Romania). Romania Hospital Inpatient Discharges 1987
Hemorrhagic Stroke	Ministry of Health (Romania). Romania Hospital Inpatient Discharges 1988
Hemorrhagic Stroke	Ministry of Health (Romania). Romania Hospital Inpatient Discharges 1989
Hemorrhagic Stroke	Ministry of Health (Romania). Romania Hospital Inpatient Discharges 1990
Hemorrhagic Stroke	Ministry of Health (Romania). Romania Hospital Inpatient Discharges 1991
Hemorrhagic Stroke	Ministry of Health (Romania). Romania Hospital Inpatient Discharges 1992
Hemorrhagic Stroke	Ministry of Health (Romania). Romania Hospital Inpatient Discharges 1993
Hemorrhagic Stroke	Ministry of Health (Romania). Romania Hospital Inpatient Discharges 1994
Hemorrhagic Stroke	Ministry of Health (Romania). Romania Hospital Inpatient Discharges 1995
Hemorrhagic Stroke	Ministry of Health (Romania). Romania Hospital Inpatient Discharges 1996
Hemorrhagic Stroke	Ministry of Health (Romania). Romania Hospital Inpatient Discharges 1997
Hemorrhagic Stroke	Ministry of Health (Romania). Romania Hospital Inpatient Discharges 1998
Hemorrhagic Stroke	Ministry of Health (Romania). Romania Hospital Inpatient Discharges 1999
Hemorrhagic Stroke	Ministry of Health (Romania). Romania Hospital Inpatient Discharges 2013
Hemorrhagic Stroke	Bureau of Statistics (Guyana), World Bank. Guyana Living Standards Measurement Survey 1992-1993
Hemorrhagic Stroke	Ministry of Statistics and Programme Implementation (India). India National Sample Survey Round 52 1995-1996. New Delhi, India: Ministry of Statistics and Programme Implementation (India)
Hemorrhagic Stroke	Ministry of Statistics and Programme Implementation (India). India National Sample Survey Round 60 2004. New Delhi, India: Ministry of Statistics and Programme Implementation (India)
Hemorrhagic Stroke	National Institute of Statistics and Informatics (Peru), World Bank (WB). Peru Living Standards Measurement Survey 1990
Hemorrhagic Stroke	National Institute of Statistics and Informatics (INEI) (Peru), World Bank. Peru Living Standards Measurement Survey 1991. Washington DC, United States: World Bank
Hemorrhagic Stroke	National Institute of Statistics and Informatics (Peru), World Bank (WB). Peru Living Standards Measurement Survey 1994
Hemorrhagic Stroke	Ministry of Social Affairs (Serbia), World Bank. Yugoslavia, Federal Republic - Serbia Living Standards Measurement Survey 2002. Washington DC, United States: World Bank
Hemorrhagic Stroke	Ministry of Social Affairs (Serbia), World Bank. Serbia and Montenegro - Serbia Living Standards Measurement Survey 2003. Washington DC, United States: World Bank
Hemorrhagic Stroke	International Institute for Population Sciences (India), World Health Organization (WHO). India World Health Survey 2003. Geneva, Switzerland: World Health Organization (WHO), 2005
Hemorrhagic Stroke	World Health Organization (WHO). Malaysia World Health Survey 2003. Geneva, Switzerland: World Health Organization (WHO), 2005
Hemorrhagic Stroke	World Health Organization (WHO). Mauritius World Health Survey 2003. Geneva, Switzerland: World Health Organization (WHO), 2005
Hemorrhagic Stroke	World Health Organization (WHO). Sweden World Health Survey 2003. Geneva, Switzerland: World Health Organization (WHO), 2005
Hemorrhagic Stroke	World Health Organization (WHO). Ukraine World Health Survey 2002-2003. Geneva, Switzerland: World Health Organization (WHO), 2005
Hemorrhagic Stroke	National Institute of Statistics and Informatics (INEI) (Peru), United Nations Economic Commission for Latin America and the Caribbean (CEPAL), Institute of Research for Development (France). Peru National Household Survey 2007. Lima, Peru: National Institute of Statistics and Informatics (INEI) (Peru)
Hemorrhagic Stroke	National Institute of Statistics and Informatics (INEI) (Peru), United Nations Economic Commission for Latin America and the Caribbean (CEPAL), Institute of Research for Development (France). Peru National Household Survey 2008. Lima, Peru: National Institute of Statistics and Informatics (INEI) (Peru)
Hemorrhagic Stroke	National Institute of Statistics and Informatics (INEI) (Peru). Peru National Household Survey 2009. Lima, Peru: National Institute of Statistics and Informatics (INEI) (Peru)
Hemorrhagic Stroke	National Institute of Statistics and Informatics (INEI) (Peru). Peru National Household Survey 2010. Lima, Peru: National Institute of Statistics and Informatics (INEI) (Peru)
Hemorrhagic Stroke	National Institute of Statistics and Informatics (INEI) (Peru). Peru National Household Survey, Second Quarter 1998. Lima, Peru: National Institute of Statistics and Informatics (INEI) (Peru)
Hemorrhagic Stroke	National Institute of Statistics and Informatics (INEI) (Peru). Peru National Household Survey, Second Quarter 1999. Lima, Peru: National Institute of Statistics and Informatics (INEI) (Peru)
Hemorrhagic Stroke	National Institute of Statistics (Albania), World Bank (WB). Albania Living Standards Measurement Survey 2002. Washington DC, United States: World Bank (WB)
Hemorrhagic Stroke	National Institute of Statistics (Albania), World Bank (WB). Albania Living Standards Measurement Survey 2005. Washington DC, United States: World Bank (WB)
Hemorrhagic Stroke	National Institute of Statistics and Informatics (INEI) (Peru). Peru National Household Survey, Second Quarter 2000. Lima, Peru: National Institute of Statistics and Informatics (INEI) (Peru)
Hemorrhagic Stroke	Ministry of Social Affairs (Serbia), World Bank. Serbia Living Standards Measurement Survey 2007. Washington DC, United States: World Bank
Hemorrhagic Stroke	National Institute of Statistics and Informatics (INEI) (Peru). Peru National Household Survey 2005. Lima, Peru: National Institute of Statistics and Informatics (INEI) (Peru)
Hemorrhagic Stroke	National Institute of Statistics and Informatics (INEI) (Peru). Peru National Household Survey 2006. Lima, Peru: National Institute of Statistics and Informatics (INEI) (Peru)

Hemorrhagic Stroke	International Institute for Population Sciences (India), World Health Organization (WHO). India WHO Study on Global Ageing and Adult Health 2007. Geneva, Switzerland: World Health Organization (WHO), 2007
Hemorrhagic Stroke	Ministry of Health (Syria), World Health Organization (WHO). Syria WHO Multi-country Survey Study on Health and Health System Responsiveness 2000-2001
Hemorrhagic Stroke	National Institute of Statistics (Niger), World Bank. Niger National Survey on Household Living Conditions and Agriculture 2011-2012
Hemorrhagic Stroke	Sridharan SE, Unnikrishnan JP, Sukumaran S, Sylaja PN, Nayak SD, Sarma PS, Radhakrishnan K. Incidence, Types, Risk Factors, and Outcome of Stroke in a Developing Country. Stroke. 2009; 40(4): 1212-8
Hemorrhagic Stroke	Appelros P, Nydevik I, Seiger Åke, Terént A. High Incidence Rates of Stroke in Orebro, Sweden: Further Support for Regional Incidence Differences within Scandinavia. Cerebrovasc Dis. 2002; 14(3-4): 161-8
Hemorrhagic Stroke	Hallström B, Jönsson A-C, Nerbrand C, Norrving B, Lindgren A. Stroke Incidence and Survival in the Beginning of the 21st Century in Southern Sweden: Comparisons With the Late 20th Century and Projections Into the Future. Stroke. 2008; 39(1): 10-5
Hemorrhagic Stroke	Dalal PM, Malik S, Bhattacharjee M, Trivedi ND, Vairale J, Bhat P, Deshmukh S, Khandelwal K, Mathur VD. Population-based stroke survey in Mumbai, India: incidence and 28-day case fatality. Neuroepidemiology. 2008; 31(4): 254-61
Hemorrhagic Stroke	Mihálka L, Smolanka V, Bulecza B, Mulesa S, Bereczki D. A Population Study of Stroke in West Ukraine: Incidence, Stroke Services, and 30-Day Case Fatality. Stroke. 2001; 32(10): 2227-31
Hemorrhagic Stroke	Terént A. Trends in Stroke Incidence and 10-Year Survival in Söderhamn, Sweden, 1975-2001. Stroke. 2003; 34(6): 1353-8
Hemorrhagic Stroke	Statistics Sweden, Swedish National Institute of Public Health. Sweden National Survey of Public Health 2009
Hemorrhagic Stroke	Statistics Sweden, Swedish National Institute of Public Health. Sweden National Survey of Public Health 2007
Hemorrhagic Stroke	Institute for Public Health, Ministry of Health (Malaysia). Malaysia National Health And Morbidity Survey 2006. Kuala Lumpur, Malaysia: Institute for Public Health, Ministry of Health (Malaysia)
Hemorrhagic Stroke	Northern Ireland Statistics and Research Agency. Central Survey Unit, Northern Ireland Health and Social Wellbeing Survey, 2001 [computer file]. Colchester, Essex: UK Data Archive [distributor], October 2002. SN: 4590 , http://dx.doi.org/10.5255/UKDA-SN-4590-1
Hemorrhagic Stroke	Northern Ireland Statistics and Research Agency. Central Survey Unit, Northern Ireland Health and Social Wellbeing Survey, 2005-2006 [computer file]. Colchester, Essex: UK Data Archive [distributor], October 2007. SN: 5710, http://dx.doi.org/10.5255/UKDA-SN-5710-1
Hemorrhagic Stroke	Northern Ireland Statistics and Research Agency. Central Survey Unit, Northern Ireland Health and Social Wellbeing Survey, 1997 [computer file]. Colchester, Essex: UK Data Archive [distributor], October 2002. SN: 4589, http://dx.doi.org/10.5255/UKDA-SN-4589-1
Hemorrhagic Stroke	Northern Ireland Statistics and Research Agency (NISRA). United Kingdom - Northern Ireland Health Survey 2010-2011 - UK Data Service
Hemorrhagic Stroke	Das SK, Banerjee TK, Biswas A, Roy T, Raut DK, Mukherjee CS, Chaudhuri A, Hazra A, Roy J. A Prospective Community-Based Study of Stroke in Kolkata, India. Stroke. 2007; 38(3): 906-10
Hemorrhagic Stroke	Banerjee TK, Mukherjee CS, Sarkhel A. Stroke in the urban population of Calcutta – an epidemiological study. Neuroepidemiology. 2001; 20(3): 201-7
Hemorrhagic Stroke	Dhamija RK, Dhamija SB. Prevalence of stroke in rural community – an overview of Indian experience. J Assoc Physicians India. 1998; 46(4): 351-4
Hemorrhagic Stroke	Koul R, Motta A, Razdan S. Epidemiology of young strokes in rural Kashmir, India. Acta Neurol Scand. 1990; 82(1): 1-3
Hemorrhagic Stroke	Saha SP, Bhattacharya S, Das SK, Maity B, Roy T, Raut DK. Epidemiological study of neurological disorders in a rural population of Eastern India. J Indian Med Assoc. 2003; 101(5): 299-304
Hemorrhagic Stroke	National Board of Health and Welfare (Sweden). Sweden National Patient Register 1998. Stockholm, Sweden: National Board of Health and Welfare (Sweden)
Hemorrhagic Stroke	National Board of Health and Welfare (Sweden). Sweden National Patient Register 1999. Stockholm, Sweden: National Board of Health and Welfare (Sweden)
Hemorrhagic Stroke	National Board of Health and Welfare (Sweden). Sweden National Patient Register 2011. Stockholm, Sweden: National Board of Health and Welfare (Sweden)
Hemorrhagic Stroke	National Board of Health and Welfare (Sweden). Sweden National Patient Register 2012. Stockholm, Sweden: National Board of Health and Welfare (Sweden)
Hemorrhagic Stroke	Statistics Sweden, Swedish National Institute of Public Health. Sweden National Survey of Public Health 2008
Hemorrhagic Stroke	Hilmarsson A, Kjartansson O, Olafsson E. Incidence of First Stroke A Population Study in Iceland. Stroke. 2013; 44(6): 1714-6
Hemorrhagic Stroke	Pilot Study, 2005 (Data Set 27-28, Cunningham, Shayna.) [machine-readable data file and documentation]. Geneva, Switzerland: World Health Organization (Producer). Los Altos, CA: Sociometrics Corporation, Data Archive of Social Research on Aging (Producer & Distributor)
Hemorrhagic Stroke	Statistics Sweden, Swedish National Institute of Public Health. Sweden National Survey of Public Health 2012
Hemorrhagic Stroke	Statistics Sweden, Swedish National Institute of Public Health. Sweden National Survey of Public Health 2010
Hemorrhagic Stroke	Statistics Sweden, Swedish National Institute of Public Health. Sweden National Survey of Public Health 2004
Hemorrhagic Stroke	Statistics Sweden, Swedish National Institute of Public Health. Sweden National Survey of Public Health 2005
Hemorrhagic Stroke	Statistics Sweden, Swedish National Institute of Public Health. Sweden National Survey of Public Health 2011
Hemorrhagic Stroke	Lindmark A, Glader E-L, Asplund K, Norrving B, Eriksson M, Riks-Stroke Collaboration. Socioeconomic disparities in stroke case fatality--Observations from Riks-Stroke, the Swedish stroke register. Int J Stroke. 2014; 9(4): 429–36
Hemorrhagic Stroke	Trinity College Dublin. Ireland Longitudinal Study on Ageing 2009-2011. Dublin, Ireland: Irish Social Science Data Archive, University College Dublin
Hemorrhagic Stroke	Statistics Sweden, Swedish National Institute of Public Health. Sweden National Survey of Public Health 2013
Hemorrhagic Stroke	Public Health Agency of Sweden, Statistics Sweden. Sweden National Survey of Public Health 2014
Hemorrhagic Stroke	International Research Associates (INRA) Europe, World Health Organization (WHO). Iceland WHO Multi-country Survey Study on Health and Health System Responsiveness 2000-2001. Geneva, Switzerland: World Health Organization (WHO)
Hemorrhagic Stroke	International Research Associates (INRA) Europe, World Health Organization (WHO). Sweden WHO Multi-country Survey Study on Health and Health System Responsiveness 2000-2001. Geneva, Switzerland: World Health Organization (WHO)
Hemorrhagic Stroke	Kiev International Institute of Sociology, World Health Organization (WHO). Ukraine WHO Multi-country Survey Study on Health and Health System Responsiveness 2000-2001. Geneva, Switzerland: World Health Organization (WHO)
Hemorrhagic Stroke	Gallup Europe, World Health Organization (WHO). Venezuela WHO Multi-country Survey Study on Health and Health System Responsiveness 2000-2001. Geneva, Switzerland: World Health Organization (WHO)
Hemorrhagic Stroke	Börsch-Supan, A. (2013). Survey of Health, Ageing and Retirement in Europe (SHARE) Wave 2. Release version: 2.6.0. SHARE-ERIC. Data set. DOI: 10.6103/SHARE.w2.260
Hemorrhagic Stroke	Börsch-Supan, A. (2013). Survey of Health, Ageing and Retirement in Europe (SHARE) Wave 4. Release version: 1.1.1. SHARE-ERIC. Data set. DOI: 10.6103/SHARE.w4.111
Hemorrhagic Stroke	Börsch-Supan, A. (2015). Survey of Health, Ageing and Retirement in Europe (SHARE) Wave 5. Release version: 1.0.0. SHARE-ERIC. Data set. DOI: 10.6103/SHARE.w5.100
Hemorrhagic Stroke	Government of India, Ministry of Statistics and Programme Implementation (India). India National Sample Survey Round 71 2014. New Delhi, India: Ministry of Statistics and Programme Implementation (India)
Hemorrhagic Stroke	Armenian Sociological Association, Concluzia-Prim Center for Survey Methodology (Moldova), Institute for Advanced Studies (Austria), London School of Hygiene and Tropical Medicine, University of Aberdeen. Armenia Health in Times of Transition Household Survey 2010

Hemorrhagic Stroke	Zhi X, Joas E, Waern M, Östling S, Börjesson-Hanson A, Skoog I. Prevalence of cardiovascular disorders and risk factors in two 75-year-old birth cohorts examined in 1976-1977 and 2005-2006. Aging Clin Exp Res. 2013; 25(4): 377-83
Hemorrhagic Stroke	Concluzia-Prim Center for Survey Methodology (Moldova), East-Ukranian Foundation For Social Research, Institute for Advanced Studies (Austria), London School of Hygiene and Tropical Medicine, University of Aberdeen. Ukraine Health in Times of Transition Household Survey 2010
Hemorrhagic Stroke	National Board of Health and Welfare (Sweden). Sweden National Patient Register 1998-2002
Hemorrhagic Stroke	National Board of Health and Welfare (Sweden). Sweden National Patient Register 2003-2007
Hemorrhagic Stroke	National Board of Health and Welfare (Sweden). Sweden National Patient Register 2008-2012
Hemorrhagic Stroke	All India Institute of Medical Sciences, New Delhi (AIIMS), Bangur Institute of Neurology (India), Indian Statistical Institute, National Neurosciences Center, Calcutta (India). India - Kolkata Study for Epidemiology of Neurological Disorders 2003-2004
Hemorrhagic Stroke	Institute for Public Health, Ministry of Health (Malaysia). Malaysia National Health and Morbidity Survey 2011
Hemorrhagic Stroke	Unnikrishnan JP, Sylaja S, Nayak SD, Radhakrishnan K. India - Trivandrum Stroke Registry 2005. [Unpublished]
Hemorrhagic Stroke	St. John's National Academy of Health Sciences. INdian Stroke ProspectIve REgistry (INSPIRE) Data 2009-2014
Hemorrhagic Stroke	Kalkonde YV, Sahane V, Deshmukh MD, Nila S, Mandava P, Bang A. High Prevalence of Stroke in Rural Gadchiroli, India: A Community-Based Study. Neuroepidemiology. 2016; 46(4): 235-9
Hemorrhagic Stroke	Moodbidri P, Mehmi G, Sharma A, Arora OP, Dhanuka AK, Sobti MK, Sehgal H, Kaur M, Grewal SS, Jhawar SS, Shadangi TN, Arora T, Saxena A, Sachdeva G, Gill JS, Brar RS, Gill A, Bakshi SS, Pawar SS, Singh G, Sikka P, Litoria PK, Sharma M. Incidence, short-term outcome, and spatial distribution of stroke patients in Ludhiana, India.
Hemorrhagic Stroke	Kulshrestha M, Vidyanand. An analysis of the risk factors and the outcomes of cerebrovascular diseases in northern India. J Clin Diagn Res. 2013; 7(1): 127–31
Hemorrhagic Stroke	Appelros P, Terent A. Characteristics of the National Institute of Health Stroke Scale: results from a population-based stroke cohort at baseline and after one year. Cerebrovasc Dis. 2004; 17(1): 21–7
Hemorrhagic Stroke	Zhu L, Fratiglioni L, Guo Z, Aguero-Torres H, Winblad B, Viitanen M. Association of stroke with dementia, cognitive impairment, and functional disability in the very old: a population-based study. Stroke. 1998; 29(10): 2094–9
Hemorrhagic Stroke	Hornsten C, Molander L, Gustafson Y. The prevalence of stroke and the association between stroke and depression among a very old population. Arch Gerontol Geriatr. 2012; 55(3): 555–9
Hemorrhagic Stroke	Institute of Public Health of Serbia. Serbia National Hospital Discharge Database 2012
Hemorrhagic Stroke	Department of Health, Social Services and Public Safety (Northern Ireland), Information Centre for Health and Social Care, NHS, NHS England, NHS Health Scotland, NHS Wales. United Kingdom Hospital Patient and Discharge Data 2006
Hemorrhagic Stroke	Directorate of Health (Iceland). Iceland Hospital Data Registry 2008
Hemorrhagic Stroke	Ministry of Health (Albania). Albania Inpatient Care Discharges per 100 1993
Hemorrhagic Stroke	Ministry of Health (Albania). Albania Inpatient Care Discharges per 100 1994
Hemorrhagic Stroke	Ministry of Health (Albania). Albania Inpatient Care Discharges per 100 1995
Hemorrhagic Stroke	Ministry of Health (Albania). Albania Inpatient Care Discharges per 100 1996
Hemorrhagic Stroke	Ministry of Health (Albania). Albania Inpatient Care Discharges per 100 1997
Hemorrhagic Stroke	Ministry of Health (Albania). Albania Inpatient Care Discharges per 100 1998
Hemorrhagic Stroke	Ministry of Health (Albania). Albania Inpatient Care Discharges per 100 1999
Hemorrhagic Stroke	Ministry of Health (Albania). Albania Inpatient Care Discharges per 100 2000
Hemorrhagic Stroke	Ministry of Health (Albania). Albania Inpatient Care Discharges per 100 2001
Hemorrhagic Stroke	Ministry of Health (Albania). Albania Inpatient Care Discharges per 100 2002
Hemorrhagic Stroke	Ministry of Health (Albania). Albania Inpatient Care Discharges per 100 2003
Hemorrhagic Stroke	Ministry of Health (Albania). Albania Inpatient Care Discharges per 100 2004
Hemorrhagic Stroke	Ministry of Health (Albania). Albania Inpatient Care Discharges per 100 2005
Hemorrhagic Stroke	Ministry of Health (Albania). Albania Inpatient Care Discharges per 100 2006
Hemorrhagic Stroke	Ministry of Health (Albania). Albania Inpatient Care Discharges per 100 2007
Hemorrhagic Stroke	Ministry of Health (Albania). Albania Inpatient Care Discharges per 100 2008
Hemorrhagic Stroke	Ministry of Health (Albania). Albania Inpatient Care Discharges per 100 2009
Hemorrhagic Stroke	Ministry of Health (Albania). Albania Inpatient Care Discharges per 100 2010
Hemorrhagic Stroke	Ministry of Health (Albania). Albania Inpatient Care Discharges per 100 2011
Hemorrhagic Stroke	Ministry of Health (Albania). Albania Inpatient Care Discharges per 100 2012
Hemorrhagic Stroke	Ministry of Health (Albania). Albania Inpatient Care Discharges per 100 2013
Hemorrhagic Stroke	Directorate of Health (Iceland). Iceland Hospital Data Registry 2010
Hemorrhagic Stroke	Directorate of Health (Iceland). Iceland Hospital Data Registry 2011
Hemorrhagic Stroke	Directorate of Health (Iceland). Iceland Hospital Data Registry 2012
Hemorrhagic Stroke	Directorate of Health (Iceland). Iceland Hospital Data Registry 2013
Hemorrhagic Stroke	Directorate of Health (Iceland). Iceland Hospital Data Registry 2014
Hemorrhagic Stroke	Directorate of Health (Iceland). Iceland Hospital Data Registry 1999
Hemorrhagic Stroke	Directorate of Health (Iceland). Iceland Hospital Data Registry 1995
Hemorrhagic Stroke	Directorate of Health (Iceland). Iceland Hospital Data Registry 1994
Hemorrhagic Stroke	Directorate of Health (Iceland). Iceland Hospital Data Registry 1993
Hemorrhagic Stroke	Directorate of Health (Iceland). Iceland Hospital Data Registry 1992
Hemorrhagic Stroke	Directorate of Health (Iceland). Iceland Hospital Data Registry 1991
Hemorrhagic Stroke	Directorate of Health (Iceland). Iceland Hospital Data Registry 1990
Hemorrhagic Stroke	Directorate of Health (Iceland). Iceland Hospital Data Registry 1989
Hemorrhagic Stroke	Directorate of Health (Iceland). Iceland Hospital Data Registry 1988
Hemorrhagic Stroke	Ministry of Health (Armenia). Armenia Inpatient Care Discharges per 100 1980
Hemorrhagic Stroke	Ministry of Health (Armenia). Armenia Inpatient Care Discharges per 100 1981
Hemorrhagic Stroke	Ministry of Health (Armenia). Armenia Inpatient Care Discharges per 100 1982
Hemorrhagic Stroke	Ministry of Health (Armenia). Armenia Inpatient Care Discharges per 100 1983
Hemorrhagic Stroke	Ministry of Health (Armenia). Armenia Inpatient Care Discharges per 100 1984
Hemorrhagic Stroke	Ministry of Health (Armenia). Armenia Inpatient Care Discharges per 100 1985
Hemorrhagic Stroke	Ministry of Health (Armenia). Armenia Inpatient Care Discharges per 100 1986
Hemorrhagic Stroke	Ministry of Health (Armenia). Armenia Inpatient Care Discharges per 100 1987
Hemorrhagic Stroke	Ministry of Health (Armenia). Armenia Inpatient Care Discharges per 100 1988
Hemorrhagic Stroke	Ministry of Health (Armenia). Armenia Inpatient Care Discharges per 100 1989
Hemorrhagic Stroke	Ministry of Health (Armenia). Armenia Inpatient Care Discharges per 100 1990

Hemorrhagic Stroke	Ministry of Health (Armenia). Armenia Inpatient Care Discharges per 100 1991
Hemorrhagic Stroke	Ministry of Health (Armenia). Armenia Inpatient Care Discharges per 100 1992
Hemorrhagic Stroke	Ministry of Health (Armenia). Armenia Inpatient Care Discharges per 100 1993
Hemorrhagic Stroke	Ministry of Health (Armenia). Armenia Inpatient Care Discharges per 100 1994
Hemorrhagic Stroke	Ministry of Health (Armenia). Armenia Inpatient Care Discharges per 100 1995
Hemorrhagic Stroke	Ministry of Health (Armenia). Armenia Inpatient Care Discharges per 100 1996
Hemorrhagic Stroke	Ministry of Health (Armenia). Armenia Inpatient Care Discharges per 100 1997
Hemorrhagic Stroke	Ministry of Health (Armenia). Armenia Inpatient Care Discharges per 100 1998
Hemorrhagic Stroke	Ministry of Health (Armenia). Armenia Inpatient Care Discharges per 100 1999
Hemorrhagic Stroke	Ministry of Health (Armenia). Armenia Inpatient Care Discharges per 100 2000
Hemorrhagic Stroke	Ministry of Health (Armenia). Armenia Inpatient Care Discharges per 100 2001
Hemorrhagic Stroke	Ministry of Health (Armenia). Armenia Inpatient Care Discharges per 100 2002
Hemorrhagic Stroke	Ministry of Health (Armenia). Armenia Inpatient Care Discharges per 100 2003
Hemorrhagic Stroke	Ministry of Health (Armenia). Armenia Inpatient Care Discharges per 100 2004
Hemorrhagic Stroke	Ministry of Health (Armenia). Armenia Inpatient Care Discharges per 100 2005
Hemorrhagic Stroke	Ministry of Health (Armenia). Armenia Inpatient Care Discharges per 100 2006
Hemorrhagic Stroke	Ministry of Health (Armenia). Armenia Inpatient Care Discharges per 100 2007
Hemorrhagic Stroke	Ministry of Health (Armenia). Armenia Inpatient Care Discharges per 100 2008
Hemorrhagic Stroke	Ministry of Health (Armenia). Armenia Inpatient Care Discharges per 100 2009
Hemorrhagic Stroke	Ministry of Health (Armenia). Armenia Inpatient Care Discharges per 100 2010
Hemorrhagic Stroke	Ministry of Health (Armenia). Armenia Inpatient Care Discharges per 100 2011
Hemorrhagic Stroke	Ministry of Health (Armenia). Armenia Inpatient Care Discharges per 100 2012
Hemorrhagic Stroke	Ministry of Health (Armenia). Armenia Inpatient Care Discharges per 100 2013
Hemorrhagic Stroke	Ministry of Health (Armenia). Armenia Inpatient Care Discharges per 100 2014
Hemorrhagic Stroke	Center for Health Statistics, Ministry of Health (Ukraine). Ukraine Inpatient Care Discharges per 100 1980
Hemorrhagic Stroke	Center for Health Statistics, Ministry of Health (Ukraine). Ukraine Inpatient Care Discharges per 100 1981
Hemorrhagic Stroke	Center for Health Statistics, Ministry of Health (Ukraine). Ukraine Inpatient Care Discharges per 100 1982
Hemorrhagic Stroke	Center for Health Statistics, Ministry of Health (Ukraine). Ukraine Inpatient Care Discharges per 100 1983
Hemorrhagic Stroke	Center for Health Statistics, Ministry of Health (Ukraine). Ukraine Inpatient Care Discharges per 100 1984
Hemorrhagic Stroke	Center for Health Statistics, Ministry of Health (Ukraine). Ukraine Inpatient Care Discharges per 100 1985
Hemorrhagic Stroke	Center for Health Statistics, Ministry of Health (Ukraine). Ukraine Inpatient Care Discharges per 100 1986
Hemorrhagic Stroke	Center for Health Statistics, Ministry of Health (Ukraine). Ukraine Inpatient Care Discharges per 100 1987
Hemorrhagic Stroke	Center for Health Statistics, Ministry of Health (Ukraine). Ukraine Inpatient Care Discharges per 100 1988
Hemorrhagic Stroke	Center for Health Statistics, Ministry of Health (Ukraine). Ukraine Inpatient Care Discharges per 100 1989
Hemorrhagic Stroke	Center for Health Statistics, Ministry of Health (Ukraine). Ukraine Inpatient Care Discharges per 100 1990
Hemorrhagic Stroke	Center for Health Statistics, Ministry of Health (Ukraine). Ukraine Inpatient Care Discharges per 100 1991
Hemorrhagic Stroke	Center for Health Statistics, Ministry of Health (Ukraine). Ukraine Inpatient Care Discharges per 100 1992
Hemorrhagic Stroke	Center for Health Statistics, Ministry of Health (Ukraine). Ukraine Inpatient Care Discharges per 100 1993
Hemorrhagic Stroke	Center for Health Statistics, Ministry of Health (Ukraine). Ukraine Inpatient Care Discharges per 100 1994
Hemorrhagic Stroke	Center for Health Statistics, Ministry of Health (Ukraine). Ukraine Inpatient Care Discharges per 100 1995
Hemorrhagic Stroke	Center for Health Statistics, Ministry of Health (Ukraine). Ukraine Inpatient Care Discharges per 100 1996
Hemorrhagic Stroke	Center for Health Statistics, Ministry of Health (Ukraine). Ukraine Inpatient Care Discharges per 100 1997
Hemorrhagic Stroke	Center for Health Statistics, Ministry of Health (Ukraine). Ukraine Inpatient Care Discharges per 100 1998
Hemorrhagic Stroke	Center for Health Statistics, Ministry of Health (Ukraine). Ukraine Inpatient Care Discharges per 100 1999
Hemorrhagic Stroke	Center for Health Statistics, Ministry of Health (Ukraine). Ukraine Inpatient Care Discharges per 100 2000
Hemorrhagic Stroke	Center for Health Statistics, Ministry of Health (Ukraine). Ukraine Inpatient Care Discharges per 100 2001
Hemorrhagic Stroke	Center for Health Statistics, Ministry of Health (Ukraine). Ukraine Inpatient Care Discharges per 100 2002
Hemorrhagic Stroke	Center for Health Statistics, Ministry of Health (Ukraine). Ukraine Inpatient Care Discharges per 100 2003
Hemorrhagic Stroke	Center for Health Statistics, Ministry of Health (Ukraine). Ukraine Inpatient Care Discharges per 100 2004
Hemorrhagic Stroke	Center for Health Statistics, Ministry of Health (Ukraine). Ukraine Inpatient Care Discharges per 100 2005
Hemorrhagic Stroke	Center for Health Statistics, Ministry of Health (Ukraine). Ukraine Inpatient Care Discharges per 100 2006
Hemorrhagic Stroke	Center for Health Statistics, Ministry of Health (Ukraine). Ukraine Inpatient Care Discharges per 100 2007
Hemorrhagic Stroke	Center for Health Statistics, Ministry of Health (Ukraine). Ukraine Inpatient Care Discharges per 100 2008
Hemorrhagic Stroke	Center for Health Statistics, Ministry of Health (Ukraine). Ukraine Inpatient Care Discharges per 100 2009
Hemorrhagic Stroke	Center for Health Statistics, Ministry of Health (Ukraine). Ukraine Inpatient Care Discharges per 100 2010
Hemorrhagic Stroke	Center for Health Statistics, Ministry of Health (Ukraine). Ukraine Inpatient Care Discharges per 100 2011
Hemorrhagic Stroke	Center for Health Statistics, Ministry of Health (Ukraine). Ukraine Inpatient Care Discharges per 100 2012
Hemorrhagic Stroke	Center for Health Statistics, Ministry of Health (Ukraine). Ukraine Inpatient Care Discharges per 100 2013
Hemorrhagic Stroke	Institute of Public Health of Serbia. Serbia Inpatient Care Discharges Per 100 2010
Hemorrhagic Stroke	Institute of Public Health of Serbia. Serbia Inpatient Care Discharges Per 100 2011
Hemorrhagic Stroke	Institute of Public Health of Serbia. Serbia Inpatient Care Discharges Per 100 2013
Hemorrhagic Stroke	Institute of Public Health of Serbia. Serbia Inpatient Care Discharges Per 100 2014
Hemorrhagic Stroke	Department of Health, Social Services and Public Safety (Northern Ireland), Information Centre for Health and Social Care, NHS, NHS England, NHS Health Scotland, NHS Wales. United Kingdom Hospital Patient and Discharge Data 2012
Hemorrhagic Stroke	Department of Health, Social Services and Public Safety (Northern Ireland), Information Centre for Health and Social Care, NHS, NHS England, NHS Health Scotland, NHS Wales. United Kingdom Hospital Patient and Discharge Data 2013
Hemorrhagic Stroke	Department of Health, Social Services and Public Safety (Northern Ireland), Information Centre for Health and Social Care, NHS, NHS England, NHS Health Scotland, NHS Wales. United Kingdom Hospital Patient and Discharge Data 2014
Hemorrhagic Stroke	National Board of Health and Welfare (Sweden). Sweden National Patient Register 1997
Hemorrhagic Stroke	National Board of Health and Welfare (Sweden). Sweden National Patient Register 1996. Stockholm, Sweden: National Board of Health and Welfare (Sweden)
Hemorrhagic Stroke	National Board of Health and Welfare (Sweden). Sweden National Patient Register 1995. Stockholm, Sweden: National Board of Health and Welfare (Sweden)
Hemorrhagic Stroke	National Board of Health and Welfare (Sweden). Sweden National Patient Register 1994. Stockholm, Sweden: National Board of Health and Welfare (Sweden)

Hemorrhagic Stroke	National Board of Health and Welfare (Sweden). Sweden National Patient Register 1993. Stockholm, Sweden: National Board of Health and Welfare (Sweden)
Hemorrhagic Stroke	National Board of Health and Welfare (Sweden). Sweden National Patient Register 1992. Stockholm, Sweden: National Board of Health and Welfare (Sweden)
Hemorrhagic Stroke	National Board of Health and Welfare (Sweden). Sweden National Patient Register 1991. Stockholm, Sweden: National Board of Health and Welfare (Sweden)
Hemorrhagic Stroke	National Board of Health and Welfare (Sweden). Sweden National Patient Register 1990. Stockholm, Sweden: National Board of Health and Welfare (Sweden)
Hemorrhagic Stroke	National Board of Health and Welfare (Sweden). Sweden National Patient Register 1989. Stockholm, Sweden: National Board of Health and Welfare (Sweden)
Hemorrhagic Stroke	National Board of Health and Welfare (Sweden). Sweden National Patient Register 1988. Stockholm, Sweden: National Board of Health and Welfare (Sweden)
Hemorrhagic Stroke	National Board of Health and Welfare (Sweden). Sweden National Patient Register 1987. Stockholm, Sweden: National Board of Health and Welfare (Sweden)
Hemorrhagic Stroke	National Board of Health and Welfare (Sweden). Sweden National Patient Register 2013
Hemorrhagic Stroke	National Board of Health and Welfare (Sweden). Sweden National Patient Register 2014
Hemorrhagic Stroke	Joint Health Surveys Unit of Social and Community Planning Research and University College London, Scottish Health Survey, 1998 [computer file]. Colchester, Essex: UK Data Archive [distributor], July 2001. SN: 4379 , http://dx.doi.org/10.5255/UKDA-SN-4379-1
Hemorrhagic Stroke	Joint Health Surveys Unit, University College London and Medical Research Council. Social and Public Health Sciences Unit, Scottish Health Survey, 2003 [computer file]. Colchester, Essex: UK Data Archive [distributor], February 2006. SN: 5318
Hemorrhagic Stroke	World Health Organization (WHO). Bosnia and Herzegovina World Health Survey 2003. Geneva, Switzerland: World Health Organization (WHO), 2005
Hemorrhagic Stroke	World Health Organization (WHO). Chad World Health Survey 2003. Geneva, Switzerland: World Health Organization (WHO), 2005
Hemorrhagic Stroke	World Health Organization (WHO). Ireland World Health Survey 2003. Geneva, Switzerland: World Health Organization (WHO), 2005
Hemorrhagic Stroke	World Health Organization (WHO). Nepal World Health Survey 2003. Geneva, Switzerland: World Health Organization (WHO), 2005
Hemorrhagic Stroke	World Health Organization (WHO). Slovakia World Health Survey 2003. Geneva, Switzerland: World Health Organization (WHO), 2005
Hemorrhagic Stroke	World Health Organization (WHO). Tunisia World Health Survey 2003. Geneva, Switzerland: World Health Organization (WHO), 2005
Hemorrhagic Stroke	Centers for Disease Control and Prevention (CDC), Ministry of Health (Jordan), World Health Organization (WHO). Jordan STEPS Noncommunicable Disease Risk Factors Survey 2007
Hemorrhagic Stroke	Srpska), Federal Office of Statistics (Bosnia and Herzegovina), Swedish International Development Agency (SIDA), UK Department for International Development (DFID), United Nations Development Programme (UNDP), European Commission (EC), Government of Japan, World Bank (WB). Bosnia and Herzegovina Living Standards
Hemorrhagic Stroke	Srpska), Federal Office of Statistics (Bosnia and Herzegovina), Independent Bureau for Humanitarian Issues (IBHI), Birks Sinclair and Associates, LTD, Institute for Social and Economic Research, University of Essex. Bosnia and Herzegovina Living Standards Measurement Survey 2002. Washington, DC, United States: World Bank (WB)
Hemorrhagic Stroke	Federal Office of Statistics (Federation of Bosnia and Herzegovina), Independent Bureau for Humanitarian Issues (IBHI), Institute for Social and Economic Research, University of Essex, Institute of Statistics (Republic of Srpska). Bosnia and Herzegovina Living Standards Measurement Survey 2004-2005
Hemorrhagic Stroke	Public Health Authority of the Slovak Republic, World Health Organization (WHO). Slovakia WHO Multi-country Survey Study on Health and Health System Responsiveness 2000
Hemorrhagic Stroke	University of Ibadan (Nigeria), World Health Organization (WHO). Nigeria WHO Multi-country Survey Study on Health and Health System Responsiveness 2000-2001
Hemorrhagic Stroke	Scottish Centre for Social Research and University College London. Department of Epidemiology and Public Health, Scottish Health Survey, 2008 [computer file]. 2nd Edition. Colchester, Essex: UK Data Archive [distributor], April 2013. SN: 6383, http://dx.doi.org/10.5255/UKDA-SN-6383-2
Hemorrhagic Stroke	Scottish Centre for Social Research and University College London. Department of Epidemiology and Public Health, Scottish Health Survey, 2009 [computer file]. 4th Edition. Colchester, Essex: UK Data Archive [distributor], November 2011. SN: 6713, http://dx.doi.org/10.5255/UKDA-SN-6713-2
Hemorrhagic Stroke	ScotCen Social Research and University College London. Department of Epidemiology and Public Health, Scottish Health Survey, 2010 [computer file]. Colchester, Essex: UK Data Archive [distributor], April 2012. SN: 6987, http://dx.doi.org/10.5255/UKDA-SN-6987-1
Hemorrhagic Stroke	and Public Health and University of Glasgow. MRC/CSO Social and Public Health Sciences Unit, Scottish Health Survey, 2011 [computer file]. 2nd Edition. Colchester, Essex: UK Data Archive [distributor], August 2013. SN: 7247, http://dx.doi.org/10.5255/UKDA-SN-7247-2
Hemorrhagic Stroke	Longe AC, Osuntokun BO. Prevalence of neurological disorders in Udo, a rural community in southern Nigeria. Trop Geogr Med. 1989; 41(1): 36-40
Hemorrhagic Stroke	Mrabet A, Attia-Romdhane N, Ben Hamida M, Gharbi N, Le Noan H, Hentati R, Ben Mansour J, Srairi I. Epidemiologic aspects of cerebrovascular accidents in Tunisia. Rev Neurol (Paris). 1990; 146(4): 297-301
Hemorrhagic Stroke	Danesi M, Okubadejo N, Ojini F. Prevalence of stroke in an urban, mixed-income community in Lagos, Nigeria. Neuroepidemiology. 2007; 28(4): 216-23
Hemorrhagic Stroke	Flynn RWV, MacDonald TM, Murray GD, Ferguson C, Shah K, Doney ASF. The Tayside Stroke Cohort: exploiting advanced regional medical informatics to create a region-wide database for studying the pharmacoepidemiology of stroke. Pharmacoepidemiol Drug Saf. 2010; 19(7): 737-44
Hemorrhagic Stroke	Maheswaran R, Pearson T, Smeeton NC, Beevers SD, Campbell MJ, Wolfe CD. Outdoor air pollution and incidence of ischemic and hemorrhagic stroke: a small-area level ecological study. Stroke. 2012; 43(1): 22-7
Hemorrhagic Stroke	Damasceno A, Gomes J, Azevedo A, Carrilho C, Lobo V, Lopes H, Madede T, Pravinrai P, Silva-Matos C, Jalla S, Stewart S, Lunet N. An Epidemiological Study of Stroke Hospitalizations in Maputo, Mozambique A High Burden of Disease in a Resource-Poor Country. Stroke. 2010; 41(11): 2463-9
Hemorrhagic Stroke	National Bureau of Statistics (Nigeria). Nigeria Living Standards Survey 2008-2010. Abuja, Nigeria: National Bureau of Statistics (Nigeria)
Hemorrhagic Stroke	Trinity College Dublin. Ireland Longitudinal Study on Ageing 2012-2013. Dublin, Ireland: Irish Social Science Data Archive, University College Dublin
Hemorrhagic Stroke	ScotCen Social Research, Scottish Health Survey, 2013 [computer file]. Colchester, Essex: UK Data Archive [distributor], December 2014. SN: 7594, http://dx.doi.org/10.5255/UKDA-SN-7594-1
Hemorrhagic Stroke	Department of Epidemiology and Public Health, University College London, Scottish Centre for Social Research (ScotCen), University of Glasgow. United Kingdom - Scottish Health Survey 2012 - Scottish Government
Hemorrhagic Stroke	LINK Institute for Market and Social Research (Switzerland), World Health Organization (WHO). Switzerland WHO Multi-country Survey Study on Health and Health System Responsiveness 2000-2001. Geneva, Switzerland: World Health Organization (WHO)
Hemorrhagic Stroke	International Research Associates (INRA) Europe, World Health Organization (WHO). Ireland WHO Multi-country Survey Study on Health and Health System Responsiveness 2000-2001. Geneva, Switzerland: World Health Organization (WHO)
Hemorrhagic Stroke	Gallup Europe, World Health Organization (WHO). Jordan WHO Multi-country Survey Study on Health and Health System Responsiveness 2000-2001. Geneva, Switzerland: World Health Organization (WHO)
Hemorrhagic Stroke	Börsch-Supan, A. (2015). Survey of Health, Ageing and Retirement in Europe (SHARE) Wave 5. Release version: 1.0.0. SHARE-ERIC. Data set. DOI: 10.6103/SHARE.w5.100

Hemorrhagic Stroke	Concluzia-Prim Center for Survey Methodology (Moldova), Institute for Advanced Studies (Austria), London School of Hygiene and Tropical Medicine, SIAR Research and Consulting (Azerbaijan), University of Aberdeen. Azerbaijan Health in Times of Transition Household Survey 2010
Hemorrhagic Stroke	ScotCen Social Research. (2015). Scottish Health Survey, 2014. [data collection]. UK Data Service. SN: 7851, http://dx.doi.org/10.5255/UKDA-SN-7851-1
Hemorrhagic Stroke	Counsell C, Dennis M, McDowall M. Predicting functional outcome in acute stroke: comparison of a simple six variable model with other predictive systems and informal clinical prediction. J Neurol Neurosurg Psychiatry. 2004; 75(3): 401–5
Hemorrhagic Stroke	Abubakar SA, Okubadejo NU, Ojo OO, Oladipo O, Ojini FI, Danesi MA. Relationship between admission serum C-reactive protein and short term outcome following acute ischaemic stroke at a tertiary health institution in Nigeria. Niger J Clin Pract. 2013; 16(3): 320–4
Hemorrhagic Stroke	Department of Health and Children (Ireland), Economic and Social Research Institute (ESRI) (Ireland). Ireland Hospital Inpatient Enquiry 2000
Hemorrhagic Stroke	Department of Health (Ireland), Economic and Social Research Institute (ESRI) (Ireland). Ireland Hospital Inpatient Enquiry 1980
Hemorrhagic Stroke	Department of Health (Ireland), Economic and Social Research Institute (ESRI) (Ireland). Ireland Hospital Inpatient Enquiry 1981
Hemorrhagic Stroke	Department of Health (Ireland), Economic and Social Research Institute (ESRI) (Ireland). Ireland Hospital Inpatient Enquiry 1982
Hemorrhagic Stroke	Department of Health (Ireland), Economic and Social Research Institute (ESRI) (Ireland). Ireland Hospital Inpatient Enquiry 1983
Hemorrhagic Stroke	Department of Health (Ireland), Economic and Social Research Institute (ESRI) (Ireland). Ireland Hospital Inpatient Enquiry 1984
Hemorrhagic Stroke	Department of Health (Ireland), Economic and Social Research Institute (ESRI) (Ireland). Ireland Hospital Inpatient Enquiry 1985
Hemorrhagic Stroke	Department of Health (Ireland), Economic and Social Research Institute (ESRI) (Ireland). Ireland Hospital Inpatient Enquiry 1986
Hemorrhagic Stroke	Department of Health (Ireland), Economic and Social Research Institute (ESRI) (Ireland). Ireland Hospital Inpatient Enquiry 1987
Hemorrhagic Stroke	Department of Health (Ireland), Economic and Social Research Institute (ESRI) (Ireland). Ireland Hospital Inpatient Enquiry 1988
Hemorrhagic Stroke	Department of Health (Ireland), Economic and Social Research Institute (ESRI) (Ireland). Ireland Hospital Inpatient Enquiry 1989
Hemorrhagic Stroke	Department of Health (Ireland), Economic and Social Research Institute (ESRI) (Ireland). Ireland Hospital Inpatient Enquiry 1990
Hemorrhagic Stroke	Department of Health (Ireland), Economic and Social Research Institute (ESRI) (Ireland). Ireland Hospital Inpatient Enquiry 1991
Hemorrhagic Stroke	Department of Health (Ireland), Economic and Social Research Institute (ESRI) (Ireland). Ireland Hospital Inpatient Enquiry 1992
Hemorrhagic Stroke	Department of Health (Ireland), Economic and Social Research Institute (ESRI) (Ireland). Ireland Hospital Inpatient Enquiry 1993
Hemorrhagic Stroke	Department of Health (Ireland), Economic and Social Research Institute (ESRI) (Ireland). Ireland Hospital Inpatient Enquiry 1994
Hemorrhagic Stroke	Department of Health (Ireland), Economic and Social Research Institute (ESRI) (Ireland). Ireland Hospital Inpatient Enquiry 1995
Hemorrhagic Stroke	Department of Health (Ireland), Economic and Social Research Institute (ESRI) (Ireland). Ireland Hospital Inpatient Enquiry 1996
Hemorrhagic Stroke	Department of Health and Children (Ireland), Economic and Social Research Institute (ESRI) (Ireland). Ireland Hospital Inpatient Enquiry 1997
Hemorrhagic Stroke	Department of Health and Children (Ireland), Economic and Social Research Institute (ESRI) (Ireland). Ireland Hospital Inpatient Enquiry 1998
Hemorrhagic Stroke	Department of Health and Children (Ireland), Economic and Social Research Institute (ESRI) (Ireland). Ireland Hospital Inpatient Enquiry 1999
Hemorrhagic Stroke	Economic and Social Research Institute (ESRI) (Ireland), Health Service Executive (HSE) (Ireland). Ireland Hospital Inpatient Enquiry 2013
Hemorrhagic Stroke	Economic and Social Research Institute (ESRI) (Ireland), Health Service Executive (HSE) (Ireland). Ireland Hospital Inpatient Enquiry 2014
Hemorrhagic Stroke	Federal Statistical Office (Switzerland). Switzerland Medical Statistics of Hospitals 1997
Hemorrhagic Stroke	Federal Statistical Office (Switzerland). Switzerland Medical Statistics of Hospitals 1998
Hemorrhagic Stroke	Federal Statistical Office (Switzerland). Switzerland Medical Statistics of Hospitals 2013
Hemorrhagic Stroke	Federal Statistical Office (Switzerland). Switzerland Medical Statistics of Hospitals 2014
Hemorrhagic Stroke	National Statistical Institute of Bulgaria. Bulgaria Living Standards Measurement Survey 2003. Washington DC, United States: World Bank
Hemorrhagic Stroke	Administrative Department of Science, Technology, and Innovation (Colombia), Center for Development Projects, Pontifical Xavierian University, Ministry of Social Protection (Colombia), Specialized Information Systems. Colombia National Health Survey 2007-2008
Hemorrhagic Stroke	Planning Institute of Jamaica, Statistical Institute of Jamaica. Jamaica Survey of Living Conditions 1988. Kingston, Jamaica: Planning Institute of Jamaica
Hemorrhagic Stroke	Statistical Institute of Jamaica. Jamaica Survey of Living Conditions 1990. Washington DC, United States: World Bank
Hemorrhagic Stroke	Planning Institute of Jamaica, Statistical Institute of Jamaica. Jamaica Survey of Living Conditions 1992
Hemorrhagic Stroke	Planning Institute of Jamaica, Statistical Institute of Jamaica. Jamaica Survey of Living Conditions 1991
Hemorrhagic Stroke	World Health Organization (WHO). Austria World Health Survey 2003. Geneva, Switzerland: World Health Organization (WHO), 2005
Hemorrhagic Stroke	Center for Scientific and Technological Information, Oswaldo Cruz Foundation and World Health Organization (WHO). Brazil World Health Survey 2003. Geneva, Switzerland: World Health Organization (WHO), 2005
Hemorrhagic Stroke	World Health Organization (WHO). Côte d'Ivoire World Health Survey 2003. Geneva, Switzerland: World Health Organization (WHO), 2005
Hemorrhagic Stroke	World Health Organization (WHO). Georgia World Health Survey 2003. Geneva, Switzerland: World Health Organization (WHO), 2005
Hemorrhagic Stroke	World Health Organization (WHO). Israel World Health Survey 2003. Geneva, Switzerland: World Health Organization (WHO), 2005
Hemorrhagic Stroke	World Health Organization (WHO). Myanmar World Health Survey 2003. Geneva, Switzerland: World Health Organization (WHO), 2005
Hemorrhagic Stroke	World Health Organization (WHO). Namibia World Health Survey 2003. Geneva, Switzerland: World Health Organization (WHO), 2005
Hemorrhagic Stroke	World Health Organization (WHO). Pakistan World Health Survey 2003-2004. Geneva, Switzerland: World Health Organization (WHO), 2005
Hemorrhagic Stroke	World Health Organization (WHO). Slovenia World Health Survey 2003. Geneva, Switzerland: World Health Organization (WHO), 2005
Hemorrhagic Stroke	World Health Organization (WHO). United Kingdom World Health Survey 2004. Geneva, Switzerland: World Health Organization (WHO), 2005
Hemorrhagic Stroke	Office of Population Censuses and Surveys. Social Survey Division, Health Survey for England, 1993 [Computer file]. Colchester, Essex: UK Data Archive [distributor], April 1995. SN: 3316, http://dx.doi.org/10.5255/UKDA-SN-3316-1
Hemorrhagic Stroke	Joint Health Surveys Unit of Social and Community Planning Research and University College London, Health Survey for England, 1994 [computer file]. 4th ed. Colchester, Essex: UK Data Archive [distributor], 26 March 2001. SN: 3640
Hemorrhagic Stroke	National Centre for Social Research, University College London Department of Epidemiology and Public Health, Health Survey for England, 1998 [computer file]. 4th ed. Colchester, Essex: UK Data Archive [distributor], 30 November 2002. SN: 4150
Hemorrhagic Stroke	National Centre for Social Research, University College London Department of Epidemiology and Public Health, Health Survey for England, 2000 [computer file]. Colchester, Essex: UK Data Archive [distributor], 23 April 2002. SN: 4487
Hemorrhagic Stroke	National Centre for Social Research and University College London. Department of Epidemiology and Public Health, Health Survey for England, 2003 [computer file]. Colchester, Essex: UK Data Archive [distributor], March 2005. SN: 5098
Hemorrhagic Stroke	National Centre for Social Research and University College London. Department of Epidemiology and Public Health, Health Survey for England, 2005 [computer file]. Colchester, Essex: UK Data Archive [distributor], July 2007. SN: 5675
Hemorrhagic Stroke	National Centre for Social Research and University College London. Department of Epidemiology and Public Health, Health Survey for England, 2006 [computer file]. 4th Edition. Colchester, Essex: UK Data Archive [distributor], July 2011. SN: 5809, http://dx.doi.org/10.5255/UKDA-SN-5809-1
Hemorrhagic Stroke	University of Wisconsin-Madison, Inter-University Consortium for Political and Social Research (ICPSR), University of São Paulo. Brazil - São Paulo Survey on Health, Well-Being, and Aging in Latin America and the Caribbean 1999-2000. Ann Arbor, United States: Inter-University Consortium for Political and Social Research (ICPSR)
Hemorrhagic Stroke	Planning Institute of Jamaica, Statistical Institute of Jamaica. Jamaica Survey of Living Conditions 1993
Hemorrhagic Stroke	Planning Institute of Jamaica, Statistical Institute of Jamaica. Jamaica Survey of Living Conditions 1994
Hemorrhagic Stroke	Planning Institute of Jamaica, Statistical Institute of Jamaica. Jamaica Survey of Living Conditions 1995
Hemorrhagic Stroke	Planning Institute of Jamaica, Statistical Institute of Jamaica. Jamaica Survey of Living Conditions 1996
Hemorrhagic Stroke	Planning Institute of Jamaica, Statistical Institute of Jamaica. Jamaica Survey of Living Conditions 1997
Hemorrhagic Stroke	Planning Institute of Jamaica, Statistical Institute of Jamaica. Jamaica Survey of Living Conditions 1999

Hemorrhagic Stroke	Planning Institute of Jamaica, Statistical Institute of Jamaica. Jamaica Survey of Living Conditions 2000
Hemorrhagic Stroke	TNS BBSS, World Bank. Bulgaria Living Standards Measurement Survey 2001. Washington DC, United States: World Bank
Hemorrhagic Stroke	National Administrative Department of Statistics (Colombia). Colombia National Quality of Life Survey 1997. Bogotá, Colombia: National Administrative Department of Statistics (Colombia)
Hemorrhagic Stroke	National Administrative Department of Statistics (Colombia). Colombia National Quality of Life Survey 2008. Bogotá, Colombia: National Administrative Department of Statistics (Colombia)
Hemorrhagic Stroke	National Administrative Department of Statistics (Colombia). Colombia National Quality of Life Survey 2010. Bogotá, Colombia: National Administrative Department of Statistics (Colombia), 2012
Hemorrhagic Stroke	Pontificia Universidad Javeriana (Colombia), World Health Organization (WHO). Colombia WHO Multi-country Survey Study on Health and Health System Responsiveness 2000-2001. Geneva, Switzerland: World Health Organization (WHO)
Hemorrhagic Stroke	AMATEM (Turkey), Plaza Ltd. Research, World Health Organization (WHO). Turkey WHO Multi-country Survey Study on Health and Health System Responsiveness 2000-2001. Geneva, Switzerland: World Health Organization (WHO)
Hemorrhagic Stroke	Institute for Polling and Marketing (Georgia), World Health Organization (WHO). Georgia WHO Multi-country Survey Study on Health and Health System Responsiveness 2000-2001
Hemorrhagic Stroke	Turkish Statistical Institute. Turkey Health Interview Survey 2010. Ankara, Turkey: Turkish Statistical Institute
Hemorrhagic Stroke	NatCen Social Research and University College London. Department of Epidemiology and Public Health, Health Survey for England, 2011 [computer file]. Colchester, Essex: UK Data Archive [distributor], April 2013. SN: 7260, http://dx.doi.org/10.5255/UKDA-SN-7260-1
Hemorrhagic Stroke	Minelli C, Fu Fen L, Camara Minelli DP. Stroke Incidence, Prognosis, 30-Day, and 1-Year Case Fatality Rates in Matão, Brazil. Stroke. 2007; 38(11): 2906-11
Hemorrhagic Stroke	Cabral NL, Gonçalves ARR, Longo AL, Moro CHC, Costa G, Amaral CH, Fonseca L a M, Eluf-Neto J. Incidence of stroke subtypes, prognosis and prevalence of risk factors in Joinville, Brazil: a 2 year community based study. J Neurol Neurosurg Psychiatr. 2009; 80(7): 755-61
Hemorrhagic Stroke	Abdul-Ghaffar NU, el-Sonbaty MR, el-Din Abdul-Baky MS, Marafie AA, al-Said AM. Stroke in Kuwait: a three-year prospective study. Neuroepidemiology. 1997; 16(1): 40-7
Hemorrhagic Stroke	Börü UT, Oztürk E, Ta?demir M, Sur H. Living alone following first-ever stroke: a prospective study in Turkey identifying the risk factors and evaluating their effects. N Z Med J. 2007; 120(1255): U2559
Hemorrhagic Stroke	Du X, Sourbutts J, Cruickshank K, Alison Summers, Roberts N, Walton E, Holmes S. A Community Based Stroke Register in a High Risk Area for Stroke in North West England. J Epidemiol Community Health. 1997; 51(5): 472-8
Hemorrhagic Stroke	Kumral E, Ozkaya B, Sagduyu A, Sirin H, Vardarli E, Pehlivan M. The Ege Stroke Registry: A Hospital-Based Study in the Aegean Region, Izmir, Turkey. Cerebrovasc Dis. 1998; 8(5): 278-88
Hemorrhagic Stroke	Tsiskaridze A, Djibuti M, van Melle G, Lomidze G, Apridonidze S, Gauarashvili I, Piechowski-Józwiak B, Shakarishvili R, Bogousslavsky J. Stroke Incidence and 30-Day Case-Fatality in a Suburb of Tbilisi: Results of the First Prospective Population-Based Study in Georgia. Stroke. 2004; 35(11): 2523-8
Hemorrhagic Stroke	Statistics Austria, World Health Organization (WHO). Austria WHO Multi-country Survey Study on Health and Health System Responsiveness 2000-2001. Geneva, Switzerland: World Health Organization (WHO)
Hemorrhagic Stroke	National Assembly for Wales, Welsh Health Survey, 1998 [computer file]. 2nd Edition. Colchester, Essex: UK Data Archive [distributor], February 2011. SN: 4176 , http://dx.doi.org/10.5255/UKDA-SN-4176-1
Hemorrhagic Stroke	National Centre for Social Research, Beaufort Research Limited and University College London. Department of Epidemiology and Public Health, Welsh Health Survey, 2003-2004 [computer file]. 2nd Edition. Colchester, Essex: UK Data Archive [distributor], February 2011. SN: 5692 , http://dx.doi.org/10.5255/UKDA-SN-5692-1
Hemorrhagic Stroke	National Centre for Social Research, Welsh Health Survey, 2005-2006 [computer file]. 2nd Edition. Colchester, Essex: UK Data Archive [distributor], February 2011. SN: 5750 , http://dx.doi.org/10.5255/UKDA-SN-5750-1
Hemorrhagic Stroke	National Centre for Social Research, Welsh Health Survey, 2007 [computer file]. 2nd Edition. Colchester, Essex: UK Data Archive [distributor], February 2011. SN: 6052, http://dx.doi.org/10.5255/UKDA-SN-6052-1
Hemorrhagic Stroke	National Centre for Social Research, Welsh Health Survey, 2008 [computer file]. 2nd Edition. Colchester, Essex: UK Data Archive [distributor], February 2011. SN: 6372
Hemorrhagic Stroke	National Centre for Social Research, Welsh Health Survey, 2009 [computer file]. 2nd Edition. Colchester, Essex: UK Data Archive [distributor], February 2011. SN: 6589, http://dx.doi.org/10.5255/UKDA-SN-6589-1
Hemorrhagic Stroke	National Centre for Social Research, Welsh Health Survey, 2010 [computer file]. Colchester, Essex: UK Data Archive [distributor], November 2011. SN: 6895, http://dx.doi.org/10.5255/UKDA-SN-6895-1
Hemorrhagic Stroke	NatCen Social Research, Welsh Health Survey, 2011 [computer file]. Colchester, Essex: UK Data Archive [distributor], January 2013. SN: 7188, http://dx.doi.org/10.5255/UKDA-SN-7188-1
Hemorrhagic Stroke	NatCen Social Research, Welsh Health Survey, 2012 [computer file]. Colchester, Essex: UK Data Archive [distributor], February 2014. SN: 7459, http://dx.doi.org/10.5255/UKDA-SN-7459-1
Hemorrhagic Stroke	Federal Ministry of Health (Austria), Statistics Austria. Austria Hospital Inpatient Discharges 1989. Vienna, Austria: Statistics Austria
Hemorrhagic Stroke	National Institute of Statistics of Rwanda. Rwanda Integrated Household Living Conditions Survey 2010-2011. Kigali, Rwanda: National Institute of Statistics of Rwanda
Hemorrhagic Stroke	Health Institute (São Paulo, Brazil), State University of Campinas, São Paulo Municipal Health Department, São Paulo State University, University of São Paulo. Brazil - São Paulo Health Survey 2008-2009
Hemorrhagic Stroke	NatCen Social Research, Welsh Health Survey, 2013 [computer file]. Colchester, Essex: UK Data Archive [distributor], January 2015. SN: 7632, http://dx.doi.org/10.5255/UKDA-SN-7632-1
Hemorrhagic Stroke	Brazilian Institute of Geography and Statistics (IBGE), Ministry of Health (Brazil), Ministry of Planning, Budget, and Management (Brazil). Brazil National Health Survey 2013. Rio de Janeiro, Brazil: Brazilian Institute of Geography and Statistics (IBGE)
Hemorrhagic Stroke	Federal Ministry of Health (Austria), Statistics Austria. Austria Hospital Inpatient Discharges 2013. Vienna, Austria: Statistics Austria
Hemorrhagic Stroke	International Research Associates (INRA) Europe, World Health Organization (WHO). Bulgaria WHO Multi-country Survey Study on Health and Health System Responsiveness 2000-2001. Geneva, Switzerland: World Health Organization (WHO)
Hemorrhagic Stroke	National Centre for Social Research (NatCen), World Health Organization (WHO). United Kingdom WHO Multi-country Survey Study on Health and Health System Responsiveness 2000-2001. Geneva, Switzerland: World Health Organization (WHO)
Hemorrhagic Stroke	Börsch-Supan, A. (2015). Survey of Health, Ageing and Retirement in Europe (SHARE) Wave 5. Release version: 1.0.0. SHARE-ERIC. Data set. DOI: 10.6103/SHARE.w5.100
Hemorrhagic Stroke	Börsch-Supan, A. (2015). Survey of Health, Ageing and Retirement in Europe (SHARE) Wave 5. Release version: 1.0.0. SHARE-ERIC. Data set. DOI: 10.6103/SHARE.w5.100
Hemorrhagic Stroke	Börsch-Supan, A. (2015). Survey of Health, Ageing and Retirement in Europe (SHARE) Wave 5. Release version: 1.0.0. SHARE-ERIC. Data set. DOI: 10.6103/SHARE.w5.100
Hemorrhagic Stroke	National Center for Disease Control and Public Health (Georgia). Georgia Hospital Data 2014
Hemorrhagic Stroke	Concluzia-Prim Center for Survey Methodology (Moldova), Georgia Opinion Research Business International (GORBI), Institute for Advanced Studies (Austria), London School of Hygiene and Tropical Medicine, University of Aberdeen. Georgia Health in Times of Transition Household Survey 2010

Hemorrhagic Stroke	Federal Ministry of Health (Austria), Statistics Austria. Austria Hospital Inpatient Discharges 1989-1992
Hemorrhagic Stroke	Federal Ministry of Health (Austria), Statistics Austria. Austria Hospital Inpatient Discharges 1993-1997
Hemorrhagic Stroke	Federal Ministry of Health (Austria), Statistics Austria. Austria Hospital Inpatient Discharges 1998-2002
Hemorrhagic Stroke	Federal Ministry of Health (Austria), Statistics Austria. Austria Hospital Inpatient Discharges 2003-2007
Hemorrhagic Stroke	Federal Ministry of Health (Austria), Statistics Austria. Austria Hospital Inpatient Discharges 2008-2012
Hemorrhagic Stroke	Ministry of Labor and Social Policy (Bulgaria), National Statistical Institute of Bulgaria, TNS Gallup, World Bank. Bulgaria Multitopic Household Survey 2007. Washington DC, United States: World Bank
Hemorrhagic Stroke	Federal Ministry of Health (Austria), Statistics Austria. Austria Hospital Inpatient Discharges 2014. Vienna, Austria: Statistics Austria
Hemorrhagic Stroke	Rivero-Arias O, Ouellet M, Gray A, Wolstenholme J, Rothwell PM, Luengo-Fernandez R. Mapping the modified Rankin scale (mRS) measurement into the generic EuroQol (EQ-5D) health outcome. Med Decis Mak. 2010; 30(3): 341–54
Hemorrhagic Stroke	Luengo-Fernandez R, Paul NLM, Gray AM, Pendlebury ST, Bull LM, Welch SJV, Cuthbertson FC, Rothwell PM, Oxford Vascular Study. Population-based study of disability and institutionalization after transient ischemic attack and stroke: 10-year results of the Oxford Vascular Study. Stroke. 2013; 44(10): 2854–61
Hemorrhagic Stroke	Federal Ministry of Health (Austria), Statistics Austria. Austria Hospital Inpatient Discharges 2013-2014
Hemorrhagic Stroke	Fernandes TG, Goulart AC, Santos-Junior WR, Alencar AP, Benseñor IM, Lotufo PA. Educational levels and the functional dependence of ischemic stroke survivors. Cad Saude Publica. 2012; 28(8): 1581–90
Hemorrhagic Stroke	National Institute of Public Health (Slovenia). Slovenia National Hospital Health Care Statistics Database 2004
Hemorrhagic Stroke	National Institute of Public Health (Slovenia). Slovenia National Hospital Health Care Statistics Database 2005
Hemorrhagic Stroke	National Institute of Public Health (Slovenia). Slovenia National Hospital Health Care Statistics Database 2006
Hemorrhagic Stroke	National Institute of Public Health (Slovenia). Slovenia National Hospital Health Care Statistics Database 2007
Hemorrhagic Stroke	National Institute of Public Health (Slovenia). Slovenia National Hospital Health Care Statistics Database 2008
Hemorrhagic Stroke	General Directorate of Curative Services, Ministry of Health (Turkey). Turkey Hospital Inpatient Discharges 2007
Hemorrhagic Stroke	General Directorate of Curative Services, Ministry of Health (Turkey). Turkey Hospital Inpatient Discharges 2008
Hemorrhagic Stroke	General Directorate of Curative Services, Ministry of Health (Turkey). Turkey Hospital Inpatient Discharges 2009
Hemorrhagic Stroke	General Directorate of Curative Services, Ministry of Health (Turkey). Turkey Hospital Inpatient Discharges 2010
Hemorrhagic Stroke	General Directorate of Curative Services, Ministry of Health (Turkey). Turkey Hospital Inpatient Discharges 2011
Hemorrhagic Stroke	National Center for Disease Control and Public Health (Georgia). Georgia Inpatient Care Discharges per 100 1991
Hemorrhagic Stroke	National Center for Disease Control and Public Health (Georgia). Georgia Inpatient Care Discharges per 100 1992
Hemorrhagic Stroke	National Center for Disease Control and Public Health (Georgia). Georgia Inpatient Care Discharges per 100 1993
Hemorrhagic Stroke	National Center for Disease Control and Public Health (Georgia). Georgia Inpatient Care Discharges per 100 1994
Hemorrhagic Stroke	National Center for Disease Control and Public Health (Georgia). Georgia Inpatient Care Discharges per 100 1995
Hemorrhagic Stroke	National Center for Disease Control and Public Health (Georgia). Georgia Inpatient Care Discharges per 100 1996
Hemorrhagic Stroke	National Center for Disease Control and Public Health (Georgia). Georgia Inpatient Care Discharges per 100 1997
Hemorrhagic Stroke	National Center for Disease Control and Public Health (Georgia). Georgia Inpatient Care Discharges per 100 1998
Hemorrhagic Stroke	National Center for Disease Control and Public Health (Georgia). Georgia Inpatient Care Discharges per 100 1999
Hemorrhagic Stroke	National Center for Disease Control and Public Health (Georgia). Georgia Inpatient Care Discharges per 100 2000
Hemorrhagic Stroke	National Center for Disease Control and Public Health (Georgia). Georgia Inpatient Care Discharges per 100 2001
Hemorrhagic Stroke	National Center for Disease Control and Public Health (Georgia). Georgia Inpatient Care Discharges per 100 2002
Hemorrhagic Stroke	National Center for Disease Control and Public Health (Georgia). Georgia Inpatient Care Discharges per 100 2003
Hemorrhagic Stroke	National Center for Disease Control and Public Health (Georgia). Georgia Inpatient Care Discharges per 100 2004
Hemorrhagic Stroke	National Center for Disease Control and Public Health (Georgia). Georgia Inpatient Care Discharges per 100 2005
Hemorrhagic Stroke	National Center for Disease Control and Public Health (Georgia). Georgia Inpatient Care Discharges per 100 2006
Hemorrhagic Stroke	National Center for Disease Control and Public Health (Georgia). Georgia Inpatient Care Discharges per 100 2007
Hemorrhagic Stroke	National Center for Disease Control and Public Health (Georgia). Georgia Inpatient Care Discharges per 100 2008
Hemorrhagic Stroke	National Center for Disease Control and Public Health (Georgia). Georgia Inpatient Care Discharges per 100 2009
Hemorrhagic Stroke	National Center for Disease Control and Public Health (Georgia). Georgia Inpatient Care Discharges per 100 2010
Hemorrhagic Stroke	National Center for Disease Control and Public Health (Georgia). Georgia Inpatient Care Discharges per 100 2011
Hemorrhagic Stroke	National Center for Disease Control and Public Health (Georgia). Georgia Inpatient Care Discharges per 100 2012
Hemorrhagic Stroke	National Center for Disease Control and Public Health (Georgia). Georgia Inpatient Care Discharges per 100 2013
Hemorrhagic Stroke	National Center for Disease Control and Public Health (Georgia). Georgia Inpatient Care Discharges per 100 2014
Hemorrhagic Stroke	Ministry of Health (Israel). Israel National Hospital Discharge Database 2011
Hemorrhagic Stroke	Ministry of Health (Israel). Israel National Hospital Discharge Database 2012
Hemorrhagic Stroke	Ministry of Health (Israel). Israel National Hospital Discharge Database 2013
Hemorrhagic Stroke	Ministry of Health (Israel). Israel National Hospital Discharge Database 2014
Hemorrhagic Stroke	National Center for Disease Control and Public Health (Georgia). Georgia Inpatient Care Discharges per 100 1980
Hemorrhagic Stroke	National Center for Disease Control and Public Health (Georgia). Georgia Inpatient Care Discharges per 100 1985
Hemorrhagic Stroke	National Center for Disease Control and Public Health (Georgia). Georgia Inpatient Care Discharges per 100 1986
Hemorrhagic Stroke	National Center for Disease Control and Public Health (Georgia). Georgia Inpatient Care Discharges per 100 1987
Hemorrhagic Stroke	National Center for Disease Control and Public Health (Georgia). Georgia Inpatient Care Discharges per 100 1988
Hemorrhagic Stroke	National Center for Disease Control and Public Health (Georgia). Georgia Inpatient Care Discharges per 100 1989
Hemorrhagic Stroke	National Center for Disease Control and Public Health (Georgia). Georgia Inpatient Care Discharges per 100 1990
Hemorrhagic Stroke	General Directorate of Curative Services, Ministry of Health (Turkey). Turkey Inpatient Care Discharges per 100 1980
Hemorrhagic Stroke	General Directorate of Curative Services, Ministry of Health (Turkey). Turkey Inpatient Care Discharges per 100 1981
Hemorrhagic Stroke	General Directorate of Curative Services, Ministry of Health (Turkey). Turkey Inpatient Care Discharges per 100 1982
Hemorrhagic Stroke	General Directorate of Curative Services, Ministry of Health (Turkey). Turkey Inpatient Care Discharges per 100 1983
Hemorrhagic Stroke	General Directorate of Curative Services, Ministry of Health (Turkey). Turkey Inpatient Care Discharges per 100 1984
Hemorrhagic Stroke	General Directorate of Curative Services, Ministry of Health (Turkey). Turkey Inpatient Care Discharges per 100 1985
Hemorrhagic Stroke	General Directorate of Curative Services, Ministry of Health (Turkey). Turkey Inpatient Care Discharges per 100 1986
Hemorrhagic Stroke	General Directorate of Curative Services, Ministry of Health (Turkey). Turkey Inpatient Care Discharges per 100 1987
Hemorrhagic Stroke	General Directorate of Curative Services, Ministry of Health (Turkey). Turkey Inpatient Care Discharges per 100 1988
Hemorrhagic Stroke	General Directorate of Curative Services, Ministry of Health (Turkey). Turkey Inpatient Care Discharges per 100 1989
Hemorrhagic Stroke	General Directorate of Curative Services, Ministry of Health (Turkey). Turkey Inpatient Care Discharges per 100 1990
Hemorrhagic Stroke	General Directorate of Curative Services, Ministry of Health (Turkey). Turkey Inpatient Care Discharges per 100 1991
Hemorrhagic Stroke	General Directorate of Curative Services, Ministry of Health (Turkey). Turkey Inpatient Care Discharges per 100 1992
Hemorrhagic Stroke	General Directorate of Curative Services, Ministry of Health (Turkey). Turkey Inpatient Care Discharges per 100 1993

Hemorrhagic Stroke	General Directorate of Curative Services, Ministry of Health (Turkey). Turkey Inpatient Care Discharges per 100 1994
Hemorrhagic Stroke	General Directorate of Curative Services, Ministry of Health (Turkey). Turkey Inpatient Care Discharges per 100 1995
Hemorrhagic Stroke	General Directorate of Curative Services, Ministry of Health (Turkey). Turkey Inpatient Care Discharges per 100 1996
Hemorrhagic Stroke	General Directorate of Curative Services, Ministry of Health (Turkey). Turkey Inpatient Care Discharges per 100 1997
Hemorrhagic Stroke	General Directorate of Curative Services, Ministry of Health (Turkey). Turkey Inpatient Care Discharges per 100 1998
Hemorrhagic Stroke	General Directorate of Curative Services, Ministry of Health (Turkey). Turkey Inpatient Care Discharges per 100 1999
Hemorrhagic Stroke	General Directorate of Curative Services, Ministry of Health (Turkey). Turkey Inpatient Care Discharges per 100 2000
Hemorrhagic Stroke	General Directorate of Curative Services, Ministry of Health (Turkey). Turkey Inpatient Care Discharges per 100 2001
Hemorrhagic Stroke	General Directorate of Curative Services, Ministry of Health (Turkey). Turkey Inpatient Care Discharges per 100 2002
Hemorrhagic Stroke	General Directorate of Curative Services, Ministry of Health (Turkey). Turkey Inpatient Care Discharges per 100 2003
Hemorrhagic Stroke	General Directorate of Curative Services, Ministry of Health (Turkey). Turkey Inpatient Care Discharges per 100 2004
Hemorrhagic Stroke	General Directorate of Curative Services, Ministry of Health (Turkey). Turkey Inpatient Care Discharges per 100 2005
Hemorrhagic Stroke	General Directorate of Curative Services, Ministry of Health (Turkey). Turkey Inpatient Care Discharges per 100 2006
Hemorrhagic Stroke	General Directorate of Curative Services, Ministry of Health (Turkey). Turkey Inpatient Care Discharges per 100 2012
Hemorrhagic Stroke	General Directorate of Curative Services, Ministry of Health (Turkey). Turkey Inpatient Care Discharges per 100 2013
Hemorrhagic Stroke	General Directorate of Curative Services, Ministry of Health (Turkey). Turkey Inpatient Care Discharges per 100 2014
Hemorrhagic Stroke	National Institute of Public Health (Slovenia). Slovenia National Hospital Health Care Statistics Database 1980
Hemorrhagic Stroke	National Institute of Public Health (Slovenia). Slovenia National Hospital Health Care Statistics Database 1986
Hemorrhagic Stroke	National Institute of Public Health (Slovenia). Slovenia National Hospital Health Care Statistics Database 1985
Hemorrhagic Stroke	National Institute of Public Health (Slovenia). Slovenia National Hospital Health Care Statistics Database 1987
Hemorrhagic Stroke	National Institute of Public Health (Slovenia). Slovenia National Hospital Health Care Statistics Database 1988
Hemorrhagic Stroke	National Institute of Public Health (Slovenia). Slovenia National Hospital Health Care Statistics Database 1989
Hemorrhagic Stroke	National Institute of Public Health (Slovenia). Slovenia National Hospital Health Care Statistics Database 1990
Hemorrhagic Stroke	National Institute of Public Health (Slovenia). Slovenia National Hospital Health Care Statistics Database 1991
Hemorrhagic Stroke	National Institute of Public Health (Slovenia). Slovenia National Hospital Health Care Statistics Database 1992
Hemorrhagic Stroke	National Institute of Public Health (Slovenia). Slovenia National Hospital Health Care Statistics Database 1993
Hemorrhagic Stroke	National Institute of Public Health (Slovenia). Slovenia National Hospital Health Care Statistics Database 1994
Hemorrhagic Stroke	National Institute of Public Health (Slovenia). Slovenia National Hospital Health Care Statistics Database 1995
Hemorrhagic Stroke	National Institute of Public Health (Slovenia). Slovenia National Hospital Health Care Statistics Database 1996
Hemorrhagic Stroke	National Institute of Public Health (Slovenia). Slovenia National Hospital Health Care Statistics Database 1997
Hemorrhagic Stroke	National Institute of Public Health (Slovenia). Slovenia National Hospital Health Care Statistics Database 1998
Hemorrhagic Stroke	National Institute of Public Health (Slovenia). Slovenia National Hospital Health Care Statistics Database 1999
Hemorrhagic Stroke	National Institute of Public Health (Slovenia). Slovenia National Hospital Health Care Statistics Database 2000
Hemorrhagic Stroke	National Institute of Public Health (Slovenia). Slovenia National Hospital Health Care Statistics Database 2001
Hemorrhagic Stroke	National Institute of Public Health (Slovenia). Slovenia National Hospital Health Care Statistics Database 2002
Hemorrhagic Stroke	National Institute of Public Health (Slovenia). Slovenia National Hospital Health Care Statistics Database 2003
Hemorrhagic Stroke	National Institute of Public Health (Slovenia). Slovenia National Hospital Health Care Statistics Database 2013
Hemorrhagic Stroke	National Institute of Public Health (Slovenia). Slovenia National Hospital Health Care Statistics Database 2014
Hemorrhagic Stroke	Bahamas Department of Statistics, Ministry of Health (The Bahamas). Bahamas Living Conditions Survey 2001. Nassau, The Bahamas: Bahamas Department of Statistics
Hemorrhagic Stroke	Agency of the Republic of Kazakhstan on Statistics, World Bank. Kazakhstan Living Standards Measurement Survey 1996. Washington DC, United States: World Bank
Hemorrhagic Stroke	Central Statistical Service (South Africa). South Africa October Household Survey 1996
Hemorrhagic Stroke	World Health Organization (WHO). Belgium World Health Survey 2002. Geneva, Switzerland: World Health Organization (WHO), 2005
Hemorrhagic Stroke	World Health Organization (WHO). China World Health Survey 2002. Geneva, Switzerland: World Health Organization (WHO), 2005
Hemorrhagic Stroke	World Health Organization (WHO). Comoros World Health Survey 2003. Geneva, Switzerland: World Health Organization (WHO), 2005
Hemorrhagic Stroke	World Health Organization (WHO). Croatia World Health Survey 2003. Geneva, Switzerland: World Health Organization (WHO), 2005
Hemorrhagic Stroke	World Health Organization (WHO). Italy World Health Survey 2003. Geneva, Switzerland: World Health Organization (WHO), 2005
Hemorrhagic Stroke	World Health Organization (WHO). Kazakhstan World Health Survey 2002-2003. Geneva, Switzerland: World Health Organization (WHO), 2005
Hemorrhagic Stroke	World Health Organization (WHO). Paraguay World Health Survey 2002-2003. Geneva, Switzerland: World Health Organization (WHO), 2005
Hemorrhagic Stroke	World Health Organization (WHO). Philippines World Health Survey 2003. Geneva, Switzerland: World Health Organization (WHO), 2005
Hemorrhagic Stroke	World Health Organization (WHO). Senegal World Health Survey 2003. Geneva, Switzerland: World Health Organization (WHO), 2005
Hemorrhagic Stroke	World Health Organization (WHO). United Arab Emirates World Health Survey 2003. Geneva, Switzerland: World Health Organization (WHO), 2005
Hemorrhagic Stroke	Ministry of Health (China), National Center for Chronic and Noncommunicable Disease Control and Prevention (China), World Health Organization (WHO). China WHO Study on Global AGEing and Adult Health 2007-2010
Hemorrhagic Stroke	Carolina Population Center, University of North Carolina at Chapel Hill, Chinese Center for Disease Control and Prevention (CCDC). China Health and Nutrition Survey. Chapel Hill, United States: Carolina Population Center, University of North Carolina at Chapel Hill
Hemorrhagic Stroke	Ministry of Public Health (Lebanon), World Health Organization (WHO). Lebanon WHO Multi-country Survey Study on Health and Health System Responsiveness 2000-2001
Hemorrhagic Stroke	Institute of Social Medicine and Health Policy, Shandong University, Shandong University School of Medicine, World Health Organization (WHO). China WHO Multi-country Survey Study on Health and Health System Responsiveness 2000-2001
Hemorrhagic Stroke	Carolei A, Marini C, Di Napoli M, Di Gianfilippo G, Santalucia P, Baldassarre M, Giorgio De Matteis M, di Orio F. High Stroke Incidence in the Prospective Community-Based L'Aquila Registry (1994-1998): First Year's Results. Stroke. 1997; 28(12): 2500-6
Hemorrhagic Stroke	Di Carlo A, Inzitari D, Galati F, Baldereschi M, Giunta V, Grillo G, Furchì A, Manno V, Naso F, Vecchio A, Consoli D. A Prospective Community-Based Study of Stroke in Southern Italy: The Vibo Valentia Incidence of Stroke Study (VISS). Cerebrovasc Dis. 2003; 16(4): 410-7
Hemorrhagic Stroke	Manobianca G, Zoccolella S, Petruzzellis A, Miccoli A, Logroscino G. The incidence of major stroke subtypes in Southern Italy: a population based study. Eur J Neurol. 2010; 17(9): 1148-55
Hemorrhagic Stroke	Corso G, Bottacchi E, Giardini G, De la Pierre F, Meloni T, Pesenti Campagnoni M, Ponzetti C, Veronese Morosini M. Community-based study of stroke incidence in the Valley of Aosta, Italy. CARe-cerebrovascular Aosta Registry: years 2004-2005. Neuroepidemiology. 2009; 32(3): 186-95
Hemorrhagic Stroke	Lauria G, Gentile M, Fassetta G, Casetta I, Agnoli F, Andreotta G, Barp C, Caneve G, Cavallaro A, Cielo R, Mongillo D, Mosca M, Olivieri P. Incidence and Prognosis of Stroke in the Belluno Province, Italy: First-Year Results of a Community-Based Study. Stroke. 1995; 26(10): 1787-93
Hemorrhagic Stroke	Sacco S, Marini C, Toni D, Olivieri L, Carolei A. Incidence and 10-Year Survival of Intracerebral Hemorrhage in a Population-Based Registry. Stroke. 2009; 40(2): 394-9
Hemorrhagic Stroke	D'Alessandro G, Bottacchi E, Di Giovanni M, Martinazzo C, Sironi L, Lia C, Carenini L, Corso G, Gerbaz V, Polillo C, Pesenti Compagnoni M. Temporal trends of stroke in Valle d'Aosta, Italy. Incidence and 30-day fatality rates. Neurol Sci. 2000; 21(1): 13-8

Hemorrhagic Stroke	Manobianca G, Zoccolella S, Petruzzellis A, Miccoli A, Logroscino G. Low Incidence of Stroke in Southern Italy: A Population-Based Study. Stroke. 2008; 39(11): 2923-8
Hemorrhagic Stroke	Hong Y, Bots ML, Pan X, Hofman A, Grobbee DE, Chen H. Stroke Incidence and Mortality in Rural and Urban Shanghai From 1984 Through 1991: Findings From a Community-Based Registry. Stroke. 1994; 25(6): 1165-9
Hemorrhagic Stroke	Zhao D, Liu J, Wang W, Zeng Z, Cheng J, Liu J, Sun J, Wu Z. Epidemiological Transition of Stroke in China Twenty-One-Year Observational Study From the Sino-MONICA-Beijing Project. Stroke. 2008; 39(6): 1668-74
Hemorrhagic Stroke	Central Statistical Service (South Africa). South Africa October Household Survey 1994
Hemorrhagic Stroke	Musolino R LSP. First-ever stroke incidence and 30-day case fatality in the Sicilian Aeolian archipelago, Italy. Stroke. 2005; 36(12): 2738-41
Hemorrhagic Stroke	Orlandi G, Gelli A, Fanucchi S, Tognoni G, Acerbi G, Murri L. Prevalence of stroke and transient ischaemic attack in the elderly population of an Italian rural community. Eur J Epidemiol. 2003; 18(9): 879-82
Hemorrhagic Stroke	Prencipe M, Ferretti C, Casini AR, Santini M, Giubilei F, Culasso F. Stroke, disability, and dementia: results of a population survey. Stroke. 1997; 28(3): 531-6
Hemorrhagic Stroke	Pikija S, Cvetko D, Malojcic B, Trkanjec Z, Pavlicek I, Lukic A, Kopjar A, Hajduk M, Androvic A, Bilic-Genter M, Trkulja V. A population-based prospective 24-month study of stroke: incidence and 30-day case-fatality rates of first-ever strokes in Croatia. Neuroepidemiology. 2012; 38(3): 164-71
Hemorrhagic Stroke	D'Alessandro G, Gallo F, Vitaliano A, Col PD, Gorraz F, Cristofaro RD, Boaretto G. Prevalence of stroke and stroke-related disability in Valle d'Aosta, Italy. Neurol Sci. 2010; 31(2): 137-41
Hemorrhagic Stroke	Zhao Y, Yao Z, D'Souza W, Zhu C, Chun H, Zhuoga C, Zhang Q, Hu X, Zhou D. An Epidemiological Survey of Stroke in Lhasa, Tibet, China. Stroke. 2010; 41(12): 2739-43
Hemorrhagic Stroke	China Center for Economic Research, Peking University. China Health and Retirement Longitudinal Study Pilot Resurvey 2012. Beijing, China: China Center for Economic Research, Peking University
Hemorrhagic Stroke	Sacco S, Stracci F, Cerone D, Ricci S, Carolei A. Epidemiology of stroke in Italy. Int J Stroke. 2011; 6(3): 219-27
Hemorrhagic Stroke	Human Sciences Research Council, South African Medical Research Council. South Africa National Health and Nutrition Examination Survey 2012
Hemorrhagic Stroke	Central American Population Center, University of Costa Rica. Costa Rica Survey of Family Health Services and Expenses 2008. San José, Costa Rica: Central American Population Center, University of Costa Rica
Hemorrhagic Stroke	Gallup Europe, World Health Organization (WHO). United Arab Emirates WHO Multi-country Survey Study on Health and Health System Responsiveness 2000-2001. Geneva, Switzerland: World Health Organization (WHO)
Hemorrhagic Stroke	International Research Associates (INRA) Europe, World Health Organization (WHO). Belgium WHO Multi-country Survey Study on Health and Health System Responsiveness 2000-2001. Geneva, Switzerland: World Health Organization (WHO)
Hemorrhagic Stroke	Gallup Europe, World Health Organization (WHO). Costa Rica WHO Multi-country Survey Study on Health and Health System Responsiveness 2000-2001. Geneva, Switzerland: World Health Organization (WHO)
Hemorrhagic Stroke	Market, Media, and Public Opinion Research (Croatia), World Health Organization (WHO). Croatia WHO Multi-country Survey Study on Health and Health System Responsiveness 2000-2001. Geneva, Switzerland: World Health Organization (WHO)
Hemorrhagic Stroke	International Research Associates (INRA) Europe, World Health Organization (WHO). Italy WHO Multi-country Survey Study on Health and Health System Responsiveness 2000-2001. Geneva, Switzerland: World Health Organization (WHO)
Hemorrhagic Stroke	Börsch-Supan, A. (2015). Survey of Health, Ageing and Retirement in Europe (SHARE) Wave 5. Release version: 1.0.0. SHARE-ERIC. Data set. DOI: 10.6103/SHARE.w5.100
Hemorrhagic Stroke	Börsch-Supan, A. (2015). Survey of Health, Ageing and Retirement in Europe (SHARE) Wave 5. Release version: 1.0.0. SHARE-ERIC. Data set. DOI: 10.6103/SHARE.w5.100
Hemorrhagic Stroke	Center for Study of Public Opinion (Kazakhstan), Concluzia-Prim Center for Survey Methodology (Moldova), Institute for Advanced Studies (Austria), London School of Hygiene and Tropical Medicine, University of Aberdeen. Kazakhstan Health in Times of Transition Household Survey 2010
Hemorrhagic Stroke	Corso G, Bottacchi E, Giardini G, Di Giovanni M, Meloni T, Pesenti Campagnoni M, Veronese Morosini M. Epidemiology of stroke in northern Italy: the Cerebrovascular Aosta Registry, 2004-2008. Neurol Sci. 2013; 34(7): 1071–81
Hemorrhagic Stroke	Kong F-Y, Tao W-D, Hao Z-L, Liu M. Predictors of one-year disability and death in Chinese hospitalized women after ischemic stroke. Cerebrovasc Dis. 2010; 29(3): 255–62
Hemorrhagic Stroke	Wang W, Jiang B, Sun H, Ru X, Sun D, Wang L, Wang L, Jiang Y, Li Y, Wang Y, Chen Z, Wu S, Zhang Y, Wang D, Wang Y, Feigin VL; NESS-China investigators. Prevalence, Incidence and Mortality of Stroke in China: Results from a Nationwide Population-Based Survey of 480,687 Adults. Circulation. 2017; 135(8): 759-71
Hemorrhagic Stroke	Center for Health Statistics and Information, National Health and Family Planning Commission (China). China National Health Statistical Information Reporting System 2013-2015
Hemorrhagic Stroke	Institute for Maternal and Child Health - IRCCS “Burlo Garofolo”. Italy - Friuli Venezia Giulia Hospital Inpatient Discharges 2010-2012
Hemorrhagic Stroke	Institute for Maternal and Child Health - IRCCS “Burlo Garofolo”. Italy - Friuli Venezia Giulia Hospital Inpatient Discharges 2013-2015
Hemorrhagic Stroke	Ricci S, Celani MG, La Rosa F, Vitali R, Duca E, Ferraguzzi R, Paolotti M, Seppoloni D, Caputo N, Chiurulla C, Scaroni R, Signorini E. SEPIVAC: a Community-based Study of Stroke Incidence in Umbria, Italy. J Neurol Neurosurg Psychiatry. 1991; 54(8): 695–8
Hemorrhagic Stroke	Ministry of Health (Italy). Italy National Hospital Discharge Database 2012
Hemorrhagic Stroke	Federal Public Service Health, Food Chain Safety, and Environment (Belgium). Belgium Minimum Clinical Summary 2000
Hemorrhagic Stroke	Federal Public Service Health, Food Chain Safety, and Environment (Belgium). Belgium Minimum Clinical Summary 2001
Hemorrhagic Stroke	Federal Public Service Health, Food Chain Safety, and Environment (Belgium). Belgium Minimum Clinical Summary 2002
Hemorrhagic Stroke	Federal Public Service Health, Food Chain Safety, and Environment (Belgium). Belgium Minimum Clinical Summary 2005
Hemorrhagic Stroke	Federal Public Service Health, Food Chain Safety, and Environment (Belgium). Belgium Minimum Clinical Summary 2006
Hemorrhagic Stroke	Federal Public Service Health, Food Chain Safety, and Environment (Belgium). Belgium Minimum Clinical Summary 2007
Hemorrhagic Stroke	Federal Public Service Health, Food Chain Safety, and Environment (Belgium). Belgium Minimum Hospital Summary 2010
Hemorrhagic Stroke	Federal Public Service Health, Food Chain Safety, and Environment (Belgium). Belgium Minimum Hospital Summary 2011
Hemorrhagic Stroke	Federal Public Service Health, Food Chain Safety, and Environment (Belgium). Belgium Minimum Hospital Summary 2012
Hemorrhagic Stroke	Federal Public Service Health, Food Chain Safety, and Environment (Belgium). Belgium Minimum Hospital Summary 2013
Hemorrhagic Stroke	Ministry of Health (Italy). Italy National Hospital Discharge Database 1970
Hemorrhagic Stroke	Ministry of Health (Italy). Italy National Hospital Discharge Database 1971
Hemorrhagic Stroke	Ministry of Health (Italy). Italy National Hospital Discharge Database 1972
Hemorrhagic Stroke	Ministry of Health (Italy). Italy National Hospital Discharge Database 1973
Hemorrhagic Stroke	Ministry of Health (Italy). Italy National Hospital Discharge Database 1974
Hemorrhagic Stroke	Ministry of Health (Italy). Italy National Hospital Discharge Database 1975
Hemorrhagic Stroke	Ministry of Health (Italy). Italy National Hospital Discharge Database 1976
Hemorrhagic Stroke	Ministry of Health (Italy). Italy National Hospital Discharge Database 1977
Hemorrhagic Stroke	Ministry of Health (Italy). Italy National Hospital Discharge Database 1978
Hemorrhagic Stroke	Ministry of Health (Italy). Italy National Hospital Discharge Database 1979
Hemorrhagic Stroke	Ministry of Health (Italy). Italy National Hospital Discharge Database 1980

Hemorrhagic Stroke	Ministry of Health (Italy). Italy National Hospital Discharge Database 1981
Hemorrhagic Stroke	Ministry of Health (Italy). Italy National Hospital Discharge Database 1982
Hemorrhagic Stroke	Ministry of Health (Italy). Italy National Hospital Discharge Database 1983
Hemorrhagic Stroke	Ministry of Health (Italy). Italy National Hospital Discharge Database 1984
Hemorrhagic Stroke	Ministry of Health (Italy). Italy National Hospital Discharge Database 1985
Hemorrhagic Stroke	Ministry of Health (Italy). Italy National Hospital Discharge Database 1986
Hemorrhagic Stroke	Ministry of Health (Italy). Italy National Hospital Discharge Database 1987
Hemorrhagic Stroke	Ministry of Health (Italy). Italy National Hospital Discharge Database 1988
Hemorrhagic Stroke	Ministry of Health (Italy). Italy National Hospital Discharge Database 1989
Hemorrhagic Stroke	Ministry of Health (Italy). Italy National Hospital Discharge Database 1990
Hemorrhagic Stroke	Ministry of Health (Italy). Italy National Hospital Discharge Database 1991
Hemorrhagic Stroke	Ministry of Health (Italy). Italy National Hospital Discharge Database 1992
Hemorrhagic Stroke	Ministry of Health (Italy). Italy National Hospital Discharge Database 1993
Hemorrhagic Stroke	Ministry of Health (Italy). Italy National Hospital Discharge Database 1994
Hemorrhagic Stroke	Ministry of Health (Italy). Italy National Hospital Discharge Database 1995
Hemorrhagic Stroke	Ministry of Health (Italy). Italy National Hospital Discharge Database 1996
Hemorrhagic Stroke	Ministry of Health (Italy). Italy National Hospital Discharge Database 1997
Hemorrhagic Stroke	Ministry of Health (Italy). Italy National Hospital Discharge Database 1998
Hemorrhagic Stroke	Ministry of Health (Italy). Italy National Hospital Discharge Database 1999
Hemorrhagic Stroke	Ministry of Health (Italy). Italy National Hospital Discharge Database 2000
Hemorrhagic Stroke	Ministry of Health (Italy). Italy National Hospital Discharge Database 2013
Hemorrhagic Stroke	Ministry of Health (Italy). Italy National Hospital Discharge Database 2014
Hemorrhagic Stroke	National Bureau of Statistics of China. China Statistical Yearbook 2015. Beijing, China: National Bureau of Statistics of China
Hemorrhagic Stroke	Ghana Statistical Service. Ghana Living Standards Measurement Survey 1991-1992. Accra, Ghana: Ghana Statistical Service
Hemorrhagic Stroke	Ghana Statistical Service. Ghana Living Standards Survey 1998-1999
Hemorrhagic Stroke	Ghana Statistical Service. Ghana Living Standards Measurement Survey 2005-2006. Accra, Ghana: Ghana Statistical Service
Hemorrhagic Stroke	Institute of Sociology, Russian Academy of Sciences, Paragon Research, University of North Carolina, World Bank. Kyrgyzstan Living Standards Measurement Survey 1993. Washington DC, United States: World Bank
Hemorrhagic Stroke	World Health Organization (WHO). Czech Republic World Health Survey 2002-2003. Geneva, Switzerland: World Health Organization (WHO), 2005
Hemorrhagic Stroke	World Health Organization (WHO). Ghana World Health Survey 2003. Geneva, Switzerland: World Health Organization (WHO), 2005
Hemorrhagic Stroke	World Health Organization (WHO). Luxembourg World Health Survey 2003. Geneva, Switzerland: World Health Organization (WHO), 2005
Hemorrhagic Stroke	World Health Organization (WHO). Sri Lanka World Health Survey 2003. Geneva, Switzerland: World Health Organization (WHO), 2005
Hemorrhagic Stroke	World Health Organization (WHO). Swaziland World Health Survey 2003. Geneva, Switzerland: World Health Organization (WHO), 2005
Hemorrhagic Stroke	University of Wisconsin-Madison, Inter-University Consortium for Political and Social Research (ICPSR), Chronic Disease Research Centre (CDRC), University of the West Indies. Barbados - Bridgetown Survey on Health, Well-Being, and Aging in Latin America and the Caribbean 1999-2000. Ann Arbor, United States: Inter-University
Hemorrhagic Stroke	National Statistical Committee of the Kyrgyz Republic, Research Triangle Institute, Inc. (RTI), World Bank. Kyrgyzstan Living Standards Measurement Survey 1997. Washington DC, United States: World Bank
Hemorrhagic Stroke	National Statistical Committee of the Kyrgyz Republic, Research Triangle Institute, Inc. (RTI), World Bank. Kyrgyzstan Living Standards Measurement Survey 1998. Washington DC, United States: World Bank
Hemorrhagic Stroke	Ashok PP, Radhakrishnan K, Sridharan R, el-Mangoush MA. Incidence and pattern of cerebrovascular diseases in Benghazi, Libya. J Neurol Neurosurg Psychiatr. 1986; 49(5): 519-23
Hemorrhagic Stroke	Morikawa Y, Nakagawa H, Naruse Y, Nishijo M, Miura K, Tabata M, Hirokawa W, Kagamimori S, Honda M, Yoshita K, Hayashi K. Trends in Stroke Incidence and Acute Case Fatality in a Japanese Rural Area: The Oyabe Study. Stroke. 2000; 31(7): 1583-7
Hemorrhagic Stroke	Kita Y, Okayama A, Ueshima H, Wada M, Nozaki A, Choudhary SR, Bonita R, Inamoto Y, Kasamatsu T. Stroke incidence and case fatality in Shiga, Japan 1989-1993. Int J Epidemiol. 1999; 28(6): 1059-65
Hemorrhagic Stroke	Gallup Europe, World Health Organization (WHO). Argentina WHO Multi-country Survey Study on Health and Health System Responsiveness 2000-2001. Geneva, Switzerland: World Health Organization (WHO)
Hemorrhagic Stroke	Kita Y, Turin TC, Ichikawa M, Sugihara H, Morita Y, Tomioka N, Rumana N, Okayama A, Nakamura Y, Abbott RD, Ueshima H. Trend of stroke incidence in a Japanese population: Takashima stroke registry, 1990-2001. Int J Stroke. 2009; 4(4): 241-9
Hemorrhagic Stroke	Kulesh SD, Filina NA, Frantava NM, Zhytko NL, Kastsinevich TM, Kliatskova LA, Shumskas MS, Hilz MJ, Schwab S, Kolominsky-Rabas PL. Incidence and Case-Fatality of Stroke on the East Border of the European Union The Grodno Stroke Study. Stroke. 2010; 41(12): 2726-30
Hemorrhagic Stroke	Melcon CM, Melcon MO. Prevalence of stroke in an Argentine community. Neuroepidemiology. 2006; 27(2): 81-8
Hemorrhagic Stroke	General Administration of Statistics and Censuses (El Salvador), Ministry of Economy (El Salvador). El Salvador Multipurpose Household Survey 2013. San Salvador, El Salvador: General Administration of Statistics and Censuses (El Salvador)
Hemorrhagic Stroke	Ghana Statistical Service, World Bank. Ghana Living Standards Measurement Survey 2012-2013. Accra, Ghana: Ghana Statistical Service
Hemorrhagic Stroke	MEMRB International, World Health Organization (WHO). Cyprus WHO Multi-country Survey Study on Health and Health System Responsiveness 2000-2001. Geneva, Switzerland: World Health Organization (WHO)
Hemorrhagic Stroke	Institute of Health Information and Statistics of the Czech Republic, International Research Associates (INRA) Europe, World Health Organization (WHO). Czech Republic WHO Multi-country Survey Study on Health and Health System Responsiveness 2000-2001. Geneva, Switzerland: World Health Organization (WHO)
Hemorrhagic Stroke	National Statistical Committee of the Kyrgyz Republic, SIAR Research and Consulting (Kyrgyzstan), World Health Organization (WHO). Kyrgyzstan WHO Multi-country Survey Study on Health and Health System Responsiveness 2000-2001. Geneva, Switzerland: World Health Organization (WHO)
Hemorrhagic Stroke	International Research Associates (INRA) Europe, World Health Organization (WHO). Luxembourg WHO Multi-country Survey Study on Health and Health System Responsiveness 2000-2001. Geneva, Switzerland: World Health Organization (WHO)
Hemorrhagic Stroke	Börsch-Supan, A. (2015). Survey of Health, Ageing and Retirement in Europe (SHARE) Wave 5. Release version: 1.0.0. SHARE-ERIC. Data set. DOI: 10.6103/SHARE.w5.100
Hemorrhagic Stroke	Börsch-Supan, A. (2015). Survey of Health, Ageing and Retirement in Europe (SHARE) Wave 5. Release version: 1.0.0. SHARE-ERIC. Data set. DOI: 10.6103/SHARE.w5.100
Hemorrhagic Stroke	Belarusian State University, Concluzia-Prim Center for Survey Methodology (Moldova), Institute for Advanced Studies (Austria), London School of Hygiene and Tropical Medicine, University of Aberdeen. Belarus Health in Times of Transition Household Survey 2010
Hemorrhagic Stroke	Studies (Austria), International Centre for Sociological, Political and Social Psychological Research (Kyrgyzstan), London School of Hygiene and Tropical Medicine, University of Aberdeen. Kyrgyzstan Health in Times of Transition Household Survey 2011
Hemorrhagic Stroke	General Administration of Statistics and Censuses (El Salvador), Ministry of Economy (El Salvador). El Salvador Multipurpose Household Survey 2014. San Salvador, El Salvador: General Administration of Statistics and Censuses (El Salvador)

Hemorrhagic Stroke	Hattori N, Hirayama T, Katayama Y. Medical care for chronic-phase stroke in Japan. Neurol Med Chir (Tokyo). 2012; 52(4): 175–80
Hemorrhagic Stroke	Department of Economics, University of Chile, Ministry of Planning (Chile). Chile National Socioeconomic Characterization Survey 1992
Hemorrhagic Stroke	Department of Economics, University of Chile, Ministry of Planning (Chile). Chile National Socioeconomic Characterization Survey 1994. Santiago, Chile: Ministry of Social Development (Chile)
Hemorrhagic Stroke	Department of Economics, University of Chile, Ministry of Planning (Chile). Chile National Socioeconomic Characterization Survey 1996. Santiago, Chile: Ministry of Social Development (Chile)
Hemorrhagic Stroke	Department of Economics, University of Chile, Ministry of Planning (Chile). Chile National Socioeconomic Characterization Survey 1998
Hemorrhagic Stroke	Department of Economics, University of Chile, Ministry of Planning (Chile). Chile National Socioeconomic Characterization Survey 2000. Santiago, Chile: Ministry of Social Development (Chile)
Hemorrhagic Stroke	Ministry of Public Health (Thailand). Thailand National Health and Examination Survey 2003-2004
Hemorrhagic Stroke	World Health Organization (WHO). Denmark World Health Survey 2003. Geneva, Switzerland: World Health Organization (WHO), 2005
Hemorrhagic Stroke	World Health Organization (WHO). Estonia World Health Survey 2003. Geneva, Switzerland: World Health Organization (WHO), 2005
Hemorrhagic Stroke	World Health Organization (WHO). Guatemala World Health Survey 2003. Geneva, Switzerland: World Health Organization (WHO), 2005
Hemorrhagic Stroke	World Health Organization (WHO). Hungary World Health Survey 2003. Geneva, Switzerland: World Health Organization (WHO), 2005
Hemorrhagic Stroke	World Health Organization (WHO). Morocco World Health Survey 2003. Geneva, Switzerland: World Health Organization (WHO), 2005
Hemorrhagic Stroke	World Health Organization (WHO). Zimbabwe World Health Survey 2003. Geneva, Switzerland: World Health Organization (WHO), 2005
Hemorrhagic Stroke	Department of Economics, University of Chile, Ministry of Planning (Chile). Chile National Socioeconomic Characterization Survey 2003. Santiago, Chile: Ministry of Social Development (Chile)
Hemorrhagic Stroke	University of Wisconsin-Madison, Inter-University Consortium for Political and Social Research (ICPSR), Institute of Nutrition and Food Technology (INTA), University of Chile, Center for Geriatrics and Gerontology, Pontifical Catholic University of Chile. Chile - Santiago Survey on Health, Well-Being, and Aging in Latin America and the
Hemorrhagic Stroke	Directorate of Statistics of the High Commission for Planning (Morocco), World Bank. Morocco Living Standards Measurement Survey 1990-1991
Hemorrhagic Stroke	Department of Economics, University of Chile, Ministry of Planning (Chile). Chile National Socioeconomic Characterization Survey 2006. Santiago, Chile: Ministry of Social Development (Chile)
Hemorrhagic Stroke	Jorgensen HS, Plesner AM, Hubbe P, Larsen K. Marked increase of stroke incidence in men between 1972 and 1990 in Frederiksberg, Denmark. Stroke. 1992; 23(12): 1701-4
Hemorrhagic Stroke	Lavados PM, Sacks C, Prina L, Escobar A, Tossi C, Araya F, Feuerhake W, Galvez M, Salinas R, Alvarez G. Incidence, 30-day case-fatality rate, and prognosis of stroke in Iquique, Chile: a 2-year community-based prospective study (PISCIS project). Lancet. 2005; 365(9478): 2206-15
Hemorrhagic Stroke	Vibo R, Kõrv J, Roose M. The Third Stroke Registry in Tartu, Estonia: Decline of Stroke Incidence and 28-Day Case-Fatality Rate Since 1991. Stroke. 2005; 36(12): 2544-8
Hemorrhagic Stroke	Health Promotion Research Institute (Hungary), Hungarian Gallup Institute. Hungary National Population Health Survey 2000
Hemorrhagic Stroke	Kôrv J, Roose M, Kaasik A-E. Changed Incidence and Case-Fatality Rates of First-Ever Stroke Between 1970 and 1993 in Tartu, Estonia. Stroke. 1996; 27(2): 199-203
Hemorrhagic Stroke	Matenga J. Stroke incidence rates among black residents of Harare - a prospective community-based study. S Afr Med J. 1997; 87(5): 606-8
Hemorrhagic Stroke	Fuh JL, Wang SJ, Larson EB, Liu HC. Prevalence of stroke in Kinmen. Stroke. 1996; 27(8): 1338-41
Hemorrhagic Stroke	Huang Z-S, Chiang T-L, Lee T-K. Stroke Prevalence in Taiwan: Findings From the 1994 National Health Interview Survey. Stroke. 1997; 28(8): 1579-84
Hemorrhagic Stroke	Ministry of Health (Chile). Chile Hospital Discharges 2001. Santiago, Chile: Ministry of Health (Chile)
Hemorrhagic Stroke	Ministry of Health (Chile). Chile Hospital Discharges 2002. Santiago, Chile: Ministry of Health (Chile)
Hemorrhagic Stroke	Ministry of Health (Chile). Chile Hospital Discharges 2003. Santiago, Chile: Ministry of Health (Chile)
Hemorrhagic Stroke	Ministry of Health (Chile). Chile Hospital Discharges 2004. Santiago, Chile: Ministry of Health (Chile)
Hemorrhagic Stroke	Ministry of Health (Chile). Chile Hospital Discharges 2005. Santiago, Chile: Ministry of Health (Chile)
Hemorrhagic Stroke	Ministry of Health (Chile). Chile Hospital Discharges 2006. Santiago, Chile: Ministry of Health (Chile)
Hemorrhagic Stroke	Ministry of Health (Chile). Chile Hospital Discharges 2007. Santiago, Chile: Ministry of Health (Chile)
Hemorrhagic Stroke	Ministry of Health (Chile). Chile Hospital Discharges 2008. Santiago, Chile: Ministry of Health (Chile)
Hemorrhagic Stroke	Ministry of Health (Chile). Chile Hospital Discharges 2009. Santiago, Chile: Ministry of Health (Chile)
Hemorrhagic Stroke	Ministry of Health (Chile). Chile Hospital Discharges 2010. Santiago, Chile: Ministry of Health (Chile)
Hemorrhagic Stroke	Ministry of Health (Chile). Chile Hospital Discharges 2011. Santiago, Chile: Ministry of Health (Chile)
Hemorrhagic Stroke	Ministry of Planning (Chile), Social Observatory, Alberto Hurtado University. Chile National Socioeconomic Characterization Survey 2009. Santiago, Chile: Ministry of Social Development (Chile)
Hemorrhagic Stroke	Korea Centers for Disease Control and Prevention. South Korea National Health and Nutrition Examination Survey 2012
Hemorrhagic Stroke	Competence Centre for Clinical Quality and Health Informatics West (Denmark). Danish Stroke Registry Data 2009. [Unpublished]
Hemorrhagic Stroke	Hu HH, Sheng WY, Chu FL, Lan CF, Chiang BN. Incidence of stroke in Taiwan. Stroke. 1992; 23: 1237–1241
Hemorrhagic Stroke	Ministry of Health (Chile). Chile Hospital Discharges 2012. Santiago, Chile: Ministry of Health (Chile)
Hemorrhagic Stroke	University of Concepcion (Chile), World Health Organization (WHO). Chile WHO Multi-country Survey Study on Health and Health System Responsiveness 2000-2001. Geneva, Switzerland: World Health Organization (WHO)
Hemorrhagic Stroke	Statistics Denmark, World Health Organization (WHO). Denmark WHO Multi-country Survey Study on Health and Health System Responsiveness 2000-2001. Geneva, Switzerland: World Health Organization (WHO)
Hemorrhagic Stroke	International Research Associates (INRA) Europe, World Health Organization (WHO). Estonia WHO Multi-country Survey Study on Health and Health System Responsiveness 2000-2001. Geneva, Switzerland: World Health Organization (WHO)
Hemorrhagic Stroke	Szonda Ipsos, World Health Organization (WHO). Hungary WHO Multi-country Survey Study on Health and Health System Responsiveness 2000-2001. Geneva, Switzerland: World Health Organization (WHO)
Hemorrhagic Stroke	Graduate School of Public Health, Seoul National University, World Health Organization (WHO). South Korea WHO Multi-country Survey Study on Health and Health System Responsiveness 2000-2001. Geneva, Switzerland: World Health Organization (WHO)
Hemorrhagic Stroke	Gallup Europe, World Health Organization (WHO). Morocco WHO Multi-country Survey Study on Health and Health System Responsiveness 2000-2001. Geneva, Switzerland: World Health Organization (WHO)
Hemorrhagic Stroke	International Research Associates (INRA) Europe, World Health Organization (WHO). Malta WHO Multi-country Survey Study on Health and Health System Responsiveness 2000-2001. Geneva, Switzerland: World Health Organization (WHO)
Hemorrhagic Stroke	Mahidol University, World Health Organization (WHO). Thailand WHO Multi-country Survey Study on Health and Health System Responsiveness 2000-2001. Geneva, Switzerland: World Health Organization (WHO)
Hemorrhagic Stroke	Börsch-Supan, A. (2015). Survey of Health, Ageing and Retirement in Europe (SHARE) Wave 5. Release version: 1.0.0. SHARE-ERIC. Data set. DOI: 10.6103/SHARE.w5.100
Hemorrhagic Stroke	Börsch-Supan, A. (2015). Survey of Health, Ageing and Retirement in Europe (SHARE) Wave 5. Release version: 1.0.0. SHARE-ERIC. Data set. DOI: 10.6103/SHARE.w5.100
Hemorrhagic Stroke	National Office of Statistics (Cuba). Cuba Statistical Yearbook 2012. Havana, Cuba: National Office of Statistics (Cuba)

Hemorrhagic Stroke	Kim J-M, Stewart R, Park M-S, Kang H-J, Kim S-W, Shin I-S, Kim H-R, Shin M-G, Cho K-H, Yoon J-S. Associations of BDNF genotype and promoter methylation with acute and long-term stroke outcomes in an East Asian cohort. PLoS One. 2012; 7(12): e51280
Hemorrhagic Stroke	Vibo R, Korv J, Roose M. One-year outcome after first-ever stroke according to stroke subtype, severity, risk factors and pre-stroke treatment. A population-based study from Tartu, Estonia. Eur J Neurol. 2007; 14(4): 435–9
Hemorrhagic Stroke	Kang H-J, Stewart R, Park M-S, Bae K-Y, Kim S-W, Kim J-M, Shin I-S, Cho K-H, Yoon J-S. White matter hyperintensities and functional outcomes at 2 weeks and 1 year after stroke. Cerebrovasc Dis. 2013; 35(2): 138–45
Hemorrhagic Stroke	Truelsen T, Gronbaek M, Schnohr P, Boysen G. Stroke case fatality in Denmark from 1977 to 1992: the Copenhagen City Heart Study. Neuroepidemiology. 2002; 21(1): 22–7
Hemorrhagic Stroke	Danish Health and Medicines Authority. Denmark National Patient Registry 2003
Hemorrhagic Stroke	Danish Health and Medicines Authority. Denmark National Patient Registry 2004
Hemorrhagic Stroke	Danish Health and Medicines Authority. Denmark National Patient Registry 2005
Hemorrhagic Stroke	Danish Health and Medicines Authority. Denmark National Patient Registry 2006
Hemorrhagic Stroke	Danish Health and Medicines Authority. Denmark National Patient Registry 2007
Hemorrhagic Stroke	Danish Health and Medicines Authority. Denmark National Patient Registry 2008
Hemorrhagic Stroke	Danish Health and Medicines Authority. Denmark National Patient Registry 2009
Hemorrhagic Stroke	Ministry of Social Affairs (Estonia), National Institute for Health Development (Estonia). Estonia Hospital Inpatient Discharges 2003
Hemorrhagic Stroke	Ministry of Social Affairs (Estonia), National Institute for Health Development (Estonia). Estonia Hospital Inpatient Discharges 2004
Hemorrhagic Stroke	Ministry of Social Affairs (Estonia), National Institute for Health Development (Estonia). Estonia Hospital Inpatient Discharges 2005
Hemorrhagic Stroke	Ministry of Social Affairs (Estonia), National Institute for Health Development (Estonia). Estonia Hospital Inpatient Discharges 2006
Hemorrhagic Stroke	Ministry of Social Affairs (Estonia), National Institute for Health Development (Estonia). Estonia Hospital Inpatient Discharges 2007
Hemorrhagic Stroke	Ministry of Social Affairs (Estonia), National Institute for Health Development (Estonia). Estonia Hospital Inpatient Discharges 2008
Hemorrhagic Stroke	Ministry of Social Affairs (Estonia), National Institute for Health Development (Estonia). Estonia Hospital Inpatient Discharges 2009
Hemorrhagic Stroke	Ministry of Social Affairs (Estonia), National Institute for Health Development (Estonia). Estonia Hospital Inpatient Discharges 2010
Hemorrhagic Stroke	Ministry of Social Affairs (Estonia), National Institute for Health Development (Estonia). Estonia Hospital Inpatient Discharges 2011
Hemorrhagic Stroke	Center for Health Care Information (GYOGYINFOK) (Hungary). Hungary Hospital Inpatient Discharges 1990
Hemorrhagic Stroke	National Institute for Health Development (Estonia). Estonia Hospital Inpatient Discharges 2012
Hemorrhagic Stroke	National Institute for Health Development (Estonia). Estonia Hospital Inpatient Discharges 2013
Hemorrhagic Stroke	National Institute for Health Development (Estonia). Estonia Hospital Inpatient Discharges 2014
Hemorrhagic Stroke	Institute of Experimental and Clinical Medicine (Estonia). Estonia Hospital Inpatient Discharges 1999
Hemorrhagic Stroke	Institute of Experimental and Clinical Medicine (Estonia). Estonia Hospital Inpatient Discharges 1998
Hemorrhagic Stroke	Institute of Experimental and Clinical Medicine (Estonia). Estonia Hospital Inpatient Discharges 1997
Hemorrhagic Stroke	Institute of Experimental and Clinical Medicine (Estonia). Estonia Hospital Inpatient Discharges 1996
Hemorrhagic Stroke	Institute of Experimental and Clinical Medicine (Estonia). Estonia Hospital Inpatient Discharges 1995
Hemorrhagic Stroke	Institute of Experimental and Clinical Medicine (Estonia). Estonia Hospital Inpatient Discharges 1994
Hemorrhagic Stroke	Institute of Experimental and Clinical Medicine (Estonia). Estonia Hospital Inpatient Discharges 1993
Hemorrhagic Stroke	Institute of Experimental and Clinical Medicine (Estonia). Estonia Hospital Inpatient Discharges 1992
Hemorrhagic Stroke	Institute of Experimental and Clinical Medicine (Estonia). Estonia Hospital Inpatient Discharges 1991
Hemorrhagic Stroke	Institute of Experimental and Clinical Medicine (Estonia). Estonia Hospital Inpatient Discharges 1990
Hemorrhagic Stroke	Institute of Experimental and Clinical Medicine (Estonia). Estonia Hospital Inpatient Discharges 1989
Hemorrhagic Stroke	Institute of Experimental and Clinical Medicine (Estonia). Estonia Hospital Inpatient Discharges 1988
Hemorrhagic Stroke	Institute of Experimental and Clinical Medicine (Estonia). Estonia Hospital Inpatient Discharges 1987
Hemorrhagic Stroke	Institute of Experimental and Clinical Medicine (Estonia). Estonia Hospital Inpatient Discharges 1986
Hemorrhagic Stroke	Institute of Experimental and Clinical Medicine (Estonia). Estonia Hospital Inpatient Discharges 1985
Hemorrhagic Stroke	Institute of Experimental and Clinical Medicine (Estonia). Estonia Hospital Inpatient Discharges 1980
Hemorrhagic Stroke	Center for Health Care Information (GYOGYINFOK) (Hungary). Hungary Hospital Inpatient Discharges 1991
Hemorrhagic Stroke	Center for Health Care Information (GYOGYINFOK) (Hungary). Hungary Hospital Inpatient Discharges 1992
Hemorrhagic Stroke	Center for Health Care Information (GYOGYINFOK) (Hungary). Hungary Hospital Inpatient Discharges 1993
Hemorrhagic Stroke	Center for Health Care Information (GYOGYINFOK) (Hungary). Hungary Hospital Inpatient Discharges 1994
Hemorrhagic Stroke	Center for Health Care Information (GYOGYINFOK) (Hungary). Hungary Hospital Inpatient Discharges 1995
Hemorrhagic Stroke	Center for Health Care Information (GYOGYINFOK) (Hungary). Hungary Hospital Inpatient Discharges 1996
Hemorrhagic Stroke	Center for Health Care Information (GYOGYINFOK) (Hungary). Hungary Hospital Inpatient Discharges 1997
Hemorrhagic Stroke	Center for Health Care Information (GYOGYINFOK) (Hungary). Hungary Hospital Inpatient Discharges 1998
Hemorrhagic Stroke	Center for Health Care Information (GYOGYINFOK) (Hungary). Hungary Hospital Inpatient Discharges 1999
Hemorrhagic Stroke	Center for Health Care Information (GYOGYINFOK) (Hungary). Hungary Hospital Inpatient Discharges 2000
Hemorrhagic Stroke	Center for Health Care Information (GYOGYINFOK) (Hungary). Hungary Hospital Inpatient Discharges 2001
Hemorrhagic Stroke	Center for Health Care Information (GYOGYINFOK) (Hungary). Hungary Hospital Inpatient Discharges 2002
Hemorrhagic Stroke	Center for Health Care Information (GYOGYINFOK) (Hungary). Hungary Hospital Inpatient Discharges 2003
Hemorrhagic Stroke	National Institute for Strategic Health Research (ESKI) (Hungary). Hungary Hospital Inpatient Discharges 2013
Hemorrhagic Stroke	National Institute for Strategic Health Research (ESKI) (Hungary). Hungary Hospital Inpatient Discharges 2014
Hemorrhagic Stroke	Danish Health and Medicines Authority. Denmark National Patient Registry 1979
Hemorrhagic Stroke	Danish Health and Medicines Authority. Denmark National Patient Registry 1980
Hemorrhagic Stroke	Danish Health and Medicines Authority. Denmark National Patient Registry 1981
Hemorrhagic Stroke	Danish Health and Medicines Authority. Denmark National Patient Registry 1982
Hemorrhagic Stroke	Danish Health and Medicines Authority. Denmark National Patient Registry 1983
Hemorrhagic Stroke	Danish Health and Medicines Authority. Denmark National Patient Registry 1984
Hemorrhagic Stroke	Danish Health and Medicines Authority. Denmark National Patient Registry 1985
Hemorrhagic Stroke	Danish Health and Medicines Authority. Denmark National Patient Registry 1986
Hemorrhagic Stroke	Danish Health and Medicines Authority. Denmark National Patient Registry 1987
Hemorrhagic Stroke	Danish Health and Medicines Authority. Denmark National Patient Registry 1988
Hemorrhagic Stroke	Danish Health and Medicines Authority. Denmark National Patient Registry 1989
Hemorrhagic Stroke	Danish Health and Medicines Authority. Denmark National Patient Registry 1990
Hemorrhagic Stroke	Danish Health and Medicines Authority. Denmark National Patient Registry 1991
Hemorrhagic Stroke	Danish Health and Medicines Authority. Denmark National Patient Registry 1992
Hemorrhagic Stroke	Danish Health and Medicines Authority. Denmark National Patient Registry 1993
Hemorrhagic Stroke	Danish Health and Medicines Authority. Denmark National Patient Registry 1994

Hemorrhagic Stroke	Danish Health and Medicines Authority. Denmark National Patient Registry 1995
Hemorrhagic Stroke	Danish Health and Medicines Authority. Denmark National Patient Registry 1996
Hemorrhagic Stroke	Danish Health and Medicines Authority. Denmark National Patient Registry 1997
Hemorrhagic Stroke	Danish Health and Medicines Authority. Denmark National Patient Registry 1998
Hemorrhagic Stroke	Danish Health and Medicines Authority. Denmark National Patient Registry 1999
Hemorrhagic Stroke	Danish Health and Medicines Authority. Denmark National Patient Registry 2000
Hemorrhagic Stroke	Danish Health and Medicines Authority. Denmark National Patient Registry 2001
Hemorrhagic Stroke	Danish Health and Medicines Authority. Denmark National Patient Registry 2002
Hemorrhagic Stroke	Danish Health and Medicines Authority. Denmark National Patient Registry 2010
Hemorrhagic Stroke	Danish Health and Medicines Authority. Denmark National Patient Registry 2011
Hemorrhagic Stroke	Danish Health and Medicines Authority. Denmark National Patient Registry 2012
Hemorrhagic Stroke	Danish Health and Medicines Authority. Denmark National Patient Registry 2013
Hemorrhagic Stroke	National State Statistical Agency (Tajikistan), World Bank. Tajikistan Living Standards Measurement Survey 2003
Hemorrhagic Stroke	National State Statistical Agency (Tajikistan), World Bank. Tajikistan Living Standards Measurement Survey 2007
Hemorrhagic Stroke	Planning Commission (Tanzania), University of Dar es Salaam, World Bank. Tanzania Living Standards Measurement Study 1993-1994. Washington DC, United States: World Bank
Hemorrhagic Stroke	National Statistics Directorate (Timor-Leste), World Bank. Timor-Leste Living Standards and Measurement Survey 2001. Washington DC, United States: World Bank
Hemorrhagic Stroke	Palestinian Central Bureau of Statistics. Palestine Demographic and Health Survey 2004
Hemorrhagic Stroke	World Health Organization (WHO). Ethiopia World Health Survey 2003. Geneva, Switzerland: World Health Organization (WHO), 2005
Hemorrhagic Stroke	World Health Organization (WHO). Finland World Health Survey 2004. Geneva, Switzerland: World Health Organization (WHO), 2005
Hemorrhagic Stroke	World Health Organization (WHO). Latvia World Health Survey 2003. Geneva, Switzerland: World Health Organization (WHO), 2005
Hemorrhagic Stroke	World Health Organization (WHO). Netherlands World Health Survey 2004. Geneva, Switzerland: World Health Organization (WHO), 2005
Hemorrhagic Stroke	World Health Organization (WHO). Uruguay World Health Survey 2002-2003. Geneva, Switzerland: World Health Organization (WHO), 2005
Hemorrhagic Stroke	National State Statistical Agency (Tajikistan), World Bank. Tajikistan Living Standards Measurement Survey 2009
Hemorrhagic Stroke	National Statistics Directorate (Timor-Leste), World Bank. Timor-Leste Living Standards and Measurement Survey 2007-2008. Washington DC, United States: World Bank
Hemorrhagic Stroke	National Bureau of Statistics (Tanzania). Tanzania Living Standards Measurement Study - Integrated Surveys on Agriculture 2010-2011. Dar es Salaam, Tanzania: National Bureau of Statistics (Tanzania)
Hemorrhagic Stroke	Numminen H, Kotila M, Waltimo O, Aho K, Kaste M. Declining Incidence and Mortality Rates of Stroke in Finland From 1972 to 1991: Results of Three Population-Based Stroke Registers. Stroke. 1996; 27(9): 1487-91
Hemorrhagic Stroke	Immonen-Räihä P, Mähönen M, Tuomilehto J, Salomaa V, Kaarsalo E, Narva EV, Salmi K, Sarti C, Sivenius J, Alhainen K, Torppa J. Trends in Case-Fatality of Stroke in Finland During 1983 to 1992. Stroke. 1997; 28(12): 2493-9
Hemorrhagic Stroke	Vaartjes I, Reitsma JB, de Bruin A, Berger-van Sijl M, Bos MJ, Breteler MM, Grobbee DE, Bots ML. Nationwide incidence of first stroke and TIA in the Netherlands. Eur J Neurol. 2008; 15(12): 1315-23
Hemorrhagic Stroke	Walker R, Unwin N, Mugusi F, Swai M, Aris E, Jusabani A, Kabadi G, Gray W, Lewanga M, Alberti G, Whiting D. Stroke incidence in rural and urban Tanzania: a prospective, community-based study. Lancet Neurol. 2010; 9(8): 786-92
Hemorrhagic Stroke	Sweileh WM, Sawalha AF, Al-Aqad SM, Zyoud SH, Al-Jabi SW. The Epidemiology of Stroke in Northern Palestine: A 1-Year, Hospital-Based Study. J Stroke Cerebrovasc Dis. 2008; 17(6): 406-11
Hemorrhagic Stroke	De Jesús Llibre J, Valhuerdi A, Fernández O, Llibre JC, Porto R, López AM, Marcheco B, Moreno C. Prevalence of stroke and associated risk factors in older adults in Havana City and Matanzas Provinces, Cuba (10/66 population-based study). MEDICC Rev. 2010; 12(3): 20-6
Hemorrhagic Stroke	Tekle-Haimanot R, Abebe M, Gebre-Mariam A, Forsgren L, Heijbel J, Holmgren G, Ekstedt J. Community-based study of neurological disorders in rural central Ethiopia. Neuroepidemiology. 1990; 9(5): 263-77
Hemorrhagic Stroke	Venketasubramanian N, Tan LCS, Sahadevan S, Chin JJ, Krishnamoorthy ES, Hong CY, Saw SM. Prevalence of Stroke Among Chinese, Malay, and Indian Singaporeans. Stroke. 2005; 36(3): 551-6
Hemorrhagic Stroke	Walker R, McLarty D, Masuki G, Kitange H, Whiting D, Moshi A, Massawe J, Amaro R, Mhina A, Alberti K. Age specific prevalence of impairment and disability relating to hemiplegic stroke in the Hai District of northern Tanzania. J Neurol Neurosurg Psychiatr. 2000; 68(6): 744-9
Hemorrhagic Stroke	Wieberdink RG, Ikram MA, Hofman A, Koudstaal PJ, Breteler MMB. Trends in stroke incidence rates and stroke risk factors in Rotterdam, the Netherlands from 1990 to 2008. Eur J Epidemiol. 2012; 27(4): 287-95
Hemorrhagic Stroke	Dutch Hospital Data (DHD). Netherlands National Medical Registry 1998
Hemorrhagic Stroke	Dutch Hospital Data (DHD). Netherlands National Medical Registry 1999
Hemorrhagic Stroke	Dutch Hospital Data (DHD). Netherlands National Medical Registry 2000
Hemorrhagic Stroke	Dutch Hospital Data (DHD). Netherlands National Medical Registry 2001
Hemorrhagic Stroke	Dutch Hospital Data (DHD). Netherlands National Medical Registry 2002
Hemorrhagic Stroke	Dutch Hospital Data (DHD). Netherlands National Medical Registry 2003
Hemorrhagic Stroke	Dutch Hospital Data (DHD). Netherlands National Medical Registry 2004
Hemorrhagic Stroke	Dutch Hospital Data (DHD). Netherlands National Medical Registry 2006
Hemorrhagic Stroke	Dutch Hospital Data (DHD). Netherlands National Medical Registry 2007
Hemorrhagic Stroke	Dutch Hospital Data (DHD). Netherlands National Medical Registry 2010
Hemorrhagic Stroke	Development Center for Welfare and Health (STAKES) (Finland), World Health Organization (WHO). Finland WHO Multi-country Survey Study on Health and Health System Responsiveness 2000-2001. Geneva, Switzerland: World Health Organization (WHO)
Hemorrhagic Stroke	Gallup Europe, World Health Organization (WHO). Latvia WHO Multi-country Survey Study on Health and Health System Responsiveness 2000-2001. Geneva, Switzerland: World Health Organization (WHO)
Hemorrhagic Stroke	International Research Associates (INRA) Europe, Netherlands Organisation for Applied Scientific Research (TNO), World Health Organization (WHO). Netherlands WHO Multi-country Survey Study on Health and Health System Responsiveness 2000-2001. Geneva, Switzerland: World Health Organization (WHO)
Hemorrhagic Stroke	University of the West Indies, World Health Organization (WHO). Trinidad and Tobago WHO Multi-country Survey Study on Health and Health System Responsiveness 2000-2001. Geneva, Switzerland: World Health Organization (WHO)
Hemorrhagic Stroke	Börsch-Supan, A. (2015). Survey of Health, Ageing and Retirement in Europe (SHARE) Wave 5. Release version: 1.0.0. SHARE-ERIC. Data set. DOI: 10.6103/SHARE.w5.100
Hemorrhagic Stroke	Centre for Disease Prevention and Control (Latvia), Riga Stradi?š University. Latvia Health Behavior Among the Adult Population 2014
Hemorrhagic Stroke	Rastas S, Verkkoniemi A, Polvikoski T, Juva K, Niinisto L, Mattila K, Lansimies E, Pirttila T, Sulkava R. Atrial fibrillation, stroke, and cognition: a longitudinal population-based study of people aged 85 and older. Stroke. 2007; 38(5): 1454–60
Hemorrhagic Stroke	Ministry of Health of the Republic of Latvia. Latvia Hospital Inpatient Discharges 2004
Hemorrhagic Stroke	Ministry of Health of the Republic of Latvia. Latvia Hospital Inpatient Discharges 2006

Hemorrhagic Stroke	Ministry of Health of the Republic of Latvia. Latvia Hospital Inpatient Discharges 2007
Hemorrhagic Stroke	Ministry of Health of the Republic of Latvia. Latvia Hospital Inpatient Discharges 2008
Hemorrhagic Stroke	Ministry of Health of the Republic of Latvia. Latvia Hospital Inpatient Discharges 2010
Hemorrhagic Stroke	Ministry of Health of the Republic of Latvia, National Health Service (Latvia). Latvia Hospital Inpatient Discharges 2011
Hemorrhagic Stroke	Ministry of Health of the Republic of Latvia, National Health Service (Latvia). Latvia Hospital Inpatient Discharges 2012
Hemorrhagic Stroke	Institute of Public Health (Macedonia). Macedonia Hospital Inpatient Discharges 2000
Hemorrhagic Stroke	Institute of Public Health (Macedonia). Macedonia Hospital Inpatient Discharges 2001
Hemorrhagic Stroke	Institute of Public Health (Macedonia). Macedonia Hospital Inpatient Discharges 2002
Hemorrhagic Stroke	Institute of Public Health (Macedonia). Macedonia Hospital Inpatient Discharges 2004
Hemorrhagic Stroke	National Public Health Institute (Finland). Finland Hospital Discharge Register 1988
Hemorrhagic Stroke	National Public Health Institute (Finland). Finland Hospital Discharge Register 1989
Hemorrhagic Stroke	National Public Health Institute (Finland). Finland Hospital Discharge Register 1990
Hemorrhagic Stroke	National Public Health Institute (Finland). Finland Hospital Discharge Register 1991
Hemorrhagic Stroke	National Public Health Institute (Finland). Finland Hospital Discharge Register 1992
Hemorrhagic Stroke	National Public Health Institute (Finland). Finland Hospital Discharge Register 1993
Hemorrhagic Stroke	Ministry of Health of the Republic of Latvia. Latvia Hospital Inpatient Discharges 1980
Hemorrhagic Stroke	Institute of Public Health (Macedonia). Macedonia Hospital Inpatient Discharges 1980
Hemorrhagic Stroke	Dutch Hospital Data (DHD). Netherlands National Medical Registry 1990
Hemorrhagic Stroke	Dutch Hospital Data (DHD). Netherlands National Medical Registry 1991
Hemorrhagic Stroke	Dutch Hospital Data (DHD). Netherlands National Medical Registry 1992
Hemorrhagic Stroke	Dutch Hospital Data (DHD). Netherlands National Medical Registry 1993
Hemorrhagic Stroke	Dutch Hospital Data (DHD). Netherlands National Medical Registry 1994
Hemorrhagic Stroke	Dutch Hospital Data (DHD). Netherlands National Medical Registry 1995
Hemorrhagic Stroke	Dutch Hospital Data (DHD). Netherlands National Medical Registry 1996
Hemorrhagic Stroke	Dutch Hospital Data (DHD). Netherlands National Medical Registry 1997
Hemorrhagic Stroke	National Public Health Institute (Finland). Finland Hospital Discharge Register 1994
Hemorrhagic Stroke	National Public Health Institute (Finland). Finland Hospital Discharge Register 1995
Hemorrhagic Stroke	National Public Health Institute (Finland). Finland Hospital Discharge Register 1996
Hemorrhagic Stroke	National Public Health Institute (Finland). Finland Hospital Discharge Register 1997
Hemorrhagic Stroke	National Public Health Institute (Finland). Finland Hospital Discharge Register 1998
Hemorrhagic Stroke	National Public Health Institute (Finland). Finland Hospital Discharge Register 1999
Hemorrhagic Stroke	National Institute for Health and Welfare (THL) (Finland). Finland Hospital Discharge Register 2013
Hemorrhagic Stroke	National Institute for Health and Welfare (THL) (Finland). Finland Hospital Discharge Register 2014
Hemorrhagic Stroke	Ministry of Health of the Republic of Latvia. Latvia Hospital Inpatient Discharges 1981
Hemorrhagic Stroke	Ministry of Health of the Republic of Latvia. Latvia Hospital Inpatient Discharges 1982
Hemorrhagic Stroke	Ministry of Health of the Republic of Latvia. Latvia Hospital Inpatient Discharges 1983
Hemorrhagic Stroke	Ministry of Health of the Republic of Latvia. Latvia Hospital Inpatient Discharges 1984
Hemorrhagic Stroke	Ministry of Health of the Republic of Latvia. Latvia Hospital Inpatient Discharges 1985
Hemorrhagic Stroke	Ministry of Health of the Republic of Latvia. Latvia Hospital Inpatient Discharges 1986
Hemorrhagic Stroke	Ministry of Health of the Republic of Latvia. Latvia Hospital Inpatient Discharges 1987
Hemorrhagic Stroke	Ministry of Health of the Republic of Latvia. Latvia Hospital Inpatient Discharges 1988
Hemorrhagic Stroke	Ministry of Health of the Republic of Latvia. Latvia Hospital Inpatient Discharges 1989
Hemorrhagic Stroke	Ministry of Health of the Republic of Latvia. Latvia Hospital Inpatient Discharges 1990
Hemorrhagic Stroke	Ministry of Health of the Republic of Latvia. Latvia Hospital Inpatient Discharges 1991
Hemorrhagic Stroke	Ministry of Health of the Republic of Latvia. Latvia Hospital Inpatient Discharges 1992
Hemorrhagic Stroke	Ministry of Health of the Republic of Latvia. Latvia Hospital Inpatient Discharges 1993
Hemorrhagic Stroke	Ministry of Health of the Republic of Latvia. Latvia Hospital Inpatient Discharges 1994
Hemorrhagic Stroke	Ministry of Health of the Republic of Latvia. Latvia Hospital Inpatient Discharges 1995
Hemorrhagic Stroke	Ministry of Health of the Republic of Latvia. Latvia Hospital Inpatient Discharges 1996
Hemorrhagic Stroke	Ministry of Health of the Republic of Latvia. Latvia Hospital Inpatient Discharges 1997
Hemorrhagic Stroke	Ministry of Health of the Republic of Latvia. Latvia Hospital Inpatient Discharges 1998
Hemorrhagic Stroke	Ministry of Health of the Republic of Latvia. Latvia Hospital Inpatient Discharges 1999
Hemorrhagic Stroke	Ministry of Health of the Republic of Latvia. Latvia Hospital Inpatient Discharges 2000
Hemorrhagic Stroke	Ministry of Health of the Republic of Latvia. Latvia Hospital Inpatient Discharges 2001
Hemorrhagic Stroke	Ministry of Health of the Republic of Latvia. Latvia Hospital Inpatient Discharges 2002
Hemorrhagic Stroke	Ministry of Health of the Republic of Latvia. Latvia Hospital Inpatient Discharges 2003
Hemorrhagic Stroke	Ministry of Health of the Republic of Latvia, National Health Service (Latvia). Latvia Hospital Inpatient Discharges 2013
Hemorrhagic Stroke	Ministry of Health of the Republic of Latvia, National Health Service (Latvia). Latvia Hospital Inpatient Discharges 2014
Hemorrhagic Stroke	Institute of Public Health (Macedonia). Macedonia Hospital Inpatient Discharges 1982
Hemorrhagic Stroke	Institute of Public Health (Macedonia). Macedonia Hospital Inpatient Discharges 1984
Hemorrhagic Stroke	Institute of Public Health (Macedonia). Macedonia Hospital Inpatient Discharges 1985
Hemorrhagic Stroke	Institute of Public Health (Macedonia). Macedonia Hospital Inpatient Discharges 1986
Hemorrhagic Stroke	Institute of Public Health (Macedonia). Macedonia Hospital Inpatient Discharges 1987
Hemorrhagic Stroke	Institute of Public Health (Macedonia). Macedonia Hospital Inpatient Discharges 1989
Hemorrhagic Stroke	Institute of Public Health (Macedonia). Macedonia Hospital Inpatient Discharges 1990
Hemorrhagic Stroke	Institute of Public Health (Macedonia). Macedonia Hospital Inpatient Discharges 1991
Hemorrhagic Stroke	Institute of Public Health (Macedonia). Macedonia Hospital Inpatient Discharges 1992
Hemorrhagic Stroke	Institute of Public Health (Macedonia). Macedonia Hospital Inpatient Discharges 1993
Hemorrhagic Stroke	Institute of Public Health (Macedonia). Macedonia Hospital Inpatient Discharges 1995
Hemorrhagic Stroke	Institute of Public Health (Macedonia). Macedonia Hospital Inpatient Discharges 1996
Hemorrhagic Stroke	Institute of Public Health (Macedonia). Macedonia Hospital Inpatient Discharges 1997
Hemorrhagic Stroke	Institute of Public Health (Macedonia). Macedonia Hospital Inpatient Discharges 2012
